# Supplementary material for: Sequential immunotherapy and targeted therapy for metastatic BRAF V600 mutated melanoma: 4-year survival and biomarkers evaluation from the phase II SECOMBIT trial
Source: Nat Commun. 2024 Jan 2;15:146. doi: 10.1038/s41467-023-44475-6 (PMC10761671; doi:10.1038/s41467-023-44475-6)
Supplement: Supplementary file 1 — Supplementary information file [file 41467_2023_44475_MOESM1_ESM.pdf]

Supplementary Figure 1: CONSORT Diagram for SECOMBIT

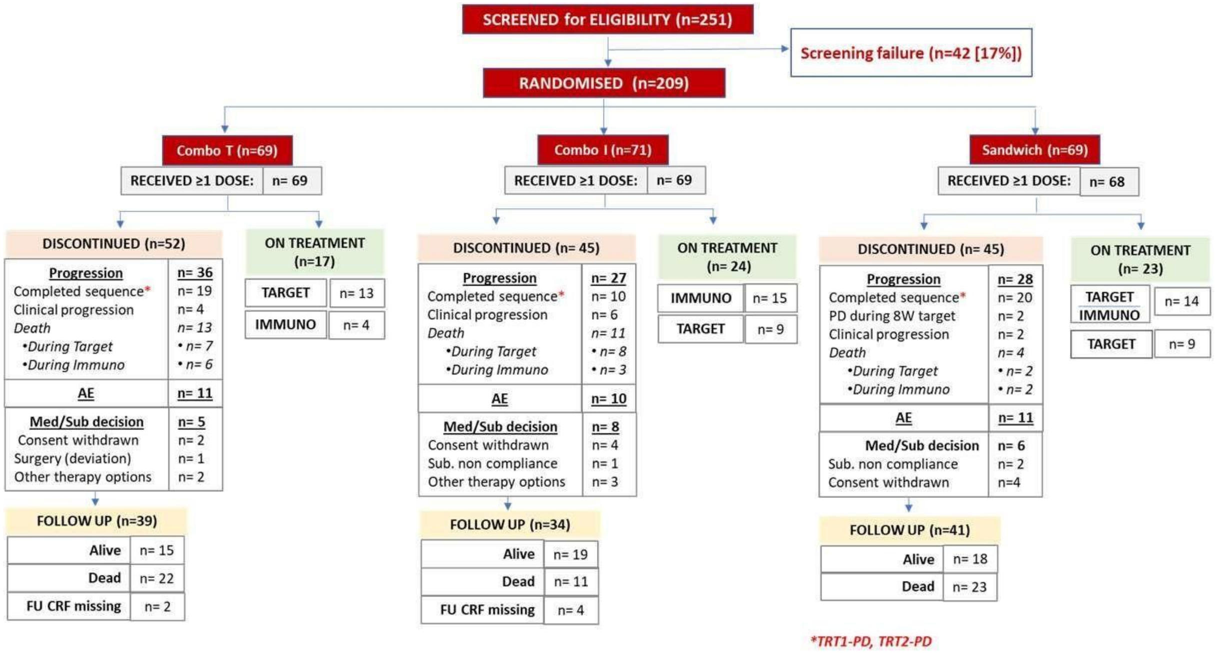

Supplementary Figure 2: TPFS and OS by Number Metastatic Sites, in Arm A (targeted therapy followed by immunotherapy), Arm B (immunotherapy followed by targeted therapy), and Arm C (a course of targeted therapy preceding immunotherapy and targeted therapy) of SECOMBIT

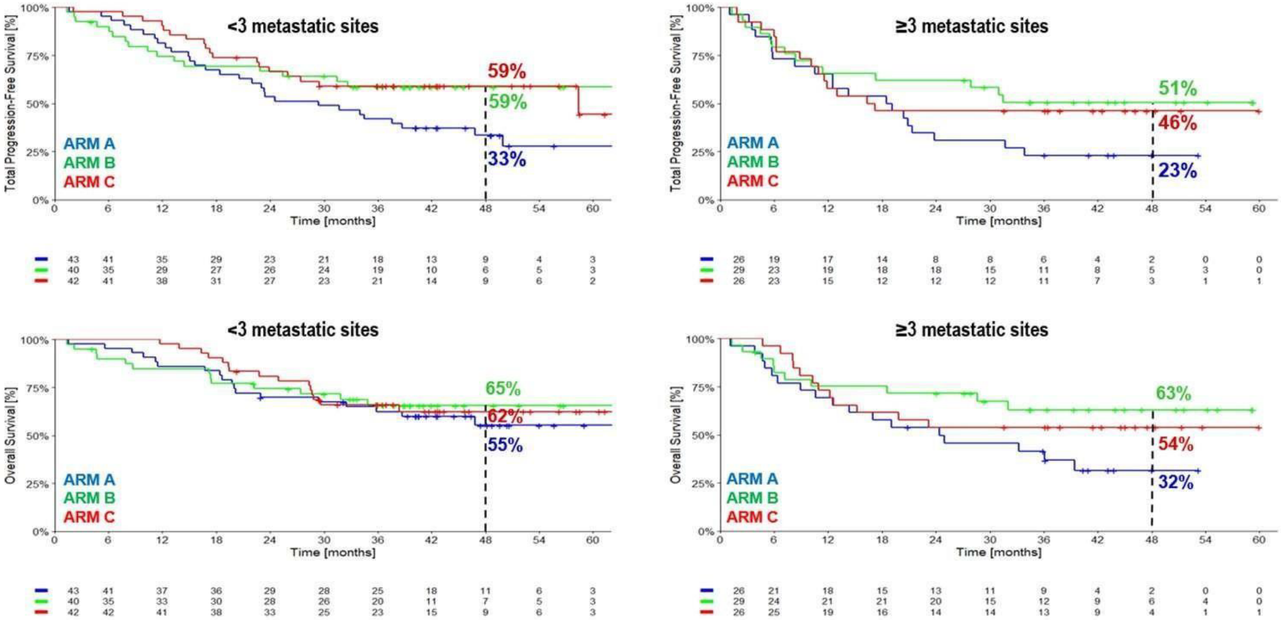

Supplementary Figure 3: TPFS and OS by LDH, in Arm A (targeted therapy followed by immunotherapy), Arm B (immunotherapy followed by targeted therapy), and Arm C (a course of targeted therapy preceding immunotherapy and targeted therapy) of SECOMBIT

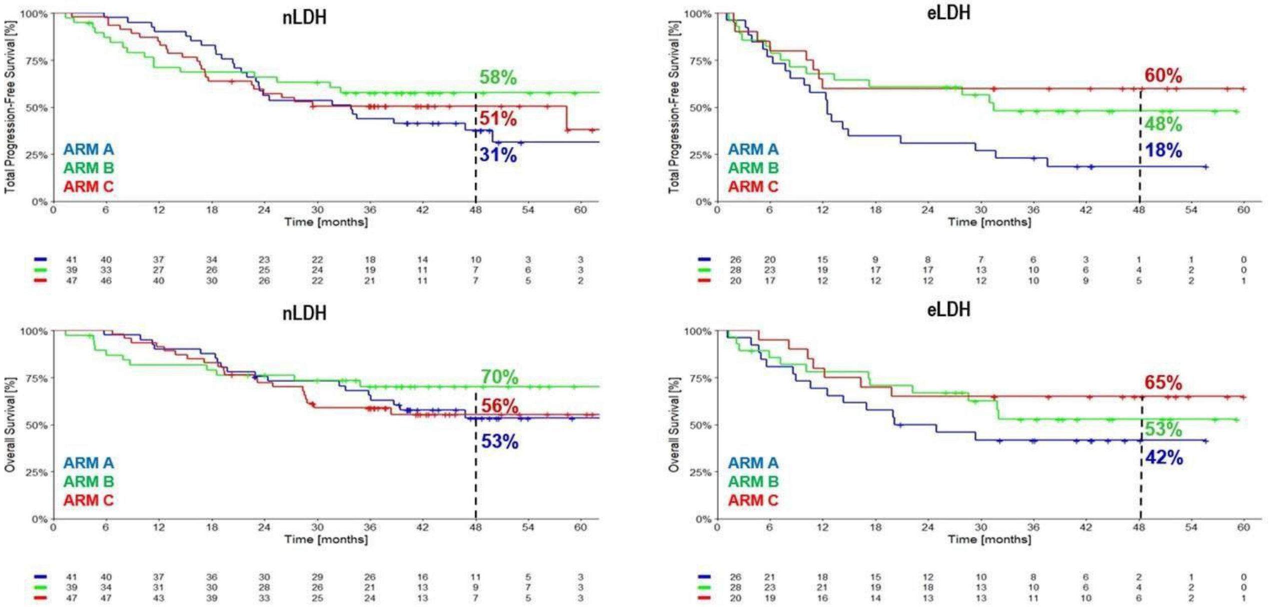

Supplementary Figure 4: OS by TMB in Arm A (targeted therapy followed by immunotherapy), Arm B (immunotherapy followed by targeted therapy), and Arm C (a course of targeted therapy preceding immunotherapy and targeted therapy) of SECOMBIT

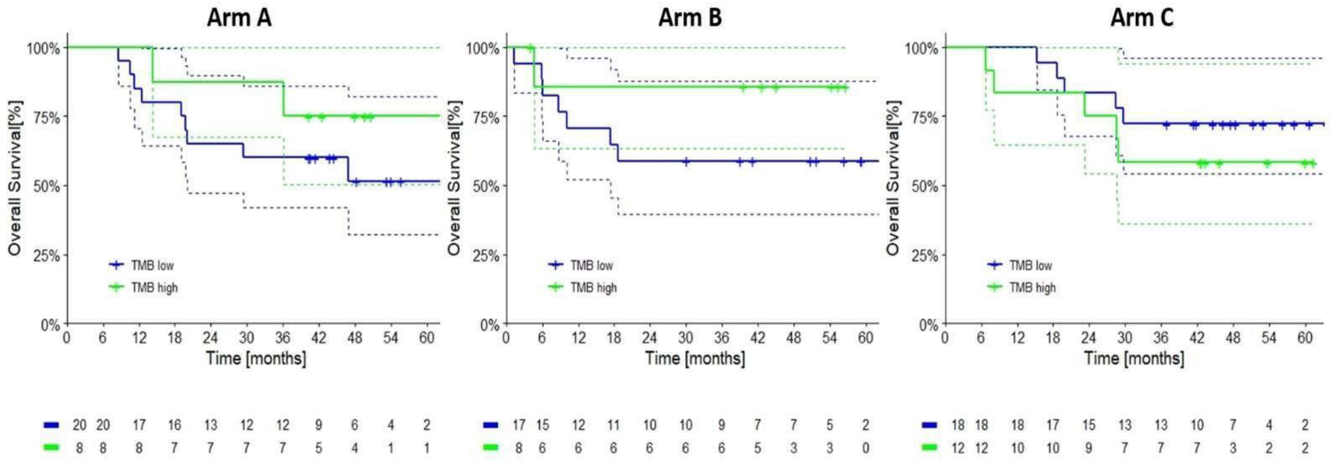

Supplementary Figure 5: MMP-9 (A) and IFN-α (B) expression in patients with either stable disease + progressive disease (SD+PD) or complete + partial response (CR+PR). Results are expressessed as p value by Mann-Whitney test. N=83

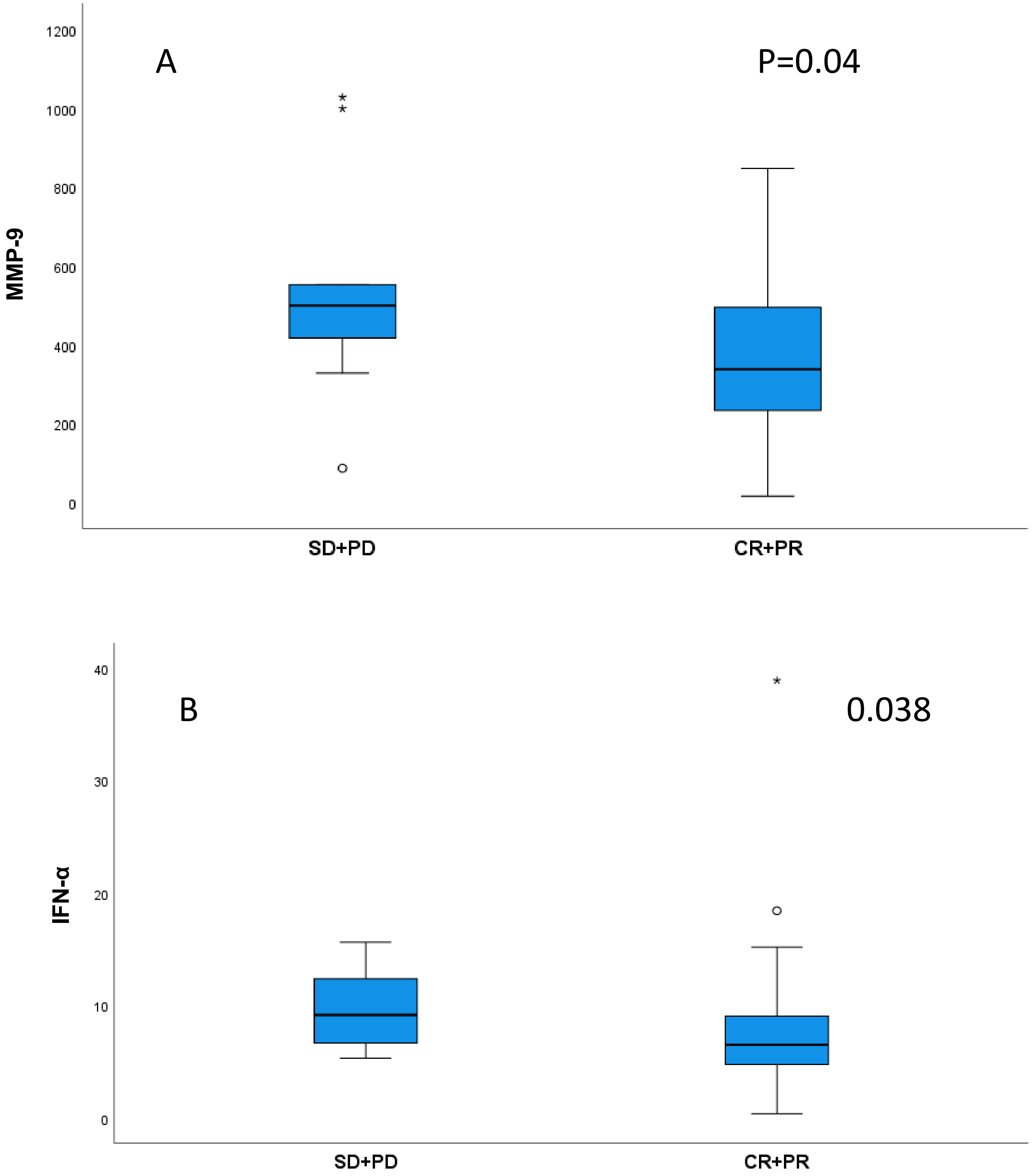

Supplementary Table 1. Significance of differences among arms in overall survival and TPFS. Values are p (log-rank test)

<table>
<tr><th>Arm</th><th>A</th><th>B</th><th>C</th></tr>
<tr><td colspan="4" align="center">Overall survival</td></tr>
<tr><td>A</td><td></td><td colspan="2" align="center">0.24</td></tr>
<tr><td>A</td><td></td><td align="center">0.13</td><td></td></tr>
<tr><td>A</td><td></td><td></td><td align="center">0.22</td></tr>
<tr><td>B</td><td></td><td></td><td align="center">0.73</td></tr>
<tr><td colspan="4" align="center">TPFS</td></tr>
<tr><td>A</td><td></td><td colspan="2" align="center">0.021</td></tr>
<tr><td>A</td><td></td><td align="center">0.017</td><td></td></tr>
<tr><td>A</td><td></td><td></td><td align="center">0.022</td></tr>
<tr><td>B</td><td></td><td></td><td align="center">0.90</td></tr>
</table>

Supplementary Table 2: PolyPhen-2 score focus in ARM B.

Patients	ARM	Gene	COSMIC ID	AA mutation	Amino Acid Change	PolyPhen-2 score	CDS mutation	FATHMM
**Pt 1**	B	JAK1	COSM1667603	**E897K**	**Glu897Lys**	**0.791**	2689G>A	0.94
**Pt 1**	B	JAK2	COSM8008807	**P279L**	**Pro279Leu**	**0.204**	836C>T	0.97
**Pt 1**	B	JAK3	COSM6932428	**E960K**	**Glu960Lys**	**0.77**	2878G>A	0.74
**Pt 2**	B	JAK1	COSM2239111	**G948R**	**Gly948Arg**	**1**	2842G>A	0.99
**Pt 3**	B	JAK1	COSM6916975	**M192I**	**Met192Ile**	**0.969**	576G>T	0.99
**Pt 3**	B	JAK1	COSM6997690	**F734L**	**Phe734Leu**	**0.999**	2200T>C	0.98
**Pt 3**	B	JAK1	COSM8245502	**Q387ter**	**Gln387ter**		**1159C>T**	0.98
**Pt 3**	B	JAK1	COSM3665161	**R1041Q**	**Arg1041Gln**	**0,805**	3122G>A	0.85
**Pt 3**	B	JAK1	COSM3790207	**R997W**	**Arg997Trp**	**0.267**	2989C>T	0.93
**Pt 3**	B	JAK2	COSM3907528	**P870S**	**Pro870Ser**	**0.997**	2608C>T	0.94
**Pt 3**	B	JAK3	COSM6980850	**T714M**	**Thr714Met**	**1**	2141C>T	0.99
**Pt 4**	B	JAK1	COSM911573	**R128H**	**Arg128His**	**0.839**	383G>A	1
**Pt 4**	B	JAK1	COSM6979549	**R1002Q**	**Arg1002Gln**	**1**	3005G>A	0.99
**Pt 4**	B	JAK1	COSM1255138	**V464M**	**Val464Met**	**0.999**	1390G>A	0.88
**Pt 4**	B	JAK1	COSM94556	**E637K**	**Glu637Lys**	**1**	1909G>A	0.97
**Pt 4**	B	JAK1	COSM7136807	**R755L**	**Arg755Leu**	**1**	2264G>T	0.96
**Pt 4**	B	JAK1	COSM7344136	**R809W**	**Arg809Trp**	**0.986**	2425C>T	0.92
**Pt 4**	B	JAK1	COSM1343904	**E927K**	**Glu927Lys**	**0.948**	2779G>A	0.98
**Pt 4**	B	JAK1	COSM9075612	**E1033D**	**Glu1033Asp**	**0.159**	3099G>T	0.86
**Pt 4**	B	JAK2	COSM303882	**D873N**	**Asp873Asn**	**0.998**	2617G>A	0.96
**Pt 4**	B	JAK2	COSM4384408	**R923C**	**Arg923Cys**	**1**	2767C>T	0.95
**Pt 4**	B	JAK3	COSM2157338	**P893L**	**Pro893Leu**	**0.648**	2678C>T	0.87
**Pt 5**	B	JAK1	**COSM911557**	**R755ter**	**Arg755ter**		2263C>T	0.9
**Pt 5**	B	JAK1	COSM8549647	**E501K**	**Glu501Lys**	**1**	1501G>A	0.91
**Pt 5**	B	JAK3	COSM3098964	**V963I**	**Val963Ile**	**0.908**	2887G>A	0.85
**Pt 6**	B	JAK1	COSM6980850	**T745M**	**Thr745Met**	**1**	2141C>T	0.99
**Pt 6**	B	JAK1	COSM9936736	**E459K**	**Glu459Lys**	**0.991**	1375G>A	0.97
**Pt 6**	B	JAK1	COSM7344128	**S407F**	**Ser407Phe**	**0,93**	1220C>T	0.98
**Pt 7**	B	JAK3	COSM5609194	**S44F**	**Ser44Phe**	**0.044**	131C>T	0.92
**Pt 7**	B	JAK1	COSM1667603	**E897K**	**Glu897Lys**	**0,791**	2689G>A	0.934039

Supplementary Note

To: ____________________

Subject: ____________________

SECOMBIT

"Sequential Combo Immuno and Target therapy (SECOMBIT) study"

"A three arms prospective, randomized phase II study to evaluate the best sequential approach with combo immunotherapy (ipilimumab/nivolumab) and combo target therapy (LGX818/MEK162) in patients with metastatic melanoma and BRAF mutation"

**Protocol Code:**	SECOMBIT
**EudraCT number**:	2014-004842-92
**Phase**:	II
**Sponsor:**	Fondazione Melanoma (ONLUS)<br>c/o Istituto Tumori Napoli - Fondazione "G. Pascale"<br>Via Mariano Semmola 80131 Naples, Italy
**Coordinating Investigator:**	Dr. Paolo Antonio Ascierto

Naples, 29 October 2021

This supplement contains the following items:

1. Original protocol v. 1.0 dated 15 October 2015, final protocol v. 10.0 dated 03 May 2021, summary of changes of intermediate versions,

2. Final statistical analysis plan v. 1.0 dated 22 October 2021.

Faithfully

Dr. Paolo A. Ascierto

STATISTICAL ANALYSIS PLAN

STUDY NUMBER: SECOMBIT

A three arms prospective, randomized phase II study to evaluate the best sequential approach with combo immunotherapy (ipilimumab/nivolumab) and combo target therapy (LGX818/MEK162) in patients with metastatic melanoma and BRAF mutation.

Sequential Combo Immuno and Target therapy (SECOMBIT) study

AUTHOR: Elizaveta Chefanova

VERSION NUMBER AND DATE: Version 1.0, 15Oct2021

APPROVAL SIGNATURE PAGE

Study Biostatistician:	Name: Elizaveta Chefanova<br>Title: Biostatistician		15 October 2021
	Signature		Date
Reviewer:	Name: Diana Giannarelli<br>Title: Senior Biostatistician		
			15 Oct 2021
	Signature		Date
Approved by:	Paolo Antonio Ascierto<br>Title: President of Fondazione Melanoma ONLUS		
			15 Oct 2021
	Signature		Date

Summary

1. List of Abbreviations 5
2. Introduction 8
3. Study Objectives 9
3.1. Primary Objective 9
3.2. Secondary Objectives 9
3.2.1. Biological markers (biomarkers study) 9
3.3. Safety Objectives 9
4. Study Design 9
4.1. Description of the study 9
4.2. Sample size 10
4.3. Description of treatments 10
4.4. Description of the study flow 10
4.5. Schedule of Events 12
4.6. Changes to Analysis from Protocol 12
5. Planned Analyses 12
5.1. Data Monitoring Committee (DMC) 12
5.2. Interim Analysis 12
5.3. Final Analysis 12
6. Major Protocol Deviations 13
7. Definition of Analysis Sets and Subgroups 14
8. General Considerations 15
8.1. Baseline 15
8.2. Windowing Conventions 15
8.3. Statistical Tests 15
8.4. Common Calculations 15
8.5. Software Version 16
9. Statistical Considerations 17
9.1. Adjustments for Covariates and Factors to be Included in Analyses 17
9.2. Multicenter Studies 17
9.3. Missing data 17
9.4. Multiple Comparisons/ Multiplicity 17
9.5. Active-Control Studies Intended to Show Non-inferiority or Equivalence 17
9.6. Examination of Subgroups 17
10. Output Presentations 18
11. Disposition and Withdrawals 19
12. Demographic and other Baseline Characteristics 20
12.1. Derivations 21
13. Medical History 22
14. Physical Examination 22
15. Systemic Adjuvant Treatments 22
16. Prior Radiation treatments 22
17. Prior Melanoma-Related Surgery 23
18. Medications 23
19. Study Medication Exposure and Compliance 23
19.1. Derivations 24
20. Efficacy Outcomes 24
20.1. Primary Efficacy 24
20.1.1. Primary Efficacy Variable & Derivation 24

20.1.2. Analysis of Primary Efficacy Variable 24
20.2. Secondary Efficacy 25
20.2.1. Secondary Efficacy Variables & Derivations....................25
20.2.2. Analysis of Secondary Efficacy Variables 26
21. Safety Outcomes 28
21.1. Adverse Events.................... 28
21.2. Laboratory Evaluations 29
21.3. Cardiological Assessments 33
21.4. Vital Signs.................... 33
22. Analysis of pharmacokinetic data.................... 34
23. Other assessments 34
23.1. Ophthalmological examination 34
23.2. Dermatological examination 35
23.3. Performance status 35
23.4. Pregnancy test 35
24. Table Shells and Specifications.................... 36
24.1. Table Specifications.................... 36
25. References 37
26. Tables and Listings Shells 40

1. LIST OF ABBREVIATIONS

AEs	Adverse Events
AESIs	Adverse Events of Special Interest
ALT	Alanine Transaminase (SGPT)
AST	Aspartate Transaminase (SGOT)
bid	Bis in die (twice daily)
BMI	Body Mass Index
BORR	Best Overall Response Rate
BRAF	B-raf murine sarcoma vioral oncogene homolog B1
BUN	Blood Urea Nitrogen
CI	Confidence Interval
CRP	C-reactive Protein
CT	Computed Tomography
DoR	Duration of Response
ECG	Electrocardiogram
EchO	Echocardiogram
ECOG	Eastern Cooperative Oncology Group
eCRF	Electronic Case Report Form
ENR	Enrolled Analysis Set
EORTC QLQ-C30	30-item European Organisation for Research and Treatment of Care quality of life questionnaire
EQ-5D	European Quality of Life 5-Dimensions
HGF	Hepatocyte Growth Factor
HRQoL	Health-related Quality of life
ICF	Informed Consent Form
IMP	Investigational Medicinal Product
INR	International Normalized Ratio
ITT	Intent-To-Treat Analysis Set
Kg	Kilogram
LDH	Lactate dehydrogenase
LVEF	Left Ventricle Ejection Fraction
MedDRA	Medical Dictionary for Regulatory Activities
MDSCs	Myeloid-derived Suppressor Cells
MEK	Methyl Ethyl Ketone
mg	Milligram
ml	Millilitre
MRI	Magnetic Resonance Imaging
MUGA	Multiple Gated Acquisition Scan

od	Once Daily
ORR	Overall Response Rate
OS	Overall Survival
PD	Progression of Disease
PFS	Progression-Free Survival
p.o.	Per os (oral route)
PP	Per Protocol Analysis Set
PS	Performance Status
RECIST	Response Evaluation Criteria In Solid Tumors
SAE	Serious Adverse Events
SAF	Safety Analysis Set
SAP	Statistical Analysis Plan
sCD25	Soluble interleukin-2 receptor
SECOMBIT	Sequential Combo Immuno and target Therapy
SNP	Single nucleotide polymorphism
sHGF	Soluble Hepatocyte Growth Factor
sVEGF	Serum Vascular Growth factor
T3	Triiodotyroxine
T4	Tyroxine
TPFS	Total Progression-Free Survival
TSH	Thyroid Stimulating Hormone
VAS	Visual Analog Scale
VEGF	Vascular Endothelial Growth Factor
WBC	White blood cell
WPAI:GH	Work Productivity and Activity Impairment: General Health
DMC	Data Monitoring Committee
FPFV	First Patient First Visit
LPLV	Last Patient Last Visit
C	Celsius
cm	centimetre
m	metre
SOC	System Organ Class
WHO-DRL	WHO-drug reference list
ATC	Anatomical Therapeutic Chemical
SAS	Statistical Analysis System
SDF	Survival Distribution Function
PFSR	Progression Free Survival Rate
SD	Standard Deviation

SE	Standard Error
RS	Raw Score
PT	Preferred Term
LLN	Lower Limit Of Normal
ULN	Upper Limit Of Normal
SI	International System of Units
CS	Clinically Significant
NCS	Not Clinically Significant
Min	The lowest observation
Max	The highest observation
HIV	Human Immunodeficiency Virus
Ms	Millisecond
mmHg	Millimeters of mercury
SBP	Systolic Blood Pressure
DBP	Diastolic Blood Pressure
bpm	beats per minute
ETDRS	Early Treatment Diabetic Retinopathy Study
OS	Left Eye
OD	Right Eye

2. INTRODUCTION

This document describes the statistical methods to be used during the analyses and reporting of study SECOMBIT. This SAP includes all details for the analysis and reporting (tables, listings and graph of the data collected as part of this protocol.

This Statistical Analysis Plan is based on the study protocol version Final 10.0, dated 03 May 2021.

3. Study Objectives

3.1. Primary Objective

The primary objective of this study is to define the best sequencing combination treatment in primary efficacy variable (Overall Survival, OS).

3.2. Secondary Objectives

The secondary objectives are to evaluate the effects of the three sequencing combination treatments on:

- Total Progression-Free Survival (PFS);
- 3 years PFS rate;
- Percentage of patients alive at 3 years;
- Best overall response rate (BORR);
- Duration of response (DoR);
- Toxicity of the investigational medicinal products (IMPs);
- Quality of life and general health status defined by:
    - Health-related quality of life (HRQoL), by means of the 30-item European Organisation for Research and Treatment of Care quality of life questionnaire (EORTC QLQ-C30);
    - General health status, by means of the European Quality of Life 5-Dimensions (EQ-5D) questionnaire;
    - Impairment of work productivity and activity, by means of the Work Productivity and Activity Impairment: General Health (WPAI:GH) questionnaire.

3.2.1. Biological markers (biomarkers study)

The objective of the biomarker study is to focus on understanding mechanisms of action/resistance. In particular, the biomarker study:

- Will inform how to sequence targeted RAF/MEK agents with immunotherapy agents (i.e., ipilumumab and nivolumab) in melanoma;
- Will be hypothesis-generating only.

3.3. Safety Objectives

The safety of the sequencing combination treatments will be evaluated, in terms of:

- Toxicity of the IMPs;
- Adverse events (AEs) and serious adverse events (SAEs);
- Vital signs;
- Laboratory safety parameters.

4. Study Design

4.1. Description of the study

This is an open-label, prospective, randomized, Phase II design study.

4.2. SAMPLE SIZE

This study was designed as a phase II, randomized trial with no formal comparative test. The sample size was discussed for the primary endpoint Overall Survival (OS).

For each arm a single-stage design as described by A'Hern (A'Hern, 2001) was used.

A median PFS of about 10 months for the combo target therapy (LGX818/MEK162) was assumed (Mc Arthur et al, 2013) and a similar value for the combo immunotherapy (ipilimumab/nivolumab) derived from the aggregate clinical activity rate of 65% (Wolchock et al, 2013) which, using an exponential distribution for PFS, could broadly give a median PFS of about 9.5 months. OS seems to be strictly correlated with total PFS.

The null hypothesis was a median OS time of 15 months (i.e. percentage of surviving patient of 33% at 24 months). The alternative hypothesis was a median OS time of 23 months (i.e. percentage of surviving of 48% at 24 months).

Using an exact 5% one-sided significance test, at least 69 patients would have to be randomized in each treatment arm when the power of the study is 80%.

For each arm the strategy would be further investigated if at least 30 patients, alive at 24 months, were observed.

Taking into account a 10% drop-out rate, a total of 230 patients would be enrolled to ensure a minimum of 207 randomized patients.

4.3. DESCRIPTION OF TREATMENTS

The following IMPs will be used in the study:

- Arm A: Combo Target (LGX818 450 mg p.o. od + MEK162 45 mg p.o. bid) until PD; then Combo Immuno (nivolumab 1 mg/kg solution i.v.combined with ipilimumab 3 mg/kg solution i.v.every 3 weeks for 4 doses then nivolumab 3 mg/kg solution i.v.every 2 weeks) until PD.
- Arm B: Combo Immuno (nivolumab 1 mg/kg solution i.v.combined with ipilimumab 3 mg/kg solution i.v.every 3 weeks for 4 doses then nivolumab 3 mg/kg solution i.v.every 2 weeks) until PD; then Combo Target (LGX818 450 mg p.o. od + MEK162 45 mg p.o. bid) until PD.
- Arm C: Combo Target (LGX818 450 mg p.o. od + MEK162 45 mg p.o. bid) for 8 weeks followed by Combo Immuno (nivolumab 1 mg/kg solution i.v.combined with ipilimumab 3 mg/kg solution i.v.every 3 weeks for 4 doses then nivolumab 3 mg/kg solution i.v.every 2 weeks) until PD; then Combo Target (LGX818 450 mg p.o. od + MEK162 45 mg p.o. bid) until PD.

4.4. DESCRIPTION OF THE STUDY FLOW

The study will be conducted according to an open-label, prospective, randomized, phase II design.

Randomization will be stratified according to stage arranged in the 3 following strata:

- IIIb/c – M1a – M1b;
- M1c with normal LDH (≤ 2ULN);

- M1c with elevated LDH (> 2 ULN).

All screening/baseline assessments as outlined in Tables 1 of the protocol (arm A), 2 (arm B) and 3 (arm C) of the Study Protocol must be performed within 28 days prior to the first administration of study drugs on Day 1. Results of tests or examinations performed as standard of care before obtaining informed consent and within the 28 days prior to commencing study drugs may be used. All assessments during the study must be performed within a window +/-3 days of the day indicated on the schedule of assessment, except for tumor evaluations for which a window of +/- 7 days will apply and for Combo Immuno visits for which a window of +/- 2 days will apply.

Eligibility for the study will be determined by the Investigator from the mandatory screening/baseline assessments performed during screening and according to the study inclusion/exclusion criteria.

First dosing of study drugs will be determined by the patient's eligibility and the laboratory assessments done on Day 1 prior to dosing.

Hematology and biochemistry assessments do not need to be repeated on Day 1 if performed within 7 days; if it is necessary to repeat these blood tests, the results must be known before the patient receives treatments to ensure inclusion/exclusion criteria related to these tests are met. Test for HIV infection is mandatory at screening.

Subject Re-enrollment: This study permits the re-enrollment of patients that was not randomized within 28 from ICF signature, after obtaining agreement from the medical monitor prior to re-enrolling a subject. If authorized to re-enrollment, the patient must be re-consented and the same subject-code can be used.

Subjects will be assessed for response by computed tomography (CT) or Magnetic Resonance Imaging (MRI). All measurable and non-measurable lesions must be documented at screening (within 28 days prior to randomization) and re-assessed at each subsequent tumor evaluation (every 8 weeks (+/- 1 week) for the first year, every 12 weeks (+/- 1 week) while the patient is on study). Tumor assessments with CT or MRI scans of the brain, chest, abdomen, and pelvis will be performed until disease progression after the second combo treatment per RECIST v1.1.

For patients who discontinue study treatment for reason other than investigator–determined disease progression, tumor assessments should continue to be performed as scheduled.

Patients will continue to be on study and can be switched to the subsequent therapy after PD as per protocol in both arm A and B.

In case of an interruption of the treatment in arm C, reason other than investigator–determined disease progression, during the first 8 weeks (2 cycles of therapy), patient can continue the study treatment with combo immuno, as scheduled.

For patients with palpable/superficial lesions, clinical disease assessments by physical examination should be performed at baseline and throughout study treatment as clinically indicated. Color photographs with ruler/calipers will be taken at baseline and at all subsequent tumor assessment time points.

The National Cancer Institute Common Toxicity Criteria for Adverse Events (NCI CTC-AE) Version 4.03 will be used to evaluate the clinical safety of the treatment in this study. Patients will be assessed for AEs at each clinical visit and as necessary throughout the study.

Biomarker study

A correlative biological study will be performed for the evaluation of biomarkers on the biological sample savailable (paraffin-embedded tissue, frozen tissue, blood, serum, etc.). Approximately 80-90 patients will take part in the biomarkerstudy.

4.5. Schedule of Events

Schedule of events can be found in Tables 1 of the protocol (arm A), 2 (arm B) and 3 (arm C) of the Section 1 of the study protocol.

4.6. Changes to Analysis from Protocol

No changes are done in statistical analysis from those planned in the final study protocol.

In this document more details about the multivariate statistical analyses have been added: for Cox's Proportional Hazards regression model on Overall Survival, total Progression Free Survival and Duration of Response the list of potential predictors have been detailed (see paragraph *9.1*).

5. Planned Analyses

Only the Final Analysis will be performed for this study.

5.1. Data Monitoring Committee (DMC)

In accordance with ICH E9 [1], Data Monitoring Committee is described in the protocol.

At least 1 database lock is planned for this study (final analysis), nonetheless, any additional DMC meetings can be conducted to assess safety and efficacy of the interventions during the clinical trial. All the necessary information will be further described in the relevant documents.

In any case, none of statistical tests will be applied to the data for DMC purposes.

5.2. Interim Analysis

There will be no Interim Analysis for this study.

5.3. Final Analysis

All final planned analyses identified in this SAP will be performed by SPARC Consulting, Milan on behalf of CRT after Sponsor Authorization of this Statistical Analysis Plan, Database Lock and Sponsor Authorization of Analysis Sets.

All summaries and listings will be performed using the SAS System version 9.4 or later under Windows 10 PRO operating system.

Continuous variables will be summarized by descriptive statistics (number of cases, mean, standard deviation, median, minimum, maximum, first and third quartile). Categorical variables will be summarized using counts of patients and percentages 95% confidence interval will be employed, unless otherwise specified.

6. MAJOR PROTOCOL DEVIATIONS

Major protocol deviations are protocol deviations that might significantly affect the completeness, accuracy, and/or reliability of the study data or that might significantly affect a subject's rights, safety, or well-being.

Major protocol deviations include:

- Any violations of Inclusion and/or Exclusion criteria;
- Incorrect dose of investigational product taken (Dose reduced/Dose delayed/Dose delayed and reduced) for more than 3 consecutive IMP cycles;
- Repeated or severe non-compliance to intake of investigational product (less than 80% or more than 120% of the prescribed dose of LGX818 and MEK162);
- Treatment with any other investigational agent at any time during the study;
- Randomized patient who received an incorrect treatment

The above major protocol deviations will be identified and confirmed prior to or at the Data Review Meeting at the latest and they will lead to the exclusion of a subject from the PP set.

Additional major protocol violations will be identified and documented following a data review meeting prior to database lock.

7. DEFINITION OF ANALYSIS SETS AND SUBGROUPS

Agreement and authorization of patients included/excluded from each analysis set will be performed on a case-by-case basis before the database lock.

The following analysis sets have been defined for this study:

Enrolled Analysis Set [ENR]

The all patients enrolled (ENR) set contains all patients who provided informed consent for this study.

Intent-to-Treat set [ITT]

The Intent-to-Treat (ITT) set will contain all randomized patients. Subjects in the ITT population will be analysed in the treatment group to which they were assigned by the randomization schedule, regardless of which study drug they receive.

The analysis of primary and secondary variables will be carried out in the ITT set.

Safety Analysis Set [SAF]

The Safety Analysis set (SAF) will contain all randomized subjects who received at least one dose of study medication. Subjects in the SAF will be analysed in the treatment group for the study drug they actually received.

The analysis of safety will be carried out in the safety population. If there is any doubt whether a subject was treated or not, they will be assumed treated for the purposes of analysis.

Per Protocol Analysis Set [PP]

The Per Protocol Analysis set (PP) will contain all randomized subjects who did not report any major protocol deviations (as detailed Paragraph 6).

The analysis on the primary variable (OS) will be repeated in the PP.

8. GENERAL CONSIDERATIONS

8.1. BASELINE

Baseline value is defined as the last available valid, non-missing observation for each subject before the first study treatment administration for each Treatment scheme. In this study Baseline 1, Baseline 2 and Baseline 3 visits will be accounted for depending on the context and on the analysis. The reference days for each baseline are described in Table 1.

Table 1 Reference days for Baseline

Arm	Treatment	Baseline	Reference Day
A	LGX818 + MEK 162	Baseline 1	Day 1 of TR1 [BAS 1]
	Nivolumab + Ipilimumab/ Nivolumab	Baseline 2 (one baseline for both treatments)	Day 1 of TR1 [BAS 2]
B	Nivolumab + Ipilimumab/ Nivolumab	Baseline 1 (one baseline for both treatments)	Day 1 of TR1 [BAS 1]
	LGX818 + MEK 162	Baseline 2	Day 1 of TR1 [BAS 2]
C	LGX818 + MEK 162	Baseline 1	Day 1 of TR1 [BAS 1]
	Nivolumab + Ipilimumab/ Nivolumab	Baseline 2 (one baseline for both treatments)	Day 1 of TR3 [BAS 2]
	LGX818 + MEK 162	Baseline 3	Day 1 of TR1 [BAS 3]

8.2. WINDOWING CONVENTIONS

All visit windows reported in the protocol will be applied to the analysis. The visit windows are described in more details for each assessment per Arm in Table 1 (Schedule of assessment) of the protocol [2]. Moreover, only values collected at scheduled study visits/time points will be presented in summary tables. However, additional exams may be scheduled as necessary to ensure the safety and well-being of subjects who experience AEs during the study. If a repeat assessment was performed, the result from the original assessment will be presented as the result at the specific visit/time point. All collected data will be included in the data listings. Additionally, unscheduled assessments will have the prefix “UNSCHEDULED” and will be sorted by the start date to its simpler recognizing.

8.3. STATISTICAL TESTS

No comparative tests between the three sequencing treatment arms will be performed and results will be presented as descriptive statistic.

8.4. COMMON CALCULATIONS

For quantitative measurements, change from baseline will be calculated with respect to the immediately previous baseline visit, i.e.:

- For values reported after Baseline 1 visit and before Baseline 2 visit, change from baseline will be calculated with respect to Baseline 1.
- For values reported after Baseline 2 visit (and before Baseline 3 visit, for Arm C), change from baseline will be calculated with respect to Baseline 2.
- For Arm C only, for values reported after Baseline 3, change from baseline will be calculated with respect to Baseline 3

The following formulas will be applied:

- Absolute: Value at Visit X – Baseline Value

- Relative: ((Value at Visit X – Baseline Value)/Baseline Value) *100

8.5. SOFTWARE VERSION

All statistical analyses will be performed using The SAS System version 9.4 or later under Windows 10 Pro operating system.

9. STATISTICAL CONSIDERATIONS

9.1. ADJUSTMENTS FOR COVARIATES AND FACTORS TO BE INCLUDED IN ANALYSES

The following adjustment for covariates is planned for this study.

The Cox's Proportional Hazards regression model on Overall Survival, Total Progression Free Survival and Duration of Response will include as covariates:

a) Treatment sequence assigned: Arm A, Arm B, Arm C;
b) Age (years): continuous variable;
c) Gender: Male and Female;
d) Melanoma Type: Cutaneous, Mucosal, Ocular;
e) Stage at current diagnosis: IIIa, IIIb, IIIc, IV – M1a, IV – M1b, IV – M1c;
f) Baseline LDH level: continuous variable;
g) ECOG PS at baseline: 0, ≥1;

9.2. MULTICENTER STUDIES

This study will be conducted by multiple investigators at multiple centers (approximately 30 sites in Italy and Europe) [2]. Prior to statistical analysis, data obtained at all sites will be combined into one data set.

Due to a large number of sites in this study and a small number of patients in this site, statistical tests by site will not be performed.

9.3. MISSING DATA

Any Missing data will not be imputed (including efficacy data).

9.4. MULTIPLE COMPARISONS/ MULTIPLICITY

Not applicable for this study.

9.5. ACTIVE-CONTROL STUDIES INTENDED TO SHOW NON-INFERIORITY OR EQUIVALENCE

Not applicable for this study.

9.6. EXAMINATION OF SUBGROUPS

No subgroup analyses will be performed for this study.

10. OUTPUT PRESENTATIONS

Appendix 1 shows conventions for presentation of data in outputs.

The templates provided with this SAP describe the presentations for this study and therefore the format and content of the summary tables, figures and listings to be provided by SPARC Consulting, Milan on behalf of CRT.

11. Disposition and Withdrawals

All subjects who provide informed consent [ENR set] will be accounted for in this study.
The following summary tables and listings will be provided:

- A summary table of patients enrolled in the study by site per Arm (including non-randomized patients)
- A summary with the number and percentage of the screening failures and of the subjects who did not satisfy inclusion/exclusion criteria, together with a data listing containing screening failures and details of the non-satisfied criteria.
- A summary with the number and percentage of the subjects included in the analysis populations (ENR, ITT, SAF, PP) and of the reasons for exclusion from ITT, SAF and PP by treatment arm. A listing of reasons for exclusion from the analysis populations will be provided.
- A frequency table and a listing of major protocol deviations
- A frequency table and a listing of end of treatment data:
    - Completion of the trial (Yes, No)
    - Primary reason for withdrawal (Progression of disease, Death, withdrawn informed consent for the study and for FU, withdrawn informed consent for the study but NOT for FU, Subject no longer meets study criteria (after the first PD), Screening Failure, AE, Poor/Non-compliance with protocol requirements, Loss of follow-up, Administrative reason by Sponsor, Subject's decision, Other)

12. DEMOGRAPHIC AND OTHER BASELINE CHARACTERISTICS

Demographic data and other baseline characteristics will be presented for the ITT set by treatment arm.
No statistical testing will be carried out for demographic or other baseline characteristics.
The following demographic and other baseline characteristics will be reported for this study by means of default summary statistics and individual data listings:

- Demographic variables:
  - Age (years) – derived as specified in Paragraph 12.1
  - Gender at birth (Male, Female)
  - Race (White/Caucasian, Black/African American, Asian, American Indian or Alaska native, Native Hawaiian or Pacific Islander, Other)
  - Ethnic Origin (Hispanic/Latino, Not Hispanic/Latino, Mixed Ethnicity, Other)
- Vital Signs at screening:
  - Body weight (Kg)
  - Height (cm)
  - BMI (kg/m^2)
  - Systolic supine blood pressure (mmHg)
  - Diastolic supine blood pressure (mmHg)
  - Heart rate (bpm)
  - Body temperature (°C)
  - Respiratory rate (breaths/min)
- ECOG PS
  - 0 = Fully active, able to carry on all pre-disease performance without restriction,
  - 1 = Restricted in physically strenuous activity but ambulatory and able to carry out work of a light or sedentary nature, e.g., light house work, office work,
  - 2 = Ambulatory and capable of all selfcare but unable to carry out any work activities. Up and about more than 50% of waking hours,
  - 3 = Capable of only limited selfcare, confined to bed or chair more than 50% of waking hours,
  - 4 = Completely disabled. Cannot carry on any selfcare. Totally confined to bed or chair,
  - 5 = Dead.

  Only numbers (from 0 to 5) will be reported in tables and listings.
- Disease History of Melanoma: Initial diagnosis
  - Melanoma Type (Cutaneous, Mucosal, Ocular, Unknown)
    - If mucosal, primary site of mucosal melanoma (Head and neck, Oesophagus, Stomach, Small Intestine, Colon, Rectum, Vagina, Bladder, Other)
    - If cutaneous, primary site of cutaneous melanoma (Head, Neck, Trunk, Legs, Arms, Buttocks, Other)
  - Melanoma subtype (Superficial spreading, Lentigo maligna, Nodular, Desmoplastic, Unknown, Other)
  - Time from first diagnosis of melanoma to study enrolment (months) – derived as specified in paragraph 12.1
  - Stage at initial diagnosis (0, I, Ia, Ib, Ic, II, IIa, IIb, IIc, III, IIIa, IIIb, IIIc, IV, Iva, IVb, IVc)

- Disease History of Melanoma: Current diagnosis
  - Stage at current diagnosis (IIIa, IIIb, IIIc, IV – M1a, IV – M1b, IV – M1c)
  - Recurrence/Relapse (Yes/No)
    - If yes, time from first recurrence/relapse (months) – derived as specified in paragraph 12.1
    - If yes, time from most recent recurrence/relapse (months) – derived as specified in paragraph 12.1
- Molecular status (local) mutant (Yes, No)

  If yes, type of mutation (Exon 15 V600E, Exon 15 V600K, Exon 15 V600D, Exon 15 V600R, Other)
- Childbearing potential status and pregnancy test:
  - Childbearing potential (Yes, No)
  - Post-menopausal (Yes, No)
  - Accepted to use an adequate contraception for the total study duration (Yes, No)
  - Serum pregnancy test (if applicable) done (Yes, No) and result (Positive, Negative).
- HIV test:
  - Performed (Yes, No, Not applicable)
  - Result (Negative, Positive)

12.1. DERIVATIONS

The following variables will be derived:

- Age (years) = integer [(Date of informed consent – "15/Jul/year of birth") / 365.25]
- Time from first diagnosis of melanoma to study enrolment (months) = [(Date of informed consent – date of first diagnosis of melanoma)/30.4]

  If Date of first diagnosis of melanoma is partial, the following conventions will be used:
  - If day is unknown, but month and year are known, use first day of the month;
  - If day and month are unknown, but year is known, use 01 January;
  - If day, month and year are unknown, leave it missing.
- Time from first recurrence/relapse to study enrolment (months) = [(Date of informed consent – date of first recurrence/relapse)/30.4]

  If Date of first recurrence/relapse is partial, the following conventions will be used:
  - If day is unknown, but month and year are known, use 15 as day;
  - If day and month are unknown, but year is known, use 01 July;
  - If day, month and year are unknown, leave it missing.
- Time from most recent recurrence/relapse to study enrolment (months) = [(Date of informed consent – date of most recent recurrence/relapse)/30.4]

  If Date of most recent recurrence/relapse is partial, the following conventions will be used:
  - If day is unknown, but month and year are known, use 15 as day;
  - If day and month are unknown, but year is known, use 01 July;
  - If day, month and year are unknown, leave it missing.

13. Medical History

Medical history information will be presented for the ITT set.

Medical history verbatims will be coded using MedDRA dictionary version 20.0 or higher.

Medical conditions with "Ongoing" field not ticked in eCRF will be considered as previous diseases while those reported as "Ongoing" in eCRF will be considered as concomitant diseases.

Previous and concomitant diseases will be analyzed separately with frequency tables reporting the number of patients who exhibited at least one disease and showing diseases by primary System Organ Class and Preferred Term by treatment arm.

Line listings of previous and concomitant diseases will be produced for the ITT set.

14. Physical Examination

Physical examination information will be presented for the ITT set.

Physical examination verbatims will be derived from the eCRF "Physical Examination" form in the "If abnormal, specify" field and will be coded using MedDRA dictionary version 20.0 or higher.

Clinically significant and not clinically significant abnormalities will be analyzed separately with frequency tables reporting the number of patients who exhibited at least one abnormality and showing abnormalities by primary System Organ Class and Preferred Term by treatment arm.

A line listing of physical examinations and significant abnormalities will be produced for the ITT set.

15. Systemic Adjuvant Treatments

Systemic adjuvant treatments will be presented for the ITT set and coded using WHO-DRL dictionary version 2017 or higher.

Prior systemic treatments are those treatments with "Ongoing" field not ticked and which stopped prior to the first dose of study medication.

Concomitant systemic treatments are those reported as "Ongoing" in the "Prior systemic treatment" eCRF form.

Frequency tables of prior and concomitant systemic treatments by primary therapeutic subgroup (3rd level ATC level subgroup) and generic name will be presented by treatment arm.

Line listings of prior and concomitant systemic treatments will be produced.

16. Prior Radiation treatments

Prior radiation treatments will be presented for the ITT set and coded using WHO-DRL dictionary version 2017 or higher.

A frequency table of prior radiation treatments by primary therapeutic subgroup (3rd level ATC level subgroup) and generic name will be presented by treatment arm.

A line listing will be produced for the ITT set.

17. PRIOR MELANOMA-RELATED SURGERY

Prior (melanoma-related) surgeries will be presented for the ITT set and coded using WHO-DRL dictionary version 2017 or higher.

A frequency table of prior surgeries by primary therapeutic subgroup (3rd level ATC level subgroup) and generic name will be presented by treatment arm.

A line listing will be produced for the ITT set.

18. MEDICATIONS

Medications will be presented for the ITT set and coded using WHO-DRL dictionary version 2017 or higher.

Prior medications are those which stopped prior to the first dose of study medication.

Concomitant medications are those which:

- Started prior to, on or after the first dose of study medication and started no later than date of last study dose,
- AND ended on or after the date of first dose of study medication or were ongoing at the date of last study dose.
- If the date of last study dose is unknown the medication will be considered as concomitant.

Also, see Table 9 for handling of partial dates for medications, in the case where it is not possible to define a medication as prior or concomitant, the medication will be classified by the worst case.

The following tables will be presented:

- A frequency tables of prior medications by primary therapeutic subgroup (3rd level ATC level subgroup) and generic name by treatment arm.
- A frequency tables of concomitant medications by primary therapeutic subgroup (3rd level ATC level subgroup) and generic name by treatment arm.

Data listings of prior and concomitant medications will be produced.

19. STUDY MEDICATION EXPOSURE AND COMPLIANCE

Exposure in days and compliance to oral IMPs (LGX818 and MEK162) will be presented for the SAF set.

The date of first study drug administration will be taken from the eCRF “LGX818 (450 mg p.o od)”, “MEK162 (45 mg p.o. bid)”, “Nivolumab 1 mg/kg solution IV”, “Ipilimumab 3 mg/kg solution IV” taking the “Start therapy date” which occurs first.

The date of last dose of study medication will be taken from the eCRF “End of Treatment” form (“Date when the investigational product was last taken”). If this date is missing, the date of last study medication will be taken from the eCRF “LGX818 (450 mg p.o od)”, “MEK162 (45 mg p.o. bid)”, “Nivolumab 1 mg/kg solution IV”, “Ipilimumab 3 mg/kg solution IV” taking the “End therapy date” which occurs last.

Temporarily interruptions and dose changes are not considered for overall duration of exposure.

Default summary statistics will be presented for overall duration of exposure and duration of exposure to each study drug by treatment arm (see specifications for derivations in Section 19.1). A line listing of duration of exposure will be produced.

Compliance to oral IMPs (LGX818 and MEK162) will be summarized with default statistics by treatment arm. A line listing will be produced.

19.1. Derivations

Overall duration of exposure [weeks]: [(date of last dose – date of first study drug administration + 1) / 7].

Duration of exposure to LGX818 [weeks]: sum over all cycles [(end therapy date – start therapy date + 1) / 7].

Duration of exposure to MEK162 [weeks]: sum over all cycles [(end therapy date – start therapy date + 1) / 7].

Duration of exposure to Nivolumab [weeks]: sum over all cycles [(end therapy date – start therapy date + 1) / 7]. For this calculation consider study periods with 1 mg/kg and 3 mg/kg IV infusion.

Duration of exposure to Ipilimumab [weeks]: sum over all cycles [(end therapy date – start therapy date + 1) / 7].

Compliance to LGX818 [%] = Total dose administered of LGX818 over all cycles / (Total daily dose to be administered as per protocol x duration of exposure to LGX818 [days]) x 100. Note that total daily dose to be administered as per protocol is 450 mg.

Compliance to MEK162 [%] = Total dose administered of MEK162 over all cycles / (Total daily dose to be administered as per protocol x duration of exposure to MEK162 [days]) x 100. Note that total daily dose to be administered as per protocol is 90 mg.

20. Efficacy Outcomes

20.1. Primary Efficacy

20.1.1. Primary Efficacy Variable & Derivation

OS is the primary endpoint of the study. OS will be calculated from the date of randomization until the date of death from any cause. Any patient not known to have died at the time of data analysis will be censored at the time of the last recorded date on which the patient was known to be alive.

OS (months) = [(Date of death or last contact – date of randomization + 1)/30.4]

20.1.2. Analysis of Primary Efficacy Variable

The analysis will be performed in the ITT set and in the PP set.

The estimate of the survivor function using the Kaplan-Meier approach will be displayed graphically by treatment arm. Median time and the corresponding two-sided 95% confidence interval will be presented together with the estimates for the other quartiles (SAS procedure PROC LIFETEST) by treatment arm separately.

SAS-code for Kaplan-Meier approach[3]:

```
PROC LIFETEST DATA = <input dataset> PLOTS=SURVIVAL(ATRISK(OUTSIDE(0.15)))
  OUTSURV= <output dataset> CONFTYPE=LOGLOG;
```

```
  TIME <OS time in months>* <flag with censored data>(0)1;
  STRATA <treatment group>;
RUN;

DATA <new dataset>;
  SET <output dataset>;
  UCL = ROUND(100*(1-SDF_UCL),.1);
  LCL = ROUND(100*(1-SDF_LCL),.1);
RUN;
```

A Cox's proportional hazard regression model will also be performed to assess the influence of some baseline covariates on OS. Covariates of interest are those reported in section 9.1. Hazard ratios estimates with their 95% confidence limits will be also presented (SAS procedure PROC PHREG).

SAS-code for Cox's proportional hazard regression model [4,5]:

```
PROC PHREG DATA = <adam.dataset>;
   CLASS <arm>(REF=FIRST) <gender>(REF=<'MALE'>) <melanoma type>(REF=FIRST) <stage of
diagnosis>(REF=FIRST) <ECOG PS>(REF=FIRST) / PARAM=REF;
   MODEL <duration of OS in months>*<censoring>(0) = <arm> <age> <gender> <melanoma type>
<stage of diagnosis> <LDH level> <ECOG PS> / TIES=EFRON RISKLIMITS;
RUN;
```

20.2. SECONDARY EFFICACY

The secondary analyses will be performed in the ITT set.

20.2.1. SECONDARY EFFICACY VARIABLES & DERIVATIONS

The secondary efficacy endpoints are:

a) Total PFS, calculated from the date of randomization until the date of the second progression (i.e., the progression to second treatment); any progression or death will be considered as an event if patient cannot complete treatment sequence.
   Any patient not known to have experienced progression or died at the time of data analysis will be censored at the time of the last recorded date on which the patient was known to be alive without disease progression.
   Total PFS (months) = [(Date of second progression or any progression/death or last contact – date of randomization +1)/30.4]
b) 3 years PFS rate (PFSR), calculated from the date of randomization until the date of progression or death from any cause. This rate estimates the proportion of patients who did not progress and were alive 3 years after randomization.
c) Percentage of patients alive at 2 and 3 years;
d) Best overall response rate (BORR), defined as the best response designation, as determined by the investigator, recorded between the date of randomization and the date of objectively documented progression per RECIST version 1.1 criteria;
e) Duration of response (DoR), calculated as the time from the date of first documented response (CR or PR) until the date of the first documented progression or death due to

[1] *where 0=censored; 1 = event.*

underlying cancer. If a patient with a CR or PR has no progression or death due to underlying cancer, the patient is censored at the date of last adequate tumor assessment;
DoR (months) = [(Date of first documented progression/death due to underlying cancer or date of last tumor assessment – date of first CR or PR +1)/30.4]

f) Health-related quality of life (HRQoL), by means of the 30-item European Organisation for Research and Treatment of Care quality of life questionnaire (EORTC QLQ-C30);
g) General health status, by means of the European Quality of Life 5-Dimensions (EQ-5D) questionnaire;
h) Impairment of work productivity and activity, by means of the Work Productivity and Activity Impairment: General Health (WPAI:GH) questionnaire.

20.2.2. ANALYSIS OF SECONDARY EFFICACY VARIABLES

The secondary efficacy analyses will be performed in ITT set.

The following statistical methods will be applied on the secondary efficacy endpoints:

a) Total PFS: The estimates of the survivor function using the Kaplan-Meier approach will be displayed graphically by treatment arm. Median time and the corresponding two-sided 95% confidence interval will be presented together with the estimates for the other quartiles (SAS procedure PROC LIFETEST) by treatment arm separately.
A Cox's proportional hazard regression model will also be performed to assess the influence of some baseline covariates on total PFS. Covariates of interest are those reported in Paragraph 9.1. Hazard ratios estimates with their 95% confidence limits will be also presented (SAS procedure PROC PHREG).
b) 3-years PFSR: The PFSR at 3 years will be estimated by the Kaplan-Meier method. The 95% confidence intervals for the PFSR will be estimated using Greenwood's estimate of the standard error (SE) and a linear transformation of the progression-free survival function.
c) Percentage of patients alive at 2 and 3 years will be reported using Wilson score intervals for each treatment arm.
d) BORR: Default summary statistics of the best overall response from the date of randomization to the date of objectively documented progression per RECIST version 1.1 criteria will be presented, by treatment arm.
For each treatment arm, the Overall Response Rate (ORR) will be calculated as the percentage of patients in the ITT set who have a CR or PR before any evidence of progression (as defined by RECIST).
A 95% confidence interval (CI) will be derived for the ORR using Wilson score intervals (CIs for a single proportion).
e) The estimates of the survivor function of DoR using the Kaplan-Meier approach will be displayed graphically by treatment arm. Median time and the corresponding two-sided 95% confidence interval will be presented together with the estimates for the other quartiles (SAS procedure PROC LIFETEST) by treatment arm separately.
A Cox's proportional hazard regression model will also be performed to assess the influence of some baseline covariates on DoR. Covariates of interest are those reported in Paragraph 9.1. Hazard ratios estimates with their 95% confidence limits will be also presented (SAS procedure PROC PHREG).
f) Default summary statistic of all scale scores of the EORTC QLQ-C30 and of the changes from baseline of each scale will be presented by treatment arm, according to the following scoring method.

For all the scales, the Raw Score (RS) is the mean of the component items:

$$RS = (I_1 + I_2 + \dots + I_n)/n.$$

A linear transformation to standardise the Raw Scores is used, so that scores range from 0 to 100; a higher score represents a higher ("better") level of functioning, or a higher ("worse") level of symptoms.

For Functional Scales, the transformation to be used is the following:

$$Score = \left\{1 - \frac{(RS-1)}{range}\right\} \times 100$$

where range is the difference between the possible maximum and the minimum response to individual items.

For Symptoms scales/items and Global health status / QoL the transformation is the following:

$$Score = \left\{\frac{(RS-1)}{range}\right\} \times 100$$

The component items and the range which define each scale score are reported in the following table.

Table 2 Component items and range for EORTC QLQ-C30 scoring

	Scale	Number of items	Item range	Item numbers
**Global health status / QoL**	QL2	2	6	29,30
**Functional scales**				
Physical functioning	PF2	5	3	1 to 5
Role functioning	RF2	2	3	6, 7
Emotional functioning	EF	4	3	21 to 24
Cognitive functioning	CF	2	3	20, 25
Social functioning	SF	2	3	26, 27
**Symptom scales / items**				
Fatigue	FA	3	3	10, 12, 18
Nausea and vomiting	NV	2	3	14, 15
Pain	PA	2	3	9, 19
Dyspnoea	DY	1	3	8
Insomnia	SL	1	3	11
Appetite loss	AP	1	3	13
Constipation	CO	1	3	16
Diarrhoea	DI	1	3	17
Financial difficulties	FI	1	3	28

g) The EQ-5D questionnaire consists of 5 5-levels items (mobility, self-care, usual activities, pain/discomfort, anxiety/depression) and of the EQ Visual Analogue Scale (EQ VAS). The digits for 5 dimensions can be combined in a 5-digit number describing the respondent's health state (11111 indicates no problems, while 55555 indicates severe problems in all the 5 dimensions). Missing values can be coded as 9. Ambiguous values should be treated as missing values. Health states will be converted into a single index values by using the UK TTO value set. Default summary statistics and changes from baseline of the EQ-5D index will be presented by treatment arm.

h) The following percentages will be calculated for each patient at each of the foreseen administrations. Default summary statistics and changes from baseline of these 4 scores will be presented by treatment arm.

- Percent work time missed due to health: Q2/(Q2+Q4) x 100;
- Percent impairment while working due to health: Q5 x 10;

- Percent overall work impairment due to health: {Q2/(Q2+Q4) + [(1-(Q2/(Q2+Q4))) x (Q5/10)]} x 100
- Percent activity impairment due to health: Q6 x 10.

21. SAFETY OUTCOMES

All outputs for safety outcomes will be based on the Safety Analysis Set (SAF).

Safety and tolerability will be assessed in terms of AEs, laboratory data (haematology, blood chemistry, urinalysis), ECG data, vital signs (heart rate and blood pressure), BMI and weight, which will be collected for all patients.

21.1. ADVERSE EVENTS

All adverse events (AEs) reported on eCRF and experienced by the patient on or after the informed consent date will be considered in the analysis. Thus, there will not be derivation to Treatment-emerged Adverse Events (TEAE) and not TEAE.

Adverse event Investigator terms will be assigned to a Preferred Term (PT) and will be classified by primary System Organ Class (SOC) according to the Medical Dictionary for Regulatory Activities (MedDRA) thesaurus, version 20.0 or higher

Counts and percentages will be presented by primary System Organ Class (SOC) and Preferred Term (PT) as defined in MedDRA thesaurus, by treatment arm.

A patient having more than one AE with the same PT will be counted only once in the incidence calculation for that PT. Similarly, if a patient has more than one AE in the same SOC, the patient will be counted only once in the total number of patients with an AE for that SOC.

An AE will be defined “Related” if causality is “Certain”, “Probable” or “Possible”.

The following frequency distributions of adverse events will be provided by treatment arm:

- An overview of AEs including the number of patients with:
  - at least one AE,
  - at least one non-serious AE,
  - at least one AE related to Ipilimumab,
  - at least one AE related to Nivolumab,
  - at least one AE related to Combo Immuno Therapy,
  - at least one AE related to LGX818,
  - at least one AE related to MEK162,
  - at least one AE related to Combo Target Therapy,
  - at least one serious AE (SAE),
  - at least one SAE related to Ipilimumab,
  - at least one SAE related to Nivolumab,
  - at least one SAE related to Combo Immuno Therapy,
  - at least one SAE related to LGX818,
  - at least one SAE related to MEK162,
  - at least one SAE related to Combo Target Therapy,
  - at least one AE leading to hospitalization,
  - at least one AE leading to withdrawal from the study,

- at least on AESI,

and the number of AEs, non-serious AEs, SAEs, AEs related to Ipilimumab, AEs related to Nivolumab, AEs related to Combo Immuno Therapy, AEs related LGX818, AEs related to MEK162, AEs related to Combo Target Therapy, SAEs related to Ipilimumab, SAEs related to Nivolumab, SAEs related to Combo Immuno Therapy, SAEs related LGX818, SAEs related to MEK162, SAEs related to Combo Target Therapy, AEs leading to hospitalization, AEs leading to death, AEs leading to study withdrawal, AESIs;

- AEs presented by Primary SOC and PT;
- AEs related to Ipilimumab presented by Primary SOC and PT;
- AEs related to Nivolumab presented by Primary SOC and PT;
- AEs related to Combo Immuno Therapy presented by Primary SOC and PT;
- AEs related to LGX818 presented by Primary SOC and PT;
- AEs related to MEK162 presented by Primary SOC and PT;
- AEs related to Combo Target Therapy presented by Primary SOC and PT;
- Serious AEs presented by Primary SOC and PT;
- AEs leading to hospitalization presented by Primary SOC and PT;
- AEs leading to death presented by Primary SOC and PT;
- AEs leading to study withdrawal presented by Primary SOC and PT.
- AESIs presented by Primary SOC and PT

The following individual data listings will be produced:

- A listing of all AEs;
- A listing of AEs related to Ipilimumab;
- A listing of AEs related to Nivolumab;
- A listing of AEs related to Combo Immuno Therapy;
- A listing of AEs related to LGX818;
- A listing of AEs related to MEK162;
- A listing of AEs related to Combo Target Therapy;
- A listing of all SAEs;
- A listing of all AEs leading to death;
- A listing of AEs leading to hospitalization;
- A listing of AEs leading to study withdrawal;
- A listing of AESIs.

21.2. LABORATORY EVALUATIONS

Laboratory data will be analyzed on the SAF set.

Descriptive summaries of hematology, blood chemistry, coagulation, thyroid function, Cardiac Muscle Enzyme and endocrine panel will be provided for actual values and changes from baseline at each visit by treatment arm.

Default frequency tabulations will be also provided for urinalysis by treatment group for each visit.

Line listings of laboratory data during the study will be provided.

Presentations for continuous variables will use SI Units: conversion will be provided using the International Laboratory Normal Ranges (SI Unit Conversion Guide, M. Laposata, NEJM, 1992).

To convert from the conventional unit to the SI unit, laboratory specific derivations will be used, multiply by the conversion factor.

Table 3- Hematology: conversion factors

Component	Conventional Unit	Conversion Factor	SI Unit
Hemoglobin	g/dL	10	g/L
Hematocrit	%	0.01	Proportion of 1.0
RBC Count	X 10^6/µl	1	X 10^{12}/L
WBC Count	X 10^3/µl	1	X 10^9/L
Neutrophils	X 10^3/µl	1	X 10^9/L
Lymphocytes	X 10^3/µl	1	X 10^9/L
Monocytes	X 10^3/µl	1	X 10^9/L
Eosinophils	X 10^3/µl	1	X 10^9/L
Basophils	X 10^3/µl	1	X 10^9/L
Platelets	X 10^3/µl	1	X 10^9/L

Table 4- Biochemistry: conversion factors

Component	Conventional Unit	Conversion Factor	SI Unit
Glucose	mg/dL	0.05551	mmol/L
Sodium	mEq/L	1	mmol/L
Potassium	mEq/L	1	mmol/L
	mg/dL	0.2558	
Calcium	mEq/L	0.5	mmol/L
	mg/dL	0.250	
Chloride	mEq/L	1	mmol/L
Bicarbonate	mEq/L	1	mmol/L
Magnesium	mg/dL	0.4114	mmol/L
Urea	mg/dL	0.167	mmol/L

BUN	mg/dL	0.357	mmol/L
Uric Acid	mg/dL	59.48	µmol/L
Albumin	g/dL	10	g/L
Creatinine	mg/dL	88.4	µmol/L
Creatinine Clearance	mL/min	0.01667	mL/s
Total Proteins	g/dL	0.01	g/L
Total Bilirubin	mg/dL	17.104	µmol/L
Indirect Bilirubin	mg/dL	17.104	µmol/L
Alkaline phosphatase	units/L	1	U/L
Alanine aminotransferase (ALT)	units/L	1	U/L
Aspartate aminotransferase (AST)	units/L	1	U/L
Gamma-GT	units/L	1	U/L
HDL Cholesterol	mg/dL	0.02586	mmol/L
LDL Cholesterol	mg/dL	0.02586	mmol/L
LDH	Units/L	1	U/L
	Units/L	0.01667	µkat/L
Amylase	Units/L	1	U/L
	Units/L	0.01667	µkat/L
Lipase	Units/L	1	U/L
	Units/L	0.01667	µkat/L

Table 5- Coagulation: conversion factors

Component	Conventional Unit	Conversion Factor	SI Unit
INR	Ratio	1	ratio

aPTT	Seconds	1	seconds
PTT	Seconds	1	seconds
Protrombin time	Seconds	1	seconds
Fibrinogen	g/dL	29.41	µmol/L
	mg/dL	0.01	g/L

Table 6 – Thyroid function test: conversion factors

Component	Conventional Unit	Conversion Factor	SI Unit
TSH	µU/mL	1	mU/L
Free T4	µg/dL	12.87	nmol/L
Free T3	ng/dL	0.01536	nmol/L

Table 7 – Cardiac Muscle Enzyme: conversion factors

Component	Conventional Unit	Conversion Factor	SI Unit
Creatine Kinase	µkat/L	59.988	U/L
	ng/dL	100	ng/mL
Troponin	ng/L	1000	
	µg/L	1	
Creatinin Kinase Isoenzyme	%	0.01	Fraction of 1.00
Myoglobin	ng/mL	17.513	nmol/L

Table 8 – Endocrine panel: conversion factors

Component	Conventional Unit	Conversion Factor	SI Unit
ACTH	pg/mL	0.2202	pmol/L
Total cortisol	µg/dL	27.59	nmol/L

The following parameters will be collected for urinalysis:

- Glucose (Neg, Trace, 1+, 2+, 3+, 4+)
- Protein (Neg, Trace, 1+, 2+, 3+, 4+)
- Blood (Neg, Trace, 1+, 2+, 3+, 4+)
- Bilirubin (Neg, Trace, 1+, 2+, 3+, 4+)
- Ketones (Neg, Trace, 1+, 2+, 3+, 4+)
- Leukocytes (Neg, Trace, 1+, 2+, 3+, 4+)
- UWBC/HPF (Not present, Present) and continuous value if present
- URBC/HPF (Not present, Present) and continuous value if present
- Casts/LPF (Not present, Present) and continuous value if present
- Bacteria (Not present, Present) and continuous value if present
- Other (Not present, Present) and continuous value if present

The following summaries will be provided for urinalysis data by treatment arm:

- Actual and change from baseline by visit for continuous values of UWBC/HPF, URBC/HPF, Casts/LPF, Bacteria and Other, if present;
- Frequency tables by visit for glucose, protein, blood, bilirubin, ketones, leukocytes, UWBC/HPF, URBC/HPF, Casts/LPF, Bacteria and Other.

21.3. CARDIOLOGICAL ASSESSMENTS

ECG and ECHO/MUGA parameters will be analysed on the SAF set.

Descriptive summaries of PR interval (ms), QT interval (ms), QRS interval (ms), QTcB interval (ms), cardiac axis (°), Ejection Fraction Value (%) will be provided for actual values and changes from baseline at each visit by treatment arm.

Default frequency tabulations will be also provided for ECG result (Normal, Abnormal) and interpretation of abnormal ECG (not clinically significant, clinically significant), type of LVEF scan (ECHO, MUGA) and ECHO/MUGA scan interpretation (Normal, Clinically insignificant abnormality, Clinically significant abnormality) by treatment arm.

Line listings of ECG and ECHO/MUGA parameters during the study will be provided.

21.4. VITAL SIGNS

Vital signs parameters will be analyzed on the SAF set.

Descriptive summaries of the following parameters will be provided for actual values and changes from baseline at each visit by treatment arm:

- Body weight (Kg)
- BMI (kg/m2)
- Systolic Blood Pressure (mmHg)
- Diastolic Blood Pressure (mmHg)
- Heart Rate (bpm)
- Body Temperature (°C)

- Respiratory rate (breaths/min)

A line listing of vital signs during the study will be provided.

22. ANALYSIS OF PHARMACOKINETIC DATA

Not applicable.

23. OTHER ASSESSMENTS

23.1. OPHTHALMOLOGICAL EXAMINATION

Ophthalmological examinations will be analyzed on the SAF set.

According to study protocol, this examination will be performed at each baseline visit (Baseline 1, Baseline 2 and Baseline 3 – for Arm C only).

Descriptive summaries of the following parameters will be provided for each visit by treatment arm:

- Ophthalmological examination performed (No, Yes, Not Applicable);
- Eye(s) assessed (Left Eye, Right Eye, Both);
- Visual Acuity assessed (No, Yes):
  - Total Visual Acuity Score (ETDRS) – Left Eye (OS),
  - Total Visual Acuity Score (ETDRS) – Right Eye (OD),
  - Total Visual Acuity snellens equivalent – Left Eye (OS),
  - Total Visual Acuity snellens equivalent – Right Eye (OD);
- Intraocular pressure assessed (No, Yes):
  - Intraocular pressure – Left eye (OS) (mmHg),
  - Intraocular pressure – Right eye (OD) (mmHg),
- Dilated fundoscopy performed (No, Yes):
  - Eye(s) assessed (Left eye, Right eye, Both),
  - For each eye, assessment (Normal, abnormal) of the following areas:
    - Viterous
    - Retina
    - Macula
    - Choroid
    - Optic nerve pallor
    - Other
- Slit lamp examination performed (No, Yes):
  - Eye(s) assessed (Left eye, Right eye, Both),
  - For each eye, assessment (Normal, Abnormal) of the following areas:
    - Lids/lashes
    - Cornea
    - Conjunctiva
    - Iris
    - Lens
    - Anterior chamber

  - Other
- Visual field testing performed (No, Yes) and Abnormalities (No, Yes);
- Optical coherence tomography performed (No, Yes) and abnormalities (No, Yes).

A line listing of the ophthalmological examinations during the study will be provided.

23.2. DERMATOLOGICAL EXAMINATION

Dermatological examinations will be analyzed on the SAF set.

According to study protocol, this examination will be performed at each baseline visit and every 8 weeks.

Descriptive summaries of the following parameters will be provided for each visit by treatment arm:

- Dermatological examination performed (No, Yes);
- Result of the dermatological examination (Normal, Abnormal);
- If Results is Abnormal, Diagnosis (Squamous cell carcinoma, Keratoacanthomas, Other pathology findings)
- Evidence of severe or uncontrolled systemic disease or concurrent undesirable condition (No, Yes)

A line listing of dermatological examinations during the study will be provided.

23.3. PERFORMANCE STATUS

A frequency table and a line listing of ECOG PS during the study will be provided.

23.4. PREGNANCY TEST

A frequency table and a corresponding listing of pregnancy test result during the study will be provided. Pregnancy test is performed at baseline 1, then every 6 weeks during study treatment, at Follow up visit and at the long-term follow-up visit.

24. TABLE SHELLS AND SPECIFICATIONS

24.1. TABLE SPECIFICATIONS

Tables will be provided as defined by the table shells.

Similar tables based on different populations will have the same number, except for the last digit.

All output will be generated by SAS and exported into a RTF format and then convert to PDF format. All output will be in landscape orientation. Font size will be Courier New 8 pt.

The header containing the sponsor' name (Fondazione Melanoma (ONLUS)) and protocol number will appear on the top left corner of each page of the output. The page number, in the format of "Page x of y", will appear on the top right corner of the output, where y = last page of corresponding output.

Column headers in tables include the total possible numbers to be included in summaries for that table, designated as "(N=XX)".

The SAS program name, and the date and time of the creation of the output (run date) will appear on the bottom left corner as follows:

Source: [program name].sas, Run on ddmmyyyy

Table Format Specification:

Maximum and minimum values will be reported with the same number of decimal places as collected. Means and medians will be reported to one additional decimal place. Standard deviations and standard errors will be reported to two decimal places more than the collected data. Percentages will be reported with one decimal place.

Data in the tables are formatted as follows:

- Text fields in the body of the tables and listings will be left-justified.
- When no data are available for a table, an empty page with the title will be produced with suitable text. Example: THERE WERE NO SERIOUS ADVERSE EVENTS.

25. REFERENCES

1. ICH. Statistical Principles for Clinical Trials, Guideline E9 [Electronic resource]. 1998.
2. Nazionale I. et al. Clinical Study Protocol (SECOMBIT). 2021.
3. SAS. SAS/STAT ® 14.2 User's Guide The LIFETEST Procedure. 2016.
4. Yu LI, Cheng L. How to Compute Custom Hazard Ratio in SAS (V8 . 2 & V9 . 2) PHREG Procedure. P. 1–8.
5. SAS. User ' s 14.3 Guide The PHREG Procedure. 2017.

APPENDIX 1. PROGRAMMING CONVENTIONS FOR OUTPUTS

DATES & TIMES

Depending on data available, dates and times will take the form ddmmyyyy hh:mm.

SPELLING FORMAT

English US.

LISTINGS

All listings will be ordered by the following (unless otherwise indicated in the template):

- Treatment Arm
- Site ID
- Patient ID
- Visit
- Date (where applicable).

APPENDIX 2. PARTIAL DATE CONVENTIONS

Imputed dates will NOT be presented in the listings.

Table 9 Algorithm for Prior / Concomitant Medications

START DATE	STOP DATE	ACTION
Known	Known	If stop date < study med start date, assign as prior<br>If stop date >= study med start date and start date <= end of treatment, assign as concomitant<br>If stop date >= study med start date and start date > end of treatment, assign as post study
	Partial	Impute stop date as latest possible date (i.e. last day of month if day unknown or 31st December if day and month are unknown), then:<br>If stop date < study med start date, assign as prior<br>If stop date >= study med start date and start date <= end of treatment, assign as concomitant<br>If stop date >= study med start date and start date > end of treatment, assign as post treatment
	Missing	If stop date is missing could never be assumed a prior medication<br>If start date <= end of treatment, assign as concomitant<br>If start date > end of treatment, assign as post treatment
Partial	Known	Impute start date as earliest possible date (i.e. first day of month if day unknown or 1st January if day and month are unknown), then:<br>If stop date < study med start date, assign as prior<br>If stop date >= study med start date and start date <= end of treatment, assign as concomitant<br>If stop date >= study med start date and start date > end of treatment, assign as post treatment
	Partial	Impute start date as earliest possible date (i.e. first day of month if day unknown or 1st January if day and month are unknown) and impute stop date as latest possible date (i.e. last day of month if day unknown or 31st December if day and month are unknown), then:<br>If stop date < study med start date, assign as prior<br>If stop date >= study med start date and start date <= end of treatment, assign as concomitant<br>If stop date >= study med start date and start date > end of treatment, assign as post treatment
	Missing	Impute start date as earliest possible date (i.e. first day of month if day unknown or 1st January if day and month are unknown), then:<br>If stop date is missing could never be assumed a prior medication<br>If start date <= end of treatment, assign as concomitant<br>If start date > end of treatment, assign as post treatment
Missing	Known	If stop date < study med start date, assign as prior<br>If stop date >= study med start date, assign as concomitant<br>Cannot be assigned as 'post treatment'
	Partial	Impute stop date as latest possible date (i.e. last day of month if day unknown or 31st December if day and month are unknown), then:<br>If stop date < study med start date, assign as prior<br>If stop date >= study med start date, assign as concomitant<br>Cannot be assigned as 'post treatment'
	Missing	Assign as concomitant

26. TABLES AND LISTINGS SHELLS

In the following pages tables and listings shells are presented.

TABLE 1. Patients enrolment by site 45
TABLE 2. Summary of screening failures 46
TABLE 3. Summary of Major Protocol Deviations 47
TABLE 4. Summary of patients' disposition 48
TABLE 5. Analysis sets 49
TABLE 6. Demographics 50
TABLE 7. Vital Signs and ECOG PS at screening 51
TABLE 8. Disease History of Melanoma: Initial diagnosis 53
TABLE 9. Disease History of Melanoma: Current diagnosis 54
TABLE 10. Molecular status 55
TABLE 11. Childbearing potential status and pregnancy test at screening 56
TABLE 12. HIV test at screening 57
TABLE 13. Summary of medical history - concomitant diseases 58
TABLE 14. Summary of medical history - previous diseases 59
TABLE 15. Summary of clinically significant physical examination abnormalities 60
TABLE 16. Summary of not clinically significant physical examination abnormalities 61
TABLE 17. Summary of prior systemic treatments 62
TABLE 18. Summary of concomitant systemic treatments 63
TABLE 19. Summary of prior radiation treatments 64
TABLE 20. Summary of prior melanoma-related surgeries 65
TABLE 21. Summary of prior medications 66
TABLE 22. Summary of concomitant medications 67
TABLE 23. Study medication exposure 68
TABLE 24. Compliance to oral IMPs (LGX818 and MEK162) 69
TABLE 25. Analysis of Primary Endpoint: Summary of Overall Survival (OS) 70
TABLE 26. Analysis of Primary Endpoint: Predictive factors of Overall Survival (months) 73
TABLE 27. Analysis of Secondary Endpoint: Summary of Total Progression Free Survival (TPFS) 77
TABLE 28. Analysis of Secondary Endpoint: Predictive factors of Total Progression Free Survival 80
TABLE 29. Analysis of Secondary Endpoint: 3-years Progression Free Survival Rate (PFSR) 82
TABLE 30. Analysis of Secondary Endpoint: Percentage of patients alive at 2 and 3 years 83
TABLE 31. Analysis of secondary endpoint: Best Overall Response (BOR) and Overall Response Rate (ORR) 84
TABLE 32. Analysis of Secondary Endpoint: Summary of Duration of Response (DoR) 85
TABLE 33. Analysis of Secondary Endpoint: Predictive factors of Duration of Response 88

TABLE 34. Analysis of Secondary Endpoint: Change from baseline of Health-Related Quality of Life (HRQoL) by means of EORTC-QLQ-C30 – ARM A....... 90
TABLE 35. Analysis of Secondary Endpoint: Change from baseline of Health-Related Quality of Life (HRQoL) by means of EORTC-QLQ-C30 – ARM B....... 92
TABLE 36. Analysis of Secondary Endpoint: Change from baseline of Health-Related Quality of Life (HRQoL) by means of EORTC-QLQ-C30 – ARM C....... 93
TABLE 37. Analysis of Secondary Endpoint: Change from baseline of EQ-5D-5L by means of EQ-5D index – ARM A....... 96
TABLE 38. Analysis of Secondary Endpoint: Change from baseline of EQ-5D-5L by means of EQ-5D index – ARM B....... 97
TABLE 39. Analysis of Secondary Endpoint: Change from baseline of EQ-5D-5L by means of EQ-5D index – ARM C....... 98
TABLE 40. Analysis of Secondary Endpoint: Change from baseline of Impairment of work productivity and activity by means of WPAI:GH – ARM A....... 101
TABLE 41. Analysis of Secondary Endpoint: Change from baseline of Impairment of work productivity and activity by means of WPAI:GH – ARM B....... 103
TABLE 42. Analysis of Secondary Endpoint: Change from baseline of Impairment of work productivity and activity by means of WPAI:GH – ARM C....... 105
TABLE 43. Summary of adverse events....... 108
TABLE 44. Incidence of AEs by primary System Organ Class and Preferred Term....... 110
TABLE 45. Incidence of Ipilimumab-related AEs by primary System Organ Class and Preferred Term....... 111
TABLE 46. Incidence of Nivolumab-related AEs by primary System Organ Class and Preferred Term....... 112
TABLE 47. Incidence of Combo Immuno Therapy-related AEs by primary System Organ Class and Preferred Term....... 113
TABLE 48. Incidence of LGX818-related AEs by primary System Organ Class and Preferred Term....... 114
TABLE 49. Incidence of MEK162-related AEs by primary System Organ Class and Preferred Term....... 115
TABLE 50. Incidence of Combo Target Therapy-related AEs by primary System Organ Class and Preferred Term....... 116
TABLE 51. Incidence of Serious AEs by primary System Organ Class and Preferred Term 117
TABLE 52. Incidence of AEs leading to hospitalization by primary System Organ Class and Preferred Term....... 118
TABLE 53. Incidence of AEs leading to study withdrawal by primary System Organ Class and Preferred Term....... 119
TABLE 54. Incidence of AESIs by primary System Organ Class and Preferred Term....... 120
TABLE 55. Incidence of AEs leading to death by primary System Organ Class and Preferred Term....... 121
TABLE 56. Hematology – ARM A....... 122
TABLE 57. Hematology – ARM B....... 124
TABLE 58. Hematology – ARM C....... 125
TABLE 59. Blood chemistry – ARM A....... 128
TABLE 60. Blood Chemistry – ARM B....... 130
TABLE 61. Blood Chemistry – ARM C....... 132
TABLE 62. Coagulation – ARM A....... 135
TABLE 63. Coagulation – ARM B....... 137

TABLE 64. Coagulation – ARM C ... 138
TABLE 65. Thyroid function test – ARM A... 141
TABLE 66. Thyroid function test – ARM B... 143
TABLE 67. Thyroid function test – ARM C... 145
TABLE 68. Cardiac Muscle Enzymes – ARM A ... 148
TABLE 69. Cardiac Muscle Enzymes – ARM B ... 150
TABLE 70. Cardiac Muscle Enzymes – ARM C ... 151
TABLE 71. Endocrine Panel – ARM A ... 154
TABLE 72. Endocrine Panel – ARM B ... 155
TABLE 73. Endocrine Panel – ARM C ... 156
TABLE 74. Urinalysis (categorical parameters – Part I) – ARM A ... 158
TABLE 75. Urinalysis (categorical parameters – Part I) – ARM B ... 160
TABLE 76. Urinalysis (categorical parameters – Part I) – ARM C ... 163
TABLE 77. Urinalysis (categorical parameters – Part II) – ARM A ... 167
TABLE 78. Urinalysis (categorical parameters – Part II) – ARM B ... 168
TABLE 79. Urinalysis (categorical parameters – Part II) – ARM C ... 169
TABLE 80. Urinalysis (continuous parameters) – ARM A ... 171
TABLE 81. Urinalysis (continuous parameters) – ARM B ... 173
TABLE 82. Urinalysis (continuous parameters) – ARM C ... 175
TABLE 83. ECG Parameters – ARM A... 178
TABLE 84. ECG Parameters - ARM B... 180
TABLE 85. ECG Parameters – ARM C... 181
TABLE 86. Cardiological Assessments – ARM A... 183
TABLE 87. Cardiological Assessments – ARM B... 184
TABLE 88. Cardiological Assessments – ARM C... 185
TABLE 89. Vital signs – ARM A ... 186
TABLE 90. Vital signs – ARM B ... 188
TABLE 91. Vital signs – ARM C ... 190
TABLE 92. Ophthalmological Examinations during the study ... 193
TABLE 93. Visual Acuity during the study ... 195
TABLE 94. Intraocular pressure during the study... 198
TABLE 95. Dilated fundoscopy during the study ... 200
TABLE 96. Slit lamp examination during the study ... 203
TABLE 97. Visual field testing and optical coherence tomography during the study207
TABLE 98. Dermatological examination during the study – ARM A... 209
TABLE 99. Dermatological examination during the study – ARM B... 210
TABLE 100. Dermatological examination during the study – ARM C ... 211
TABLE 101. Performance Status during the study – ARM A... 212
TABLE 102. Performance Status during the study – ARM B... 213
TABLE 103. Performance Status during the study – ARM C... 214
TABLE 104. Pregnancy test during the study... 215
Listing 1. Screening Failures ... 216
Listing 2. Inclusion Criteria ... 217
Listing 3. Exclusion Criteria ... 218

Listing 4. End of treatment ... 219
Listing 5. Analysis Sets ... 220
Listing 6. Major Protocol Deviations ... 221
Listing 7. Enrollment visit and signature of informed consent ... 222
Listing 8. Demographic Characteristics ... 223
Listing 9. Disease History of Melanoma: Initial Diagnosis ... 224
Listing 10. Disease History of Melanoma: Current Diagnosis ... 225
Listing 11. BRAF mutation testing and molecular status ... 226
Listing 12. Childbearing potential status ... 227
Listing 13. HIV test at screening ... 228
Listing 14. Medical History: previous diseases ... 229
Listing 15. Medical History: concomitant diseases ... 230
Listing 16. Prior Systemic Treatments ... 231
Listing 17. Concomitant Systemic Treatments ... 232
Listing 18. Prior Radiation Treatments ... 233
Listing 19. Prior melanoma-related surgery ... 234
Listing 20. Serum/urine pregnancy test ... 235
Listing 21. Vital signs and ECOG PS ... 236
Listing 22. Physical Examination ... 237
Listing 23. Hematology ... 238
Listing 24. Blood Chemistry ... 239
Listing 25. Coagulation ... 240
Listing 26. Urinalysis (dipstick analysis) ... 241
Listing 27. Microscopic analysis ... 242
Listing 28. Thyroid Function Test ... 243
Listing 29. Cardiac Muscle Enzyme ... 244
Listing 30. Endocrine Panel ... 245
Listing 31. ECG ... 246
Listing 32. Left Ventricular Ejection Fraction ... 247
Listing 33. Ophthalmological examination: Visual Acuity and Intraocular pressure 248
Listing 34. Ophthalmological examination: Indirect Dilated Fundoscopy (ophthalmoscopy) ... 249
Listing 35. Ophthalmological examination: Slit Lamp Examination ... 250
Listing 36. Ophthalmological examination: Visual Field testing and optical coherence tomography ... 251
Listing 37. Dermatological examination ... 252
Listing 38. Work Productivity and Activity Impairment Questionnaire (WPAI:GH) ... 253
Listing 39. General Health status EQ-5D-5L ... 254
Listing 40. Health-related quality of life EORTC QLQ-C30: items ... 255
Listing 41. Health-related quality of life EORTC QLQ-C30: scale scores ... 256
Listing 42. Admnistration with Investigational Products ... 257
Listing 43. LGX818 ... 258
Listing 44. MEK162 ... 259
Listing 45. Nivolumab ... 260
Listing 46. Ipilimumab ... 261

Listing 47. Duration of exposure to IMPs and compliance 262
Listing 48. Tumor assessment: target lesions 263
Listing 49. Tumor assessment: non-target lesions 264
Listing 50. Tumor assessment: tumor response according to RECIST criteria v.1.1. 265
Listing 51. Overall survival 266
Listing 52. Total progression free survival, 3-years PFS and BORR 267
Listing 53. Duration of Response (DoR) 268
Listing 54. Adverse events 269
Listing 55. Adverse events related to Ipilimumab 270
Listing 56. Adverse events related to Nivolumab 271
Listing 57. Adverse events related to Combo Immuno Therapy 272
Listing 58. Adverse events related to LGX818 273
Listing 59. Adverse events related to MEK162 274
Listing 60. Adverse events related to Combo Target Therapy 275
Listing 61. Serious Adverse events 276
Listing 62. Adverse events leading to death 277
Listing 63. Adverse events leading to hospitalization 278
Listing 64. Adverse events leading to study withdrawal 279
Listing 65. Adverse events of special interest (AESI) 280
Listing 66. Prior Medications 281
Listing 67. Concomitant Medications 282

TABLE 1. Patients enrolment by sites

POPULATION: ENR

	Statistic	Arm A (N = XX)	Arm B (N = XX)	Arm C (N = XX)	Not randomized (N = XX)
Number of Enrolled Patients					
Site 01	n (%)	xx (xx.x%)	xx (xx.x%)	xx (xx.x%)	xx (xx.x%)
Site 02	n (%)	xx (xx.x%)	xx (xx.x%)	xx (xx.x%)	xx (xx.x%)
..					
Site nn	n (%)	xx (xx.x%)	xx (xx.x%)	xx (xx.x%)	xx (xx.x%)
Total	n (%)	xx (xx.x%)	xx (xx.x%)	xx (xx.x%)	xx (xx.x%)

Note: a) Percentages are calculated relative to the total number of patient by arm. enrolled.
b) FPFV: DDMONYYYY Patient XX.
c) LPLV: DDMONYYYY Patient YY.

Source: XXXX.SAS, Run on DDMMMYYYY

TABLE 2. Summary of screening failures

POPULATION: ENR

	Statistic	N = XX
Number of total screening failures	n (%)	xx (xx.x%)
Number of patients who did not meet at least one inclusion criterion	n (%)	xx (xx.x%)
Number of patients who did not meet at least one exclusion criterion	n (%)	xx (xx.x%)
Number of patients who did not meet at least one inclusion and/or exclusion criterion	n (%)	xx (xx.x%)

Note: Percentages are calculated relative to the total number of patients in the ENR population.

Source: XXXX.SAS, Run on DDMMMYYYY

TABLE 3. Summary of Major Protocol Deviations

POPULATION: ENR

	Statistic	Arm A (N = XX)	Arm B (N = XX)	Arm C (N = XX)
Number of patients with at least one major deviation	n (%)	xx (xx.x%)	xx (xx.x%)	xx (xx.x%)
Type of Major Protocol Deviations				
XXXXXXXXXXXXXXX	n (%)	xx (xx.x%)	xx (xx.x%)	xx (xx.x%)
XXXXXXXXXXXXXXX	n (%)	xx (xx.x%)	xx (xx.x%)	xx (xx.x%)
XXXXXXXXXXXXXXX	n (%)	xx (xx.x%)	xx (xx.x%)	xx (xx.x%)
XXXXXXXXXXXXXXX	n (%)	xx (xx.x%)	xx (xx.x%)	xx (xx.x%)

Note: a) Percentages are calculated relative to the total number of patients in the ENR Population by treatment arm.
b) Patients may be counted in more than one major protocol deviation category

Source: XXXX.SAS, Run on DDMMMYYYY

TABLE 4. Summary of patients' disposition

POPULATION: ENR

	Statistic	Arm A (N = XX)	Arm B (N = XX)	Arm C (N = XX)	Not Randomized (N = XX)
Has the subject completed the trial?					
No	n (%)	xx (xx.x%)	xx (xx.x%)	xx (xx.x%)	xx (xx.x%)
Yes	n (%)	xx (xx.x%)	xx (xx.x%)	xx (xx.x%)	xx (xx.x%)
Primary reason for withdrawal					
Progression of disease	n (%)	xx (xx.x%)	xx (xx.x%)	xx (xx.x%)	xx (xx.x%)
Death	n (%)	xx (xx.x%)	xx (xx.x%)	xx (xx.x%)	xx (xx.x%)
Withdrawn informed consent for the study and for FU	n (%)	xx (xx.x%)	xx (xx.x%)	xx (xx.x%)	xx (xx.x%)
Withdrawn informed consent for the study but NOT for FU	n (%)	xx (xx.x%)	xx (xx.x%)	xx (xx.x%)	xx (xx.x%)
Subject no longer meets study criteria (after the first PD)	n (%)	xx (xx.x%)	xx (xx.x%)	xx (xx.x%)	xx (xx.x%)
Screening failure	n (%)	xx (xx.x%)	xx (xx.x%)	xx (xx.x%)	xx (xx.x%)
Adverse event	n (%)	xx (xx.x%)	xx (xx.x%)	xx (xx.x%)	xx (xx.x%)
Poor/Non-compliance with protocol requirements	n (%)	xx (xx.x%)	xx (xx.x%)	xx (xx.x%)	xx (xx.x%)
Lost to follow-up	n (%)	xx (xx.x%)	xx (xx.x%)	xx (xx.x%)	xx (xx.x%)
Administrative reason by Sponsor	n (%)	xx (xx.x%)	xx (xx.x%)	xx (xx.x%)	xx (xx.x%)
Subject's decision	n (%)	xx (xx.x%)	xx (xx.x%)	xx (xx.x%)	xx (xx.x%)
Other					

Note:
Percentages are calculated relative to the total number of patients in the ENR population by arm.

Source: XXXX.SAS, Run on DDMMMYYYY

TABLE 5. Analysis sets

POPULATION: ENR

	Statistic	Arm A (N = XX)	Arm B (N = XX)	Arm C (N = XX)	Not Randomized (N = XX)
Number of Patients in the Enrolled Analysis Set (ENR)	n (%)	xx	xx	xx	xx
Number of Patients in the Intent-to-Treat Set (ITT)	n (%)	xx (xx.x%)	xx (xx.x%)	xx (xx.x%)	xx (xx.x%)
Number of Patients Excluded from the ITT	n (%)	xx (xx.x%)	xx (xx.x%)	xx (xx.x%)	xx (xx.x%)
Reason 01	n (%)	xx (xx.x%)	xx (xx.x%)	xx (xx.x%)	xx (xx.x%)
Reason 02	n (%)	xx (xx.x%)	xx (xx.x%)	xx (xx.x%)	xx (xx.x%)
Number of Patients in the Safety Analysis Set (SAF)	n (%)	xx (xx.x%)	xx (xx.x%)	xx (xx.x%)	xx (xx.x%)
Number of Patients Excluded from the SAF	n (%)	xx (xx.x%)	xx (xx.x%)	xx (xx.x%)	xx (xx.x%)
Reason 01	n (%)	xx (xx.x%)	xx (xx.x%)	xx (xx.x%)	xx (xx.x%)
Reason 02	n (%)	xx (xx.x%)	xx (xx.x%)	xx (xx.x%)	xx (xx.x%)
Number of Patients in the Per Protocol Analysis Set (PP)	n (%)	xx (xx.x%)	xx (xx.x%)	xx (xx.x%)	xx (xx.x%)
Number of Patients Excluded from the PP	n (%)	xx (xx.x%)	xx (xx.x%)	xx (xx.x%)	xx (xx.x%)
Reason 01	n (%)	xx (xx.x%)	xx (xx.x%)	xx (xx.x%)	xx (xx.x%)
Reason 02	n (%)	xx (xx.x%)	xx (xx.x%)	xx (xx.x%)	xx (xx.x%)

Notes:
Percentages are calculated relative to the total number of patients in the ENR population by arm.
Patients may be counted in more than one reason for exclusion.

The all patients enrolled (ENR) set contains all patients who provided informed consent for this study.

- The Intent-to-Treat (ITT) set contains all randomized patients.
- The Safety Analysis set (SAF) contains all enrolled patients who received at least one dose of study medication.
- The Per Protocol Analysis set (PP) contains all randomized patients who did not report any major protocol deviations

Source: XXXX.SAS, Run on DDMMMYYYY

TABLE 6. Demographics

POPULATION: ITT

	Statistic	Arm A (N = XX)	Arm B (N = XX)	Arm C (N = XX)
Age (years)	n	xx	xx	xx
	Mean (SD)	xx.x (xx.x)	xx.x (xx.x)	xx.x (xx.x)
	Median	xx.x	xx.x	xx.x
	Min/Max	xx / xx	xx / xx	xx / xx
Gender at birth				
Male	n (%)	xx (xx.x%)	xx (xx.x%)	xx (xx.x%)
Female	n (%)	xx (xx.x%)	xx (xx.x%)	xx (xx.x%)
Race				
White/Caucasian	n (%)	xx (xx.x%)	xx (xx.x%)	xx (xx.x%)
Black/African American	n (%)	xx (xx.x%)	xx (xx.x%)	xx (xx.x%)
Asian	n (%)	xx (xx.x%)	xx (xx.x%)	xx (xx.x%)
American Indian or Alaska Native	n (%)	xx (xx.x%)	xx (xx.x%)	xx (xx.x%)
Native Hawaiian or Pacific Islander	n (%)	xx (xx.x%)	xx (xx.x%)	xx (xx.x%)
Other	n (%)	xx (xx.x%)	xx (xx.x%)	xx (xx.x%)
Ethnic Origin				
Hispanic/Latino	n (%)	xx (xx.x%)	xx (xx.x%)	xx (xx.x%)
Not Hispanic/Latino	n (%)	xx (xx.x%)	xx (xx.x%)	xx (xx.x%)
Mixed Ethnicity	n (%)	xx (xx.x%)	xx (xx.x%)	xx (xx.x%)
Other	n (%)	xx (xx.x%)	xx (xx.x%)	xx (xx.x%)

Note:
Percentages are calculated relative to the total number of patients in the ITT population by treatment arm.
Age (years) was calculated as: integer [(Date of informed consent - 15/JUL/year of birth)/365.25].

Source: XXXX.SAS, Run on DDMMMYYYY

TABLE 7. Vital Signs and ECOG PS at screening

POPULATION: ITT

	Statistic	Arm A (N = XX)	Arm B (N = XX)	Arm C (N = XX)
Body Weight (Kg)	n	xx	xx	xx
	Mean (SD)	xx.x (xx.x)	xx.x (xx.x)	xx.x (xx.x)
	Median	xx.x	xx.x	xx.x
	Min/Max	xx / xx	xx / xx	xx / xx
Height (cm)	n	xx	xx	xx
	Mean (SD)	xx.x (xx.x)	xx.x (xx.x)	xx.x (xx.x)
	Median	xx.x	xx.x	xx.x
	Min/Max	xx / xx	xx / xx	xx / xx
BMI (kg/m2)	n	xx	xx	xx
	Mean (SD)	xx.x (xx.x)	xx.x (xx.x)	xx.x (xx.x)
	Median	xx.x	xx.x	xx.x
	Min/Max	xx / xx	xx / xx	xx / xx
Systolic Supine Blood Pressure (mmHg)	n	xx	xx	xx
	Mean (SD)	xx.x (xx.x)	xx.x (xx.x)	xx.x (xx.x)
	Median	xx.x	xx.x	xx.x
	Min/Max	xx / xx	xx / xx	xx / xx
Diastolic Supine Blood Pressure (mmHg)	n	xx	xx	xx
	Mean (SD)	xx.x (xx.x)	xx.x (xx.x)	xx.x (xx.x)
	Median	xx.x	xx.x	xx.x
	Min/Max	xx / xx	xx / xx	xx / xx
Heart rate (bpm)	n	xx	xx	xx
	Mean (SD)	xx.x (xx.x)	xx.x (xx.x)	xx.x (xx.x)
	Median	xx.x	xx.x	xx.x
	Min/Max	xx / xx	xx / xx	xx / xx
Body temperature (°C)	n	xx	xx	xx
	Mean (SD)	xx.x (xx.x)	xx.x (xx.x)	xx.x (xx.x)
	Median	xx.x	xx.x	xx.x
	Min/Max	xx / xx	xx / xx	xx / xx
Respiratory Rate (breaths/min)	n	xx	xx	xx
	Mean (SD)	xx.x (xx.x)	xx.x (xx.x)	xx.x (xx.x)
	Median	xx.x	xx.x	xx.x
	Min/Max	xx / xx	xx / xx	xx / xx
ECOG PS				
0	n (%)	xx (xx.x%)	xx (xx.x%)	xx (xx.x%)
1	n (%)	xx (xx.x%)	xx (xx.x%)	xx (xx.x%)
2	n (%)	xx (xx.x%)	xx (xx.x%)	xx (xx.x%)
3	n (%)	xx (xx.x%)	xx (xx.x%)	xx (xx.x%)
4	n (%)	xx (xx.x%)	xx (xx.x%)	xx (xx.x%)

5	n (%)	xx (xx.x%)	xx (xx.x%)	xx (xx.x%)

Note:
Percentages are calculated relative to the total number of patients in the ITT population by treatment arm.

Source: XXXX.SAS, Run on DDMMMYYYY

TABLE 8. Disease History of Melanoma: Initial diagnosis

POPULATION: ITT

	Statistic	Arm A (N = XX)	Arm B (N = XX)	Arm C (N = XX)
Melanoma type				
Cutaneous	n (%)	xx (xx.x%)	xx (xx.x%)	xx (xx.x%)
Mucosal	n (%)	xx (xx.x%)	xx (xx.x%)	xx (xx.x%)
Ocular	n (%)	xx (xx.x%)	xx (xx.x%)	xx (xx.x%)
Unknown	n (%)	xx (xx.x%)	xx (xx.x%)	xx (xx.x%)
If mucosal, primary site of mucosal melanoma (*)				
Head and neck	n (%)	xx (xx.x%)	xx (xx.x%)	xx (xx.x%)
Esophagus	n (%)	xx (xx.x%)	xx (xx.x%)	xx (xx.x%)
Stomach	n (%)	xx (xx.x%)	xx (xx.x%)	xx (xx.x%)
Small intestine	n (%)	xx (xx.x%)	xx (xx.x%)	xx (xx.x%)
Colon	n (%)	xx (xx.x%)	xx (xx.x%)	xx (xx.x%)
Rectum	n (%)	xx (xx.x%)	xx (xx.x%)	xx (xx.x%)
Vagina	n (%)	xx (xx.x%)	xx (xx.x%)	xx (xx.x%)
Bladder	n (%)	xx (xx.x%)	xx (xx.x%)	xx (xx.x%)
Other	n (%)	xx (xx.x%)	xx (xx.x%)	xx (xx.x%)
If cutaneous, primary site of cutaneous melanoma (#)				
Head	n (%)	xx (xx.x%)	xx (xx.x%)	xx (xx.x%)
Neck	n (%)	xx (xx.x%)	xx (xx.x%)	xx (xx.x%)
Trunk	n (%)	xx (xx.x%)	xx (xx.x%)	xx (xx.x%)
Legs	n (%)	xx (xx.x%)	xx (xx.x%)	xx (xx.x%)
Arms	n (%)	xx (xx.x%)	xx (xx.x%)	xx (xx.x%)
Buttocks	n (%)	xx (xx.x%)	xx (xx.x%)	xx (xx.x%)
Other	n (%)	xx (xx.x%)	xx (xx.x%)	xx (xx.x%)
Melanoma Subtype				
Superficial spreading	n (%)	xx (xx.x%)	xx (xx.x%)	xx (xx.x%)
Lentigo maligna	n (%)	xx (xx.x%)	xx (xx.x%)	xx (xx.x%)
Nodular	n (%)	xx (xx.x%)	xx (xx.x%)	xx (xx.x%)
Desmoplastic	n (%)	xx (xx.x%)	xx (xx.x%)	xx (xx.x%)
Unknown	n (%)	xx (xx.x%)	xx (xx.x%)	xx (xx.x%)
Other	n (%)	xx (xx.x%)	xx (xx.x%)	xx (xx.x%)
Time from first diagnosis of melanoma to study enrolment (months)	n	xx	xx	xx
	Mean (SD)	xx.x (xx.x)	xx.x (xx.x)	xx.x (xx.x)
	Median	xx.x	xx.x	xx.x
	Min/Max	xx / xx	xx / xx	xx / xx
Stage at initial diagnosis				
0	n (%)	xx (xx.x%)	xx (xx.x%)	xx (xx.x%)
I	n (%)	xx (xx.x%)	xx (xx.x%)	xx (xx.x%)
Ia	n (%)	xx (xx.x%)	xx (xx.x%)	xx (xx.x%)
Ib	n (%)	xx (xx.x%)	xx (xx.x%)	xx (xx.x%)
...	n (%)	xx (xx.x%)	xx (xx.x%)	xx (xx.x%)
IVc	n (%)	xx (xx.x%)	xx (xx.x%)	xx (xx.x%)

Note:
Percentages are calculated relative to the total number of patients in the ITT population by treatment arm.
Time from first diagnosis of melanoma to study enrolment (months) was calculated as: [(Date of informed consent – date of first diagnosis of melanoma)/30.4].
(*) Percentages are calculated relative to the total number of patients in the ITT set with mucosal melanoma by treatment arm.
(#) Percentages are calculated relative to the total number of patients in the ITT set with cutaneous melanoma by treatment arm.

Source: XXXX.SAS, Run on DDMMMYYYY

TABLE 9. Disease History of Melanoma: Current diagnosis

POPULATION: ITT

	Statistic	Arm A (N = XX)	Arm B (N = XX)	Arm C (N = XX)
Stage at current diagnosis				
IIIa	n (%)	xx (xx.x%)	xx (xx.x%)	xx (xx.x%)
IIIb	n (%)	xx (xx.x%)	xx (xx.x%)	xx (xx.x%)
IIIc	n (%)	xx (xx.x%)	xx (xx.x%)	xx (xx.x%)
IV – M1a	n (%)	xx (xx.x%)	xx (xx.x%)	xx (xx.x%)
IV – M1b	n (%)	xx (xx.x%)	xx (xx.x%)	xx (xx.x%)
IV – M1c	n (%)	xx (xx.x%)	xx (xx.x%)	xx (xx.x%)
Recurrence / Relapse				
Yes	n (%)	xx (xx.x%)	xx (xx.x%)	xx (xx.x%)
No	n (%)	xx (xx.x%)	xx (xx.x%)	xx (xx.x%)
Time from first recurrence/relapse (months)	n	xx	xx	xx
	Mean (SD)	xx.x (xx.x)	xx.x (xx.x)	xx.x (xx.x)
	Median	xx.x	xx.x	xx.x
	Min/Max	xx / xx	xx / xx	xx / xx
Time from most recent recurrence/relapse (months)	n	xx	xx	xx
	Mean (SD)	xx.x (xx.x)	xx.x (xx.x)	xx.x (xx.x)
	Median	xx.x	xx.x	xx.x
	Min/Max	xx / xx	xx / xx	xx / xx

Note:
Percentages are calculated relative to the total number of patients in the ITT population by treatment arm.
Time from first recurrence/relapse (months) was calculated as: [(Date of informed consent – date of first recurrence/relapse)/30.4].
Time from most recent recurrence/relapse (months) was calculated as: [(Date of informed consent – date of most recent recurrence/relapse)/30.4].

Source: XXXX.SAS, Run on DDMMMYYYY

TABLE 10. Molecular status

POPULATION: ITT

	Statistic	Arm A (N = XX)	Arm B (N = XX)	Arm C (N = XX)
Molecular status (local) mutant				
Yes	n (%)	xx (xx.x%)	xx (xx.x%)	xx (xx.x%)
No	n (%)	xx (xx.x%)	xx (xx.x%)	xx (xx.x%)
Type of mutation (*)				
Exon 15 V600E	n (%)	xx (xx.x%)	xx (xx.x%)	xx (xx.x%)
Exon 15 V600K	n (%)	xx (xx.x%)	xx (xx.x%)	xx (xx.x%)
Exon 15 V600D	n (%)	xx (xx.x%)	xx (xx.x%)	xx (xx.x%)
Exon 15 V600R	n (%)	xx (xx.x%)	xx (xx.x%)	xx (xx.x%)
Other	n (%)	xx (xx.x%)	xx (xx.x%)	xx (xx.x%)

Note:
Percentages are calculated relative to the total number of patients in the ITT population by treatment arm.
(*) Percentages are calculated relative to the total number of patients in the ITT population with molecular status mutant by treatment arm.

Source: XXXX.SAS, Run on DDMMMYYYY

TABLE 11. Childbearing potential status and pregnancy test at screening

POPULATION: ITT

	Statistic	Arm A (N = XX)	Arm B (N = XX)	Arm C (N = XX)
Childbearing potential				
Yes	n (%)	xx (xx.x%)	xx (xx.x%)	xx (xx.x%)
No	n (%)	xx (xx.x%)	xx (xx.x%)	xx (xx.x%)
Post-menopausal				
Yes	n (%)	xx (xx.x%)	xx (xx.x%)	xx (xx.x%)
No	n (%)	xx (xx.x%)	xx (xx.x%)	xx (xx.x%)
Accepted to use an adequate contraception for the total study duration?				
Yes	n (%)	xx (xx.x%)	xx (xx.x%)	xx (xx.x%)
No	n (%)	xx (xx.x%)	xx (xx.x%)	xx (xx.x%)
Serum pregnancy test done				
Yes	n (%)	xx (xx.x%)	xx (xx.x%)	xx (xx.x%)
No	n (%)	xx (xx.x%)	xx (xx.x%)	xx (xx.x%)
Not Applicable	n (%)	xx (xx.x%)	xx (xx.x%)	xx (xx.x%)
Result (*)				
Positive	n (%)	xx (xx.x%)	xx (xx.x%)	xx (xx.x%)
Negative	n (%)	xx (xx.x%)	xx (xx.x%)	xx (xx.x%)

Note:
Percentages are calculated relative to the total number of female patients in the ITT population by treatment arm.
(*) Percentages are calculated relative to the total number of female patients in the ITT population who performed serum pregnancy test by treatment arm.

Source: XXXX.SAS, Run on DDMMMYYYY

TABLE 12. HIV test at screening

POPULATION: ITT

	Statistic	Arm A (N = XX)	Arm B (N = XX)	Arm C (N = XX)
HIV test performed				
Yes	n (%)	xx (xx.x%)	xx (xx.x%)	xx (xx.x%)
No	n (%)	xx (xx.x%)	xx (xx.x%)	xx (xx.x%)
Not applicable	n (%)	xx (xx.x%)	xx (xx.x%)	xx (xx.x%)
Result (*)				
Positive	n (%)	xx (xx.x%)	xx (xx.x%)	xx (xx.x%)
Negative	n (%)	xx (xx.x%)	xx (xx.x%)	xx (xx.x%)

Note:
Percentages are calculated relative to the total number of patients in the ITT population by treatment arm.
(*) Percentages are calculated relative to the total number of patients in the ITT population who performed HIV test by treatment arm.

Source: XXXX.SAS, Run on DDMMMYYYY

TABLE 13. Summary of medical history – concomitant diseases

POPULATION: ITT

	Statistic	Arm A (N = XX)	Arm B (N = XX)	Arm C (N = XX)
Number of Patients with any concomitant disease	n (%)	xx (xx.x%)	xx (xx.x%)	xx (xx.x%)
<Primary SOC>	n (%)	xx (xx.x%)	xx (xx.x%)	xx (xx.x%)
<PT>	n (%)	xx (xx.x%)	xx (xx.x%)	xx (xx.x%)
<PT>	n (%)	xx (xx.x%)	xx (xx.x%)	xx (xx.x%)
<PT>	n (%)	xx (xx.x%)	xx (xx.x%)	xx (xx.x%)
<Primary SOC>	n (%)	xx (xx.x%)	xx (xx.x%)	xx (xx.x%)
<PT>	n (%)	xx (xx.x%)	xx (xx.x%)	xx (xx.x%)
<PT>	n (%)	xx (xx.x%)	xx (xx.x%)	xx (xx.x%)
<PT>	n (%)	xx (xx.x%)	xx (xx.x%)	xx (xx.x%)
<Primary SOC>	n (%)	xx (xx.x%)	xx (xx.x%)	xx (xx.x%)
<PT>	n (%)	xx (xx.x%)	xx (xx.x%)	xx (xx.x%)
<PT>	n (%)	xx (xx.x%)	xx (xx.x%)	xx (xx.x%)
<PT>	n (%)	xx (xx.x%)	xx (xx.x%)	xx (xx.x%)
<Primary SOC>	n (%)	xx (xx.x%)	xx (xx.x%)	xx (xx.x%)
<PT>	n (%)	xx (xx.x%)	xx (xx.x%)	xx (xx.x%)
<PT>	n (%)	xx (xx.x%)	xx (xx.x%)	xx (xx.x%)
<PT>	n (%)	xx (xx.x%)	xx (xx.x%)	xx (xx.x%)
<Primary SOC>	n (%)	xx (xx.x%)	xx (xx.x%)	xx (xx.x%)
<PT>	n (%)	xx (xx.x%)	xx (xx.x%)	xx (xx.x%)
<PT>	n (%)	xx (xx.x%)	xx (xx.x%)	xx (xx.x%)
<PT>	n (%)	xx (xx.x%)	xx (xx.x%)	xx (xx.x%)

Notes:
Percentages are calculated relative to the total number of patients in the ITT population by treatment arm.
Surgical/Medical History terms were coded using MedDRA thesaurus version XX.X.
This table includes medical terms reported as “Ongoing” in the Medical History CRF Form.

Source: XXXX.SAS, Run on DDMMMYYYY

TABLE 14. Summary of medical history – previous diseases

POPULATION: ITT

	Statistic	Arm A (N = XX)	Arm B (N = XX)	Arm C (N = XX)
Number of Patients with any previous disease	n (%)	xx (xx.x%)	xx (xx.x%)	xx (xx.x%)
<Primary SOC>	n (%)	xx (xx.x%)	xx (xx.x%)	xx (xx.x%)
<PT>	n (%)	xx (xx.x%)	xx (xx.x%)	xx (xx.x%)
<PT>	n (%)	xx (xx.x%)	xx (xx.x%)	xx (xx.x%)
<PT>	n (%)	xx (xx.x%)	xx (xx.x%)	xx (xx.x%)
<Primary SOC>	n (%)	xx (xx.x%)	xx (xx.x%)	xx (xx.x%)
<PT>	n (%)	xx (xx.x%)	xx (xx.x%)	xx (xx.x%)
<PT>	n (%)	xx (xx.x%)	xx (xx.x%)	xx (xx.x%)
<PT>	n (%)	xx (xx.x%)	xx (xx.x%)	xx (xx.x%)
<Primary SOC>	n (%)	xx (xx.x%)	xx (xx.x%)	xx (xx.x%)
<PT>	n (%)	xx (xx.x%)	xx (xx.x%)	xx (xx.x%)
<PT>	n (%)	xx (xx.x%)	xx (xx.x%)	xx (xx.x%)
<PT>	n (%)	xx (xx.x%)	xx (xx.x%)	xx (xx.x%)
<Primary SOC>	n (%)	xx (xx.x%)	xx (xx.x%)	xx (xx.x%)
<PT>	n (%)	xx (xx.x%)	xx (xx.x%)	xx (xx.x%)
<PT>	n (%)	xx (xx.x%)	xx (xx.x%)	xx (xx.x%)
<PT>	n (%)	xx (xx.x%)	xx (xx.x%)	xx (xx.x%)
<Primary SOC>	n (%)	xx (xx.x%)	xx (xx.x%)	xx (xx.x%)
<PT>	n (%)	xx (xx.x%)	xx (xx.x%)	xx (xx.x%)
<PT>	n (%)	xx (xx.x%)	xx (xx.x%)	xx (xx.x%)
<PT>	n (%)	xx (xx.x%)	xx (xx.x%)	xx (xx.x%)

Notes:
Percentages are calculated relative to the total number of patients in the ITT population by treatment arm.
Surgical/Medical History terms were coded using MedDRA thesaurus version XX.X.
This table includes medical terms reported as not “Ongoing” in the Medical History CRF Form.

Source: XXXX.SAS, Run on DDMMMYYYY

TABLE 15. Summary of clinically significant physical examination abnormalities

POPULATION: ITT

	Statistic	Arm A (N = XX)	Arm B (N = XX)	Arm C (N = XX)
Number of Patients with any clinically significant abnormality	n (%)	xx (xx.x%)	xx (xx.x%)	xx (xx.x%)
<Primary SOC>	n (%)	xx (xx.x%)	xx (xx.x%)	xx (xx.x%)
<PT>	n (%)	xx (xx.x%)	xx (xx.x%)	xx (xx.x%)
<PT>	n (%)	xx (xx.x%)	xx (xx.x%)	xx (xx.x%)
<PT>	n (%)	xx (xx.x%)	xx (xx.x%)	xx (xx.x%)
<Primary SOC>	n (%)	xx (xx.x%)	xx (xx.x%)	xx (xx.x%)
<PT>	n (%)	xx (xx.x%)	xx (xx.x%)	xx (xx.x%)
<PT>	n (%)	xx (xx.x%)	xx (xx.x%)	xx (xx.x%)
<PT>	n (%)	xx (xx.x%)	xx (xx.x%)	xx (xx.x%)
<Primary SOC>	n (%)	xx (xx.x%)	xx (xx.x%)	xx (xx.x%)
<PT>	n (%)	xx (xx.x%)	xx (xx.x%)	xx (xx.x%)
<PT>	n (%)	xx (xx.x%)	xx (xx.x%)	xx (xx.x%)
<PT>	n (%)	xx (xx.x%)	xx (xx.x%)	xx (xx.x%)
<Primary SOC>	n (%)	xx (xx.x%)	xx (xx.x%)	xx (xx.x%)
<PT>	n (%)	xx (xx.x%)	xx (xx.x%)	xx (xx.x%)
<PT>	n (%)	xx (xx.x%)	xx (xx.x%)	xx (xx.x%)
<PT>	n (%)	xx (xx.x%)	xx (xx.x%)	xx (xx.x%)
<Primary SOC>	n (%)	xx (xx.x%)	xx (xx.x%)	xx (xx.x%)
<PT>	n (%)	xx (xx.x%)	xx (xx.x%)	xx (xx.x%)
<PT>	n (%)	xx (xx.x%)	xx (xx.x%)	xx (xx.x%)
<PT>	n (%)	xx (xx.x%)	xx (xx.x%)	xx (xx.x%)

Notes:
Percentages are calculated relative to the total number of patients in the ITT population by treatment arm.
Physical examination abnormalities were coded using MedDRA thesaurus version XX.X.
This table includes medical terms reported as “Abnormal” and “Clinically significant” in the Physical Examination CRF Form.

Source: XXXX.SAS, Run on DDMMMYYYY

TABLE 16. Summary of not clinically significant physical examination abnormalities

POPULATION: ITT

	Statistic	Arm A (N = XX)	Arm B (N = XX)	Arm C (N = XX)
Number of Patients with any not clinically significant abnormality	n (%)	xx (xx.x%)	xx (xx.x%)	xx (xx.x%)
<Primary SOC>	n (%)	xx (xx.x%)	xx (xx.x%)	xx (xx.x%)
<PT>	n (%)	xx (xx.x%)	xx (xx.x%)	xx (xx.x%)
<PT>	n (%)	xx (xx.x%)	xx (xx.x%)	xx (xx.x%)
<PT>	n (%)	xx (xx.x%)	xx (xx.x%)	xx (xx.x%)
<Primary SOC>	n (%)	xx (xx.x%)	xx (xx.x%)	xx (xx.x%)
<PT>	n (%)	xx (xx.x%)	xx (xx.x%)	xx (xx.x%)
<PT>	n (%)	xx (xx.x%)	xx (xx.x%)	xx (xx.x%)
<PT>	n (%)	xx (xx.x%)	xx (xx.x%)	xx (xx.x%)
<Primary SOC>	n (%)	xx (xx.x%)	xx (xx.x%)	xx (xx.x%)
<PT>	n (%)	xx (xx.x%)	xx (xx.x%)	xx (xx.x%)
<PT>	n (%)	xx (xx.x%)	xx (xx.x%)	xx (xx.x%)
<PT>	n (%)	xx (xx.x%)	xx (xx.x%)	xx (xx.x%)
<Primary SOC>	n (%)	xx (xx.x%)	xx (xx.x%)	xx (xx.x%)
<PT>	n (%)	xx (xx.x%)	xx (xx.x%)	xx (xx.x%)
<PT>	n (%)	xx (xx.x%)	xx (xx.x%)	xx (xx.x%)
<PT>	n (%)	xx (xx.x%)	xx (xx.x%)	xx (xx.x%)
<Primary SOC>	n (%)	xx (xx.x%)	xx (xx.x%)	xx (xx.x%)
<PT>	n (%)	xx (xx.x%)	xx (xx.x%)	xx (xx.x%)
<PT>	n (%)	xx (xx.x%)	xx (xx.x%)	xx (xx.x%)
<PT>	n (%)	xx (xx.x%)	xx (xx.x%)	xx (xx.x%)

Notes:
Percentages are calculated relative to the total number of patients in the ITT population by treatment arm.
Physical examination abnormalities were coded using MedDRA thesaurus version XX.X.
This table includes medical terms reported as “Abnormal” and “Not Clinically significant” in the Physical Examination CRF Form.

Source: XXXX.SAS, Run on DDMMMYYYY

TABLE 17. Summary of prior systemic treatments

POPULATION: ITT

	Statistic	Arm A (N = XX)	Arm B (N = XX)	Arm C (N = XX)
Number of patients who received any prior systemic treatment	n (%)	xx (xx.x%)	xx (xx.x%)	xx (xx.x%)
< Level subgroup AAA>	n (%)	xx (xx.x%)	xx (xx.x%)	xx (xx.x%)
< Generic Name #1 >	n (%)	xx (xx.x%)	xx (xx.x%)	xx (xx.x%)
< Generic Name #2 >	n (%)	xx (xx.x%)	xx (xx.x%)	xx (xx.x%)
< Generic Name #3 >	n (%)	xx (xx.x%)	xx (xx.x%)	xx (xx.x%)
< Level subgroup BBB>	n (%)	xx (xx.x%)	xx (xx.x%)	xx (xx.x%)
< Generic Name #1 >	n (%)	xx (xx.x%)	xx (xx.x%)	xx (xx.x%)
< Generic Name #2 >	n (%)	xx (xx.x%)	xx (xx.x%)	xx (xx.x%)
< Generic Name #3 >				
< Level subgroup ZZZ>	n (%)	xx (xx.x%)	xx (xx.x%)	xx (xx.x%)
< Generic Name #1 >	n (%)	xx (xx.x%)	xx (xx.x%)	xx (xx.x%)
< Generic Name #2 >	n (%)	xx (xx.x%)	xx (xx.x%)	xx (xx.x%)
< Generic Name #3 >	n (%)	xx (xx.x%)	xx (xx.x%)	xx (xx.x%)

Notes:
Percentages are calculated relative to the total number of patients in the ITT set by treatment arm.
Prior systemic treatments were those treatments captured on the eCRF form “Prior Systemic Treatment”, reported as not “Ongoing” and which stopped prior to the first dose of study medication.
Prior systemic treatments were coded using the WHO-DRL Dictionary version XXXX .
Treatments were classified according to 3rd level ATC codes and Generic name.

Source: XXXX.SAS, Run on DDMMMYYYY

TABLE 18. Summary of concomitant systemic treatments

POPULATION: ITT

	Statistic	Arm A (N = XX)	Arm B (N = XX)	Arm C (N = XX)
Number of patients who received any concomitant systemic treatment	n (%)	xx (xx.x%)	xx (xx.x%)	xx (xx.x%)
< Level subgroup AAA>	n (%)	xx (xx.x%)	xx (xx.x%)	xx (xx.x%)
< Generic Name #1 >	n (%)	xx (xx.x%)	xx (xx.x%)	xx (xx.x%)
< Generic Name #2 >	n (%)	xx (xx.x%)	xx (xx.x%)	xx (xx.x%)
< Generic Name #3 >	n (%)	xx (xx.x%)	xx (xx.x%)	xx (xx.x%)
< Level subgroup BBB>	n (%)	xx (xx.x%)	xx (xx.x%)	xx (xx.x%)
< Generic Name #1 >	n (%)	xx (xx.x%)	xx (xx.x%)	xx (xx.x%)
< Generic Name #2 >	n (%)	xx (xx.x%)	xx (xx.x%)	xx (xx.x%)
< Generic Name #3 >				
< Level subgroup ZZZ>	n (%)	xx (xx.x%)	xx (xx.x%)	xx (xx.x%)
< Generic Name #1 >	n (%)	xx (xx.x%)	xx (xx.x%)	xx (xx.x%)
< Generic Name #2 >	n (%)	xx (xx.x%)	xx (xx.x%)	xx (xx.x%)
< Generic Name #3 >	n (%)	xx (xx.x%)	xx (xx.x%)	xx (xx.x%)

Notes:
Percentages are calculated relative to the total number of patients in the ITT set by treatment arm.
Concomitant systemic treatments were those treatments captured on the eCRF form “Prior Systemic Treatment” and reported as “Ongoing”.
Concomitant systemic treatments were coded using the WHO-DRL Dictionary version XXXX .
Treatments were classified according to 3rd level ATC codes and Generic name.

Source: XXXX.SAS, Run on DDMMMYYYY

TABLE 19. Summary of prior radiation treatments

POPULATION: ITT

	Statistic	Arm A (N = XX)	Arm B (N = XX)	Arm C (N = XX)
Number of patients who received any prior radiation treatment	n (%)	xx (xx.x%)	xx (xx.x%)	xx (xx.x%)
< Level subgroup AAA>	n (%)	xx (xx.x%)	xx (xx.x%)	xx (xx.x%)
< Generic Name #1 >	n (%)	xx (xx.x%)	xx (xx.x%)	xx (xx.x%)
< Generic Name #2 >	n (%)	xx (xx.x%)	xx (xx.x%)	xx (xx.x%)
< Generic Name #3 >	n (%)	xx (xx.x%)	xx (xx.x%)	xx (xx.x%)
< Level subgroup BBB>	n (%)	xx (xx.x%)	xx (xx.x%)	xx (xx.x%)
< Generic Name #1 >	n (%)	xx (xx.x%)	xx (xx.x%)	xx (xx.x%)
< Generic Name #2 >	n (%)	xx (xx.x%)	xx (xx.x%)	xx (xx.x%)
< Generic Name #3 >				
< Level subgroup ZZZ>	n (%)	xx (xx.x%)	xx (xx.x%)	xx (xx.x%)
< Generic Name #1 >	n (%)	xx (xx.x%)	xx (xx.x%)	xx (xx.x%)
< Generic Name #2 >	n (%)	xx (xx.x%)	xx (xx.x%)	xx (xx.x%)
< Generic Name #3 >	n (%)	xx (xx.x%)	xx (xx.x%)	xx (xx.x%)

Notes:
Percentages are calculated relative to the total number of patients in the ITT set by treatment arm.
Prior radiation treatments were those treatments captured on the eCRF form “Prior radiation treatment”.
Prior radiation treatments were coded using the WHO-DRL Dictionary version XXXX .
Treatments were classified according to 3rd level ATC codes and Generic name.

Source: XXXX.SAS, Run on DDMMMYYYY

TABLE 20. Summary of prior melanoma-related surgeries

POPULATION: ITT

	Statistic	Arm A (N = XX)	Arm B (N = XX)	Arm C (N = XX)
Number of patients who received any prior melanoma-related surgery	n (%)	xx (xx.x%)	xx (xx.x%)	xx (xx.x%)
< Level subgroup AAA>	n (%)	xx (xx.x%)	xx (xx.x%)	xx (xx.x%)
< Generic Name #1 >	n (%)	xx (xx.x%)	xx (xx.x%)	xx (xx.x%)
< Generic Name #2 >	n (%)	xx (xx.x%)	xx (xx.x%)	xx (xx.x%)
< Generic Name #3 >	n (%)	xx (xx.x%)	xx (xx.x%)	xx (xx.x%)
< Level subgroup BBB>	n (%)	xx (xx.x%)	xx (xx.x%)	xx (xx.x%)
< Generic Name #1 >	n (%)	xx (xx.x%)	xx (xx.x%)	xx (xx.x%)
< Generic Name #2 >	n (%)	xx (xx.x%)	xx (xx.x%)	xx (xx.x%)
< Generic Name #3 >				
< Level subgroup ZZZ>	n (%)	xx (xx.x%)	xx (xx.x%)	xx (xx.x%)
< Generic Name #1 >	n (%)	xx (xx.x%)	xx (xx.x%)	xx (xx.x%)
< Generic Name #2 >	n (%)	xx (xx.x%)	xx (xx.x%)	xx (xx.x%)
< Generic Name #3 >	n (%)	xx (xx.x%)	xx (xx.x%)	xx (xx.x%)

Notes:
Percentages are calculated relative to the total number of patients in the ITT set by treatment arm.
Prior melanoma-related surgeries were those surgeries captured on the eCRF form “Prior surgery (melanoma related)”.
Prior melanoma-related surgeries were coded using the WHO-DRL Dictionary version XXXX .
Surgeries were classified according to 3rd level ATC codes and Generic name.

Source: XXXX.SAS, Run on DDMMMYYYY

TABLE 21. Summary of prior medications

POPULATION: ITT

	Statistic	Arm A (N = XX)	Arm B (N = XX)	Arm C (N = XX)
Number of patients who received any prior medication	n (%)	xx (xx.x%)	xx (xx.x%)	xx (xx.x%)
< Level subgroup AAA>	n (%)	xx (xx.x%)	xx (xx.x%)	xx (xx.x%)
< Generic Name #1 >	n (%)	xx (xx.x%)	xx (xx.x%)	xx (xx.x%)
< Generic Name #2 >	n (%)	xx (xx.x%)	xx (xx.x%)	xx (xx.x%)
< Generic Name #3 >	n (%)	xx (xx.x%)	xx (xx.x%)	xx (xx.x%)
< Level subgroup BBB>	n (%)	xx (xx.x%)	xx (xx.x%)	xx (xx.x%)
< Generic Name #1 >	n (%)	xx (xx.x%)	xx (xx.x%)	xx (xx.x%)
< Generic Name #2 >	n (%)	xx (xx.x%)	xx (xx.x%)	xx (xx.x%)
< Generic Name #3 >				
< Level subgroup ZZZ>	n (%)	xx (xx.x%)	xx (xx.x%)	xx (xx.x%)
< Generic Name #1 >	n (%)	xx (xx.x%)	xx (xx.x%)	xx (xx.x%)
< Generic Name #2 >	n (%)	xx (xx.x%)	xx (xx.x%)	xx (xx.x%)
< Generic Name #3 >	n (%)	xx (xx.x%)	xx (xx.x%)	xx (xx.x%)

Notes:
Percentages are calculated relative to the total number of patients in the ITT set by treatment arm.
Prior medications were those medications captured on the eCRF form “Concomitant Medications” and which stopped prior to the first dose of study medication.
Prior medications were coded using the WHO-DRL Dictionary version XXXX .
Medications were classified according to 3rd level ATC codes and Generic name.

Source: XXXX.SAS, Run on DDMMMYYYY

TABLE 22. Summary of concomitant medications

POPULATION: ITT

	Statistic	Arm A (N = XX)	Arm B (N = XX)	Arm C (N = XX)
Number of patients who received any concomitant medication	n (%)	xx (xx.x%)	xx (xx.x%)	xx (xx.x%)
< Level subgroup AAA>	n (%)	xx (xx.x%)	xx (xx.x%)	xx (xx.x%)
< Generic Name #1 >	n (%)	xx (xx.x%)	xx (xx.x%)	xx (xx.x%)
< Generic Name #2 >	n (%)	xx (xx.x%)	xx (xx.x%)	xx (xx.x%)
< Generic Name #3 >	n (%)	xx (xx.x%)	xx (xx.x%)	xx (xx.x%)
< Level subgroup BBB>	n (%)	xx (xx.x%)	xx (xx.x%)	xx (xx.x%)
< Generic Name #1 >	n (%)	xx (xx.x%)	xx (xx.x%)	xx (xx.x%)
< Generic Name #2 >	n (%)	xx (xx.x%)	xx (xx.x%)	xx (xx.x%)
< Generic Name #3 >				
< Level subgroup ZZZ>	n (%)	xx (xx.x%)	xx (xx.x%)	xx (xx.x%)
< Generic Name #1 >	n (%)	xx (xx.x%)	xx (xx.x%)	xx (xx.x%)
< Generic Name #2 >	n (%)	xx (xx.x%)	xx (xx.x%)	xx (xx.x%)
< Generic Name #3 >	n (%)	xx (xx.x%)	xx (xx.x%)	xx (xx.x%)

Notes:
Percentages are calculated relative to the total number of patients in the ITT set by treatment arm.
Concomitant medications were those medications captured on the eCRF form "Concomitant Medications" and which:

- Started prior to, on or after the first dose of study medication or were ongoing at the date of last study dose AND
- Ended on or after the date of first dose of study medication or were ongoing at the date of last study dose.

Concomitant medications were coded using the WHO-DRL Dictionary version XXXX .
Medications were classified according to 3rd level ATC codes and Generic name.

Source: XXXX.SAS, Run on DDMMMYYYY

TABLE 23. Study medication exposure

POPULATION: ITT

	Statistic	Arm A (N = XX)	Arm B (N = XX)	Arm C (N = XX)
Overall duration of exposure (weeks)	n	xx	xx	xx
	Mean (SD)	xx.x (xx.x)	xx.x (xx.x)	xx.x (xx.x)
	Median	xx.x	xx.x	xx.x
	Min/Max	xx / xx	xx / xx	xx / xx
Duration of exposure to LGX818 (weeks)	n	xx	xx	xx
	Mean (SD)	xx.x (xx.x)	xx.x (xx.x)	xx.x (xx.x)
	Median	xx.x	xx.x	xx.x
	Min/Max	xx / xx	xx / xx	xx / xx
Duration of exposure to MEK162 (weeks)	n	xx	xx	xx
	Mean (SD)	xx.x (xx.x)	xx.x (xx.x)	xx.x (xx.x)
	Median	xx.x	xx.x	xx.x
	Min/Max	xx / xx	xx / xx	xx / xx
Duration of exposure to Nivolumab (weeks)	n	xx	xx	xx
	Mean (SD)	xx.x (xx.x)	xx.x (xx.x)	xx.x (xx.x)
	Median	xx.x	xx.x	xx.x
	Min/Max	xx / xx	xx / xx	xx / xx
Duration of exposure to Ipilimumab (weeks)	n	xx	xx	xx
	Mean (SD)	xx.x (xx.x)	xx.x (xx.x)	xx.x (xx.x)
	Median	xx.x	xx.x	xx.x
	Min/Max	xx / xx	xx / xx	xx / xx

Source: XXXX.SAS, Run on DDMMMYYYY

TABLE 24. Compliance to oral IMPs (LGX818 and MEK162)

POPULATION: ITT

	Statistic	Arm A (N = XX)	Arm B (N = XX)	Arm C (N = XX)
Compliance to LGX818 (%)	n	xx	xx	xx
	Mean (SD)	xx.x (xx.x)	xx.x (xx.x)	xx.x (xx.x)
	Median	xx.x	xx.x	xx.x
	Min/Max	xx / xx	xx / xx	xx / xx
Compliance to MEK162 (%)	n	xx	xx	xx
	Mean (SD)	xx.x (xx.x)	xx.x (xx.x)	xx.x (xx.x)
	Median	xx.x	xx.x	xx.x
	Min/Max	xx / xx	xx / xx	xx / xx

Source: XXXX.SAS, Run on DDMMMYYYY

TABLE 25. Analysis of Primary Endpoint: Summary of Overall Survival (OS)

POPULATION: ITT

		Statistic	Arm A (N = XX)	Arm B (N = XX)	Arm C (N = XX)
Overall Survival (months)		25th [95% CI]	xx.x [xx.x;xx.x]	xx.x [xx.x;xx.x]	xx.x [xx.x;xx.x]
		Median [95% CI]	xx.x [xx.x;xx.x]	xx.x [xx.x;xx.x]	xx.x [xx.x;xx.x]
		95th [95% CI]	xx.x [xx.x;xx.x]	xx.x [xx.x;xx.x]	xx.x [xx.x;xx.x]
Number of patients	Events	n (%)	xx (xx.x)	xx (xx.x)	xx (xx.x)
	Censored	n (%)	xx (xx.x)	xx (xx.x)	xx (xx.x)

Notes:
Overall Survival (OS) is defined as the time from the date of randomization to the date of death.
OS (months) = [(Date of death or last contact - date of randomization +1)]/30.4.
Any patient not known to have died at the time of data analysis will be censored at the time of the last recorded date on which the patient was known to be alive.
Percentages are calculated relative to the total number of the patients in the ITT set by treatment arm.
OS (months) will be estimated using Kaplan-Meier method.

TABLE 25. Analysis of Primary Endpoint: Summary of Overall Survival (OS) (cont.)

POPULATION: ITT

Arm A

Time Interval (months)	Censoring Indicator	Survival Distribution Function Estimate	Failure	Survival Standard Error	Number Failed	Number Left	SDF Lower 95% Confidence Limit	SDF Upper 95% Confidence Limit
x.xxxx	x	x.xxxx	x	x	x	xx	x.xxxx	x.xxxx
x.xxxx	x	x.xxxx	x	x	x	xx	x.xxxx	x.xxxx
x.xxxx	x	x.xxxx	x	x	x	xx	x.xxxx	x.xxxx
x.xxxx	x	x.xxxx	x	x	x	xx	x.xxxx	x.xxxx
x.xxxx	x	x.xxxx	x	x	x	xx	x.xxxx	x.xxxx
x.xxxx	x	x.xxxx	x	x	x	xx	x.xxxx	x.xxxx
x.xxxx	x	x.xxxx	x	x	x	xx	x.xxxx	x.xxxx
x.xxxx	x	x.xxxx	x	x	x	xx	x.xxxx	x.xxxx
x.xxxx	x	x.xxxx	x	x	x	xx	x.xxxx	x.xxxx

Source: XXXX.SAS, Run on DDMMMYYYY

< Note for SAS Programmer: This must be produced for each arm separately>

TABLE 25. Analysis of Primary Endpoint: Summary of Overall Survival (OS) (cont.)

POPULATION: ITT

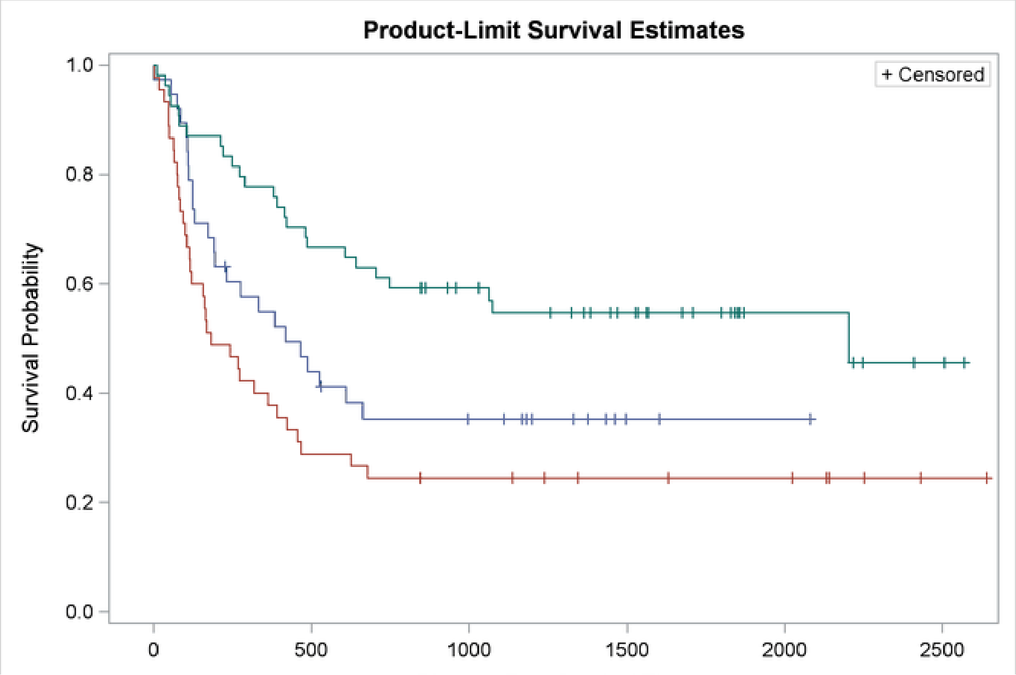

Source: XXXX.SAS, Run on DDMMMYYYY

TABLE 26. Analysis of Primary Endpoint: Predictive factors of Overall Survival (months)

POPULATION: ITT

The PHREG Procedure

Model Information

Data Set	xxxx	
Dependent Variable	xxxx	Overall Survival (Months)
Censoring Variable	xxxx	Censor
Censoring Value(s)	0	
Ties Handling	BRESLOW	

Number of Observations Read	xxx
Number of Observations Used	xxx

Summary of the Number of Event and Censored Values

Total	Event	Censored	Percent Censored
xxx	xx	xx	xx.xx

Convergence Status

Convergence criterion (GCONV=1E-8) satisfied.

Model Fit Statistics

Criterion	Without Covariates	With Covariates
-2 LOG L	xxxx.xxx	xxxx.xxx
AIC	xxxx.xxx	xxxx.xxx
SBC	xxxx.xxx	xxxx.xxx

Testing Global Null Hypothesis: BETA=0

Test	Chi-Square	DF	Pr > ChiSq
Likelihood Ratio	xx.xxxx	xx	x.xxxx
Score	xx.xxxx	xx	x.xxxx
Wald	xx.xxxx	xx	x.xxxx

Source: XXXX.SAS, Run on DDMMMYYYY

TABLE 26. Analysis of Primary Endpoint: Predictive factors of Overall Survival

POPULATION: ITT

The PHREG Procedure

Analysis of Maximum Likelihood Estimates

Parameter	DF	Parameter Estimate	Standard Error	Chi-Square	Pr > ChiSq	Hazard Ratio	95% Confidence Interval Lower	Upper
VAR1	x	x.xxxxx	x.xxxxx	x.xxxx	x.xxxx	x.xxx	x.xxx	x.xxx
VAR2	x	x.xxxxx	x.xxxxx	x.xxxx	x.xxxx	x.xxx	x.xxx	x.xxx
VAR3	x	x.xxxxx	x.xxxxx	x.xxxx	x.xxxx	x.xxx	x.xxx	x.xxx
VAR4	x	x.xxxxx	x.xxxxx	x.xxxx	x.xxxx	x.xxx	x.xxx	x.xxx
…								
VARn	x	x.xxxxx	x.xxxxx	x.xxxx	x.xxxx	x.xxx	x.xxx	x.xxx

Note for programming:

List of predictive factors to be included in the Cox’s model:

a) Treatment sequence assigned: Arm A, Arm B, Arm C;
b) Age (years): continuous variable;
c) Gender: Male and Female;
d) Melanoma Type: Cutaneous, Mucosal, Ocular;
e) Stage at current diagnosis: IIIa, IIIb, IIIc, IV – M1a, IV – M1b, IV – M1c;
f) Baseline LDH level: continuous variable;
g) ECOG PS at baseline: 0, ≥1;

Source: XXXX.SAS, Run on DDMMMYYYY

TABLE 27. Analysis of Primary Endpoint: Summary of Overall Survival (OS)

POPULATION: PP

< Note for SAS Programmer: Please repeat the Table 25 for “Per-Protocol Population”>

TABLE 28. Analysis of Primary Endpoint: Predictive factors of Overall Survival (months)

POPULATION: PP

< Note for SAS Programmer: Please repeat the Table 27 for "Per-Protocol Population">

TABLE 29. Analysis of Secondary Endpoint: Summary of Total Progression Free Survival (TPFS)

POPULATION: ITT

		Statistic	Arm A (N = XX)	Arm B (N = XX)	Arm C (N = XX)
Total Progression Free Survival (months)		25th [95% CI]	xx.x [xx.x;xx.x]	xx.x [xx.x;xx.x]	xx.x [xx.x;xx.x]
		Median [95% CI]	xx.x [xx.x;xx.x]	xx.x [xx.x;xx.x]	xx.x [xx.x;xx.x]
		95th [95% CI]	xx.x [xx.x;xx.x]	xx.x [xx.x;xx.x]	xx.x [xx.x;xx.x]
Number of patients	Events	n (%)	xx (xx.x)	xx (xx.x)	xx (xx.x)
	Censored	n (%)	xx (xx.x)	xx (xx.x)	xx (xx.x)

Notes:
Total Progression Free Survival (TPFS) is defined as the time from the date of randomization to the date of second progression (i.e. the progression to second treatment); any progression or death will be considered as an event if patient cannot complete treatment sequence.
TPFS (months) = [(Date of second progression or any progression/death or last contact – date of randomization +1)]/30.4.
Any patient not known to have experienced progression or died at the time of data analysis will be censored at the time of the last recorded date on which the patient was known to be alive without progression.
Percentages are calculated relative to the total number of the patients in the ITT set by treatment arm.
TPFS (months) will be estimated using Kaplan-Meier method.

TABLE 29. Analysis of Secondary Endpoint: Summary of Total Progression Free Survival (TPFS) (cont.)

POPULATION: ITT

Arm A

Time Interval (months)	Censoring Indicator	Survival Distribution Function Estimate	Failure	Survival Standard Error	Number Failed	Number Left	SDF Lower 95% Confidence Limit	SDF Upper 95% Confidence Limit
x.xxxx	x	x.xxxx	x	x	x	xx	x.xxxx	x.xxxx
x.xxxx	x	x.xxxx	x	x	x	xx	x.xxxx	x.xxxx
x.xxxx	x	x.xxxx	x	x	x	xx	x.xxxx	x.xxxx
x.xxxx	x	x.xxxx	x	x	x	xx	x.xxxx	x.xxxx
x.xxxx	x	x.xxxx	x	x	x	xx	x.xxxx	x.xxxx
x.xxxx	x	x.xxxx	x	x	x	xx	x.xxxx	x.xxxx
x.xxxx	x	x.xxxx	x	x	x	xx	x.xxxx	x.xxxx
x.xxxx	x	x.xxxx	x	x	x	xx	x.xxxx	x.xxxx
x.xxxx	x	x.xxxx	x	x	x	xx	x.xxxx	x.xxxx

Source: XXXX.SAS, Run on DDMMMYYYY

< Note for SAS Programmer: This must be produced for each arm separately>

TABLE 29. Analysis of Secondary Endpoint: Summary of Total Progression Free Survival (TPFS) (cont.)

POPULATION: ITT

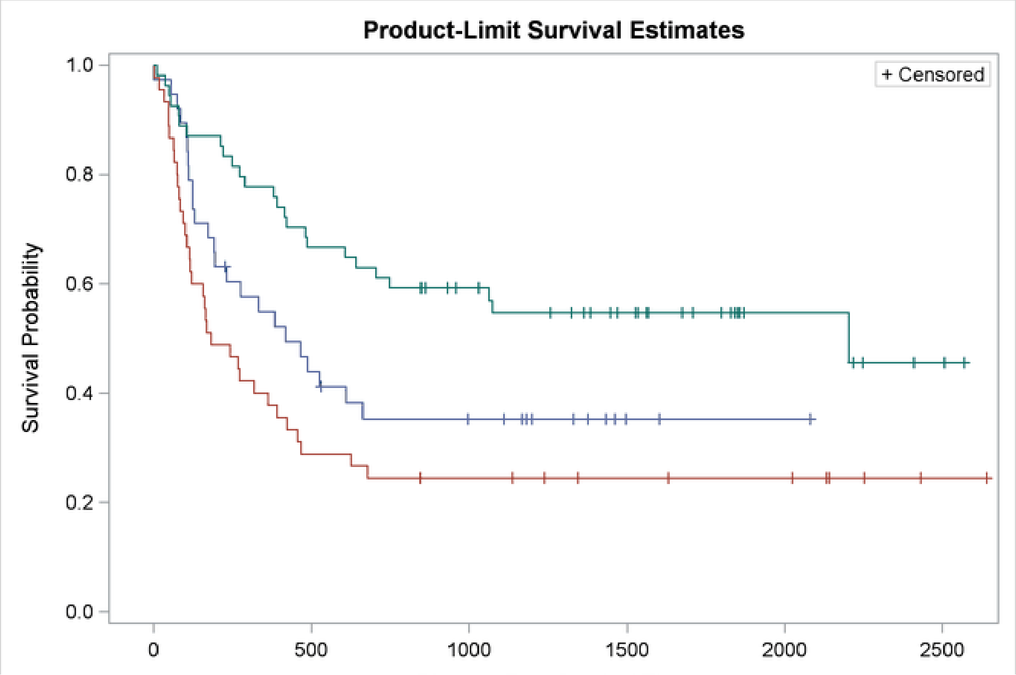

Source: XXXX.SAS, Run on DDMMMYYYY

TABLE 30. Analysis of Secondary Endpoint: Predictive factors of Total Progression Free Survival

POPULATION: ITT

The PHREG Procedure

Model Information

Data Set	xxxx	
Dependent Variable	xxxx	Total Progression Free Survival (Months)
Censoring Variable	xxxx	Censor
Censoring Value(s)	0	
Ties Handling	BRESLOW	

Number of Observations Read	xxx
Number of Observations Used	xxx

Summary of the Number of Event and Censored Values

Total	Event	Censored	Percent Censored
xxx	xx	xx	xx.xx

Convergence Status

Convergence criterion (GCONV=1E-8) satisfied.

Model Fit Statistics

Criterion	Without Covariates	With Covariates
-2 LOG L	xxxx.xxx	xxxx.xxx
AIC	xxxx.xxx	xxxx.xxx
SBC	xxxx.xxx	xxxx.xxx

Testing Global Null Hypothesis: BETA=0

Test	Chi-Square	DF	Pr > ChiSq
Likelihood Ratio	xx.xxxx	xx	x.xxxx
Score	xx.xxxx	xx	x.xxxx
Wald	xx.xxxx	xx	x.xxxx

Source: XXXX.SAS, Run on DDMMMYYYY

TABLE 30. Analysis of Secondary Endpoint: Predictive factors of Total Progression Free Survival

POPULATION: ITT

The PHREG Procedure

Analysis of Maximum Likelihood Estimates

Parameter	DF	Parameter Estimate	Standard Error	Chi-Square	Pr > ChiSq	Hazard Ratio	95% Confidence Interval Lower	Upper
VAR1	x	x.xxxxx	x.xxxxx	x.xxxx	x.xxxx	x.xxx	x.xxx	x.xxx
VAR2	x	x.xxxxx	x.xxxxx	x.xxxx	x.xxxx	x.xxx	x.xxx	x.xxx
VAR3	x	x.xxxxx	x.xxxxx	x.xxxx	x.xxxx	x.xxx	x.xxx	x.xxx
VAR4	x	x.xxxxx	x.xxxxx	x.xxxx	x.xxxx	x.xxx	x.xxx	x.xxx
…								
VARn	x	x.xxxxx	x.xxxxx	x.xxxx	x.xxxx	x.xxx	x.xxx	x.xxx

Note for programming:

List of predictive factors to be included in the Cox's model:

a) Treatment sequence assigned: Arm A, Arm B, Arm C;
b) Age (years): continuous variable;
c) Gender: Male and Female;
d) Melanoma Type: Cutaneous, Mucosal, Ocular;
e) Stage at current diagnosis: IIIa, IIIb, IIIc, IV – M1a, IV – M1b, IV – M1c;
f) Baseline LDH level: continuous variable;
g) ECOG PS at baseline: 0, ≥1;

Source: XXXX.SAS, Run on DDMMMYYYY

TABLE 31. Analysis of Secondary Endpoint: 3-years Progression Free Survival Rate (PFSR)

POPULATION: ITT

Statistic	Arm A (N = XX)	Arm B (N = XX)	Arm C (N = XX)
3-years PFSR [95% CI]	xx.x [xx.x;xx.x]	xx.x [xx.x;xx.x]	xx.x [xx.x;xx.x]

Notes:
The PFSR at 3 years was estimated by the Kaplan-Meier method.
The 95% CIs for the PFSR were estimated using Greenwood's estimate of the standard error and a linear transformation of the PFS function.

Source: XXXX.SAS, Run on DDMMMYYYY

TABLE 32. Analysis of Secondary Endpoint: Percentage of patients alive at 2 and 3 years

POPULATION: ITT

	Statistic	Arm A (N = XX)	Arm B (N = XX)	Arm C (N = XX)
Patients alive at 2 years	n (%) [95% CI]	xxx (xx.x) [xx.x;xx.x]	xxx (xx.x) [xx.x;xx.x]	xxx (xx.x) [xx.x;xx.x]
Patients alive at 3 years	n (%) [95% CI]	xxx (xx.x) [xx.x;xx.x]	xxx (xx.x) [xx.x;xx.x]	xxx (xx.x) [xx.x;xx.x]

Notes:
The 95% CIs were calculated using Wilson Score method.

Source: XXXX.SAS, Run on DDMMMYYYY

< Note for SAS Programmer: use the “binomial(Wilson)” option in FREQ procedure>

TABLE 33. Analysis of secondary endpoint: Best Overall Response (BOR) and Overall Response Rate (ORR)

POPULATION: ITT

	Statistic	Arm A (N = XX)	Arm B (N = XX)	Arm C (N = XX)
Best Overall Response (*)				
PD	n (%)	xx (xx.x%)	xx (xx.x%)	xx (xx.x%)
SD	n (%)	xx (xx.x%)	xx (xx.x%)	xx (xx.x%)
PR	n (%)	xx (xx.x%)	xx (xx.x%)	xx (xx.x%)
CR	n (%)	xx (xx.x%)	xx (xx.x%)	xx (xx.x%)
Overall Response Rate (°)	% [95% CI]	xx.x%[xx.x-xx.x]	xx.x%[xx.x-xx.x]	xx.x%[xx.x-xx.x]

Note:
Percentages are calculated relative to the total number of patients in the ITT population by treatment arm.
(*) Best overall response from the date of randomization to the date of objectively documented progression per RECIST version 1.1 criteria.
(°) Overall Response Rate is the percentage of patients with CR or PR before any evidence of progression per RECIST version 1.1 criteria. 95% CI was derived using Wilson Score interval.

Source: XXXX.SAS, Run on DDMMMYYYY

TABLE 34. Analysis of Secondary Endpoint: Summary of Duration of Response (DoR)

POPULATION: ITT

		Statistic	Arm A (N = XX)	Arm B (N = XX)	Arm C (N = XX)
Duration of Response (months)		25th [95% CI]	xx.x [xx.x;xx.x]	xx.x [xx.x;xx.x]	xx.x [xx.x;xx.x]
		Median [95% CI]	xx.x [xx.x;xx.x]	xx.x [xx.x;xx.x]	xx.x [xx.x;xx.x]
		95th [95% CI]	xx.x [xx.x;xx.x]	xx.x [xx.x;xx.x]	xx.x [xx.x;xx.x]
Number of patients	Events	n (%)	xx (xx.x)	xx (xx.x)	xx (xx.x)
	Censored	n (%)	xx (xx.x)	xx (xx.x)	xx (xx.x)

Notes:
Duration of Response (DoR) is defined as the time from the date of first documented response (CR or PR) to the date of first documented progression or death due to underlying cancer;
DoR (months) = [(Date of first documented progression/death due to underlying cancer or date of last tumor assessment - date of first CR or PR +1)]/30.4.
If a patient with a CR or PR has no progression or death due to underlying cancer, the patient is censored at the date of last adequate tumor assessment.
Percentages are calculated relative to the total number of the patients in the ITT set by treatment arm.
DoR (months) will be estimated using Kaplan-Meier method.

Source: XXXX.SAS, Run on DDMMMYYYY

TABLE 34. Analysis of Secondary Endpoint: Summary of Duration of Response (DoR) (cont.)

POPULATION: ITT

Arm A

Time Interval (months)	Censoring Indicator	Survival Distribution Function Estimate	Failure	Survival Standard Error	Number Failed	Number Left	SDF Lower 95% Confidence Limit	SDF Upper 95% Confidence Limit
x.xxxx	x	x.xxxx	x	x	x	xx	x.xxxx	x.xxxx
x.xxxx	x	x.xxxx	x	x	x	xx	x.xxxx	x.xxxx
x.xxxx	x	x.xxxx	x	x	x	xx	x.xxxx	x.xxxx
x.xxxx	x	x.xxxx	x	x	x	xx	x.xxxx	x.xxxx
x.xxxx	x	x.xxxx	x	x	x	xx	x.xxxx	x.xxxx
x.xxxx	x	x.xxxx	x	x	x	xx	x.xxxx	x.xxxx
x.xxxx	x	x.xxxx	x	x	x	xx	x.xxxx	x.xxxx
x.xxxx	x	x.xxxx	x	x	x	xx	x.xxxx	x.xxxx
x.xxxx	x	x.xxxx	x	x	x	xx	x.xxxx	x.xxxx

Source: XXXX.SAS, Run on DDMMMYYYY

< Note for SAS Programmer: This must be produced for each arm separately>

TABLE 34. Analysis of Secondary Endpoint: Summary of Duration of Response (DoR) (cont.)

POPULATION: ITT

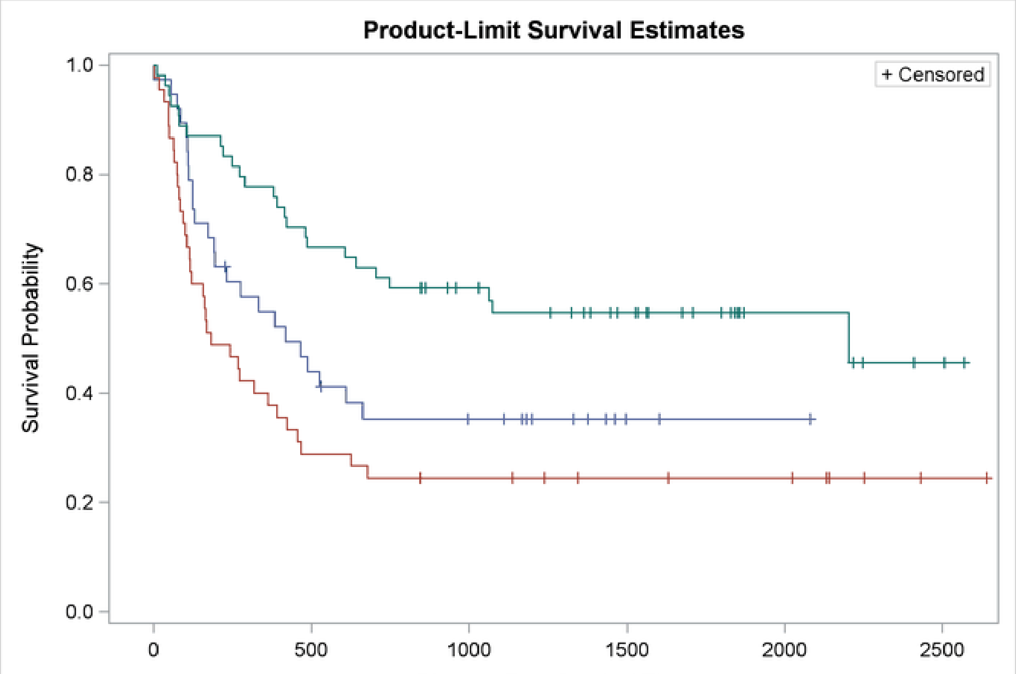

Source: XXXX.SAS, Run on DDMMMYYYY

TABLE 35. Analysis of Secondary Endpoint: Predictive factors of Duration of Response

POPULATION: ITT

The PHREG Procedure

Model Information

Data Set	xxxx	
Dependent Variable	xxxx	Duration of Response (Months)
Censoring Variable	xxxx	Censor
Censoring Value(s)	0	
Ties Handling	BRESLOW	

Number of Observations Read	xxx
Number of Observations Used	xxx

Summary of the Number of Event and Censored Values

Total	Event	Censored	Percent Censored
xxx	xx	xx	xx.xx

Convergence Status

Convergence criterion (GCONV=1E-8) satisfied.

Model Fit Statistics

Criterion	Without Covariates	With Covariates
-2 LOG L	xxxx.xxx	xxxx.xxx
AIC	xxxx.xxx	xxxx.xxx
SBC	xxxx.xxx	xxxx.xxx

Testing Global Null Hypothesis: BETA=0

Test	Chi-Square	DF	Pr > ChiSq
Likelihood Ratio	xx.xxxx	xx	x.xxxx
Score	xx.xxxx	xx	x.xxxx
Wald	xx.xxxx	xx	x.xxxx

Source: XXXX.SAS, Run on DDMMMYYYY

Table 35. Analysis of Secondary Endpoint: Predictive factors of Duration of Response

POPULATION: ITT

The PHREG Procedure

Analysis of Maximum Likelihood Estimates

Parameter	DF	Parameter Estimate	Standard Error	Chi-Square	Pr > ChiSq	Hazard Ratio	95% Confidence Interval Lower	95% Confidence Interval Upper
VAR1	x	x.xxxxx	x.xxxxx	x.xxxx	x.xxxx	x.xxx	x.xxx	x.xxx
VAR2	x	x.xxxxx	x.xxxxx	x.xxxx	x.xxxx	x.xxx	x.xxx	x.xxx
VAR3	x	x.xxxxx	x.xxxxx	x.xxxx	x.xxxx	x.xxx	x.xxx	x.xxx
VAR4	x	x.xxxxx	x.xxxxx	x.xxxx	x.xxxx	x.xxx	x.xxx	x.xxx
…								
VARn	x	x.xxxxx	x.xxxxx	x.xxxx	x.xxxx	x.xxx	x.xxx	x.xxx

Note for programming:

List of predictive factors to be included in the Cox’s model:

a) Treatment sequence assigned: Arm A, Arm B, Arm C;
b) Age (years): continuous variable;
c) Gender: Male and Female;
d) Melanoma Type: Cutaneous, Mucosal, Ocular;
e) Stage at current diagnosis: IIIa, IIIb, IIIc, IV – M1a, IV – M1b, IV – M1c;
f) Baseline LDH level: continuous variable;
g) ECOG PS at baseline: 0, ≥1;

Source: XXXX.SAS, Run on DDMMMYYYY

TABLE 36. Analysis of Secondary Endpoint: Change from baseline of Health-Related Quality of Life (HRQoL) by means of EORTC-QLQ-C30 – ARM A

POPULATION: ITT **< Note for SAS Programmer: the following must be presented for each scale of the EORTC-QLQ-C30 >**

	Statistic	Value	Change from Baseline (*)
Global Health Status / QoL			
Baseline 1	n	xx	
	Mean (SD)	xx.x (x.x)	
	Median	xx.x	
	Min/Max	xx/xx	
Week *<Every 4 wks>* – Combo Target	n	xx	xx
	Mean (SD)	xx.x (x.x)	xx.x (x.x)
	Median	xx.x	xx.x
	Min/Max	xx/xx	xx/xx
Baseline 2	n	xx	
	Mean (SD)	xx.x (x.x)	
	Median	xx.x	
	Min/Max	xx/xx	
Week *<Every 3 wks x 4 times>* – Nivolumab + Ipilimumab	n	xx	xx
	Mean (SD)	xx.x (x.x)	xx.x (x.x)
	Median	xx.x	xx.x
	Min/Max	xx/xx	xx/xx
Week *<Every 4 wks>* – Nivolumab	n	xx	xx
	Mean (SD)	xx.x (x.x)	xx.x (x.x)
	Median	xx.x	xx.x
	Min/Max	xx/xx	xx/xx
End of Treatment	n	xx	xx
	Mean (SD)	xx.x (x.x)	xx.x (x.x)
	Median	xx.x	xx.x
	Min/Max	xx/xx	xx/xx
Follow-up	n	xx	xx
	Mean (SD)	xx.x (x.x)	xx.x (x.x)
	Median	xx.x	xx.x
	Min/Max	xx/xx	xx/xx

Notes:
(*) Change from baseline is calculated relative to Baseline 1 for values between Baseline 1 and Baseline 2; Baseline 2 for values after Baseline 2. Change from baseline values include only those patients with both a Baseline value and a value for summarized time period.
n represents number of patients contributing to summary statistics.

Source: XXXX.SAS, Run on DDMMMYYYY

TABLE 37. Analysis of Secondary Endpoint: Change from baseline of Health-Related Quality of Life (HRQoL) by means of EORTC-QLQ-C30 – ARM B

POPULATION: ITT **< Note for SAS Programmer: the following must be presented for each scale of the EORTC-QLQ-C30 >**

	Statistic	Value	Change from Baseline (*)
Global Health Status / QoL			
Baseline 1	n	xx	
	Mean (SD)	xx.x (x.x)	
	Median	xx.x	
	Min/Max	xx/xx	
Week *<Every 3 wks x 4 times>* – Nivolumab + Ipilimumab	n	xx	xx
	Mean (SD)	xx.x (x.x)	xx.x (x.x)
	Median	xx.x	xx.x
	Min/Max	xx/xx	xx/xx
Week *<Every 4 wks>* – Nivolumab	n	xx	xx
	Mean (SD)	xx.x (x.x)	xx.x (x.x)
	Median	xx.x	xx.x
	Min/Max	xx/xx	xx/xx
Baseline 2	n	xx	
	Mean (SD)	xx.x (x.x)	
	Median	xx.x	
	Min/Max	xx/xx	
Week *<Every 4 wks>* – Combo Target	n	xx	xx
	Mean (SD)	xx.x (x.x)	xx.x (x.x)
	Median	xx.x	xx.x
	Min/Max	xx/xx	xx/xx
End of Treatment	n	xx	xx
	Mean (SD)	xx.x (x.x)	xx.x (x.x)
	Median	xx.x	xx.x
	Min/Max	xx/xx	xx/xx
Follow-up	n	xx	xx
	Mean (SD)	xx.x (x.x)	xx.x (x.x)
	Median	xx.x	xx.x
	Min/Max	xx/xx	xx/xx

Notes:
(*) Change from baseline is calculated relative to Baseline 1 for values between Baseline 1 and Baseline 2; Baseline 2 for values after Baseline 2. Change from baseline values include only those patients with both a Baseline value and a value for summarized time period.
n represents number of patients contributing to summary statistics.
Source: XXXX.SAS, Run on DDMMMYYYY

TABLE 38. Analysis of Secondary Endpoint: Change from baseline of Health-Related Quality of Life (HRQoL) by means of EORTC-QLQ-C30 – ARM C

POPULATION: ITT **< Note for SAS Programmer: the following must be presented for each scale of the EORTC-QLQ-C30 >**

	Statistic	Value	Change from Baseline (*)
Global Health Status / QoL			
Baseline 1	n	xx	
	Mean (SD)	xx.x (x.x)	
	Median	xx.x	
	Min/Max	xx/xx	
Day 1 – Combo Target	n	xx	xx
	Mean (SD)	xx.x (x.x)	xx.x (x.x)
	Median	xx.x	xx.x
	Min/Max	xx/xx	xx/xx
Week 4 – Combo Target	n	xx	xx
	Mean (SD)	xx.x (x.x)	xx.x (x.x)
	Median	xx.x	xx.x
	Min/Max	xx/xx	xx/xx
Baseline 2	n	xx	
	Mean (SD)	xx.x (x.x)	
	Median	xx.x	
	Min/Max	xx/xx	
Week *<Every 3 wks x 4 times>* – Nivolumab + Ipilimumab	n	xx	xx
	Mean (SD)	xx.x (x.x)	xx.x (x.x)
	Median	xx.x	xx.x
	Min/Max	xx/xx	xx/xx
Week *<Every 4 wks>* – Nivolumab	n	xx	xx
	Mean (SD)	xx.x (x.x)	xx.x (x.x)
	Median	xx.x	xx.x
	Min/Max	xx/xx	xx/xx
Baseline 3	n	xx	
	Mean (SD)	xx.x (x.x)	
	Median	xx.x	
	Min/Max	xx/xx	
Week *<Every 4 wks>* – Combo Target	n	xx	xx
	Mean (SD)	xx.x (x.x)	xx.x (x.x)
	Median	xx.x	xx.x
	Min/Max	xx/xx	xx/xx

Notes:

(*) Change from baseline is calculated relative to Baseline 1 for values between Baseline 1 and Baseline 2; Baseline 2 for values between Baseline 2 and Baseline 3; Baseline 3 for values after Baseline 3. Change from baseline values include only those patients with both a Baseline value and a value for summarized time period.
n represents number of patients contributing to summary statistics.

Source: XXXX.SAS, Run on DDMMMYYYY

TABLE 38. Analysis of Secondary Endpoint: Change from baseline of Health-Related Quality of Life (HRQoL) by means of EORTC-QLQ-C30 – ARM C (cont.)

POPULATION: ITT **< Note for SAS Programmer: the following must be presented for each scale of the EORTC-QLQ-C30 >**

	Statistic	Value	Change from Baseline (*)
Global Health Status / QoL			
End of Treatment	n	xx	xx
	Mean (SD)	xx.x (x.x)	xx.x (x.x)
	Median	xx.x	xx.x
	Min/Max	xx/xx	xx/xx
Follow-up	n	xx	xx
	Mean (SD)	xx.x (x.x)	xx.x (x.x)
	Median	xx.x	xx.x
	Min/Max	xx/xx	xx/xx

Notes:
(*) Change from baseline is calculated relative to Baseline 1 for values between Baseline 1 and Baseline 2; Baseline 2 for values between Baseline 2 and Baseline 3; Baseline 3 for values after Baseline 3. Change from baseline values include only those patients with both a Baseline value and a value for summarized time period.
n represents number of patients contributing to summary statistics.

Source: XXXX.SAS, Run on DDMMMYYYY

TABLE 39. Analysis of Secondary Endpoint: Change from baseline of EQ-5D-5L by means of EQ-5D index – ARM A

POPULATION: ITT

	Statistic	Value	Change from Baseline (*)
Baseline 1	n	xx	
	Mean (SD)	xx.x (x.x)	
	Median	xx.x	
	Min/Max	xx/xx	
Week *<Every 4 wks>* – Combo Target	n	xx	xx
	Mean (SD)	xx.x (x.x)	xx.x (x.x)
	Median	xx.x	xx.x
	Min/Max	xx/xx	xx/xx
Baseline 2	n	xx	
	Mean (SD)	xx.x (x.x)	
	Median	xx.x	
	Min/Max	xx/xx	
Week *<Every 3 wks x 4 times>* – Nivolumab + Ipilimumab	n	xx	xx
	Mean (SD)	xx.x (x.x)	xx.x (x.x)
	Median	xx.x	xx.x
	Min/Max	xx/xx	xx/xx
Week *<Every 4 wks>* – Nivolumab	n	xx	xx
	Mean (SD)	xx.x (x.x)	xx.x (x.x)
	Median	xx.x	xx.x
	Min/Max	xx/xx	xx/xx
End of Treatment	n	xx	xx
	Mean (SD)	xx.x (x.x)	xx.x (x.x)
	Median	xx.x	xx.x
	Min/Max	xx/xx	xx/xx
Follow-up	n	xx	xx
	Mean (SD)	xx.x (x.x)	xx.x (x.x)
	Median	xx.x	xx.x
	Min/Max	xx/xx	xx/xx

Notes:
(*) Change from baseline is calculated relative to Baseline 1 for values between Baseline 1 and Baseline 2; Baseline 2 for values after Baseline 2. Change from baseline values include only those patients with both a Baseline value and a value for summarized time period.
n represents number of patients contributing to summary statistics.

Source: XXXX.SAS, Run on DDMMMYYYY

TABLE 40. Analysis of Secondary Endpoint: Change from baseline of EQ-5D-5L by means of EQ-5D index – ARM B

POPULATION: ITT

	Statistic	Value	Change from Baseline (*)
Baseline 1	n	xx	
	Mean (SD)	xx.x (x.x)	
	Median	xx.x	
	Min/Max	xx/xx	
Week *<Every 3 wks x 4 times>* – Nivolumab + Ipilimumab	n	xx	xx
	Mean (SD)	xx.x (x.x)	xx.x (x.x)
	Median	xx.x	xx.x
	Min/Max	xx/xx	xx/xx
Week *<Every 4 wks>* – Nivolumab	n	xx	xx
	Mean (SD)	xx.x (x.x)	xx.x (x.x)
	Median	xx.x	xx.x
	Min/Max	xx/xx	xx/xx
Baseline 2	n	xx	
	Mean (SD)	xx.x (x.x)	
	Median	xx.x	
	Min/Max	xx/xx	
Week *<Every 4 wks>* – Combo Target	n	xx	xx
	Mean (SD)	xx.x (x.x)	xx.x (x.x)
	Median	xx.x	xx.x
	Min/Max	xx/xx	xx/xx
End of Treatment	n	xx	xx
	Mean (SD)	xx.x (x.x)	xx.x (x.x)
	Median	xx.x	xx.x
	Min/Max	xx/xx	xx/xx
Follow-up	n	xx	xx
	Mean (SD)	xx.x (x.x)	xx.x (x.x)
	Median	xx.x	xx.x
	Min/Max	xx/xx	xx/xx

Notes:
(*) Change from baseline is calculated relative to Baseline 1 for values between Baseline 1 and Baseline 2; Baseline 2 for values after Baseline 2. Change from baseline values include only those patients with both a Baseline value and a value for summarized time period.
n represents number of patients contributing to summary statistics.
Source: XXXX.SAS, Run on DDMMMYYYY

TABLE 41. Analysis of Secondary Endpoint: Change from baseline of EQ-5D-5L by means of EQ-5D index – ARM C

POPULATION: ITT

	Statistic	Value	Change from Baseline (*)
Baseline 1	n	xx	
	Mean (SD)	xx.x (x.x)	
	Median	xx.x	
	Min/Max	xx/xx	
Day 1 – Combo Target	n	xx	xx
	Mean (SD)	xx.x (x.x)	xx.x (x.x)
	Median	xx.x	xx.x
	Min/Max	xx/xx	xx/xx
Week 4 – Combo Target	n	xx	xx
	Mean (SD)	xx.x (x.x)	xx.x (x.x)
	Median	xx.x	xx.x
	Min/Max	xx/xx	xx/xx
Baseline 2	n	xx	
	Mean (SD)	xx.x (x.x)	
	Median	xx.x	
	Min/Max	xx/xx	
Week *<Every 3 wks x 4 times>* – Nivolumab + Ipilimumab	n	xx	xx
	Mean (SD)	xx.x (x.x)	xx.x (x.x)
	Median	xx.x	xx.x
	Min/Max	xx/xx	xx/xx
Week *<Every 4 wks>* – Nivolumab	n	xx	xx
	Mean (SD)	xx.x (x.x)	xx.x (x.x)
	Median	xx.x	xx.x
	Min/Max	xx/xx	xx/xx
Baseline 3	n	xx	
	Mean (SD)	xx.x (x.x)	
	Median	xx.x	
	Min/Max	xx/xx	
Week *<Every 4 wks>* – Combo Target	n	xx	xx
	Mean (SD)	xx.x (x.x)	xx.x (x.x)
	Median	xx.x	xx.x
	Min/Max	xx/xx	xx/xx

Notes:

(*) Change from baseline is calculated relative to Baseline 1 for values between Baseline 1 and Baseline 2; Baseline 2 for values between Baseline 2 and Baseline 3; Baseline 3 for values after Baseline 3. Change from baseline values include only those patients with both a Baseline value and a value for summarized time period.
n represents number of patients contributing to summary statistics.

Source: XXXX.SAS, Run on DDMMMYYYY

TABLE 41. Analysis of Secondary Endpoint: Change from baseline of Health-Related Quality of Life (HRQoL) by means of EORTC-QLQ-C30 – ARM C (cont.)

POPULATION: ITT **< Note for SAS Programmer: the following must be presented for each scale of the EORTC-QLQ-C30 >**

	Statistic	Value	Change from Baseline (*)
Global Health Status / QoL			
End of Treatment	n	xx	xx
	Mean (SD)	xx.x (x.x)	xx.x (x.x)
	Median	xx.x	xx.x
	Min/Max	xx/xx	xx/xx
Follow-up	n	xx	xx
	Mean (SD)	xx.x (x.x)	xx.x (x.x)
	Median	xx.x	xx.x
	Min/Max	xx/xx	xx/xx

Notes:
(*) Change from baseline is calculated relative to Baseline 1 for values between Baseline 1 and Baseline 2; Baseline 2 for values between Baseline 2 and Baseline 3; Baseline 3 for values after Baseline 3. Change from baseline values include only those patients with both a Baseline value and a value for summarized time period.
n represents number of patients contributing to summary statistics.

Source: XXXX.SAS, Run on DDMMMYYYY

TABLE 42. Analysis of Secondary Endpoint: Change from baseline of Impairment of work productivity and activity by means of WPAI:GH – ARM A

POPULATION: ITT **< Note for SAS Programmer: the following must be presented for each scale of the WPAI:GH >**

	Statistic	Value	Change from Baseline (*)
Percent work time missed due to health			
Baseline 1	n	xx	
	Mean (SD)	xx.x (x.x)	
	Median	xx.x	
	Min/Max	xx/xx	
Week *<Every 4 wks>* – Combo Target	n	xx	xx
	Mean (SD)	xx.x (x.x)	xx.x (x.x)
	Median	xx.x	xx.x
	Min/Max	xx/xx	xx/xx
Baseline 2	n	xx	
	Mean (SD)	xx.x (x.x)	
	Median	xx.x	
	Min/Max	xx/xx	
Week *<Every 3 wks x 4 times>* – Nivolumab + Ipilimumab	n	xx	xx
	Mean (SD)	xx.x (x.x)	xx.x (x.x)
	Median	xx.x	xx.x
	Min/Max	xx/xx	xx/xx
Week *<Every 4 wks>* – Nivolumab	n	xx	xx
	Mean (SD)	xx.x (x.x)	xx.x (x.x)
	Median	xx.x	xx.x
	Min/Max	xx/xx	xx/xx
End of Treatment	n	xx	xx
	Mean (SD)	xx.x (x.x)	xx.x (x.x)
	Median	xx.x	xx.x
	Min/Max	xx/xx	xx/xx
Follow-up	n	xx	xx
	Mean (SD)	xx.x (x.x)	xx.x (x.x)
	Median	xx.x	xx.x
	Min/Max	xx/xx	xx/xx

Notes:

(*) Change from baseline is calculated relative to Baseline 1 for values between Baseline 1 and Baseline 2; Baseline 2 for values after Baseline 2. Change from baseline values include only those patients with both a Baseline value and a value for summarized time period.
n represents number of patients contributing to summary statistics.

Source: XXXX.SAS, Run on DDMMMYYYY

TABLE 43. Analysis of Secondary Endpoint: Change from baseline of Impairment of work productivity and activity by means of WPAI:GH – ARM B

POPULATION: ITT **< Note for SAS Programmer: the following must be presented for each scale of the WPAI:GH >**

	Statistic	Value	Change from Baseline (*)
Percent work time missed due to health			
Baseline 1	n	xx	
	Mean (SD)	xx.x (x.x)	
	Median	xx.x	
	Min/Max	xx/xx	
Week *<Every 3 wks x 4 times>* – Nivolumab + Ipilimumab	n	xx	xx
	Mean (SD)	xx.x (x.x)	xx.x (x.x)
	Median	xx.x	xx.x
	Min/Max	xx/xx	xx/xx
Week *<Every 4 wks>* – Nivolumab	n	xx	xx
	Mean (SD)	xx.x (x.x)	xx.x (x.x)
	Median	xx.x	xx.x
	Min/Max	xx/xx	xx/xx
Baseline 2	n	xx	
	Mean (SD)	xx.x (x.x)	
	Median	xx.x	
	Min/Max	xx/xx	
Week *<Every 4 wks>* – Combo Target	n	xx	xx
	Mean (SD)	xx.x (x.x)	xx.x (x.x)
	Median	xx.x	xx.x
	Min/Max	xx/xx	xx/xx
End of Treatment	n	xx	xx
	Mean (SD)	xx.x (x.x)	xx.x (x.x)
	Median	xx.x	xx.x
	Min/Max	xx/xx	xx/xx
Follow-up	n	xx	xx
	Mean (SD)	xx.x (x.x)	xx.x (x.x)
	Median	xx.x	xx.x
	Min/Max	xx/xx	xx/xx

Notes:
(*) Change from baseline is calculated relative to Baseline 1 for values between Baseline 1 and Baseline 2; Baseline 2 for values after Baseline 2. Change from baseline values include only those patients with both a Baseline value and a value for summarized time period.
n represents number of patients contributing to summary statistics.
Source: XXXX.SAS, Run on DDMMMYYYY

TABLE 44. Analysis of Secondary Endpoint: Change from baseline of Impairment of work productivity and activity by means of WPAI:GH – ARM C

POPULATION: ITT **< Note for SAS Programmer: the following must be presented for each scale of the WPAI:GH >**

	Statistic	Value	Change from Baseline (*)
Percent work time missed due to health			
Baseline 1	n	xx	
	Mean (SD)	xx.x (x.x)	
	Median	xx.x	
	Min/Max	xx/xx	
Day 1 – Combo Target	n	xx	xx
	Mean (SD)	xx.x (x.x)	xx.x (x.x)
	Median	xx.x	xx.x
	Min/Max	xx/xx	xx/xx
Week 4 – Combo Target	n	xx	xx
	Mean (SD)	xx.x (x.x)	xx.x (x.x)
	Median	xx.x	xx.x
	Min/Max	xx/xx	xx/xx
Baseline 2	n	xx	
	Mean (SD)	xx.x (x.x)	
	Median	xx.x	
	Min/Max	xx/xx	
Week *<Every 3 wks x 4 times>* – Nivolumab + Ipilimumab	n	xx	xx
	Mean (SD)	xx.x (x.x)	xx.x (x.x)
	Median	xx.x	xx.x
	Min/Max	xx/xx	xx/xx
Week *<Every 4 wks>* – Nivolumab	n	xx	xx
	Mean (SD)	xx.x (x.x)	xx.x (x.x)
	Median	xx.x	xx.x
	Min/Max	xx/xx	xx/xx
Baseline 3	n	xx	
	Mean (SD)	xx.x (x.x)	
	Median	xx.x	
	Min/Max	xx/xx	
Week *<Every 4 wks>* – Combo Target	n	xx	xx
	Mean (SD)	xx.x (x.x)	xx.x (x.x)
	Median	xx.x	xx.x

Min/Max	xx/xx	xx/xx

Notes:
(*) Change from baseline is calculated relative to Baseline 1 for values between Baseline 1 and Baseline 2; Baseline 2 for values between Baseline 2 and Baseline 3; Baseline 3 for values after Baseline 3. Change from baseline values include only those patients with both a Baseline value and a value for summarized time period.
n represents number of patients contributing to summary statistics.

Source: XXXX.SAS, Run on DDMMMYYYY

Table 44. Analysis of Secondary Endpoint: Change from baseline of Impairment of work productivity and activity by means of WPAI:GH – ARM C (cont.)

POPULATION: ITT **< Note for SAS Programmer: the following must be presented for each scale of the WPAI:GH >**

	Statistic	Value	Change from Baseline (*)
Percent work time missed due to health			
End of Treatment	n	xx	xx
	Mean (SD)	xx.x (x.x)	xx.x (x.x)
	Median	xx.x	xx.x
	Min/Max	xx/xx	xx/xx
Follow-up	n	xx	xx
	Mean (SD)	xx.x (x.x)	xx.x (x.x)
	Median	xx.x	xx.x
	Min/Max	xx/xx	xx/xx

Notes:
(*) Change from baseline is calculated relative to Baseline 1 for values between Baseline 1 and Baseline 2; Baseline 2 for values between Baseline 2 and Baseline 3; Baseline 3 for values after Baseline 3. Change from baseline values include only those patients with both a Baseline value and a value for summarized time period.
n represents number of patients contributing to summary statistics.

Source: XXXX.SAS, Run on DDMMMYYYY

TABLE 45. Summary of adverse events

POPULATION: SAF

	Statistic	Arm A (N = XX)	Arm B (N = XX)	Arm C (N = XX)
Number of AEs	n	xx	xx	xx
Number of non-serious AEs	n	xx	xx	xx
Number of AEs related to Ipilimumab	n	xx	xx	xx
Number of AEs related to Nivolumab	n	xx	xx	xx
Number of AEs related to Combo Immuno Therapy	n	xx	xx	xx
Number of AEs related to LGX818	n	xx	xx	xx
Number of AEs related to MEK162	n	xx	xx	xx
Number of AEs related to Combo Target Therapy	n	xx	xx	xx
Number of serious AEs (SAE)	n	xx	xx	xx
Number of SAEs related to Ipilimumab	n	xx	xx	xx
Number of SAEs related to Nivolumab	n	xx	xx	xx
Number of SAEs related to Combo Immuno Therapy	n	xx	xx	xx
Number of SAEs related to Combo LGX818	n	xx	xx	xx
Number of SAEs related to Combo MEK162	n	xx	xx	xx
Number of SAEs related to Combo Target Therapy	n	xx	xx	xx
Number of AEs leading to hospitalization	n	xx	xx	xx
Number of AEs leading to death	n	xx	xx	xx
Number of AEs leading to withdrawal from the study	n	xx	xx	xx
Number of AEs of special interest (AESI)	n	xx	xx	xx
Number of Patients with at Least One AE	n (%)	xx (xx.x%)	xx (xx.x%)	xx (xx.x%)
Number of Patients with at Least One non-serious AE	n (%)	xx (xx.x%)	xx (xx.x%)	xx (xx.x%)
Number of Patients with at Least One AE related to Ipilimumab	n (%)	xx (xx.x%)	xx (xx.x%)	xx (xx.x%)
Number of Patients with at Least One AE related to Nivolumab	n (%)	xx (xx.x%)	xx (xx.x%)	xx (xx.x%)
Number of Patients with at Least One AE related to Combo Immuno Therapy	n (%)	xx (xx.x%)	xx (xx.x%)	xx (xx.x%)
Number of Patients with at Least One AE related to LGX818	n (%)	xx (xx.x%)	xx (xx.x%)	xx (xx.x%)
Number of Patients with at Least One AE related to MEK162	n (%)	xx (xx.x%)	xx (xx.x%)	xx (xx.x%)
Number of Patients with at Least One AE related to Combo Target Therapy	n (%)	xx (xx.x%)	xx (xx.x%)	xx (xx.x%)
Number of Patients with at Least One serious AE (SAE)	n (%)	xx (xx.x%)	xx (xx.x%)	xx (xx.x%)
Number of Patients with at Least One SAE related to Ipilimumab	n (%)	xx (xx.x%)	xx (xx.x%)	xx (xx.x%)
Number of Patients with at Least One SAE related to Nivolumab	n (%)	xx (xx.x%)	xx (xx.x%)	xx (xx.x%)
Number of Patients with at Least One SAE related to Combo Immuno Therapy	n (%)	xx (xx.x%)	xx (xx.x%)	xx (xx.x%)
Number of Patients with at Least One SAE related to Combo LGX818	n (%)	xx (xx.x%)	xx (xx.x%)	xx (xx.x%)
Number of Patients with at Least One SAE related to Combo MEK162	n (%)	xx (xx.x%)	xx (xx.x%)	xx (xx.x%)
Number of Patients with at Least One SAE related to Combo Target Therapy	n (%)	xx (xx.x%)	xx (xx.x%)	xx (xx.x%)
Number of Patients with at Least One AE leading to hospitalization	n (%)	xx (xx.x%)	xx (xx.x%)	xx (xx.x%)
Number of Patients with at Least One AE leading to withdrawal from the study	n (%)	xx (xx.x%)	xx (xx.x%)	xx (xx.x%)
Number of Patients with at Least One AESI	n (%)	xx (xx.x%)	xx (xx.x%)	xx (xx.x%)

Note: a) Percentages are calculated relative to the total number of patients in the SAF population by treatment arm.
b) An AE is defined as "related" to a study drug if relationship is "Certain", "Probable" or "Possible" to that specific drug. Missing data were considered as Related.

Source: XXXX.SAS, Run on DDMMMYYYY

TABLE 46. Incidence of AEs by primary System Organ Class and Preferred Term

POPULATION: SAF

	Statistic	Arm A (N = XX)	Arm B (N = XX)	Arm C (N = XX)
<Primary SOC>	n (%) E	xx (xx.x%) xx	xx (xx.x%) xx	xx (xx.x%) xx
<PT>	n (%) E	xx (xx.x%) xx	xx (xx.x%) xx	xx (xx.x%) xx
<PT>	n (%) E	xx (xx.x%) xx	xx (xx.x%) xx	xx (xx.x%) xx
<PT>	n (%) E	xx (xx.x%) xx	xx (xx.x%) xx	xx (xx.x%) xx
<Primary SOC>	n (%) E	xx (xx.x%) xx	xx (xx.x%) xx	xx (xx.x%) xx
<PT>	n (%) E	xx (xx.x%) xx	xx (xx.x%) xx	xx (xx.x%) xx
<PT>	n (%) E	xx (xx.x%) xx	xx (xx.x%) xx	xx (xx.x%) xx
<PT>	n (%) E	xx (xx.x%) xx	xx (xx.x%) xx	xx (xx.x%) xx
<Primary SOC>	n (%) E	xx (xx.x%) xx	xx (xx.x%) xx	xx (xx.x%) xx
<PT>	n (%) E	xx (xx.x%) xx	xx (xx.x%) xx	xx (xx.x%) xx
<PT>	n (%) E	xx (xx.x%) xx	xx (xx.x%) xx	xx (xx.x%) xx
<PT>	n (%) E	xx (xx.x%) xx	xx (xx.x%) xx	xx (xx.x%) xx
<Primary SOC>	n (%) E	xx (xx.x%) xx	xx (xx.x%) xx	xx (xx.x%) xx
<PT>	n (%) E	xx (xx.x%) xx	xx (xx.x%) xx	xx (xx.x%) xx
<PT>	n (%) E	xx (xx.x%) xx	xx (xx.x%) xx	xx (xx.x%) xx
<PT>	n (%) E	xx (xx.x%) xx	xx (xx.x%) xx	xx (xx.x%) xx

Note: a) Percentages are calculated relative to the total number of patients in the SAF Population by treatment arm.
b) Each patient is counted at most once within each SOC and PT.
c) AEs were coded using MedDRA version 20.0.
d) n = number of patients, E = number of events.

Source: XXXX.SAS, Run on DDMMMYYYY

TABLE 47. Incidence of Ipilimumab-related AEs by primary System Organ Class and Preferred Term

POPULATION: SAF

	Statistic	Arm A (N = XX)	Arm B (N = XX)	Arm C (N = XX)
<Primary SOC>	n (%) E	xx (xx.x%) xx	xx (xx.x%) xx	xx (xx.x%) xx
<PT>	n (%) E	xx (xx.x%) xx	xx (xx.x%) xx	xx (xx.x%) xx
<PT>	n (%) E	xx (xx.x%) xx	xx (xx.x%) xx	xx (xx.x%) xx
<PT>	n (%) E	xx (xx.x%) xx	xx (xx.x%) xx	xx (xx.x%) xx
<Primary SOC>	n (%) E	xx (xx.x%) xx	xx (xx.x%) xx	xx (xx.x%) xx
<PT>	n (%) E	xx (xx.x%) xx	xx (xx.x%) xx	xx (xx.x%) xx
<PT>	n (%) E	xx (xx.x%) xx	xx (xx.x%) xx	xx (xx.x%) xx
<PT>	n (%) E	xx (xx.x%) xx	xx (xx.x%) xx	xx (xx.x%) xx
<Primary SOC>	n (%) E	xx (xx.x%) xx	xx (xx.x%) xx	xx (xx.x%) xx
<PT>	n (%) E	xx (xx.x%) xx	xx (xx.x%) xx	xx (xx.x%) xx
<PT>	n (%) E	xx (xx.x%) xx	xx (xx.x%) xx	xx (xx.x%) xx
<PT>	n (%) E	xx (xx.x%) xx	xx (xx.x%) xx	xx (xx.x%) xx
<Primary SOC>	n (%) E	xx (xx.x%) xx	xx (xx.x%) xx	xx (xx.x%) xx
<PT>	n (%) E	xx (xx.x%) xx	xx (xx.x%) xx	xx (xx.x%) xx
<PT>	n (%) E	xx (xx.x%) xx	xx (xx.x%) xx	xx (xx.x%) xx
<PT>	n (%) E	xx (xx.x%) xx	xx (xx.x%) xx	xx (xx.x%) xx

Note: a) Percentages are calculated relative to the total number of patients in the SAF Population by treatment arm.
b) Each patient is counted at most once within each SOC and PT.
c) AEs were coded using MedDRA version XX.X.
d) n = number of patients, E = number of events.
e) An AE is "related" to a study drug if relationship is "Certain", "Probable" or "Possible". Missing data were considered as Related.

Source: XXXX.SAS, Run on DDMMMYYYY

TABLE 48. Incidence of Nivolumab-related AEs by primary System Organ Class and Preferred Term

POPULATION: SAF

	Statistic	Arm A (N = XX)	Arm B (N = XX)	Arm C (N = XX)
<Primary SOC>	n (%) E	xx (xx.x%) xx	xx (xx.x%) xx	xx (xx.x%) xx
<PT>	n (%) E	xx (xx.x%) xx	xx (xx.x%) xx	xx (xx.x%) xx
<PT>	n (%) E	xx (xx.x%) xx	xx (xx.x%) xx	xx (xx.x%) xx
<PT>	n (%) E	xx (xx.x%) xx	xx (xx.x%) xx	xx (xx.x%) xx
<Primary SOC>	n (%) E	xx (xx.x%) xx	xx (xx.x%) xx	xx (xx.x%) xx
<PT>	n (%) E	xx (xx.x%) xx	xx (xx.x%) xx	xx (xx.x%) xx
<PT>	n (%) E	xx (xx.x%) xx	xx (xx.x%) xx	xx (xx.x%) xx
<PT>	n (%) E	xx (xx.x%) xx	xx (xx.x%) xx	xx (xx.x%) xx
<Primary SOC>	n (%) E	xx (xx.x%) xx	xx (xx.x%) xx	xx (xx.x%) xx
<PT>	n (%) E	xx (xx.x%) xx	xx (xx.x%) xx	xx (xx.x%) xx
<PT>	n (%) E	xx (xx.x%) xx	xx (xx.x%) xx	xx (xx.x%) xx
<PT>	n (%) E	xx (xx.x%) xx	xx (xx.x%) xx	xx (xx.x%) xx
<Primary SOC>	n (%) E	xx (xx.x%) xx	xx (xx.x%) xx	xx (xx.x%) xx
<PT>	n (%) E	xx (xx.x%) xx	xx (xx.x%) xx	xx (xx.x%) xx
<PT>	n (%) E	xx (xx.x%) xx	xx (xx.x%) xx	xx (xx.x%) xx
<PT>	n (%) E	xx (xx.x%) xx	xx (xx.x%) xx	xx (xx.x%) xx

Note: a) Percentages are calculated relative to the total number of patients in the SAF Population by treatment arm.
b) Each patient is counted at most once within each SOC and PT.
c) AEs were coded using MedDRA version XX.X.
d) n = number of patients, E = number of events.
e) An AE is “related” to a study drug if relationship is “Certain”, “Probable” or “Possible”. Missing data were considered as Related.

Source: XXXX.SAS, Run on DDMMMYYYY

TABLE 49. Incidence of Combo Immuno Therapy-related AEs by primary System Organ Class and Preferred Term

POPULATION: SAF

	Statistic	Arm A (N = XX)	Arm B (N = XX)	Arm C (N = XX)
<Primary SOC>	n (%) E	xx (xx.x%) xx	xx (xx.x%) xx	xx (xx.x%) xx
<PT>	n (%) E	xx (xx.x%) xx	xx (xx.x%) xx	xx (xx.x%) xx
<PT>	n (%) E	xx (xx.x%) xx	xx (xx.x%) xx	xx (xx.x%) xx
<PT>	n (%) E	xx (xx.x%) xx	xx (xx.x%) xx	xx (xx.x%) xx
<Primary SOC>	n (%) E	xx (xx.x%) xx	xx (xx.x%) xx	xx (xx.x%) xx
<PT>	n (%) E	xx (xx.x%) xx	xx (xx.x%) xx	xx (xx.x%) xx
<PT>	n (%) E	xx (xx.x%) xx	xx (xx.x%) xx	xx (xx.x%) xx
<PT>	n (%) E	xx (xx.x%) xx	xx (xx.x%) xx	xx (xx.x%) xx
<Primary SOC>	n (%) E	xx (xx.x%) xx	xx (xx.x%) xx	xx (xx.x%) xx
<PT>	n (%) E	xx (xx.x%) xx	xx (xx.x%) xx	xx (xx.x%) xx
<PT>	n (%) E	xx (xx.x%) xx	xx (xx.x%) xx	xx (xx.x%) xx
<PT>	n (%) E	xx (xx.x%) xx	xx (xx.x%) xx	xx (xx.x%) xx
<Primary SOC>	n (%) E	xx (xx.x%) xx	xx (xx.x%) xx	xx (xx.x%) xx
<PT>	n (%) E	xx (xx.x%) xx	xx (xx.x%) xx	xx (xx.x%) xx
<PT>	n (%) E	xx (xx.x%) xx	xx (xx.x%) xx	xx (xx.x%) xx
<PT>	n (%) E	xx (xx.x%) xx	xx (xx.x%) xx	xx (xx.x%) xx

Note: a) Percentages are calculated relative to the total number of patients in the SAF Population by treatment arm.
b) Each patient is counted at most once within each SOC and PT.
c) AEs were coded using MedDRA version XX.X.
d) n = number of patients, E = number of events.
e) An AE is “related” to a study drug if relationship is “Certain”, “Probable” or “Possible”. Missing data were considered as Related.

Source: XXXX.SAS, Run on DDMMMYYYY

TABLE 50. Incidence of LGX818-related AEs by primary System Organ Class and Preferred Term

POPULATION: SAF

	Statistic	Arm A (N = XX)	Arm B (N = XX)	Arm C (N = XX)
<Primary SOC>	n (%) E	xx (xx.x%) xx	xx (xx.x%) xx	xx (xx.x%) xx
<PT>	n (%) E	xx (xx.x%) xx	xx (xx.x%) xx	xx (xx.x%) xx
<PT>	n (%) E	xx (xx.x%) xx	xx (xx.x%) xx	xx (xx.x%) xx
<PT>	n (%) E	xx (xx.x%) xx	xx (xx.x%) xx	xx (xx.x%) xx
<Primary SOC>	n (%) E	xx (xx.x%) xx	xx (xx.x%) xx	xx (xx.x%) xx
<PT>	n (%) E	xx (xx.x%) xx	xx (xx.x%) xx	xx (xx.x%) xx
<PT>	n (%) E	xx (xx.x%) xx	xx (xx.x%) xx	xx (xx.x%) xx
<PT>	n (%) E	xx (xx.x%) xx	xx (xx.x%) xx	xx (xx.x%) xx
<Primary SOC>	n (%) E	xx (xx.x%) xx	xx (xx.x%) xx	xx (xx.x%) xx
<PT>	n (%) E	xx (xx.x%) xx	xx (xx.x%) xx	xx (xx.x%) xx
<PT>	n (%) E	xx (xx.x%) xx	xx (xx.x%) xx	xx (xx.x%) xx
<PT>	n (%) E	xx (xx.x%) xx	xx (xx.x%) xx	xx (xx.x%) xx
<Primary SOC>	n (%) E	xx (xx.x%) xx	xx (xx.x%) xx	xx (xx.x%) xx
<PT>	n (%) E	xx (xx.x%) xx	xx (xx.x%) xx	xx (xx.x%) xx
<PT>	n (%) E	xx (xx.x%) xx	xx (xx.x%) xx	xx (xx.x%) xx
<PT>	n (%) E	xx (xx.x%) xx	xx (xx.x%) xx	xx (xx.x%) xx

Note: a) Percentages are calculated relative to the total number of patients in the SAF Population by treatment arm.
b) Each patient is counted at most once within each SOC and PT.
c) AEs were coded using MedDRA version XX.X.
d) n = number of patients, E = number of events.
e) An AE is “related” to a study drug if relationship is “Certain”, “Probable” or “Possible”. Missing data were considered as Related.

Source: XXXX.SAS, Run on DDMMMYYYY

TABLE 51. Incidence of MEK162-related AEs by primary System Organ Class and Preferred Term

POPULATION: SAF

	Statistic	Arm A (N = XX)	Arm B (N = XX)	Arm C (N = XX)
<Primary SOC>	n (%) E	xx (xx.x%) xx	xx (xx.x%) xx	xx (xx.x%) xx
<PT>	n (%) E	xx (xx.x%) xx	xx (xx.x%) xx	xx (xx.x%) xx
<PT>	n (%) E	xx (xx.x%) xx	xx (xx.x%) xx	xx (xx.x%) xx
<PT>	n (%) E	xx (xx.x%) xx	xx (xx.x%) xx	xx (xx.x%) xx
<Primary SOC>	n (%) E	xx (xx.x%) xx	xx (xx.x%) xx	xx (xx.x%) xx
<PT>	n (%) E	xx (xx.x%) xx	xx (xx.x%) xx	xx (xx.x%) xx
<PT>	n (%) E	xx (xx.x%) xx	xx (xx.x%) xx	xx (xx.x%) xx
<PT>	n (%) E	xx (xx.x%) xx	xx (xx.x%) xx	xx (xx.x%) xx
<Primary SOC>	n (%) E	xx (xx.x%) xx	xx (xx.x%) xx	xx (xx.x%) xx
<PT>	n (%) E	xx (xx.x%) xx	xx (xx.x%) xx	xx (xx.x%) xx
<PT>	n (%) E	xx (xx.x%) xx	xx (xx.x%) xx	xx (xx.x%) xx
<PT>	n (%) E	xx (xx.x%) xx	xx (xx.x%) xx	xx (xx.x%) xx
<Primary SOC>	n (%) E	xx (xx.x%) xx	xx (xx.x%) xx	xx (xx.x%) xx
<PT>	n (%) E	xx (xx.x%) xx	xx (xx.x%) xx	xx (xx.x%) xx
<PT>	n (%) E	xx (xx.x%) xx	xx (xx.x%) xx	xx (xx.x%) xx
<PT>	n (%) E	xx (xx.x%) xx	xx (xx.x%) xx	xx (xx.x%) xx

Note: a) Percentages are calculated relative to the total number of patients in the SAF Population by treatment arm.
b) Each patient is counted at most once within each SOC and PT.
c) AEs were coded using MedDRA version XX.X.
d) n = number of patients, E = number of events.
e) An AE is "related" to a study drug if relationship is "Certain", "Probable" or "Possible". Missing data were considered as Related.

Source: XXXX.SAS, Run on DDMMMYYYY

TABLE 52. Incidence of Combo Target Therapy-related AEs by primary System Organ Class and Preferred Term

POPULATION: SAF

	Statistic	Arm A (N = XX)	Arm B (N = XX)	Arm C (N = XX)
<Primary SOC>	n (%) E	xx (xx.x%) xx	xx (xx.x%) xx	xx (xx.x%) xx
<PT>	n (%) E	xx (xx.x%) xx	xx (xx.x%) xx	xx (xx.x%) xx
<PT>	n (%) E	xx (xx.x%) xx	xx (xx.x%) xx	xx (xx.x%) xx
<PT>	n (%) E	xx (xx.x%) xx	xx (xx.x%) xx	xx (xx.x%) xx
<Primary SOC>	n (%) E	xx (xx.x%) xx	xx (xx.x%) xx	xx (xx.x%) xx
<PT>	n (%) E	xx (xx.x%) xx	xx (xx.x%) xx	xx (xx.x%) xx
<PT>	n (%) E	xx (xx.x%) xx	xx (xx.x%) xx	xx (xx.x%) xx
<PT>	n (%) E	xx (xx.x%) xx	xx (xx.x%) xx	xx (xx.x%) xx
<Primary SOC>	n (%) E	xx (xx.x%) xx	xx (xx.x%) xx	xx (xx.x%) xx
<PT>	n (%) E	xx (xx.x%) xx	xx (xx.x%) xx	xx (xx.x%) xx
<PT>	n (%) E	xx (xx.x%) xx	xx (xx.x%) xx	xx (xx.x%) xx
<PT>	n (%) E	xx (xx.x%) xx	xx (xx.x%) xx	xx (xx.x%) xx
<Primary SOC>	n (%) E	xx (xx.x%) xx	xx (xx.x%) xx	xx (xx.x%) xx
<PT>	n (%) E	xx (xx.x%) xx	xx (xx.x%) xx	xx (xx.x%) xx
<PT>	n (%) E	xx (xx.x%) xx	xx (xx.x%) xx	xx (xx.x%) xx
<PT>	n (%) E	xx (xx.x%) xx	xx (xx.x%) xx	xx (xx.x%) xx

Note: a) Percentages are calculated relative to the total number of patients in the SAF Population by treatment arm.
b) Each patient is counted at most once within each SOC and PT.
c) AEs were coded using MedDRA version XX.X.
d) n = number of patients, E = number of events.
e) An AE is "related" to a study drug if relationship is "Certain", "Probable" or "Possible". Missing data were considered as Related.

Source: XXXX.SAS, Run on DDMMMYYYY

TABLE 53. Incidence of Serious AEs by primary System Organ Class and Preferred Term

POPULATION: SAF

	Statistic	Arm A (N = XX)	Arm B (N = XX)	Arm C (N = XX)
<Primary SOC>	n (%) E	xx (xx.x%) xx	xx (xx.x%) xx	xx (xx.x%) xx
<PT>	n (%) E	xx (xx.x%) xx	xx (xx.x%) xx	xx (xx.x%) xx
<PT>	n (%) E	xx (xx.x%) xx	xx (xx.x%) xx	xx (xx.x%) xx
<PT>	n (%) E	xx (xx.x%) xx	xx (xx.x%) xx	xx (xx.x%) xx
<Primary SOC>	n (%) E	xx (xx.x%) xx	xx (xx.x%) xx	xx (xx.x%) xx
<PT>	n (%) E	xx (xx.x%) xx	xx (xx.x%) xx	xx (xx.x%) xx
<PT>	n (%) E	xx (xx.x%) xx	xx (xx.x%) xx	xx (xx.x%) xx
<PT>	n (%) E	xx (xx.x%) xx	xx (xx.x%) xx	xx (xx.x%) xx
<Primary SOC>	n (%) E	xx (xx.x%) xx	xx (xx.x%) xx	xx (xx.x%) xx
<PT>	n (%) E	xx (xx.x%) xx	xx (xx.x%) xx	xx (xx.x%) xx
<PT>	n (%) E	xx (xx.x%) xx	xx (xx.x%) xx	xx (xx.x%) xx
<PT>	n (%) E	xx (xx.x%) xx	xx (xx.x%) xx	xx (xx.x%) xx
<Primary SOC>	n (%) E	xx (xx.x%) xx	xx (xx.x%) xx	xx (xx.x%) xx
<PT>	n (%) E	xx (xx.x%) xx	xx (xx.x%) xx	xx (xx.x%) xx
<PT>	n (%) E	xx (xx.x%) xx	xx (xx.x%) xx	xx (xx.x%) xx
<PT>	n (%) E	xx (xx.x%) xx	xx (xx.x%) xx	xx (xx.x%) xx

Note: a) Percentages are calculated relative to the total number of patients in the SAF Population by treatment arm.
b) Each patient is counted at most once within each SOC and PT.
c) AEs were coded using MedDRA version XX.X.
d) n = number of patients, E = number of events.

Source: XXXX.SAS, Run on DDMMMYYYY

TABLE 54. Incidence of AEs leading to hospitalization by primary System Organ Class and Preferred Term

POPULATION: SAF

	Statistic	Arm A (N = XX)	Arm B (N = XX)	Arm C (N = XX)
<Primary SOC>	n (%) E	xx (xx.x%) xx	xx (xx.x%) xx	xx (xx.x%) xx
<PT>	n (%) E	xx (xx.x%) xx	xx (xx.x%) xx	xx (xx.x%) xx
<PT>	n (%) E	xx (xx.x%) xx	xx (xx.x%) xx	xx (xx.x%) xx
<PT>	n (%) E	xx (xx.x%) xx	xx (xx.x%) xx	xx (xx.x%) xx
<Primary SOC>	n (%) E	xx (xx.x%) xx	xx (xx.x%) xx	xx (xx.x%) xx
<PT>	n (%) E	xx (xx.x%) xx	xx (xx.x%) xx	xx (xx.x%) xx
<PT>	n (%) E	xx (xx.x%) xx	xx (xx.x%) xx	xx (xx.x%) xx
<PT>	n (%) E	xx (xx.x%) xx	xx (xx.x%) xx	xx (xx.x%) xx
<Primary SOC>	n (%) E	xx (xx.x%) xx	xx (xx.x%) xx	xx (xx.x%) xx
<PT>	n (%) E	xx (xx.x%) xx	xx (xx.x%) xx	xx (xx.x%) xx
<PT>	n (%) E	xx (xx.x%) xx	xx (xx.x%) xx	xx (xx.x%) xx
<PT>	n (%) E	xx (xx.x%) xx	xx (xx.x%) xx	xx (xx.x%) xx
<Primary SOC>	n (%) E	xx (xx.x%) xx	xx (xx.x%) xx	xx (xx.x%) xx
<PT>	n (%) E	xx (xx.x%) xx	xx (xx.x%) xx	xx (xx.x%) xx
<PT>	n (%) E	xx (xx.x%) xx	xx (xx.x%) xx	xx (xx.x%) xx
<PT>	n (%) E	xx (xx.x%) xx	xx (xx.x%) xx	xx (xx.x%) xx

Note: a) Percentages are calculated relative to the total number of patients in the SAF Population by treatment arm.
b) Each patient is counted at most once within each SOC and PT.
c) AEs were coded using MedDRA version XX.X.
d) n = number of patients, E = number of events.

Source: XXXX.SAS, Run on DDMMMYYYY

TABLE 55. Incidence of AEs leading to study withdrawal by primary System Organ Class and Preferred Term

POPULATION: SAF

	Statistic	Arm A (N = XX)	Arm B (N = XX)	Arm C (N = XX)
<Primary SOC>	n (%) E	xx (xx.x%) xx	xx (xx.x%) xx	xx (xx.x%) xx
<PT>	n (%) E	xx (xx.x%) xx	xx (xx.x%) xx	xx (xx.x%) xx
<PT>	n (%) E	xx (xx.x%) xx	xx (xx.x%) xx	xx (xx.x%) xx
<PT>	n (%) E	xx (xx.x%) xx	xx (xx.x%) xx	xx (xx.x%) xx
<Primary SOC>	n (%) E	xx (xx.x%) xx	xx (xx.x%) xx	xx (xx.x%) xx
<PT>	n (%) E	xx (xx.x%) xx	xx (xx.x%) xx	xx (xx.x%) xx
<PT>	n (%) E	xx (xx.x%) xx	xx (xx.x%) xx	xx (xx.x%) xx
<PT>	n (%) E	xx (xx.x%) xx	xx (xx.x%) xx	xx (xx.x%) xx
<Primary SOC>	n (%) E	xx (xx.x%) xx	xx (xx.x%) xx	xx (xx.x%) xx
<PT>	n (%) E	xx (xx.x%) xx	xx (xx.x%) xx	xx (xx.x%) xx
<PT>	n (%) E	xx (xx.x%) xx	xx (xx.x%) xx	xx (xx.x%) xx
<PT>	n (%) E	xx (xx.x%) xx	xx (xx.x%) xx	xx (xx.x%) xx
<Primary SOC>	n (%) E	xx (xx.x%) xx	xx (xx.x%) xx	xx (xx.x%) xx
<PT>	n (%) E	xx (xx.x%) xx	xx (xx.x%) xx	xx (xx.x%) xx
<PT>	n (%) E	xx (xx.x%) xx	xx (xx.x%) xx	xx (xx.x%) xx
<PT>	n (%) E	xx (xx.x%) xx	xx (xx.x%) xx	xx (xx.x%) xx

Note: a) Percentages are calculated relative to the total number of patients in the SAF Population by treatment arm.
b) Each patient is counted at most once within each SOC and PT.
c) AEs were coded using MedDRA version XX.X.
d) n = number of patients, E = number of events.
e) An AE is “related” to a study drug if relationship is “Certain”, “Probable” or “Possible”. Missing data have been considered as Related.

Source: XXXX.SAS, Run on DDMMMYYYY

TABLE 56. Incidence of AESIs by primary System Organ Class and Preferred Term

POPULATION: SAF

	Statistic	Arm A (N = XX)	Arm B (N = XX)	Arm C (N = XX)
<Primary SOC>	n (%) E	xx (xx.x%) xx	xx (xx.x%) xx	xx (xx.x%) xx
<PT>	n (%) E	xx (xx.x%) xx	xx (xx.x%) xx	xx (xx.x%) xx
<PT>	n (%) E	xx (xx.x%) xx	xx (xx.x%) xx	xx (xx.x%) xx
<PT>	n (%) E	xx (xx.x%) xx	xx (xx.x%) xx	xx (xx.x%) xx
<Primary SOC>	n (%) E	xx (xx.x%) xx	xx (xx.x%) xx	xx (xx.x%) xx
<PT>	n (%) E	xx (xx.x%) xx	xx (xx.x%) xx	xx (xx.x%) xx
<PT>	n (%) E	xx (xx.x%) xx	xx (xx.x%) xx	xx (xx.x%) xx
<PT>	n (%) E	xx (xx.x%) xx	xx (xx.x%) xx	xx (xx.x%) xx
<Primary SOC>	n (%) E	xx (xx.x%) xx	xx (xx.x%) xx	xx (xx.x%) xx
<PT>	n (%) E	xx (xx.x%) xx	xx (xx.x%) xx	xx (xx.x%) xx
<PT>	n (%) E	xx (xx.x%) xx	xx (xx.x%) xx	xx (xx.x%) xx
<PT>	n (%) E	xx (xx.x%) xx	xx (xx.x%) xx	xx (xx.x%) xx
<Primary SOC>	n (%) E	xx (xx.x%) xx	xx (xx.x%) xx	xx (xx.x%) xx
<PT>	n (%) E	xx (xx.x%) xx	xx (xx.x%) xx	xx (xx.x%) xx
<PT>	n (%) E	xx (xx.x%) xx	xx (xx.x%) xx	xx (xx.x%) xx
<PT>	n (%) E	xx (xx.x%) xx	xx (xx.x%) xx	xx (xx.x%) xx

Note: a) Percentages are calculated relative to the total number of patients in the SAF Population by treatment arm.
b) Each patient is counted at most once within each SOC and PT.
c) AEs were coded using MedDRA version XX.X.
d) n = number of patients, E = number of events.

Source: XXXX.SAS, Run on DDMMMYYYY

TABLE 57. Incidence of AEs leading to death by primary System Organ Class and Preferred Term

POPULATION: SAF

	Statistic	Arm A (N = XX)	Arm B (N = XX)	Arm C (N = XX)
<Primary SOC>	n (%) E	xx (xx.x%) xx	xx (xx.x%) xx	xx (xx.x%) xx
<PT>	n (%) E	xx (xx.x%) xx	xx (xx.x%) xx	xx (xx.x%) xx
<PT>	n (%) E	xx (xx.x%) xx	xx (xx.x%) xx	xx (xx.x%) xx
<PT>	n (%) E	xx (xx.x%) xx	xx (xx.x%) xx	xx (xx.x%) xx
<Primary SOC>	n (%) E	xx (xx.x%) xx	xx (xx.x%) xx	xx (xx.x%) xx
<PT>	n (%) E	xx (xx.x%) xx	xx (xx.x%) xx	xx (xx.x%) xx
<PT>	n (%) E	xx (xx.x%) xx	xx (xx.x%) xx	xx (xx.x%) xx
<PT>	n (%) E	xx (xx.x%) xx	xx (xx.x%) xx	xx (xx.x%) xx
<Primary SOC>	n (%) E	xx (xx.x%) xx	xx (xx.x%) xx	xx (xx.x%) xx
<PT>	n (%) E	xx (xx.x%) xx	xx (xx.x%) xx	xx (xx.x%) xx
<PT>	n (%) E	xx (xx.x%) xx	xx (xx.x%) xx	xx (xx.x%) xx
<PT>	n (%) E	xx (xx.x%) xx	xx (xx.x%) xx	xx (xx.x%) xx
<Primary SOC>	n (%) E	xx (xx.x%) xx	xx (xx.x%) xx	xx (xx.x%) xx
<PT>	n (%) E	xx (xx.x%) xx	xx (xx.x%) xx	xx (xx.x%) xx
<PT>	n (%) E	xx (xx.x%) xx	xx (xx.x%) xx	xx (xx.x%) xx
<PT>	n (%) E	xx (xx.x%) xx	xx (xx.x%) xx	xx (xx.x%) xx

Note: a) Percentages are calculated relative to the total number of patients in the SAF Population by treatment arm.
b) Each patient is counted at most once within each SOC and PT.
c) AEs were coded using MedDRA version XX.X.
d) n = number of patients, E = number of events.

Source: XXXX.SAS, Run on DDMMMYYYY

TABLE 58. Hematology – ARM A

POPULATION: SAF **< Note for SAS Programmer: the following must be presented for each hematology parameter >**

	Statistic	Value	Change from Baseline (*)
Hemoglobin			
Baseline 1	n	xx	
	Mean (SD)	xx.x (x.x)	
	Median	xx.x	
	Min/Max	xx/xx	
Week *<Every 4 wks>* – Combo Target	n	xx	xx
	Mean (SD)	xx.x (x.x)	xx.x (x.x)
	Median	xx.x	xx.x
	Min/Max	xx/xx	xx/xx
Baseline 2	n	xx	
	Mean (SD)	xx.x (x.x)	
	Median	xx.x	
	Min/Max	xx/xx	
Week *<Every 3 wks x 4 times>* – Nivolumab + Ipilimumab	n	xx	xx
	Mean (SD)	xx.x (x.x)	xx.x (x.x)
	Median	xx.x	xx.x
	Min/Max	xx/xx	xx/xx
Week *<Every 2 wks>* – Nivolumab	n	xx	xx
	Mean (SD)	xx.x (x.x)	xx.x (x.x)
	Median	xx.x	xx.x
	Min/Max	xx/xx	xx/xx
End of Treatment	n	xx	xx
	Mean (SD)	xx.x (x.x)	xx.x (x.x)
	Median	xx.x	xx.x
	Min/Max	xx/xx	xx/xx
Follow-up	n	xx	xx
	Mean (SD)	xx.x (x.x)	xx.x (x.x)
	Median	xx.x	xx.x
	Min/Max	xx/xx	xx/xx

Notes:
(*) Change from baseline is calculated relative to Baseline 1 for values between Baseline 1 and Baseline 2; Baseline 2 for values after Baseline 2. Change from baseline values include only those patients with both a Baseline value and a value for summarized time period.

n represents number of patients contributing to summary statistics.

Source: XXXX.SAS, Run on DDMMMYYYY

TABLE 59. Hematology – ARM B

POPULATION: SAF **< Note for SAS Programmer: the following must be presented for each hematology parameter >**

	Statistic	Value	Change from Baseline (*)
Hemoglobin			
Baseline 1	n	xx	
	Mean (SD)	xx.x (x.x)	
	Median	xx.x	
	Min/Max	xx/xx	
Week *<Every 3 wks x 4 times>* – Nivolumab + Ipilimumab	n	xx	xx
	Mean (SD)	xx.x (x.x)	xx.x (x.x)
	Median	xx.x	xx.x
	Min/Max	xx/xx	xx/xx
Week *<Every 2 wks>* – Nivolumab	n	xx	xx
	Mean (SD)	xx.x (x.x)	xx.x (x.x)
	Median	xx.x	xx.x
	Min/Max	xx/xx	xx/xx
Baseline 2	n	xx	
	Mean (SD)	xx.x (x.x)	
	Median	xx.x	
	Min/Max	xx/xx	
Week *<Every 4 wks>* – Combo Target	n	xx	xx
	Mean (SD)	xx.x (x.x)	xx.x (x.x)
	Median	xx.x	xx.x
	Min/Max	xx/xx	xx/xx
End of Treatment	n	xx	xx
	Mean (SD)	xx.x (x.x)	xx.x (x.x)
	Median	xx.x	xx.x
	Min/Max	xx/xx	xx/xx
Follow-up	n	xx	xx
	Mean (SD)	xx.x (x.x)	xx.x (x.x)
	Median	xx.x	xx.x
	Min/Max	xx/xx	xx/xx

Notes:
(*) Change from baseline is calculated relative to Baseline 1 for values between Baseline 1 and Baseline 2; Baseline 2 for values after Baseline 2. Change from baseline values include only those patients with both a Baseline value and a value for summarized time period.

n represents number of patients contributing to summary statistics.
Source: XXXX.SAS, Run on DDMMMYYYY

TABLE 60. Hematology – ARM C

POPULATION: SAF **< Note for SAS Programmer: the following must be presented for each hematology parameter >**

	Statistic	Value	Change from Baseline (*)
Hemoglobin			
Baseline 1	n	xx	
	Mean (SD)	xx.x (x.x)	
	Median	xx.x	
	Min/Max	xx/xx	
Day 1 – Combo Target	n	xx	xx
	Mean (SD)	xx.x (x.x)	xx.x (x.x)
	Median	xx.x	xx.x
	Min/Max	xx/xx	xx/xx
Week 4 – Combo Target	n	xx	xx
	Mean (SD)	xx.x (x.x)	xx.x (x.x)
	Median	xx.x	xx.x
	Min/Max	xx/xx	xx/xx
Baseline 2	n	xx	
	Mean (SD)	xx.x (x.x)	
	Median	xx.x	
	Min/Max	xx/xx	
Week *<Every 3 wks x 4 times>* – Nivolumab + Ipilimumab	n	xx	xx
	Mean (SD)	xx.x (x.x)	xx.x (x.x)
	Median	xx.x	xx.x
	Min/Max	xx/xx	xx/xx
Week *<Every 2 wks>* – Nivolumab	n	xx	xx
	Mean (SD)	xx.x (x.x)	xx.x (x.x)
	Median	xx.x	xx.x
	Min/Max	xx/xx	xx/xx
Baseline 3	n	xx	
	Mean (SD)	xx.x (x.x)	
	Median	xx.x	
	Min/Max	xx/xx	
Week *<Every 4 wks>* – Combo Target	n	xx	xx

	Mean (SD)	xx.x (x.x)	xx.x (x.x)
	Median	xx.x	xx.x
	Min/Max	xx/xx	xx/xx

Notes:
(*) Change from baseline is calculated relative to Baseline 1 for values between Baseline 1 and Baseline 2; Baseline 2 for values between Baseline 2 and Baseline 3; Baseline 3 for values after Baseline 3. Change from baseline values include only those patients with both a Baseline value and a value for summarized time period.
n represents number of patients contributing to summary statistics.

Source: XXXX.SAS, Run on DDMMMYYYY

TABLE 60. Hematology – ARM C (cont.)

POPULATION: SAF **< Note for SAS Programmer: the following must be presented for each hematology parameter >**

	Statistic	Value	Change from Baseline (*)
Hemoglobin			
End of Treatment	n	xx	xx
	Mean (SD)	xx.x (x.x)	xx.x (x.x)
	Median	xx.x	xx.x
	Min/Max	xx/xx	xx/xx
Follow-up	n	xx	xx
	Mean (SD)	xx.x (x.x)	xx.x (x.x)
	Median	xx.x	xx.x
	Min/Max	xx/xx	xx/xx

Notes:
(*) Change from baseline is calculated relative to Baseline 1 for values between Baseline 1 and Baseline 2; Baseline 2 for values between Baseline 2 and Baseline 3; Baseline 3 for values after Baseline 3. Change from baseline values include only those patients with both a Baseline value and a value for summarized time period.
n represents number of patients contributing to summary statistics.

Source: XXXX.SAS, Run on DDMMMYYYY

TABLE 61. Blood chemistry – ARM A

POPULATION: SAF **< Note for SAS Programmer: the following must be presented for each blood chemistry parameter >**

	Statistic	Value	Change from Baseline (*)
Glucose			
Baseline 1	n	xx	
	Mean (SD)	xx.x (x.x)	
	Median	xx.x	
	Min/Max	xx/xx	
Week *<Every 4 wks>* – Combo Target	n	xx	xx
	Mean (SD)	xx.x (x.x)	xx.x (x.x)
	Median	xx.x	xx.x
	Min/Max	xx/xx	xx/xx
Baseline 2	n	xx	
	Mean (SD)	xx.x (x.x)	
	Median	xx.x	
	Min/Max	xx/xx	
Week *<Every 3 wks x 4 times>* – Nivolumab + Ipilimumab	n	xx	xx
	Mean (SD)	xx.x (x.x)	xx.x (x.x)
	Median	xx.x	xx.x
	Min/Max	xx/xx	xx/xx
Week *<Every 2 wks>* – Nivolumab	n	xx	xx
	Mean (SD)	xx.x (x.x)	xx.x (x.x)
	Median	xx.x	xx.x
	Min/Max	xx/xx	xx/xx
End of Treatment	n	xx	xx
	Mean (SD)	xx.x (x.x)	xx.x (x.x)
	Median	xx.x	xx.x
	Min/Max	xx/xx	xx/xx
Follow-up	n	xx	xx
	Mean (SD)	xx.x (x.x)	xx.x (x.x)
	Median	xx.x	xx.x
	Min/Max	xx/xx	xx/xx

Notes:
(*) Change from baseline is calculated relative to Baseline 1 for values between Baseline 1 and Baseline 2; Baseline 2 for values after Baseline 2. Change from baseline values include only those patients with both a Baseline value and a value for summarized time period.

n represents number of patients contributing to summary statistics.

Source: XXXX.SAS, Run on DDMMMYYYY

TABLE 62. Blood Chemistry – ARM B

POPULATION: SAF **< Note for SAS Programmer: the following must be presented for each blood chemistry parameter >**

	Statistic	Value	Change from Baseline (*)
Glucose			
Baseline 1	n	xx	
	Mean (SD)	xx.x (x.x)	
	Median	xx.x	
	Min/Max	xx/xx	
Week *<Every 3 wks x 4 times>* – Nivolumab + Ipilimumab	n	xx	xx
	Mean (SD)	xx.x (x.x)	xx.x (x.x)
	Median	xx.x	xx.x
	Min/Max	xx/xx	xx/xx
Week *<Every 2 wks>* – Nivolumab	n	xx	xx
	Mean (SD)	xx.x (x.x)	xx.x (x.x)
	Median	xx.x	xx.x
	Min/Max	xx/xx	xx/xx
Baseline 2	n	xx	
	Mean (SD)	xx.x (x.x)	
	Median	xx.x	
	Min/Max	xx/xx	
Week *<Every 4 wks>* – Combo Target	n	xx	xx
	Mean (SD)	xx.x (x.x)	xx.x (x.x)
	Median	xx.x	xx.x
	Min/Max	xx/xx	xx/xx
End of Treatment	n	xx	xx
	Mean (SD)	xx.x (x.x)	xx.x (x.x)
	Median	xx.x	xx.x
	Min/Max	xx/xx	xx/xx
Follow-up	n	xx	xx
	Mean (SD)	xx.x (x.x)	xx.x (x.x)
	Median	xx.x	xx.x
	Min/Max	xx/xx	xx/xx

Notes:
(*) Change from baseline is calculated relative to Baseline 1 for values between Baseline 1 and Baseline 2; Baseline 2 for values after Baseline 2. Change from baseline values include only those patients with both a Baseline value and a value for summarized time period.

n represents number of patients contributing to summary statistics.

Source: XXXX.SAS, Run on DDMMMYYYY

TABLE 63. Blood Chemistry – ARM C

POPULATION: SAF **< Note for SAS Programmer: the following must be presented for each blood chemistry parameter >**

	Statistic	Value	Change from Baseline (*)
Glucose			
Baseline 1	n	xx	
	Mean (SD)	xx.x (x.x)	
	Median	xx.x	
	Min/Max	xx/xx	
Day 1 – Combo Target	n	xx	xx
	Mean (SD)	xx.x (x.x)	xx.x (x.x)
	Median	xx.x	xx.x
	Min/Max	xx/xx	xx/xx
Week 4 – Combo Target	n	xx	xx
	Mean (SD)	xx.x (x.x)	xx.x (x.x)
	Median	xx.x	xx.x
	Min/Max	xx/xx	xx/xx
Baseline 2	n	xx	
	Mean (SD)	xx.x (x.x)	
	Median	xx.x	
	Min/Max	xx/xx	
Week *<Every 3 wks x 4 times>* – Nivolumab + Ipilimumab	n	xx	xx
	Mean (SD)	xx.x (x.x)	xx.x (x.x)
	Median	xx.x	xx.x
	Min/Max	xx/xx	xx/xx
Week *<Every 2 wks>* – Nivolumab	n	xx	xx
	Mean (SD)	xx.x (x.x)	xx.x (x.x)
	Median	xx.x	xx.x
	Min/Max	xx/xx	xx/xx
Baseline 3	n	xx	
	Mean (SD)	xx.x (x.x)	
	Median	xx.x	
	Min/Max	xx/xx	
Week *<Every 4 wks>* – Combo Target	n	xx	xx
	Mean (SD)	xx.x (x.x)	xx.x (x.x)
	Median	xx.x	xx.x
	Min/Max	xx/xx	xx/xx

Notes:
(*) Change from baseline is calculated relative to Baseline 1 for values between Baseline 1 and Baseline 2; Baseline 2 for values between Baseline 2 and Baseline 3; Baseline 3 for values after Baseline 3. Change from baseline values include only those patients with both a Baseline value and a value for summarized time period.
n represents number of patients contributing to summary statistics.

Source: XXXX.SAS, Run on DDMMMYYYY

TABLE 63. Blood Chemistry – ARM C (cont.)

POPULATION: SAF **< Note for SAS Programmer: the following must be presented for each blood chemistry parameter >**

	Statistic	Value	Change from Baseline (*)
Glucose			
End of Treatment	n	xx	xx
	Mean (SD)	xx.x (x.x)	xx.x (x.x)
	Median	xx.x	xx.x
	Min/Max	xx/xx	xx/xx
Follow-up	n	xx	xx
	Mean (SD)	xx.x (x.x)	xx.x (x.x)
	Median	xx.x	xx.x
	Min/Max	xx/xx	xx/xx

Notes:
(*) Change from baseline is calculated relative to Baseline 1 for values between Baseline 1 and Baseline 2; Baseline 2 for values between Baseline 2 and Baseline 3; Baseline 3 for values after Baseline 3. Change from baseline values include only those patients with both a Baseline value and a value for summarized time period.
n represents number of patients contributing to summary statistics.

Source: XXXX.SAS, Run on DDMMMYYYY

TABLE 64. Coagulation – ARM A

POPULATION: SAF **< Note for SAS Programmer: the following must be presented for each coagulation parameter >**

	Statistic	Value	Change from Baseline (*)
INR			
Baseline 1	n	xx	
	Mean (SD)	xx.x (x.x)	
	Median	xx.x	
	Min/Max	xx/xx	
Week *<Every 4 wks>* – Combo Target	n	xx	xx
	Mean (SD)	xx.x (x.x)	xx.x (x.x)
	Median	xx.x	xx.x
	Min/Max	xx/xx	xx/xx
Baseline 2	n	xx	
	Mean (SD)	xx.x (x.x)	
	Median	xx.x	
	Min/Max	xx/xx	
Week *<Every 3 wks x 4 times>* – Nivolumab + Ipilimumab	n	xx	xx
	Mean (SD)	xx.x (x.x)	xx.x (x.x)
	Median	xx.x	xx.x
	Min/Max	xx/xx	xx/xx
Week *<Every 2 wks>* – Nivolumab	n	xx	xx
	Mean (SD)	xx.x (x.x)	xx.x (x.x)
	Median	xx.x	xx.x
	Min/Max	xx/xx	xx/xx
End of Treatment	n	xx	xx
	Mean (SD)	xx.x (x.x)	xx.x (x.x)
	Median	xx.x	xx.x
	Min/Max	xx/xx	xx/xx
Follow-up	n	xx	xx
	Mean (SD)	xx.x (x.x)	xx.x (x.x)
	Median	xx.x	xx.x
	Min/Max	xx/xx	xx/xx

Notes:
(*) Change from baseline is calculated relative to Baseline 1 for values between Baseline 1 and Baseline 2; Baseline 2 for values after Baseline 2. Change from baseline values include only those patients with both a Baseline value and a value for summarized time period.

n represents number of patients contributing to summary statistics.

Source: XXXX.SAS, Run on DDMMMYYYY

TABLE 65. Coagulation – ARM B

POPULATION: SAF **< Note for SAS Programmer: the following must be presented for each coagulation parameter >**

	Statistic	Value	Change from Baseline (*)
INR			
Baseline 1	n	xx	
	Mean (SD)	xx.x (x.x)	
	Median	xx.x	
	Min/Max	xx/xx	
Week *<Every 3 wks x 4 times>* – Nivolumab + Ipilimumab	n	xx	xx
	Mean (SD)	xx.x (x.x)	xx.x (x.x)
	Median	xx.x	xx.x
	Min/Max	xx/xx	xx/xx
Week *<Every 2 wks>* – Nivolumab	n	xx	xx
	Mean (SD)	xx.x (x.x)	xx.x (x.x)
	Median	xx.x	xx.x
	Min/Max	xx/xx	xx/xx
Baseline 2	n	xx	
	Mean (SD)	xx.x (x.x)	
	Median	xx.x	
	Min/Max	xx/xx	
Week *<Every 4 wks>* – Combo Target	n	xx	xx
	Mean (SD)	xx.x (x.x)	xx.x (x.x)
	Median	xx.x	xx.x
	Min/Max	xx/xx	xx/xx
End of Treatment	n	xx	xx
	Mean (SD)	xx.x (x.x)	xx.x (x.x)
	Median	xx.x	xx.x
	Min/Max	xx/xx	xx/xx
Follow-up	n	xx	xx
	Mean (SD)	xx.x (x.x)	xx.x (x.x)
	Median	xx.x	xx.x
	Min/Max	xx/xx	xx/xx

Notes:
(*) Change from baseline is calculated relative to Baseline 1 for values between Baseline 1 and Baseline 2; Baseline 2 for values after Baseline 2. Change from baseline values include only those patients with both a Baseline value and a value for summarized time period.

n represents number of patients contributing to summary statistics.
Source: XXXX.SAS, Run on DDMMMYYYY

TABLE 66. Coagulation – ARM C

POPULATION: SAF **< Note for SAS Programmer: the following must be presented for each coagulation parameter >**

	Statistic	Value	Change from Baseline (*)
INR			
Baseline 1	n	xx	
	Mean (SD)	xx.x (x.x)	
	Median	xx.x	
	Min/Max	xx/xx	
Day 1 – Combo Target	n	xx	xx
	Mean (SD)	xx.x (x.x)	xx.x (x.x)
	Median	xx.x	xx.x
	Min/Max	xx/xx	xx/xx
Week 4 – Combo Target	n	xx	xx
	Mean (SD)	xx.x (x.x)	xx.x (x.x)
	Median	xx.x	xx.x
	Min/Max	xx/xx	xx/xx
Baseline 2	n	xx	
	Mean (SD)	xx.x (x.x)	
	Median	xx.x	
	Min/Max	xx/xx	
Week *<Every 3 wks x 4 times>* – Nivolumab + Ipilimumab	n	xx	xx
	Mean (SD)	xx.x (x.x)	xx.x (x.x)
	Median	xx.x	xx.x
	Min/Max	xx/xx	xx/xx
Week *<Every 2 wks>* – Nivolumab	n	xx	xx
	Mean (SD)	xx.x (x.x)	xx.x (x.x)
	Median	xx.x	xx.x
	Min/Max	xx/xx	xx/xx
Baseline 3	n	xx	
	Mean (SD)	xx.x (x.x)	
	Median	xx.x	
	Min/Max	xx/xx	
Week *<Every 4 wks>* – Combo Target	n	xx	xx

	Mean (SD)	xx.x (x.x)	xx.x (x.x)
	Median	xx.x	xx.x
	Min/Max	xx/xx	xx/xx

Notes:
(*) Change from baseline is calculated relative to Baseline 1 for values between Baseline 1 and Baseline 2; Baseline 2 for values between Baseline 2 and Baseline 3; Baseline 3 for values after Baseline 3. Change from baseline values include only those patients with both a Baseline value and a value for summarized time period.
n represents number of patients contributing to summary statistics.

Source: XXXX.SAS, Run on DDMMMYYYY

TABLE 66. Coagulation – ARM C (cont.)

POPULATION: SAF **< Note for SAS Programmer: the following must be presented for each coagulation parameter >**

	Statistic	Value	Change from Baseline (*)
INR			
End of Treatment	n	xx	xx
	Mean (SD)	xx.x (x.x)	xx.x (x.x)
	Median	xx.x	xx.x
	Min/Max	xx/xx	xx/xx
Follow-up	n	xx	xx
	Mean (SD)	xx.x (x.x)	xx.x (x.x)
	Median	xx.x	xx.x
	Min/Max	xx/xx	xx/xx

Notes:
(*) Change from baseline is calculated relative to Baseline 1 for values between Baseline 1 and Baseline 2; Baseline 2 for values between Baseline 2 and Baseline 3; Baseline 3 for values after Baseline 3. Change from baseline values include only those patients with both a Baseline value and a value for summarized time period.
n represents number of patients contributing to summary statistics.

Source: XXXX.SAS, Run on DDMMMYYYY

TABLE 67. Thyroid function test – ARM A

POPULATION: SAF **< Note for SAS Programmer: the following must be presented for each thyroid function parameter >**

	Statistic	Value	Change from Baseline (*)
TSH			
Baseline 1	n	xx	
	Mean (SD)	xx.x (x.x)	
	Median	xx.x	
	Min/Max	xx/xx	
Week *<Every 4 wks>* – Combo Target	n	xx	xx
	Mean (SD)	xx.x (x.x)	xx.x (x.x)
	Median	xx.x	xx.x
	Min/Max	xx/xx	xx/xx
Baseline 2	n	xx	
	Mean (SD)	xx.x (x.x)	
	Median	xx.x	
	Min/Max	xx/xx	
Week *<Every 3 wks x 4 times>* – Nivolumab + Ipilimumab	n	xx	xx
	Mean (SD)	xx.x (x.x)	xx.x (x.x)
	Median	xx.x	xx.x
	Min/Max	xx/xx	xx/xx
Week *<Every 2 wks>* – Nivolumab	n	xx	xx
	Mean (SD)	xx.x (x.x)	xx.x (x.x)
	Median	xx.x	xx.x
	Min/Max	xx/xx	xx/xx
End of Treatment	n	xx	xx
	Mean (SD)	xx.x (x.x)	xx.x (x.x)
	Median	xx.x	xx.x
	Min/Max	xx/xx	xx/xx
Follow-up	n	xx	xx
	Mean (SD)	xx.x (x.x)	xx.x (x.x)
	Median	xx.x	xx.x
	Min/Max	xx/xx	xx/xx

Notes:
(*) Change from baseline is calculated relative to Baseline 1 for values between Baseline 1 and Baseline 2; Baseline 2 for values after Baseline 2. Change from baseline values include only those patients with both a Baseline value and a value for summarized time period.

n represents number of patients contributing to summary statistics.

Source: XXXX.SAS, Run on DDMMMYYYY

TABLE 68. Thyroid function test – ARM B

POPULATION: SAF **< Note for SAS Programmer: the following must be presented for each thyroid function parameter >**

	Statistic	Value	Change from Baseline (*)
TSH			
Baseline 1	n	xx	
	Mean (SD)	xx.x (x.x)	
	Median	xx.x	
	Min/Max	xx/xx	
Week *<Every 3 wks x 4 times>* – Nivolumab + Ipilimumab	n	xx	xx
	Mean (SD)	xx.x (x.x)	xx.x (x.x)
	Median	xx.x	xx.x
	Min/Max	xx/xx	xx/xx
Week *<Every 2 wks>* – Nivolumab	n	xx	xx
	Mean (SD)	xx.x (x.x)	xx.x (x.x)
	Median	xx.x	xx.x
	Min/Max	xx/xx	xx/xx
Baseline 2	n	xx	
	Mean (SD)	xx.x (x.x)	
	Median	xx.x	
	Min/Max	xx/xx	
Week *<Every 4 wks>* – Combo Target	n	xx	xx
	Mean (SD)	xx.x (x.x)	xx.x (x.x)
	Median	xx.x	xx.x
	Min/Max	xx/xx	xx/xx
End of Treatment	n	xx	xx
	Mean (SD)	xx.x (x.x)	xx.x (x.x)
	Median	xx.x	xx.x
	Min/Max	xx/xx	xx/xx
Follow-up	n	xx	xx
	Mean (SD)	xx.x (x.x)	xx.x (x.x)
	Median	xx.x	xx.x
	Min/Max	xx/xx	xx/xx

Notes:
(*) Change from baseline is calculated relative to Baseline 1 for values between Baseline 1 and Baseline 2; Baseline 2 for values after Baseline 2. Change from baseline values include only those patients with both a Baseline value and a value for summarized time period.

n represents number of patients contributing to summary statistics.

Source: XXXX.SAS, Run on DDMMMYYYY

TABLE 69. Thyroid function test – ARM C

POPULATION: SAF **< Note for SAS Programmer: the following must be presented for each thyroid function parameter >**

	Statistic	Value	Change from Baseline (*)
TSH			
Baseline 1	n	xx	
	Mean (SD)	xx.x (x.x)	
	Median	xx.x	
	Min/Max	xx/xx	
Day 1 – Combo Target	n	xx	xx
	Mean (SD)	xx.x (x.x)	xx.x (x.x)
	Median	xx.x	xx.x
	Min/Max	xx/xx	xx/xx
Week 4 – Combo Target	n	xx	xx
	Mean (SD)	xx.x (x.x)	xx.x (x.x)
	Median	xx.x	xx.x
	Min/Max	xx/xx	xx/xx
Baseline 2	n	xx	
	Mean (SD)	xx.x (x.x)	
	Median	xx.x	
	Min/Max	xx/xx	
Week *<Every 3 wks x 4 times>* – Nivolumab + Ipilimumab	n	xx	xx
	Mean (SD)	xx.x (x.x)	xx.x (x.x)
	Median	xx.x	xx.x
	Min/Max	xx/xx	xx/xx
Week *<Every 2 wks>* – Nivolumab	n	xx	xx
	Mean (SD)	xx.x (x.x)	xx.x (x.x)
	Median	xx.x	xx.x
	Min/Max	xx/xx	xx/xx
Baseline 3	n	xx	
	Mean (SD)	xx.x (x.x)	
	Median	xx.x	
	Min/Max	xx/xx	
Week *<Every 4 wks>* – Combo Target	n	xx	xx
	Mean (SD)	xx.x (x.x)	xx.x (x.x)
	Median	xx.x	xx.x
	Min/Max	xx/xx	xx/xx

Notes:
(*) Change from baseline is calculated relative to Baseline 1 for values between Baseline 1 and Baseline 2; Baseline 2 for values between Baseline 2 and Baseline 3; Baseline 3 for values after Baseline 3. Change from baseline values include only those patients with both a Baseline value and a value for summarized time period.
n represents number of patients contributing to summary statistics.

Source: XXXX.SAS, Run on DDMMMYYYY

TABLE 69. Thyroid function test – ARM C (cont.)

POPULATION: SAF **< Note for SAS Programmer: the following must be presented for each thyroid function parameter >**

	Statistic	Value	Change from Baseline (*)
TSH			
End of Treatment	n	xx	xx
	Mean (SD)	xx.x (x.x)	xx.x (x.x)
	Median	xx.x	xx.x
	Min/Max	xx/xx	xx/xx
Follow-up	n	xx	xx
	Mean (SD)	xx.x (x.x)	xx.x (x.x)
	Median	xx.x	xx.x
	Min/Max	xx/xx	xx/xx

Notes:
(*) Change from baseline is calculated relative to Baseline 1 for values between Baseline 1 and Baseline 2; Baseline 2 for values between Baseline 2 and Baseline 3; Baseline 3 for values after Baseline 3. Change from baseline values include only those patients with both a Baseline value and a value for summarized time period.
n represents number of patients contributing to summary statistics.

Source: XXXX.SAS, Run on DDMMMYYYY

TABLE 70. Cardiac Muscle Enzymes – ARM A

POPULATION: SAF **< Note for SAS Programmer: the following must be presented for each cardiac muscle enzymes >**

	Statistic	Value	Change from Baseline (*)
Creatine Kinase			
Baseline 1	n	xx	
	Mean (SD)	xx.x (x.x)	
	Median	xx.x	
	Min/Max	xx/xx	
Week *<Every 4 wks>* – Combo Target	n	xx	xx
	Mean (SD)	xx.x (x.x)	xx.x (x.x)
	Median	xx.x	xx.x
	Min/Max	xx/xx	xx/xx
Baseline 2	n	xx	
	Mean (SD)	xx.x (x.x)	
	Median	xx.x	
	Min/Max	xx/xx	
Week *<Every 3 wks x 4 times>* – Nivolumab + Ipilimumab	n	xx	xx
	Mean (SD)	xx.x (x.x)	xx.x (x.x)
	Median	xx.x	xx.x
	Min/Max	xx/xx	xx/xx
Week *<Every 2 wks>* – Nivolumab	n	xx	xx
	Mean (SD)	xx.x (x.x)	xx.x (x.x)
	Median	xx.x	xx.x
	Min/Max	xx/xx	xx/xx
End of Treatment	n	xx	xx
	Mean (SD)	xx.x (x.x)	xx.x (x.x)
	Median	xx.x	xx.x
	Min/Max	xx/xx	xx/xx
Follow-up	n	xx	xx
	Mean (SD)	xx.x (x.x)	xx.x (x.x)
	Median	xx.x	xx.x
	Min/Max	xx/xx	xx/xx

Notes:
(*) Change from baseline is calculated relative to Baseline 1 for values between Baseline 1 and Baseline 2; Baseline 2 for values after Baseline 2. Change from baseline values include only those patients with both a Baseline value and a value for summarized time period.

n represents number of patients contributing to summary statistics.

Source: XXXX.SAS, Run on DDMMMYYYY

TABLE 71. Cardiac Muscle Enzymes – ARM B

POPULATION: SAF **< Note for SAS Programmer: the following must be presented for each cardiac muscle enzymes >**

	Statistic	Value	Change from Baseline (*)
Creatine Kinase			
Baseline 1	n	xx	
	Mean (SD)	xx.x (x.x)	
	Median	xx.x	
	Min/Max	xx/xx	
Week *<Every 3 wks x 4 times>* – Nivolumab + Ipilimumab	n	xx	xx
	Mean (SD)	xx.x (x.x)	xx.x (x.x)
	Median	xx.x	xx.x
	Min/Max	xx/xx	xx/xx
Week *<Every 2 wks>* – Nivolumab	n	xx	xx
	Mean (SD)	xx.x (x.x)	xx.x (x.x)
	Median	xx.x	xx.x
	Min/Max	xx/xx	xx/xx
Baseline 2	n	xx	
	Mean (SD)	xx.x (x.x)	
	Median	xx.x	
	Min/Max	xx/xx	
Week *<Every 4 wks>* – Combo Target	n	xx	xx
	Mean (SD)	xx.x (x.x)	xx.x (x.x)
	Median	xx.x	xx.x
	Min/Max	xx/xx	xx/xx
End of Treatment	n	xx	xx
	Mean (SD)	xx.x (x.x)	xx.x (x.x)
	Median	xx.x	xx.x
	Min/Max	xx/xx	xx/xx
Follow-up	n	xx	xx
	Mean (SD)	xx.x (x.x)	xx.x (x.x)
	Median	xx.x	xx.x
	Min/Max	xx/xx	xx/xx

Notes:
(*) Change from baseline is calculated relative to Baseline 1 for values between Baseline 1 and Baseline 2; Baseline 2 for values after Baseline 2. Change from baseline values include only those patients with both a Baseline value and a value for summarized time period.

n represents number of patients contributing to summary statistics.
Source: XXXX.SAS, Run on DDMMMYYYY

TABLE 72. Cardiac Muscle Enzymes – ARM C

POPULATION: SAF **< Note for SAS Programmer: the following must be presented for each cardiac muscle enzymes >**

	Statistic	Value	Change from Baseline (*)
Creatine Kinase			
Baseline 1	n	xx	
	Mean (SD)	xx.x (x.x)	
	Median	xx.x	
	Min/Max	xx/xx	
Day 1 – Combo Target	n	xx	xx
	Mean (SD)	xx.x (x.x)	xx.x (x.x)
	Median	xx.x	xx.x
	Min/Max	xx/xx	xx/xx
Week 4 – Combo Target	n	xx	xx
	Mean (SD)	xx.x (x.x)	xx.x (x.x)
	Median	xx.x	xx.x
	Min/Max	xx/xx	xx/xx
Baseline 2	n	xx	
	Mean (SD)	xx.x (x.x)	
	Median	xx.x	
	Min/Max	xx/xx	
Week *<Every 3 wks x 4 times>* – Nivolumab + Ipilimumab	n	xx	xx
	Mean (SD)	xx.x (x.x)	xx.x (x.x)
	Median	xx.x	xx.x
	Min/Max	xx/xx	xx/xx
Week *<Every 2 wks>* – Nivolumab	n	xx	xx
	Mean (SD)	xx.x (x.x)	xx.x (x.x)
	Median	xx.x	xx.x
	Min/Max	xx/xx	xx/xx
Baseline 3	n	xx	
	Mean (SD)	xx.x (x.x)	
	Median	xx.x	
	Min/Max	xx/xx	
Week *<Every 4 wks>* – Combo Target	n	xx	xx

	Mean (SD)	xx.x (x.x)	xx.x (x.x)
	Median	xx.x	xx.x
	Min/Max	xx/xx	xx/xx

Notes:
(*) Change from baseline is calculated relative to Baseline 1 for values between Baseline 1 and Baseline 2; Baseline 2 for values between Baseline 2 and Baseline 3; Baseline 3 for values after Baseline 3. Change from baseline values include only those patients with both a Baseline value and a value for summarized time period.
n represents number of patients contributing to summary statistics.

Source: XXXX.SAS, Run on DDMMMYYYY

TABLE 72. Cardiac Muscle Enzymes – ARM C (cont.)

POPULATION: SAF **< Note for SAS Programmer: the following must be presented for each cardiac muscle enzymes >**

	Statistic	Value	Change from Baseline (*)
Creatine Kinase			
End of Treatment	n	xx	xx
	Mean (SD)	xx.x (x.x)	xx.x (x.x)
	Median	xx.x	xx.x
	Min/Max	xx/xx	xx/xx
Follow-up	n	xx	xx
	Mean (SD)	xx.x (x.x)	xx.x (x.x)
	Median	xx.x	xx.x
	Min/Max	xx/xx	xx/xx

Notes:
(*) Change from baseline is calculated relative to Baseline 1 for values between Baseline 1 and Baseline 2; Baseline 2 for values between Baseline 2 and Baseline 3; Baseline 3 for values after Baseline 3. Change from baseline values include only those patients with both a Baseline value and a value for summarized time period.
n represents number of patients contributing to summary statistics.

Source: XXXX.SAS, Run on DDMMMYYYY

TABLE 73. Endocrine Panel – ARM A

POPULATION: SAF **< Note for SAS Programmer: the following must be presented for Total Cortisol as well >**

	Statistic	Value	Change from Baseline (*)
ACTH			
Baseline 1	n	xx	
	Mean (SD)	xx.x (x.x)	
	Median	xx.x	
	Min/Max	xx/xx	
Baseline 2	n	xx	
	Mean (SD)	xx.x (x.x)	
	Median	xx.x	
	Min/Max	xx/xx	
Week *<Every 3 wks x 4 times>* – Nivolumab + Ipilimumab	n	xx	xx
	Mean (SD)	xx.x (x.x)	xx.x (x.x)
	Median	xx.x	xx.x
	Min/Max	xx/xx	xx/xx
Week *<Every 2 wks>* – Nivolumab	n	xx	xx
	Mean (SD)	xx.x (x.x)	xx.x (x.x)
	Median	xx.x	xx.x
	Min/Max	xx/xx	xx/xx
End of Treatment	n	xx	xx
	Mean (SD)	xx.x (x.x)	xx.x (x.x)
	Median	xx.x	xx.x
	Min/Max	xx/xx	xx/xx
Follow-up	n	xx	xx
	Mean (SD)	xx.x (x.x)	xx.x (x.x)
	Median	xx.x	xx.x
	Min/Max	xx/xx	xx/xx

Notes:
(*) Change from baseline is calculated relative to Baseline 1 for values between Baseline 1 and Baseline 2; Baseline 2 for values after Baseline 2. Change from baseline values include only those patients with both a Baseline value and a value for summarized time period.
n represents number of patients contributing to summary statistics.

Source: XXXX.SAS, Run on DDMMMYYYY

TABLE 74. Endocrine Panel – ARM B

POPULATION: SAF **< Note for SAS Programmer: the following must be presented for Total Cortisol as well >**

	Statistic	Value	Change from Baseline (*)
ACTH			
Baseline 1	n	xx	
	Mean (SD)	xx.x (x.x)	
	Median	xx.x	
	Min/Max	xx/xx	
Week *<Every 3 wks x 4 times>* – Nivolumab + Ipilimumab	n	xx	xx
	Mean (SD)	xx.x (x.x)	xx.x (x.x)
	Median	xx.x	xx.x
	Min/Max	xx/xx	xx/xx
Week *<Every 2 wks>* – Nivolumab	n	xx	xx
	Mean (SD)	xx.x (x.x)	xx.x (x.x)
	Median	xx.x	xx.x
	Min/Max	xx/xx	xx/xx
Baseline 2	n	xx	
	Mean (SD)	xx.x (x.x)	
	Median	xx.x	
	Min/Max	xx/xx	
End of Treatment	n	xx	xx
	Mean (SD)	xx.x (x.x)	xx.x (x.x)
	Median	xx.x	xx.x
	Min/Max	xx/xx	xx/xx
Follow-up	n	xx	xx
	Mean (SD)	xx.x (x.x)	xx.x (x.x)
	Median	xx.x	xx.x
	Min/Max	xx/xx	xx/xx

Notes:
(*) Change from baseline is calculated relative to Baseline 1 for values between Baseline 1 and Baseline 2; Baseline 2 for values after Baseline 2. Change from baseline values include only those patients with both a Baseline value and a value for summarized time period.
n represents number of patients contributing to summary statistics.

Source: XXXX.SAS, Run on DDMMMYYYY

TABLE 75. Endocrine Panel – ARM C

POPULATION: SAF **< Note for SAS Programmer: the following must be presented for Total Cortisol as well >**

	Statistic	Value	Change from Baseline (*)
ACTH			
Baseline 1	n	xx	
	Mean (SD)	xx.x (x.x)	
	Median	xx.x	
	Min/Max	xx/xx	
Baseline 2	n	xx	
	Mean (SD)	xx.x (x.x)	
	Median	xx.x	
	Min/Max	xx/xx	
Week *<Every 3 wks x 4 times>* – Nivolumab + Ipilimumab	n	xx	xx
	Mean (SD)	xx.x (x.x)	xx.x (x.x)
	Median	xx.x	xx.x
	Min/Max	xx/xx	xx/xx
Week *<Every 2 wks>* – Nivolumab	n	xx	xx
	Mean (SD)	xx.x (x.x)	xx.x (x.x)
	Median	xx.x	xx.x
	Min/Max	xx/xx	xx/xx
Baseline 3	n	xx	
	Mean (SD)	xx.x (x.x)	
	Median	xx.x	
	Min/Max	xx/xx	
End of Treatment	n	xx	xx
	Mean (SD)	xx.x (x.x)	xx.x (x.x)
	Median	xx.x	xx.x
	Min/Max	xx/xx	xx/xx
Follow-up	n	xx	xx
	Mean (SD)	xx.x (x.x)	xx.x (x.x)
	Median	xx.x	xx.x
	Min/Max	xx/xx	xx/xx

Notes:
(*) Change from baseline is calculated relative to Baseline 1 for values between Baseline 1 and Baseline 2; Baseline 2 for values between Baseline 2 and Baseline 3; Baseline 3 for values after Baseline 3. Change from baseline values include only those patients with both a Baseline value and a value for summarized time period.

n represents number of patients contributing to summary statistics.

Source: XXXX.SAS, Run on DDMMMYYYY

TABLE 76. Urinalysis (categorical parameters – Part I) – ARM A

POPULATION: SAF **< Note for SAS Programmer: the following must be presented for protein, blood, bilirubin, ketones, leukocytes as well >**

	Statistic	Arm A (N=XX)
Glucose		
Baseline 1		
Neg	n (%)	xx (xx.x%)
Trace	n (%)	xx (xx.x%)
1+	n (%)	xx (xx.x%)
2+	n (%)	xx (xx.x%)
3+	n (%)	xx (xx.x%)
4+	n (%)	xx (xx.x%)
Week *<Every 4 wks>* – Combo Target		
Neg	n (%)	xx (xx.x%)
Trace	n (%)	xx (xx.x%)
1+	n (%)	xx (xx.x%)
2+	n (%)	xx (xx.x%)
3+	n (%)	xx (xx.x%)
4+	n (%)	xx (xx.x%)
Baseline 2		
Neg	n (%)	xx (xx.x%)
Trace	n (%)	xx (xx.x%)
1+	n (%)	xx (xx.x%)
2+	n (%)	xx (xx.x%)
3+	n (%)	xx (xx.x%)
4+	n (%)	xx (xx.x%)
Week *<Every 3 wks x 4 times>* – Nivolumab + Ipilimumab		
Neg	n (%)	xx (xx.x%)
Trace	n (%)	xx (xx.x%)
1+	n (%)	xx (xx.x%)
2+	n (%)	xx (xx.x%)
3+	n (%)	xx (xx.x%)
4+	n (%)	xx (xx.x%)
Week *<Every 2 wks>* – Nivolumab		
Neg	n (%)	xx (xx.x%)
Trace	n (%)	xx (xx.x%)
1+	n (%)	xx (xx.x%)
2+	n (%)	xx (xx.x%)
3+	n (%)	xx (xx.x%)
4+	n (%)	xx (xx.x%)

Notes:
Percentages are calculated relative to the total number of patients of ARM A in the SAF set.

Source: XXXX.SAS, Run on DDMMMYYYY

TABLE 76. Urinalysis (categorical parameters – Part I) – ARM A (cont.)

POPULATION: SAF **< Note for SAS Programmer: the following must be presented for protein, blood, bilirubin, ketones, leukocytes as well >**

	Statistic	Arm A (N=XX)
End of Treatment		
Neg	n (%)	xx (xx.x%)
Trace	n (%)	xx (xx.x%)
1+	n (%)	xx (xx.x%)
2+	n (%)	xx (xx.x%)
3+	n (%)	xx (xx.x%)
4+	n (%)	xx (xx.x%)
Follow-up		
Neg	n (%)	xx (xx.x%)
Trace	n (%)	xx (xx.x%)
1+	n (%)	xx (xx.x%)
2+	n (%)	xx (xx.x%)
3+	n (%)	xx (xx.x%)
4+	n (%)	xx (xx.x%)

Notes:
Percentages are calculated relative to the total number of patients of ARM A in the SAF set.

Source: XXXX.SAS, Run on DDMMMYYYY

TABLE 77. Urinalysis (categorical parameters – Part I) – ARM B

POPULATION: SAF **< Note for SAS Programmer: the following must be presented for protein, blood, bilirubin, ketones, leukocytes as well >**

	Statistic	Arm B (N=XX)
Glucose		
Baseline 1		
Neg	n (%)	xx (xx.x%)
Trace	n (%)	xx (xx.x%)
1+	n (%)	xx (xx.x%)
2+	n (%)	xx (xx.x%)
3+	n (%)	xx (xx.x%)
4+	n (%)	xx (xx.x%)
Week *<Every 3 wks x 4 times>* – Nivolumab + Ipilimumab		
Neg	n (%)	xx (xx.x%)
Trace	n (%)	xx (xx.x%)
1+	n (%)	xx (xx.x%)
2+	n (%)	xx (xx.x%)
3+	n (%)	xx (xx.x%)
4+	n (%)	xx (xx.x%)
Week *<Every 2 wks>* – Nivolumab		
Neg	n (%)	xx (xx.x%)
Trace	n (%)	xx (xx.x%)
1+	n (%)	xx (xx.x%)
2+	n (%)	xx (xx.x%)
3+	n (%)	xx (xx.x%)
4+	n (%)	xx (xx.x%)
Baseline 2		
Neg	n (%)	xx (xx.x%)
Trace	n (%)	xx (xx.x%)
1+	n (%)	xx (xx.x%)
2+	n (%)	xx (xx.x%)
3+	n (%)	xx (xx.x%)
4+	n (%)	xx (xx.x%)
Week *<Every 4 wks>* – Combo Target		
Neg	n (%)	xx (xx.x%)
Trace	n (%)	xx (xx.x%)
1+	n (%)	xx (xx.x%)
2+	n (%)	xx (xx.x%)
3+	n (%)	xx (xx.x%)
4+	n (%)	xx (xx.x%)

Notes:
Percentages are calculated relative to the total number of patients of ARM B in the SAF set.

Source: XXXX.SAS, Run on DDMMMYYYY

TABLE 77. Urinalysis (categorical parameters – Part I) – ARM B (cont.)

POPULATION: SAF **< Note for SAS Programmer: the following must be presented for protein, blood, bilirubin, ketones, leukocytes as well >**

	Statistic	Arm B (N=XX)
End of Treatment		
Neg	n (%)	xx (xx.x%)
Trace	n (%)	xx (xx.x%)
1+	n (%)	xx (xx.x%)
2+	n (%)	xx (xx.x%)
3+	n (%)	xx (xx.x%)
4+	n (%)	xx (xx.x%)
Follow-up		
Neg	n (%)	xx (xx.x%)
Trace	n (%)	xx (xx.x%)
1+	n (%)	xx (xx.x%)
2+	n (%)	xx (xx.x%)
3+	n (%)	xx (xx.x%)
4+	n (%)	xx (xx.x%)

Notes:
Percentages are calculated relative to the total number of patients of ARM B in the SAF set.

Source: XXXX.SAS, Run on DDMMMYYYY

TABLE 78. Urinalysis (categorical parameters – Part I) – ARM C

POPULATION: SAF **< Note for SAS Programmer: the following must be presented for protein, blood, bilirubin, ketones, leukocytes as well >**

	Statistic	Arm C (N=XX)
Glucose		
Baseline 1		
Neg	n (%)	xx (xx.x%)
Trace	n (%)	xx (xx.x%)
1+	n (%)	xx (xx.x%)
2+	n (%)	xx (xx.x%)
3+	n (%)	xx (xx.x%)
4+	n (%)	xx (xx.x%)
Day 1 – Combo Target		
Neg	n (%)	xx (xx.x%)
Trace	n (%)	xx (xx.x%)
1+	n (%)	xx (xx.x%)
2+	n (%)	xx (xx.x%)
3+	n (%)	xx (xx.x%)
4+	n (%)	xx (xx.x%)
Week 4 – Combo Target		
Neg	n (%)	xx (xx.x%)
Trace	n (%)	xx (xx.x%)
1+	n (%)	xx (xx.x%)
2+	n (%)	xx (xx.x%)
3+	n (%)	xx (xx.x%)
4+	n (%)	xx (xx.x%)
Baseline 2		
Neg	n (%)	xx (xx.x%)
Trace	n (%)	xx (xx.x%)
1+	n (%)	xx (xx.x%)
2+	n (%)	xx (xx.x%)
3+	n (%)	xx (xx.x%)
4+	n (%)	xx (xx.x%)
Week *<Every 3 wks x 4 times>* – Nivolumab + Ipilimumab		
Neg	n (%)	xx (xx.x%)
Trace	n (%)	xx (xx.x%)
1+	n (%)	xx (xx.x%)
2+	n (%)	xx (xx.x%)
3+	n (%)	xx (xx.x%)
4+	n (%)	xx (xx.x%)

Notes:
Percentages are calculated relative to the total number of patients of ARM C in the SAF set.

Source: XXXX.SAS, Run on DDMMMYYYY

TABLE 78. Urinalysis (categorical parameters – Part I) – ARM C (cont.)

POPULATION: SAF **< Note for SAS Programmer: the following must be presented for protein, blood, bilirubin, ketones, leukocytes as well >**

	Statistic	Arm C (N=XX)
Glucose		
Week *<Every 2 wks>* – Nivolumab		
Neg	n (%)	xx (xx.x%)
Trace	n (%)	xx (xx.x%)
1+	n (%)	xx (xx.x%)
2+	n (%)	xx (xx.x%)
3+	n (%)	xx (xx.x%)
4+	n (%)	xx (xx.x%)
Baseline 3		
Neg	n (%)	xx (xx.x%)
Trace	n (%)	xx (xx.x%)
1+	n (%)	xx (xx.x%)
2+	n (%)	xx (xx.x%)
3+	n (%)	xx (xx.x%)
4+	n (%)	xx (xx.x%)
Week *<Every 4 wks>* – Combo Target		
Neg	n (%)	xx (xx.x%)
Trace	n (%)	xx (xx.x%)
1+	n (%)	xx (xx.x%)
2+	n (%)	xx (xx.x%)
3+	n (%)	xx (xx.x%)
4+	n (%)	xx (xx.x%)
End of Treatment		
Neg	n (%)	xx (xx.x%)
Trace	n (%)	xx (xx.x%)
1+	n (%)	xx (xx.x%)
2+	n (%)	xx (xx.x%)
3+	n (%)	xx (xx.x%)
4+	n (%)	xx (xx.x%)
Follow-up		
Neg	n (%)	xx (xx.x%)
Trace	n (%)	xx (xx.x%)
1+	n (%)	xx (xx.x%)
2+	n (%)	xx (xx.x%)
3+	n (%)	xx (xx.x%)
4+	n (%)	xx (xx.x%)

Notes:
Percentages are calculated relative to the total number of patients of ARM C in the SAF set.

Source: XXXX.SAS, Run on DDMMMYYYY

TABLE 79. Urinalysis (categorical parameters – Part II) – ARM A

POPULATION: SAF **< Note for SAS Programmer: the following must be presented for URBC/HPF, Casts/LPF, Bacteria, Other as well >**

	Statistic	Arm A (N=XX)
UWBC/HPF		
Baseline 1		
Not present	n (%)	xx (xx.x%)
Present	n (%)	xx (xx.x%)
Week *<Every 4 wks>* – Combo Target		
Not present	n (%)	xx (xx.x%)
Present	n (%)	xx (xx.x%)
Baseline 2		
Not present	n (%)	xx (xx.x%)
Present	n (%)	xx (xx.x%)
Week *<Every 3 wks x 4 times>* – Nivolumab + Ipilimumab		
Not present	n (%)	xx (xx.x%)
Present	n (%)	xx (xx.x%)
Week *<Every 2 wks>* – Nivolumab		
Not present	n (%)	xx (xx.x%)
Present	n (%)	xx (xx.x%)
End of Treatment		
Not present	n (%)	xx (xx.x%)
Present	n (%)	xx (xx.x%)
Follow-up		
Not present	n (%)	xx (xx.x%)
Present	n (%)	xx (xx.x%)

Notes:
Percentages are calculated relative to the total number of patients of ARM A in the SAF set.

Source: XXXX.SAS, Run on DDMMMYYYY

TABLE 80. Urinalysis (categorical parameters – Part II) – ARM B

POPULATION: SAF **< Note for SAS Programmer: the following must be presented for URBC/HPF, Casts/LPF, Bacteria, Other as well >**

	Statistic	Arm B (N=XX)
UWBC/HPF		
Baseline 1		
Not present	n (%)	xx (xx.x%)
Present	n (%)	xx (xx.x%)
Week *<Every 3 wks x 4 times>* – Nivolumab + Ipilimumab		
Neg	n (%)	xx (xx.x%)
Trace	n (%)	xx (xx.x%)
Week *<Every 2 wks>* – Nivolumab		
Not present	n (%)	xx (xx.x%)
Present	n (%)	xx (xx.x%)
Baseline 2		
Not present	n (%)	xx (xx.x%)
Present	n (%)	xx (xx.x%)
Week *<Every 4 wks>* – Combo Target		
Not present	n (%)	xx (xx.x%)
Present	n (%)	xx (xx.x%)
End of Treatment		
Not present	n (%)	xx (xx.x%)
Present	n (%)	xx (xx.x%)
Follow-up		
Not present	n (%)	xx (xx.x%)
Present	n (%)	xx (xx.x%)

Notes:
Percentages are calculated relative to the total number of patients of ARM B in the SAF set.

Source: XXXX.SAS, Run on DDMMMYYYY

TABLE 81. Urinalysis (categorical parameters – Part II) – ARM C

POPULATION: SAF **< Note for SAS Programmer: the following must be presented for URBC/HPF, Casts/LPF, Bacteria, Other as well >**

	Statistic	Arm C (N=XX)
UWBC/HPF		
Baseline 1		
Not present	n (%)	xx (xx.x%)
Present	n (%)	xx (xx.x%)
Day 1 – Combo Target		
Not present	n (%)	xx (xx.x%)
Present	n (%)	xx (xx.x%)
Week 4 – Combo Target		
Not present	n (%)	xx (xx.x%)
Present	n (%)	xx (xx.x%)
Baseline 2		
Not present	n (%)	xx (xx.x%)
Present	n (%)	xx (xx.x%)
Week *<Every 3 wks x 4 times>* – Nivolumab + Ipilimumab		
Not present	n (%)	xx (xx.x%)
Present	n (%)	xx (xx.x%)
Week *<Every 2 wks>* – Nivolumab		
Not present	n (%)	xx (xx.x%)
Present	n (%)	xx (xx.x%)
Baseline 3		
Not present	n (%)	xx (xx.x%)
Present	n (%)	xx (xx.x%)
Week *<Every 4 wks>* – Combo Target		
Not present	n (%)	xx (xx.x%)
Present	n (%)	xx (xx.x%)
End of Treatment		
Not present	n (%)	xx (xx.x%)
Present	n (%)	xx (xx.x%)
Follow-up		
Not present	n (%)	xx (xx.x%)
Present	n (%)	xx (xx.x%)

Notes:
Percentages are calculated relative to the total number of patients of ARM C in the SAF set.

Source: XXXX.SAS, Run on DDMMMYYYY

TABLE 82. Urinalysis (continuous parameters) – ARM A

POPULATION: SAF **< Note for SAS Programmer: the following must be presented for continuous values of URBC/HPF, Casts/LPF, Bacteria, Other as well >**

	Statistic	Value	Change from Baseline (*)
UWBC/HPF			
Baseline 1	n	xx	
	Mean (SD)	xx.x (x.x)	
	Median	xx.x	
	Min/Max	xx/xx	
Week *<Every 4 wks>* – Combo Target	n	xx	xx
	Mean (SD)	xx.x (x.x)	xx.x (x.x)
	Median	xx.x	xx.x
	Min/Max	xx/xx	xx/xx
Baseline 2	n	xx	
	Mean (SD)	xx.x (x.x)	
	Median	xx.x	
	Min/Max	xx/xx	
Week *<Every 3 wks x 4 times>* – Nivolumab + Ipilimumab	n	xx	xx
	Mean (SD)	xx.x (x.x)	xx.x (x.x)
	Median	xx.x	xx.x
	Min/Max	xx/xx	xx/xx
Week *<Every 2 wks>* – Nivolumab	n	xx	xx
	Mean (SD)	xx.x (x.x)	xx.x (x.x)
	Median	xx.x	xx.x
	Min/Max	xx/xx	xx/xx
End of Treatment	n	xx	xx
	Mean (SD)	xx.x (x.x)	xx.x (x.x)
	Median	xx.x	xx.x
	Min/Max	xx/xx	xx/xx
Follow-up	n	xx	xx
	Mean (SD)	xx.x (x.x)	xx.x (x.x)
	Median	xx.x	xx.x
	Min/Max	xx/xx	xx/xx

Notes:

(*) Change from baseline is calculated relative to Baseline 1 for values between Baseline 1 and Baseline 2; Baseline 2 for values after Baseline 2. Change from baseline values include only those patients with both a Baseline value and a value for summarized time period.
n represents number of patients contributing to summary statistics.

Source: XXXX.SAS, Run on DDMMMYYYY

TABLE 83. Urinalysis (continuous parameters) - ARM B

POPULATION: SAF **< Note for SAS Programmer: the following must be presented for continuous values of URBC/HPF, Casts/LPF, Bacteria, Other as well >**

	Statistic	Value	Change from Baseline (*)
UWBC/HPF			
Baseline 1	n	xx	
	Mean (SD)	xx.x (x.x)	
	Median	xx.x	
	Min/Max	xx/xx	
Week *<Every 3 wks x 4 times>* – Nivolumab + Ipilimumab	n	xx	xx
	Mean (SD)	xx.x (x.x)	xx.x (x.x)
	Median	xx.x	xx.x
	Min/Max	xx/xx	xx/xx
Week *<Every 2 wks>* – Nivolumab	n	xx	xx
	Mean (SD)	xx.x (x.x)	xx.x (x.x)
	Median	xx.x	xx.x
	Min/Max	xx/xx	xx/xx
Baseline 2	n	xx	
	Mean (SD)	xx.x (x.x)	
	Median	xx.x	
	Min/Max	xx/xx	
Week *<Every 4 wks>* – Combo Target	n	xx	xx
	Mean (SD)	xx.x (x.x)	xx.x (x.x)
	Median	xx.x	xx.x
	Min/Max	xx/xx	xx/xx
End of Treatment	n	xx	xx
	Mean (SD)	xx.x (x.x)	xx.x (x.x)
	Median	xx.x	xx.x
	Min/Max	xx/xx	xx/xx
Follow-up	n	xx	xx
	Mean (SD)	xx.x (x.x)	xx.x (x.x)
	Median	xx.x	xx.x
	Min/Max	xx/xx	xx/xx

Notes:

(*) Change from baseline is calculated relative to Baseline 1 for values between Baseline 1 and Baseline 2; Baseline 2 for values after Baseline 2. Change from baseline values include only those patients with both a Baseline value and a value for summarized time period.
n represents number of patients contributing to summary statistics.

Source: XXXX.SAS, Run on DDMMMYYYY

TABLE 84. Urinalysis (continuous parameters) – ARM C

POPULATION: SAF **< Note for SAS Programmer: the following must be presented for continuous values of URBC/HPF, Casts/LPF, Bacteria, Other as well >**

	Statistic	Value	Change from Baseline (*)
UWBC/HPF			
Baseline 1	n	xx	
	Mean (SD)	xx.x (x.x)	
	Median	xx.x	
	Min/Max	xx/xx	
Day 1 – Combo Target	n	xx	xx
	Mean (SD)	xx.x (x.x)	xx.x (x.x)
	Median	xx.x	xx.x
	Min/Max	xx/xx	xx/xx
Week 4 – Combo Target	n	xx	xx
	Mean (SD)	xx.x (x.x)	xx.x (x.x)
	Median	xx.x	xx.x
	Min/Max	xx/xx	xx/xx
Baseline 2	n	xx	
	Mean (SD)	xx.x (x.x)	
	Median	xx.x	
	Min/Max	xx/xx	
Week *<Every 3 wks x 4 times>* – Nivolumab + Ipilimumab	n	xx	xx
	Mean (SD)	xx.x (x.x)	xx.x (x.x)
	Median	xx.x	xx.x
	Min/Max	xx/xx	xx/xx
Week *<Every 2 wks>* – Nivolumab	n	xx	xx
	Mean (SD)	xx.x (x.x)	xx.x (x.x)
	Median	xx.x	xx.x
	Min/Max	xx/xx	xx/xx
Baseline 3	n	xx	
	Mean (SD)	xx.x (x.x)	
	Median	xx.x	
	Min/Max	xx/xx	
Week *<Every 4 wks>* – Combo Target	n	xx	xx
	Mean (SD)	xx.x (x.x)	xx.x (x.x)
	Median	xx.x	xx.x

Min/Max	xx/xx	xx/xx

Notes:
(*) Change from baseline is calculated relative to Baseline 1 for values between Baseline 1 and Baseline 2; Baseline 2 for values between Baseline 2 and Baseline 3; Baseline 3 for values after Baseline 3. Change from baseline values include only those patients with both a Baseline value and a value for summarized time period.
n represents number of patients contributing to summary statistics.

Source: XXXX.SAS, Run on DDMMMYYYY

TABLE 84. Urinalysis (continuous parameters) – ARM C (cont.)

POPULATION: SAF **< Note for SAS Programmer: the following must be presented for continuous values of URBC/HPF, Casts/LPF, Bacteria, Other as well >**

	Statistic	Value	Change from Baseline (*)
UWBC/HPF			
End of Treatment	n	xx	xx
	Mean (SD)	xx.x (x.x)	xx.x (x.x)
	Median	xx.x	xx.x
	Min/Max	xx/xx	xx/xx
Follow-up	n	xx	xx
	Mean (SD)	xx.x (x.x)	xx.x (x.x)
	Median	xx.x	xx.x
	Min/Max	xx/xx	xx/xx

Notes:
(*) Change from baseline is calculated relative to Baseline 1 for values between Baseline 1 and Baseline 2; Baseline 2 for values between Baseline 2 and Baseline 3; Baseline 3 for values after Baseline 3. Change from baseline values include only those patients with both a Baseline value and a value for summarized time period.
n represents number of patients contributing to summary statistics.

Source: XXXX.SAS, Run on DDMMMYYYY

TABLE 85. ECG Parameters – ARM A

POPULATION: SAF **< Note for SAS Programmer: the following must be presented for QT, QRS, QTcB, cardfiac axis, Ejection Fraction value as well >**

	Statistic	Value	Change from Baseline (*)
PR interval (ms)			
Baseline 1	n	xx	
	Mean (SD)	xx.x (x.x)	
	Median	xx.x	
	Min/Max	xx/xx	
Week 4 – Combo Target	n	xx	xx
	Mean (SD)	xx.x (x.x)	xx.x (x.x)
	Median	xx.x	xx.x
	Min/Max	xx/xx	xx/xx
Week *<Every 12 wks>* – Combo Target	n	xx	xx
	Mean (SD)	xx.x (x.x)	xx.x (x.x)
	Median	xx.x	xx.x
	Min/Max	xx/xx	xx/xx
Baseline 2	n	xx	
	Mean (SD)	xx.x (x.x)	
	Median	xx.x	
	Min/Max	xx/xx	
Week *4* – Nivolumab + Ipilimumab	n	xx	xx
	Mean (SD)	xx.x (x.x)	xx.x (x.x)
	Median	xx.x	xx.x
	Min/Max	xx/xx	xx/xx
Week *<Every 12 wks>* – Nivolumab	n	xx	xx
	Mean (SD)	xx.x (x.x)	xx.x (x.x)
	Median	xx.x	xx.x
	Min/Max	xx/xx	xx/xx

Notes:
(*) Change from baseline is calculated relative to Baseline 1 for values between Baseline 1 and Baseline 2; Baseline 2 for values after Baseline 2. Change from baseline values include only those patients with both a Baseline value and a value for summarized time period.
n represents number of patients contributing to summary statistics.

Source: XXXX.SAS, Run on DDMMMYYYY

TABLE 86. ECG Parameters - ARM B

POPULATION: SAF **< Note for SAS Programmer: the following must be presented for QT, QRS, QTcB, cardfiac axis, Ejection Fraction value as well >**

	Statistic	Value	Change from Baseline (*)
PR interval (ms)			
Baseline 1	n	xx	
	Mean (SD)	xx.x (x.x)	
	Median	xx.x	
	Min/Max	xx/xx	
Week 4 – Nivolumab + Ipilimumab	n	xx	xx
	Mean (SD)	xx.x (x.x)	xx.x (x.x)
	Median	xx.x	xx.x
	Min/Max	xx/xx	xx/xx
Week *<Every 12 wks>* – Nivolumab	n	xx	xx
	Mean (SD)	xx.x (x.x)	xx.x (x.x)
	Median	xx.x	xx.x
	Min/Max	xx/xx	xx/xx
Baseline 2	n	xx	
	Mean (SD)	xx.x (x.x)	
	Median	xx.x	
	Min/Max	xx/xx	
Week 4 – Combo Target	n	xx	xx
	Mean (SD)	xx.x (x.x)	xx.x (x.x)
	Median	xx.x	xx.x
	Min/Max	xx/xx	xx/xx
Week *<Every 12 wks>* – Combo Target	n	xx	xx
	Mean (SD)	xx.x (x.x)	xx.x (x.x)
	Median	xx.x	xx.x
	Min/Max	xx/xx	xx/xx

Notes:
(*) Change from baseline is calculated relative to Baseline 1 for values between Baseline 1 and Baseline 2; Baseline 2 for values after Baseline 2. Change from baseline values include only those patients with both a Baseline value and a value for summarized time period.
n represents number of patients contributing to summary statistics.

Source: XXXX.SAS, Run on DDMMMYYYY

TABLE 87. ECG Parameters – ARM C

POPULATION: SAF **< Note for SAS Programmer: the following must be presented for continuous values of URBC/HPF, Bacteria, Other as well >**

	Statistic	Value	Change from Baseline (*)
PR interval (ms)			
Baseline 1	n	xx	
	Mean (SD)	xx.x (x.x)	
	Median	xx.x	
	Min/Max	xx/xx	
Week 4 – Combo Target	n	xx	xx
	Mean (SD)	xx.x (x.x)	xx.x (x.x)
	Median	xx.x	xx.x
	Min/Max	xx/xx	xx/xx
Baseline 2	n	xx	
	Mean (SD)	xx.x (x.x)	
	Median	xx.x	
	Min/Max	xx/xx	
Week 4 – Nivolumab + Ipilimumab	n	xx	xx
	Mean (SD)	xx.x (x.x)	xx.x (x.x)
	Median	xx.x	xx.x
	Min/Max	xx/xx	xx/xx
Week *<Every 12 wks>* – Nivolumab	n	xx	xx
	Mean (SD)	xx.x (x.x)	xx.x (x.x)
	Median	xx.x	xx.x
	Min/Max	xx/xx	xx/xx
Baseline 3	n	xx	
	Mean (SD)	xx.x (x.x)	
	Median	xx.x	
	Min/Max	xx/xx	
Week 4 – Combo Target	n	xx	xx
	Mean (SD)	xx.x (x.x)	xx.x (x.x)
	Median	xx.x	xx.x
	Min/Max	xx/xx	xx/xx
Week *<Every 12 wks>* – Combo Target	n	xx	xx
	Mean (SD)	xx.x (x.x)	xx.x (x.x)
	Median	xx.x	xx.x
	Min/Max	xx/xx	xx/xx

Notes:
(*) Change from baseline is calculated relative to Baseline 1 for values between Baseline 1 and Baseline 2; Baseline 2 for values between Baseline 2 and Baseline 3; Baseline 3 for values after Baseline 3. Change from baseline values include only those patients with both a Baseline value and a value for summarized time period.
n represents number of patients contributing to summary statistics.

Source: XXXX.SAS, Run on DDMMMYYYY

TABLE 88. Cardiological Assessments – ARM A

POPULATION: SAF **< Note for SAS Programmer: the following must be presented for each visit during the study >**

	Statistic	Arm A (N=XX)
<Visit n>		
ECG Result		
Normal	n (%)	xx (xx.x%)
Abnormal	n (%)	xx (xx.x%)
Interpretation of abnormality (*)		
Not clinically significant	n (%)	xx (xx.x%)
Clinically significant	n (%)	xx (xx.x%)
Type of LVEF scan		
ECHO	n (%)	xx (xx.x%)
MUGA	n (%)	xx (xx.x%)
Interpretation of ECHO/MUGA scan		
Normal	n (%)	xx (xx.x%)
Abnormal, NCS	n (%)	xx (xx.x%)
Normal, CS	n (%)	xx (xx.x%)

Notes:
Percentages are calculated relative to the total number of patients of ARM A in the SAF set.
(*) Percentages are calculated relative to the total number of patients of ARM A in the SAF set with abnormal ECG.
NCS = Not Clinically Significant; CS = Clinically Significant.

Source: XXXX.SAS, Run on DDMMMYYYY

TABLE 89. Cardiological Assessments – ARM B

POPULATION: SAF **< Note for SAS Programmer: the following must be presented for each visit during the study >**

	Statistic	Arm B (N=XX)
<Visit n>		
ECG Result		
Normal	n (%)	xx (xx.x%)
Abnormal	n (%)	xx (xx.x%)
Interpretation of abnormality (*)		
Not clinically significant	n (%)	xx (xx.x%)
Clinically significant	n (%)	xx (xx.x%)
Type of LVEF scan		
ECHO	n (%)	xx (xx.x%)
MUGA	n (%)	xx (xx.x%)
Interpretation of ECHO/MUGA scan		
Normal	n (%)	xx (xx.x%)
Abnormal, NCS	n (%)	xx (xx.x%)
Normal, CS	n (%)	xx (xx.x%)

Notes:
Percentages are calculated relative to the total number of patients of ARM B in the SAF set.
(*) Percentages are calculated relative to the total number of patients of ARM B in the SAF set with abnormal ECG.
NCS = Not Clinically Significant; CS = Clinically Significant.

Source: XXXX.SAS, Run on DDMMMYYYY

TABLE 90. Cardiological Assessments – ARM C

POPULATION: SAF **< Note for SAS Programmer: the following must be presented for each visit during the study >**

	Statistic	Arm C (N=XX)
<Visit n>		
ECG Result		
Normal	n (%)	xx (xx.x%)
Abnormal	n (%)	xx (xx.x%)
Interpretation of abnormality (*)		
Not clinically significant	n (%)	xx (xx.x%)
Clinically significant	n (%)	xx (xx.x%)
Type of LVEF scan		
ECHO	n (%)	xx (xx.x%)
MUGA	n (%)	xx (xx.x%)
Interpretation of ECHO/MUGA scan		
Normal	n (%)	xx (xx.x%)
Abnormal, NCS	n (%)	xx (xx.x%)
Normal, CS	n (%)	xx (xx.x%)

Notes:
Percentages are calculated relative to the total number of patients of ARM C in the SAF set.
(*) Percentages are calculated relative to the total number of patients of ARM C in the SAF set with abnormal ECG.
NCS = Not Clinically Significant; CS = Clinically Significant.

Source: XXXX.SAS, Run on DDMMMYYYY

TABLE 91. Vital signs – ARM A

POPULATION: SAF **< Note for SAS Programmer: the following must be presented for each vital sign >**

	Statistic	Value	Change from Baseline (*)
Body weight (Kg)			
Baseline 1	n	xx	
	Mean (SD)	xx.x (x.x)	
	Median	xx.x	
	Min/Max	xx/xx	
Week *<Every 4 wks>* – Combo Target	n	xx	xx
	Mean (SD)	xx.x (x.x)	xx.x (x.x)
	Median	xx.x	xx.x
	Min/Max	xx/xx	xx/xx
Baseline 2	n	xx	
	Mean (SD)	xx.x (x.x)	
	Median	xx.x	
	Min/Max	xx/xx	
Week *<Every 3 wks x 4 times>* – Nivolumab + Ipilimumab	n	xx	xx
	Mean (SD)	xx.x (x.x)	xx.x (x.x)
	Median	xx.x	xx.x
	Min/Max	xx/xx	xx/xx
Week *<Every 2 wks>* – Nivolumab	n	xx	xx
	Mean (SD)	xx.x (x.x)	xx.x (x.x)
	Median	xx.x	xx.x
	Min/Max	xx/xx	xx/xx
End of Treatment	n	xx	xx
	Mean (SD)	xx.x (x.x)	xx.x (x.x)
	Median	xx.x	xx.x
	Min/Max	xx/xx	xx/xx
Follow-up	n	xx	xx
	Mean (SD)	xx.x (x.x)	xx.x (x.x)
	Median	xx.x	xx.x
	Min/Max	xx/xx	xx/xx

Notes:
(*) Change from baseline is calculated relative to Baseline 1 for values between Baseline 1 and Baseline 2; Baseline 2 for values after Baseline 2. Change from baseline values include only those patients with both a Baseline value and a value for summarized time period.

n represents number of patients contributing to summary statistics.

Source: XXXX.SAS, Run on DDMMMYYYY

TABLE 92. Vital signs – ARM B

POPULATION: SAF **< Note for SAS Programmer: the following must be presented for each vital sign >**

	Statistic	Value	Change from Baseline (*)
Body weight (Kg)			
Baseline 1	n	xx	
	Mean (SD)	xx.x (x.x)	
	Median	xx.x	
	Min/Max	xx/xx	
Week *<Every 3 wks x 4 times>* – Nivolumab + Ipilimumab	n	xx	xx
	Mean (SD)	xx.x (x.x)	xx.x (x.x)
	Median	xx.x	xx.x
	Min/Max	xx/xx	xx/xx
Week *<Every 2 wks>* – Nivolumab	n	xx	xx
	Mean (SD)	xx.x (x.x)	xx.x (x.x)
	Median	xx.x	xx.x
	Min/Max	xx/xx	xx/xx
Baseline 2	n	xx	
	Mean (SD)	xx.x (x.x)	
	Median	xx.x	
	Min/Max	xx/xx	
Week *<Every 4 wks>* – Combo Target	n	xx	xx
	Mean (SD)	xx.x (x.x)	xx.x (x.x)
	Median	xx.x	xx.x
	Min/Max	xx/xx	xx/xx
End of Treatment	n	xx	xx
	Mean (SD)	xx.x (x.x)	xx.x (x.x)
	Median	xx.x	xx.x
	Min/Max	xx/xx	xx/xx
Follow-up	n	xx	xx
	Mean (SD)	xx.x (x.x)	xx.x (x.x)
	Median	xx.x	xx.x
	Min/Max	xx/xx	xx/xx

Notes:
(*) Change from baseline is calculated relative to Baseline 1 for values between Baseline 1 and Baseline 2; Baseline 2 for values after Baseline 2. Change from baseline values include only those patients with both a Baseline value and a value for summarized time period.

n represents number of patients contributing to summary statistics.

Source: XXXX.SAS, Run on DDMMMYYYY

TABLE 93. Vital signs – ARM C

POPULATION: SAF **< Note for SAS Programmer: the following must be presented for each vital sign >**

	Statistic	Value	Change from Baseline (*)
Body weight (Kg)			
Baseline 1	n	xx	
	Mean (SD)	xx.x (x.x)	
	Median	xx.x	
	Min/Max	xx/xx	
Day 1 – Combo Target	n	xx	xx
	Mean (SD)	xx.x (x.x)	xx.x (x.x)
	Median	xx.x	xx.x
	Min/Max	xx/xx	xx/xx
Week 4 – Combo Target	n	xx	xx
	Mean (SD)	xx.x (x.x)	xx.x (x.x)
	Median	xx.x	xx.x
	Min/Max	xx/xx	xx/xx
Baseline 2	n	xx	
	Mean (SD)	xx.x (x.x)	
	Median	xx.x	
	Min/Max	xx/xx	
Week *<Every 3 wks x 4 times>* – Nivolumab + Ipilimumab	n	xx	xx
	Mean (SD)	xx.x (x.x)	xx.x (x.x)
	Median	xx.x	xx.x
	Min/Max	xx/xx	xx/xx
Week *<Every 2 wks>* – Nivolumab	n	xx	xx
	Mean (SD)	xx.x (x.x)	xx.x (x.x)
	Median	xx.x	xx.x
	Min/Max	xx/xx	xx/xx
Baseline 3	n	xx	
	Mean (SD)	xx.x (x.x)	
	Median	xx.x	
	Min/Max	xx/xx	
Week *<Every 4 wks>* – Combo Target	n	xx	xx
	Mean (SD)	xx.x (x.x)	xx.x (x.x)
	Median	xx.x	xx.x
	Min/Max	xx/xx	xx/xx

Notes:
(*) Change from baseline is calculated relative to Baseline 1 for values between Baseline 1 and Baseline 2; Baseline 2 for values between Baseline 2 and Baseline 3; Baseline 3 for values after Baseline 3. Change from baseline values include only those patients with both a Baseline value and a value for summarized time period.
n represents number of patients contributing to summary statistics.

Source: XXXX.SAS, Run on DDMMMYYYY

TABLE 93. Vital signs – ARM C (cont.)

POPULATION: SAF **< Note for SAS Programmer: the following must be presented for each vital sign >**

	Statistic	Value	Change from Baseline (*)
Body weight (Kg)			
End of Treatment	n	xx	xx
	Mean (SD)	xx.x (x.x)	xx.x (x.x)
	Median	xx.x	xx.x
	Min/Max	xx/xx	xx/xx
Follow-up	n	xx	xx
	Mean (SD)	xx.x (x.x)	xx.x (x.x)
	Median	xx.x	xx.x
	Min/Max	xx/xx	xx/xx

Notes:
(*) Change from baseline is calculated relative to Baseline 1 for values between Baseline 1 and Baseline 2; Baseline 2 for values between Baseline 2 and Baseline 3; Baseline 3 for values after Baseline 3. Change from baseline values include only those patients with both a Baseline value and a value for summarized time period.
n represents number of patients contributing to summary statistics.

Source: XXXX.SAS, Run on DDMMMYYYY

TABLE 94. Ophthalmological Examinations during the study

POPULATION: SAF

	Statistic	Arm A (N=XX)	Arm B (N=XX)	Arm C (N=XX)
Baseline 1				
Ophthalmological examination performed?				
No	n (%)	xx (xx.x%)	xx (xx.x%)	xx (xx.x%)
Yes	n (%)	xx (xx.x%)	xx (xx.x%)	xx (xx.x%)
Not Applicable	n (%)	xx (xx.x%)	xx (xx.x%)	xx (xx.x%)
Eye(s) assessed (*)				
Left Eye	n (%)	xx (xx.x%)	xx (xx.x%)	xx (xx.x%)
Right Eye	n (%)	xx (xx.x%)	xx (xx.x%)	xx (xx.x%)
Both	n (%)	xx (xx.x%)	xx (xx.x%)	xx (xx.x%)
Baseline 2				
Ophthalmological examination performed?				
No	n (%)	xx (xx.x%)	xx (xx.x%)	xx (xx.x%)
Yes	n (%)	xx (xx.x%)	xx (xx.x%)	xx (xx.x%)
Not Applicable	n (%)	xx (xx.x%)	xx (xx.x%)	xx (xx.x%)
Eye(s) assessed (*)				
Left Eye	n (%)	xx (xx.x%)	xx (xx.x%)	xx (xx.x%)
Right Eye	n (%)	xx (xx.x%)	xx (xx.x%)	xx (xx.x%)
Both	n (%)	xx (xx.x%)	xx (xx.x%)	xx (xx.x%)
Baseline 3				
Ophthalmological examination performed?				
No	n (%)	xx (xx.x%)	xx (xx.x%)	xx (xx.x%)
Yes	n (%)	xx (xx.x%)	xx (xx.x%)	xx (xx.x%)
Not Applicable	n (%)	xx (xx.x%)	xx (xx.x%)	xx (xx.x%)
Eye(s) assessed (*)				
Left Eye	n (%)	xx (xx.x%)	xx (xx.x%)	xx (xx.x%)
Right Eye	n (%)	xx (xx.x%)	xx (xx.x%)	xx (xx.x%)
Both	n (%)	xx (xx.x%)	xx (xx.x%)	xx (xx.x%)

Notes:
Percentages are calculated relative to the total number of patients in the SAF set by treatment arm.
(*) Percentages are calculated relative to the total number of patients in the SAF set who performed the ophthalmological examination by treatment arm.
Baseline 3 is applicable to Arm C only.

Source: XXXX.SAS, Run on DDMMMYYYY

TABLE 95. Visual Acuity during the study

POPULATION: SAF

	Statistic	Arm A (N=XX)	Arm B (N=XX)	Arm C (N=XX)
Baseline 1				
Visual Acuity assessed (*)				
No	n (%)	xx (xx.x%)	x (xx.x%)	x (xx.x%)
Yes	n (%)	xx (xx.x%)	x (xx.x%)	x (xx.x%)
Total Visual Acuity Score (ETDRS) - Left Eye (OS)	n	xx	xx	xx
	Mean (SD)	xx.x (x.x)	xx.x (x.x)	xx.x (x.x)
	Median	xx.x	xx.x	xx.x
	Min/Max	xx/xx	xx/xx	xx/xx
Total Visual Acuity Score (ETDRS) - Right Eye (OD)	n	xx	xx	xx
	Mean (SD)	xx.x (x.x)	xx.x (x.x)	xx.x (x.x)
	Median	xx.x	xx.x	xx.x
	Min/Max	xx/xx	xx/xx	xx/xx
Total Visual Acuity snelles equivalent - Left Eye (OS)	n	xx	xx	xx
	Mean (SD)	xx.x (x.x)	xx.x (x.x)	xx.x (x.x)
	Median	xx.x	xx.x	xx.x
	Min/Max	xx/xx	xx/xx	xx/xx
Total Visual Acuity snelles equivalent - Right Eye (OD)	n	xx	xx	xx
	Mean (SD)	xx.x (x.x)	xx.x (x.x)	xx.x (x.x)
	Median	xx.x	xx.x	xx.x
	Min/Max	xx/xx	xx/xx	xx/xx
Baseline 2				
Visual Acuity assessed (*)				
No	n (%)	xx (xx.x%)	x (xx.x%)	x (xx.x%)
Yes	n (%)	xx (xx.x%)	x (xx.x%)	x (xx.x%)
Total Visual Acuity Score (ETDRS) - Left Eye (OS)	n	xx	xx	xx
	Mean (SD)	xx.x (x.x)	xx.x (x.x)	xx.x (x.x)
	Median	xx.x	xx.x	xx.x
	Min/Max	xx/xx	xx/xx	xx/xx
Total Visual Acuity Score (ETDRS) - Right Eye (OD)	n	xx	xx	xx
	Mean (SD)	xx.x (x.x)	xx.x (x.x)	xx.x (x.x)
	Median	xx.x	xx.x	xx.x
	Min/Max	xx/xx	xx/xx	xx/xx
Total Visual Acuity snelles equivalent - Left Eye (OS)	n	xx	xx	xx
	Mean (SD)	xx.x (x.x)	xx.x (x.x)	xx.x (x.x)
	Median	xx.x	xx.x	xx.x
	Min/Max	xx/xx	xx/xx	xx/xx

Total Visual Acuity snelles equivalent - Right Eye (OD)	n	xx	xx	xx
	Mean (SD)	xx.x (x.x)	xx.x (x.x)	xx.x (x.x)
	Median	xx.x	xx.x	xx.x
	Min/Max	xx/xx	xx/xx	xx/xx

Notes:
(*) Percentages are calculated relative to the total number of patients in the SAF set who performed the ophthalmological examination by treatment arm.
Baseline 3 is applicable to Arm C only.

Source: XXXX.SAS, Run on DDMMMYYYY

Table 95. Visual Acuity during the study (cont.)

POPULATION: SAF

	Statistic	Arm A (N=XX)	Arm B (N=XX)	Arm C (N=XX)
Baseline 3				
Visual Acuity assessed (*)				
No	n (%)	xx (xx.x%)	x (xx.x%)	x (xx.x%)
Yes	n (%)	xx (xx.x%)	x (xx.x%)	x (xx.x%)
Total Visual Acuity Score (ETDRS) – Left Eye (OS)	n	xx	xx	xx
	Mean (SD)	xx.x (x.x)	xx.x (x.x)	xx.x (x.x)
	Median	xx.x	xx.x	xx.x
	Min/Max	xx/xx	xx/xx	xx/xx
Total Visual Acuity Score (ETDRS) – Right Eye (OD)	n	xx	xx	xx
	Mean (SD)	xx.x (x.x)	xx.x (x.x)	xx.x (x.x)
	Median	xx.x	xx.x	xx.x
	Min/Max	xx/xx	xx/xx	xx/xx
Total Visual Acuity snelles equivalent – Left Eye (OS)	n	xx	xx	xx
	Mean (SD)	xx.x (x.x)	xx.x (x.x)	xx.x (x.x)
	Median	xx.x	xx.x	xx.x
	Min/Max	xx/xx	xx/xx	xx/xx
Total Visual Acuity snelles equivalent – Right Eye (OD)	n	xx	xx	xx
	Mean (SD)	xx.x (x.x)	xx.x (x.x)	xx.x (x.x)
	Median	xx.x	xx.x	xx.x
	Min/Max	xx/xx	xx/xx	xx/xx

Notes:
(*) Percentages are calculated relative to the total number of patients in the SAF set who performed the ophthalmological examination by treatment arm.
Baseline 3 is applicable to Arm C only.

Source: XXXX.SAS, Run on DDMMMYYYY

TABLE 96. Intraocular pressure during the study

POPULATION: SAF

	Statistic	Arm A (N=XX)	Arm B (N=XX)	Arm C (N=XX)
Baseline 1				
Intraocular Pressure Assessed (*)				
No	n (%)	xx (xx.x%)	x (xx.x%)	x (xx.x%)
Yes	n (%)	xx (xx.x%)	x (xx.x%)	x (xx.x%)
Intraocular pressure - Left Eye (OS) (mmHg)	n	xx	xx	xx
	Mean (SD)	xx.x (x.x)	xx.x (x.x)	xx.x (x.x)
	Median	xx.x	xx.x	xx.x
	Min/Max	xx/xx	xx/xx	xx/xx
Intraocular pressure - Right Eye (OD) (mmHg)	n	xx	xx	xx
	Mean (SD)	xx.x (x.x)	xx.x (x.x)	xx.x (x.x)
	Median	xx.x	xx.x	xx.x
	Min/Max	xx/xx	xx/xx	xx/xx
Baseline 2				
Intraocular Pressure Assessed (*)				
No	n (%)	xx (xx.x%)	x (xx.x%)	x (xx.x%)
Yes	n (%)	xx (xx.x%)	x (xx.x%)	x (xx.x%)
Intraocular pressure - Left Eye (OS) (mmHg)	n	xx	xx	xx
	Mean (SD)	xx.x (x.x)	xx.x (x.x)	xx.x (x.x)
	Median	xx.x	xx.x	xx.x
	Min/Max	xx/xx	xx/xx	xx/xx
Intraocular pressure - Right Eye (OD) (mmHg)	n	xx	xx	xx
	Mean (SD)	xx.x (x.x)	xx.x (x.x)	xx.x (x.x)
	Median	xx.x	xx.x	xx.x
	Min/Max	xx/xx	xx/xx	xx/xx
Baseline 3				
Intraocular Pressure Assessed (*)				
No	n (%)	xx (xx.x%)	x (xx.x%)	x (xx.x%)
Yes	n (%)	xx (xx.x%)	x (xx.x%)	x (xx.x%)
Intraocular pressure - Left Eye (OS) (mmHg)	n	xx	xx	xx
	Mean (SD)	xx.x (x.x)	xx.x (x.x)	xx.x (x.x)
	Median	xx.x	xx.x	xx.x
	Min/Max	xx/xx	xx/xx	xx/xx
Intraocular pressure - Right Eye (OD) (mmHg)	n	xx	xx	xx
	Mean (SD)	xx.x (x.x)	xx.x (x.x)	xx.x (x.x)
	Median	xx.x	xx.x	xx.x
	Min/Max	xx/xx	xx/xx	xx/xx

Notes:
(*) Percentages are calculated relative to the total number of patients in the SAF set who performed the ophthalmological examination by treatment arm.
Baseline 3 is applicable to Arm C only.

Source: XXXX.SAS, Run on DDMMMYYYY

TABLE 97. Dilated fundoscopy during the study

POPULATION: SAF

	Statistic	Arm A (N=XX)	Arm B (N=XX)	Arm C (N=XX)
Baseline <N>				
Dilated fundoscopy Assessed (*)				
No	n (%)	xx (xx.x%)	x (xx.x%)	x (xx.x%)
Yes	n (%)	xx (xx.x%)	x (xx.x%)	x (xx.x%)
Eye(s) assessed (#)				
Left Eye	n (%)	xx (xx.x%)	x (xx.x%)	x (xx.x%)
Right Eye	n (%)	xx (xx.x%)	x (xx.x%)	x (xx.x%)
Both	n (%)	xx (xx.x%)	x (xx.x%)	x (xx.x%)
Right Eye assessment (§)				
Viterous				
Normal	n (%)	xx (xx.x%)	x (xx.x%)	x (xx.x%)
Abnormal	n (%)	xx (xx.x%)	x (xx.x%)	x (xx.x%)
Retina				
Normal	n (%)	xx (xx.x%)	x (xx.x%)	x (xx.x%)
Abnormal	n (%)	xx (xx.x%)	x (xx.x%)	x (xx.x%)
Macula				
Normal	n (%)	xx (xx.x%)	x (xx.x%)	x (xx.x%)
Abnormal	n (%)	xx (xx.x%)	x (xx.x%)	x (xx.x%)
Choroid				
Normal	n (%)	xx (xx.x%)	x (xx.x%)	x (xx.x%)
Abnormal	n (%)	xx (xx.x%)	x (xx.x%)	x (xx.x%)
Optic nerve pallor				
Normal	n (%)	xx (xx.x%)	x (xx.x%)	x (xx.x%)
Abnormal	n (%)	xx (xx.x%)	x (xx.x%)	x (xx.x%)
Other				
Normal	n (%)	xx (xx.x%)	x (xx.x%)	x (xx.x%)
Abnormal	n (%)	xx (xx.x%)	x (xx.x%)	x (xx.x%)

Notes:
(*) Percentages are calculated relative to the total number of patients in the SAF set who performed the ophthalmological examination by treatment arm.
(#) Percentages are calculated relative to the total number of patients in the SAF set for whom dilated fundoscopy was assessed by treatment arm.
(§) Percentages are calculated relative to the total number of patients in the SAF set for whom assessment was done on right eye by treatment arm.
(^) Percentages are calculated relative to the total number of patients in the SAF set for whom assessment was done on left eye by treatment arm.

Baseline 3 is applicable to Arm C only.

Source: XXXX.SAS, Run on DDMMMYYYY

< Note for SAS Programmer: in this table data from all baseline visits must be reported >

Table 97. Dilated fundoscopy during the study (cont.)
POPULATION: SAF

	Statistic	Arm A (N=XX)	Arm B (N=XX)	Arm C (N=XX)
Baseline <N>				
Left Eye assessment (^)				
Viterous				
Normal	n (%)	xx (xx.x%)	x (xx.x%)	x (xx.x%)
Abnormal	n (%)	xx (xx.x%)	x (xx.x%)	x (xx.x%)
Retina				
Normal	n (%)	xx (xx.x%)	x (xx.x%)	x (xx.x%)
Abnormal	n (%)	xx (xx.x%)	x (xx.x%)	x (xx.x%)
Macula				
Normal	n (%)	xx (xx.x%)	x (xx.x%)	x (xx.x%)
Abnormal	n (%)	xx (xx.x%)	x (xx.x%)	x (xx.x%)
Choroid				
Normal	n (%)	xx (xx.x%)	x (xx.x%)	x (xx.x%)
Abnormal	n (%)	xx (xx.x%)	x (xx.x%)	x (xx.x%)
Optic nerve pallor				
Normal	n (%)	xx (xx.x%)	x (xx.x%)	x (xx.x%)
Abnormal	n (%)	xx (xx.x%)	x (xx.x%)	x (xx.x%)
Other				
Normal	n (%)	xx (xx.x%)	x (xx.x%)	x (xx.x%)
Abnormal	n (%)	xx (xx.x%)	x (xx.x%)	x (xx.x%)

Notes:
(*) Percentages are calculated relative to the total number of patients in the SAF set who performed the ophthalmological examination by treatment arm.
(#) Percentages are calculated relative to the total number of patients in the SAF set for whom dilated fundoscopy was assessed by treatment arm.
(§) Percentages are calculated relative to the total number of patients in the SAF set for whom assessment was done on right eye by treatment arm.
(^) Percentages are calculated relative to the total number of patients in the SAF set for whom assessment was done on left eye by treatment arm.
Baseline 3 is applicable to Arm C only.

Source: XXXX.SAS, Run on DDMMMYYYY

< Note for SAS Programmer: in this table data from all baseline visits must be reported >

TABLE 98. Slit lamp examination during the study
POPULATION: SAF

	Statistic	Arm A (N=XX)	Arm B (N=XX)	Arm C (N=XX)
Baseline <N>				
Slit lamp examination performed (*)				
No	n (%)	xx (xx.x%)	x (xx.x%)	x (xx.x%)
Yes	n (%)	xx (xx.x%)	x (xx.x%)	x (xx.x%)
Eye(s) assessed (#)				
Left Eye	n (%)	xx (xx.x%)	x (xx.x%)	x (xx.x%)
Right Eye	n (%)	xx (xx.x%)	x (xx.x%)	x (xx.x%)
Both	n (%)	xx (xx.x%)	x (xx.x%)	x (xx.x%)
Right Eye assessment (§)				
Lids/lashes				
Normal	n (%)	xx (xx.x%)	x (xx.x%)	x (xx.x%)
Abnormal	n (%)	xx (xx.x%)	x (xx.x%)	x (xx.x%)
Cornea				
Normal	n (%)	xx (xx.x%)	x (xx.x%)	x (xx.x%)
Abnormal	n (%)	xx (xx.x%)	x (xx.x%)	x (xx.x%)
Conjunctiva				
Normal	n (%)	xx (xx.x%)	x (xx.x%)	x (xx.x%)
Abnormal	n (%)	xx (xx.x%)	x (xx.x%)	x (xx.x%)
Iris				
Normal	n (%)	xx (xx.x%)	x (xx.x%)	x (xx.x%)
Abnormal	n (%)	xx (xx.x%)	x (xx.x%)	x (xx.x%)
Lens				
Normal	n (%)	xx (xx.x%)	x (xx.x%)	x (xx.x%)
Abnormal	n (%)	xx (xx.x%)	x (xx.x%)	x (xx.x%)
Anterior chamber				
Normal	n (%)	xx (xx.x%)	x (xx.x%)	x (xx.x%)
Abnormal	n (%)	xx (xx.x%)	x (xx.x%)	x (xx.x%)
Other				
Normal	n (%)	xx (xx.x%)	x (xx.x%)	x (xx.x%)
Abnormal	n (%)	xx (xx.x%)	x (xx.x%)	x (xx.x%)

Notes:
(*) Percentages are calculated relative to the total number of patients in the SAF set who performed the ophthalmological examination by treatment arm.
(#) Percentages are calculated relative to the total number of patients in the SAF set for whom dilated fundoscopy was assessed by treatment arm.

(§) Percentages are calculated relative to the total number of patients in the SAF set for whom assessment was done on right eye by treatment arm.
(^) Percentages are calculated relative to the total number of patients in the SAF set for whom assessment was done on left eye by treatment arm.
Baseline 3 is applicable to Arm C only.

Source: XXXX.SAS, Run on DDMMMYYYY

< Note for SAS Programmer: in this table data from all baseline visits must be reported >

Table 98. Slit lamp examination during the study (cont.)
POPULATION: SAF

	Statistic	Arm A (N=XX)	Arm B (N=XX)	Arm C (N=XX)
Baseline <N>				
Left Eye assessment (^)				
Lids/lashes				
Normal	n (%)	xx (xx.x%)	x (xx.x%)	x (xx.x%)
Abnormal	n (%)	xx (xx.x%)	x (xx.x%)	x (xx.x%)
Cornea				
Normal	n (%)	xx (xx.x%)	x (xx.x%)	x (xx.x%)
Abnormal	n (%)	xx (xx.x%)	x (xx.x%)	x (xx.x%)
Conjunctiva				
Normal	n (%)	xx (xx.x%)	x (xx.x%)	x (xx.x%)
Abnormal	n (%)	xx (xx.x%)	x (xx.x%)	x (xx.x%)
Iris				
Normal	n (%)	xx (xx.x%)	x (xx.x%)	x (xx.x%)
Abnormal	n (%)	xx (xx.x%)	x (xx.x%)	x (xx.x%)
Lens				
Normal	n (%)	xx (xx.x%)	x (xx.x%)	x (xx.x%)
Abnormal	n (%)	xx (xx.x%)	x (xx.x%)	x (xx.x%)
Anterior chamber				
Normal	n (%)	xx (xx.x%)	x (xx.x%)	x (xx.x%)
Abnormal	n (%)	xx (xx.x%)	x (xx.x%)	x (xx.x%)
Other				
Normal	n (%)	xx (xx.x%)	x (xx.x%)	x (xx.x%)
Abnormal	n (%)	xx (xx.x%)	x (xx.x%)	x (xx.x%)

Notes:
(*) Percentages are calculated relative to the total number of patients in the SAF set who performed the ophthalmological examination by treatment arm.
(#) Percentages are calculated relative to the total number of patients in the SAF set for whom slit lamp examination was performed by treatment arm.
(§) Percentages are calculated relative to the total number of patients in the SAF set for whom esamination was done on right eye by treatment arm.
(^) Percentages are calculated relative to the total number of patients in the SAF set for whom esamination was done on left eye by treatment arm.
Baseline 3 is applicable to Arm C only.

Source: XXXX.SAS, Run on DDMMMYYYY

< Note for SAS Programmer: in this table data from all baseline visits must be reported >

TABLE 99. Visual field testing and optical coherence tomography during the study
POPULATION: SAF

	Statistic	Arm A (N=XX)	Arm B (N=XX)	Arm C (N=XX)
Baseline 1				
Visual field testing performed (*)				
No	n (%)	xx (xx.x%)	x (xx.x%)	x (xx.x%)
Yes	n (%)	xx (xx.x%)	x (xx.x%)	x (xx.x%)
Abnormalities? (§)				
No	n (%)	xx (xx.x%)	x (xx.x%)	x (xx.x%)
Yes	n (%)	xx (xx.x%)	x (xx.x%)	x (xx.x%)
Optical coherence tomography performed (*)				
No	n (%)	xx (xx.x%)	x (xx.x%)	x (xx.x%)
Yes	n (%)	xx (xx.x%)	x (xx.x%)	x (xx.x%)
Abnormalities? (#)				
No	n (%)	xx (xx.x%)	x (xx.x%)	x (xx.x%)
Yes	n (%)	xx (xx.x%)	x (xx.x%)	x (xx.x%)
Baseline 2				
Visual field testing performed (*)				
No	n (%)	xx (xx.x%)	x (xx.x%)	x (xx.x%)
Yes	n (%)	xx (xx.x%)	x (xx.x%)	x (xx.x%)
Abnormalities? (§)				
No	n (%)	xx (xx.x%)	x (xx.x%)	x (xx.x%)
Yes	n (%)	xx (xx.x%)	x (xx.x%)	x (xx.x%)
Optical coherence tomography performed (*)				
No	n (%)	xx (xx.x%)	x (xx.x%)	x (xx.x%)
Yes	n (%)	xx (xx.x%)	x (xx.x%)	x (xx.x%)
Abnormalities? (#)				
No	n (%)	xx (xx.x%)	x (xx.x%)	x (xx.x%)
Yes	n (%)	xx (xx.x%)	x (xx.x%)	x (xx.x%)
Baseline 3				
Visual field testing performed (*)				
No	n (%)	xx (xx.x%)	x (xx.x%)	x (xx.x%)
Yes	n (%)	xx (xx.x%)	x (xx.x%)	x (xx.x%)
Abnormalities? (§)				
No	n (%)	xx (xx.x%)	x (xx.x%)	x (xx.x%)
Yes	n (%)	xx (xx.x%)	x (xx.x%)	x (xx.x%)
Optical coherence tomography performed (*)				
No	n (%)	xx (xx.x%)	x (xx.x%)	x (xx.x%)
Yes	n (%)	xx (xx.x%)	x (xx.x%)	x (xx.x%)

Abnormalities? (#)				
No	n (%)	xx (xx.x%)	x (xx.x%)	x (xx.x%)
Yes	n (%)	xx (xx.x%)	x (xx.x%)	x (xx.x%)

Notes:
(*) Percentages are calculated relative to the total number of patients in the SAF set who performed the ophthalmological examination by treatment arm.
(§) Percentages are calculated relative to the total number of patients in the SAF set who performed the visual field testing by treatment arm.
(#) Percentages are calculated relative to the total number of patients in the SAF set who performed the optical coherence tomography by treatment arm.
Baseline 3 is applicable to Arm C only.
Source: XXXX.SAS, Run on DDMMMYYYY

TABLE 100. Dermatological examination during the study – ARM A

POPULATION: SAF **< Note for SAS Programmer: the following must be presented for each visit: Baseline 1, Q8W, Baseline 2, Q8W >**

	Statistic	Arm A (N=XX)
<Visit n>		
Dermatological Examination performed		
No	n (%)	xx (xx.x%)
Yes	n (%)	xx (xx.x%)
Result (*)		
Normal	n (%)	xx (xx.x%)
Abnormal	n (%)	xx (xx.x%)
Diagnosis (§)		
Squamous cell carcinoma	n (%)	xx (xx.x%)
Keratoacanthomas	n (%)	xx (xx.x%)
Other pathology findings	n (%)	xx (xx.x%)
Evidence of severe or uncontrolled systemic disease or concurrent undesirable condition		
No	n (%)	xx (xx.x%)
Yes	n (%)	xx (xx.x%)

Notes:
Percentages are calculated relative to the total number of patients of ARM A in the SAF set.
(*) Percentages are calculated relative to the total number of patients of ARM A in the SAF set who performed the dermatological examination.
(§) Percentages are calculated relative to the total number of patients of ARM A in the SAF set who resulted abnormal.

Source: XXXX.SAS, Run on DDMMMYYYY

TABLE 101. Dermatological examination during the study – ARM B

POPULATION: SAF **< Note for SAS Programmer: the following must be presented for each visit: Baseline 1, Q8W, Baseline 2, Q8W >**

	Statistic	Arm B (N=XX)
<Visit n>		
Dermatological Examination performed		
No	n (%)	xx (xx.x%)
Yes	n (%)	xx (xx.x%)
Result (*)		
Normal	n (%)	xx (xx.x%)
Abnormal	n (%)	xx (xx.x%)
Diagnosis (§)		
Squamous cell carcinoma	n (%)	xx (xx.x%)
Keratoacanthomas	n (%)	xx (xx.x%)
Other pathology findings	n (%)	xx (xx.x%)
Evidence of severe or uncontrolled systemic disease or concurrent undesirable condition		
No	n (%)	xx (xx.x%)
Yes	n (%)	xx (xx.x%)

Notes:
Percentages are calculated relative to the total number of patients of ARM B in the SAF set.
(*) Percentages are calculated relative to the total number of patients of ARM B in the SAF set who performed the dermatological examination.
(§) Percentages are calculated relative to the total number of patients of ARM B in the SAF set who resulted abnormal.

Source: XXXX.SAS, Run on DDMMMYYYY

TABLE 102. Dermatological examination during the study – ARM C

POPULATION: SAF **< Note for SAS Programmer: the following must be presented for each visit: Baseline 1, Baseline 2, Q8W, Baseline 3>**

	Statistic	Arm C (N=XX)
<Visit n>		
Dermatological Examination performed		
No	n (%)	xx (xx.x%)
Yes	n (%)	xx (xx.x%)
Result (*)		
Normal	n (%)	xx (xx.x%)
Abnormal	n (%)	xx (xx.x%)
Diagnosis (§)		
Squamous cell carcinoma	n (%)	xx (xx.x%)
Keratoacanthomas	n (%)	xx (xx.x%)
Other pathology findings	n (%)	xx (xx.x%)
Evidence of severe or uncontrolled systemic disease or concurrent undesirable condition		
No	n (%)	xx (xx.x%)
Yes	n (%)	xx (xx.x%)

Notes:
Percentages are calculated relative to the total number of patients of ARM C in the SAF set.
(*) Percentages are calculated relative to the total number of patients of ARM C in the SAF set who performed the dermatological examination.
(§) Percentages are calculated relative to the total number of patients of ARM C in the SAF set who resulted abnormal.

Source: XXXX.SAS, Run on DDMMMYYYY

TABLE 103. Performance Status during the study – ARM A

POPULATION: SAF

ECOG PS	Statistic	Arm A (N=XX)
<Visit n>		
0	n (%)	xx (xx.x%)
1	n (%)	xx (xx.x%)
2	n (%)	xx (xx.x%)
3	n (%)	xx (xx.x%)
4	n (%)	xx (xx.x%)
5	n (%)	xx (xx.x%)

Notes:
Percentages are calculated relative to the total number of patients of the SAF set in the ARM A.

Source: XXXX.SAS, Run on DDMMMYYYY

< Note for SAS Programmer: In this table ECOG PS at all visits must be reported.>

TABLE 104. Performance Status during the study – ARM B

POPULATION: SAF

ECOG PS	Statistic	Arm B (N=XX)
<Visit n>		
0	n (%)	xx (xx.x%)
1	n (%)	xx (xx.x%)
2	n (%)	xx (xx.x%)
3	n (%)	xx (xx.x%)
4	n (%)	xx (xx.x%)
5	n (%)	xx (xx.x%)

Notes:
Percentages are calculated relative to the total number of patients of the SAF set in the ARM B.

Source: XXXX.SAS, Run on DDMMMYYYY

< Note for SAS Programmer: In this table ECOG PS at all visits must be reported.>

TABLE 105. Performance Status during the study – ARM C

POPULATION: SAF

ECOG PS	Statistic	Arm C (N=XX)
<Visit n>		
0	n (%)	xx (xx.x%)
1	n (%)	xx (xx.x%)
2	n (%)	xx (xx.x%)
3	n (%)	xx (xx.x%)
4	n (%)	xx (xx.x%)
5	n (%)	xx (xx.x%)

Notes:
Percentages are calculated relative to the total number of patients of the SAF set in the ARM C.

Source: XXXX.SAS, Run on DDMMMYYYY

< Note for SAS Programmer: In this table ECOG PS at all visits must be reported.>

TABLE 106. Pregnancy test during the study

POPULATION: SAF

ECOG PS	Statistic	Arm A (N=XX)	Arm B (N=XX)	Arm C (N=XX)
Baseline 1				
Negative	n (%)	xx (xx.x%)	xx (xx.x%)	xx (xx.x%)
Positive	n (%)	xx (xx.x%)	xx (xx.x%)	xx (xx.x%)
<Every 6 weeks>				
Negative	n (%)	xx (xx.x%)	xx (xx.x%)	xx (xx.x%)
Positive	n (%)	xx (xx.x%)	xx (xx.x%)	xx (xx.x%)
Follow-up visit				
Negative	n (%)	xx (xx.x%)	xx (xx.x%)	xx (xx.x%)
Positive	n (%)	xx (xx.x%)	xx (xx.x%)	xx (xx.x%)
Long-term follow-up visit				
Negative	n (%)	xx (xx.x%)	xx (xx.x%)	xx (xx.x%)
Positive	n (%)	xx (xx.x%)	xx (xx.x%)	xx (xx.x%)

Notes:
Percentages are calculated relative to the total number of female patients of the SAF set by treatment arm.

Source: XXXX.SAS, Run on DDMMMYYYY

Listing 1) Screening Failures

POPULATION: ENR

Site	Patient Number	Category	Criteria violated number	Criteria Description
XXX	XXXXXXXX	Inclusion/Exclusion	X	XXXXXXXXXXXXXXXXXXXXXXXXXXXXXXXXXXXXXXXXXXXXXXXXXXXXXXX
XXX	XXXXXXXX	XXXXXXXX	X	XXXXXXXXXXXXXXXXXXXXXXXXXXXXXXXXXXXXXXXXXXXXXXXXXXXXXXX
XXX	XXXXXXXX	XXXXXXXX	X	XXXXXXXXXXXXXXXXXXXXXXXXXXXXXXXXXXXXXXXXXXXXXXXXXXXXXXX
XXX	XXXXXXXX	XXXXXXXX	X	XXXXXXXXXXXXXXXXXXXXXXXXXXXXXXXXXXXXXXXXXXXXXXXXXXXXXXX

Source: XXXX.SAS, Run on DDMMMYYYY

Listing 2) Inclusion Criteria

POPULATION: ENR

			Inclusion Criteria							
Treatment Arm	Site	Patient Number	#1	#2	#3	#4	#5	..	#14	#15
XXXXXXXX	XXX	XXXXXXXX	Y/N	X	X	X	X	X	X	X
XXXXXXXX	XXX	XXXXXXXX	X	X	X	X	X	X	X	X
XXXXXXXX	XXX	XXXXXXXX	X	X	X	X	X	X	X	X
XXXXXXXX	XXX	XXXXXXXX	X	X	X	X	X	X	X	X

Source: XXXX.SAS, Run on DDMMMYYYY

Listing 3) Exclusion Criteria

POPULATION: ENR

			Exclusion Criteria							
Treatment Arm	Site	Patient Number	#1	#2	#3	#4	#5	..	#13	#14
XXXXXXXX	XXX	XXXXXXXX	X	X	X	X	X	X	X	X
XXXXXXXX	XXX	XXXXXXXX	X	X	X	X	X	X	X	X
XXXXXXXX	XXX	XXXXXXXX	X	X	X	X	X	X	X	X
XXXXXXXX	XXX	XXXXXXXX	X	X	X	X	X	X	X	X

Source: XXXX.SAS, Run on DDMMMYYYY

Listing 4) End of treatment

POPULATION: ENR

Site	Patient Number	Treatment Arm	Date of visit (DDMMMYYYY)	Date when the IMP was last taken (DDMMMYYYY)	Has the subject completed the trial?	Date of termination (DDMMMYYYY)	Date of withdrawal (DDMMMYYYY)	Primary reason for withdrawal	Please specify	If SAE was the reason for withdrawal, has it been reported to the Sponsor?
XXX	XXX	XXX	DDMMMYYYY	DDMMMYYYY	XXX	DDMMMYYYY	DDMMMYYYY	XXXXXXXXXXXXXX	XXXXXXXXXXXXXX	XXX
XXX	XXX	XXX	DDMMMYYYY	DDMMMYYYY	XXX	DDMMMYYYY	DDMMMYYYY	XXXXXXXXXXXXXX	XXXXXXXXXXXXXX	XXX
XXX	XXX	XXX	DDMMMYYYY	DDMMMYYYY	XXX	DDMMMYYYY	DDMMMYYYY	XXXXXXXXXXXXXX	XXXXXXXXXXXXXX	XXX
XXX	XXX	XXX	DDMMMYYYY	DDMMMYYYY	XXX	DDMMMYYYY	DDMMMYYYY	XXXXXXXXXXXXXX	XXXXXXXXXXXXXX	XXX

Source: XXXX.SAS, Run on DDMMMYYYY

Listing 5) Analysis Sets

POPULATION: ENR

Site	Patient Number	Treatment Arm	Inclusion in ENR?	Inclusion in ITT?	Reason for exclusion from ITT	Inclusion in SAF?	Reason for exclusion from SAF	Inclusion in PP?	Reason for exclusion from PP
XXX	XXXXXXXX	XXXXXXXX	X	X	XXXXXXXXXX XX	X	XXXXXXXXXXX XX	X	XXXXXXXXXXX XX
XXX	XXXXXXXX	XXXXXXXX	X	X	XXXXXXXXXX XX	X	XXXXXXXXXXX XX	X	XXXXXXXXXXX XX
XXX	XXXXXXXX	XXXXXXXX	X	X	XXXXXXXXXX XX	X	XXXXXXXXXXX XX	X	XXXXXXXXXXX XX
XXX	XXXXXXXX	XXXXXXXX	X	X	XXXXXXXXXX XX	X	XXXXXXXXXXX XX	X	XXXXXXXXXXX XX

Source: XXXX.SAS, Run on DDMMMYYYY

Listing 6) Major Protocol Deviations

POPULATION: ENR

Site	Patient Number	Treatment Arm	Gender	Age (yrs)	Type	Description of major protocol deviation
XXX	XXXXXXXX	XXX	XXX	XXX	XXXXXXXXXXXX	XXXXXXXXXXXX
XXX	XXXXXXXX	XXX	XXX	XXX	XXXXXXXXXXXX	XXXXXXXXXXXX
XXX	XXXXXXXX	XXX	XXX	XXX	XXXXXXXXXXXX	XXXXXXXXXXXX
XXX	XXXXXXXX	XXX	XXX	XXX	XXXXXXXXXXXX	XXXXXXXXXXXX

Source: XXXX.SAS, Run on DDMMMYYYY

Listing 7) Enrollment visit and signature of informed consent

POPULATION: ENR

Site	Patient Number	Treatment Arm	Date of enrollment visit (DDMMMYYYY)	Was informed consent obtained?	Date of signature of informed consent (DDMMMYYYY)	Was optional translational research informed consent obtained?	Date of signature of optional informed consent (DDMMMYYYY)
XXX	XXX	XXX	DDMMMYYYY	XXX	DDMMMYYYY	XXX	DDMMMYYYY
XXX	XXX	XXX	DDMMMYYYY	XXX	DDMMMYYYY	XXX	DDMMMYYYY
XXX	XXX	XXX	DDMMMYYYY	XXX	DDMMMYYYY	XXX	DDMMMYYYY
XXX	XXX	XXX	DDMMMYYYY	XXX	DDMMMYYYY	XXX	DDMMMYYYY

Source: XXXX.SAS, Run on DDMMMYYYY

Listing 8) Demographic Characteristics

POPULATION: ITT

Treatment Arm	Sit e	Patient Number	Gender at birth	Year of birth (YYYY)	Derived Age (years)	Race	Ethnic origin	Weight (Kg)	Height (cm)	BMI (kg/m^2)
XXX	XXX	XXX	XXX	YYYY	XXX	XXX	XXX	XXX	XXX	XXX
XXX	XXX	XXX	XXX	YYYY	XXX	XXX	XXX	XXX	XXX	XXX
XXX	XXX	XXX	XXX	YYYY	XXX	XXX	XXX	XXX	XXX	XXX
XXX	XXX	XXX	XXX	YYYY	XXX	XXX	XXX	XXX	XXX	XXX

Source: XXXX.SAS, Run on DDMMMYYYY

Listing 9) Disease History of Melanoma: Initial Diagnosis

POPULATION: ITT

Treatment Arm	Site	Patient Number	Melanoma Type	If mucosal specify primary site	If cutaneous, specify primary site	Melanoma Subtype	Date of first diagnosis of melanoma (DDMMMYYYY)	Time from first diagnosis of melanoma (months)	Stage at initial diagnosis
XXX	XXX	XXX	XXX	XXX	XXX	XXX	DDMMMYYYY	XXX	XXX
XXX	XXX	XXX	XXX	XXX	XXX	XXX	DDMMMYYYY	XXX	XXX
XXX	XXX	XXX	XXX	XXX	XXX	XXX	DDMMMYYYY	XXX	XXX
XXX	XXX	XXX	XXX	XXX	XXX	XXX	DDMMMYYYY	XXX	XXX

Source: XXXX.SAS, Run on DDMMMYYYY

Listing 10) Disease History of Melanoma: Current Diagnosis

POPULATION: ITT

Treatment Arm	Site	Patient Number	Stage at current diagnosis	Date of first recurrence/relapse (DDMMMYYYY)	Date of most recent recurrence/relapse (DDMMMMYYY)	Time from first recurrence/ relapse (months)	Time from most recent recurrence/ relapse (months)
XXX	XXX	XXX	XXX	DDMMMYYYY	DDMMMYYYY	XXX	XXX
XXX	XXX	XXX	XXX	DDMMMYYYY	DDMMMYYYY	XXX	XXX
XXX	XXX	XXX	XXX	DDMMMYYYY	DDMMMYYYY	XXX	XXX
XXX	XXX	XXX	XXX	DDMMMYYYY	DDMMMYYYY	XXX	XXX

Source: XXXX.SAS, Run on DDMMMYYYY

Listing 11) BRAF mutation testing and molecular status

POPULATION: ITT

Treatment Arm	Site	Patient Number	Date of assessment (DDMMMYYYY)	Molecular status (local) mutant	Type of mutation	If other, specify
XXX	XXX	XXX	DDMMMYYYY	XXX	XXX	XXX
XXX	XXX	XXX	DDMMMYYYY	XXX	XXX	XXX
XXX	XXX	XXX	DDMMMYYYY	XXX	XXX	XXX
XXX	XXX	XXX	DDMMMYYYY	XXX	XXX	XXX

Source: XXXX.SAS, Run on DDMMMYYYY

Listing 12) Childbearing potential status

POPULATION: ITT

Treatment Arm	Site	Patient Number	Childbearing potential / sexually active with women childbearing potential?	Is the subject post-menopausal?	Does the subject accept to use adequate contraception for the total study duration?
XXX	XXX	XXX	XXX	XXX	XXX
XXX	XXX	XXX	XXX	XXX	XXX
XXX	XXX	XXX	XXX	XXX	XXX
XXX	XXX	XXX	XXX	XXX	XXX

Source: XXXX.SAS, Run on DDMMMYYYY

Listing 13) HIV test at screening

POPULATION: ITT

Treatmen t Arm	Site	Patient Number	Has an HIV test been performed?	If not performed, specify reason	Date of HIV test (DDMMMYYYY)	HIV test result
XXX	XXX	XXX	XXX	XXX	DDMMMYYYY	XXX
XXX	XXX	XXX	XXX	XXX	DDMMMYYYY	XXX
XXX	XXX	XXX	XXX	XXX	DDMMMYYYY	XXX
XXX	XXX	XXX	XXX	XXX	DDMMMYYYY	XXX

Source: XXXX.SAS, Run on DDMMMYYYY

Listing 14) Medical History: previous diseases

POPULATION: ITT

Treatment Arm	Site	Patient Number	Verbatim	SOC	Preferred Term	Start Date (DDMMMYYYY)	Stop Date (DDMMMYYYY)	Ongoing?	Under treatment?
XXX	XXX	XXX	XXXXXXX	XXXXXXX	XXXXXXX	DDMMMYYYY	DDMMMYYYY	XXX	XXX
XXX	XXX	XXX	XXXXXXX	XXXXXXX	XXXXXXX	DDMMMYYYY	DDMMMYYYY	XXX	XXX
XXX	XXX	XXX	XXXXXXX	XXXXXXX	XXXXXXX	DDMMMYYYY	DDMMMYYYY	XXX	XXX
XXX	XXX	XXX	XXXXXXX	XXXXXXX	XXXXXXX	DDMMMYYYY	DDMMMYYYY	XXX	XXX

Note: Medical History: previous diseases were coded using the MedDRA Version XX.X.

Source: XXXX.SAS, Run on DDMMMYYYY

Listing 15) Medical History: concomitant diseases

POPULATION: ITT

Treatment Arm	Site	Patient Number	Verbatim	SOC	Preferred Term	Start Date (DDMMMYYYY)	Stop Date (DDMMMYYYY)	Ongoing?	Under treatment?
XXX	XXX	XXX	XXXXXXX	XXXXXXX	XXXXXXX	DDMMMYYYY	DDMMMYYYY	XXX	XXX
XXX	XXX	XXX	XXXXXXX	XXXXXXX	XXXXXXX	DDMMMYYYY	DDMMMYYYY	XXX	XXX
XXX	XXX	XXX	XXXXXXX	XXXXXXX	XXXXXXX	DDMMMYYYY	DDMMMYYYY	XXX	XXX
XXX	XXX	XXX	XXXXXXX	XXXXXXX	XXXXXXX	DDMMMYYYY	DDMMMYYYY	XXX	XXX

Note: Medical History: concomitant diseases were coded using the MedDRA Version XX.X.

Source: XXXX.SAS, Run on DDMMMYYYY

Listing 16) Prior Systemic Treatments

POPULATION: ITT

Treatment Arm	Site	Patient Number	MED (#)	Type	Other, specify	Drug	Generic name	3rd level ATC subgroup	Dosage	Unit	Start Date (DDMMMYYYY)	Stop Date (DDMMMYYYY)	Ongoing
XXX	XXX	XXX	XX	XXXXXXX	XXXXXXX	XXXXXXX	XXXXXXX	XXXXXXX	XXX	XXX	DDMMMYYYY	DDMMMYYYY	XXX
			XX	XXXXXXX	XXXXXXX	XXXXXXX	XXXXXXX	XXXXXXX	XXX	XXX	DDMMMYYYY	DDMMMYYYY	XXX
			XX	XXXXXXX	XXXXXXX	XXXXXXX	XXXXXXX	XXXXXXX	XXX	XXX	DDMMMYYYY	DDMMMYYYY	XXX
			XX	XXXXXXX	XXXXXXX	XXXXXXX	XXXXXXX	XXXXXXX	XXX	XXX	DDMMMYYYY	DDMMMYYYY	XXX
XXX	XXX	XXX	XX	XXXXXXX	XXXXXXX	XXXXXXX	XXXXXXX	XXXXXXX	XXX	XXX	DDMMMYYYY	DDMMMYYYY	XXX
			XX	XXXXXXX	XXXXXXX	XXXXXXX	XXXXXXX	XXXXXXX	XXX	XXX	DDMMMYYYY	DDMMMYYYY	XXX
			XX	XXXXXXX	XXXXXXX	XXXXXXX	XXXXXXX	XXXXXXX	XXX	XXX	DDMMMYYYY	DDMMMYYYY	XXX
			XX	XXXXXXX	XXXXXXX	XXXXXXX	XXXXXXX	XXXXXXX	XXX	XXX	DDMMMYYYY	DDMMMYYYY	XXX
XXX	XXX	XXX	XX	XXXXXXX	XXXXXXX	XXXXXXX	XXXXXXX	XXXXXXX	XXX	XXX	DDMMMYYYY	DDMMMYYYY	XXX

Note: Prior Systemic Treatments were coded using the WHO-DRL Version XXXX.

Source: XXXX.SAS, Run on DDMMMYYYY

Listing 17) Concomitant Systemic Treatments

POPULATION: ITT

Treatment Arm	Site	Patient Number	MED (#)	Type	Other, specify	Drug	Generic name	3rd level ATC subgroup	Dosage	Unit	Start Date (DDMMMYYYY)	Stop Date (DDMMMYYYY)	Ongoing
XXX	XXX	XXX	XX	XXXXXXX	XXXXXXX	XXXXXXX	XXXXXXX	XXXXXXX	XXX	XXX	DDMMMYYYY	DDMMMYYYY	XXX
			XX	XXXXXXX	XXXXXXX	XXXXXXX	XXXXXXX	XXXXXXX	XXX	XXX	DDMMMYYYY	DDMMMYYYY	XXX
			XX	XXXXXXX	XXXXXXX	XXXXXXX	XXXXXXX	XXXXXXX	XXX	XXX	DDMMMYYYY	DDMMMYYYY	XXX
			XX	XXXXXXX	XXXXXXX	XXXXXXX	XXXXXXX	XXXXXXX	XXX	XXX	DDMMMYYYY	DDMMMYYYY	XXX
XXX	XXX	XXX	XX	XXXXXXX	XXXXXXX	XXXXXXX	XXXXXXX	XXXXXXX	XXX	XXX	DDMMMYYYY	DDMMMYYYY	XXX
			XX	XXXXXXX	XXXXXXX	XXXXXXX	XXXXXXX	XXXXXXX	XXX	XXX	DDMMMYYYY	DDMMMYYYY	XXX
			XX	XXXXXXX	XXXXXXX	XXXXXXX	XXXXXXX	XXXXXXX	XXX	XXX	DDMMMYYYY	DDMMMYYYY	XXX
			XX	XXXXXXX	XXXXXXX	XXXXXXX	XXXXXXX	XXXXXXX	XXX	XXX	DDMMMYYYY	DDMMMYYYY	XXX
XXX	XXX	XXX	XX	XXXXXXX	XXXXXXX	XXXXXXX	XXXXXXX	XXXXXXX	XXX	XXX	DDMMMYYYY	DDMMMYYYY	XXX

Note: Concomitant Systemic Treatments were coded using the WHO-DRL Version XXXX.

Source: XXXX.SAS, Run on DDMMMYYYY

Listing 18) Prior Radiation Treatments

POPULATION: ITT

Treatment Arm	Site	Patient Number	MED (#)	Site	Generic name	3rd level ATC subgroup	Total cumulative dose (Gy)	Start Date of the last radiotherapy (DDMMMYYYY)	Stop Date of the last radiotherapy (DDMMMYYYY)	Purpose
XXX	XXX	XXX	XX	XXXXXX X	XXXXXXX	XXXXXXX	XXXXXXX	DDMMMYYYY	DDMMMYYYY	XXX
			XX	XXXXXX X	XXXXXXX	XXXXXXX	XXXXXXX	DDMMMYYYY	DDMMMYYYY	XXX
			XX	XXXXXX X	XXXXXXX	XXXXXXX	XXXXXXX	DDMMMYYYY	DDMMMYYYY	XXX
			XX	XXXXXX X	XXXXXXX	XXXXXXX	XXXXXXX	DDMMMYYYY	DDMMMYYYY	XXX
XXX	XXX	XXX	XX	XXXXXX X	XXXXXXX	XXXXXXX	XXXXXXX	DDMMMYYYY	DDMMMYYYY	XXX
			XX	XXXXXX X	XXXXXXX	XXXXXXX	XXXXXXX	DDMMMYYYY	DDMMMYYYY	XXX
			XX	XXXXXX X	XXXXXXX	XXXXXXX	XXXXXXX	DDMMMYYYY	DDMMMYYYY	XXX
			XX	XXXXXX X	XXXXXXX	XXXXXXX	XXXXXXX	DDMMMYYYY	DDMMMYYYY	XXX
XXX	XXX	XXX	XX	XXXXXX X	XXXXXXX	XXXXXXX	XXXXXXX	DDMMMYYYY	DDMMMYYYY	XXX

Note: Prior Radiation Treatments were coded using the WHO-DRL Version XXXX.

Source: XXXX.SAS, Run on DDMMMYYYY

Listing 19) Prior melanoma-related surgery

POPULATION: ITT

Treatment Arm	Site	Patient Number	SURG (#)	Procedure	If other, specify	Anatomic Site involved	If other, specify	Generic name	3rd level ATC subgroup	Date of surgery (DDMMMYYYY)	Type of resection (if applicable)	Specify if remarkable comments
XXX	XXX	XXX	XX	XXXXXXX	XXXXXXX	XXXXXXX	XXXXXXX	XXXXXXX	XXXXXXX	DDMMMYYYY	XXX	XXX
			XX	XXXXXXX	XXXXXXX	XXXXXXX	XXXXXXX	XXXXXXX	XXXXXXX	DDMMMYYYY	XXX	XXX
			XX	XXXXXXX	XXXXXXX	XXXXXXX	XXXXXXX	XXXXXXX	XXXXXXX	DDMMMYYYY	XXX	XXX
			XX	XXXXXXX	XXXXXXX	XXXXXXX	XXXXXXX	XXXXXXX	XXXXXXX	DDMMMYYYY	XXX	XXX
XXX	XXX	XXX	XX	XXXXXXX	XXXXXXX	XXXXXXX	XXXXXXX	XXXXXXX	XXXXXXX	DDMMMYYYY	XXX	XXX
			XX	XXXXXXX	XXXXXXX	XXXXXXX	XXXXXXX	XXXXXXX	XXXXXXX	DDMMMYYYY	XXX	XXX
			XX	XXXXXXX	XXXXXXX	XXXXXXX	XXXXXXX	XXXXXXX	XXXXXXX	DDMMMYYYY	XXX	XXX
			XX	XXXXXXX	XXXXXXX	XXXXXXX	XXXXXXX	XXXXXXX	XXXXXXX	DDMMMYYYY	XXX	XXX
XXX	XXX	XXX	XX	XXXXXXX	XXXXXXX	XXXXXXX	XXXXXXX	XXXXXXX	XXXXXXX	DDMMMYYYY	XXX	XXX

Note: Prior melanoma-related surgery was coded using the WHO-DRL Version XXXX.

Source: XXXX.SAS, Run on DDMMMYYYY

Listing 20) Serum/urine pregnancy test

POPULATION: ITT

Treatment Arm	Site	Patient Number	Serum Pregnancy test performed?	If not performed, specify reason	Date of pregnancy test (DDMMMYYYY)	Beta Serum pregnancy test result (mIU/ml)	If abnormal, CS or NCS?	Urine pregnancy test result
XXX	XXX	XXX	XXX	XXX	DDMMMYYYY	XXX	XXX	XXX
			XXX	XXX	DDMMMYYYY	XXX	XXX	XXX
			Etc.					
XXX	XXX	XXX	XXX	XXX	DDMMMYYYY	XXX	XXX	XXX
			XXX	XXX	DDMMMYYYY	XXX	XXX	XXX
			Etc.					
XXX	XXX	XXX	XXX	XXX	DDMMMYYYY	XXX	XXX	XXX
			XXX	XXX	DDMMMYYYY	XXX	XXX	XXX
			Etc.					
XXX	XXX	XXX	XXX	XXX	DDMMMYYYY	XXX	XXX	XXX
			XXX	XXX	DDMMMYYYY	XXX	XXX	XXX
			Etc.					

Note: CS = Clinically Significant; NCS = Not Clinically Significant.

Source: XXXX.SAS, Run on DDMMMYYYY

Listing 21) Vital signs and ECOG PS

POPULATION: ITT

Treatment Arm	Site	Patient Number	Visit	Vital signs measured?	If not, Reason	Date of assessment (DDMMMYYYY)	Supine SBP (mmHg)	Supine DBP (mmHg)	Heart Rate (bpm)	Cardiovascular signs/symptoms?	If yes, specify	Body temperature (°C)	Respiratory Rate (breaths/min)	ECOG PS
XXX	XXX	XXX	XXX	XXX	XXX	DDMMMYYYY	XXX	XXX	XXX	XXX	XXX	XXX	XXX	XXX
			XXX	XXX	XXX	DDMMMYYYY	XXX	XXX	XXX	XXX	XXX	XXX	XXX	XXX
			Etc.											
XXX	XXX	XXX	XXX	XXX	XXX	DDMMMYYYY	XXX	XXX	XXX	XXX	XXX	XXX	XXX	XXX
			XXX	XXX	XXX	DDMMMYYYY	XXX	XXX	XXX	XXX	XXX	XXX	XXX	XXX
			Etc.											
XXX	XXX	XXX	XXX	XXX	XXX	DDMMMYYYY	XXX	XXX	XXX	XXX	XXX	XXX	XXX	XXX
			XXX	XXX	XXX	DDMMMYYYY	XXX	XXX	XXX	XXX	XXX	XXX	XXX	XXX
			Etc.											
XXX	XXX	XXX	XXX	XXX	XXX	DDMMMYYYY	XXX	XXX	XXX	XXX	XXX	XXX	XXX	XXX
			XXX	XXX	XXX	DDMMMYYYY	XXX	XXX	XXX	XXX	XXX	XXX	XXX	XXX
			Etc.											

Source: XXXX.SAS, Run on DDMMMYYYY

Listing 22) Physical Examination

POPULATION: ITT

Treatment Arm	Site	Patient Number	Visit	Physical Examination performed?	If not performed, reason	Date of physical examination (DDMMMYYYY)	Body area	Examination finding	If abnormal, specify	SOC	Preferred Term
XXX	XXX	XXX	XXXXXXX	XXX	XXXXXXX	DDMMMYYYY	XXX	XXX	XXX	XXX	XXX
							XXX	XXX	XXX	XXX	XXX
							XXX	XXX	XXX	XXX	XXX
							XXX	XXX	XXX	XXX	XXX
			XXXXXXX	XXX	XXXXXXX	DDMMMYYYY	XXX	XXX	XXX	XXX	XXX
							XXX	XXX	XXX	XXX	XXX
							XXX	XXX	XXX	XXX	XXX
							XXX	XXX	XXX	XXX	XXX
XXX	XXX	XXX	XXXXXXX	XXX	XXXXXXX	DDMMMYYYY	XXX	XXX	XXX	XXX	XXX

Note: Physical Examination were coded using the MedDRA Version XX.X.

Source: XXXX.SAS, Run on DDMMMYYYY

Listing 23) Hematology

POPULATION: SAF

															SI	
Treatment Arm	Site	Patient Number	Visit	Sample collected?	If not, specify reason	Sample date (DDMMMYYYY)	Exam	Done?	Result	Unit	LLN	ULN	Normal reference	If abnormal, CS?	Result	Unit
XXX	XXX	XXXXXXXX	XXX	XXX	XXX	DDMMMYYYY	XXX	XXX	XXX	XXX	XXX	XXX	XXX	XXX	XXX	XXX
			XXX	XXX	XXX	DDMMMYYYY	XXX	XXX	XXX	XXX	XXX	XXX	XXX	XXX	XXX	XXX
			Etc.													
XXX	XXX	XXXXXXXX	XXX	XXX	XXX	DDMMMYYYY	XXX	XXX	XXX	XXX	XXX	XXX	XXX	XXX	XXX	XXX
			XXX	XXX	XXX	DDMMMYYYY	XXX	XXX	XXX	XXX	XXX	XXX	XXX	XXX	XXX	XXX
			Etc.													
XXX	XXX	XXXXXXXX	XXX	XXX	XXX	DDMMMYYYY	XXX	XXX	XXX	XXX	XXX	XXX	XXX	XXX	XXX	XXX
			XXX	XXX	XXX	DDMMMYYYY	XXX	XXX	XXX	XXX	XXX	XXX	XXX	XXX	XXX	XXX
			Etc.													
XXX	XXX	XXXXXXXX	XXX	XXX	XXX	DDMMMYYYY	XXX	XXX	XXX	XXX	XXX	XXX	XXX	XXX	XXX	XXX
			XXX	XXX	XXX	DDMMMYYYY	XXX	XXX	XXX	XXX	XXX	XXX	XXX	XXX	XXX	XXX
			Etc.													

Source: XXXX.SAS, Run on DDMMMYYYY

Listing 24) Blood Chemistry

POPULATION: SAF

															SI	
Treatment Arm	Site	Patient Number	Visit	Sample collected?	If not, specify reason	Sample date (DDMMMYYYY)	Exam	Done?	Result	Unit	LLN	ULN	Normal reference	If abnormal, CS?	Result	Unit
XXX	XXX	XXXXXXXX	XXX	XXX	XXX	DDMMMYYYY	XXX	XXX	XXX	XXX	XXX	XXX	XXX	XXX	XXX	XXX
			XXX	XXX	XXX	DDMMMYYYY	XXX	XXX	XXX	XXX	XXX	XXX	XXX	XXX	XXX	XXX
			Etc.													
XXX	XXX	XXXXXXXX	XXX	XXX	XXX	DDMMMYYYY	XXX	XXX	XXX	XXX	XXX	XXX	XXX	XXX	XXX	XXX
			XXX	XXX	XXX	DDMMMYYYY	XXX	XXX	XXX	XXX	XXX	XXX	XXX	XXX	XXX	XXX
			Etc.													
XXX	XXX	XXXXXXXX	XXX	XXX	XXX	DDMMMYYYY	XXX	XXX	XXX	XXX	XXX	XXX	XXX	XXX	XXX	XXX
			XXX	XXX	XXX	DDMMMYYYY	XXX	XXX	XXX	XXX	XXX	XXX	XXX	XXX	XXX	XXX
			Etc.													
XXX	XXX	XXXXXXXX	XXX	XXX	XXX	DDMMMYYYY	XXX	XXX	XXX	XXX	XXX	XXX	XXX	XXX	XXX	XXX
			XXX	XXX	XXX	DDMMMYYYY	XXX	XXX	XXX	XXX	XXX	XXX	XXX	XXX	XXX	XXX
			Etc.													

Source: XXXX.SAS, Run on DDMMMYYYY

Listing 25) Coagulation

POPULATION: SAF

															SI	
Treatment Arm	Site	Patient Number	Visit	Sample collected?	If not, specify reason	Sample date (DDMMMYYYY)	Exam	Done?	Result	Unit	LLN	ULN	Normal reference	If abnormal, CS?	Result	Unit
XXX	XXX	XXXXXXXX	XXX	XXX	XXX	DDMMMYYYY	XXX	XXX	XXX	XXX	XXX	XXX	XXX	XXX	XXX	XXX
			XXX	XXX	XXX	DDMMMYYYY	XXX	XXX	XXX	XXX	XXX	XXX	XXX	XXX	XXX	XXX
			Etc.													
XXX	XXX	XXXXXXXX	XXX	XXX	XXX	DDMMMYYYY	XXX	XXX	XXX	XXX	XXX	XXX	XXX	XXX	XXX	XXX
			XXX	XXX	XXX	DDMMMYYYY	XXX	XXX	XXX	XXX	XXX	XXX	XXX	XXX	XXX	XXX
			Etc.													
XXX	XXX	XXXXXXXX	XXX	XXX	XXX	DDMMMYYYY	XXX	XXX	XXX	XXX	XXX	XXX	XXX	XXX	XXX	XXX
			XXX	XXX	XXX	DDMMMYYYY	XXX	XXX	XXX	XXX	XXX	XXX	XXX	XXX	XXX	XXX
			Etc.													
XXX	XXX	XXXXXXXX	XXX	XXX	XXX	DDMMMYYYY	XXX	XXX	XXX	XXX	XXX	XXX	XXX	XXX	XXX	XXX
			XXX	XXX	XXX	DDMMMYYYY	XXX	XXX	XXX	XXX	XXX	XXX	XXX	XXX	XXX	XXX
			Etc.													

Source: XXXX.SAS, Run on DDMMMYYYY

Listing 26) Urinalysis (dipstick analysis)

POPULATION: SAF

Treatment Arm	Site	Patient Number	Visit	Sample collected?	If not, specify reason	Date of assessment (DDMMMYYYY)	Exam	Done?	Result	If abnormal, CS?
XXX	XXX	XXXXXXXX	XXX	XXX	XXX	DDMMMYYYY	XXX	XXX	XXX	XXX
XXX	XXX	XXXXXXXX	XXX	XXX	XXX	DDMMMYYYY	XXX	XXX	XXX	XXX
XXX	XXX	XXXXXXXX	XXX	XXX	XXX	DDMMMYYYY	XXX	XXX	XXX	XXX
XXX	XXX	XXXXXXXX	XXX	XXX	XXX	DDMMMYYYY	XXX	XXX	XXX	XXX

Source: XXXX.SAS, Run on DDMMMYYYY

Listing 27) Microscopic analysis

POPULATION: SAF

Treatment Arm	Site	Patient Number	Visit	Sample collected?	If not, specify reason	Date of assessment (DDMMMYYYY)	Exam	State	Value	If abnormal, CS?
XXX	XXX	XXXXXXXX	XXX	XXX	XXX	DDMMMYYYY	XXX	XXX	XXX	XXX
			XXX	XXX	XXX	DDMMMYYYY	XXX	XXX	XXX	XXX
			Etc.							
XXX	XXX	XXXXXXXX	XXX	XXX	XXX	DDMMMYYYY	XXX	XXX	XXX	XXX
			XXX	XXX	XXX	DDMMMYYYY	XXX	XXX	XXX	XXX
			Etc.							
XXX	XXX	XXXXXXXX	XXX	XXX	XXX	DDMMMYYYY	XXX	XXX	XXX	XXX
			XXX	XXX	XXX	DDMMMYYYY	XXX	XXX	XXX	XXX
			Etc.							
XXX	XXX	XXXXXXXX	XXX	XXX	XXX	DDMMMYYYY	XXX	XXX	XXX	XXX
			XXX	XXX	XXX	DDMMMYYYY	XXX	XXX	XXX	XXX
			Etc.							

Source: XXXX.SAS, Run on DDMMMYYYY

Listing 28) Thyroid Function Test

POPULATION: SAF

															SI	
Treatment Arm	Site	Patient Number	Visit	Sample collected?	If not, specify reason	Sample date (DDMMMYYYY)	Exam	Done?	Result	Unit	LLN	ULN	Normal reference	If abnormal, CS?	Result	Unit
XXX	XXX	XXXXXXXX	XXX	XXX	XXX	DDMMMYYYY	XXX	XXX	XXX	XXX	XXX	XXX	XXX	XXX	XXX	XXX
			XXX	XXX	XXX	DDMMMYYYY	XXX	XXX	XXX	XXX	XXX	XXX	XXX	XXX	XXX	XXX
			Etc.													
XXX	XXX	XXXXXXXX	XXX	XXX	XXX	DDMMMYYYY	XXX	XXX	XXX	XXX	XXX	XXX	XXX	XXX	XXX	XXX
			XXX	XXX	XXX	DDMMMYYYY	XXX	XXX	XXX	XXX	XXX	XXX	XXX	XXX	XXX	XXX
			Etc.													
XXX	XXX	XXXXXXXX	XXX	XXX	XXX	DDMMMYYYY	XXX	XXX	XXX	XXX	XXX	XXX	XXX	XXX	XXX	XXX
			XXX	XXX	XXX	DDMMMYYYY	XXX	XXX	XXX	XXX	XXX	XXX	XXX	XXX	XXX	XXX
			Etc.													
XXX	XXX	XXXXXXXX	XXX	XXX	XXX	DDMMMYYYY	XXX	XXX	XXX	XXX	XXX	XXX	XXX	XXX	XXX	XXX
			XXX	XXX	XXX	DDMMMYYYY	XXX	XXX	XXX	XXX	XXX	XXX	XXX	XXX	XXX	XXX
			Etc.													

Source: XXXX.SAS, Run on DDMMMYYYY

Listing 29) Cardiac Muscle Enzyme

POPULATION: SAF

															SI	
Treatmen t Arm	Site	Patient Number	Visit	Sample collec ted?	If not, specify reason	Sample date (DDMMMYYYY)	Exa m	Done?	Result	Uni t	LLN	ULN	Normal referenc e	If abnormal , CS?	Result	Uni t
XXX	XXX	XXXXXXX X	XXX	XXX	XXX	DDMMMYYYY	XXX	XXX	XXX	XXX	XXX	XXX	XXX	XXX	XXX	XXX
			XXX	XXX	XXX	DDMMMYYYY	XXX	XXX	XXX	XXX	XXX	XXX	XXX	XXX	XXX	XXX
			Etc.													
XXX	XXX	XXXXXXX X	XXX	XXX	XXX	DDMMMYYYY	XXX	XXX	XXX	XXX	XXX	XXX	XXX	XXX	XXX	XXX
			XXX	XXX	XXX	DDMMMYYYY	XXX	XXX	XXX	XXX	XXX	XXX	XXX	XXX	XXX	XXX
			Etc.													
XXX	XXX	XXXXXXX X	XXX	XXX	XXX	DDMMMYYYY	XXX	XXX	XXX	XXX	XXX	XXX	XXX	XXX	XXX	XXX
			XXX	XXX	XXX	DDMMMYYYY	XXX	XXX	XXX	XXX	XXX	XXX	XXX	XXX	XXX	XXX
			Etc.													
XXX	XXX	XXXXXXX X	XXX	XXX	XXX	DDMMMYYYY	XXX	XXX	XXX	XXX	XXX	XXX	XXX	XXX	XXX	XXX
			XXX	XXX	XXX	DDMMMYYYY	XXX	XXX	XXX	XXX	XXX	XXX	XXX	XXX	XXX	XXX
			Etc.													

Source: XXXX.SAS, Run on DDMMMYYYY

Listing 30) Endocrine Panel

POPULATION: SAF

Treatment Arm	Site	Patient Number	Visit	Sample collected?	If not, specify reason	Sample date (DDMMMYYYY)	Exam	Done?	Result	Unit	LLN	ULN	Normal reference	If abnormal, CS?	SI Result	SI Unit
XXX	XXX	XXXXXXXX	XXX	XXX	XXX	DDMMMYYYY	XXX	XXX	XXX	XXX	XXX	XXX	XXX	XXX	XXX	XXX
			XXX	XXX	XXX	DDMMMYYYY	XXX	XXX	XXX	XXX	XXX	XXX	XXX	XXX	XXX	XXX
			Etc.													
XXX	XXX	XXXXXXXX	XXX	XXX	XXX	DDMMMYYYY	XXX	XXX	XXX	XXX	XXX	XXX	XXX	XXX	XXX	XXX
			XXX	XXX	XXX	DDMMMYYYY	XXX	XXX	XXX	XXX	XXX	XXX	XXX	XXX	XXX	XXX
			Etc.													
XXX	XXX	XXXXXXXX	XXX	XXX	XXX	DDMMMYYYY	XXX	XXX	XXX	XXX	XXX	XXX	XXX	XXX	XXX	XXX
			XXX	XXX	XXX	DDMMMYYYY	XXX	XXX	XXX	XXX	XXX	XXX	XXX	XXX	XXX	XXX
			Etc.													
XXX	XXX	XXXXXXXX	XXX	XXX	XXX	DDMMMYYYY	XXX	XXX	XXX	XXX	XXX	XXX	XXX	XXX	XXX	XXX
			XXX	XXX	XXX	DDMMMYYYY	XXX	XXX	XXX	XXX	XXX	XXX	XXX	XXX	XXX	XXX
			Etc.													

Source: XXXX.SAS, Run on DDMMMYYYY

Listing 31) ECG

POPULATION: SAF

Treatment Arm	Site	Patient Number	Visit	Was ECG performed?	If not, specify reason	Date of assessment (DDMMMYYYY)	Result	If abnormal, NCS/CS	PR interval (ms)	QT interval (ms)	QRS interval (ms)	QTcB interval (ms)	Cardiac axis(°)
XXX	XXX	XXXXXXXX	XXX	XXX	XXX	DDMMMYYYY	XXX	XXX	XXX	XXX	XXX	XXX	XXX
XXX	XXX	XXXXXXXX	XXX	XXX	XXX	DDMMMYYYY	XXX	XXX	XXX	XXX	XXX	XXX	XXX
XXX	XXX	XXXXXXXX	XXX	XXX	XXX	DDMMMYYYY	XXX	XXX	XXX	XXX	XXX	XXX	XXX
XXX	XXX	XXXXXXXX	XXX	XXX	XXX	DDMMMYYYY	XXX	XXX	XXX	XXX	XXX	XXX	XXX

Source: XXXX.SAS, Run on DDMMMYYYY

Listing 32) Left Ventricular Ejection Fraction

POPULATION: SAF

Treatment Arm	Site	Patient Number	Visit	Was the LVEF performed?	If not, specify reason	Date of scan (DDMMMYYYY)	Type of scan	Ejection Fraction Value (%)	Scan interpretation	Specify
XXX	XXX	XXXXXXXX	XXX	XXX	XXX	DDMMMYYYY	XXX	XXX	XXX	XXX
XXX	XXX	XXXXXXXX	XXX	XXX	XXX	DDMMMYYYY	XXX	XXX	XXX	XXX
XXX	XXX	XXXXXXXX	XXX	XXX	XXX	DDMMMYYYY	XXX	XXX	XXX	XXX
XXX	XXX	XXXXXXXX	XXX	XXX	XXX	DDMMMYYYY	XXX	XXX	XXX	XXX

Source: XXXX.SAS, Run on DDMMMYYYY

Listing 33) Ophthalmological examination: Visual Acuity and Intraocular pressure

POPULATION: SAF

Treatment Arm	Site	Patient Number	Visit	Performed?	Eye(s) assessed?	Visual acuity assessed?	If not, reason	Total Visual Acuity Score (ETDRS) – Left Eye (OS)	Total Visual Acuity Score (ETDRS) – Right Eye (OD)	Total Visual Acuity snellens equivalent – Left Eye (OS)	Total Visual Acuity snellens equivalent – Right Eye (OD)	Intraocular pressure assessed?	Intraocular pressure – left eye (OS) (mmHg)	Intraocular pressure – Right Eye (OD) (mmHg)
XXX	XXX	XXXXXXXX	XXX	XXX	XXX	XXX	XXX	XXX	XXX	XXX	XXX	XXX	XXX	XXX
XXX	XXX	XXXXXXXX	XXX	XXX	XXX	XXX	XXX	XXX	XXX	XXX	XXX	XXX	XXX	XXX
XXX	XXX	XXXXXXXX	XXX	XXX	XXX	XXX	XXX	XXX	XXX	XXX	XXX	XXX	XXX	XXX
XXX	XXX	XXXXXXXX	XXX	XXX	XXX	XXX	XXX	XXX	XXX	XXX	XXX	XXX	XXX	XXX

Source: XXXX.SAS, Run on DDMMMYYYY

Listing 34) Ophthalmological examination: Indirect Dilated Fundoscopy (ophthalmoscopy)

POPULATION: SAF

Treatment Arm	Site	Patient Number	Visit	Dilated fundoscopy performed?	If not, reason	Date of assessment (DDMMMYYYY)	Eye(s) assessed	Eye	Area	Done?	Assessment	If abnormal, specify
XXX	XXX	XXXXXXXX	XXX	XXX	XXX	DDMMMYYYY	XXX	Right	XXX	XXX	XXX	XXX
									XXX	XXX	XXX	XXX
									XXX	XXX	XXX	XXX
									XXX	XXX	XXX	XXX
XXX	XXX	XXXXXXXX	XXX	XXX	XXX	DDMMMYYYY	XXX	Left	XXX	XXX	XXX	XXX
									XXX	XXX	XXX	XXX
									XXX	XXX	XXX	XXX
									XXX	XXX	XXX	XXX
XXX	XXX	XXXXXXXX	XXX	XXX	XXX	DDMMMYYYY	XXX	XXX	XXX	XXX	XXX	XXX
									XXX	XXX	XXX	XXX
									XXX	XXX	XXX	XXX
									XXX	XXX	XXX	XXX
XXX	XXX	XXXXXXXX	XXX	XXX	XXX	DDMMMYYYY	XXX	XXX	XXX	XXX	XXX	XXX
									XXX	XXX	XXX	XXX
									XXX	XXX	XXX	XXX
									XXX	XXX	XXX	XXX

Source: XXXX.SAS, Run on DDMMMYYYY

Listing 35) Ophthalmological examination: Slit Lamp Examination

POPULATION: SAF

Treatment Arm	Site	Patient Number	Visit	Slit lamp examination performed?	If not, reason	Date of assessment (DDMMMYYYY)	Eye(s) assessed	Eye	Area	Done?	Assessment	If abnormal, specify
XXX	XXX	XXXXXXXX	XXX	XXX	XXX	DDMMMYYYY	XXX	Right	XXX	XXX	XXX	XXX
									XXX	XXX	XXX	XXX
									XXX	XXX	XXX	XXX
									XXX	XXX	XXX	XXX
XXX	XXX	XXXXXXXX	XXX	XXX	XXX	DDMMMYYYY	XXX	Left	XXX	XXX	XXX	XXX
									XXX	XXX	XXX	XXX
									XXX	XXX	XXX	XXX
									XXX	XXX	XXX	XXX
XXX	XXX	XXXXXXXX	XXX	XXX	XXX	DDMMMYYYY	XXX	XXX	XXX	XXX	XXX	XXX
									XXX	XXX	XXX	XXX
									XXX	XXX	XXX	XXX
									XXX	XXX	XXX	XXX
XXX	XXX	XXXXXXXX	XXX	XXX	XXX	DDMMMYYYY	XXX	XXX	XXX	XXX	XXX	XXX
									XXX	XXX	XXX	XXX
									XXX	XXX	XXX	XXX
									XXX	XXX	XXX	XXX

Source: XXXX.SAS, Run on DDMMMYYYY

Listing 36) Ophthalmological examination: Visual Field testing and optical coherence tomography

POPULATION: SAF

Treatment Arm	Site	Patient Number	Visit	Visual Field Testing performed?	If not, reason	Date of test (DDMMMYYYY)	Are there any abnormalities?	If abnormal, specify	Optical coherence tomography performed?	If not, reason	Date of tomography (DDMMMYYYY)	Are there any abnormalities?	If abnormal, specify
XXX	XXX	XXXXXXXX	XXX	XXX	XXX	DDMMMYYYY	XXX	XXX	XXX	XXX	DDMMMYYYY	XXX	XXX
XXX	XXX	XXXXXXXX	XXX	XXX	XXX	DDMMMYYYY	XXX	XXX	XXX	XXX	DDMMMYYYY	XXX	XXX
XXX	XXX	XXXXXXXX	XXX	XXX	XXX	DDMMMYYYY	XXX	XXX	XXX	XXX	DDMMMYYYY	XXX	XXX
XXX	XXX	XXXXXXXX	XXX	XXX	XXX	DDMMMYYYY	XXX	XXX	XXX	XXX	DDMMMYYYY	XXX	XXX

Source: XXXX.SAS, Run on DDMMMYYYY

Listing 37) Dermatological examination

POPULATION: SAF

Treatment Arm	Site	Patient Number	Visit	Dermatological examination performed?	If not, reason	Date of visit (DDMMMYYYY)	Result	Result abnormal, specify	Result abnormal, diagnosis	Evidence of severe or uncontrolled systemic disease or any concurrent condition
XXX	XXX	XXXXXXXX	XXX	XXX	XXX	DDMMMYYYY	XXX	XXX	XXX	XXX
XXX	XXX	XXXXXXXX	XXX	XXX	XXX	DDMMMYYYY	XXX	XXX	XXX	XXX
XXX	XXX	XXXXXXXX	XXX	XXX	XXX	DDMMMYYYY	XXX	XXX	XXX	XXX
XXX	XXX	XXXXXXXX	XXX	XXX	XXX	DDMMMYYYY	XXX	XXX	XXX	XXX

Source: XXXX.SAS, Run on DDMMMYYYY

Listing 38) Work Productivity and Activity Impairment Questionnaire (WPAI:GH)

POPULATION: ITT

Treatment Arm	Site	Patient Number	Visit	WPAI:GH questionnaire completed?	If not, reason	Date of visit (DDMMMYYYY)	Item #1	Item #2	..	Item #6	Percent work time missed due to health	Percent impairment while working due to health	Percent overall work impairment due to health	Percent activity impairment due to health
XXX	XXX	XXXXXXXX	XXX	XXX	XXX	DDMMMYYYY	XXX	XXX	XXX	XXX	XXX	XXX	XXX	XXX
XXX	XXX	XXXXXXXX	XXX	XXX	XXX	DDMMMYYYY	XXX	XXX	XXX	XXX	XXX	XXX	XXX	XXX
XXX	XXX	XXXXXXXX	XXX	XXX	XXX	DDMMMYYYY	XXX	XXX	XXX	XXX	XXX	XXX	XXX	XXX
XXX	XXX	XXXXXXXX	XXX	XXX	XXX	DDMMMYYYY	XXX	XXX	XXX	XXX	XXX	XXX	XXX	XXX

Source: XXXX.SAS, Run on DDMMMYYYY

Listing 39) General Health status EQ-5D-5L

POPULATION: ITT

Treatment Arm	Site	Patient Number	Visit	EQ-5D-5L questionnaire completed?	If not, reason	Date of visit (DDMMMYYYY)	Item #1	Item #2	..	Item #5	Health State	EQ-5D-5L index
XXX	XXX	XXXXXXXX	XXX	XXX	XXX	DDMMMYYYY	XXX	XXX	XXX	XXX	XXX	XXX
XXX	XXX	XXXXXXXX	XXX	XXX	XXX	DDMMMYYYY	XXX	XXX	XXX	XXX	XXX	XXX
XXX	XXX	XXXXXXXX	XXX	XXX	XXX	DDMMMYYYY	XXX	XXX	XXX	XXX	XXX	XXX
XXX	XXX	XXXXXXXX	XXX	XXX	XXX	DDMMMYYYY	XXX	XXX	XXX	XXX	XXX	XXX

Source: XXXX.SAS, Run on DDMMMYYYY

Listing 40) Health-related quality of life EORTC QLQ-C30: items

POPULATION: ITT

Treatment Arm	Site	Patient Number	Visit	EORTC-QLQ-C30 questionnaire completed?	If not, reason	Date of visit (DDMMMYYYY)	Item #1	Item #2	..	Item #30
XXX	XXX	XXXXXXXX	XXX	XXX	XXX	DDMMMYYYY	XXX	XXX	XXX	XXX
XXX	XXX	XXXXXXXX	XXX	XXX	XXX	DDMMMYYYY	XXX	XXX	XXX	XXX
XXX	XXX	XXXXXXXX	XXX	XXX	XXX	DDMMMYYYY	XXX	XXX	XXX	XXX
XXX	XXX	XXXXXXXX	XXX	XXX	XXX	DDMMMYYYY	XXX	XXX	XXX	XXX

Source: XXXX.SAS, Run on DDMMMYYYY

Listing 41) Health-related quality of life EORTC QLQ-C30: scale scores

POPULATION: ITT

Treatment Arm	Site	Patient Number	Visit	EORTC-QLQ-C30 questionnaire completed?	If not, reason	Date of visit (DDMMMYYYY)	QL2	PF2	..	FI
XXX	XXX	XXXXXXXX	XXX	XXX	XXX	DDMMMYYYY	XXX	XXX	XXX	XXX
XXX	XXX	XXXXXXXX	XXX	XXX	XXX	DDMMMYYYY	XXX	XXX	XXX	XXX
XXX	XXX	XXXXXXXX	XXX	XXX	XXX	DDMMMYYYY	XXX	XXX	XXX	XXX
XXX	XXX	XXXXXXXX	XXX	XXX	XXX	DDMMMYYYY	XXX	XXX	XXX	XXX

Source: XXXX.SAS, Run on DDMMMYYYY

Listing 42) Admnistration with Investigational Products

POPULATION: ITT

Treatmen t Arm	Site	Patient Number	Visit	IMP	IMP admini stered ?	If not, reaso n	Date of dispensatio n (DDMMMYYYY)	Start therapy date (DDMMMYYYY)	End therapy date (DDMMMYYYY)	Dos e	Unit	Administrati on	Reason for reduction/del ay/definite interruption	Reduce d dose
XXX	XXX	XXXXXXX X	XXX	XXX	XXX	XXX	DDMMMYYYY	DDMMMYYYY	DDMMMYYYY	XXX	XXX	XXX	XXX	XXX
				XXX	XXX	XXX	DDMMMYYYY	DDMMMYYYY	DDMMMYYYY	XXX	XXX	XXX	XXX	XXX
			XXX	XXX	XXX	XXX	DDMMMYYYY	DDMMMYYYY	DDMMMYYYY	XXX	XXX	XXX	XXX	XXX
				XXX	XXX	XXX	DDMMMYYYY	DDMMMYYYY	DDMMMYYYY	XXX	XXX	XXX	XXX	XXX
			XXX	XXX	XXX	XXX	DDMMMYYYY	DDMMMYYYY	DDMMMYYYY	XXX	XXX	XXX	XXX	XXX
				XXX	XXX	XXX	DDMMMYYYY	DDMMMYYYY	DDMMMYYYY	XXX	XXX	XXX	XXX	XXX
XXX	XXX	XXXXXXX X	XXX	XXX	XXX	XXX	DDMMMYYYY	DDMMMYYYY	DDMMMYYYY	XXX	XXX	XXX	XXX	XXX
XXX	XXX	XXXXXXX X	XXX	XXX	XXX	XXX	DDMMMYYYY	DDMMMYYYY	DDMMMYYYY	XXX	XXX	XXX	XXX	XXX

Source: XXXX.SAS, Run on DDMMMYYYY

Listing 43) LGX818

POPULATION: ITT

Trt Arm	Site	Pt Nr.	Visit	Date of dispensation (DDMMMYYYY)	Batch #1 (50 mg)	Batch #2 (100 mg)	Batch #3 (100 mg)	Batch #4 (100 mg)	Batch #5 (100 mg)	Dose	Taken correctly?	If not, reason	Nr. of capsules returned
XXX	XXX	XXXXXXXX	XXX	DDMMMYYYY	XXX	XXX	XXX	XXX	XXX	XXX	XXX	XXX	XXX
				DDMMMYYYY	XXX	XXX	XXX	XXX	XXX	XXX	XXX	XXX	XXX
			XXX	DDMMMYYYY	XXX	XXX	XXX	XXX	XXX	XXX	XXX	XXX	XXX
				DDMMMYYYY	XXX	XXX	XXX	XXX	XXX	XXX	XXX	XXX	XXX
			XXX	DDMMMYYYY	XXX	XXX	XXX	XXX	XXX	XXX	XXX	XXX	XXX
				DDMMMYYYY	XXX	XXX	XXX	XXX	XXX	XXX	XXX	XXX	XXX
XXX	XXX	XXXXXXXX	XXX	DDMMMYYYY	XXX	XXX	XXX	XXX	XXX	XXX	XXX	XXX	XXX
XXX	XXX	XXXXXXXX	XXX	DDMMMYYYY	XXX	XXX	XXX	XXX	XXX	XXX	XXX	XXX	XXX

Source: XXXX.SAS, Run on DDMMMYYYY

Listing 44) MEK162

POPULATION: ITT

Trt Arm	Site	Pt Nr.	Visit	Date of dispensation (DDMMMYYYY)	Batch #1 (15 mg)	Batch #2 (15 mg)	Batch #3 (15 mg)	Total Daily Dose	Taken correctly?	If not, reason	Nr. of capsules returned	Diary completed correctly and returned?
XXX	XXX	XXXXXXXX	XXX	DDMMMYYYY	XXX	XXX	XXX	XXX	XXX	XXX	XXX	XXX
				DDMMMYYYY	XXX	XXX	XXX	XXX	XXX	XXX	XXX	XXX
			XXX	DDMMMYYYY	XXX	XXX	XXX	XXX	XXX	XXX	XXX	XXX
				DDMMMYYYY	XXX	XXX	XXX	XXX	XXX	XXX	XXX	XXX
			XXX	DDMMMYYYY	XXX	XXX	XXX	XXX	XXX	XXX	XXX	XXX
				DDMMMYYYY	XXX	XXX	XXX	XXX	XXX	XXX	XXX	XXX
XXX	XXX	XXXXXXXX	XXX	DDMMMYYYY	XXX	XXX	XXX	XXX	XXX	XXX	XXX	XXX
XXX	XXX	XXXXXXXX	XXX	DDMMMYYYY	XXX	XXX	XXX	XXX	XXX	XXX	XXX	XXX

Source: XXXX.SAS, Run on DDMMMYYYY

Listing 45) Nivolumab

POPULATION: ITT

Trt Arm	Site	Pt Nr.	Visit	Start therapy date (DDMMMYYYY)	Batch #1	Batch #2)	Dose (mg)	Total volume prepared (ml)	Total Volume infused (ml)	Infusion Start time (hh:mm)	Infusion End time (hh:mm)
XXX	XXX	XXXXXXXX	XXX	DDMMMYYYY	XXX	XXX	XXX	XXX	XXX	XXX	XXX
				DDMMMYYYY	XXX	XXX	XXX	XXX	XXX	XXX	XXX
			XXX	DDMMMYYYY	XXX	XXX	XXX	XXX	XXX	XXX	XXX
				DDMMMYYYY	XXX	XXX	XXX	XXX	XXX	XXX	XXX
			XXX	DDMMMYYYY	XXX	XXX	XXX	XXX	XXX	XXX	XXX
				DDMMMYYYY	XXX	XXX	XXX	XXX	XXX	XXX	XXX
XXX	XXX	XXXXXXXX	XXX	DDMMMYYYY	XXX	XXX	XXX	XXX	XXX	XXX	XXX
XXX	XXX	XXXXXXXX	XXX	DDMMMYYYY	XXX	XXX	XXX	XXX	XXX	XXX	XXX

Source: XXXX.SAS, Run on DDMMMYYYY

Listing 46) Ipilimumab

POPULATION: ITT

Trt Arm	Site	Pt Nr.	Visit	Start therapy date (DDMMMYYYY)	Batch #1	Batch #2)	Dose (mg)	Total volume prepared (ml)	Total Volume infused (ml)	Infusion Start time (hh:mm)	Infusion End time (hh:mm)
XXX	XXX	XXXXXXXX	XXX	DDMMMYYYY	XXX	XXX	XXX	XXX	XXX	XXX	XXX
				DDMMMYYYY	XXX	XXX	XXX	XXX	XXX	XXX	XXX
			XXX	DDMMMYYYY	XXX	XXX	XXX	XXX	XXX	XXX	XXX
				DDMMMYYYY	XXX	XXX	XXX	XXX	XXX	XXX	XXX
			XXX	DDMMMYYYY	XXX	XXX	XXX	XXX	XXX	XXX	XXX
				DDMMMYYYY	XXX	XXX	XXX	XXX	XXX	XXX	XXX
XXX	XXX	XXXXXXXX	XXX	DDMMMYYYY	XXX	XXX	XXX	XXX	XXX	XXX	XXX
XXX	XXX	XXXXXXXX	XXX	DDMMMYYYY	XXX	XXX	XXX	XXX	XXX	XXX	XXX

Source: XXXX.SAS, Run on DDMMMYYYY

Listing 47) Duration of exposure to IMPs and compliance

POPULATION: ITT

Treatment Arm	Site	Patient Number	Visit	Overall duration of exposure to study drugs (weeks)	Duration of exposure to LGX818 (weeks)	Duration of exposure to MEK162 (weeks)	Duration of exposure to Nivolumab (weeks)	Duration of exposure to Ipilimumab (weeks)	Compliance to LGX818 (%)	Compliance to MEK162 (%)
XXX	XXX	XXXXXXXX	XXX	XXX	XXX	XXX	XXX	XXX	XXX	XXX
XXX	XXX	XXXXXXXX	XXX	XXX	XXX	XXX	XXX	XXX	XXX	XXX
XXX	XXX	XXXXXXXX	XXX	XXX	XXX	XXX	XXX	XXX	XXX	XXX
XXX	XXX	XXXXXXXX	XXX	XXX	XXX	XXX	XXX	XXX	XXX	XXX
XXX	XXX	XXXXXXXX	XXX	XXX	XXX	XXX	XXX	XXX	XXX	XXX
XXX	XXX	XXXXXXXX	XXX	XXX	XXX	XXX	XXX	XXX	XXX	XXX
XXX	XXX	XXXXXXXX	XXX	XXX	XXX	XXX	XXX	XXX	XXX	XXX
XXX	XXX	XXXXXXXX	XXX	XXX	XXX	XXX	XXX	XXX	XXX	XXX
XXX	XXX	XXXXXXXX	XXX	XXX	XXX	XXX	XXX	XXX	XXX	XXX

Source: XXXX.SAS, Run on DDMMMYYYY

Listing 48) Tumor assessment: target lesions

POPULATION: ITT

Treatment Arm	Site	Patient Number	TA #	Date of assessment (DDMMMYYYY)	Organ Site	If other, specify	Location of lesion	Method of assessment	Longest diameter (mm)	Shortest diameter (mm)
XXX	XXX	XXXXXXXX	XXX	DDMMMYYYY	XXXXXXXX	XXX	XXX	XXX	XXX	XXX
XXX	XXX	XXXXXXXX	XXX	DDMMMYYYY	XXXXXXXX	XXX	XXX	XXX	XXX	XXX
XXX	XXX	XXXXXXXX	XXX	DDMMMYYYY	XXXXXXXX	XXX	XXX	XXX	XXX	XXX
XXX	XXX	XXXXXXXX	XXX	DDMMMYYYY	XXXXXXXX	XXX	XXX	XXX	XXX	XXX
XXX	XXX	XXXXXXXX	XXX	DDMMMYYYY	XXXXXXXX	XXX	XXX	XXX	XXX	XXX
XXX	XXX	XXXXXXXX	XXX	DDMMMYYYY	XXXXXXXX	XXX	XXX	XXX	XXX	XXX
XXX	XXX	XXXXXXXX	XXX	DDMMMYYYY	XXXXXXXX	XXX	XXX	XXX	XXX	XXX
XXX	XXX	XXXXXXXX	XXX	DDMMMYYYY	XXXXXXXX	XXX	XXX	XXX	XXX	XXX

Source: XXXX.SAS, Run on DDMMMYYYY

Listing 49) Tumor assessment: non-target lesion

POPULATION: ITT

Treatment Arm	Site	Patient Number	TA #	Date of assessment (DDMMMYYYY)	Organ Site	If other, specify	Location of lesion	Method of assessment	If other, specify	Status
XXX	XXX	XXXXXXXX	XXX	DDMMMYYYY	XXXXXXXX	XXX	XXX	XXX	XXX	XXX
XXX	XXX	XXXXXXXX	XXX	DDMMMYYYY	XXXXXXXX	XXX	XXX	XXX	XXX	XXX
XXX	XXX	XXXXXXXX	XXX	DDMMMYYYY	XXXXXXXX	XXX	XXX	XXX	XXX	XXX
XXX	XXX	XXXXXXXX	XXX	DDMMMYYYY	XXXXXXXX	XXX	XXX	XXX	XXX	XXX
XXX	XXX	XXXXXXXX	XXX	DDMMMYYYY	XXXXXXXX	XXX	XXX	XXX	XXX	XXX
XXX	XXX	XXXXXXXX	XXX	DDMMMYYYY	XXXXXXXX	XXX	XXX	XXX	XXX	XXX
XXX	XXX	XXXXXXXX	XXX	DDMMMYYYY	XXXXXXXX	XXX	XXX	XXX	XXX	XXX
XXX	XXX	XXXXXXXX	XXX	DDMMMYYYY	XXXXXXXX	XXX	XXX	XXX	XXX	XXX

Source: XXXX.SAS, Run on DDMMMYYYY

Listing 50) Tumor assessment: tumor response according to RECIST criteria v.1.1

POPULATION: ITT

Treatment Arm	Site	Patient Number	Visit	Date of assessment (DDMMMYYYY)	Sum of Target lesions (mm)	Target lesions	Non-target lesions	New lesions	Overall response
XXX	XXX	XXXXXXXX	XXXXXXXX	DDMMMYYYY	XXX	XXX	XXX	XXX	XXX
			XXXXXXXX	DDMMMYYYY	XXX	XXX	XXX	XXX	XXX
			XXXXXXXX	DDMMMYYYY	XXX	XXX	XXX	XXX	XXX
			XXXXXXXX	DDMMMYYYY	XXX	XXX	XXX	XXX	XXX
			XXXXXXXX	DDMMMYYYY	XXX	XXX	XXX	XXX	XXX
XXX	XXX	XXXXXXXX	XXXXXXXX	DDMMMYYYY	XXX	XXX	XXX	XXX	XXX
XXX	XXX	XXXXXXXX	XXXXXXXX	DDMMMYYYY	XXX	XXX	XXX	XXX	XXX
XXX	XXX	XXXXXXXX	XXXXXXXX	DDMMMYYYY	XXX	XXX	XXX	XXX	XXX

Source: XXXX.SAS, Run on DDMMMYYYY

Listing 51) Overall survival

POPULATION: ITT

Treatment Arm	Site	Patient Number	Date of randomization (DDMMMYYYY)	Date of death from any cause / last contact date (DDMMMYYYY)	Censoring (0=censored, 1=event)	OS (months)	Alive at 2 years (Yes/No)	Alive at 3 years (Yes/No)
XXX	XXX	XXXXXXXX	DDMMMYYYY	DDMMMYYYY	X	XXX	XXX	XXX
XXX	XXX	XXXXXXXX	DDMMMYYYY	DDMMMYYYY	X	XXX	XXX	XXX
XXX	XXX	XXXXXXXX	DDMMMYYYY	DDMMMYYYY	X	XXX	XXX	XXX
XXX	XXX	XXXXXXXX	DDMMMYYYY	DDMMMYYYY	X	XXX	XXX	XXX
XXX	XXX	XXXXXXXX	DDMMMYYYY	DDMMMYYYY	X	XXX	XXX	XXX
XXX	XXX	XXXXXXXX	DDMMMYYYY	DDMMMYYYY	X	XXX	XXX	XXX
XXX	XXX	XXXXXXXX	DDMMMYYYY	DDMMMYYYY	X	XXX	XXX	XXX
XXX	XXX	XXXXXXXX	DDMMMYYYY	DDMMMYYYY	X	XXX	XXX	XXX

Source: XXXX.SAS, Run on DDMMMYYYY

Listing 52) Total progression free survival, 3-years PFS and BORR

POPULATION: ITT

Treatment Arm	Site	Patient Number	Date of randomization (DDMMMYYYY)	Date of second progression / last contact date (DDMMMYYYY)	Censoring (0=censored, 1=event)	Total PFS (months)	3-years PFS (Yes/No)	BORR
XXX	XXX	XXXXXXXX	DDMMMYYYY	DDMMMYYYY	X	XXX	XXX	XXX
XXX	XXX	XXXXXXXX	DDMMMYYYY	DDMMMYYYY	X	XXX	XXX	XXX
XXX	XXX	XXXXXXXX	DDMMMYYYY	DDMMMYYYY	X	XXX	XXX	XXX
XXX	XXX	XXXXXXXX	DDMMMYYYY	DDMMMYYYY	X	XXX	XXX	XXX
XXX	XXX	XXXXXXXX	DDMMMYYYY	DDMMMYYYY	X	XXX	XXX	XXX
XXX	XXX	XXXXXXXX	DDMMMYYYY	DDMMMYYYY	X	XXX	XXX	XXX
XXX	XXX	XXXXXXXX	DDMMMYYYY	DDMMMYYYY	X	XXX	XXX	XXX
XXX	XXX	XXXXXXXX	DDMMMYYYY	DDMMMYYYY	X	XXX	XXX	XXX

Source: XXXX.SAS, Run on DDMMMYYYY

Listing 53) Duration of Response (DoR)

POPULATION: ITT

Treatment Arm	Site	Patient Number	Date of first documented response (CR or PR) (DDMMMYYYY)	Date of first progression or death du to cancer / last tumor assessment date (DDMMMYYYY)	Censoring (0=censored, 1=event)	DoR (months)
XXX	XXX	XXXXXXXX	DDMMMYYYY	DDMMMYYYY	X	XXX
XXX	XXX	XXXXXXXX	DDMMMYYYY	DDMMMYYYY	X	XXX
XXX	XXX	XXXXXXXX	DDMMMYYYY	DDMMMYYYY	X	XXX
XXX	XXX	XXXXXXXX	DDMMMYYYY	DDMMMYYYY	X	XXX
XXX	XXX	XXXXXXXX	DDMMMYYYY	DDMMMYYYY	X	XXX
XXX	XXX	XXXXXXXX	DDMMMYYYY	DDMMMYYYY	X	XXX
XXX	XXX	XXXXXXXX	DDMMMYYYY	DDMMMYYYY	X	XXX
XXX	XXX	XXXXXXXX	DDMMMYYYY	DDMMMYYYY	X	XXX

Source: XXXX.SAS, Run on DDMMMYYYY

Listing 54) Adverse events

POPULATION: SAF

Patient Number	AE#	Date on which the investigator finds out of the event (DDMMMYYYY)	Description of event	Preferred Term	SOC	Start Date (DDMMMYYYY)/ End Date (DDMMMYYYY)	Causality	Ongoing?	Related to	SAE	AESI	Grade	Outcome	Action taken	Trt. Req.?
XXXXX	X	DDMONYYYY	XXXXXX	XXXXXXXX	XXXXXXXX	DDMONYYYY / DDMONYYYY	XXXXXX	XXXXXX	XXXXXX	X	X	xxx	xxx	xxx	xxx
	X	DDMONYYYY	XXXXXX	XXXXXXXX	XXXXXXXX	DDMONYYYY / DDMONYYYY	XXXXXX	XXXXXX	XXXXXX	X	X	xxx	xxx	xxx	xxx
	X	DDMONYYYY	XXXXXX	XXXXXXXX	XXXXXXXX	DDMONYYYY / DDMONYYYY	XXXXXX	XXXXXX	XXXXXX	X	X	xxx	xxx	xxx	xxx
	X	DDMONYYYY	XXXXXX	XXXXXXXX	XXXXXXXX	DDMONYYYY / DDMONYYYY	XXXXXX	XXXXXX	XXXXXX	X	X	xxx	xxx	xxx	xxx
XXXX	X	DDMONYYYY	XXXXXX	XXXXXXXX	XXXXXXXX	DDMONYYYY / DDMONYYYY	XXXXXX	XXXXXX	XXXXXX	X	X	xxx	xxx	xxx	xxx
	X	DDMONYYYY	XXXXXX	XXXXXXXX	XXXXXXXX	DDMONYYYY / DDMONYYYY	XXXXXX	XXXXXX	XXXXXX	X	X	xxx	xxx	xxx	xxx

Source: XXXX.SAS, Run on DDMMMYYYY

Listing 55) Adverse events related to Ipilimumab

POPULATION: SAF

Patient Number	AE #	Date on which the investigator finds out of the event (DDMMMYYYY)	Description of event	Preferred Term	SOC	Start Date (DDMMMYYYY) / End Date (DDMMMYYYY)	Causality	Ongoing?	Related to	SAE	AESI	Grade	Outcome	Action taken	Trt. Req.?
XXXXX	X	DDMONYYYY	XXXXXX	XXXXXXXX	XXXXXXXX	DDMONYYYY / DDMONYYYY	XXXXXX	XXXXXX	XXXXXX	X	X	xxx	xxx	xxx	xxx
	X	DDMONYYYY	XXXXXX	XXXXXXXX	XXXXXXXX	DDMONYYYY / DDMONYYYY	XXXXXX	XXXXXX	XXXXXX	X	X	xxx	xxx	xxx	xxx
	X	DDMONYYYY	XXXXXX	XXXXXXXX	XXXXXXXX	DDMONYYYY / DDMONYYYY	XXXXXX	XXXXXX	XXXXXX	X	X	xxx	xxx	xxx	xxx
	X	DDMONYYYY	XXXXXX	XXXXXXXX	XXXXXXXX	DDMONYYYY / DDMONYYYY	XXXXXX	XXXXXX	XXXXXX	X	X	xxx	xxx	xxx	xxx
XXXX	X	DDMONYYYY	XXXXXX	XXXXXXXX	XXXXXXXX	DDMONYYYY / DDMONYYYY	XXXXXX	XXXXXX	XXXXXX	X	X	xxx	xxx	xxx	xxx
	X	DDMONYYYY	XXXXXX	XXXXXXXX	XXXXXXXX	DDMONYYYY / DDMONYYYY	XXXXXX	XXXXXX	XXXXXX	X	X	xxx	xxx	xxx	xxx

Source: XXXX.SAS, Run on DDMMMYYYY

Listing 56) Adverse events related to Nivolumab

POPULATION: SAF

Patient Number	AE #	Date on which the investigator finds out of the event (DDMMMYYYY)	Description of event	Preferred Term	SOC	Start Date (DDMMMYYYY) / End Date (DDMMMYYYY)	Causality	Ongoing?	Related to	SAE	AESI	Grade	Outcome	Action taken	Trt. Req.?
XXXXX	X	DDMONYYYY	XXXXXX	XXXXXXXX	XXXXXXXX	DDMONYYYY / DDMONYYYY	XXXXXX	XXXXXX	XXXXXX	X	X	xxx	xxx	xxx	xxx
	X	DDMONYYYY	XXXXXX	XXXXXXXX	XXXXXXXX	DDMONYYYY / DDMONYYYY	XXXXXX	XXXXXX	XXXXXX	X	X	xxx	xxx	xxx	xxx
	X	DDMONYYYY	XXXXXX	XXXXXXXX	XXXXXXXX	DDMONYYYY / DDMONYYYY	XXXXXX	XXXXXX	XXXXXX	X	X	xxx	xxx	xxx	xxx
	X	DDMONYYYY	XXXXXX	XXXXXXXX	XXXXXXXX	DDMONYYYY / DDMONYYYY	XXXXXX	XXXXXX	XXXXXX	X	X	xxx	xxx	xxx	xxx
XXXX	X	DDMONYYYY	XXXXXX	XXXXXXXX	XXXXXXXX	DDMONYYYY / DDMONYYYY	XXXXXX	XXXXXX	XXXXXX	X	X	xxx	xxx	xxx	xxx
	X	DDMONYYYY	XXXXXX	XXXXXXXX	XXXXXXXX	DDMONYYYY / DDMONYYYY	XXXXXX	XXXXXX	XXXXXX	X	X	xxx	xxx	xxx	xxx

Source: XXXX.SAS, Run on DDMMMYYYY

Listing 57) Adverse events related to Combo Immuno Therapy

POPULATION: SAF

Patient Number	AE #	Date on which the investigator finds out of the event (DDMMMYYYY)	Description of event	Preferred Term	SOC	Start Date (DDMMMYYYY) / End Date (DDMMMYYYY)	Causality	Ongoing ?	Related to	SAE	AESI	Grade	Outcome	Action taken	Trt. Req.?
XXXXX	X	DDMONYYYY	XXXXXX	XXXXXXXX	XXXXXXXX	DDMONYYYY / DDMONYYYY	XXXXXX	XXXXXX	XXXXXX	X	X	xxx	xxx	xxx	xxx
	X	DDMONYYYY	XXXXXX	XXXXXXXX	XXXXXXXX	DDMONYYYY / DDMONYYYY	XXXXXX	XXXXXX	XXXXXX	X	X	xxx	xxx	xxx	xxx
	X	DDMONYYYY	XXXXXX	XXXXXXXX	XXXXXXXX	DDMONYYYY / DDMONYYYY	XXXXXX	XXXXXX	XXXXXX	X	X	xxx	xxx	xxx	xxx
	X	DDMONYYYY	XXXXXX	XXXXXXXX	XXXXXXXX	DDMONYYYY / DDMONYYYY	XXXXXX	XXXXXX	XXXXXX	X	X	xxx	xxx	xxx	xxx
XXXX	X	DDMONYYYY	XXXXXX	XXXXXXXX	XXXXXXXX	DDMONYYYY / DDMONYYYY	XXXXXX	XXXXXX	XXXXXX	X	X	xxx	xxx	xxx	xxx
	X	DDMONYYYY	XXXXXX	XXXXXXXX	XXXXXXXX	DDMONYYYY / DDMONYYYY	XXXXXX	XXXXXX	XXXXXX	X	X	xxx	xxx	xxx	xxx

Source: XXXX.SAS, Run on DDMMMYYYY

Listing 58) Adverse events related to LGX818

POPULATION: SAF

Patient Number	AE#	Date on which the investigator finds out of the event (DDMMMYYYY)	Description of event	Preferred Term	SOC	Start Date (DDMMMYYYY) / End Date (DDMMMYYYY)	Causality	Ongoing?	Related to	SAE	AESI	Grade	Outcome	Action taken	Trt. Req.?
XXXXX	X	DDMONYYYY	XXXXXX	XXXXXXXX	XXXXXXXX	DDMONYYYY / DDMONYYYY	XXXXXX	XXXXXX	XXXXXX	X	X	xxx	xxx	xxx	xxx
	X	DDMONYYYY	XXXXXX	XXXXXXXX	XXXXXXXX	DDMONYYYY / DDMONYYYY	XXXXXX	XXXXXX	XXXXXX	X	X	xxx	xxx	xxx	xxx
	X	DDMONYYYY	XXXXXX	XXXXXXXX	XXXXXXXX	DDMONYYYY / DDMONYYYY	XXXXXX	XXXXXX	XXXXXX	X	X	xxx	xxx	xxx	xxx
	X	DDMONYYYY	XXXXXX	XXXXXXXX	XXXXXXXX	DDMONYYYY / DDMONYYYY	XXXXXX	XXXXXX	XXXXXX	X	X	xxx	xxx	xxx	xxx
XXXX	X	DDMONYYYY	XXXXXX	XXXXXXXX	XXXXXXXX	DDMONYYYY / DDMONYYYY	XXXXXX	XXXXXX	XXXXXX	X	X	xxx	xxx	xxx	xxx
	X	DDMONYYYY	XXXXXX	XXXXXXXX	XXXXXXXX	DDMONYYYY / DDMONYYYY	XXXXXX	XXXXXX	XXXXXX	X	X	xxx	xxx	xxx	xxx

Source: XXXX.SAS, Run on DDMMMYYYY

Listing 59) Adverse events related to MEK162

POPULATION: SAF

Patient Number	AE #	Date on which the investigator finds out of the event (DDMMMYYYY)	Description of event	Preferred Term	SOC	Start Date (DDMMMYYYY) / End Date (DDMMMYYYY)	Causality	Ongoing ?	Related to	SAE	AESI	Grade	Outcome	Action taken	Trt. Req.?
XXXXX	X	DDMONYYYY	XXXXXX	XXXXXXXX	XXXXXXXX	DDMONYYYY / DDMONYYYY	XXXXXX	XXXXXX	XXXXXX	X	X	xxx	xxx	xxx	xxx
	X	DDMONYYYY	XXXXXX	XXXXXXXX	XXXXXXXX	DDMONYYYY / DDMONYYYY	XXXXXX	XXXXXX	XXXXXX	X	X	xxx	xxx	xxx	xxx
	X	DDMONYYYY	XXXXXX	XXXXXXXX	XXXXXXXX	DDMONYYYY / DDMONYYYY	XXXXXX	XXXXXX	XXXXXX	X	X	xxx	xxx	xxx	xxx
	X	DDMONYYYY	XXXXXX	XXXXXXXX	XXXXXXXX	DDMONYYYY / DDMONYYYY	XXXXXX	XXXXXX	XXXXXX	X	X	xxx	xxx	xxx	xxx
XXXX	X	DDMONYYYY	XXXXXX	XXXXXXXX	XXXXXXXX	DDMONYYYY / DDMONYYYY	XXXXXX	XXXXXX	XXXXXX	X	X	xxx	xxx	xxx	xxx
	X	DDMONYYYY	XXXXXX	XXXXXXXX	XXXXXXXX	DDMONYYYY / DDMONYYYY	XXXXXX	XXXXXX	XXXXXX	X	X	xxx	xxx	xxx	xxx

Source: XXXX.SAS, Run on DDMMMYYYY

Listing 60) Adverse events related to Combo Target Therapy

POPULATION: SAF

Patient Number	AE #	Date on which the investigator finds out of the event (DDMMMYYYY)	Description of event	Preferred Term	SOC	Start Date (DDMMMYYYY) / End Date (DDMMMYYYY)	Causality	Ongoing ?	Related to	SAE	AESI	Grade	Outcome	Action taken	Trt. Req. ?
XXXXX	X	DDMONYYYY	XXXXXX	XXXXXXXX	XXXXX	DDMONYYYY / DDMONYYYY	XXXXXX	XXXXXX	XXXXXX	X	X	xxx	xxx	xxx	xxx
	X	DDMONYYYY	XXXXXX	XXXXXXXX	XXXXX	DDMONYYYY / DDMONYYYY	XXXXXX	XXXXXX	XXXXXX	X	X	xxx	xxx	xxx	xxx
	X	DDMONYYYY	XXXXXX	XXXXXXXX	XXXXX	DDMONYYYY / DDMONYYYY	XXXXXX	XXXXXX	XXXXXX	X	X	xxx	xxx	xxx	xxx
	X	DDMONYYYY	XXXXXX	XXXXXXXX	XXXXX	DDMONYYYY / DDMONYYYY	XXXXXX	XXXXXX	XXXXXX	X	X	xxx	xxx	xxx	xxx
XXXX	X	DDMONYYYY	XXXXXX	XXXXXXXX	XXXXX	DDMONYYYY / DDMONYYYY	XXXXXX	XXXXXX	XXXXXX	X	X	xxx	xxx	xxx	xxx
	X	DDMONYYYY	XXXXXX	XXXXXXXX	XXXXX	DDMONYYYY / DDMONYYYY	XXXXXX	XXXXXX	XXXXXX	X	X	xxx	xxx	xxx	xxx

Source: XXXX.SAS, Run on DDMMMYYYY

Listing 61) Serious Adverse events

POPULATION: SAF

Patient Number	AE #	Date on which the investigator finds out of the event (DDMMMYYYY)	Description of event	Preferred Term	SOC	Start Date (DDMMMYYYY) / End Date (DDMMMYYYY)	Causality	Ongoing ?	Related to	SAE	AESI	Grade	Outcome	Action taken	Trt. Req.?
XXXXX	X	DDMONYYYY	XXXXXX	XXXXXXXX	XXXXXXXX	DDMONYYYY / DDMONYYYY	XXXXXX	XXXXXX	XXXXXX	X	X	xxx	xxx	xxx	xxx
	X	DDMONYYYY	XXXXXX	XXXXXXXX	XXXXXXXX	DDMONYYYY / DDMONYYYY	XXXXXX	XXXXXX	XXXXXX	X	X	xxx	xxx	xxx	xxx
	X	DDMONYYYY	XXXXXX	XXXXXXXX	XXXXXXXX	DDMONYYYY / DDMONYYYY	XXXXXX	XXXXXX	XXXXXX	X	X	xxx	xxx	xxx	xxx
	X	DDMONYYYY	XXXXXX	XXXXXXXX	XXXXXXXX	DDMONYYYY / DDMONYYYY	XXXXXX	XXXXXX	XXXXXX	X	X	xxx	xxx	xxx	xxx
XXXX	X	DDMONYYYY	XXXXXX	XXXXXXXX	XXXXXXXX	DDMONYYYY / DDMONYYYY	XXXXXX	XXXXXX	XXXXXX	X	X	xxx	xxx	xxx	xxx
	X	DDMONYYYY	XXXXXX	XXXXXXXX	XXXXXXXX	DDMONYYYY / DDMONYYYY	XXXXXX	XXXXXX	XXXXXX	X	X	xxx	xxx	xxx	xxx

Source: XXXX.SAS, Run on DDMMMYYYY

Listing 62) Adverse events leading to death

POPULATION: SAF

Patient Number	AE #	Date on which the investigator finds out of the event (DDMMMYYYY)	Description of event	Preferred Term	SOC	Start Date (DDMMMYYYY) / End Date (DDMMMYYYY)	Causality	Ongoing?	Related to	SAE	AESI	Grade	Outcome	Action taken	Trt. Req.?
XXXXX	X	DDMONYYYY	XXXXXX	XXXXXXXX	XXXXXXXX	DDMONYYYY / DDMONYYYY	XXXXXX	XXXXXX	XXXXXX	X	X	xxx	xxx	xxx	xxx
	X	DDMONYYYY	XXXXXX	XXXXXXXX	XXXXXXXX	DDMONYYYY / DDMONYYYY	XXXXXX	XXXXXX	XXXXXX	X	X	xxx	xxx	xxx	xxx
	X	DDMONYYYY	XXXXXX	XXXXXXXX	XXXXXXXX	DDMONYYYY / DDMONYYYY	XXXXXX	XXXXXX	XXXXXX	X	X	xxx	xxx	xxx	xxx
	X	DDMONYYYY	XXXXXX	XXXXXXXX	XXXXXXXX	DDMONYYYY / DDMONYYYY	XXXXXX	XXXXXX	XXXXXX	X	X	xxx	xxx	xxx	xxx
XXXX	X	DDMONYYYY	XXXXXX	XXXXXXXX	XXXXXXXX	DDMONYYYY / DDMONYYYY	XXXXXX	XXXXXX	XXXXXX	X	X	xxx	xxx	xxx	xxx
	X	DDMONYYYY	XXXXXX	XXXXXXXX	XXXXXXXX	DDMONYYYY / DDMONYYYY	XXXXXX	XXXXXX	XXXXXX	X	X	xxx	xxx	xxx	xxx

Source: XXXX.SAS, Run on DDMMMYYYY

Listing 63) Adverse events leading to hospitalization

POPULATION: SAF

Patient Number	AE#	Date on which the investigator finds out of the event (DDMMMYYYY)	Description of event	Preferred Term	SOC	Start Date (DDMMMYYYY) / End Date (DDMMMYYYY)	Causality	Ongoing?	Related to	SAE	AESI	Grade	Outcome	Action taken	Trt. Req.?
XXXXX	X	DDMONYYYY	XXXXXX	XXXXXXXX	XXXXXXXX	DDMONYYYY / DDMONYYYY	XXXXXX	XXXXXX	XXXXXX	X	X	xxx	xxx	xxx	xxx
	X	DDMONYYYY	XXXXXX	XXXXXXXX	XXXXXXXX	DDMONYYYY / DDMONYYYY	XXXXXX	XXXXXX	XXXXXX	X	X	xxx	xxx	xxx	xxx
	X	DDMONYYYY	XXXXXX	XXXXXXXX	XXXXXXXX	DDMONYYYY / DDMONYYYY	XXXXXX	XXXXXX	XXXXXX	X	X	xxx	xxx	xxx	xxx
	X	DDMONYYYY	XXXXXX	XXXXXXXX	XXXXXXXX	DDMONYYYY / DDMONYYYY	XXXXXX	XXXXXX	XXXXXX	X	X	xxx	xxx	xxx	xxx
XXXX	X	DDMONYYYY	XXXXXX	XXXXXXXX	XXXXXXXX	DDMONYYYY / DDMONYYYY	XXXXXX	XXXXXX	XXXXXX	X	X	xxx	xxx	xxx	xxx
	X	DDMONYYYY	XXXXXX	XXXXXXXX	XXXXXXXX	DDMONYYYY / DDMONYYYY	XXXXXX	XXXXXX	XXXXXX	X	X	xxx	xxx	xxx	xxx

Source: XXXX.SAS, Run on DDMMMYYYY

Listing 64) Adverse events leading to study withdrawal

POPULATION: SAF

Patient Number	AE #	Date on which the investigator finds out of the event (DDMMMYYYY)	Description of event	Preferred Term	SOC	Start Date (DDMMMYYYY) / End Date (DDMMMYYYY)	Causality	Ongoing ?	Related to	SAE	AESI	Grade	Outcome	Action taken	Trt. Req.?
XXXXX	X	DDMONYYYY	XXXXXX	XXXXXXXX	XXXXXXXX	DDMONYYYY / DDMONYYYY	XXXXXX	XXXXXX	XXXXXX	X	X	xxx	xxx	xxx	xxx
	X	DDMONYYYY	XXXXXX	XXXXXXXX	XXXXXXXX	DDMONYYYY / DDMONYYYY	XXXXXX	XXXXXX	XXXXXX	X	X	xxx	xxx	xxx	xxx
	X	DDMONYYYY	XXXXXX	XXXXXXXX	XXXXXXXX	DDMONYYYY / DDMONYYYY	XXXXXX	XXXXXX	XXXXXX	X	X	xxx	xxx	xxx	xxx
	X	DDMONYYYY	XXXXXX	XXXXXXXX	XXXXXXXX	DDMONYYYY / DDMONYYYY	XXXXXX	XXXXXX	XXXXXX	X	X	xxx	xxx	xxx	xxx
XXXX	X	DDMONYYYY	XXXXXX	XXXXXXXX	XXXXXXXX	DDMONYYYY / DDMONYYYY	XXXXXX	XXXXXX	XXXXXX	X	X	xxx	xxx	xxx	xxx
	X	DDMONYYYY	XXXXXX	XXXXXXXX	XXXXXXXX	DDMONYYYY / DDMONYYYY	XXXXXX	XXXXXX	XXXXXX	X	X	xxx	xxx	xxx	xxx

Source: XXXX.SAS, Run on DDMMMYYYY

Listing 65) Adverse events of special interest (AESI)

POPULATION: SAF

Patient Number	AE #	Date on which the investigator finds out of the event (DDMMMYYYY)	Description of event	Preferred Term	SOC	Start Date (DDMMMYYYY) / End Date (DDMMMYYYY)	Causality	Ongoing?	Related to	SAE	AESI	Grade	Outcome	Action taken	Trt. Req.?
XXXXX	X	DDMONYYYY	XXXXXX	XXXXXXXX	XXXXXXXX	DDMONYYYY / DDMONYYYY	XXXXXX	XXXXXX	XXXXXX	X	X	xxx	xxx	xxx	xxx
	X	DDMONYYYY	XXXXXX	XXXXXXXX	XXXXXXXX	DDMONYYYY / DDMONYYYY	XXXXXX	XXXXXX	XXXXXX	X	X	xxx	xxx	xxx	xxx
	X	DDMONYYYY	XXXXXX	XXXXXXXX	XXXXXXXX	DDMONYYYY / DDMONYYYY	XXXXXX	XXXXXX	XXXXXX	X	X	xxx	xxx	xxx	xxx
	X	DDMONYYYY	XXXXXX	XXXXXXXX	XXXXXXXX	DDMONYYYY / DDMONYYYY	XXXXXX	XXXXXX	XXXXXX	X	X	xxx	xxx	xxx	xxx
XXXX	X	DDMONYYYY	XXXXXX	XXXXXXXX	XXXXXXXX	DDMONYYYY / DDMONYYYY	XXXXXX	XXXXXX	XXXXXX	X	X	xxx	xxx	xxx	xxx
	X	DDMONYYYY	XXXXXX	XXXXXXXX	XXXXXXXX	DDMONYYYY / DDMONYYYY	XXXXXX	XXXXXX	XXXXXX	X	X	xxx	xxx	xxx	xxx

Source: XXXX.SAS, Run on DDMMMYYYY

Listing 66) Prior Medications

POPULATION: ITT

Site	Patient Number	CM (#)	Drug	Generic name	3rd level ATC subgroup	Dose	Unit	Start Date (DDMMMYYYY)	End Date (DDMMMYYYY)	Ongoing?	Route	Frequency	Indication	AE #
XXX	XXXXXXXX	XXX	XXXXXXXX	XXXX	XXX	XXX	XXX	DDMMMYYYY	DDMMMYYYY	XXX	XXX	XXX	XXX	XXX
XXX	XXXXXXXX	XXX	XXXXXXXX	XXXX	XXX	XXX	XXX	DDMMMYYYY	DDMMMYYYY	XXX	XXX	XXX	XXX	XXX
XXX	XXXXXXXX	XXX	XXXXXXXX	XXXX	XXX	XXX	XXX	DDMMMYYYY	DDMMMYYYY	XXX	XXX	XXX	XXX	XXX
XXX	XXXXXXXX	XXX	XXXXXXXX	XXXX	XXX	XXX	XXX	DDMMMYYYY	DDMMMYYYY	XXX	XXX	XXX	XXX	XXX

Notes:
Prior Medications were coded using the WHO-DRL Version XXXX.

CD = Drug prescribed for concomitant disease, P = Drugs administered prophylactically, AEn = Number of corresponding AE.
Prior medications are those which stopped prior to the first dose of study medication.

Source: XXXX.SAS, Run on DDMMMYYYY

Listing 67) Concomitant Medications

POPULATION: ITT

Site	Patient Number	CM (#)	Drug	Generic name	3rd level ATC subgroup	Dose	Unit	Start Date (DDMMMYYYY)	End Date (DDMMMYYYY)	Ongoing?	Route	Frequ ency	Indica tion	AE #
XXX	XXXXXXXX	XXX	XXXXXXXX	XXXX	XXX	XXX	XXX	DDMMMYYYY	DDMMMYYYY	XXX	XXX	XXX	XXX	XXX
XXX	XXXXXXXX	XXX	XXXXXXXX	XXXX	XXX	XXX	XXX	DDMMMYYYY	DDMMMYYYY	XXX	XXX	XXX	XXX	XXX
XXX	XXXXXXXX	XXX	XXXXXXXX	XXXX	XXX	XXX	XXX	DDMMMYYYY	DDMMMYYYY	XXX	XXX	XXX	XXX	XXX
XXX	XXXXXXXX	XXX	XXXXXXXX	XXXX	XXX	XXX	XXX	DDMMMYYYY	DDMMMYYYY	XXX	XXX	XXX	XXX	XXX

Notes:
Concomitant Medications were coded using the WHO-DRL Version XXXX.
CD = Drug prescribed for concomitant disease, P = Drugs administered prophylactically, AEn = Number of corresponding AE.
Concomitant Medications are those medications which:
started prior to, on or after the first dose of study medication and started no later than date of last study dose,
AND
ended on or after the date of first dose of study medication or were ongoing at the end of the study.

Source: XXXX.SAS, Run on DDMMMYYYY

Study Code: SECOMBIT
EudraCT Number: 2014-004842-92
Study sponsor: Fondazione Melanoma ONLUS
Investigational Products: LGX818/MEK162/Nivolumab/Ipilimumab
Study title:" Sequential Combo Immuno and Target therapy (SECOMBIT) study"
"A three arms prospective, randomized phase II study to evaluate the best sequential approach with combo immunotherapy (ipilimumab/nivolumab) and combo target therapy (LGX818/MEK162) in patients with metastatic melanoma and BRAF mutation"

Re: Summary of changes – Study protocol from v 1.0 dated 22 Oct 2015 to version 10.0 dated 03 May 2021.

Salerno, 03 May 2021

SECTIONS UPDATED –PROTOCOL VERSION 2.0

Section	page	Description of change	Reason for change
Synopsis<br>Section 6.3	p. 15<br>p. 76	Milestones of the study postponed	Timeline adjusted according to regulatory submission timeline
Section 3.5.3	p. 40	HIV test added to screening procedures	Italian CA request

SECTIONS UPDATED –PROTOCOL VERSION 3.0

Section	page	Description of change	Reason for change
Synopsis	p. 8	Number of sites incresed from 20 to 30	Sponsor's decision in order to meet study timelines
Synopsis (Study plan)	p. 9	Strata were reduce from 4 to 3, matching LDH range values were fixed	Steering Committee request
Synopsis (Study plan)	p. 9	The following sentences have been added:<br>Tumor assessments including measurable and non-measurable lesions (baseline brain CT or MRI, CT/MRI C/A/P, bone scan if clinically indicated).	Clarifications

Clinical Research Technology S.r.l.
www.cr-technology.com
info@cr-technology.com

Section	page	Description of change	Reason for change
		Follow up visit is to be performed within 28 days from discontinuation of treatments and thereafter every 12 weeks until 24 months for long term survival follow-up	
Synopsis (Study treatment duration, dose and schedule)<br><br>Section 3.4	p. 12,13<br><br>p. 39	The following sentences have been added:<br><br>If the AE does not resolve or decrease to at least grade 1, during the second screening period (28 days), patient should be followed up for additional 28 days. All screening procedures must be repeated before starting the new combination therapy (tumor assessment, to exclude progression disease, included). If AE does not resolve or decrease to al least grade 1, during this additional period (until 56 days after PD1), patient will be permanently discontinued<br><br>Patients in the ARM C, with a progression disease documented at the first tumor evaluation, will be discontinued from the study and will be treated as per institutional standard of care thereafter.<br><br>Patients who discontinue from the study will be asked to return to the clinic within 28 days of the last dose of IMP for the follow-up visit and to be contacted every 12 weeks until 24 months for long term survival follow-up.<br><br>Hematology and biochemistry	Clarifications

Section	page	Description of change	Reason for change
		assessments do not need to be repeated on Day 1 if performed within 7 days; if it is necessary to repeat these blood tests, the results must be known before the patient receives treatments to ensure inclusion/exclusion criteria related to these tests are met. Test for HIV infection is mandatory at screening.	
Synopsis (Secondary Endpoints)	p. 13	A new secondary endpoint has been added (percentage of PFS after 3 years from randomization);	Steering Committee request
Synopsis<br>Section 6.3	p. 15<br>p. 76	Timelines of the study postponed	Timeline adjusted according to regulatory submission timeline
Section 3.5.3	p. 41,42, 43, 44	Vital signs have been specified as follows: respiratory rate, pulse, blood pressure and temperature will be obtained in the same position, as appropriate prior to any blood collection. Two readings of supine blood pressure, in the same arm, separated by 2 min should be recorded in the Source Documents and the mean of the two consecutive readings, should be recorded in the eCRF throughout the study	Clarification
Section 3.5.3<br>Section 3.5.8<br>Section 7.5.3	p. 44<br>p 53<br>p. 88	A serum pregnancy test to be performed every 6 (± 1) weeks during the treatment period and at end of study treatment and follow-up visit up to 31 weeks after the last dose of nivolumab/ipilimumab	Clarification
Section 3.5.3	p. 45	Procedures related to Long term follow up visit (to be performed every 12 weeks until 24 months)	Clarification

Clinical Research Technology S.r.l.
www.cr-technology.com
info@cr-technology.com

CLINICAL RESEARCH TECHNOLOGY

Section	page	Description of change	Reason for change
		have been detailed	
Section 3.5.4 and 3.5.5	p. 48, 49	It has been clarified biological samples collection timeline	Clarification
Section 6.2.2	p. 78	The definition of the Per Protocol Population has been added	Addition
Section 7.5.2	p. 87	Emergency contact updated	Update
Several sections	Several pages	Deletions and retouches	Text Hamonization and minor corrections

SECTIONS UPDATED - PROTOCOL VERSION 4.0

Section	page	Description of change	Reason for change
Synopsis (Study plan)	p. 10	CT/MRI of brain as clinically indicated has been added.	Clinical rationale
Synopsis (inclusion criteria)<br>Section 4.1	p. 10<br>p. 54	Incl #3 retouched to:<br>Treatment naïve for metastatic disease patients. Previous adjuvant treatment, included checkpoint inhibitors anti CTLA-4, anti PD-1/PDL-1 is allowed. (if completed at least 6 weeks prior to randomization, and all related adverse events have either returned to baseline or stabilized). BRAF inhibitor treatment in adjuvant setting is not permitted	Clinical rationale
Section 2.4	p. 32	Final Risk/Benefit Statement section added	Addition
Section 3.5.0	p. 40	Subject Re-enrollment: This study permits the re-enrollment of patients that was not randomized within 28 from ICF signature, after	Clarification

Section	page	Description of change	Reason for change
		obtaining agreement from the medical monitor prior to re--enrolling a subject. If authorized to re-enrollment, the patient must be re-consented and the same subject-code can be used.	
Section 3.5.3<br><br>Section 3.5.8	p. 41, 42, 43, 44, 45, 46<br>p.53	Protocol Procedured aligned with the schedule of assessments	Adjustments and clarifications
Section 5.1.2<br>Section 5.2.2	p. 69<br>p. 74	The following sentences have been added: Patients who discontinued permanently the LGX818/MEK162 treatment can be followed up until progression disease and remain in the SECOMBIT study unless in the judgment of the Investigator, presents a substantial clinical risk to the subject with the study treatments.<br><br>Patients who discontinued permanently the nivolumab/ipilimumab treatment can be followed up until progression disease and remain in the SECOMBIT study unless in the judgment of the Investigator, presents a substantial clinical risk to the subject with the study treatments.	Clinical rationale
Section 10	p. 93	The section “Protocol adherence” has been reworded	Clarifications
Several sections	Several pages	Deletions and retouches	Text Hamonization and minor corrections

SECTIONS UPDATED - PROTOCOL VERSION 5.0

The main reason for this protocol amendment was to update the safety risk section, better describe protocol procedures and correct typing errors.

**Section**	**page**	**Description of change**	**Reason for change**
Synopsis<br>Number of participant centers	p. 3	Study Coordinator has been substituted with Global Chief Investigator.	Clarification
Synopsis<br>Inclusion criterium #3<br>Protocol	p. 10<br>p. 60	The inclusion criterium has been amended to exclude patients with stage IV of disease.	Clinical rational
Synopsis<br>Inclusion criterium #8<br>Protocol	p. 11<br>p. 60	The inclusion criterium has been modified to state that female subjects of childbearing potential must practice not one reliable, but two highly effective methods of contraception and that additional pregnancy testing must be performed every 6 weeks during the treatment Combo-Immuno and every 4 weeks during the treatment Combo-Target, as well as at the end of the systemic exposure.	Clinical rational
Synopsis<br>Exclusion criterium #4<br>Protocol	p. 11<br>p. 61	The exclusion criterium has been amended to exclude patients only with stage III (unresecteble) or stage IV melanoma.	Clinical rational
Synopsis<br>Exclusion criterium #7<br>Protocol	p. 12<br>p. 61	The exclusion criterium has been amended to exclude patients only with uncontrolled cardiovascular disease.	Clinical rational

**Section**	**page**	**Description of change**	**Reason for change**
Synopsis<br>New exclusion criterium #8<br><br>Protocol	p. 12<br><br>p. 61	The exclusion criterion number 8 has been added to exclude patients with previous or concurrent malignant, except: adequately treated basal cell or squamous cell skin cancer; in situ carcinoma of the cervix, treated curatively and without evidence of recurrence for at least 3 years prior to study entry; or other solid tumor treated curatively, and without evidence of recurrence for at least 3 years prior to study entry.	New exclusion criteria
Synopsis<br>Biomarkers ancillary study endpoints<br><br>Protocol<br>3.2 Endpoints of the study - Biological markers (Biomarkers ancillary translational study)	p. 14<br><br>p. 39	The following sentence has been deleted: Immunotherapy SNP Panel will be also assessed at baseline	Clinical rational
Synopsis<br>Duration of the study	p.15	Reference of the study dates has been amended to global and the Global Recruitment end has been postponed to December.	Timelines update
Schedule Assessment<br>Arm A/Arm B/Arm C	p. 19-23	The Schedule Assessment has been harmonized with minor corrections.	Text Hamonization and minor corrections
Protocol<br>2.2.4 Nivolumab	p. 29	Sentence: ‘In a recent study’ has been amended to ‘In a previous study’.	Text harmonization
	p.30	The following information has been added:<br>In the phase III trial (Robert et al.),418 previously untreated	Addition

Section	page	Description of change	Reason for change
		patiens who had metastatic melanoma without a BRAF mutation were randomized to receive nivolumab (at a dose of 3 mg per kilogram of body weight every 2 weeks and dacarbazine-matched placebo every 3 weeks) or dacarbazine (at a dose of 1000 mg per square meter of body-surface area every 3 weeks and nivolumab-matched placebo every 2 weeks). The primary end point was overall survival. At 1 year, the overall rate of survival was 72.9% (95% confidence interval [CI], 65.5 to 78.9) in the nivolumab group, as compared with 42.1% (95% CI, 33.0 to 50.9) in the dacarbazine group (hazard ratio for death, 0.42; 99.79% CI, 0.25 to 0.73; P<0.001). The median progression-free survival was 5.1 months in the nivolumab group versus 2.2 months in the dacarbazine group (hazard ratio for death or progression of disease, 0.43; 95% CI, 0.34 to 0.56; P<0.001). The objective response rate was 40.0% (95% CI, 33.3 to 47.0) in the nivolumab group versus 13.9% (95% CI, 9.5 to 19.4) in the dacarbazine group (odds ratio, 4.06; P<0.001). The survival benefit with nivolumab versus dacarbazine was observed across prespecified subgroups, including subgroups defined by status regarding the programmed death ligand 1 (PD-L1). Common adverse events associated with nivolumab included fatigue, pruritus, and nausea. Drugrelated adverse events of grade 3 or 4 occurred in 11.7% of the patients treated withnivolumab and 17.6%	

Section	page	Description of change	Reason for change
		of those treated with dacarbazine.	
2.2.6 Nivolumab and Ipilimumab combination	p. 32	The following information has been added: Recently updated data about the comination have been published (Wolchok et al, 2017). At a minimum follow-up of 36 months, the median overall survival had not been reached in the nivolumab-plus-ipilimumab group and was 37.6 months in the nivolumab group, as compared with 19.9 months in the ipilimumab group (hazard ratio for death with nivolumab plus ipilimumab vs. ipilimumab, 0.55 [P [P<0.001];hazard ratio for death with nivolumab vs. ipilimumab, 0.65 [P<0.001]). The overall survival rate at 3 years was 58% in the nivolumab-plus-ipilimumab group and 52% in the nivolumab group, as compared with 34% in the ipilimumab group. The safety profile was unchanged from the initial report. Treatment-related adverse events of grade 3 or 4 occurred in 59% of the patients in the nivolumab-plus-ipilimumab group, in 21% of those in the nivolumab group, and in 28% of those in the ipilimumab group.	Addition
2.3 Study rationale	p. 33-34	The following information has been added: Taking into account the recent updated data of Check-Mate 067 (Wolchok et al. N Engl J Med. 2017) In the two nivolumab-containing groups, the median overall survival was not reached among patients with BRAF mutations, and the rate of overall survival at 3 years was 68% in the nivolumab-plus-ipilimumab group and 56% in the	Addition

Section	page	Description of change	Reason for change
		nivolumab group. In a descriptive analysis, the hazard ratio for death with nivolumab plus ipilimumab versus nivolumab was 0.69 (95% CI, 0.44 to 1.07). Among patients without BRAF mutations, the median overall survival was reached in all three treatment groups. Additional analyses were performed to investigate efficacy according to the tumor PD-L1 expression level. Descriptive comparisons between the two nivolumab-containing groups suggest that as the data become more mature, better survival outcomes may be obtained with combination therapy than with monotherapy in patients with a lower tumor PD-L1 expression level. However, overall survival was similar between the nivolumab-plus-ipilimumab group and the nivolumab group among patients with a tumor PD-L1 expression level of 1% or more or a level of 5% or more. The overall response rate was higher in the nivolumab-plus-ipilimumab group than in the nivolumab group at each tu-mor PD-L1 expression level tested.<br><br>Moreover in the light of the recent published data of Check-Mate 067 the combination therapy resulted in a higher rate of objective response than nivolumab alone regardless of the tumor PD-L1 expression level. Data from a phase 1b study, CA209-038, showed that tumor PD-L1 expression is up-regulated to a greater extent with combination treatment than with nivolumab alone, concomitant with a greater increase in	

Section	page	Description of change	Reason for change
		interferon-γ, CXCL9,and CXCL10 expression in the tumor microenvironment. The ROC-curve analyses did not identify a threshold of tumor PD-L1 expression for the discrimination of a difference in overall survival, which suggests that the tumor PD-L1 expression level alone may not be a definitive predictive biomarker of outcomes in patients with advanced melanoma, thus the tumor PD-L1 testing is not required.	
2.4 Final Risk/Benefit Statement	p. 35-36	The following information has been added: All hepatic adverse events were asymptomatic and reversible with either temporary discontinuation of the study drugs or administration of glucocorticoids.<br><br>The risks of administration of a combination of ipilimumab and nivolumab are known to be potential clinically meaningful drug-related AEs, which may require early recognition and prompt intervention. Management algorithms have therefore been developed for suspected pulmonary toxicity, GI, hepatotoxicity, endocrinopathy, skin toxicity, neurological toxicity and nephrotoxicity (Appendix III). Recommendations are to follow the nivolumab Investigator's Brochure adverse event algorithms. Dosing delay or discontinuation may then be implemented based on individual safety and tolerability (Section 5.2.2).<br><br>Ongoing clinical studies suggest that the combination of LGX818	New section

Section	page	Description of change	Reason for change
		with MEK162 may in fact have an improved safety profile compared to the respective single agent therapies. However, known risks of this combination comprise MEK inhibitor-associated retinal syndromes and other toxic effects on the skin (e.g. rash) and gastro-intestinal system (e.g. nausea). Again, management algorithms are in place (Appendices I and II) to enable timely intervention if required. Dosing modifications, interruptions and delays can then be implemented based on individual safety and tolerability (Section 5.1.2).<br><br>…<br><br>Updated data were reported on targeted therapies, confirming the excellent results previously reported (Larkin J e t al. Combined vemurafenib and cobimetinib in BRAF-mutated melanoma. N Engl J Med 371:1867–1876 2014; Long GV et al. Combined BRAF and MEK inhibition versus BRAF inhibition alone in melanoma. N Engl J Med 371:1877–1888 2014)<br><br>An update on the CoBRIM trial of combined BRAF inhibitor (vemurafenib) plus MEK inhibitor (cobimetinib) in patients with BRAFV600 mutation-positive tumors confirmed its superior impact on progression-free survival (PFS) compared to vemurafenib monotherapy [12.3 vs 7.2 months; hazard ratio (HR) 0.58 (0.46–0.72)]. An update on overall survival (OS) from the Combi-D study of combined dabrafenib plus trametinib in	

Section	page	Description of change	Reason for change
		patients with BRAF V600E/K metastatic melanoma was also reported (Long GV et al. 2015).<br><br>Patients treated with the combination of dabrafenib and trametinib achieved a median OS of 25.1 months with 51% of patients still alive at 2 years, these findings confirmed results reported from the phase I–II study in 2014 (Flaherty et al. 2014)<br><br>Finally, data from a phase Ib/II open-label study of patients with BRAFV600-mutant cutaneous melanoma treated with the newer combination of encorafenib plus binimetinib showed an overall response rate (ORR) of 74.5% and a disease control rate (DCR) of 96.4%. Of interest, in the cohort receiving a dosage regimen of encorafenib 400/450 mg and binimetinib 45 mg, the ORR was 77.5% and the DCR was 100%. e combination was also well tolerated, with no grade 3–4 pyrexia or skin tox- icity events reported (Sullivan RJ et al. 2015).	
3.5.3 Clinical Assessments and Procedures Screening		The information about pregnancy test performace has been amendended. The test should be performed during the treatment, Combo-Immuno and every 4 (± 1) weeks (within 24 hours prior to administration of study drug both serum or urine are accepted) during the treatment Combo-Target.	Text Hamonization and minor corrections
3.5.4 Biomarker study - Tumor biopsies	p.51	The following sections have been added:<br><br>Note: Baseline tumor biopsies can be archival if is collected	New section

Section	page	Description of change	Reason for change
	p. 52	after prior systemic therapy; otherwise a fresh biopsy will be collected.<br><br>Sample handling<br><br>The ancillary study requires blood sample collection at baseline, on-treatment (within first 4 weeks after first dose), and upon progression when feasible.<br><br>To assess the immunological biomarkers, in all arms the following peripheral blood samples must be collected:<br><br>A. 10 ml of non-anticoagulated whole blood for collection of serum (2 tubes x 5 ml tubes with red/yellow stopper);<br><br>B. 9 ml of whole blood in heparin for collection of plasma (2 x 4.5 ml tubes with green stopper);<br><br>C. 50 ml of whole blood in EDTA tubes for isolation of PBMC (5 x 10 ml tubes with lilac stopper);<br><br>D. 5 ml in serum tubes (1 tube x 5 ml with stopper);<br><br>The samples will be processed locally and then shipped in a central laboratory.<br><br>The details of sample processing will be provided separately to each site in a study sample manual.	
4.2.1 Women of	p. 62	The following section has been added: A woman is considered to	New Section

Section	page	Description of change	Reason for change
Childbearing Potential		be of childbearing potential if she is postmenarcheal, has not reached a postmenopausal state (≥ 12 continuous months of amenorrhea with no identified cause other than menopause), and has not undergone surgical sterilization (removal of ovaries and/or uterus).<br><br>Examples of contraceptive methods with a failure rate of < 1% per year include bilateral tubal ligation, male sterilization, hormonal contraceptives that inhibit ovulation, hormone−releasing intrauterine devices, and copper intrauterine devices. Hormonal contraceptive methods must be supplemented by a barrier method. The reliability of sexual abstinence should be evaluated in relation to the duration of the clinical trial and the preferred and usual lifestyle of the patient. Periodic abstinence (e.g., calendar, ovulation, symptothermal, or postovulation methods) and withdrawal are not acceptable methods of contraception<br><br>For men: agreement to remain abstinent (refrain from heterosexual intercourse) or use contraceptive measures, and agreement to refrain from donating sperm, as defined below: With female partners of childbearing potential, men must remain abstinent or use a condom plus an additional contraceptive method that together result in a failure rate of < 1% per year during the treatment period and for at least 6 months after the last dose of study treatment. Men must refrain from donating sperm during this same period. With	

Section	page	Description of change	Reason for change
		pregnant female partners, men must remain abstinent or use a condom during the treatment period and for 6 months after the last dose of study treatment to avoid exposing the embryo. The reliability of sexual abstinence should be evaluated in relation to the duration of the clinical trial and the preferred and usual lifestyle of the patient. Periodic abstinence (e.g., calendar, ovulation, symptothermal, or postovulation methods) and withdrawal are not acceptable methods of contraception	
4.6 Criteria for Premature Withdrawal	p. 65	The following paragraphs have been added: In addition, the Investigator may discontinue a participant, without their consent, from the trial at any time if the Investigator considers it necessary for any reason including:<br>• Pregnancy<br>• Ineligibility (either arising during the trial or retrospectively having been overlooked at screening)<br>• Significant protocol deviation<br>• Significant non-compliance with treatment regimen or trial requirements<br>• An adverse event which requires discontinuation of the trial medication or results in inability to continue to comply with trial procedures<br>• Any other condition which requires discontinuation of the trial medication or results in inability to continue to comply	New Section

Section	page	Description of change	Reason for change
	p. 65<br><br>p. 65	with trial procedures<br>• Withdrawal of Consent<br>• Loss to follow up<br><br>Patients who discontinue from a study treatment due to an Adverse event will be follow until the Progression disease according to the protocol (CT SCAN every 8 weeks from day 1). A safety follow up within 28 days of the last dose is required. The patient can switched at other ARM after the progression disease.<br><br>Any administrative or other reasons for withdrawal must be documented and explained to the patient.	
		The following paragraph has been deleted: When applicable, patients should be informed of circumstances under which their participation may be terminated by the Investigator without their consent. The Investigator may withdraw patients from the study in the event of intercurrent illness, AEs, treatment failure after a prescribed procedure, lack of compliance with the study and/or study procedures (e.g., dosing instructions, study visits), or any reason where it is felt by the Investigator that it is in the best interest of the patient to be terminated from the study. Any administrative or other reasons for withdrawal must be documented and explained to the patient.	Minor correction

Section	page	Description of change	Reason for change
4.7 Definition of End of Trial	p. 66	The following sentence has been added: The end of trial is the date of the last visit of the last participant	New Section
5.1.2 Dose Modifications, Interruption and delays criteria for Combo Target	p. 68	The following sentence has been added: In case of adverse events or other reasons, the patient restarts the treatment from the current day (day corresponding to the current treatment cycle day) and not from the last day.	New Section
5.4 Treatment after the End of the Study	p. 85	The following sentence has been added: Study drugs will not be available to subjects after the study has concluded. However, if further treatment is required, local standard of care will apply.	New Section
7.5.3 Pregnancy	p. 97-98	The following section has been added: Investigators will counsel WOCBP and male subjects who are sexually active with WOCBP on the importance of pregnancy prevention and the implications of an unexpected pregnancy.<br><br>Investigators will advise WOCBP and male subjects who are sexually active with WOCBP on the use of highly effective methods of contraception. Highly effective methods of contraception have a failure rate of < 1% when used consistently and correctly.<br><br>As stated in the inclusion criteria, at a minimum, subjects must agree to the use of two methods of contraception, with one method being highly effective and the other method being either highly effective or less effective as listed below:<br><br>Highly effective methods of	New Section

Section	page	Description of change	Reason for change
		contraception have a failure rate of <1% when used consistently and correctly. WOCBP and female partners of male subjects, who are WOCBP, are expected to use one of the highly effective methods of contraception listed below. Male subjects must inform their female partners who are WOCBP of the contraceptive requirements of the protocol and are expected to adhere to using contraception with their partner. Contraception methods are as follows:<br>1. Progestogen only hormonal contraception associated with inhibition of ovulation.<br>2. Hormonal methods of contraception including oral contraceptive pills containing combined estrogen + progesterone, vaginal ring, injectables, implants and intrauterine devices (IUDs) such as Mirena.<br>3. Nonhormonal IUDs, such as ParaGard.<br>4. Bilateral tubal occlusion.<br>5. Vasectomised partner with documented azoospermia 90 days after procedure. Vasectomised partner is a highly effective birth control method provided that partner is the sole sexual partner of the WOCBP trial participant and that the vasectomized partner has received medical assessment of the surgical success.<br>6. Intrauterine hormone-releasing system (IUS).	

Section	page	Description of change	Reason for change
		7. Complete abstinence:<br><br>a. Complete abstinence is defined as the complete avoidance of heterosexual intercourse. (refer to Glossary of Terms).<br><br>b. Complete abstinence is an acceptable form of contraception for all study drugs and must be used throughout the duration of the study treatment (plus 5 half-lives of the investigational drug plus 30 days).<br><br>c. It is not necessary to use any other method of contraception when complete abstinence is elected.<br><br>d. Subjects who choose complete abstinence must continue to have pregnancy tests, as specified in Section 3.5.3.<br><br>e. Acceptable alternate methods of highly effective contraception must be discussed in the event that the subject chooses to forego complete abstinence.<br><br>f. The reliability of sexual abstinence needs to be evaluated in relation to the duration of the clinical trial and the preferred and usual lifestyle of the subject.<br><br>Less effective methods of contraception:<br><br>1. Diaphragm with spermicide<br><br>2. Cervical cap with spermicide<br><br>3. Vaginal sponge with spermicide	

Section	page	Description of change	Reason for change
		4. Male or female condom with or without spermicide*<br>5. Progestogen-only oral hormonal contraception, where inhibition of ovulation is not the primary mode of action.<br>*A male and a female condom must not be used together.<br>Unacceptable methods of contraception:<br>1. Periodic abstinence (calendar, symptothermal, post-ovulation methods)<br>2. Withdrawal (coitus interruptus)<br>3. Spermicide only<br>4. Lactation amenorrhea method (LAM)	

SECTIONS UPDATED - PROTOCOL VERSION 6.0

Section	page	Description of change	Reason for change
Synopsis<br>Exclusion criteria #15<br><br>Section 4.2	p. 12<br><br><br>p. 62	The exclusion criteria in the protocol have been modified to state that patients who receive live vaccines within the previous 30 days are excluded.	Request from MHRA in UK
section 4.5	p. 65	Drug Interaction has been amended to indicate that use of live vaccines is prohibited during the study and for three months after last dose of PD-1 blocker (nivolumab).	Request from MHRA in UK
Synopsis<br>Exclusion criterium #16<br><br>Section 4.2	p. 12<br><br><br>p. 62	The exclusion criteria in the protocol have been modified to explicitly exclude patients with a history of severe or life-threatening skin adverse events or reactions.	Request from MHRA in UK

Section	page	Description of change	Reason for change
Section 7.5.2	p. 95	Reporting of SAEs (immediately reportable) has been amended in accordance with the regulation by replacing "one working day" with "24 hours".	Request from MHRA in UK
Section 8.5	p. 100	The study does have a DMC and the protocol has been amended to clarify the functions of the DMC () and its membership and their areas of expertise.	Request from MHRA in UK
Synopsis<br>Exclusion criterium #13<br><br>Section 4.2	p. 12<br><br><br>p. 62	The exclusion criterion number 13 has been amended to include a positive result for HIV testing.	Request from MHRA in UK

SECTIONS UPDATED - PROTOCOL VERSION 7.0

Section	page	Description of change	Reason for change
Synopsis<br>Inclusion criterium #8-9<br><br>Section 4.1	p. 11<br><br><br>p. 60	Inclusion criteria #8 and #9 have been changed according to Array's current standard contraception language	Request from ANSM in France
Synopsis<br>Inclusion criterium # 16-17<br><br>Section 4.1	p. 11<br><br><br>p. 61	The inclusion criteria #16 an #17 have been added – (Array's standard cardiac inclusion criterion recommendations) to include patient with adequate cardiac function.	Request from ANSM in France
Section 5.1.2	p. 73	An additional dose modification language has been incorporated in the protocol, as a procedure in case of QT/QTc > 500 msec	Request from ANSM in France

SECTIONS UPDATED - PROTOCOL VERSION 8.0

Section	page	Description of change	Reason for change
Several sections	p. 9-13-15-	Time-point at 2 years has	Study Statistician

Section	page	Description of change	Reason for change
	37-38-39-88-91	been deleted	decision based on CEC request of clarification in Austria
Statistical methods	p. 14 - 89	A sentence to specify that the total PFS will be considered the main secondary criteria (together with the other secondary endpoints) to select the best strategy has been added.	Study Statistician decision based on CEC request of clarification in Austria
Section 3.5.4	p. 51	The allocation of biopsies to the main study or to the biomarker study has been clarified.	Sponsor's decision based on CEC request of clarification in Austria
Section 3.5.4	p. 51	The procedure tissue sample collection has been better described, adding more information on which conditions general anesthesia me be done.	CEC request of clarification in Austria
Section 3.5.4	p. 52	baseline 2 time-point for a mandatory peripheral blood samples collection has been added	Correction.
Section 5.1.2 – table of Recommended Dose Modifications for LGX818/MEK162 combination	p. 74	Creatine phosphokinase (CPK) G3 and G 4– change from 14 to 28 the time limit for MEK162 dose resuming or permanent discontinuation	Sponsor's decision based on Columbus study data
Section 6.2.1	p. 89	Percentage of surviving at 24 months derivation has been explained.	Study Statistician decision based on CEC request of clarification in Austria
Section 6.2.1	p. 89	The conditions for further investigations have been more detailed.	Study Statistician decision based on CEC request of clarification in Austria
Section 6.2.3	p. 90	A sentence has been added to specify for each endpoint whether one or two-sided analysis is used.	Study Statistician decision based on CEC request of clarification in Austria
Throughout the document	Several pages	The Wording *Biomarker study* has been reported in every section the study on	Wording harmonization

Section	page	Description of change	Reason for change
		biomarkers was mentioned (the wording *ancillary* and *translational* has been removed)	

SECTIONS UPDATED - PROTOCOL VERSION 9.0

Section	page	Description of change	Reason for change
Synopsis	p. 16	The following information regarding Duration of the Study have been updated:<br>'Global Recruitment end' and 'Global Study end' have been 6 months postponed.	Basing on the enrollment rate, there was a prolongation of the recruitment phase.
Section 5.1.1-5.1.3	p.70<br>p.80	The information regarding supply of LGX818 has been amended. LGX818 will be provided as 75 and 50 mg capsules.	Array Biopharma's decision of changing the supply of LGX818, by replacing 100 mg capsules with 75mg ones.
Section 5.1.3	p.80	The following sentence has been added:<br>LGX818 50 mg will be provided in small quantities to support dose reductions (dose level -2, -3, -4).	Array Biopharma's decision about the use of 50 mg capsules of LGX818.
Section 5.2.1	p.81	In relation to Combo Immuno dose and treatment schedule, the following sentence has been added:<br>Subjects may be dosed no less than 12 days from the previous dose.	Sponsor decision to outline this point
Synopsis,<br>Tables 1-3,<br>Sections:<br>3.3,	p.10<br>p. 20-25<br>p. 42-50<br>p. 58	Information regarding the schedule of assessments have been added.	Sponsor decision to outline this point

Clinical Research Technology S.r.l.
www.cr-technology.com
info@cr-technology.com

3.4 - 3.5.4<br>3.5.6			
Section 4.6	p. 68	Information regarding discontinuation of study treatment have been added.	Sponsor decision to outline this point
Section 6.3	p. 93	The following information regarding Duration of the Study have been updated:<br>'Global Recruitment end' and 'Global Study end' have been 6 months postponed.	Basing on the enrollment rate there was a prolongation of recruitment phase.
Section 7.2.5	p. 97	Information regarding the management of the event 'Death due to progression of disease' have been added.	Sponsor decision to outline this point
Synopsis,<br>Table 1-3,<br>Sections:<br>3.3,<br>3.4<br>3.5.3,<br>4.6,<br>7.3,<br>7.5.2	p.10, 13,21,<br>23,25,41,44, 51,68,98, 102	The Follow up period has been increased from 24 to 36 months	Sponsor decision to monitor patients survival until 3 years from the end of study treatment

CLINICAL RESEARCH TECHNOLOGY

SECTIONS UPDATED - PROTOCOL VERSION 10.0

Section	page	Description of change	Reason for change
Synopsis (Study Plan and Study Treatent duration, dose and schedule)<br>Schedule of assessments<br>Section 3.4<br>Section 3.5.3<br>Section 4.6<br>Section 7.7<br>Section 7.3<br>Section 7.5.2	p. 10, 13<br>p 21,23,25<br>p 44<br>p. 51<br>p. 68<br>p. 69<br>p. 99<br>p 103	The Follow up period has been increased from 36 to 60 months from randomization	Sponsor decision to monitor patients survival until 5 years from randomization of last patient
Synopsis (Study Treatent duration, dose and schedule)<br>Schedule of assessments<br>Section 3<br>Section 3.4<br>Section 4.3<br>Section 5<br>Section 5.2.1	p.13<br>p 21,23,25<br>p. 38<br>p.43<br>p. 65<br>p. 70<br>p. 81	The following doses and schedules of Nivolumab have been added: 240 mg every 2 weeks or 480 mg every 4 weeks	Dose and schedule of Nivolumab 240 mg every 2 weeks or 480 mg every 4 weeks have been approved by CA
Synopsis (Study Treatent duration, dose and schedule)<br>Section 3.4<br>Section 4.6<br>Section 5.2.2	p. 13<br>p. 43<br>p. 68<br>p. 85	The following sentence has been added:<br>To reduce the risk of long-term toxicity, no more than 2 years of ipilimumab/nivolumab dosing will be administered to study patients	Evidence-based sponsor's decision<br>(CheckMate 067 and KEYNOTE-001 KEYNOTE-006, CheckMate 153)
Synopsis (Duration of the Study)<br>Section 6.3	p. 16<br>p. 94	Study duration was postponed to May 2024. Patients still on treatment in May 2024 will be treated according to clinical practice	Change due to FU prolongation

CLINICAL STUDY PROTOCOL

SECOMBIT

”Sequential Combo Immuno and Target therapy (SECOMBIT) study”

“A three arms prospective, randomized phase II study to evaluate the best sequential approach with combo immunotherapy (ipilimumab/nivolumab) and combo target therapy (LGX818/MEK162) in patients with metastatic melanoma and BRAF mutation”

Principal Investigators (PI): **Dr. Paolo A. Ascierto**
Istituto Nazionale dei Tumori, Fondazione “G. Pascale”
U.O.C. Melanoma, Immunoterapia Oncologica e Terapie Innovative
Via M. Semmola 80131 - Naples

Protocol code: SECOMBIT
Protocol version: 1.0
Protocol date: 22th October 2015
EudraCT number: 2014-004842-92
Sponsor: Fondazione Melanoma (ONLUS)
c/o Istituto Tumori Napoli
Fondazione “G. Pascale”
Via Mariano Semmola 80131 Naples, Italy

CONFIDENTIAL

This document contains confidential information belonging to Sponsor. Except as may be otherwise agreed to in writing, by accepting or reviewing these materials, you agree to hold such information in confidence and not to disclose it to others (except where required by applicable law), nor to use it for unauthorized purposes. In the event of actual or suspected breach of this obligation Sponsor should be promptly notified.

SPONSOR SIGNATURE

Dr. Gennaro Parisi
Fondazione Melanoma ONLUS
Sponsor representative

Signature

22/OCT/2015
Date

PRINCIPAL INVESTIGATOR

Dr. Paolo A. Ascierto
Istituto Nazionale dei Tumori, Fondazione "G. Pascale"
U.O.C. Melanoma, Immunoterapia Oncologica e Terapie Innovative
Via M. Semmola 80131 - Naples

Signature

27/OCT/2015
Date

STATISTICIAN

Dr. Diana Giannarelli
IFO- Istituto Nazionale Tumori Regina Elena
Unità di Biostatistica
Via Elio Chianesi, n. 53 - (Eur) - Roma

Signature

18/NOV/2015
Date

PROTOCOL APPROVAL

CLINICAL STUDY PROTOCOL

SECOMBIT

"Sequential Combo Immuno and Target therapy (SECOMBIT) study"

"A three arms prospective randomized phase II study to evaluate the best sequential approach with combo immunotherapy (ipilimumab/nivolumab) and combo target therapy (LGX818/MEK162) in patients with metastatic melanoma and BRAF mutation"

I agree to the terms of this study protocol. I will conduct the study according to the procedures specified herein, and according to principles of Good Clinical Practices and local regulations and requirements.

Investigator:

Site Number: ____________________________________

Name: ____________________________________

Signature: ____________________________________

Date: _________________

ADDITIONAL TRIAL PERSONNEL/SITE INFORMATION

**Principal Investigators (PI)**	**Dr. Paolo A. Ascierto**<br>Istituto Nazionale dei Tumori, Fondazione "G. Pascale"<br>U.O.C. Melanoma, Immunoterapia Oncologica e Terapie Innovative<br>Via M. Semmola 80131 - Naples	Tel: +39 081 5903 236<br>Fax: +39 081 5903 841<br>Email paolo.ascierto@gmail.com
**Study coordinator and Data management**	**Dr. Marcello Curvietto**<br>Istituto Nazionale dei Tumori, Fondazione "G. Pascale"<br>U.O.C. Melanoma, Immunoterapia Oncologica e Terapie Innovative<br>Via M. Semmola 80131 - Naples	Tel: +39 081 5903 841<br>Fax: +39 081 5903 841<br>Email curvietto.ma@gmail.com
**Statistician**	**Dr. Diana Giannarelli**<br>IFO- Istituto Nazionale Tumori Regina Elena<br>Unità di Biostatistica<br>Via Elio Chianesi, n. 53 - (Eur) - Roma	Tel: +39 06 52665607<br>Fax: +39 06 52662463<br>Email giannarelli@ifo.it
**Safety desk (SAE reporting)**	**Clinical Research Technology srl**<br>Pharmacovigilance Unit	Tel: +39 089.301545<br>Fax: +39 089.7724155<br>e-mail: pvg@cr-technology.com

**Emergency contact**	**Dr. Paolo A. Ascierto**<br>Istituto Nazionale dei Tumori, Fondazione "G. Pascale"<br>U.O.C. Melanoma, Immunoterapia Oncologica e Terapie Innovative<br>Via M. Semmola 80131 - Naples	Tel: +39 081 5903 236<br>Fax: +39 081 5903 841<br>Email paolo.ascierto@gmail.com
	**Clinical Research Technology srl**<br>Project Management	Tel: +39 089.301545<br>Fax: +39 089.7724155<br>e-mail: pvg@cr-technology.com

TABLE OF CONTENTS

SECOMBIT 1
TABLE OF CONTENTS 5
PROTOCOL SYNOPSIS 8
LIST OF ABBREVIATIONS 16
1. SCHEDULE ASSESSMENT 19
2. BACKGROUND AND RATIONALE 25
2.1 OVERVIEW OF DISEASE EPIDEMIOLOGY AND CURRENT TREATMENT 25
2.2 INTRODUCTION TO INVESTIGATIONAL TREATMENTS AND OTHER STUDY TREATMENT(S) 27
2.3 STUDY RATIONALE 32
3. STUDY AIMS AND DESIGN 33
3.1 OBJECTIVES OF THE STUDY 33
3.2 ENDPOINTS OF THE STUDY 34
3.3 STUDY DESIGN 36
3.4 STUDY SCHEDULE 37
3.5 SCHEDULE OF ASSESSMENTS AND PROCEDURES 38
4. STUDY POPULATION 50
4.1 INCLUSION CRITERIA 50
4.2 EXCLUSION CRITERIA 51
4.3 STUDY TREATMENTS 52
4.4 CONCOMITANT MEDICATION AND TREATMENT 52
4.5 DRUG INTERACTION 53
4.6 CRITERIA FOR PREMATURE WITHDRAWAL 54
5. INVESTIGATIONAL MEDICINAL PRODUCTS 55
5.1 COMBO TARGET 55
5.2 COMBO IMMUNO 65
5.3 ACCOUNTABILITY, ASSESSMENT OF COMPLIANCE AND DESTRUCTION OF THE DRUGS 71
6. STATISTICAL CONSIDERATIONS 73
6.1 STUDY ENDPOINTS 73
6.2 SAMPLE SIZE AND ANALYSIS POPULATIONS 73
6.3 STUDY DURATION 76
7. SAFETY INSTRUCTIONS AND GUIDANCE 77
7.1 WARNING AND PRECAUTIONS 77
7.2 ADVERSE EVENTS AND LABORATORY ABNORMALITIES 77

7.3 TREATMENT AND FOLLOW-UP OF AEs (100 DAYS POST DISCONTINUATION OF STUDY DRUGS) .. 80
7.4 LABORATORY TEST ABNORMALITIES 81
7.5 HANDLING OF SAFETY PARAMETERS 81
8. DATA COLLECTION AND MANAGEMENT 85
8.1 DATA CONFIDENTIALITY 85
8.2 SITE MONITORING 85
8.3 DATA COLLECTION 85
8.4 DATABASE MANAGEMENT AND QUALITY CONTROL 86
9. ETHICAL CONSIDERATION 87
9.1 REGULATORY AND ETHICAL COMPLIANCE 87
9.2 RESPONSIBILITIES OF THE INVESTIGATOR AND IEC 87
9.3 INFORMED CONSENT PROCEDURES 87
9.4 PUBLICATION OF STUDY PROTOCOL AND RESULTS 87
9.5 STUDY DOCUMENTATION, RECORD KEEPING AND RETENTION OF DOCUMENTS 87
9.6 CONFIDENTIALITY OF STUDY DOCUMENTS AND PATIENT RECORDS 88
9.7 AUDITS AND INSPECTIONS 88
9.8 FINANCIAL DISCLOSURES 88
10. PROTOCOL ADHERENCE 88
10.1 AMENDMENTS TO THE PROTOCOL 88
11. REFERENCES 90
12. APPENDIX 92
I. RECOMMENDED GUIDELINES FOR THE MANAGEMENT OF STUDY DRUG (LGX818 AND MEK162) INDUCED SKIN TOXICITY 92
II. RECOMMENDED GUIDELINES FOR THE MANAGEMENT OF MEK162 INDUCED DIARRHOEA 94
III. RECOMMENDED ALGORITHMS FOR USE OF NIVOLUMAB AND IPILIMUMAB 96
IV. EORTC QLQ-C30 (VERSION 3) 103
V. EQ-5D 106
VI. WPAI:GH 108
VII. ECOG PERFORMANCE STATUS 110

Table 1: Schedule of Assessments and Procedures in Arm A 19
Table 2: Schedule of Assessments and Procedures in Arm B 21
Table 3: Schedule of Assessments and Procedures in Arm C 23
Table 4: Summary of study aim 36
Table 5: Study treatment scheme 38
Table 6: Biomarker study treatment scheme 43
Table 7: Treatments used in the study 52
Table 8: Combo Target dose and treatment schedule 55
Table 9: Dose reduction for LGX818 and MEK162 56
Table 10: Recommended dose modifications associated with treatment-related adverse events 63
Table 11: Packaging and labelling 64
Table 12: Combo Immuno dose and treatment schedule 65

PROTOCOL SYNOPSIS

**Study Title**	A three arms prospective randomized phase II study to evaluate the best sequential approach with combo immunotherapy (ipilimumab/nivolumab) and combo target therapy (LGX818/MEK162) in patients with metastatic melanoma and BRAF mutation.
**Study ID**	**SECOMBIT** (Sequential Combo Immuno and Target therapy study)
**Number of participant centers**	Participating sites: Approximately 20 sites in Italy and Europe<br>**Study coordinator:**<br>Paolo Antonio Ascierto<br>e-mail: paolo.ascierto@gmail.com<br>Tel: +39 0815903431<br>Fax: +39 0815903841<br>Mobile: +39 338 7402333
**Phase**	II
**Study Hypothesis**	To evaluate the best sequencing approach with the combination of target agents (LGX818 plus MEK162) and the combination of immunomodulatory antibodies (ipilimumab plus nivolumab) in patients with metastatic melanoma and BRAF V600 mutation.
**Background and rationale**	The combination BRAF (B-raf murine sarcoma viral oncogene homolog B1) inhibitor plus mitogen-activated protein kinase (MEK) inhibitor seems to be more effective in the V600 BRAF mutated advanced melanoma patients compared to treatment with the BRAF inhibitors alone. In fact, a phase I-II study (*Flaherty et al, 2012*) showed a better overall response rate (ORR) and progression-free survival (PFS) in the combination arm (dabrafenib plus trametinib) respect to the single agent treatment (dabrafenib): 76% and 9.4 months versus 54% and 5.8 months respectively. Another phase I study with a similar combination (vemurafenib plus cobimetinib) showed an ORR of 85% in vemurafenib-naïve patients (*Martinez Garcia et al, 2012*)<br>Recently, the results of a phase I study about the combination ipilimumab plus nivolumab have been reported (*Wolchock et al, 2013*). In this study at the selected schedule (ipilimumab 3 mg/kg and nivolumab 1 mg/kg), 53% of patients had an objective response, all with tumor reduction of 80% or more. Reponses were durable, although longer follow-up is needed.<br>A recent phase I study has shown a high rate of liver toxicity with the combo ipilimumab plus vemurafenib (*Ribas et al, 2013*) which makes difficult a combination with these two different drugs. Moreover, a better efficacy of the sequencing treatment BRAF inhibitors/ipilimumab vs. the single agent treatment was also observed; for this reason it was also suggested to start immunotherapy treatment in the BRAF V600 mutated melanoma population as first option, in order to increase the percentage of patients who can benefit from the sequencing (*Ascierto et al, 2012*; *Ascierto et al, 2013*), considering the possibility of a fast progression of the disease after the BRAF inhibitors treatment (*Ascierto et al, 2012*).

<table>
<tr><td></td><td> Taking into account these considerations, it seems impossible to think to combine all the four compounds (the target agents and immunomodulating monoclonal antibodies). The risk of a high rate of toxicity is realistic and would render this approach inapplicable. Sequencing with these different combinations seems to be more feasible. However, also in this case it would be important to start with the best combination in order to give to the patients the best chance to increase the overall survival. The aim of this prospective randomized phase II study is to evaluate the sequencing of these two different combinations and evaluate which is the best of these approaches. </td></tr>
<tr><td>Primary Objectives</td><td>To define the best sequencing combination treatment in primary efficacy variable overall survival (OS).</td></tr>
<tr><td>Secondary Objectives</td><td> To evaluate the effects of the two sequencing combination treatments on: Total PFS;
Percentage of patients alive at 2 and 3 years;
Best overall response rate (BORR);
Duration of response (DoR);
Toxicity of the investigational medicinal products (IMPs)
Quality of life and general health status defined by:
Health-related quality of life (HRQoL), by means of the 30-item European Organisation for Research and Treatment of Care quality of life questionnaire (EORTC QLQ-C30);
General health status, by means of the European Quality of Life 5-Dimensions(EQ-5D) questionnaire;
Impairment of work productivity and activity, by means of the Work Productivity and Activity Impairment: General Health (WPAI:GH) questionnaire; <u>Biological markers (biomarkers ancillary study)</u> The objective of the biomarkers ancillary study is to focus on understanding mechanisms of action/resistance. In particular, the ancillary study: Will inform how to sequence targeted RAF/MEK agents with immunotherapy agents (i.e. ipilumumab and nivolumab) in melanoma;
Will be hypothesis-generating only.</td></tr>
<tr><td>Study plan</td><td> The study will be conducted according to an open-label, prospective, randomized, phase II design. Randomization will be stratified according to stage arranged in the 4 following strata: IIIb/c – M1a
M1b
M1c with normal LDH
M1c with elevated LDH. Subjects will be assessed for response by computed tomography (CT) or Magnetic Resonance Imaging (MRI). </td></tr>
</table>

	All measurable and non-measurable lesions must be documented at screening (within 28 days prior to randomization) and re-assessed at each subsequent tumor evaluation (every 8 weeks for the first year, every 12 weeks while the patient is on study). Tumor assessments with CT or MRI scans of the chest, abdomen, and pelvis will be performed until disease progression after the second combo treatment per RECIST v1.1. Imaging of the neck should be included if clinically indicated. In the event positron emission tomography (PET)/CT scanner is used for tumor assessments, the CT portion of the PET/CT must meet criteria for diagnostic quality. All scans will be collected for a possible independent review.<br>For patients who discontinue study treatment for reason other than investigator–determined disease progression, tumor assessments should continue to be performed as scheduled.<br>For patients with palpable/superficial lesions, clinical disease assessments by physical examination should be performed at baseline and throughout study treatment as clinically indicated. Color photographs with ruler/calipers will be taken at baseline and at all subsequent tumor assessment time points.<br>The National Cancer Institute Common Toxicity Criteria for Adverse Events (NCI CTC-AE) Version 4.03 will be used to evaluate the clinical safety of the treatment in this study. Patients will be assessed for AEs at each clinical visit and as necessary throughout the study.<br><u>Biomarkers ancillary study</u><br>A correlative biological study will be performed for the evaluation of biomarkers on the biological samples available (paraffin-embedded tissue, frozen tissue, blood, serum, etc.).
**Sample size**	A total of 230 patients will be enrolled to ensure a minimum of 207 randomized patients.
**Study population**	The study population will include patients of either sex aged ≥ 18 years with metastatic melanoma and BRAF V600 mutation not pretreated.
**Inclusion criteria**	1) Patients of either sex aged ≥ 18 years;<br>2) Histologically confirmed stage III (unresectable) or stage IV melanoma with the BRAF V600 mutation. Patients with mucosal melanoma (but not those with ocular melanoma) are eligible for study participation;<br>3) Treatment naïve patients. As previous systemic treatment for melanoma only interferon is permitted (note that prior adjuvant melanoma therapy is permitted if completed at least 6 weeks prior to randomization, and all related adverse events have either returned to baseline or stabilized).<br>4) Measurable disease by computed tomography (CT) or Magnetic Resonance Imaging (MRI) per RECIST 1.1 criteria;<br>5) Presence of BRAF V600E or V600K mutation in tumor tissue prior to enrollment;<br>6) Eastern Cooperative Oncology Group (ECOG) performance status (PS) 0 or 1;<br>7) Tumor tissue from an unresectable or metastatic site of disease must be provided for biomarker analyses. An archive sample is mandatory at the screening visit; however, a fresh sample would be preferable;

	8) Female subjects of childbearing potential must have a negative pregnancy test result at Baseline and must practice a reliable method of contraception for the total study duration plus 23 weeks (i.e. 30 days plus the time required for nivolumab to undergo five half lives) after the last dose of nivolumab and ipilimumab;<br>9) Men who are sexually active with women of childbearing potential must practice a reliable method of contraception for the total study duration plus 31 weeks (i.e. 80 days plus the time required for nivolumab to undergo five half lives) after the last dose of nivolumab and ipilimumab;<br>10) Adequate bone marrow haematological function: absolute neutrophil count (ANC) ≥ 1.5 x 10^9/L AND platelet count ≥ 100 x 10^9/L AND haemoglobin ≥ 9 g/dL;<br>11) Adequate liver function: total bilirubin ≤ 1.5 x upper limit of normal (ULN) AND aspartate aminotransferase (AST)/alanine aminotransferase (ALT) ≤ 2.5 X ULN (< 5 x ULN if liver metastases);<br>12) Adequate renal function: serum creatinine ≤ 1.5 mg/dL OR creatinine clearance ≥ 60 mL/min in males and ≥ 50 mL/min in females (calculated according to Cockroft-Gault formula);<br>13) Serum calcium levels, international normalised ratio (INR) and partial thromboplastin time were within normal limits;<br>14) Life expectancy of at least 3 months;<br>15) Ability to understand study-related patient information and provision of written informed consent for participation in the study.
**Exclusion criteria**	1) Active brain metastases. Subjects with brain metastases are eligible if these have been treated and there is no magnetic resonance imaging (MRI) evidence of progression for at least 8 weeks after treatment is complete and within 28 days prior to first dose of study drug administration. There must also be no requirement for immunosuppressive doses of systemic corticosteroids (> 10 mg/day prednisone equivalents) for at least 2 weeks prior to study drug administration;<br>2) Subjects with active, known or suspected autoimmune disease;<br>3) Subjects with a condition requiring systemic treatment with either corticosteroids (>10 mg daily prednisone equivalents) or other immunosuppressive medications within 14 days of treatment;<br>4) Prior treatment with an anti-Programmed Death receptor-1 (PD-1), anti-Programmed Death-1 ligand-1 (PD-L1), anti-PD-L2, or anti-cytotoxic T lymphocyte associated antigen-4 (anti-CTLA-4) antibody;<br>5) Female subjects who are pregnant (positive pregnancy test), breast-feeding, or who are of childbearing potential and not practicing a reliable method of birth control;<br>6) Evidence of severe or uncontrolled systemic disease or any concurrent condition which in the investigator's opinion makes it undesirable for the patient to participate in the study, or which would jeopardize compliance with the protocol, or would interfere with the results of the study;<br>7) Patients with a history of cardiovascular or interstitial lung disease and evidence or risk of retinal vein occlusion or central serous retinopathy (Patients with a history of cardiovascular or interstitial lung disease and evidence or risk of retinal vein occlusion or central serous retinopathy (Past or present evidence of rethinophaty central serous retinopathy - CSR -, occlusion of retinal - RVOo retinal degenerative disease) or ophthalmopathy, which according to the ophthalmologic

	evaluation at baseline could be considered a risk factor for CSR / RVO (eg. cupping of the optic disc, visual field defect, intraocular pressure - (eg: central IOP - > 21 mmHg).;<br>8) History of Gilbert's syndrome;<br>9) Inability to regularly access centre facilities for logistical or other reasons;<br>10) History of poor co-operation, non-compliance with medical treatment, or unreliability;<br>11) Participation in any interventional drug or medical device study within 30 days prior to treatment start.<br>12) Positive test for hepatitis B virus surface antigen (HBV sAg) or hepatitis C virus ribonucleic acid (HCV antibody) indicating acute or chronic infection;<br>13) Known history of testing positive for human immunodeficiency virus (HIV) or known acquired immunodeficiency syndrome (AIDS)
**Study treatment duration, dose and schedule**	The following IMPs will be used in the study:<br>• **Arm A**: Combo Target (LGX818 450 mg p.o. od + MEK162. 45 mg p.o bid) until PD; then Combo Immuno (nivolumab 1 mg/kg solution intravenously (IV) combined with ipilimumab 3 mg/kg solution IV every 3 weeks for 4 doses then nivolumab 3 mg/kg solution IV every 2 weeks) until progression of disease (PD).<br>• **Arm B**: Combo Immuno (nivolumab 1 mg/kg solution IV combined with ipilimumab 3 mg/kg solution IV every 3 weeks for 4 doses then nivolumab 3 mg/kg solution IV every 2 weeks) until PD; then Combo Target (LGX818 450 mg p.o.od + MEK162 45 mg p.o. bid) until PD.<br>• **Arm C**: Combo Target (LGX818 450 mg p.o. od + MEK162 45 mg p.o. bid) for 8 weeks followed by Combo Immuno (nivolumab 1 mg/kg solution IV combined with ipilimumab 3 mg/kg solution IV every 3 weeks for 4 doses then nivolumab 3 mg/kg solution IV every 2 weeks) until PD; then Combo Target (LGX818 450 mg p.o. od + MEK162 45 mg p.o. bid) until PD.<br>Shift from the one combination therapy to the following one can be done (once the procedures of the new screening have been completed) in case of absence of AEs events from the previous combination therapy or in case of grade 1 AEs. In case of grade ≥ 2 AEs from the previous combination therapy, shift to the following combination therapy can be done only when the AE is resolved or has decreased in intensity to at least grade 1.
**Primary endpoint**	OS is primary endpoint of the study. OS will be calculated as the time from the date of randomization until the date of death from any cause. Any patient not know to have died at the time of data analysis will be censored at the time of the last recorded date on which the patient was know to be alive.
**Secondary endpoints**	• Total PFS, calculated from the date of randomization until the date of the second progression (i.e. the progression to second treatment); any progression or death will be considered as an event if patient cannot complete treatment sequence;<br>• Percentage of patients alive at 2 and 3 years;<br>• Best overall response rate (BORR);<br>• Duration of response (DoR) calculated as the time from the date of first documented response (CR or PR) until the date of the first documented progression or death due

	to underlying cancer. If the patient with a CR or PR has no progression or death due to underlying cancer, the patient will be censored at the time of last adequate tumor assessment;<br>• Biological markers (biomarkers ancillary study);<br>• Health-related quality of life (HRQoL), by means of the 30-item European Organisation for Research and Treatment of Care quality of life questionnaire (EORTC QLQ-C30);<br>• General health status, by means of the European Quality of Life 5-Dimensions (EQ-5D) questionnaire;<br>• Impairment of work productivity and activity, by means of the Work Productivity and Activity Impairment: General Health (WPAI:GH) questionnaire.
**Safety endpoints**	• Toxicity of the IMPs (NCI CTC-AE Version 4.03 criteria);<br>• Adverse events (AEs) and serious adverse events (SAEs)<br>• Vital signs (weight, BMI, heart rate, blood pressure);<br>• Laboratory safety parameters (haematology, blood chemistry, urinalysis).
**Biomarkers ancillary study endpoints**	Tumor tissue biomarkers:<br>• Immune status: CD3, CD8, and CD4 T cells; Activated T cells; T regulatory cells; Dendritic cells;<br>• Resistance to immunotherapy agents: Checkpoint receptors/ligands; myeloid-derived suppressor cells (MDSCs);<br>• Resistance to targeted agents: aberrations in MEK/PI3K pathways, cytokines that interact with tyrosine kinase receptors (VEGF, HGF and their cognate receptors);<br>• Mutational load and neoantigen profile.<br>Peripheral blood biomarkers:<br>• Immune status/"Resistance" to immunotherapy agents: Activated T cells; Memory/Exhausted T cells; T regulatory cells; MDSCs; Inflammatory response; C-reactive protein (CRP); TCR Sequencing/Gene Expression analysis;<br>• Response/Resistance to targeted agents: Apoptotic tumor cells (as measured by circulating tumor DNA); soluble hepatocyte growth factor (sHGF); serum vascular endothelial growth factor (sVEGF), soluble interleukin-2 receptor (sCD25);<br>• Immunotherapy SNP Panel will be also assessed at baseline.<br>Approximately 80-90 patients will take part in the ancillary study.
**Statistical methods**	Sample size determination<br>This study is designed as a phase II, randomized trial with no formal comparative test. The sample size is discussed for the primary endpoint of the study (Overall Survival).<br>For each arm a single-stage design will be used. We have assumed a median PFS of about 10 months for the combo target therapy (LGX818/MEK162) and a similar value for the combo immunotherapy (ipilimumab/nivolumab) derived from the aggregate clinical activity rate of 65% which, using an exponential distribution for PFS, could broadly give a median PFS of about 9.5 months. OS seems to be strictly correlated with total PFS.

	The null hypothesis is a median OS time of 15 months (i.e. percentage of surviving patients of 33% at 24 months). The altervative hypothesis is a median OS time of 23 months (i.e. percentage of surviving of 48% at 24 months).<br>Using an exact 5% one-sided significance test at least 69 patients have to be randomized in each arm when the power of the study is 80%.<br>For each arm the strategy will be further investigated if at least 30 patients, alive at 24 months, are observed. Taking in account a 10% drop-out rate, a total of 230 patients will be enrolled to ensure a minimum of 207 randomized patients.
	Data handling<br>The Overall Survival (OS) will be calculated as the time from the date of randomization until the date of death from any cause. Any patient not know to have died at the time of data analysis will be censored at the time of the last recorded date on which the patient was know to be alive.<br>Total Progression Free Survival (TPFS) will be calculated from the date of randomization to the date of the second progression (i.e. the progression to second treatment); any progression or death will be considered as an event if patient cannot complete treatment sequence.<br>Duration of response (Dor) will be calculated as the time from the date of first documented response (CR or PR) until the date of the first documented progression or death due to underlying cancer.<br>If the patient with a CR o PR has no progression death due to underlying cancer, the patient will be censored at the date of last adeguate tumor assessment.<br>Statistical analysis<br>A comprehensive Statistical Analysis Plan (SAP) will be prepared before database lock.<br>All enrolled patients in the study will be considered for the Screened Population.<br>All randomized will be considered the Intention-To-Treat population (ITT).<br>The subset of patient of the ITT population receiving at least one dose of the study medication will define the Safety Population (SP).<br>Analysis of efficacy endpoints will be performed in the ITT population whereas the safety analysis will be performed in the Safety Population.<br>No comparative tests between the three arms will be performed and results will be presented as descriptive statistics.<br>The standard summary statistics will be used for both continuous and discrete variables.<br>The objective response rate (ORR) and the percentage of patients alive at 2 o 3 years will be reported with its 95% confidence interval (CI).<br>The time-dependent endpoint will be analyzed according to the Kaplan-Meier method.<br>Median with 95% confidence intervals will be derived from the K-M curves and presenting time-dependent endpoints as K-M plot (with a 95% CI over time).<br>Cox's proportional hazard model will be used to assess the impact of known prognostic factors and treatment assigned.<br>The list of the covariates to be included in the Cox's model will be presented and clinically justified in the statistical analysis plan.

	Safety and tolerability data will be presented by treatment received.<br>Appropriate summaries of these data will be presented. Safety and tolerability will be assessed in terms of AEs, laboratory data, ECG data, vital signs and weight, which will be collected for all patients. AEs (both in terms of MedDRA preferred terms and CTCAE grade), laboratory data, ECG data, vital signs data and weight will be listed individually by the patient and summarised by treatment received.
**Duration of the study**	Treatment duration: until PD (2 years estimated)<br>Study Start (First Patient First Visit Date): Febrary 2016<br>Recruitment end (Last Patient First Visit): September 2017<br>Study end (Last Patient Last Visit): Semptember 2020*<br>*This date is dependent on the clinical course of the disease and may therefore occur earlier than indicated

LIST OF ABBREVIATIONS

AEs	Adverse Events
AESIs	Adverse Events of Special Interest
ALT	Alanine Transaminase (SGPT)
ANC	Absolute Neutrophil Count
AST	Aspartate Transaminase (SGOT)
ATP	Adenosin Triphosphate
AUC	Area Under the plasma concentration-time Curve
BCRP	Breast Cancer Resistant Protein
bid	Bis in die (twice daily)
BMI	Body Mass Index
BORR	Best Overall Response Rate
BRAF	B-raf murine sarcoma vioral oncogene homolog B1
BUN	Blood Urea Nitrogen
C/A/P	Chest/Abdomen/Pelvis
CI	Confidence Interval
CK	Creatin-kinase
Cmax	Maximum plasma concentration
CRP	C-reactive Protein
CT	Computed Tomography
CTC-AE	Common Terminology Criteria for Adverse Events
CTLA-4	Cytotoxic T Lymphocyte Antigen 4
DEHP	Di-(2-ethylexil-phthalate)
DILI	Drug-induced Liver Injury
DoR	Duration of Response
ECG	Electrocardiogram
EchO	Echocardiogram
ECOG	Eastern Cooperative Oncology Group
eCRF	Electronic Case Report Form
EORTC QLQ-C30	30-item European Organisation for Research and Treatment of Care quality of life questionnaire
EQ-5D	European Quality of Life 5-Dimensions
EOS	End of Study Visit
ERK	Extracellular Signal-Regulated Kinase
FDA	Food and Drug Administration

FFPE	Formalin-fixed Paraffin-embedded
HFSR	Hand Foot Skin Reaction
HCG	Human Chorionic Gonadotropine
HGF	Hepatocyte Growth Factor
HRQoL	Health-related Quality of Life
ICF	Informed Consent Form
ICH	International Conference of Harmonization
IMP	Investigational Medicinal Product
INR	International Normalized Ratio
IRB/IEC	Institutional Review Board / Independent Ethics Committee
IV	Intravenous
KA	Keratoacanthoma
Kg	Kilogram
LDH	Lactate dehydrogenase
LFT	Liver Function Tests
LLN	Lower Limit of Normal
LVEF	Left Ventricle Ejection Fraction
MAPK	Mitogen Activated Protein Kinase
MedDRA	Medical Dictionary for Regulatory Activities
MDSCs	Myeloid-derived Suppressor Cells
MEK	Methyl Ethyl Ketone
mg	Milligram
ml	Millilitre
MRI	Magnetic Resonance Imaging
mRNA	Messenger Ribonucleic Acid
MTD	Maximum Tolerated Dose
MUGA	Multiple Gated Acquisition Scan
NCI	National Cancer Institute
od	Once Daily
ORR	Overall Response Rate
OS	Overall Survival
PBMC	Peripheral Blood Mononuclear Cells
PCN	Packaging Control Number
PD	Progression of Disease
PD-1	Programmed Death Receptor-1

PET	Positron Emission Tomography
PFS	Progression-Free Survival
PK	Pharmacokinetic
p.o.	Per os (oral route)
PS	Performance Status
PVC	Polyvinyl Chloride
RECIST	Response Evaluation Criteria In Solid Tumors
RL	Room Light
RT	Room Temperature
RVO	Retinal Vein Occlusion
SAE	Serious Adverse Events
SAP	Statistical Analysis Plan
SCC	Squamous Cell Carcinoma
sCD25	Soluble interleukin-2 receptor
SECOMBIT	Sequential Combo Immuno and target Therapy
sHGF	Soluble Hepatocyte Growth Factor
sICAM-1	Soluble Intercellular Adhesion Molecule-1
SUSAR	Suspected Unexpected Serious Adverse Reactions
sVEGF	Serum Vascular Growth factor
T3	Triiodotyroxine
T4	Tyroxine
TdP	Torsade de Points
TPFS	Total Progression-Free Survival
TSH	Thyroid Stimulating Hormone
ULN	Upper Limit of Normal
VAS	Visual Analog Scale
VEGF	Vascular Endothelial Growth Factor
WBC	White blood cell
WPAI:GH	Work Productivity and Activity Impairment: General Health

1. SCHEDULE ASSESSMENT

ARM A

ARM A<br>Combo Target (LGX818 450 mg p.o. od + MEK162. 45 mg p.obid) until PD; then Combo Immuno (nivolumab 1 mg/kg solution intravenously (IV) combined with ipilimumab 3 mg/kg solution IV every 3 weeks for 4 doses then nivolumab 3 mg/kg solution IV every 2 weeks) until progression of disease (PD).															
	Screening1/ Baseline1[1]	Treatment period[2]												End of Study Visit[3]	Follow-up Visit[4]
		Combo Target LGX818+MEK162				Screening 2/ Baseline2[13]	Combo Immuno								
							Nivolumab+Ipilimumab				Nivolumab				
**Day**	**-28 to 0**	**1**	**29**	**57**	**Q 28 days up to PD**	**-28 to 0**	**1**	**21**	**42**	**63**	**75**	**87**	**Q14 days up to PD**		
Informed Consent[5]	X														
Demographics	X														
Medical History	X														
Physical Examination & Vital Signs[6]	X	X	X	X	X	X	X	X	X	X	X	X	X	X	
ECOG Performance Status	X	X	X	X	X	X	X	X	X	X	X	X	X	X	
Hematology	X	X[8]	X	X	X	X	X	X	X	X	X	X	X	X	
Biochemistry	X	X[8]	X	X	X	X	X	X	X	X	X	X	X	X	
Cardiac/Muscle Enzymes[23]	X	X	X	X	X	X	X	X	X	X	X	X	X	X	
Urinalysis	X	Only if clinically indicated													
Endocrine Panel	X	X	X	X	X	X	X	X	X	X	X	X	X	X	
BRAF Mutation testing[22]	X														
Serum Pregnancy Test[9]	X	A serum pregnancy test to be performed every 6 (± 1) weeks during the treatment period												X	X
Tumor Assessments (CT/MRI)[10]	X		Every 8 weeks for the first year, every 12 weeks thereafter while the patient is on study												X
Quality of life and general Health Status questionnaires [24]	X	X	X	X	X	X	X	X	X	X		X	X[24]	X	X
Biomarkers study: Biopsy[11]	X	X (4W)			X (PD)		X (4W)						X (PD)		
Biomarkers study: Blood Drawn[12]	X	X (4W)			X (PD)	X	X (4W)						X (PD)		
Ophthalmologic exam[14]	X					X									
Dermatologic evaluation[15]	X	A dermatologic evaluation will be performed at screening/baseline and every 8 weeks while the patient is on study													
ECG[7]	X	An ECG will be performed at screening/baseline, at the 2nd baseline, at the 1st month and every 12 weeks while the patient is on study													
Echocardiogram[16]	X	An echo will be performed at screening/baseline, at the 2nd baseline, at the 1st month and q12 weeks while the patient is on study													
AEs/SAEs[17]	X	X	X	X	X	X	X	X	X	X	X	X	X	X	X
Concomitant medication[18]	X		X	X	X	X	X	X	X	X	X	X	X	X	X
Combo target dispensing & accountability[19]		X	X	X	X										
Combo Target Dosing Exception Diary[20]			X	X	X										
Combo immuno administration[21]							X	X	X	X	X	X	X		
Patient diary		X	X	X	X										

Table 1: Schedule of Assessments and Procedures in Arm A

Notes:
Day 1 = first dose of IMP
1. All screening/baseline assessments must be performed -28 to 0 days prior to the first administration of the IMP on Day 1 with the exception of the serum pregnancy test to be done within 24 hours. Results of tests or examinations (including tumor assessments) performed before obtaining informed consent and within the 28 Days prior to Day 1 may be used.
2. A window of 2 days prior to the scheduled visit date and 2 days after the scheduled visit date (- 2 days / + 2 days) is allowed for each visit, except for tumor evaluations for which a window of +/- 5 days will apply.
3. End of Study Visit (EOS) will be performed when the patient discontinues treatments regardless of when it occurs.
4. Follow up visit is to be performed within 28 days from discontinuation of treatments and thereafter every 12 weeks until 24 months for long term survival follow-up.
5. Informed consent must be obtained prior to perform any study procedure including screening/baseline assessments.
6. Height is taken at screening only. For patients with palpable/superficial lesions, clinical disease assessments by physical examination should be performed at baseline and throughout study treatment as clinically indicated. Color photographs with ruler/calipers will be taken at baseline and at all subsequent tumor assessment time points.
7. A 12-lead ECG will be performed at screening/baseline, at the 2nd baseline, at the 1st month and every 12 weeks while the patient is on study
8. Hematology and biochemistry assessments do not need to be repeated on Day 1 if performed within 7 days; if it is necessary to repeat these blood tests, the results must be known before the patient receives treatments to ensure inclusion/exclusion criteria related to these tests are met.
9. Serum pregnancy test to be performed within 24 hours prior to Day 1. A serum pregnancy test to be performed every 6 (± 1) weeks during the treatment period and at end of study and follow-up visit.
10. All measurable and non-measurable lesions must be documented at screening (within 28 days prior to randomization) and re-assessed at each subsequent tumor evaluation (every 8 weeks for the first year, every 12 weeks while the patient is on study). Tumor assessments with CT or MRI scans of the chest, abdomen, and pelvis will be performed until disease progression after the second combo treatment per RECIST v1.1. Imaging of the neck should be included if clinically indicated. In the event PET/CT scanner is used for tumor assessments, the CT portion of the PET/CT must meet criteria for diagnostic quality. All scans w ill be collected for a possible independent review.
11. Biopsy of tumoral lesions for the biological study will be performed at baseline 1, Week 4 and disease progression 1 (DP 1) during Combo Target therapy; and at Week 4 and disease progression 2 (DP 2) during Combo Immuno therapy. Biopsy of tumoral lesions will be mandatory at baseline 1 and at the first disease progression (PD1), and will be optional at the other time-points.
12. Serum, plasma (for circulating tumor DNA) and whole blood for PBMC processing will be taken at baseline 1, Week 4 and disease progression 1 (DP 1) during Combo Target therapy; and at baseline 2, Week 4 and disease progression 2 (DP 2) during Combo Immuno therapy. Whole blood for SNP will be taken at baseline 1 only.
13. The second baseline visit will be performed -28 to 0 days prior to the first administration of the Combo Immuno treatment on Day 1.Shift from the one combination therapy to the following one can be done (once the procedures of the new screening have been completed) in case of absence of AEs events from the prevsious combination therapy or in case of grade 1 AEs. In case of grade ≥ 2 AEs from the previous combination therapy, shift to the following combination therapy can be done only when the AE is resolved or has decreased in intensity to at least grade 1.
14. An ophthalmologic examination will be performed at the screening/baseline visit, at the 2nd baseline visit and then when clinically indicated.
15. A dermatologic evaluation will be performed at the screening/baseline visit and every8 weeks while the patient is on study.
16. An echocardiogram will be performed at screening/baseline, at the 2nd baseline, at the 1st month and every 12 weeks while the patient is on study
17. During screening AEs are not recorded in the eCRF unless they are SAEs which are related to protocol-mandated procedures. All AEs (including SAEs) must be recorded from the time of first treatments administration. After the last treatment, any new, non-serious AEs which the Investigator considers may be related to treatments should be reported according Section 7 – Safety instruction and guidance
18. All concomitant medications during the study started within 14 Days prior to the screening visit and up to the end of study visit must be recorded.
19. Combo Target (LGX818 450 mg p.o.od + MEK162 45 mg p.o. bid) will be administered until PD.
20. Patients will keep a diary to record ONLY those occasions when a Combo Target dose was missed. The patient will bring this diary with him/her to each study visit to allow missed doses to be recorded by the Investigator.
21. Combo Immuno (nivolumab 1 mg/kg solution IV combined with ipilimumab 3 mg/kg solution IV every 3 weeks for 4 doses then nivolumab 3 mg/kg solution IVevery 2 weeks) will be administered until PD following the Combo Target and subsequent PD.
22. Presence of BRAF V600E or V600K mutation in tumor tissue prior to enrollment
23. Troponin, Creatine Kinase (CK). If total CK ≥3 X ULN, then measure isoenzymes and myoglobin in blood or urine weekly
24. Quality of life and general health status defined by: Health-related quality of life (HRQoL), by means of the 30-item European Organisation for Research and Treatment of Care quality of life questionnaire (EORTC QLQ-C30); General health status, by means of the European Quality of Life 5-Dimensions (EQ-5D) questionnaire; Impairment of work productivity and activity, by means of the Work Productivity and Activity Impairment: General Health (WPAI:GH) questionnaire; every two administrations of nivolumab (i.e. every 28 days)

ARM B

ARM B<br>Combo Immuno (nivolumab 1 mg/kg solution IV combined with ipilimumab 3 mg/kg solution IV every 3 weeks for 4 doses then nivolumab 3 mg/kg solution IV every 2 weeks) until PD; then Combo Target (LGX818 450 mg p.o. od + MEK162 45 mg p.o. bid) until PD.															
	**Screening/ Baseline1[1]**	**Treatment period[2]**												**End of Study Visit[3]**	**Follow-up Visit[4]**
		**Combo Immuno**							**Baseline2[13]**	**Combo Target LGX818 + MEK162**					
		**Nivolumab+Ipilimumab**				**Nivolumab**									
**Day**	**-28 to 0**	**1**	**21**	**42**	**63**	**75**	**87**	**Q14 days up to PD**	**-28 to 0**	**1**	**29**	**57**	**Q 28 days up to PD**		
Informed Consent[5]	X														
Demographics	X														
Medical History	X														
Physical Examination & Vital signs[6]	X	X	X	X	X	X	X	X	X	X	X	X	X	X	
ECOG Performance Status	X	X	X	X	X	X	X	X	X	X	X	X	X	X	
Hematology	X	X[8]	X	X	X	X	X	X	X	X[8]	X	X	X	X	
Biochemistry	X	X[8]	X	X	X	X	X	X	X	X[8]	X	X	X	X	
Cardiac/Muscle Enzymes[23]	X	X	X	X	X	X	X	X	X	X	X	X	X	X	
Urinalysis	X	Only if clinically indicated													
Endocrine Panel	X	X	X	X	X	X	X	X	X	X	X	X	X	X	
BRAF Mutation testing[22]	X														
Serum Pregnancy Test[9]	X	A serum pregnancy test to be performed every 6 (± 1) weeks during the treatment period												X	X
Tumor Assessments (CT/MRI)[10]	X	Every 8 weeks for the first year, every 12 weeks thereafter while the patient is on study													X
Quality of life and general Health Status questionnaires [24]	X	X	X	X	X		X	X[24]	X	X	X	X	X	X	X
Biomarkers study: Biopsy[11]	X	X (4W)						X (PD)		X (4W)			X (PD)		
Biomarkers study: Blood Drawn[12]	X	X (4W)						X (PD)	X	X (4W)			X (PD)		
Ophthalmologic exam[14]	X							X							
Dermatologic evaluation[15]	X	A dermatologic evaluation will be performed at screening/baseline and every 8 weeks while the patient is on study													
Echocardiogram[16]	X	An echo will be performed at screening/baseline, at the 2nd baseline, at the 1st month and every 12 weeks while the patient is on study													
ECG[7]	X	An ECG will be performed at screening/baseline, at the 2nd baseline, at the 1st month and every 12 weeks while the patient is on study													
AEs/SAEs[17]	X	X	X	X	X	X	X	X	X	X	X	X	X	X	X
Concomitant medication[18]	X	X	X	X	X	X	X	X	X		X	X	X	X	X
Combo target dispensing & aqccountability[19]										X	X	X	X		
Combo Target Dosing Exception Diary[20]											X	X	X		
Combo immuno administration[21]		X	X	X	X	X	X	X							
Patient diary										X	X	X	X		

Table 2: Schedule of Assessments and Procedures in Arm B

Notes:
Day 1 = first dose of IMP

1. All screening/baseline assessments must be performed -28 to 0 days prior to the first administration of the IMP on Day 1 with the exception of the serum pregnancy test to be done within 24 hours. Results of tests or examinations (including tumor assessments) performed before obtaining informed consent and within the 28 Days prior to Day 1 may be used.
2. A window of 2 days prior to the scheduled visit date and 2 days after the scheduled visit date (- 2 days / + 2 days) is allowed for each visit, except for tumor evaluations for which a window of +/- 5 days will apply.
3. End of Study Visit (EOS) will be performed when the patient discontinues treatments regardless of when it occurs.
4. Follow up visit is to be performed within 28 days from discontinuation of treatments and thereafter every 12 weeks until 24 months for long term survival follow-up.
5. Informed consent must be obtained prior to perform any study procedure including screening/baseline assessments.
6. Height is taken at screening only. For patients with palpable/superficial lesions, clinical disease assessments by physical examination should be performed at baseline and throughout study treatment as clinically indicated. Color photographs with ruler/calipers will be taken at baseline and all subsequent tumor assessment time points.
7. A 12-lead ECG will be performed at screening/baseline, at the 2nd baseline, at the 1st month and every 12 weeks while the patient is on study
8. Hematology and biochemistry assessments do not need to be repeated on Day 1 if performed within 7 days; if it is necessary to repeat these blood tests, the results must be known before the patient receives treatments to ensure inclusion/exclusion criteria related to these tests are met.
9. Serum pregnancy test to be performed within 24 hours prior to Day 1. A serum pregnancy test to be performed every 6 (± 1) weeks during the treatment period and at end of study and follow-up visit.
10. All measurable and non-measurable lesions must be documented at screening (within 28 days prior to randomization) and re-assessed at each subsequent tumor evaluation (every 8 weeks for the first year, every 12 weeks while the patient is on study). Tumor assessments with CT or MRI scans of the chest, abdomen, and pelvis will be performed until disease progression after the second combo treatment per RECIST v1.1. Imaging of the neck should be included if clinically indicated. In the event PET/CT scanner is used for tumor assessments, the CT portion of the PET/CT must meet criteria for diagnostic quality. All scans will be collected for a possible independent review.
11. Biopsy of tumoral lesions for the biological study will be performed at baseline 1, Week 4 and disease progression 1 (DP 1) during Combo Immuno therapy; and at Week 4 and disease progression 2 (DP 2) during Combo Target therapy. Biopsy of tumoral lesions will be mandatory at baseline 1 and at the first disease progression (PD1), and will be optional at the other time-points.
12. Serum, plasma (for circulating tumor DNA) and whole blood for PBMC processing will be taken at baseline 1, Week 4 and disease progression 1 (DP 1) during Combo Immuno therapy; and at baseline 2, Week 4 and disease progression 2 (DP 2) during Combo Target therapy. Whole blood for SNP will be taken at baseline 1 only.
13. The second baseline visit will be performed -28 to 0 days prior to the first administration of the Combo Target treatment on Day 1.Shift from the one combination therapy to the following one can be done (once the procedures of the new screening have been completed) in case of absence of AEs events from the prevsious combination therapy or in case of grade 1 AEs. In case of grade ≥ 2 AEs from the previous combination therapy, shift to the following combination therapy can be done only when the AE is resolved or has decreased in intensity to at least grade 1.
14. An ophthalmologic examination will be performed at the screening/baseline visit, at the 2nd baseline visit and then when clinically indicated.
15. A dermatologic evaluation will be performed at the screening/baseline visit and every8 weeks while the patient is on study.
16. An echocardiogram will be performed at screening/baseline, at the 2nd baseline, at the 1st month and every 12 weeks while the patient is on study
17. During screening AEs are not recorded in the eCRF unless they are SAEs which are related to protocol-mandated procedures. All AEs (including SAEs) must be recorded from the time of first treatments administration. After the last treatment, any new, non-serious AEs which the Investigator considers may be related to treatments should be reported according Section 7 – Safety instruction and guidance
18. All concomitant medications during the study started within 14 Days prior to the screening visit and up to the end of study visit must be recorded.
19. Combo Target (LGX818 450 mg p.o.od + MEK162 45 mg p.o. bid) will be administered until PD.
20. Patients will keep a diary to record ONLY those occasions when a Combo Target dose was missed. The patient will bring this diary with him/her to each study visit to allow missed doses to be recorded by the Investigator.
21. Combo Immuno (nivolumab 1 mg/kg solution IV combined with ipilimumab 3 mg/kg solution IV every 3 weeks for 4 doses then nivolumab 3 mg/kg solution IV every 2 weeks) will be administered until PD following the Combo Target and subsequent PD.
22. Presence of BRAF V600E or V600K mutation in tumor tissue prior to enrolment.
23. Troponin, Creatine Kinase (CK). If total CK ≥3 X ULN, then measure isoenzymes and myoglobin in blood or urine weekly.
24. Quality of life and general health status defined by: Health-related quality of life (HRQoL), by means of the 30-item European Organisation for Research and Treatment of Care quality of life questionnaire (EORTC QLQ-C30); General health status, by means of the European Quality of Life 5-Dimensions (EQ-5D) questionnaire; Impairment of work productivity and activity, by means of the Work Productivity and Activity Impairment: General Health (WPAI:GH) questionnaire; every two administrations of nivolumab (i.e. every 28 days)

ARM C

ARM C

Combo Target (LGX818 450 mg p.o. od + MEK162 45 mg p.o. bid) for 8 weeks followed by Combo Immuno (nivolumab 1 mg/kg solution IV combined with ipilimumab 3 mg/kg solution IV every 3 weeks for 4 doses then nivolumab 3 mg/kg solution IV every 2 weeks) until PD; then Combo Target (LGX818 450 mg p.o. od + MEK162 45 mg p.o. bid) until PD.

	Screening/ Baseline1[1]	Treatment period[2]																End of Study Visit[3]	Follow-up Visit[4]
		Combo-Target			Baseline2[13]	Combo Immuno							Baseline3[14]	Combo Target LGX818+MEK162					
						Nivolumab+Ipilimumab				Nivolumab									
**Day**	**-28 to 0**	**1**	**29**	**57**		**1**	**21**	**42**	**63**	**75**	**87**	**Q 14 days up to PD**		**1**	**29**	**57**	**Q 28 days up to PD**		
Informed Consent[5]	X																		
Demographics	X																		
Medical History	X																		
Physical Examination & Vital Signs[6]	X	X	X	X	X	X	X	X	X	X	X	X	X	X	X	X	X	X	X
ECOG Performance Status	X	X	X	X	X	X	X	X	X	X	X	X	X	X	X	X	X	X	X
Hematology	X	X[8]	X	X	X	X	X	X	X	X	X	X	X	X	X	X	X	X	X
Biochemistry	X	X[8]	X	X	X	X	X	X	X	X	X	X	X	X	X	X	X	X	X
Cardiac/Muscle Enzymes[24]	X	X	X	X	X	X	X	X	X	X	X	X	X	X	X	X	X		
Urinalysis	X	Only if clinically indicated																	
Endocrine Panel	X	X	X	X	X	X	X	X	X	X	X	X	X	X	X	X	X		
BRAF Mutation testing[23]	X																		
Serum Pregnancy Test[9]	X	A serum pregnancy test to be performed every 6 (± 1) weeks during the treatment period																X	X
Tumor Assessments (CT/MRI)[10]	X		Every 8 weeks for the first year, every 12 weeks thereafter while the patient is on study																X
Quality of life and general Health Status questionnaires [24]	X	X	X	X	X	X	X	X	X		X	X[25]	X	X	X	X	X	X	X
Biomarkers study: Biopsy[11]	X					X (4W)						X (PD)		X (4W)			X (PD)		
Biomarkers study: Blood Drawn[12]	X					X (4W)						X (PD)		X (4W)			X (PD)		
Ophthalmologic exam[15]	X				X								X						
Dermatologic evaluation[16]	X	A dermatologic evaluation will be performed at screening/baseline and every 8 weeks while the patient is on study																	
Echocardiogram[17]	X	An echo will be performed at screening/baseline, at the 2nd and 3rd baseline, at the 1st month and every 12 weeks while the patient is on study																	
ECG[7]	X	An ECG will be performed at screening/baseline, at the 2nd baseline, at the 1st month and every 12 weeks while the patient is on study																	X
AEs/SAEs[18]	X	X	X	X	X	X	X	X	X	X	X	X	X	X	X	X	X	X	X
Concomitant medication[19]	X		X	X	X	X	X	X	X	X	X	X	X	X	X	X	X	X	X
Combo target dispensing & acountability[20]		X	X	X										X	X	X	X		
Combo Target Dosing Exception Diary[21]			X	X										X	X	X	X		
Combo immuno administration[22]						X	X	X	X	X	X	X							
Patient diary		X	X	X										X	X	X	X		

Table 3: Schedule of Assessments and Procedures in Arm C

Notes:
Day 1 = first dose of IMP
1. All screening/baseline assessments must be performed -28 to 0 days prior to the first administration of the IMPon Day 1 with the exception of the serum pregnancy test to be done within 24 hours. Results of tests or examinations (including tumor assessments) performed before obtaining informed consent and within the 28 Days prior to Day 1 may be used.
2. A window of 2 days prior to the scheduled visit date and 2 days after the scheduled visit date (- 2 days / + 2 days) is allowed for each visit, except for tumor evaluations for which a window of +/- 5 days will apply.
3. End of Study Visit (EOS) will be performed when the patient discontinues treatments regardless of when it occurs.
4. Follow up visit is to be performed within 28 days from discontinuation of treatments and thereafter every 12 weeks until 24 months for long term survival follow-up.
5. Informed consent must be obtained prior to perform any study procedure including screening/baseline assessments.
6. Height is taken at screening only. For patients with palpable/superficial lesions, clinical disease assessments by physical examination should be performed at baseline and throughout study treatment as clinically indicated. Color photographs with ruler/calipers will be taken at baseline and at all subsequent tumor assessment time points.
7. A 12-lead ECG will be performed at screening/baseline, at the 2nd baseline, at the 1st month and every 12 weeks while the patient is on study
8. Hematology and biochemistry assessments do not need to be repeated on Day 1 if performed within 7 days; if it is necessary to repeat these blood tests, the results must be known before the patient receives treatments to ensure inclusion/exclusion criteria related to these tests are met.
9. Serum pregnancy test to be performed within 24 hoursprior to Day 1. A serum pregnancy test to be performed every 6 (± 1) weeks during the treatment period and at end of study and follow-up visit.
10. All measurable and non-measurable lesions must be documented at screening (within 28 days prior to randomization) and re-assessed at each subsequent tumor evaluation (every 8 weeks for the first year, every 12 weeks while the patient is on study). Tumor assessments with CT or MRI scans of the chest, abdomen, and pelvis will be performed until disease progression after the second combo treatment per RECIST v1.1. Imaging of the neck should be included if clinically indicated. In the event PET/CT scanner is used for tumor assessments, the CT portion of the PET/CT must meet criteria for diagnostic quality. All scans will be collected for a possible independent review.
11. Biopsy of tumoral lesions for the biological study will be performed at baseline 1, Week 4 and disease progression 1 (DP 1) during Combo Immuno therapy; and at Week 4 and disease progression 2 (DP 2) during the 2nd Combo Target therapy. Biopsy of tumoral lesions will be mandatory at baseline 1 and at the first disease progression (PD1), and will be optional at the other time-points.
12. Serum, plasma (for circulating tumor DNA) and whole blood for PBMC processing will be taken at baseline 1, at baseline 2, Week 4 and disease progression 1 (DP 1) during Combo Immuno therapy; and at baseline 3, Week 4 and disease progression 2 (DP 2) during the 2nd Combo Target therapy. Whole blood for SNP will be taken at baseline 1 only.
13. The second baseline visit will be performed -28 to 0 days prior to the first administration of the Combo Immuno treatment on Day 1.Shift from the one combination therapy to the following one can be done (once the procedures of the new screening have been completed) in case of absence of AEs events from the prevsious combination therapy or in case of grade 1 AEs. In case of grade ≥ 2 AEs from the previous combination therapy, shift to the following combination therapy can be done only when the AE is resolved or has decreased in intensity to at least grade 1.
14. The third baseline visit will be performed -28 to 0 days prior to the first administration of the second Combo Target treatment on Day 1.See rules for switching as above.
15. An ophthalmologic examination will be performed at the screening/baseline visit, at the 2nd baseline visit, at the 3rd baseline visit and then when clinically indicated.
16. A dermatologic evaluation will be performed at the screening/baseline visit and every8 weeks while the patient is on study.
17. An echocardiogram will be performed at screening/baseline, at the 2nd and 3rd baseline, at the 1st month and every 12 weeks while the patient is on study
18. During screening AEs are not recorded in the eCRF unless they are SAEs which are related to protocol-mandated procedures. All AEs (including SAEs) must be recorded from the time of first treatments administration. After the last treatment, any new, non-serious AEs which the Investigator considers may be related to treatments should be reported according Section 7 – Safety instruction and guidance
19. All concomitant medications during the study started within 14 Days prior to the screening visit and up to the end of study visit must be recorded.
20. Combo Target (LGX818 450 mg p.o.od + MEK162. 45 mg p.o bid) will be administered for initial 8 weeks and then will be administered until PD following the Combo Immuno and subsequent PD.
21. Patients will keep a diary to record ONLY those occasions when a Combo Target dose was missed. The patient will bring this diary with him/her to each study visit to allow missed doses to be recorded by the Investigator.
22. Combo Immuno (nivolumab 1 mg/kg solution IV combined with ipilimumab 3 mg/kg solution IV every 3 weeks for 4 doses then nivolumab 3 mg/kg solution IV every 2 weeks) will be administered until PD following the Combo Target and subsequent PD.
23. Presence of BRAF V600E or V600K mutation in tumor tissue prior to enrollment
24. Troponin, Creatine Kinase (CK). If total CK ≥3 X ULN, then measure isoenzymes and myoglobin in blood or urine weekly.
25. Quality of life and general health status defined by: Health-related quality of life (HRQoL), by means of the 30-item European Organisation for Research and Treatment of Care quality of life questionnaire (EORTC QLQ-C30); General health status, by means of the European Quality of Life 5-Dimensions (EQ-5D) questionnaire; Impairment of work productivity and activity, by means of the Work Productivity and Activity Impairment: General Health (WPAI:GH) questionnaire; every two administrations of nivolumab (i.e. every 28 days)

2. BACKGROUND AND RATIONALE

2.1 Overview of disease epidemiology and current treatment

2.1.1 Epidemiology of melanoma

Melanoma is the most serious form of skin cancer and strikes adults of all ages. Both incidence of melanoma and mortality rate are rapidly increasing throughout the world, constituting a significant and growing health burden (*Ferlay et al, 2010*). The worldwide incidence of melanoma in 2010 was estimated at 208,251 (*Jemal et al, 2011*), and approximately 76,250 men and women were diagnosed with melanoma in 2012 in the United States alone (*American Cancer Society, 2012*)

About 80% of melanomas are detected in a localized stage, and can be treated with surgical resection. When detected early, the 5-year survival rate of melanoma is above 90%; however, when melanoma is diagnosed after distant metastasis, the prognosis is by contrast very poor.

The 5-year survival rate decreases to 15% with a median survival between 8 and 9 months (Jemal et al, 2011). Advanced melanoma is one of the most aggressive human malignancies. In 2012, an estimated 9,000 melanoma patients in the US have died from their disease (*American Cancer Society, 2012*).

2.1.2 Treatment options in patients with advanced melanoma

For the last 40 years, treatment progresses in advanced melanoma have been largely stagnant, with traditional options, such as chemotherapy, lacking substantial efficacy. This has changed in 2011 with the Food and Drug Administration (FDA) approval of novel immunotherapy, ipilimumab (Yervoy®, Bristol-Myers Squibb), an antibody against the cytotoxic T-lymphocyte antigen-4 (anti-CTLA-4), although melanoma that has spread to distant sites remains rarely curable.

The discovery of the genetic underpinnings of melanoma and their characterization has uncovered potential targets for therapy, B-raf murine sarcoma viral oncogene homolog B1 (BRAF) mutations being principal among them. BRAF mutations have been identified as an important target in melanoma and discovery of oncogenic BRAF mutations highlighted the significant role of BRAF kinase in signalling pathways that control cellular proliferation. More than half of patients with metastatic melanoma have mutations that keep the BRAF protein constantly activated.

Mutations in BRAF exon 15 account for over 95% of activating BRAF mutations in metastatic melanoma. The two most common BRAF V600 mutations V600E and V600K have been reported to account for anywhere between 66-91% and 7-30% of BRAF V600 mutant metastatic melanoma patients respectively. Both BRAF

V600E and V600K mutant patients account for 92-98% of all BRAF V600 mutant metastatic melanoma patients (*Colombino et al, 2012*; *Jakob et al, 2011*; *Greaves et al, 2013*). These mutations constitutively activate BRAF and downstream signal transduction in the RAF/MEK/ERK pathway, which signals for cancer cell proliferation and survival. Moreover, oncogenic BRAF mutations generally correlate with poor prognosis in a variety of different tumor types.

The selective BRAF inhibitor vemurafenib was approved by the FDA (17 August 2011) for the treatment of patients with unresectable or metastatic melanoma with the BRAF V600E mutation as detected by an FDA approved test. In February 2012, vemurafenib received approval in the European Union (EU) as a monotherapy for the treatment of adult patients with BRAF V600 mutation-positive unresectable or metastatic melanoma.

Although studies have shown that BRAF-targeted therapy is effective in BRAF mutant melanoma, data also indicate that the duration of response is often short lived, with resistance developing quickly, within approximately 6 months (*Flaherty et al, 2010*; *Sosman et al, 2012*; *Chapman et al, 2011*; *Hauschild et al, 2012*). Re-activation of mitogen activated protein kinase (MAPK) signalling occurs in the majority of cases of acquired resistance to BRAF inhibitors. In an attempt to delay resistance to BRAF inhibition, the combination of a selective BRAF- and a MEK1/2-inhibitor is currently being investigated by several clinical trials in patients with advanced BRAF mutant melanoma. Results of the first such phase I/II study [NCT01072175] of the combination of investigational agents dabrafenib and trametinib, and of the phase Ib dose escalation study [NCT01271803] of vemurafenib (Zelboraf®, Roche), in combination with an investigational MEK inhibitor GDC-0973 (Roche), have been recently reported (*Flaherty et al, 2012*). Median PFS was 9.4 months in the dabrafenib plus trametinib group vs. 5.8 months in the dabrafenib group (*Flaherty et al, 2012*).
Despite recent treatment breakthroughs, advanced melanoma remains an aggressive disease with a poor prognosis. There is a need to develop new treatment regimens to improve the duration of response and delay the emergence of resistance, expanding the therapeutic options for patients with unresectable or metastatic melanoma, including those with BRAF V600 mutation.

2.2 Introduction to investigational treatments and other study treatment(s)

2.2.1 LGX818

LGX818 (encorafenib) is a highly selective ATP-competitive small molecule RAF kinase inhibitor, which suppresses the RAF/MEK/ERK pathway in tumor cells expressing BRAF V600. The narrow kinase profile and potent anti-proliferative activity of LGX818 translates into a very wide therapeutic index in vivo.

LGX818 was evaluated in rats and Cynomolgus monkeys in toxicology studies ranging from 1 to 4 weeks in duration. Overall, LGX818 was well tolerated at doses at which tumor regression was observed. Significant toxicities were mainly observed in the female rat at the highest dose of 400 mg/kg/day, a dose well above the MTD. Other findings included hyperplasia and hyperkeratosis in the skin (plantar surface of feet) and non-glandular stomach in rat, which was apparent at all dose levels and presented with recovery 4 weeks after stopping treatment, and an absence of the later stages of spermatid maturation in the male rats. Preclinical cardiovascular safety pharmacology data did not indicate a clinical risk for QTc prolongation based on the findings of the hERG assay and ECG evaluation in the GLP 4-week monkey study. Also, there were no clinical signs in the 4-week GLP rat and monkey studies that would indicate an effect on the central nervous system or respiratory system. No teratogenicity studies have been completed to date.For further details on non-clinical pharmacology and toxicology, please refer to the current LGX818 Investigator’s Brochure.

The results of a phase I study assessing the maximum tolerated dose (MTD) and of the recommended dose phase II study of LGX818 in patients with locally advanced and metastatic BRAF mutated melanoma have confirmed the potency and the wide therapeutic index of LGX818 with clinical efficacy observed from the lowest tested dose of 50 mg/day up to the MTD of 450 mg/day.For further details, please refer to the current LGX818 Investigator’s Brochure.

2.2.2 MEK162

MEK162 (binimetinib), previously named ARRY 438162, is a potent and selective allosteric, ATP (Adenosine Tri-Phosphate) non-competitive inhibitor of MEK1/2 that is active in inhibiting pERK and growth of BRAF mutant cancer cells in the low nanomolar range.

Acute, subchronic, chronic and reproductive toxicity, genotoxicity and phototoxicity studies were completed to support the chronic administration of MEK162 to adult cancer patients. The toxic effects of MEK inhibitors in humans are similar to the toxic effects observed in monkeys. The toxic effects include gastro-intestinal intolerance and diarrhea, rash, central serous retinopathy (only seen in humans) and retinal vein occlusion (rarely seen in humans). In vitro and in vivo phototoxicity studies conducted in mice indicate that MEK162 has a very

low risk of weak phototoxic potential at therapeutic doses. Furthermore, there has been no evidence of phototoxicity or photosensitivity in humans being treated with MEK162 for cancer or for rheumatoid arthritis. Given the embryo-lethal effects seen in rats and rabbits and the teratogenic effects seen in rabbits, MEK162 should not be used in pregnant women. Women of child-bearing potential must be advised to use highly effective contraception methods.

For further details on non-clinical pharmacology and toxicology, please refer to the current MEK162 Investigator's Brochure.

In oncology settings, MEK162 is currently being investigated both as a single agent and in combination with PI3K or RAF inhibitors in patients with selected advanced or metastatic solid tumors, including biliary cancer, colorectal cancer and melanoma.

The safety and efficacy of MEK162 as a single agent in patients with advanced melanoma have been evaluated in one Phase II study (CMEK162X2201), in which 45 mg and 60 mg bid dose levels have been investigated. In study CMEK162X2201, the most frequently treatment-related occurring events (≥ 20%) were dermatitis acneiform, diarrhea, peripheral edema, increase in creatin-kinase (CK) levels, nausea, fatigue and rash. The most common treatment-related Grade 3-4 AEs were CK increased, dermatitis acneiform and diarrhoea.

In the BRAF mutant population, the ORR was 20% (8 PR, 2 confirmed and 6 unconfirmed), and the median PFS was 3.6 months (CI: 2.0; 3.8) among the 41 patients treated with MEK162 at 45 mg bid (i.e. the recommended dose). In patients not previously treated with a BRAF inhibitor (n=34) the median PFS was 3.7 months. The majority of these patients received at least one prior anticancer treatment (*Ascierto et al, 2013*).For further details, please refer to the current MEK162 Investigator's Brochure.

2.2.3 LGX818 and MEK162 combination

PK data from the clinical study in BRAF mutated cancer patients up to doses of 600 mg od LGX818 in combination with 45 mg bid MEK162 show that, although the concentrations of MEK162 tended to be on the higher end of the variability range, the PK parameter of MEK162 remained unchanged when administered with 50 to 600 mg LGX818. Exposure to LGX818 on Day 15, as determined by Cmax and AUC, was 30 to 70% less compared with Day 1, likely due to CYP induction by LGX818 (auto-induction). PK characteristics of LGX818 when co-administered with MEK162 are similar to those observed when given as single agent.

Although clinical data from ongoing studies with LGX818 and MEK162 as single agents suggest that overlapping toxicities for the proposed combination, including effects on the skin (e.g. rash) and gastro-intestinal system (e.g. nausea) may potentially be dose-limiting, initial reports with a similar class of compounds have shown that selective BRAF inhibitor combines safely with MEK inhibitor with a decreased occurrence of skin

toxicities (rash, SCC). These data suggest that the combination of LGX818 with MEK162 may have an improved safety profile compared to the respective single agent therapies.

Soon after the initiation of clinical oncology trials with MEK inhibitors, it was observed that some participants developed an eye condition resembling central serous chorioretinopathy. A recently published article (Umer-Bloch et al, 2014) has examined the clinical features and management of MEK inhibitor-associated retinal syndromes in patients with advanced cutaneous melanoma treated with MEK162 in different Phase 1b or 2 clinical trials. Twenty patients on MEK162 monotherapy and 5 on MEK162 plus LGX818 underwent ophthalmological examinations at regular intervals, including determination of best corrected visual acuity, perimetry, colour vision testing, dilated fundus examination, and multimodal imaging. Grade 1-2 bilateral retinopathies with multiple lesions were observed in 13 of 20 patients on MEK162 monotherapy and in 2 of 5 patients on MEK162 plus LGX818. Retinopathy events appeared during the first 4 weeks, and in some cases, during the first few days of treatment. Patients reported mild and only short-lived visual symptoms. Optical coherence tomography revealed neuroretinal elevations. Central retinal thickness and volume showed dose-dependent increases after the start of treatment, followed by a marked decrease despite continued treatment, which was associated with symptom resolution. No vascular abnormalities were found with fluorescein and indocyanine green angiography. This analysis showed that treatment with MEK162 monotherapy or plus LGX818 induced transient retinopathy with multiple bilateral lesions in some patients. MEK162-induced retinopathy was usually mild, self-limiting, and tolerable as visual function was not seriously impaired.

2.2.4 Nivolumab

Nivolumab is a fully human, IgG4 (kappa) isotype, mAb that binds programmed death receptor-1 (PD-1). Blockade of PD-1 by monoclonal antibodies (mAbs) can enhance the anti--tumor immune response and result in tumor rejection.

In a recent study (*Topalian et al, 2014*), 107 patients with advanced melanoma enrolled between 2008 and 2012 received IV nivolumab in an outpatient setting every 2 weeks in 8-week treatment cycles for up to 96 weeks and were observed for overall survival, long-term safety, and response duration after treatment discontinuation. Nivolumab was administered at 1, 3, or 10 mg/kg during dose escalation. After completion of dose escalation, each dose cohort was expanded to accrue approximately 16 patients. Additional melanoma cohorts randomly assigned to 0.1, 0.3, and 1.0 mg/kg were enrolled. Median overall survival in nivolumab-treated patients (62% with two to five prior systemic therapies) was 16.8 months, and 1- and 2-year survival rates were 62% and 43%, respectively. Among 33 patients with objective tumor regressions (31%), the Kaplan-Meier estimated median response duration was 2 years. Seventeen patients discontinued therapy for reasons other than disease progression, and 12 (71%) of 17 maintained responses off-therapy for at least 16 weeks (range, 16 to 56+ weeks).

Objective response and toxicity rates were similar to those reported previously; in an extended analysis of all 306 patients treated on this trial (including those with other cancer types), exposure-adjusted toxicity rates were not cumulative.

In this study, OS following nivolumab treatment in patients with advanced treatment-refractory melanoma compares favourably with that in literature studies of similar patient populations. Responses were durable and persisted after drug discontinuation. Long-term safety was acceptable.

Based on its mechanism of action and data from animal studies, nivolumab can cause fetal harm when administered to a pregnant woman. In animal reproduction studies, administration of nivolumab to cynomolgus monkeys from the onset of organogenesis through delivery resulted in increased abortion and premature infant death. Advise pregnant women of the potential risk to a fetus. Advise females of reproductive potential to use effective contraception during treatment with nivolumab and for at least 5 months after the last dose of nivolumab.

2.2.5 Ipilimumab

Ipilimumab is a recombinant, human monoclonal antibody that binds to the CTLA-4. Blockade of CTLA-4 augments T-cell activation and proliferation and ipilimumab works via T-cell mediated anti-tumor immune responses.

A variety of studies have demonstrated a clinically meaningful and statistically significant survival benefit with the use of ipilimumab in advanced melanoma.

A survival update at a follow-up of >5 years (5.5 to 6 years) for patients with advanced melanoma who previously received ipilimumab in phase II clinical trials has been recently published (*Lebbè et al, 2014*). Patients who previously received ipilimumab 0.3, 3, or 10 mg/kg in one of six phase II trials have been evaluated. Upon enrolment, patients initially received ipilimumab retreatment, extended maintenance therapy, or were followed for survival only. OS rates were evaluated in patients from 4 studies, and safety and best overall response during ipilimumab retreatment at 10 mg/kg were assessed in one study. Five-year OS rates for previously treated patients who received ipilimumab induction at 0.3, 3, or 10 mg/kg were 12.3%, 12.3% to 16.5%, and 15.5% to 28.4%, respectively. Five-year OS rates for treatment-naive patients who received ipilimumab induction at 3 or 10 mg/kg were 26.8% and 21.4% to 49.5%, respectively. Little to no change in OS was observed from year 5 up to year 6. The objective response rate among retreated patients was 23%. Grade 3/4 immune-related adverse events occurred in 25%, 5.9%, and 13.2% of retreated patients who initially received ipilimumab 0.3, 3, and 10 mg/kg, with the most common being observed in the skin (4.2%, 2.9%, 3.8%) and gastrointestinal tract (12.5%, 2.9%, 3.8%), respectively. At a follow-up of 5 to 6 years, ipilimumab continues to

demonstrate durable, long-term survival in a proportion of patients with advanced melanoma. In some patients, ipilimumab retreatment can re-establish disease control with a safety profile that is comparable to that observed during ipilimumab induction.

There are no adequate and well-controlled studies of ipilimumab in pregnant women. Ipilimumab is classified in pregnancy category C. Ipilimumab should be used during pregnancy only if the potential benefit justifies the potential risk to the fetus. In a combined study of embryo-fetal and peri-postnatal development, pregnant cynomolgus monkeys received ipilimumab every 3 weeks from the onset of organogenesis in the first trimester through parturition, at exposure levels either 2.6 or 7.2 times higher by AUC than the exposures at the clinical dose of 3 mg/kg of ipilimumab. No treatment-related adverse effects on reproduction were detected during the first two trimesters of pregnancy. Beginning in the third trimester, the ipilimumab-treated groups experienced higher incidences of severe toxicities including abortion, stillbirth, premature delivery (with corresponding lower birth weight), and higher incidences of infant mortality in a dose-related manner compared to controls. Human IgG1 is known to cross the placental barrier and ipilimumab is an IgG1; therefore, ipilimumab has the potential to be transmitted from the mother to the developing fetus

2.2.6 Nivolumab and ipilimumab combination

Recently, the results of a phase I study about the combination ipilimumab plus nivolumab in patients with advances melanoma have been reported (*Wolchock et al, 2013*). In this study, IV doses of nivolumab and ipilimumab were administered every 3 weeks for 4 doses, followed by nivolumab alone every 3 weeks for 4 doses (concurrent regimen). The combined treatment was subsequently administered every 12 weeks for up to 8 doses. In a sequenced regimen, patients previously treated with ipilimumab received nivolumab every 2 weeks for up to 48 doses. A total of 53 patients received concurrent therapy with nivolumab and ipilimumab, and 33 received sequenced treatment. The ORR for all patients in the concurrent-regimen group was 40%. Evidence of clinical activity (conventional, unconfirmed, or immune-related response or stable disease for ≥24 weeks) was observed in 65% of patients. At the maximum doses that were associated with an acceptable level of adverse events (nivolumab at a dose of 1 mg per kilogram of body weight and ipilimumab at a dose of 3 mg/kg), 53% of patients had an objective response, all with tumor reduction of 80% or more. Grade 3 or 4 adverse events related to therapy occurred in 53% of patients in the concurrent-regimen group but were qualitatively similar to previous experience with monotherapy and were generally reversible. Among patients in the sequenced-regimen group, 18% had grade 3 or 4 adverse events related to therapy and the objective-response rate was 20%. In this study, concurrent therapy with nivolumab and ipilimumab had a manageable safety profile and provided clinical activity that appears to be distinct from that in published data on monotherapy, with rapid and deep tumor regression in a substantial proportion of patients.

2.3 Study rationale

The combination BRAF inhibitor plus MEK inhibitor seems to be more effective in the V600 BRAF mutated advanced melanoma patients compared to treatment with the BRAF inhibitors alone. In fact, a phase I-II study (*Flaherty et al, 2012*) showed a better ORR and PFS in the combination arm (dabrafenib plus trametinib) respect to the single agent treatment (dabrafenib): 76% and 9.4 months versus 54% and 5.8 months respectively. Another phase I study with a similar combination (vemurafenib plus cobimetinib) showed an ORR of 85% in vemurafenib-naïve patients (*Martinez Garcia et al, 2012*)

The above reported phase I study on the combination ipilimumab plus nivolumab (*Wolchock et al, 2013*) has shown that more than half of patients treated at the selected schedule (ipilimumab 3 mg/kg and nivolumab 1 mg/kg) had an objective response, all with tumor reduction of ≥ 80%. Reponses were durable, although longer follow-up is needed.

A recent phase I study has shown a high rate of liver toxicity with the combo ipilimumab plus vemurafenib (*Ribas et al, 2013*) which makes difficult a combination with these two different drugs. Moreover, a better efficacy of the sequencing treatment BRAF inhibitors/ipilimumab vs. the single agent treatment was also observed; for this reason it was also suggested to start immunotherapy treatment in the BRAF V600 mutated melanoma population as first option, in order to increase the percentage of patients who can benefit from the sequencing (*Ascierto et al, 2012*; *Ascierto et al, 2013*), considering the possibility of a fast progression of the disease after the BRAF inhibitors treatment (*Ascierto et al, 2012*).

Taking into account these considerations, it seems impossible to think to combine all the four compounds (the target agents and immunomodulating monoclonal antibodies). The risk of a high rate of toxicity is realistic and would render this approach inapplicable.

Sequencing with these different combinations seems to be more feasible. However, also in this case it would be important to start with the best combination in order to give to the patients the best chance to increase the overall survival.
The aim of this prospective randomized phase II study is to evaluate the sequencing of these different combinations and evaluate which is the best of these approaches.

3. STUDY AIMS AND DESIGN

This prospective randomized phase II study is aimed at evaluating the effects of the sequencing of the two different tested combinations (Combo Target: LGX818 450 mg p.o. od + MEK162 45 mg p.o. bid; Combo Immuno: nivolumab 1 mg/kg solution IV combined with ipilimumab 3 mg/kg solution IV every 3 weeks for 4 doses then nivolumab 3 mg/kg solution IV every 2 weeks) and at evaluating which is the best of the three tested approaches.

3.1 Objectives of the study

Primary Objectives

The primary objective is to define the best sequencing combination treatment in primary efficacy variable OS.

Secondary Objectives

The secondary objectives are to evaluate the effects of the two sequencing combination treatments on:

- Total PFS;
- Percentage of patients alive at 2 and 3 years;
- Best overall response rate (BORR);
- Duration of response (DoR);
- Biological markers (biomarkers ancillary study).
- Toxicity of the investigational medicinal products (IMPs).
- Quality of life and general health status defined by:
  - Health-related quality of life (HRQoL), by means of the 30-item European Organisation for Research and Treatment of Care quality of life questionnaire (EORTC QLQ-C30);
  - General health status, by means of the European Quality of Life 5-Dimensions (EQ-5D) questionnaire;
  - Impairment of work productivity and activity, by means of the Work Productivity and Activity Impairment: General Health (WPAI:GH) questionnaire;

Biological markers (biomarkers ancillary study)

The objective of the biomarkers ancillary study (to be conducted in a subgroup of approximately 80-90 patients) is to focus on understanding mechanisms of action/resistance. In particular, the ancillarystudy will inform how to sequence targeted RAF/MEK agents with immunotherapy agents (i.e. ipilumumab and nivolumab) in melanoma and will be hypothesis-generating only.

3.2 Endpoints of the study

Primary Endpoint

OS is primary efficacy endpoint of the study. OS will be calculated as the time from the date of randomization until the date of death from any cause.

Any patient not know to have died at the time of data analysis will be censored at the time of the last recorded date on which the patient was know to be alive.

Secondary Endpoints

- Total PFS, calculated from the date of randomization until the date of the second progression (i.e. the progression to second treatment); any progression or death will be considered as an event if patient cannot complete treatment sequence;
- Percentage of patients alive at 2 and 3 years;
- Best overall response rate (BORR);
- Duration of response (DoR) calculated as the time from the date of the first documented response (CR or PR) until the date of the first documented progression or death due to underlying cancer. If the patient with a CR or PR has no progression or death due to underlying cancer, the patient will be censored at the date of last adeguate tumor assessment;
- Health-related quality of life (HRQoL), by means of the 30-item European Organisation for Research and Treatment of Care quality of life questionnaire (EORTC QLQ-C30);
- General health status, by means of the European Quality of Life 5-Dimensions (EQ-5D) questionnaire;
- Impairment of work productivity and activity, by means of the Work Productivity and Activity Impairment: General Health (WPAI:GH) questionnaire;
- Biological markers (biomarkers ancillary study).

Biological markers (Biomarkers ancillary study)

<u>Tumor tissue biomarkers:</u>

- Immune status: CD3, CD8, and CD4 T cells; Activated T cells; T regulatory cells; Dendritic cells;
- Resistance to immunotherapy agents: Checkpoint receptors/ligands; myeloid-derived suppressor cells (MDSCs);
- Resistance to targeted agents: aberrations in MEK/PI3K pathways, cytokines that interact with tyrosine kinase receptors (VEGF, HGF and their cognate receptors);
- Mutational load and neoantigen profile.

<u>Peripheral blood biomarkers:</u>

- Immune status/"Resistance" to immunotherapy agents: Activated T cells; Memory/Exhausted T cells; T regulatory cells; MDSCs; Inflammatory response; C-reactive protein (CRP); TCR Sequencing/Gene Expression analysis;
- Response/Resistance to targeted agents: Apoptotic tumor cells (as measured by circulating tumor DNA); soluble hepatocyte growth factor (sHGF); serum vascular endothelial growth factor (sVEGF); soluble interleukine-2 receptor (sCD25);
- Immunotherapy SNP Panel will be also assessed at baseline.

<table>
<tr><th>Study Aim</th><th>Objectives</th><th>End point</th></tr>
<tr><td>Primary</td><td>The primary objective of this study is to define the best sequencing combination treatment in primary efficacy variable OS.</td><td>Overall Survival (OS) will be calculated as the time from the date of randomization until the date of death from any cause. Any patient not kinow to have died at the time of data analysis will be censored at the time of the last recorded date on which the patient was know to be alive.</td></tr>
<tr><td>Secondary</td><td>The secondary objectives of the study are to evaluate the effects of the two sequencing combination treatments on:
• Total PFS;
• Percentage of patients alive at 2 and 3 years;
• Best overall response rate (BORR);
• Duration of response (DoR);
• Biological markers (biomarkers ancillary study);
• Toxicity of the investigational medicinal products (IMPs);
• Quality of life and general health status defined by:
- Health-related quality of life (HRQoL), by means of the 30-item European Organisation for Research and Treatment of Care quality of life questionnaire (EORTC QLQ-C30);
- General health status, by means of the European Quality of Life 5-Dimensions (EQ-5D) questionnaire;
- Impairment of work productivity and activity, by means of the Work Productivity and Activity Impairment: General Health (WPAI:GH) questionnaire.</td><td>• Total PFS, calculated from the date of randomization to the date of the second progression (i.e. the progression to second treatment); any progression or death will be considered as an event if patient cannot complete treatment sequence;
• Percentage of patients alive at 2 and 3 years;
• Best overall response rate (BORR);
• Duration of response (DoR), calculated as the time from the date of first documented response (CR o PR) until the date of the first documented progression or death due to underlying cancer. If a patient with a CR o PR has no progression or death due to underlying cancer, the patient will be censored at the date of last adeguate tumor assessment;
• Health-related quality of life (HRQoL), by means of the 30-item European Organisation for Research and Treatment of Care quality of life questionnaire (EORTC QLQ-C30);
• General health status, by means of the European Quality of Life 5-Dimensions (EQ-5D) questionnaire;
• Impairment of work productivity and activity, by means of the Work Productivity and Activity Impairment: General Health (WPAI:GH) questionnaire;
• Biological markers (biomarkers ancillary study).</td></tr>
</table>

Study Aim	Objectives	End point
**Biomarkers Ancillary Study**	The objective of the biomarkers ancillary study is to focus on understanding mechanisms of action/resistance. In particular, the ancillary study:<br>• Will inform how to sequence targeted RAF/MEK agents with immunotherapy agents (i.e. ipilumumab and nivolumab) in melanoma;<br>• Will be hypothesis-generating only.	Tumor tissue biomarkers:<br>• Immune status: CD3, CD8, CD4, CD45RO and CD11c T cells; Activated T cells; T regulatory cells; Dendritic cells;<br>• Resistance to immunotherapy agents: Checkpoint receptors/ligands; myeloid-derived suppressor cells (MDSCs);<br>• Resistance to targeted agents: aberrations in MEK/PI3K pathways; mRNA expression (nanostring method).<br>Peripheral blood biomarkers:<br>• Immune status/"Resistance" to immunotherapy agents: Activated T cells; Memory/Exhausted T cells; T regulatory cells; MDSCs; Inflammatory response; CRP; TCR Sequencing/Gene Expression analysis;<br>• Response/Resistance to targeted agents: Apoptotic tumor cells (as measured by circulating tumor DNA); soluble hepatocyte growth factor (sHGF); serum vascular endothelial growth factor (sVEGF); soluble intercellular adhesion molecule-1 (sICAM-1), soluble interleukine-2 receptor (sCD25);<br>• Immunotherapy SNP Panel will be also assessed at baseline.

Table 4: Summary of study aim

3.3 Study Design

The study will be conducted according to an open-label, prospective, randomized, phase II design.
Randomization will be stratified according to stage arranged in the 4 following strata:

- IIIb/c – M1a
- M1b
- M1c with normal LDH
- M1c with elevated LDH.

Subjects will be assessed for response by computed tomography (CT) or Magnetic Resonance Imaging (MRI).

All measurable and non-measurable lesions must be documented at screening (within 28 days prior to randomization) and re-assessed at each subsequent tumor evaluation (every 8 weeks for the first year, every 12 weeks while the patient is on study). Tumor assessments with CT or MRI scans of the chest, abdomen, and pelvis will be performed until disease progression after the second combo treatment per RECIST v1.1. Imaging of the neck should be included if clinically indicated. In the event PET/CT scanner is used for tumor assessments, the CT portion of the PET/CT must meet criteria for diagnostic quality. All scans will be collected for a possible independent review.

For patients who discontinue study treatment for reason other than investigator–determined disease progression, tumor assessments should continue to be performed as scheduled.

For patients with palpable/superficial lesions, clinical disease assessments by physical examination should be performed at baseline and throughout study treatment as clinically indicated. Color photographs with ruler/calipers will be taken at baseline and at all subsequent tumor assessment time points.

The National Cancer Institute Common Toxicity Criteria for Adverse Events (NCI CTC-AE) Version 4.03 will be used to evaluate the clinical safety of the treatment in this study. Patients will be assessed for AEs at each clinical visit and as necessary throughout the study.

Biomarkers ancillary study

A correlative biological study will be performed for the evaluation of biomarkers on the biological samples available (paraffin-embedded tissue, frozen tissue, blood, serum, etc.). Approximately 80-90 patients will take part in the ancillary study.

3.4 Study Schedule

The following IMPs will be used in the study according the scheme shown in Figure 1:

- **Arm A**: Combo Target (LGX818 450 mg p.o. od + MEK162 45 mg p.o. bid) until PD; then Combo Immuno (nivolumab 1 mg/kg solution IV combined with ipilimumab 3 mg/kg solution IV every 3 weeks for 4 doses then nivolumab 3 mg/kg solution IV every 2 weeks) until PD.
- **Arm B**: Combo Immuno (nivolumab 1 mg/kg solution IV combined with ipilimumab 3 mg/kg solution IV every 3 weeks for 4 doses then nivolumab 3 mg/kg solution IV every 2 weeks) until PD; then Combo Target (LGX818 450 mg p.o. od + MEK162 45 mg p.o. bid) until PD.
- **Arm C**: Combo Target (LGX818 450 mg p.o. od + MEK162 45 mg p.o. bid) for 8 weeks followed by Combo Immuno (nivolumab 1 mg/kg solution IV combined with ipilimumab 3 mg/kg solution IV every 3 weeks for 4 doses then nivolumab 3 mg/kg solution IV every 2 weeks) until PD; then Combo Target (LGX818 450 mg p.o. od + MEK162 45 mg p.o. bid) until PD.

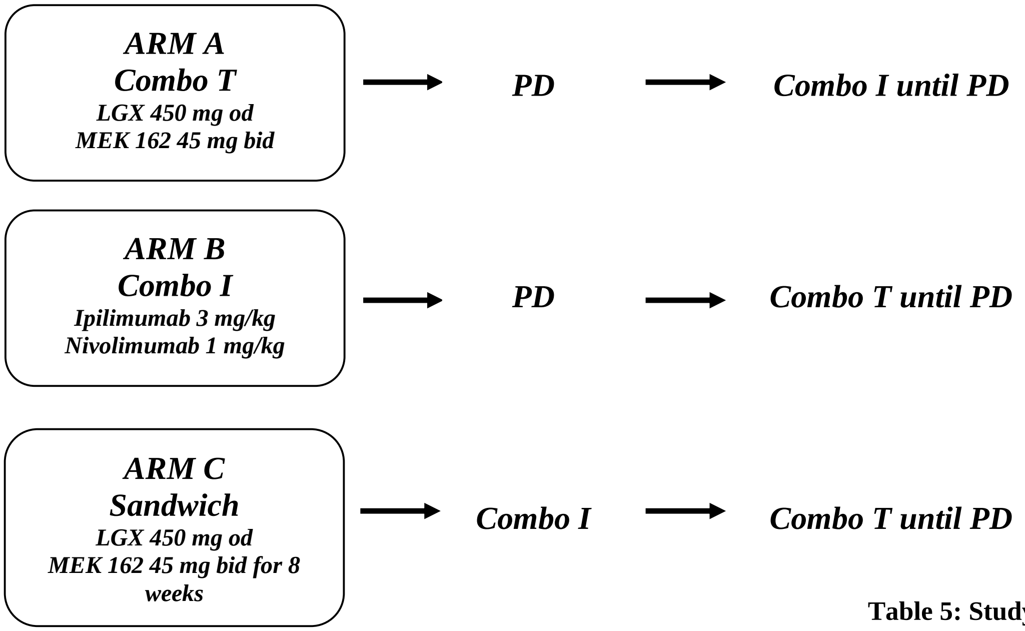

Table 5: Study treatment scheme

Shift from the one combination therapy to the following one can be done (once the procedures of the new screening have been completed) in case of absence of AEs events from the previous combination therapy or in case of grade 1 AEs. In case of grade ≥ 2 AEs from the previous combination therapy, shift to the following combination therapy can be done only when the AE is resolved or has decreased in intensity to at least grade 1. Treatment with any of the treatment schemes will be continued until the development of progressive disease (as per Investigator assessment), unacceptable toxicity, consent withdrawal, death, reasons deemed by the treating physician or study termination by the Sponsor.

3.5 Schedule of Assessments and Procedures

All screening/baseline assessments as outlined in Tables 1 (arm A), 2 (arm B) and 3 (arm C) must be performed within 28 days prior to the first administration of study drugs on Day 1. Results of tests or examinations performed as standard of care before obtaining informed consent and within the 28 days prior to commencing study drugs may be used. All assessments during the study must be performed within a window – 2/+2 days of the day indicated on the schedule of assessment.

Eligibility for the study will be determined by the Investigator from the mandatory screening/baseline assessments performed during screening and according to the study inclusion/exclusion criteria.

First dosing of study drugs will be determined by the patient's eligibility and the laboratory assessments done on Day 1 prior to dosing.

Patients who discontinue study drugs for any reason (e.g. AEs, etc) other than disease progression will continue to be followed until disease progression. Follow-up will continue until the patient has documented disease progression, starts another cancer therapy or withdraws consent.

3.5.1 Screening Examination and Eligibility Screening Form

Written informed consent must be obtained before any study specific assessments or procedures are performed. All screening/baseline evaluations must be performed between Day -28 and -1. Patients who fulfill all the inclusion and none of the exclusion criteria will be accepted into the study.

3.5.2 Procedures for Enrolment of Eligible Patients

A patient who has fulfilled the entry criteria will be given an identifying number. Each identifying number will be unique to the patient for whom it is issued. A patient number will not be re-used if the patient leaves the study. Under no circumstances will patients who enroll in this study and have completed treatment as specified be permitted to re-enroll in the study. A Patient Enrolment and Identification Code List must be maintained by the Investigator.

Eligible patients will be then randomised in one of the three arms in a 1:1:1 ratio, i.e. an equal number of patients will be assigned to one of the three treatment schedules. In each arm and overall, the randomisation will be stratified according to the baseline value of LDH (normal or high – See section 3.3 of the protocol)

3.5.3 Clinical Assessments and Procedures

The following clinical assessments and procedures must be completed for all patients enrolled in this study at screening/baseline and during study visits. All assessments must be performed within a window of 2 days prior to the scheduled visit date and 2 days after the scheduled visit date (- 2 days / + 2 days) for each visit indicated on the schedule of assessment, except for tumor evaluations for which a window of +/- 5 days will apply.

Please refer to Tables 1 (arm A), 2 (Arm B) and 3 (arm C) for specific details and time points collected on clinical assessments and procedures outlined below.

Screening/Baseline:

- Informed Consent Form;
- Demographic data (age, gender, race);
- Medical history (including demographics, relevant medical history, previous and current diseases, prior therapies for melanoma including surgeries and relative responses, prior skin cancer history, therapies and procedures, all medications started within 14 days prior to screening visit);

- Physical exam including height (screening only) and weight and skin examination. Changes from baseline abnormalities should be recorded at each subsequent physical examination. New or worsened abnormalities should be recorded as AEs if appropriate. Tumor lesions accessible by physical examination should be recorded as well for biopsy purpose also. For patients with palpable/superficial lesions, clinical disease assessments by physical examination should be performed at baseline and throughout study treatment as clinically indicated. Color photographs with ruler/calipers will be taken at baseline and at all subsequent tumor assessment time points;
- Vital signs (blood pressure, pulse, temperature, respiratory rate);
- A 12-lead ECG;
- ECOG PS;
- Ophthalmological and dermatological examination;
- Echocardiogram;
- Hematology (including hemoglobin, hematocrit, Platelet Count, WBC, ANC);
- Biochemistry (including glucose, BUN, creatinine or creatinine clearance, sodium, potassium, calcium, magnesium, choloride, amylase, lipase, TSH, free T4, free T3,bicarbonate [if routinely performed on venous blood samples], total bilirubin with fractionation into direct and indirect (if total bilirubin elevated), alkaline phosphatase, AST [SGOT], ALT [SGPT]) and LDH;
- Serum pregnancy test (within 24 hours prior to commencement of dosing) for women of child-bearing potential confirmed by serum HCG laboratory test;
- Presence of BRAF V600E or V600K mutation in tumor tissue prior to enrolment;
- Cardiac/Muscle Enzymes: Troponin, Creatine Kinase (CK). If total CK ≥3 X ULN, then measure isoenzymes and myoglobin in blood or urine weekly;
- Urinalysis;
- Tumor assessments including measurable and non-measurable lesions (baseline brain CT or MRI, CT/MRI C/A/P, bone scan if clinically indicated);
- Assessment of HRQoL (EORTC QLQ-C30), general health status (EQ-5D) and impairment of work productivity and activity (WPAI:GH);
- Concomitant therapy;
- AEs (including SAEs) related to study-mandated procedures from time signed Informed Consent is obtained until first dose of study drugs;
- Biopsy of tumoral lesions for the biological study;
- Peripheral Blood Mononuclear Cells (PBMC) for the biological study.

During Study

- Physical exam at every visit;
- Vital signs (blood pressure, pulse, temperature, respiratory rate) at every visit;
- ECOG PS at every visit;
- A 12-lead ECG will be performed at at screening/baseline, at 2nd baseline at the 1th month and every 12 weeks while the patient is on study;
- The ophthalmologic examination will be performed at the 2nd baseline visit and then when clinically indicated;
- The dermatologic evaluation will be performed every 8 weeks while the patient is on study;
- The echocardiogram will be performed at screening/baseline, at the 2nd baseline (and 3rd baseline ARM C only), at the 1st month and every 12 weeks while the patient is on study;
- Hematology (including hemoglobin, hematocrit, platelet count, WBC, ANC) at every visit
- Biochemistry (including glucose, BUN, creatinine or creatinine clearance, sodium, potassium, calcium, magnesium, choloride, amylase, lipase, TSH, free T4, free T3,bicarbonate [if routinely performed on venous blood samples], total bilirubin with fractionation into direct and indirect (if total bilirubin elevated), alkaline phosphatase, AST [SGOT], ALT [SGPT] and lactate dehydrogenase (LDH) at every visit;

- A serum pregnancy test to be performed every 6 (± 1) weeks during the treatment period;
- Cardiac/Muscle Enzymes: Troponin, Creatine Kinase (CK). If total CK ≥3 X ULN, then measure isoenzymes and myoglobin in blood or urine weekly;
- Urinalysis (only if clinically indicated);
- Biopsy of tumoral lesions for the biological study (if applicable);
- PBMCs for the biological study will be taken (if applicable).
- Tumor assessments of both measurable and non-measurable disease (baseline brain CT or MRI, CT/MRI C/A/P, bone scan if clinically indicated) every 8 weeks for the first year and every 12 weeks thereafter while the patient is on study;
- Assessment of HRQoL (EORTC QLQ-C30), general health status (EQ-5D) and impairment of work productivity and activity (WPAI:GH) will be performed every 28 days during the Combo Target therapy and at administration of nivolimumab+ipilimumab (i.e. every 21 days for 4 doses) and then every two administrations (i.e.every 28 days) of nivolimumab during the Combo Immuno therapy;
- Concomitant therapy throughout the study;
- AEs (including SAEs) throughout the study;
- IMP administration throughout the study.

End of Study Visit (when patient discontinues study drugs)

- Physical exam;
- Vital signs (blood pressure, pulse, temperature, respiratory rate);
- 12-lead ECG (if clinically indicated)
- ECOG PS
- Hematology (including hemoglobin, hematocrit, Platelet Count, WBC, ANC);
- Biochemistry (including glucose, BUN, creatinine or creatinine clearance, sodium, potassium, calcium, magnesium, choloride, amylase, lipase, TSH, free T4, free T3,bicarbonate [if routinely performed on venous blood samples], total bilirubin with fractionation into direct and indirect (if total bilirubin elevated), alkaline phosphatase, AST [SGOT], ALT [SGPT]) and LDH;
- Cardiac/Muscle Enzymes: Troponin, Creatine Kinase (CK). If total CK ≥3 X ULN, then measure isoenzymes and myoglobin in blood or urine weekly;
- Urinalysis;
- Serum pregnancy test;
- Tumor assessments if not performed within the prior 6 weeks (CT/MRI C/A/P, CT/MRI of brain as clinically indicated) including assessment of any tumor lesions accessible by physical examination;
- Assessment of HRQoL (EORTC QLQ-C30), general health status (EQ-5D) and impairment of work productivity and activity (WPAI:GH);
- Concomitant therapy;
- AEs (including SAEs);

Follow up visit within 28 days from discontinuation of study drugs

- Monitoring of AEs and SAEs
- Follow up for disease progression for those patients who have discontinued study drug for any reason (i.e. AE, etc) other than disease progression;
- Assessment of HRQoL (EORTC QLQ-C30), general health status (EQ-5D) and impairment of work productivity and activity (WPAI:GH);
- Serum pregnancy test;
- New anti-cancer therapy administration.

3.5.4 Biomarker study

Tumor biopsies and peripheral blood samples will be taken according to the following scheme (Table 6):

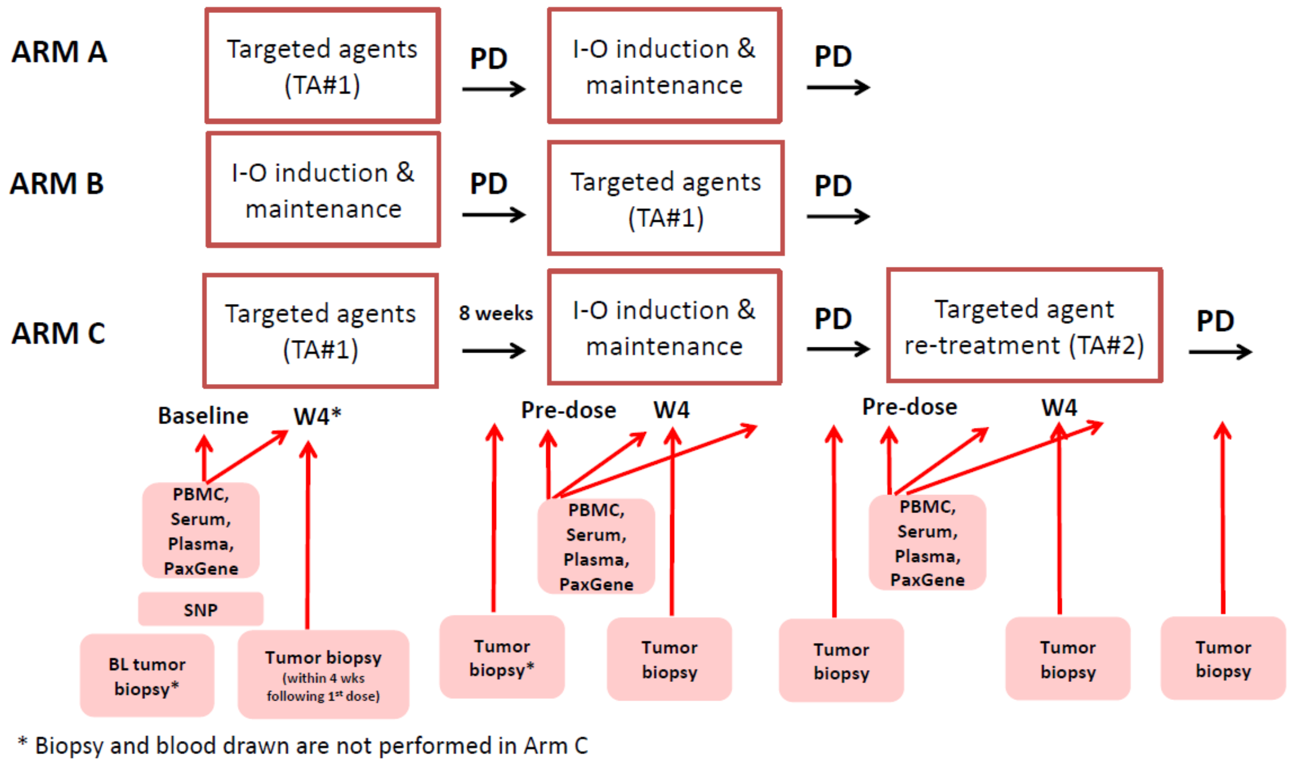

Table 6: Biomarker study treatment scheme

Note: Baseline tumor biopsies can be archival if is collected after prior systemic therapy; otherwise a fresh biopsy will be collected

Tumor biopsies:

The ancillary study requires tumor biopsies at baseline, on-treatment (within first 4 weeks after first dose), and upon progression when feasible.

In arm A, biopsy of tumoral lesions for the biological study will be performed at baseline 1, Week 4 and disease progression 1 (DP 1) during Combo Target therapy; and at Week 4 and disease progression 2 (DP 2) during Combo Immuno therapy. Biopsy of tumoral lesions will be mandatory at baseline 1 and at the first disease progression (PD1), and will be optional at the other time-points.

In arm B, biopsy of tumoral lesions for the biological study will be performed at baseline 1, Week 4 and disease progression 1 (DP 1) during Combo Immuno therapy; and at Week 4 and disease progression 2 (DP 2) during Combo Target therapy. Biopsy of tumoral lesions will be mandatory at baseline 1 and at the first disease progression (PD1), and will be optional at the other time-points.

In arm C, biopsy of tumoral lesions for the biological study will be performed at baseline 1, Week 4 and disease progression 1 (DP 1) during Combo Immuno therapy; and at Week 4 and disease progression 2 (DP 2) during the 2nd Combo Target therapy.

Biopsy of tumoral lesions will be mandatory at baseline 1 and at the first disease progression (PD1), and will be optional at the other time-points. A minimum of 15 x tissue sections will be collected for each patient/time point.

Fresh baseline biopsies will be performed in patients with easily accessible biopsy sites (subcutaneous and lymph nodes) and archived samples will be allowed for patients that cannot be biopsied. Procedures for sample collection and processing will be given to Investigators. Sites will be provided with appropriately containers with formalin. Sample will remain in the 10% Neutral Buffered Formalin for a minimum of 24-48 hours but no more than 96 hours. A matched paraffin embedded (FFPE) tissue block will be then stored ambient at the site until shipment.

Collection of the on-treatment sample should be encouraged, in order to obtain at least 30 (out of possible 138) matched pre- and on-treatment samples for Arm A/C and 30 (out of 69 patients) matched pre- and on-treatment samples for Arm B. Path assessments will be regularly performed to ensure that collected samples are of good quality.

Peripheral blood samples:

In arm A, peripheral blood samples will be taken at baseline 1, Week 4 and disease progression 1 (DP 1) during Combo Target therapy; and at baseline 2, Week 4 and disease progression 2 (DP 2) during Combo Immuno therapy.

In arm B, peripheral blood samples will be taken at baseline 1, Week 4 and disease progression 1 (DP 1) during Combo Immuno therapy; and at baseline 2, Week 4 and disease progression 2 (DP 2) during Combo Target therapy.

In arm C, peripheral blood samples will be taken at baseline 1, baseline 2 and Week 4 during Combo Immuno therapy; and at baseline 3 and Week 4 during the 2nd Combo Target therapy. In arm C, peripheral blood samples will be taken at baseline 1, at baseline 2, Week 4 and disease progression 1 (DP 1) during Combo Immuno therapy; and at baseline 3, Week 8 and disease progression 2 (DP 2) during the 2nd Combo Target therapy.

In all arms, whole blood for SNP will be taken at baseline 1 only.

3.5.5 Efficacy Assessments and biomarkers study

Biopsies

Biopsies at baseline, on-treatment (within first 8 weeks after first dose), and upon progression when feasible, will be obtained for patients with accessible tumors upon patient's consent to participate in the biomarker study. Accessible lesions are defined as tumor lesions which are easily biopsiable i.e. cutaneous, sub-cutaneous and palpable lymph nodes. Failure to obtain sufficient tumor sample, after making best efforts, will not be considered a protocol violation. Lesions with the biggest change in size, based on interval evaluation, are recommended to

be excised at time of progressive disease. Whenever possible, biopsies at progression should be obtained within 3 days of study drug discontinuation. This may require prolonging the treatment a short time after tumor evaluation demonstrates progressive disease.

A minimum of 15 x tissue sections will be collected for each patient/time point. Biopsies will be immediately transferred into the provided vials, filled with formalin. The biopsies will be fixed for 24±2 hours, transferred into 70% ethanol and then (always in ethanol) shipped to a central pathology lab for paraffin embedding. In cases where a reasonable size biopsy (e.g., excisional biopsy, 5 mm punch, or more than a single 14 gauge core biopsy) could be collected with formalin fixation, every effort should also be made to collect a fresh frozen biopsy at disease progression. Optimally both specimens should be available when biopsy is of a reasonable size.

The following tumor tissue biomarkers will be measured:

- Immune status: CD3, CD8, CD4, CD45RO and CD11c T cells; Activated T cells; T regulatory cells; Dendritic cells;
- Resistance to immunotherapy agents: Checkpoint receptors/ligands; myeloid-derived suppressor cells (MDSCs);
- Resistance to targeted agents: aberrations in MEK/PI3K pathways; mRNA expression (nanostring method).

<u>Gene panel:</u>

Using the Ion Torrent technology, the following gene-panel options (which have been already successfully tested on DNA samples from FFPE tissues) can be used for the assessment of the alterations into the MAPK-PI3K pathways

1. Hotspot regions in Ion Ampliseq Cancer Hotspot panel (~2,800 mutations of 50 oncogenes and tumor suppressor genes): 1 pool (10-15 ng per DNA sample)*

ABL1	*EGFR*	*GNAS*	*KRAS*	*PTPN11*
AKT1	*ERBB2*	*GNAQ*	*MET*	*RB1*
ALK	*ERBB4*	*HNF1A*	*MLH1*	*RET*
APC	*EZH2*	*HRAS*	*MPL*	*SMAD4*
ATM	*FBXW7*	*IDH1*	*NOTCH1*	*SMARCB1*
BRAF	*FGFR1*	*JAK2*	*NPM1*	*SMO*
CDH1	*FGFR2*	*JAK3*	*NRAS*	*SRC*
CDKN2A	*FGFR3*	*IDH2*	*PDGFRA*	*STK11*
CSF1R	*FLT3*	*KDR*	*PIK3CA*	*TP53*
CTNNB1	*GNA11*	*KIT*	*PTEN*	*VHL*

2. Exons within melanoma-associated genes from *Comprehensive Cancer Panel*: 3 pools (10-15 ng per pool, 30-45 ng per DNA sample)*

GENE	Position	GENE	Position
AKT3	chr1	HRAS	chr11
NRAS	chr1	ARID2	chr12
IDH1	chr2	CDK4	chr12
BAP1	chr3	KRAS	chr12
MITF	chr3	RB1	chr13
PIK3CA	chr3	AKT1	chr14
KDR	chr4	IDH2	chr15
KIT	chr4	MAP2K1	chr15
BRAF	chr7	ERBB2	chr17
MET	chr7	NF1	chr17
CDKN2A	chr9	TP53	chr17
GNAQ	chr9	AKT2	chr19
NOTCH1	chr9	GNA11	chr19
PPP6C	chr9	MAP2K2	chr19
PTEN	chr10	GNAS	chr20
CCND1	chr11	DDX3X	chrX

3. Exons within >400 oncogenes and tumor suppressor genes (*Comprehensive Cancer Panel*): 4 pools (10-15 ng per pool, 40-60 ng per DNA sample)*

**amount is referred to good quality DNA; thus, quantity of DNA from FFPE samples could be higher*

Study of peripheral blood biomarkers

Peripheral blood mononuclear cell (PBMC) samples will be collected for the biomarkers study.

The following peripheral blood biomarkers will be measured:

- Immune status/“Resistance” to immunotherapy agents: Activated T cells; Memory/Exhausted T cells; T regulatory cells; MDSCs; Inflammatory response; CRP; TCR Sequencing/Gene Expression analysis;
- Response/Resistance to targeted agents: Apoptotic tumor cells (as measured by circulating tumor DNA); soluble hepatocyte growth factor (sHGF); serum vascular endothelial growth factor (sVEGF); soluble intercellular adhesion molecule-1 (sICAM-1); soluble interleukine-2 receptor (sCD25).

Immunotherapy SNP Panel will be also assessed at baseline.

3.5.6 Tumor Response Criteria

Tumor evaluation will be assessed at screening/baseline (between Day -28 and -1) by means of CT or MRI of the chest, abdomen and pelvis (C/A/P), every 8 weeks for the first year and every 12 weeks thereafter while the patient is on study, and at the end of study visit. A window of +/-5 days of scheduled visit is allowed to complete tumor assessments at the required intervals.

Radiological tumor assessments of C/A/P will be done for measuring extent of disease. In addition, all patients must have a baseline brain CT and/or MRI to assess for brain metastasis. Patients with known or suspected bone metastases should undergo radionuclide bone scan or PET scan at baseline and as per institutional standard of care thereafter.

All measurable and non-measurable lesions must be documented at screening (within 28 days prior to randomization) and re-assessed at each subsequent tumor evaluation (every 8 weeks for the first year, every 12 weeks while the patient is on study). Tumor assessments with CT or MRI scans of the chest, abdomen, and pelvis will be performed until disease progression after the second combo treatment. Imaging of the neck should be included if clinically indicated. In the event PET/CT scanner is used for tumor assessments, the CT portion of the PET/CT must meet criteria for diagnostic quality. All scans will be collected for a possible independent review.

In case of a single new lesion treated with surgery or radiotherapy, if all the other sites are under response patients should be treated beyond progression with the first therapy. For switching to the next treatment there should be measurable disease and the progression should be evident even in the other lesions.

As indicated in Section 4.6, patients with PD and evidence of unoperable brain metastases without the involvement of other sites will not undergo the biopsy and will continue treatment according the protocol arm.

For patients who discontinue study treatment for reason other than investigator–determined disease progression, tumor assessments should continue to be performed as scheduled.

Tumor responses will be assessed by the Investigator according to RECIST Criteria (version 1.1). Both measurable and non-measurable lesions will be assessed by the Investigator. In the case of SD, measurements must have SD for at least 6 weeks. For assessing response in patients with measurable disease, the preferred radiologic tumor response assessment is the CT scan with oral and IV contrast. If IV contrast is contraindicated, a non-contrast chest CT will be done with abdominal/pelvic contrast enhanced MRI. If contrast enhanced MRI is contraindicated then non-contrast MRI will suffice. CT/MRI scans of extremities may be done as appropriate in individual patients. PET scan, bone scan, and ultrasound, are not adequate for RECIST response assessment. Patients should be assessed at designated time-points using a consistent imaging modality. The same method of assessment and the same technique should be used to characterize each identified and reported lesion at baseline and during follow-up. If more than one method of assessment is used at baseline, select the most accurate method according to RECIST when recording data; in addition, this method should again be performed in all subsequent evaluations. Tumor measurements should be made by the same Investigator/radiologist for each patient during the study to the extent that this is feasible. Objective responses by RECIST (Version 1.1) should be confirmed by repeat assessments at least 4 weeks after initial documentation of response. Clinical lesions will only be considered measurable when they are superficial and > 10 mm diameter as assessed using calipers (e.g. skin

nodules). For skin lesions, documentation by color photography with ruler is required. CT scan is the preferred modality for skin lesions and should be used wherever possible.

3.5.7 Outcomes Research Assessments

Health-related Quality of Life

HRQoL will be evaluated by means of the 30-item European Organisation for Research and Treatment of Care quality of life questionnaire (EORTC QLQ-C30) (Appendix 4).

The EORTC QLQ-C30 (*Aaronson et al, 1993*) is the mostcommonly used QoL instrument in advanced melanoma clinical studies.

It is a 30--item instrument that has gained wide acceptance in oncology clinical studies. TheEORTC QLQ--C30 comprises six functional scales (physical functioning, cognitive functioning, emotional functioning, social functioning and global quality of life) as well as nine symptomscales (fatigue, pain, nausea/vomiting, dyspnea, insomnia, appetite loss, constipation, diarrhea, and financial difficulties). Except for the overall health status and global quality of life items,responses for all items are 4 point categorical scales ranging from 0 (Not at all) to4 (Very much). The overall health status/quality of life responses are 7-point Likert scales.

General Health Status

General health status will be evaluated by means of the European Quality of Life 5-Dimensions (EQ-5D) (*Brooks, 1996*) (Appendix 5), that comprises 5 dimensions of health (mobility, self-care, usual activities, pain/discomfort, anxiety), each consisting of 3 levels (no, some/moderate and extreme problems), and a 0-100 mm visual analog scale (VAS) where 0 = worst imaginable health state and 100 = best imaginable health state. The utility data generated from the EQ-5D is recommended for and commonly used in cost effectiveness analysis.

Work Productivity and Activity Impairment

Impairment of work productivity and activity will be evaluated by means of the Work Productivity and Activity Impairment: General Health (WPAI:GH) questionnaire (*Reilly et al, 1993*) (Appendix 6).

This questionnaire is a 6-item questionnaire yielding four different types of scores. TheWPAI:GH was created as a patient--reported quantitative assessment of the amount ofabsenteeism (work time missed), presenteeism (impairment at work /reduced onthejobeffectiveness), work productivity (overall work impairment/absenteeism plus presenteeism) anddaily activity impairment attributable to general health. WPAI outcomes are expressed asimpairment percentages, with higher numbers indicating greater impairment and lessproductivity, i.e. worse outcomes. The recall period in all WPAI validation studies is 7 days. Thegeneral literature on recall burden suggests that a longer recall period would not be suitable forthe type of information being elicited in the WPAI.

In theory, a shorter recall period wouldimprove accuracy of WPAI responses, but this has not been tested. Assessment of workproductivity will be conducted at each site (or remotely) with the appropriately translated andvalidated version of the WPAI.

3.5.8 Laboratory Assessments

Hematology and biochemistry will be done as part of regular safety assessments. Specifically:

- Hematology: Hemoglobin, WBC, ANC, platelet count
- Biochemistry: glucose, BUN, creatinine or creatinine-clearance, sodium, potassium, calcium, magnesium, choloride, amylase, lipase, TSH, free T4, free T3,bicarbonate (if routinely performed on venous blood samples), total bilirubin with fractionation into direct and indirect bilirubin (if total bilirubin is elevated), alkaline phosphatase, AST [SGOT], ALT [SGPT]) and LDH
- Serum Pregnancy Test in women of child-bearing potential (within 24 hours prior to first administration of study drug).

4. STUDY POPULATION

4.1 Inclusion Criteria

A subject is eligible for the study if all of the following criteria are met:

1) Patients of either sex aged ≥ 18 years;
2) Histologically confirmed stage III (unresectable) or stage IV melanoma with the BRAF V600 mutation. Patients with mucosal melanoma (but not those with ocular melanoma) are eligible for study participation;
3) Treatment naïve patients. As previous systemic treatmtne for melanoma only interferon is permitted (note that prior adjuvant melanoma therapy is permitted if completed at least 6 weeks prior to randomization, and all related adverse events have either returned to baseline or stabilized).
4) Measurable disease by computed tomography (CT) or Magnetic Resonance Imaging (MRI) per RECIST 1.1 criteria;
5) Presence of BRAF V600E or V600K mutation in tumor tissue prior to enrolment;
6) Eastern Cooperative Oncology Group (ECOG) performance status (PS) 0 or 1 (appendix 7);
7) Tumor tissue from an unresectable or metastatic site of disease must be provided for biomarker analyses. An archive sample is mandatory at the screening visit; however, a fresh sample would be preferable;
8) Female subjects of childbearing potential must have a negative serum pregnancy test result at Baseline and must practice a reliable method of contraception for the total study duration plus 23 weeks (i.e. 30 days plus the time required for nivolumab to undergo five half lives) after the last dose of nivolumab and ipilimumab;
9) Men who are sexually active with women of childbearing potential must practice a reliable method of contraception for the total study duration plus 31 weeks (i.e. 80 days plus the time required for nivolumab to undergo five half lives) after the last dose of nivolumab and ipilimumab;
10) Adequate bone marrow haematological function: absolute neutrophil count (ANC) ≥ 1.5 x 10^9/L AND platelet count ≥ 100 x 10^9/L AND haemoglobin ≥ 9 g/dL;
11) Adequate liver function: total bilirubin ≤ 1.5 x upper limit of normal (ULN) AND aspartate aminotransferase (AST)/alanine aminotransferase (ALT) ≤ 2.5 X ULN (< 5 x ULN if liver metastases);
12) Adequate renal function: serum creatinine ≤ 1.5 mg/dL OR creatinine clearance ≥ 60 mL/min in males and ≥ 50 mL/min in females (calculated according to Cockroft-Gault formula);
13) Serum calcium levels, international normalised ratio (INR) and partial thromboplastin time were within normal limits;
14) Life expectancy of at least 3 months;
15) Ability to understand study-related patient information and provision of written informed consent for participation in the study.

4.2 Exclusion Criteria

A subject is excluded from the study if any of the following criteria are met:

1) Active brain metastases. Subjects with brain metastases are eligible if these have been treated and there is no magnetic resonance imaging (MRI) evidence of progression for at least 8 weeks after treatment is complete and within 28 days prior to first dose of study drug administration. There must also be no requirement for immunosuppressive doses of systemic corticosteroids (> 10 mg/day prednisone equivalents) for at least 2 weeks prior to study drug administration;
2) Subjects with active, known or suspected autoimmune disease;
3) Subjects with a condition requiring systemic treatment with either corticosteroids (>10 mg daily prednisone equivalents) or other immunosuppressive medications within 14 days of treatment;
4) Prior treatment with an anti-Programmed Death receptor-1 (PD-1), anti-Programmed Death-1 ligand-1 (PD-L1), anti-PD-L2, or anti-cytotoxic T lymphocyte associated antigen-4 (anti-CTLA-4) antibody;
5) Female subjects who are pregnant (positive pregnancy test), breast-feeding, or who are of childbearing potential and not practicing a reliable method of birth control;
6) Evidence of severe or uncontrolled systemic disease or any concurrent condition which in the investigator's opinion makes it undesirable for the patient to participate in the study, or which would jeopardize compliance with the protocol, or would interfere with the results of the study;
7) Patients with a history of cardiovascular or interstitial lung disease and evidence or risk of retinal vein occlusion or central serous retinopathy (Past or present evidence of rethinophaty central serous retinopathy - CSR -, occlusion of retinal - RVOo retinal degenerative disease) or ophthalmopathy, which according to the ophthalmologic evaluation at baseline could be considered a risk factor for CSR / RVO (eg. cupping of the optic disc, visual field defect, intraocular pressure - (eg: central IOP - > 21 mmHg).;
8) History of Gilbert's syndrome;
9) Inability to regularly access centre facilities for logistical or other reasons;
10) History of poor co-operation, non-compliance with medical treatment, or unreliability;
11) Participation in any interventional drug or medical device study within 30 days prior to treatment start.
12) Positive test for hepatitis B virus surface antigen (HBV sAg) or hepatitis C virus ribonucleic acid (HCV antibody) indicating acute or chronic infection;
13) Known history of testing positive for human immunodeficiency virus (HIV) or known acquired immunodeficiency syndrome (AIDS);

4.3 Study treatments

Treatments information is given in the following table (Table 7).

Treatment Arm	Number of Pts Planned	Type of Study Drug	Compound	Minimum dose and unit	Frequency	Administration route
**Arm A**	69	Investigational	LGX818	450 mg	Daily	PO
		Investigational	MEK162	90 mg	Daily	PO
		Investigational	Ipilimumab	3 mg/kg	q3 weeks for 4 cycles	IV
		Investigational	Nivolumab	1 mg/kg	q3 weeks for 4 cycles then q2 weeks	IV
**Arm B**	69	Investigational	LGX818	450 mg	Daily	PO
		Investigational	MEK162	90 mg	Daily	PO
		Investigational	Ipilimumab	3 mg/kg	q3 weeks for 4 cycles	IV
		Investigational	Nivolumab	1 mg/kg	q3 weeks for 4 cycles then q2 weeks	IV
**Arm C**	69	Investigational	LGX818	450 mg	Daily	PO
		Investigational	MEK162	90 mg	Daily	PO
		Investigational	Ipilimumab	3 mg/kg	q3 weeks for 4 cycles	IV
		Investigational	Nivolumab	1 mg/kg	q3 weeks for 4 cycles then q2 weeks	IV
**TOTAL**	207					

Table 7: Treatments used in the study

4.4 Concomitant Medication and Treatment

The patient must notify the investigational site about any new medications he/she takes after the start of the study drug. All medications (other than study drug) and significant non-drug therapies (including physical therapy, herbal/natural medications and blood transfusions) administered during the study must be listed on the Concomitant Medications or the Surgical and Medical Procedures eCRF.

Patients taking concomitant medications chronically should maintain the same dose and dose schedule throughout the study if medically feasible. On the days PK blood sampling is performed, the patient should continue their consistent use of other concomitant medication.

However, if a concomitant medication is used intermittently during the study, this medication should be avoided on these days, if medically feasible.

A single new lesion can be treated with surgery or radiotherapy, provided that all the other measurable lesions have not yet progressed.

Prohibited concomitant therapy

Anticancer therapies (including chemo- or biologic-therapy or radiation therapy, covering >30% of the red bone marrow reserve, and surgery) are prohibited while the patients are receiving study treatment. If such therapeutic measures are required for a patient then the patient must be discontinued from study treatment.

4.5 Drug Interaction

LGX818 is a reversible inhibitor of CYP2B6, CYP2C9, CYP3A4 and UGT1A1. It is also a time dependent inhibitor of CYP3A4. MEK162 is also a reversible inhibitor of CYP2B6.

Permitted medications to be used with caution in this study include those that are sensitive substrates of CYP2B6, CYP2C9, CYP3A4, and UGT1A1 or those substrates that have a narrow therapeutic index (NTI).

There is a potential for MEK162 and LGX818 to induce CYP3A4 at concentrations >10-50 uM, which may reduce the effectiveness of hormonal contraception methods. Therefore, the use of at least one form of non-hormonal contraception will be needed during the participation in this study. Caution should be used in patients receiving concomitant treatment with other drugs that are substrates of CYP3A4 as the efficacy of these drugs could be reduced when administered with MEK162 and/or LGX818.

LGX818 has been identified to be primarily metabolized by CYP3A4 *in vitro*. It is advised that LGX818 should be taken with caution when co-administered with strong inhibitors of CYP3A4.

MEK162 has been identified to be primarily metabolized by UGT1A1 *in vitro*. It is advised that inhibitors and inducers of UGT1A1 should be taken with caution.

In vitro data showed that both MEK162 and LGX818 are substrates of P-gp. MEK162 is also a substrate of breast cancer resistant protein (BCRP). Thus, the use of drugs that are known to inhibit or induce P-gp and BCRP should be used with caution. LGX818 is a BCRP inhibitor. It is also a potent inhibitorof the renal transporters OAT1, OAT3 and OCT2 and the hepatic transporters OATP1B1 and OATP1B3. Therefore the co-administration of drugs that are known to be sensitive or NTI substrate of BCRP, OAT1, OAT3, OCT2, OATP1B1 and OATP1B3 should be used with caution.

The solubility of MEK162 and LGX818 is pH dependent and a 10-fold decrease in solubility is observed between pH 1 and 2. Patients receiving concomitant treatments that could potentially modify the gastric pH (i.e. PPI) should be instructed to take them at least two hours after the administration of MEK162.

Drugs with a conditional, possible, or known risk to induce Torsade de Pointes (TdP) should be used with caution. Patients receiving such medications must be carefully monitored for potentiating of toxicity due to any individual concomitant medication, and may require dose titration of the drug substance. Investigators should use caution when prescribing comedications, as clinical experience with these compounds in patients with cancer is often limited. Investigators should contact the Sponsor when they are unsure whether a drug should be prescribed to a patient in the clinical trial.

4.6 Criteria for Premature Withdrawal

Patients have the right to withdraw from the treatment or from the study at any time and irrespective of the reason. Patients who discontinue from the study will be asked to return to the clinic within 28 days of the last dose of IMP for the follow-up visit and to be contacted every 12 weeks until 24 months for long term survival follow-up.

If lost to follow-up, the Investigator should make every effort to contact the patient by telephone or by sending a registered letter to establish as completely as possible the reason for the withdrawal. A complete final evaluation at the time of the patient's withdrawal should be made with an explanation of why the patient is withdrawing from the study.

When applicable, patients should be informed of circumstances under which their participation may be terminated by the Investigator without their consent. The Investigator may withdraw patients from the study in the event of intercurrent illness, AEs, treatment failure after a prescribed procedure, lack of compliance with the study and/or study procedures (e.g., dosing instructions, study visits), or any reason where it is felt by the Investigator that it is in the best interest of the patient to be terminated from the *study*. Any administrative or other reasons for withdrawal must be documented and explained to the patient.

If the reason for removal of a patient from the study is an AE, the principal specific event will be recorded on the eCRF. The patient should be followed until the AE has resolved, if possible. All patients will be followed for safety for 28 days following the last dose of study medication and every 12 week until 24 months after last patient enrolled (long term follow-up visit).

Patients with PD and evidence of unoperable brain metastases without the involvement of other sites will not undergo the biopsy, and will continue treatment according protocol arm.

5. INVESTIGATIONAL MEDICINAL PRODUCTS

The investigational drugs are LGX818 and MEK162 given in combination (Combo Target) and nivolumab 1 mg/kg solution intravenously combined with ipilimumab 3 mg/kg solution intravenously every 3 weeks for 4 doses then nivolumab 3 mg/kg solution intravenously every 2 weeks (Combo Immuno).

5.1 Combo Target

5.1.1 Recommended Dose

Combo Target (LGX818 450 mg p.o. od + MEK162 45 mg p.o. bid) will be given until PD in arm A, and will be given for 8 weeks and then following PD 1 after Combo Immuno until PD 2 in arm C.

LGX818 and MEK162 will be administered orally on a daily schedule as a fixed combination dose, and not by body weight or body surface area (Table 8).

Study treatments	Pharmaceutical form and route of administration	Single Dose	Frequency	Total Daily Dose
LGX818[a]	Capsules for oral use	450 mg	Once daily	450 mg
MEK162[b]	Tablets for oral use	45 mg	Twice daily	90mg

[a]LGX818 will be provided as 100 mg and 50 mg capsules
[b]MEK162 will be provided as 15 mg tablets

Table 8: Combo Target dose and treatment schedule

Patients will be supplied with a sufficient number of tablets and/or capsules for the number of doses to be taken prior to the next scheduled visit. In addition, patients will be provided a dosing diary and should document in this diary each prescribed dose, and whether it was taken or not.

If a patient vomits at any time after dosing, the dose of study drug should not be re-administered. Doses of MEK162 that are omitted for AEs or any other reason should not be made up later in the day, or at the end of the dosing period. Doses of LGX818 that are omitted for AEs or any other reason can be taken up to 12 hours prior to the next dose.

Patients must avoid consumption of grapefruit or grapefruit juice during the entire study and preferably 7 days before the first dose of study medications, due to potential CYP3A4 interaction with the study medications. Orange juice is allowed.

Complete dosing instructions will be provided to study patients and will include the minimum times between doses and instructions for missed doses. Patients will also be instructed not to chew, crush, or dissolve tablets and/or capsules of study drugs. The investigator or responsible site personnel should instruct the patient to take the study drugs as per protocol (promote compliance). All dosages prescribed and dispensed to the patient and

all dose changes and all missed doses during the study must be recorded on the Dosage Administration Record eCRF.

Drug accountability must be performed on a regular basis. Patients will be instructed to return unused study drugs to the site at the next visit. The site personnel will ensure that the appropriate dose of each study drug is administered at each visit and will provide the patient with the correct amount of drugs for subsequent dosing.

5.1.2 Dose Modifications, Interruption and delays criteria for Combo Target

Patients will be monitored for adverse events at each visit with the NCI CTCAE version 4.03 used for all grading.

For patients who do not tolerate LGX818 and/or MEK162 initial dosing schedule, dose adjustment is permitted in order to allow the patient to continue on study drug (see Table 9). A dose reduction below 50 mg bid for LGX818 and below 15 mg od for MEK162 is not allowed. Dose interruptions of more than 28 consecutive days are not allowed.

Dose level	LGX818	MEK162
0 (starting dose)	450 mg od	45 mg bid
-1	300 mg od	30 mg bid
-2	200 mg od	15 mg bid
-3	100 mg od	-
-4	50 mg od	-

Dose reduction should be based on the highest AE grade

Table 9: Dose reduction for LGX818 and MEK162

Doses of MEK162 that are omitted for AEs or any other reason should not be made up later in the day, or at the end of the dosing period. Doses of LGX818 that are omitted for AEs or any other reason can be taken up to 12 hours prior to the next dose. For both LGX818 and MEK162, when the toxicity that resulted in a dose reduction improves to Grade 1 or less, the dose can be re-escalated at the investigators discretion provided there are no other concomitant toxicities.

If MEK162 is dose reduced due to left ventricular dysfunction, no dose re-escalation is allowed.

All dosing interruptions and changes must be recorded on the Dosage Administration Record eCRF.

Dose reduction/interruption/discontinuation decisions should be based on the CTCAE grade of the toxicity and the guidelines provided below (Table 10). All dose modifications should be based on the worst preceding toxicity (CTCAE version 4.03).

In general, doses should not be reduced or interrupted for Grade 1 toxicities, but treatment to control symptoms should be provided as appropriate. All AEs should be followed weekly or as clinically appropriate until stabilization or resolution.

If a patient on the Combo Target therapy discontinues treatment with MEK162, the patient may continue treatment with LGX818. However, due to the limited efficacy of MEK162 alone in the study population, if a patient on the Combo Target therapy discontinues treatment with LGX818, he/she must discontinue treatment with MEK162, complete the end of treatment visit and continue to be followed until disease progression.

Please refer to Table 10 for dose adjustment recommendations for LGX818 and/or MEK162 induced toxicities. Please refer to Appendix 1and Appendix 2 for additional supportive care guidelines for the management of LGX818/MEK162 and MEK162-induced skin toxicity and diarrhoea respectively.

**Recommended Dose Modifications for LGX818/MEK162 combination**	
**Worst Toxicity CTCAE v4.03 Grade (unless otherwise specified)[a]**	**Recommended Dose Modifications any time during a cycle of therapy**
No toxicity	Maintain dose level
**Blood and lymphatic system disorder**	
Febrile neutropenia<br>(ANC < 1.0 x 10^9/L, fever ≥ 38.5°C)[b]	Omit dose until resolved, then ↓ 1 dose level of LGX818 and MEK162
**Investigations (blood)**	
Neutropenia (neutrophil count (ANC) decreased)	
Grade 1 (ANC < LLN - 1.5 x 10^9/L) or<br>Grade 2 (ANC < 1.5 - 1.0 x 10^9/L)	Maintain dose level of LGX818 and MEK162
Grade 3 (ANC < 1.0 - 0.5 x 10^9/L)	Omit dose of LGX818 and MEK162 until resolved to ≤ Grade 2, then:<br>- If resolved in ≤ 7 days, maintain dose level of LGX818 and MEK162<br>- If resolved in > 7 days, then ↓ 1 dose level* of LGX818 and maintain dose level of MEK162
Grade 4 (ANC < 0.5 x 10^9/L)	Omit dose of LGX818 and MEK162 until resolved to ≤ Grade 2, then ↓ 1 dose level* of LGX818 and MEK162
Thrombocytopenia (platelet count decreased)	
Grade 1 (PLT < LLN - 75 x 10^9/L) or<br>Grade 2 (PLT <75 - 50 x 10^9/L)	Maintain dose level of LGX818 and MEK162
Grade 3 (PLT < 50-25 x 10^9/L)	Omit dose of LGX818 and MEK162 until resolved to ≤ Grade 1, then:<br>- If resolved in ≤ 7 days, maintain dose level of LGX818 and MEK162.<br>- If resolved in > 7 days and/or with signs of bleeding, then ↓ 1 dose level* of LGX818 and MEK162
Grade 4 (PLT < 25 x 10^9/L)	Omit dose of LGX818 and MEK162 and discontinue patient from study drug treatment.
**Gastrointestinal disorders**	
Diarrhea	
Grade 1	Maintain dose level of LGX818 and MEK162, but initiate anti-diarrhea treatment (see Appendix 3).
Grade 2	Omit dose of LGX818 and MEK162 until resolved to Grade ≤ 1 and then maintain dose level of LGX818 and MEK162<br>- For 2nd occurrence of diarrhea Grade 2 within 15 days, omit dose of LGX818 and MEK162 until resolved to Grade ≤ 1, then reduce MEK162 by 1 dose level* and maintain dose level of LGX818
Grade 3	Omit dose of LGX818 and MEK162 until resolved to Grade ≤ 1, then reduce dose of LGX818 and MEK162 by 1 dose level*.

Grade 4	Omit dose of LGX818 and MEK162 and discontinue patient from study drug treatment.<br>Note: Anti-diarrhea medication is recommended at the first sign of abdominal cramping, loose stools or overt diarrhea.
Nausea/Vomiting	
Grade 1 or 2	Maintain dose level of LGX818 and MEK162
Grade 3	Omit dose of LGX818 and MEK162 until resolved to Grade ≤ 1, then ↓ 1 dose level* of LGX818 and MEK162
Grade 4	Omit dose of LGX818 and MEK162 and discontinue patient from study drug treatment.<br>Note: Omit dose for ≥ grade 3 vomiting or nausea only if the vomiting or nausea cannot be controlled with optimal antiemetics (as per local practice).
Pancreatitis	
Grade 1 or 2	Maintain dose level of LGX818 and MEK162
Grade ≥ 3	Omit dose of LGX818 and MEK162 and discontinue patient from study drug treatment
**Investigations (Renal)**	
Serum creatinine	
Grade 1 (> ULN – 1.5 x ULN)	Maintain dose level of LGX818 and MEK162
Grade 2 (> 1.5 - 3.0 x ULN)	Omit dose of LGX818 and MEK162 until resolved to ≤ Grade 1, then maintain dose level of LGX818 and MEK162
Grade ≥3 (> 3.0 x ULN)	Omit dose of LGX818 and MEK162 and discontinue patient from study treatment.
**Investigations (Hepatic)**	
Bilirubin	
Grade 1 (> ULN – 1.5 x ULN)	Maintain dose level of LGX818 and MEK162
Grade 2 (> 1.5 – 3.0 x ULN)	Omit dose of LGX818 and MEK162 until resolved to Grade ≤ 1, then:<br>- If resolved in ≤ 7 days, maintain dose level of LGX818 and MEK162<br>- If resolved in > 7 days, ↓ 1 dose level* of LGX818 and MEK162
Grade ≥ 3 (> 3.0 x ULN)	Omit dose of LGX818 and MEK162 and discontinue patient from study drug treatment.<br>Note: If Grade 3 or 4 hyperbilirubinemia is due to the indirect (non-conjugated) component only, and hemolysis as the etiology has been ruled out as per institutional guidelines (e.g., review of peripheral blood smear and haptoglobin determination), then ↓ 1 dose level* and continue treatment at the discretion of the investigator.

AST or ALT	
Grade 1	Maintain dose level of LGX818 and MEK162
Grade 2 or Grade 3	Omit dose of LGX818 and MEK162 until resolved to Grade ≤ 1 (or Grade ≤ 2 in case of liver metastasis), then<br>- If resolved in ≤ 7 days, maintain dose level of LGX818 and MEK162<br>- If resolved in > 7 days, ↓ 1 dose level* of LGX818 and MEK162
Grade 4	Omit dose of LGX818 and MEK162 and discontinue patient from study drug treatment.
AST or ALT and Bilirubin	
AST or ALT > 3.0 - 5.0 x ULN and total blood bilirubin ≥ Grade 2	Omit dose of LGX818 and MEK162 until resolved to Grade ≤ 1, then<br>- If resolved in ≤ 7 days, ↓ 1 dose level* of LGX818 and MEK162<br>- If resolved in > 7 days, discontinue patient from study drug treatment.
AST or ALT > 5.0 x ULN and total blood bilirubin ≥Grade 2	Omit dose of LGX818 and MEK162 and discontinue patient from study drug treatment.
**Investigations (Metabolic)**	
Asymptomatic amylase and/or lipase elevation	
Grade 1 (> ULN - 1.5 x ULN) or<br>Grade 2 (> 1.5 - 2.0 x ULN)	Maintain dose level of LGX818 and MEK162
Grade 3 (> 2.0 - 5.0 x ULN)	Omit dose of LGX818 and MEK162 until resolved to Grade ≤ 2, then :<br>- If resolved in ≤ 7 days, maintain dose level of LGX818 and MEK162<br>- If resolved in<br>> 7 days, ↓ 1 dose level* of LGX818 and MEK162
Grade 4 (> 5.0 x ULN)	Omit dose of LGX818 and MEK162 and discontinue patient from study drug treatment.<br>Note: A CT scan or other imaging study to assess the pancreas, liver, and gallbladder must be performed within 1 week of the first occurrence of any grade ≥ 3 of amylase and/or lipase. If asymptomatic Grade 2 elevations of lipase and/or amylase occur again at the reduced dose, patients will be discontinued permanently from study treatment.
**Cardiac disorders**	
Cardiac general	
Grade 1 or 2	Maintain dose level of LGX818 and MEK162
Grade 3	Omit dose of LGX818 and MEK162, until resolved to Grade ≤ 1, then ↓ 1 dose level* of LGX818 and MEK162
Grade 4	Omit dose of LGX818 and MEK162 and discontinue patient from study drug treatment.

Creatine phosphokinase (CPK)	
Grade 1 (> ULN – 2.5 x ULN) or<br>Grade 2 (> 2.5 - 5.0 x ULN)	Maintain dose level of LGX818 and MEK162
Grade 3 (> 5.0 - 10.0 x ULN)	If asymptomatic: Maintain dose level of LGX818 and MEK162<br>If symptomatic: Omit dose of MEK162 and maintain dose of LGX818 until resolved to Grade ≤ 1, then:<br>- If resolved in ≤ 14 days, then ↓ 1 dose level* of MEK 162 and maintain dose level of LGX8182<br>- If resolved in > 14 days, then discontinue patient from study drug treatment with LGX818 and MEK162
Grade 4 (> 10.0 x ULN)	Omit dose of MEK162 and maintain dose of LGX818 until resolved to CTCAE Grade ≤ 1, then:<br>- If resolved in ≤ 14 days, then ↓ 1 dose level of MEK162 and maintain dose level of LGX818<br>- If resolved in > 14 days, then discontinue patient from study drug treatment with LGX818 and MEK162
If CPK increase measure serum creatinine to assess for renal impairment.<br>Rhabdomyolysis definition according this protocol:<br>• Muscle symptoms (typically muscle pain, weakness in the literature; specific PTs to be defined per cited current MedDRA version for the CSPD)<br>• CK >10X ULN (CTCAE Grade 4) or CK >10,000 IU/L<br>• hospitalization/medical intervention with IV hydration	
**LV systolic dysfunction (not according CTCAE)**	
Asymptomatic decrease of > 10% in LVEF compared to baseline and the ejection fraction is below the institution's lower limit of normal and CTCAE Grade 2	Omit dose of MEK162 until LVEF recovers (defined as ≥ LLN and decrease ≤ 10% compared to baseline).<br>- If the LVEF recovers ≤ 21 days, then ↓ 1 dose level of MEK162, maintain dose of LGX818 and monitor LVEF 2 weeks after restarting on MEK162, every 4 weeks for 12 weeks and subsequently as per protocol<br>- If the LVEF recovers >21 days, then discontinue patient from study drug treatment with MEK162 and LGX818, and closely monitor LVEF until resolution (or for 16 weeks).
Grade ≥ 3	Omit dose of MEK162 and LGX818 and discontinue patient from study drug treatment.
**Vascular disorders**	
Hypertension	
Grade 1 or 2	Maintain dose level of LGX818 and MEK162
Grade 3 (requiring more than one drug or more intensive therapy than previously)	Omit dose of LGX818 and MEK162, until resolved to Grade ≤ 1, then ↓ 1 dose level* of LGX818 and MEK162
Grade 4 (life-threatening)	Omit dose of LGX818 and MEK162, and discontinue patient from study drug treatment
**Eye disorders**	
Eye disorders – RVO[e]	Note: Results of ophthalmic examinations must be made available upon request. This includes scans/images of fluorescein angiography.
any Grade	Omit dose of LGX818 and MEK162 and discontinue patient from study drug treatment[c]

Eye disorders – Retinal events, Uveitis[e]	Note: Results and images of ophthalmic examinations must be made available upon request. This includes scans/images of OCTs.
Grade 1	Maintain dose of LGX818 and MEK162 and increase frequency of ophthalmic monitoring by ophthalmologist to at least every 14 days
Grade 2	Maintain dose of LGX818 and MEK162 and refer the patient to ophthalmologist within one week. Reassess the patient weekly (ophthalmic examination) until resolution to Grade ≤ 1:<br>- If resolved to Grade ≤ 1 in ≤ 21 days, maintain dose of LGX818 and MEK162<br>- If not resolved to Grade ≤ 1 in ≤ 21 days, reduce 1 dose level[d] of LGX818 and MEK162<br>At any time if symptoms worsen, or persist with the same severity for more than 7 days,<br>reduce 1 dose leveld of LGX818 and MEK162
Grade 3	Omit dose of LGX818 and MEK162 and refer the patient to ophthalmologist monitoring within one week[e]:<br>- If resolved to Grade ≤ 1 in ≤ 21 days, reduce 1 dose level[b] of LGX818 and MEK162<br>- If not resolved to Grade ≤ 1 in ≤ 21 days, permanently discontinue LGX818 and MEK162, and refer the patient to ophthalmologist monitoring
Grade 4	Permanently discontinue LGX818 and MEK162, and refer the patient to ophthalmologist monitoring[e]
Eye disorders – any other (i.e. retinal detachment)	
Grade 1 or 2	Maintain dose level of LGX818 and MEK162 and increase frequency of ophthalmic monitoring to at least every 14 days.<br>At any time if symptoms worsen, or persist with the same severity for more than 7 days, reduce 1 dose level[d] of LGX818 and MEK162
Grade 3	Omit dose of LGX818 and MEK162 and refer patient to ophthalmologist monitoring within one week[e]:<br>- If resolved to Grade ≤ 1 in ≤ 21 days, reduce 1 dose level[d] of LGX818 and MEK162<br>- If not resolved to Grade ≤ 1 in ≤ 21 days, permanently discontinue LGX818 and MEK162,<br>and refer the patient to ophthalmologist monitoring[e]
Grade 4	Permanently discontinue LGX818 and MEK162, and refer the patient to ophthalmologist Monitoring[e]
**Skin and subcutaneous tissue disorders**	
Rash/ HFSR/ photosensitivity	
Grade 1	Maintain dose level of LGX818 and MEK162, but consider initiating appropriate skin toxicity therapy (see Appendix 3)
Grade 2	Maintain dose level of LGX818 and MEK162, but initiate/intensify appropriate skin toxicity therapy

Grade 3, despite skin toxicity therapy	Omit dose of LGX818 and MEK162, until resolved to Grade ≤ 1 then:<br>- If resolved in ≤ 7 days, ↓ 1 dose level* of LGX818 and MEK162<br>- If resolved in > 7 days, discontinue patient from study drug treatment with LGX818 and MEK162
Grade 4, despite skin toxicity therapy	Omit dose LGX818 and MEK162, and discontinue patient from study drug treatment with LGX818 and MEK162
**General disorders and administration site conditions**	
Fatigue	
Grade 1 or 2	Maintain dose level of LGX818 and MEK162
Grade 3	Omit dose of LGX818 and MEK162, until resolved to Grade ≤ 1, then:<br>- If resolved in ≤ 7 days, maintain dose level of LGX818 and MEK162<br>- If resolved in > 7 days, ↓ 1 dose level* of LGX818 and MEK162
Edema	
Grade 1 or 2	Maintain dose level of LGX818 and MEK162
Grade 3	Omit dose of LGX818 and MEK162 until resolved to Grade ≤ 1, then :<br>- If resolved in ≤ 14 days, ↓ 1 dose level* of MEK162 and maintain dose of LGX818<br>- If resolved in > 14 days, discontinue patient from study drug treatment with LGX818 and MEK162
**Other adverse events[c]**	
Grade 1 or 2	Maintain dose level of LGX818 and MEK162
Grade 3	Omit dose of LGX818 and MEK162, until resolved to Grade ≤ 1, then ↓ 1 dose level* of LGX818 and MEK162
Grade 4	Omit dose of LGX818 and MEK162 and discontinue patient from study drug treatment
[a] All dose modifications should be based on the worst preceding toxicity.<br>[b] Not CTCAE grading<br>[c] except: 1) lymphopenia unless clinically significant, 2) occurrence of KA and/or cutaneous SCC, 3) alkaline phosphatase, 4) AEs not considered clinically significant like alopecia.<br>[d] Dose reduction below 50 mg QD for LGX818, and below 15 mg BID for MEK162 is not allowed<br>[e] Ophthalmic monitoring mandated for retinal event, posterior uveitis, RVO: further evaluation with specialized retinal imaging (e.g. ocular coherence tomography, angiography)<br>* ↓ 1 dose level refers to: next lower dose level of LGX818, MEK162	

Table 10: Recommended dose modifications associated with treatment-related adverse events

Patients whose treatment is interrupted or permanently discontinued due to an adverse event or clinically significant laboratory value, must be followed up at least once a week (or more frequently if required by institutional practices, or if clinically indicated) for 4 weeks, and subsequently at approximately 4-week intervals, until resolution or stabilization of the event, whichever comes first.

Appropriate clinical experts such as an ophthalmologist, cardiologist or dermatologist should be consulted as deemed necessary.

5.1.3 Formulation, Packaging and Labelling of Combo Target

Study drug packaging will bear a label with the identification required by local law, the protocol number, drug identification and dosage (Table 11).

Medication labels (LGX818, MEK162) will be in the local language and comply with the legal requirements of each country in which the study will be conducted. They will include storage conditions for the drugs, and a unique medication number

Study treatments	Packaging	Labelling (and dosing frequency)
MEK162	Tablets in bottles	Labelled as "MEK162" (BID)<br>Study treatment packaging has a label containing a write-in space for the patient number which will be hand-written onto the label by the responsible site personnel.<br>A unique medication number is printed on this label.
LGX818	Capsules in bottles	Labelled as "LGX818" (OD)<br>Study treatment packaging has a label containing a write-in space for the patient number which will be hand-written onto the label by the responsible site personnel.<br>A unique medication number is printed on each part of this label.

Table 11: Packaging and labelling

LGX818

LGX818 100 and 50 mg will be provided as capsules and packaged per strength into bottle.

Each bottle will be labelled at a minimum with a unique identifier (medication number), the lot number, contents (number of capsules), dosage strength and storage conditions and the name and address of the sponsor.

MEK162

MEK162 15 mg will be provided as film-coated tablets and packaged into high-density polyethylene bottles. Each bottle will be labelled at a minimum with a unique identifier (medication number), contents (number of tablets), dosage strength, storage conditions and the name and address of the sponsor.

5.2 Combo Immuno

5.2.1 Recommended Dose

Combo Immuno (nivolumab 1 mg/kg solution (IV) combined with ipilimumab 3 mg/kg solution (IV) every 3 weeks for 4 doses then nivolumab 3 mg/kg solution (IV) every 2 weeks) will be given until PD in arm B, and will be given following PD after Combo Target in arm A and C.

Nivolumab and ipilimumamb will be administered IV, as reported in Table 12.

Study treatments	Pharmaceutical form and route of administration	Single Dose	Frequency
Nivolumab	Solution for infusion	1 mg/kg	Every 3 weeks for 4 doses in combination with ipilimumab than every two weeks until PD as monotherapy
Ipilimumamb	Solution for infusion	3 mg/kg	Every 3 weeks for 4 doses in combination with nivolumab

Table 12: Combo Immuno dose and treatment schedule

The dosing calculations should be based on the body weight. If the subject's weight on the day of dosing differs by > 10% from the weight used to calculate the dose, the dose must be recalculated. All doses should be rounded to the nearest milligram. There will be no dose modifications allowed.

The first dose is to be administered within 3 days following randomization. Nivolumab is to be administered first. The second infusion will always be the ipilimumab study drug, and will start no sooner than 30 minutes after completion of the nivolumab infusion. Separate infusion bags and filters for nivolumab and ipilimumab must be used for each infusion.

Premedications or medications used to treat infusion-related reactions should be sourced by the investigative sites if available and permitted by local regulations.

Antiemetic premedications should not be routinely administered prior to dosing of drugs. See belowfor premedication recommendations following a nivolumab or ipilimumab related infusion reaction.

Nivolumab

Nivolumab is to be administered as a 60-minute IV infusion, using a volumetric pump with a 0.2/0.22 micron in-line filter at the protocol-specified dose. The drug can be diluted with 0.9% normal saline for delivery but the total drug concentration of the solution cannot be below 0.35 mg/ml. It is not to be administered as an IV push or bolus injection. At the end of the infusion, flush the line with a sufficient quantity of normal saline.

Nivolumab vials must be stored at a temperature of 2° C to 8° C and should be protected from light and freezing. If stored in a glass front refrigerator, vials should be stored in the carton. Recommended safety measures for preparation and handling of nivolumab include laboratory coats and gloves.

For details on prepared drug storage and use time of nivolumab under room temperature/light and refrigeration, please refer to the nivolumab Investigator Brochure section for “Recommended Storage and Use Conditions” and/or pharmacy reference sheets.

Care must be taken to assure sterility of the prepared solution as the product does not contain any anti-microbial preservative or bacteriostatic agent. No incompatibilities between nivolumab and polyolefin bags have been observed.

Ipilimumab

Ipilimumab is to be administered as a 90-minute IV infusion, using a volumetric pump with a 0.2 to 1.2 micron in-line filter at the protocol-specified dose. The drug can be diluted with 0.9% normal saline or 5% Dextrose Injection to concentrations between 1 mg/mL and 4 mg/mL. It is not to be administered as an IV push or bolus injection. Care must be taken to assure sterility of the prepared solutions, since the drug product does not contain any antimicrobial preservatives or bacteriostatic agents.

The nivolumab infusion must be promptly followed by a saline flush to clear the line of nivolumab before starting the ipilimumab infusion.

Ipilimumab injection can be used for IV administration without dilution after transferring to a PVC (polyvinyl chloride), non-PVC/non-DEHP (di-2-ethylhexyl-phthalate) or glass containers and is stable for 24 hours at 2-8 ºC or room temperature/room light (RT/RL). For ipilimumab storage instructions, refer to ipilimumab Investigator Brochure and/or pharmacy reference sheets.

Recommended safety measures for preparation and handling include protective clothing, gloves, and safety cabinets.

5.2.2 Dose Modifications, Interruptions and delays criteria for Combo Immuno

Dose delay criteria

Dose delay criteria apply for all drug-related adverse events (regardless of whether or not the event is attributed to nivolumab, ipilimumab, or both). All study drugs must be delayed until treatment can resume

Nivolumab and ipilimumab administration should be delayed for the following:

- Any Grade ≥ 2 non-skin, drug-related adverse event, with the following exceptions:

- Grade 2 drug-related fatigue or laboratory abnormalities do not require a treatment delay

- Any Grade 3 skin, drug-related adverse event
- Any Grade 3 drug-related laboratory abnormality, with the following exceptions for AST, ALT, or total bilirubin:
  - If a subject has a baseline AST, ALT, or total bilirubin that is within normal limits, delay dosing for drug-related Grade ≥2 toxicity
  - If a subject has baseline AST, ALT, or total bilirubin within the Grade 1 toxicity range, delay dosing for drug-related Grade ≥3 toxicity
- Any adverse event, laboratory abnormality, or intercurrent illness which, in the judgment of the investigator, warrants delaying the dose of study medication.

Because of the potential for clinically meaningful nivolumab or ipilimumab related AEs requiring early recognition and prompt intervention, management algorithms have been developed for suspected pulmonary toxicity, GI, hepatotoxicity, endocrinopathy, skin toxicity, neurological toxicity and nephrotoxicity.

In order to standardize the management across the Combo Immuno therapy, for the overlapping adverse event management algorithms present in both the nivolumab and ipilimumab Investigator's Brochure (GI, hepatic, and endocrine algorithms), the recommendations are to follow the nivolumab Investigator's Brochure adverse event algorithms as opposed to the ipilimumab Investigator's Brochure algorithms. Therefore, the algorithms recommended for utilization of nivolumab and ipilimumab are included in Appendix 3.

Dose modifications criteria

Dose reductions or dose escalations are not permitted for both nivolumab and ipilimumab.

Criteria to resume treatment

Subjects may resume treatment with study drug when the drug-related AE(s) resolve to Grade ≤ 1 or baseline value, with the following exceptions:

- Subjects may resume treatment in the presence of Grade 2 fatigue
- Subjects who have not experienced a Grade 3 drug-related skin AE may resume treatment in the presence of Grade 2 skin toxicity
- Subjects with baseline Grade 1 AST/ALT or total bilirubin who require dose delays for reasons other than a 2-grade shift in AST/ALT or total bilirubin may resume treatment in the presence of Grade 2 AST/ALT OR total bilirubin

- Subjects with combined Grade 2 AST/ALT AND total bilirubin values meeting discontinuation parameters (see below) should have treatment permanently discontinued
- Drug-related pulmonary toxicity, diarrhoea, or colitis, must have resolved to baseline before treatment is resumed
- Drug-related endocrinopathies adequately controlled with only physiologic hormone replacement may resume treatment

If the criteria to resume treatment are met, the subject should restart treatment at the next scheduled time-point per protocol. However, if the treatment is delayed past the next scheduled time-point per protocol, the next scheduled time-point will be delayed until dosing resumes.

If treatment is delayed > 6 weeks, the subject must be permanently discontinued from study therapy, except as specified below.

Discontinuation criteria

Treatment with nivolumab and ipilimumabshould be permanently discontinued for the following:

- Any Grade 2 drug-related uveitis or eye pain or blurred vision that does not respond to topical therapy and does not improve to Grade 1 severity within the re-treatment period OR requires systemic treatment
- Any Grade 3 non-skin, drug-related adverse event lasting > 7 days, with the following exceptions for drug--related laboratory abnormalities, uveitis, pneumonitis, bronchospasm, diarrhoea, colitis, neurologic toxicity, hypersensitivity reactions, and infusion reactions:
  - Grade 3 drug-related uveitis, pneumonitis, bronchospasm, diarrhoea, colitis, neurologic toxicity, hypersensitivity reaction, or infusion reaction of any duration requires discontinuation
  - Grade 3 drug-related laboratory abnormalities do not require treatment discontinuation except:
    a) Grade 3 drug-related thrombocytopenia > 7 days or associated with bleeding requires discontinuation
    b) Any drug-related liver function test (LFT) abnormality that meets the following criteria require discontinuation:

       AST or ALT > 8 x ULN

       Total bilirubin > 5 x ULN

       Concurrent AST or ALT > 3 x ULN and total bilirubin > 2 x ULN

- Any Grade 4 drug-related adverse event or laboratory abnormality, except for the following events which do not require discontinuation:
    - Isolated Grade 4 amylase or lipase abnormalities that are not associated with symptoms or clinical manifestations of pancreatitis and decrease to < Grade 4 within 1 week of onset.
    - Isolated Grade 4 electrolyte imbalances/abnormalities that are not associated with clinical sequelae and are corrected with supplementation/appropriate management within 72 hours of their onset
- Any dosing interruption lasting > 6 weeks with the following exceptions:
    - Dosing interruptions to allow for prolonged steroid tapers to manage drug-related adverse events are allowed. Tumor assessments should continue as per protocol even if dosing is interrupted.
    - Dosing interruptions > 6 weeks that occur for non-drug-related reasons may be allowed. Tumor assessments should continue as per protocol even if dosing is interrupted.
- Any adverse event, laboratory abnormality, or intercurrent illness which, in the judgment of the Investigator, presents a substantial clinical risk to the subject with continued nivolumab or ipilimumab dosing.

Treatment of Nivolumab- or Ipilimumab-Related Infusion Reactions

Since nivolumab and ipilimumab contains only human immunoglobulin protein sequences, it is unlikely to be immunogenic and induce infusion or hypersensitivity reactions. However, if such a reaction were to occur, it might manifest with fever, chills, rigors, headache, rash, pruritis, arthralgias, hypo- or hypertension, bronchospasm, or other symptoms. All Grade 3 or 4 infusion reactions should be reported as a SAE if criteria are met. Infusion reactions should be graded according to NCI CTCAE (version 4.03) guidelines.

Treatment recommendations are provided below and may be modified based on local treatment standards and guidelines as appropriate:

For Grade 1 symptoms: (Mild reaction; infusion interruption not indicated; intervention not indicated)

Remain at bedside and monitor subject until recovery from symptoms. The following prophylactic premedications are recommended for future infusions: diphenhydramine 50 mg (or equivalent) and/or paracetamol 325 to 1000 mg (acetaminophen) at least 30 minutes before additional nivolumab administrations.

For Grade 2 symptoms: (Moderate reaction requires therapy or infusion interruption but responds promptly to symptomatic treatment [eg, antihistamines, non-steroidal anti-inflammatory drugs, narcotics, corticosteroids, bronchodilators, IV fluids]; prophylactic medications indicated for ≤24 hours).

Stop the nivolumab or ipilimumab infusion, begin an IV infusion of normal saline, and treat the subject with diphenhydramine 50 mg IV (or equivalent) and/or paracetamol 325 to 1000 mg (acetaminophen);remain at bedside and monitor subject until resolution of symptoms.

Corticosteroid or bronchodilator therapy may also be administered as appropriate. If the infusion is interrupted, then restart the infusion at 50% of the original infusion rate when symptoms resolve; if no further complications ensue after 30 minutes, the rate may be increased to 100% of the original infusion rate. Monitor subject closely. If symptoms recur then no further nivolumab or ipilimumab will be administered at that visit. Administer diphenhydramine 50 mg IV, and remain at bedside and monitor the subject until resolution of symptoms. The amount of study drug infused must be recorded on the eCRF. The following prophylactic premedications are recommended for future infusions: diphenhydramine 50 mg (or equivalent) and/or paracetamol 325 to 1000 mg (acetaminophen) should be administered at least 30 minutes before additional nivolumab or ipilimumab administrations. If necessary, corticosteroids (recommended dose: up to 25 mg of IV hydrocortisone or equivalent) may be used.

For Grade 3 or Grade 4 symptoms: (Severe reaction, Grade 3: prolonged [i.e., not rapidly responsive to symptomatic medication and/or brief interruption of infusion]; recurrence of symptoms following initial improvement; hospitalization indicated for other clinical sequelae [e.g., renal impairment, pulmonary infiltrates]). Grade 4: (life-threatening; pressure or ventilator support indicated).

Immediately discontinue infusion of nivolumab or ipilimumab. Begin an IV infusion of normal saline, and treat the subject as follows. Recommend bronchodilators, epinephrine 0.2 to 1 mg of a 1:1,000 solution for subcutaneous administration or 0.1 to 0.25 mg of a 1:10,000 solution injected slowly for IV administration, and/or diphenhydramine 50 mg IV with methylprednisolone 100 mg IV (or equivalent), as needed. Subject should be monitored until the investigator is comfortable that the symptoms will not recur. Nivolumab or ipilimumab will be permanently discontinued. Investigators should follow their institutional guidelines for the treatment of anaphylaxis. Remain at bedside and monitor subject until recovery from symptoms. In the case of late-occurring hypersensitivity symptoms (e.g., appearance of a localized or generalized pruritus within 1 week after treatment), symptomatic treatment may be given (eg, oral antihistamine, or corticosteroids).

5.2.3 Formulation, Packaging and Labelling of Combo Immuno

Nivolumab will be made available as cartons each containing 10 vials. Ipilimumab will be made available as cartons each containing 4 vials.

Study drug packaging will bear a label with the identification required by local law, the protocol number, drug identification and dosage.

Nivolumab and ipilimumab will be administered open label. Nivolumab and ipilimumab will be required to be labelled locally as per local SOPs and regulations.

Medication labels (nivolumab, ipilimumab) will be in the local language and comply with the legal requirements of each country in which the study will be conducted.

5.3 Accountability, assessment of compliance and destruction of the drugs

Combo Target

Accountability and patient compliance for Combo Target will be assessed by maintaining adequate "drug dispensing" and return records.

These records must contain the following information:

- Documentation of drug shipments received (date received, quantity and batch number)
- Disposition of unused study drug not dispensed to patient

A Drug Dispensing Log must be kept current and should contain the following information:

- Identification code of the patient to whom the study medication was dispensed
- Date(s), quantity and batch number of the study medication dispensed to the patient
- Date(s), quantity and batch number of the study medication returned by the patient

Patients' compliance will be assessed by maintaining adequate study drug dispensing records. Patients will be asked to return all used and unused drug supply containers (of both LGX818 and MEK162) at any visit as a measure of compliance. The Investigator is responsible for ensuring that dosing is administered in compliance with the protocol.

All supplies, including partially used or empty containers and copies of the dispensing and inventory logs, must be returned to the Monitor at the end of the study, unless alternate destruction has been authorized by the Sponsor, or required by local or institutional regulations.

Study treatments must be received by designated personnel at the study site, handled and stored safely and properly, and kept in a secured location to which only the Investigator and designated site personnel have access.

Upon receipt, the study drugs should be stored according to the instructions specified on the drug labels and Investigator's Brochure for LGX818 and MEK162. Study medication is to be stored in a secure locked area while under the responsibility of the Investigator. Receipt and dispensing of study medication must be recorded by an authorized person at the Investigator's site.

Records of drug formulation, batch number, and number of blisters/bottles dispensed must be recorded in the pharmacy study file.

<u>Combo Immuno</u>

Study treatments must be received by designated personnel at the study site, handled and stored safely and properly, and kept in a secured location to which only the Investigator and designated site personnel have access.

Upon receipt, the study drugs should be stored according to the instructions specified on the drug labels and Investigator’s Brochure for nivolumab and ipilimumab. Study medication is to be stored in a secure locked area while under the responsibility of the Investigator. Receipt and dispensing of study medication must be recorded by an authorized person at the Investigator’s site.

Nivolumab and ipilimumab such as partially used study drug containers, vials and syringes may be destroyed on site.

Any unused study drugs can only be destroyed after being inspected and reconciled by the responsible Study Monitor unless study drug containers must be immediately destroyed as required for safety, or to meet local regulations (e.g., cytotoxics or biologics).

On-site destruction is allowed provided the following minimal standards are met:

- On-site disposal practices must not expose humans to risks from the drug.
- On-site disposal practices and procedures are in agreement with applicable laws and regulations, including any special requirements for controlled or hazardous substances.
- Written procedures for on-site disposal are available and followed. The procedures must be filed with the site’s SOPs and a copy provided to the study sponsor upon request.
- Records are maintained that allow for traceability of each container, including the date disposed of, quantity disposed, and identification of the person disposing the containers. The method of disposal, i.e., incinerator, licensed sanitary landfill, or licensed waste disposal vendor must be documented.
- Accountability and disposal records are complete, updated, and available for the Monitor to review throughout the clinical trial period.

If conditions for destruction cannot be met the responsible Study Monitor will make arrangements for return of study drug.

It is the investigator’s responsibility to arrange for disposal of all empty containers, provided that procedures for proper disposal have been established according to applicable federal, state, local, and institutional guidelines and procedures, and provided that appropriate records of disposal are kept.

6. STATISTICAL CONSIDERATIONS

6.1 Study Endpoints

OS is primary endpoint of the study. OS will be calculated from the date of randomization until the date of death from any cause. Any patient not know to have died at the time of data analysis will be censored at the time of the last recorded date on which the patient was know to be alive.

Secondary endpoints of the study will be:

- Total PFS, calculated from the date of randomization until the date of the second progression (i.e. the progression to second treatment); any progression or death will be considered as an event if patient cannot complete treatment sequence;
- Percentage of patients alive at 2 and 3 years;
- Best overall response rate (BORR), defined as the best response designation, as determined by the investigator, recorded between the date of randomization and the date of objectively documented progression per RECIST version 1.1 criteria;
- Duration of response (DoR), calculated as the time from the date of first documented response (CR or PR) until the date of the first documented progression or death due to underlying cancer. If a patient with a CR or PR has no progression or death due to underlying cancer, the patient is censored at the date of last adequate tumor assessment;
- Biological markers (biomarkers ancillary study).

6.2 Sample Size and Analysis Populations

6.2.1 Sample size

This study is designed as a phase II, randomized trial with no formal comparative test. The sample size is discussed for the primary endpoint Overall Survival (OS).

For each arm a single-stage design as described by A'Hern (*A'Hern, 2001*) will be used.

We have assumed a median PFS of about 10 months for the combo target therapy (LGX818/MEK162) (*Mc Arthur et al, 2013*) and a similar value for the combo immunotherapy (ipilimumab/nivolumab) derived from the aggregate clinical activity rate of 65% (*Wolchock et al, 2013*) which, using an exponential distribution for PFS, could broadly give a median PFS of about 9.5 months. OS seems to be strictly correlated with total PFS.

The null hypothesis is a median OS time of 15 months (i.e. percentage of surviving patient of 33% at 24 months). The alternative hypothesis is a median OS time of 23 months (i.e. percentage of surviving of 48% at 24 months)

Using an exact 5% one-sided significance test at least 69 patients have to be randomized in each treatment arm when the power of the study is 80%.

For each arm the strategy will be further investigated if at least 30 patients, alive at 24 months, are observed.

Taking in account a 10% drop-out rate, a total of 230 patients will be enrolled to ensure a minimum of 207 randomized patients.

6.2.2 Analysis Populations

Baseline is defined as the last valid visit or day before the treatment start

The following populations are defined for this study.

All Enrolled Set

All screened subjects who have been enrolled.

Intention To Treat (ITT) set

All randomized patients will be considered for the Intention-To-Treat population (ITT)

Safety Set

All patients of the ITT population receiving at least one dose of the study medication.

6.2.3 Statistical analysis

Data handling

The Overall Survival (OS) will be calculated as the date of randomization until the date of death from any cause.Any patient not know to have died at the time of data analysis will be censored at the time of the last recorded date on which the patient was know to be alive.

Total Progression Free Survival (TPFS) will be calculated from the date of randomization until the date of the second progression (i.e. the progression to second treatment); any progression or death will be considered as an event if patient cannot complete treatment sequence.

Duration of response (DoR) will be calculated as the time from the date of first documented response (Cr5 o PR) until the date of the first documented progression or death due to underlying cancer. If a patient with CR o PR has no progression or death due to underlying cancer, the patient will be censored at the date of the last adeguate tumor assessment.

Statistical analysis

A comprehensive Statistical Analysis Plan (SAP) will be prepared before database lock.

Deviations from methods described in this paragraph might appear.

In case of such deviations the reason for the deviation will be stated in the statistical analysis plan.

Analysis of efficacy endpoints will be performed in the ITT population whereas the safety analysis will be performed in the Safety Population.

No comparative tests between the three arms will be perfomed and results will be presented aas descriptive statistic. Site related differences will be evaluated.

Continuous variables will be summarized by descriptive statistics (number of cases, mean, standard deviation, median, minimum, maximum, first and third quartile). Categorical variables will be summarized using counts of patients and percentages 95% confidence interval will be employed, unless otherwise specified.

The time-dependent endpoints will be analyzed according to the Kaplan-Maier method. Medians with 95% confidence intervals will be derived from the K-M curves and presenting time-dependent endpoints as K-M plot (with a 95% CI over time).

Cox's proportional hazard model will be used to assess the impact of know prognostic factors and treatment assigned.

The list of the covariates to be included in the Cox's model will be presented and clinically justified in the statistical analysis plan.

The ORR will be calculated as the percentage of ITT population patients who have a CRo o PR before any evidence of progression (as defined by RECIST).

A 95% confidence interval (CI) will be derived for the ORR using Wilson score intervals (CIs for a single proportion).

Percentage of patients alive at 2 and 3 years will be reported using Wilson score intervals.

Adverse event are those with start date beyond or equal to the informed consent date,

Analysis includes adverse events with starting date until 30 days after the last study drug dose intake.

All adverse event will be assigned to a Preferred Term (PT) and will be classified by primary System Organ Class (SOC) according to MedDRA thesaurus version 18 or higher.

Adverse events will be reported on a per-patient basis within Preferred Term. This mean that even if a patient will be report the same event repeatedly (i.e. events mapped to the preferred term) the event will be counted only once.

In the latter case the event will be assigned the worst CTCAE severity and the stroingest relationship to the study drug. The earliest date will regard as start date of the event and the latest date will regard as stop date of the event. Adverse events will be assessed accorind to CTCAE version 4.03.

Appropriate summaries of these data will be presented.

Safety and tolerability will be assessed in terms of AEs, laboratory data, ECG data, vitals signs and weight, which will be collected for all patients. AEs (both in terms of MedDRA preferred terms and CTCAE grade), laboratory data, ECG data, vital signs and weight will be listes individually by patient and summarized by treatment received. ECG changes will be summarized for each treatment group.

Vital signs data will be listes for each patient and changes in vital signs will be summarized for each treatment group.

Previous and concomitant medications will be coded using the ATC dictionary, latest version.

Changes from baseline in EQ-5D and QLQ-C£0 total score will be summarized by means of descriptive statistical methods.

6.3 Study Duration

This is a multicenter study that will be conducted in 24 sites located in Europe. It is expected that 230 eligible patients will be enrolled in total for this phase II study by the participating centers to ensure a minimum of 207 evaluable patients.

Treatment duration will be until PD (2 years estimated). It is expected that the study will start (First Patient First Visit Date) on Febrary 2016, the recruitment will end (Last Patient First Visit) on September 2017, and the Study will end (Last Patient Last Visit) on. September 2020. The date of study end is dependent on the clinical course of the disease and may therefore occur earlier than indicated.

7. SAFETY INSTRUCTIONS AND GUIDANCE

7.1 Warning and precautions

No evidence available at the time of the approval of this study protocol indicated that special warnings or precautions were appropriate, other than those noted in the provided Investigator's Brochure for all the IMPs. Additional safety information collected between Investigator's Brochure updates will be communicated in the form of Investigator Notifications. This information will be included in the patient informed consent and should be discussed with the patient during the study as needed.

The recommendations to be followed for the management of toxicities and adverse events are detailed in Section 5.1.2 (Combo Target) and in Section 5.2.2 (Combo Immuno).

Adverse events of special interest for LGX818 and MEK162 are detailed in Section 7.5.2.

7.2 Adverse Events and Laboratory Abnormalities

7.2.1 Clinical Adverse Events (AEs)

According to the International Conference of Harmonization (ICH), an AE is any untoward medical occurrence in a patient or clinical investigation subject (patient) administered a pharmaceutical product and which does not necessarily have a causal relationship with this treatment. An AE can therefore be any unfavourable and unintended sign [including an abnormal laboratory finding], symptom, or disease temporally associated with the use of a medicinal (investigational) product, whether or not considered related to the medicinal (investigational) product. Pre-existing conditions which worsen during a study are to be reported as AEs.

7.2.2 Intensity

Intensity of all AEs will be graded according to the NCI Common Terminology Criteria for Adverse Events v4.03 (CTCAE v 4.03 most recent sub-version) on a five-point scale (Grade 1 to 5) and reported in detail on the CRF.

AEs not listed on the CTCAE should be graded as follows:

CTC Grade Equivalent To:

Definition

- Grade 1: Mild Discomfort noticed but no disruption of normal daily activity

- Grade 2: Moderate Discomfort sufficient to reduce or affect daily activity; no treatment or medical intervention is indicated although this could improve the overall well-being or symptoms of the patient
- Grade 3: Severe Inability to work or perform normal daily activity; treatment or medical intervention is indicated in order to improve the overall wellbeing or symptoms; delaying the onset of treatment is not putting the survival of the patient at direct risk
- Grade 4: Life threatening/disabling. An immediate threat to life or leading to a permanent mental or physical conditions that prevents work or performing normal daily activities; treatment or medical intervention is required in order to maintain survival
- Grade 5: AE resulting in death

7.2.3 Drug AE relationship

The causality relationship of study drug to the AE will be assessed by the Investigator as either: *Yes* or *No*

If there is a reasonable suspected causal relationship to the study medication, i.e. there are facts (evidence) or arguments to suggest a causal relationship, drug-event relationship should be assessed as Yes.

The following criteria should be considered in order to assess the relationship as Yes:

- Reasonable temporal association with drug administration
- It may or may not have been produced by the patient’s clinical state, environmental or toxic factors, or other modes of therapy administered to the patient
- Known response pattern to suspected drug
- Disappears or decreases on cessation or reduction in dose
- Reappears on re-challenge

The following criteria should be considered in order to assess the relationship as No:

- It does not follow a reasonable temporal sequence from administration of the drug
- It may readily have been produced by the patient’s clinical state, environmental or toxic factors, or other modes of therapy administered to the patient
- It does not follow a known pattern of response to the suspected drug
- It does not reappear or worsen when the drug is re-administered

7.2.4 Serious Adverse Events

A serious adverse event (SAE) is any experience that suggests a significant hazard, contraindication, side effect or precaution. It is any AE that at any dose fulfils at least one of the following criteria:

- is fatal (results in death; NOTE: death is an outcome, not an event)

- is Life-Threatening (NOTE: the term "Life-Threatening" refers to an event in which the patient was at immediate risk of death at the time of the event; it does not refer to an event which could hypothetically have caused a death if it had been more severe).
- requires in-patient hospitalization or prolongation of existing hospitalization;
- results in persistent or significant disability/incapacity;
- is a congenital anomaly/birth defect;
- is a cancer;
- is associated with an overdose;
- is another Important Medical Event (any important adverse events/reactions that is not immediately life-threatening or do not result in death or hospitalization, but may jeopardize the subject or may require medically significant or requires intervention to prevent one or other of the outcomes listed above).Examples of such events include, but are not limited to, intensive treatment in an emergency room or at home for allergic bronchospasm, blood dyscrasias or convulsions that do not result in hospitalization). Potential drug induced liver injury (DILI) is also considered an important medical event.

Note: The term sudden death should be used only when the cause is of a cardiac origin as per standard definition. The terms death and sudden death are clearly distinct and must not be used interchangeably. The study will comply with all local regulatory requirements and adhere to the full requirements of the ICH Guideline for Clinical Safety Data Management, Definitions and Standards for Expedited Reporting.

Overdose

An overdose is a significant variation above the recommended/scheduled dosage for a product. In this current trial an overdose of the IMPs is any dose higher than the dose specified in the in Sections 5.1.1 and 5.2.1 of this protocol.

Planned Hospitalization

A hospitalization planned by the subject prior to signing the informed consent form (ICF) is considered a therapeutic intervention and not the result of a new SAE and should be recorded as medical history. If the planned hospitalization or procedure is executed as planned, the record in the subject's medical history is considered complete. However, if the event/condition worsens during the trial, it must be reported as an AE.

7.2.5 Progression of Underlying Malignancy

Progression of underlying malignancy is not reported as an AE if it is clearly consistent with the suspected progression of the underlying cancer as defined by RECIST criteria, or other criteria as determined by protocol. Hospitalization due solely to the progression of underlying malignancy should NOT be reported as a SAE. Clinical symptoms of progression may be reported as AEs if the symptom cannot be determined as exclusively due to the progression of the underlying malignancy, or does not fit the expected pattern of progression for the disease under study.

Symptomatic deterioration may occur in some patients. In this case, progression is evident in the patient's clinical symptoms, but is not supported by the tumor measurements. Or, the disease progression is so evident that the Investigator may elect not to perform further disease assessments. In such cases, the determination of clinical progression is based on symptomatic deterioration. These determinations should be a rare exception as every effort should be made to document the objective progression of underlying malignancy. If there is any uncertainty about an AE being due only to the disease under study, it should be reported as an AE or SAE.

7.3 Treatment and Follow-up of AEs (100 days post discontinuation of study drugs)

After 100 days from the last dose of study drugs continue to follow-up AEs as follows:

- For Related AEs, follow until one of the following occurs:
  - Resolved or improved to baseline
  - Relationship is reassessed as unrelated
  - Death
  - Start of new anti-cancer regimen
  - Investigator confirms that no further improvement can be expected
  - Clinical or safety data will no longer be collected, or final database closure
- For Unrelated severe or life-threatening AEs, follow until one of the following occurs:
  - Resolved or improved to baseline
  - Severity improved to Grade 2
  - Death
  - Start of new anti-cancer regimen
  - Investigator confirms that no further improvement can be expected

- Clinical or safety data will no longer be collected, or final database closure

Unrelated Grade 1 or Grade 2 AEs: follow-up until 100 days after last dose of study drugs and every 12 weeks until 24 months for long term follow-up. The final outcome of each AE must be recorded on the CRF.

7.4 Laboratory Test abnormalities

Laboratory test results will be recorded on the laboratory results form of the eCRF, or appear on electronically produced laboratory reports submitted directly from the central laboratory, if applicable.

Any laboratory result abnormality fulfilling the criteria for an adverse event of special interest (AESI) or a SAE should be reported as such, in addition to being recorded as an AE in the CRF.

Any treatment-emergent abnormal laboratory result which is clinically significant, i.e., meeting one or more of the following conditions, should be recorded as a single diagnosis on the AE page in the eCRF:

- Accompanied by clinical symptoms
- Leading to a change in study medication (e.g. dose modification, interruption or permanent discontinuation)
- Requiring a change in concomitant therapy (e.g. addition of, interruption of, discontinuation of, or any other change in a concomitant medication, therapy or treatment)

This applies to any protocol and non-protocol specified safety and efficacy laboratory result from tests performed after the first dose of study medication, which falls outside the laboratory reference range and meets the clinical significance criteria.

7.4.1 Follow-up of Abnormal Laboratory Test Values

In the event of medically significant unexplained abnormal laboratory test values, the tests should be repeated and followed up until they have returned to the normal range and/or an adequate explanation of the abnormality is found. If a clear explanation is established it should be recorded on the eCRF.

7.5 Handling of Safety Parameters

7.5.1 Reporting of AEs

Information about all adverse events, whether volunteered by the patient, discovered by Investigator questioning, or detected through physical examination, laboratory test or other means, will be collected on the Adverse Event CRF page, documented in the patient's medical records, and followed as appropriate.

The NCI CTC-AE (Version 4.03) will be used to evaluate the clinical safety of the treatment in this study. Patients will be assessed for AEs at each clinical visit and as necessary throughout the study.

The following Adverse Events of special interest assessed as non-serious must be reported to Sponsor within one working day of the Investigator becoming aware of the event (expedited reporting):

- Palmar-plantar erythrodysaesthesia syndrome
- Rash and related events
- Squamous cell carcinoma (SCC), keratoacanthoma (KA) and any other suspicious skin lesions.
- Ocular/visual events
- Retinal vein occlusion
- Rash and related events
- Peripheral/generalized edema/anasarca
- Serum creatinkinase (CK) elevation
- Cardiac failure related events
- Hepatic events.

LGX818

As a result of signals observed from previous LGX818 studies, several AEs requiring a close follow-up were identified. For each category, selected AEs similar in nature, will be identified and grouped:

- Palmar-plantar erythrodysaesthesia syndrome
- Rash and related events
- Squamous cell carcinoma (SCC), keratoacanthoma (KA) and any other suspicious skin lesions.

MEK162

As a result of signals observed from previous MEK162 studies, several AEs requiring a close follow-up were identified. For each category, selected AEs similar in nature, will be identified and grouped:

- Ocular/visual events
- Retinal vein occlusion
- Rash and related events
- Peripheral/generalized edema/anasarca
- Serum creatin-kinase (CK) elevation
- Cardiac failure related events
- Hepatic events.

7.5.2 Reporting of SAEs (immediately reportable)

Any clinical AE, or abnormal laboratory test value assessed as serious(as defined above), AESI or pregnancy case, occurred during the course of this study from the enrolment visit (start of study screening procedures), including long term follow-up, must be reported to Sponsor and PV Manufacturer within *one* working day of the Investigator becoming aware of the event (expedited reporting). The following proviso applies:

During the screening period, after written informed consent has been signed only SAEs related to protocol procedures will be reported. The Investigator must complete the SAE formand forward it to the SAE Responsible person:

**Emergency contact**	**Dr. Paolo A. Ascierto**<br>Istituto Nazionale dei Tumori, Fondazione "G. Pascale"<br>U.O.C. Melanoma, Immunoterapia Oncologica e Terapie Innovative<br>Via M. Semmola 80131 - Naples	Tel: +39 081 5903 236<br>Fax: +39 081 5903 841<br>Email paolo.ascierto@gmail.com
	**Clinical Research Technology srl**<br>Project Management	Tel: +39 089.301545<br>Fax: +39 089.7724155<br>e-mail: pvg@cr-technology.com

In addition, report of Adverse Events will be done, according to current Italian law, to local Health Authorities.

From the first administration of IMPs, all SAEs must be reported. Related SAEs MUST be collected and reported regardless of the time elapsed from the last study drugs administration, even if the study has been closed. Suspected Unexpected Serious Adverse Reactions (SUSARs) are reported to Investigators at each site and associated IRB/IEC when the following conditions occur:

- The event must be a SAE
- There must be a certain probability that the event is an adverse reaction from the administered drugs
- The adverse reaction must be unexpected, that is to say, not foreseen in the Investigator's Brochure of IMPs. When all patients at a particular site are off treatment as defined by the protocol:
  - only individual SUSAR reports originating in that particular trial will be forwarded to the site and associated IRB/IEC on an expedited basis
  - individual SUSARs considered to be a significant safety issue and/or which result in recommending a change to the ICF, will be reported in an expedited manner to all Investigators and IRBs/IECs

- SUSAR reports originating from other trials using the same IMP will be provided as six monthly SUSAR Reports to Investigators and IRBs/IECs where long-term follow-up studies are carried out, unless they are considered significant

Unrelated SAEs must be collected and reported during the study and for up to 100 days after the last dose of study medication and every 12 weeks until 24 months for long-term follow up.

This study adheres to the definition and reporting requirements of ICH Guideline for Clinical Safety Data Management, Definitions and Standards for Expedited Reporting.

7.5.3 Pregnancy

Female patients must be instructed to stop taking IMP and immediately inform the Investigator if become pregnant during the study. The Investigator should report all pregnancies within 24 hours to the Sponsor, using the *Clinical Trial Pregnancy Reporting Form*. The Investigator should counsel the patient, discuss the risks of continuing with the pregnancy and the possible effects on the fetus.

Pregnancies occurring up to 6 months after the completion of the study medication must also be reported to the Investigator. Pregnancies occurring in the partner of a male patient participating in the study should be reported to the Investigator and the Sponsor. The partner should be counselled, the risks of continuing the pregnancy discussed, as well as the possible effects on the foetus. Monitoring of the patient should continue until conclusion of the pregnancy.

8. DATA COLLECTION AND MANAGEMENT

8.1 Data confidentiality

Information about study subjects will be kept confidential and managed under the applicable laws and regulations. The data collection system for this study uses built-in security features to encrypt all data for transmission in both directions, preventing unauthorized access to confidential participant information. Access to the system will be controlled by a sequence of individually assigned user identification codes and passwords, made available only to authorized personnel who have completed prerequisite training.

8.2 Site monitoring

Before study initiation, at a site initiation visit or at an investigator's meeting, Sponsor personnel (or designated CRO) will review the protocol and CRFs with the investigators and their staff. During the study, the field monitor will visit the site regularly to check the completeness of patient records, the accuracy of entries on the CRFs, the adherence to the protocol to Good Clinical Practice, the progress of enrolment, and to ensure that study treatment is being stored, dispensed, and accounted according to specifications. Key study personnel must be available to assist the field monitor during these visits. The investigator must maintain source documents for each patient in the study, consisting of case and visit notes (hospital or clinic medical records) containing demographic and medical information, laboratory data, electrocardiograms and the results of any other tests or assessments. All information recorded on CRFs must be traceable to source documents in the patient's file. The investigator must also keep the original signed informed consent form (a signed copy is given to the patient). The investigator must give the monitor access to all relevant source documents to confirm their consistency with the CRF entries. The Sponsor monitoring standards require full verification for the presence of informed consent, adherence to the inclusion/exclusion criteria and documentation of SAEs.

8.3 Data collection

This study will use an Electronic Data Capture (EDC) system (eClinical platform provided by Clinical Research Technology). The designated investigator staff will enter the data required by the protocol into the Electronic Case Report Forms (eCRF). The eCRFs have been built using eClinical platform provided by Clinical Research Technology, a fully validated secure web-enabled software that conforms to FDA requirements. Investigator site staff will not be given access to the EDC system until they have been trained. Automatic validation programs check for data discrepancies in the eCRFs allow modification or verification of the entered data by the investigator staff. The Principal Investigator is responsible for assuring that the data entered into eCRF is complete, accurate, and that entry and updates are performed in a timely manner.

8.4 Database management and quality control

The Sponsor personnel (or designated CRO) will review the data entered by investigational staff for completeness and accuracy. Electronic data queries stating the nature of the problem and requesting clarification will be created for discrepancies and missing values and sent to the investigational site via the EDC system. Designated investigator site staff is required to respond promptly to queries and to make any necessary changes to the data. Concomitant treatments entered into the database will be coded using the WHO Drug Reference List, which employs the Anatomical Therapeutic Chemical classification system. Medical history/current medical conditions and adverse events will be coded using the Medical dictionary for regulatory activities (MedDRA) terminology. The occurrence of any protocol violations will be determined. After the data has been verified to be complete and accurate, the database will be declared locked. Authorization is required prior to making any database changes to locked data, by joint written agreement between the Biostatistics and Data Management and the Sponsor.

9. ETHICAL CONSIDERATION

9.1 Regulatory and ethical compliance

This clinical study was designed, shall be implemented and reported in accordance with the ICH Harmonized Tripartite Guidelines for Good Clinical Practice, with applicable local regulations (including European Directive 2001/20/EC), and with the ethical principles laid down in the Declaration of Helsinki.

9.2 Responsibilities of the investigator and IEC

The protocol and the proposed informed consent form must be reviewed and approved by Independent Ethics Committee (IEC) of all participating centers before study start.

9.3 Informed consent procedures

Eligible patients may only be included in the study after providing written (witnessed, where required by law or regulation), IEC-approved informed. Informed consent must be obtained before conducting any study-specific procedures (i.e. all of the procedures described in the protocol). The process of obtaining informed consent should be documented in the patient source documents. The date when a subject's Informed Consent was actually obtained will be captured in their CRFs.

9.4 Publication of study protocol and results

The key design elements of this protocol will be posted in the publicly accessible database clinicaltrials.gov. The Investigators assure that results of this study will be submitted for publication and reported in scientific meetings.

9.5 Study documentation, record keeping and retention of documents

Each participating site will maintain appropriate medical and research records for this trial, in compliance with Section 4.9 of the ICH E6 GCP, and regulatory and institutional requirements for the protection of confidentiality of subjects. As part of participating in a Fondazione Melanoma-sponsored study, each site will permit authorized representatives of the sponsor(s) and regulatory agencies to examine (and when required by applicable law, to copy) clinical records for the purposes of quality assurance reviews, audits and evaluation of the study safety and progress. Source data are all information, original records of clinical findings, observations, or other activities in a clinical trial necessary for the reconstruction and evaluation of the trial. Examples of these original documents and data records include, but are not limited to, hospital records, clinical and office charts, laboratory notes, memoranda, subjects' diaries or evaluation checklists, pharmacy dispensing records, recorded data from automated instruments, copies or transcriptions certified after verification as being accurate and complete, microfiches, photographic negatives, microfilm or magnetic media, x-rays, and subject files and records kept at the pharmacy, at the laboratories, and medico-technical departments involved in the clinical trial. Data collection

is the responsibility of the clinical trial staff at the site under the supervision of the site Principal Investigator. The study case report form (CRF) is the primary data collection instrument for the study. The investigator should ensure the accuracy, completeness, legibility, and timeliness of the data reported in the CRFs and all other required reports. Data reported on the CRF, that are derived from source documents, should be consistent with the source documents or the discrepancies should be explained. All data requested on the CRF must be recorded. Any missing data must be explained. For electronic CRFs an audit trail will be maintained by the system. The investigator/institution should maintain the trial documents as specified in Essential Documents for the Conduct of a Clinical Trial (ICH E6 Section 8) and as required by applicable regulations and/or guidelines. The investigator/institution should take measures to prevent accidental or premature destruction of these documents. Essential documents (written and electronic) should be retained for a period of not less than seven (7) years from the completion of the Clinical Trial unless Sponsor provides written permission to dispose of them or, requires their retention for an additional period of time because of applicable laws, regulations and/or guidelines

9.6 Confidentiality of study documents and patient records

The investigator must ensure anonymity of the patients; patients must not be identified by names in any documents submitted to the Sponsor. Signed informed consent forms and patient enrolment log must be kept strictly confidential to enable patient identification at the site.

9.7 Audits and inspections

Source data/documents must be available to inspections by the Sponsor or designee or Health Authorities.

9.8 Financial disclosures

Financial disclosures should be provided by study personnel who is directly involved in the treatment or evaluation of patients at the site - prior to study start.

10. PROTOCOL ADHERENCE

Investigators ascertain they will apply due diligence to avoid protocol deviations. Under no circumstances should the investigator contact the Sponsor, if any, monitoring the study to request approval of a protocol deviation, as no authorized deviations are permitted. If the investigator feels a protocol deviation would improve the conduct of the study this must be considered a protocol amendment, and unless such an amendment is agreed upon by the Sponsor and approved by the IEC it cannot be implemented.

10.1 Amendments to the protocol

Any change or addition to the protocol can only be made in a written protocol amendment that must be approved by the Sponsor, Health Authorities where required, and the IEC. Only amendments that are required for patient

safety may be implemented prior to IEC approval. Notwithstanding the need for approval of formal protocol amendments, the investigator is expected to take any immediate action required for the safety of any patient included in this study, even if this action represents a deviation from the protocol. In such cases, the Sponsor should be notified of this action and the IEC at the study site should be informed within 10 working days.

11. REFERENCES

Aaronson NK, Ahmedzai S, Bergman B et al. The European Organization for Research and Treatment of Cancer QLQ-C30: a quality-of-life instrument for use in international clinical trials in oncology. J Natl Cancer Inst 1993; 85: 365-76.

A'Hern RP. Sample size tables for exact single-stage phase II designs. Statist Med 2001, 20: 859-66.

American Cancer Society. Cancer Facts & Figures 2012. Atlanta: American Cancer Society; 2012.

Ascierto PA, Simeone E, Sileni VC et al. Sequential treatment with ipilimumab and BRAF inhibitors in patients with metastatic melanoma: data from the Italian cohort of the ipilimumab expanded access program. Cancer Invest 2014; 32 (4): 144-9.

Ascierto PA, Schadendorf D, Berking C, et al. MEK162 for patients with advanced melanoma harbouring NRAS or Val600 BRAF mutations: a non-randomised, open-label phase 2 study. Lancet Oncol 2013; 14: 249 – 256.

Ascierto PA, Simeone E, Giannarelli D et al. Sequencing of BRAF inhibitors and ipilimumab in patients with metastatic melanoma: a possible algorithm for clinical use. J Transl Med 2012; 10: 107.

Ascierto PA, Simeone E, Grimaldi AM et al. Do BRAF inhibitors select for populations with different disease progression kinetics?J Transl Med 2013; 11: 61.

Brooks R. EuroQoL: the current state of play. Health Policy 1996; 37 (1): 53-72.

Chapman PB, Hauschild A, Robert C et al. Improved survival with vemurafenib in melanoma with BRAF V600E mutation. N Engl J Med 2011; 364:2507-16.

Colombino M, Capone M, Lissia A et al. BRAF/NRAS mutation frequencies among primary tumors and metastases in patients with melanoma. J ClinOncol 2012; 30: 2522-9.

Ferlay J, Shin HR, Bray F et al. Estimates of worldwide burden of cancer in 2008: GLOBOCAN 2008. Int J Cancer. 2010 Dec 15; 127 (12): 2893-917.

Flaherty KT, Infante JR, DaudA et al. Combined BRAF and MEK inhibition in melanoma with BRAF V600 mutations. N Engl J Med 2012; 367 (18): 1694-703.

Flaherty KT, Puzanov I, Kim KB et al. Inhibition of Mutated, Activated BRAF in Metastatic Melanoma. NEJM 2010; 363: 809-19.

Greaves WO, Verma S, Patel KP et al. Frequency and Spectrum of BRAF Mutations in a Retrospective, Single-Institution Study of 1112 Cases of Melanoma. J MolDiagn 2013; 15: 220-6.

Hauschild A, Grob JJ, Demidov LV, et al. Dabrafenib in BRAF-mutated metastatic melanoma: a multicentre, open-label, phase 3 randomised controlled trial. Lancet 2012; 9839: 358-65.

Jakob JA, Bassett RL Jr, Ng CS et al. NRAS mutation status is an independent prognostic factor in metastatic melanoma.Cancer 2012; 118 (16): 4014-23.

Jemal A, Saraiya M, Patel P et al. Recent trends in cutaneous melanoma incidence and death rates in the United States, 1992-2006. J Am AcadDermatol 2011; 65 (5 Suppl 1): S17-25.

Lebbé C, Weber JS, Maio M et al. Survival follow-up and ipilimumab retreatment for patients with advanced melanoma who received ipilimumab in prior phase II studies.Ann Oncol 2014 Sep 10.

LGX818 Investigator's Brochure.

Martinez-Garcia M, Banerji U, Albanell J et al. First-in-human, phase I dose-escalation study of the safety, pharmacokinetics, and pharmacodynamics of RO5126766, a first-in-class dual MEK/RAF inhibitor in patients with solid tumors. Clin Cancer Res 2012; 18 (17): 4806-19.

McArthur G, Gonzalez R, Pavlick A, et al. Vemurafenib (VEM) and MEK inhibitor, cobimetinib (GDC0973), in advanced BRAF V600-mutated melanoma (BRIM-7): dose-exalation and expansion results of a phase Ib study. Eur J Cancer 2013; 49 (Suppl. 2): abstract 3703

MEK162 Investigator's Brochure.

Reilly MC, Zbrozek AS, Dukes EM. The validity and reproducibility of a work productivity and activity impairment instrument. Pharmacoeconomics 1993; 4 (5): 353-65.

Ribas A, Hodi FS, Callahan M et al. Hepatotoxicity with combination of vemurafenib and ipilimumab. N Engl J Med 2013; 368 (14): 1365-6.

Sosman JA, Kim KB, Schuchter L et al. Survival in BRAF V600- mutant advanced melanoma treated with vemurafenib. N Engl J Med 2012; 366:707–14.

Topalian SL, Sznol M, McDermott DF et al. Survival, durable tumor remission, and long-term safety in patients with advanced melanoma receiving nivolumab.J ClinOncol 2014; 32 (10): 1020-30.

Urner-Bloch U, Urner M, Stieger P et al. Transient MEK inhibitor-associated retinopathy in metastatic melanoma. Ann Oncol 2014; 25 (7): 1437-41.

Wolchok JD, Kluger H, Callahan MK et al. Nivolumab plus ipilimumab in advanced melanoma. N Engl J Med 2013; 369 (2): 122-33.

12. APPENDIX

I. Recommended guidelines for the management of study drug (LGX818 and MEK162) induced skin toxicity

Clinical judgment and experience of the treating physician should guide the management plan of each patient. In general, the following interventions are in addition to the rash dosing guidelines in Table 7of the protocol:

- Prophylaxis of skin toxicity to be initiated 24 hours prior to the first treatment with study drug or later as needed
- Application of topical agents to the most commonly affected skin areas such as face, scalp, neck, upper chest and upper back

Topical agents include non oily sunscreen (PABA free, SPF ≥ 30, UVA/UVB protection), topical steroids (preferably mometasone cream and topical erythromycin evening or topical pimocrolimus

Note: Topical agents should be applied on a daily basis starting on Day 1 of study treatment or 24 hours prior to the first dose, and more often as needed.

- Possibly oral doxycycline (100 mg daily) for the first 2-3 weeks of study drug administration.

Other effective medications are antihistamines, other topical corticosteroids, other topical antibiotics and low-dose systemic corticosteroids.

The treatment algorithm based on CTCAE grade is as follows:

Mild rash (CTCAE Grade 1)

- Consider prophylactic rash treatment if not already started
- Topical or other topical corticosteroid (i.e. mometasone cream) and/or topical antibiotic (i.e. erythromycin 2%) are recommended.
- The patient should be reassessed within a maximum of 2 weeks or as per investigator opinion.

Moderate rash (CTCAE Grade 2)

- Use of topical erythromycin or clindamycin (1%) plus topical mometasone or pimecrolimus cream (1%) plus oral antibiotics such as:,lymecycline (408 mg OD), doxycycline (100 mg BID) or minocycline (50 to 100 mg OD).
- Although there has been no evidence of phototoxicity or photosensitivity in patients being treated with LGX818 or MEK162, doxycycline (or minocycline as secondline) should be used with thorough UV protection (i.e., avoidance of direct exposure to sunlight, use of sunscreen and sunglasses, etc.).
- Use of acitretin is not recommended

Severe rash (CTCAE Grade 3-4)

CTCAE Grade 3

- In addition to the interventions recommended for moderate rash, consider oral prednisolone at a dose of 0.5 mg/kg. Upon improvement, taper the dose in a stepwise manner (25 mg for 7 days, subsequently decreasing the dose by 5 mg/day every day).
- Alternatively, in addition to the interventions recommended for moderate rash, consider oral isotretinoin (low doses, i.e. 0.3 to 0.5 mg/kg)
- Use of acitretin is not recommended

CTCAE Grade 4

- Immediately discontinue the patient from study drug and treat the patient with oral and topical medications (see recommendation CTCAE Grade 3).

Symptomatic treatment:

It is strongly recommended that patients who develop rash/skin toxicities receive symptomatic treatment:

- For pruritic lesions, use cool compresses and oral antihistaminic agents
- For fissuring, use Monsel's solution, silver nitrate, or zinc oxide cream. If not sufficient use mild steroid ointments or combinations of steroids and antibiotics
- For desquamation, use emollients with mild pH 5/neutral (best containing urea 10%)
- For paronychia, antiseptic bath and local potent corticosteroids, use oral antibiotics and if no improvement is seen, refer to a dermatologist or surgeon
- For infected lesions, obtain bacterial and fungal cultures and treat with topical or systemic antibiotics based on sensitivity of culture

II. Recommended guidelines for the management of MEK162 induced diarrhoea

Proactively investigate for occurrence of diarrhoea and educate patients

a. Remind patients at each visit to contact the Investigator immediately upon the first sign of loose stool or symptoms of abdominal pain. Additionally, at each study visit, each patient should be asked regarding occurrence of diarrhoea or diarrhoea-related symptoms. If the patient has symptoms, the patient should be asked regarding the actions taken for these symptoms and re-instruct if indicated

b. The patients should be instructed on dietary modifications and on early warning signs of diarrhoea and potentially life-threatening illnesses (e.g. severe cramping might be a sign for severe diarrhoea, fever with diarrhoea might be a sign for infection, fever and dizziness on standing might be a sign for shock)

c. Patients should be educated about what to report to the Investigator (i.e., number of stools, stool composition, stool volume)

Anti-diarrhoea therapy

In order to effectively manage diarrhoea and mitigate the escalation in severity or duration of diarrhoea, patient education as outlined above as well as proper management of diarrhoea is important.

Management of diarrhoea should be instituted at the first sign of abdominal cramping, loose stools or overt diarrhoea. All concomitant therapies used for treatment of diarrhoea must be recorded on the Concomitant Medications eCRF. It is recommended that patients be provided loperamide tablets and are instructed on the use of loperamide at on the first day of MEK162 treatment. In addition to the MEK162 induced diarrhea dosing guidelines in Table 7 of the protocol, these instructions should be provided at each visit and the site should ensure that the patient understands the instructions

Explain the frequency of diarrhoea and its relationship to NCI CTCAE grading.

Determine if diarrhoea is complicated or uncomplicated.

Rule out other or concomitant causes.

These may include:

- Infection with Candida, Salmonella, Clostridium difficile, Campylobacter, Giardia, Entamoeba and Cryptosporidium species can lead to severe infections in immunosuppressed patients
- Medication-induced diarrhoea
- Malabsorption/lactose intolerance
- Faecal impaction, partial bowel obstruction

For uncomplicated Grade 1/2 diarrhoea

- Stop all lactose-containing products, alcohol and eat frequent small meals that include bananas, rice, applesauce or toast)
- Stop laxatives, bulk fiber and stool softeners
- Stop high-osmolar food supplements
- Drink 8 to 10 large glasses of clear liquids per day
- Consider administration of standard dose of loperamide: initial administration 4 mg, then 2 mg every 4 hours (maximum of 16 mg/day) or after each unformed stool.
- Discontinue loperamide after 12-hours diarrhoea-free (Grade 0) interval.
- If uncomplicated Grade 1 to 2 diarrhoea persists for more than 24 hours, escalate to high dose loperamide: 2 mg every 2 hours (max. of 16 mg/day) or after each unformed stool.

Note: Oral antibiotics may be started as prophylaxis for infections under the discretion of the physician.

- If uncomplicated Grade 1 to 2 diarrhoea persists after 48 hours of treatment with loperamide, discontinue loperamide and begin a second-line agent which can be an opiate (opium tincture or paregoric), octreotide acetate or steroid (budesonide)

For complicated Grade 1/2 diarrhoea or any Grade 3 to 4 diarrhea

- The patient must call the investigator immediately
- If loperamide has not been initiated, initiate loperamide immediately. Initial administration 4 mg, then 2 mg every 4 hours (maximum of 16 mg/day) or after each unformed stool.
- Administer IV fluids and electrolytes as needed. In case of severe dehydration, replace loperamide by octreotide.
- Monitor/continue IV fluids and antibiotics as needed. Intervention should be continued until the patient is diarrhoea free for at least 24 hours.

Hospitalization may need to be considered.

III. Recommended algorithms for use of nivolumab and ipilimumab

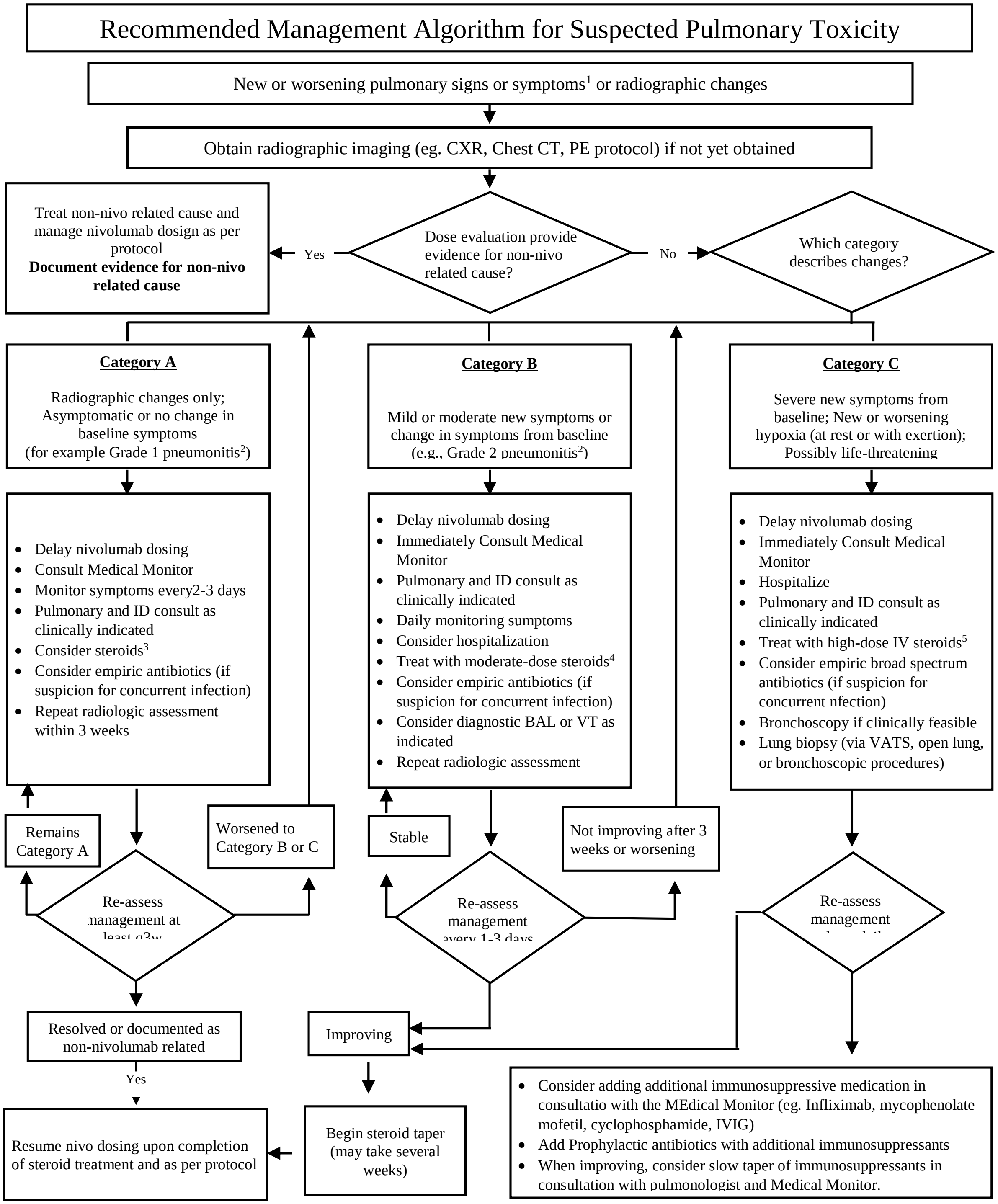

Footnotes

4. Signs and synptoms include dyspnea, cough, hypoxia and other respiratory compliants
5. Grading as per NCI CTCAE 4.03
6. Recommended initial costicosteroid regimen for category A: prednisone 60 mg/day PO or methylprednisone 1 mg/Kg/day IV
7. Recommended initial costicosteroid regimen for category B: prednisone 240 mg/day PO or methylprednisone 4 mg/Kg/day IV
8. Recommended initial costicosteroid regimen for category C: methylprednisone 1 g/Kg/day IV

Recommended Management Algorithm for Diarrhea or Colitis

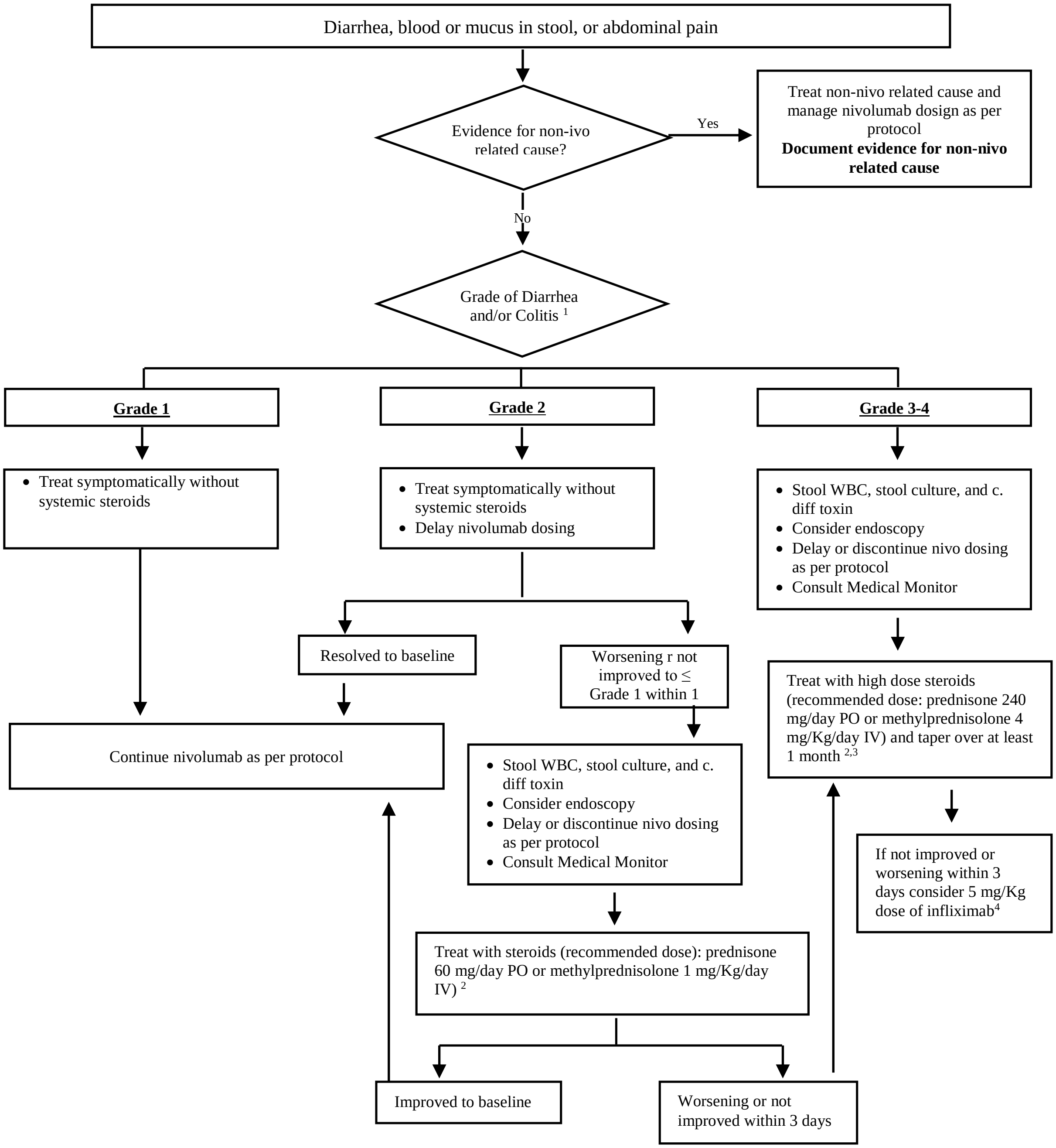

Footnotes

1. Grading as per NCI CTCAE version 4.03. If both diarrhea and colitis are present, manage as per toxicity with higher grade.
2. If infection work-up is positive, do not give steroids, stop following algorithm and treat specific infection.
3. If re-treatment with nivolumab is allowed as per protocol after completion of steroid taper, consult with Medical Monitor if considering re-treatment
4. Do not use infliximab if perforation or sepsis is present

Recommended Management Algorithm for Suspected Endocrinopathy

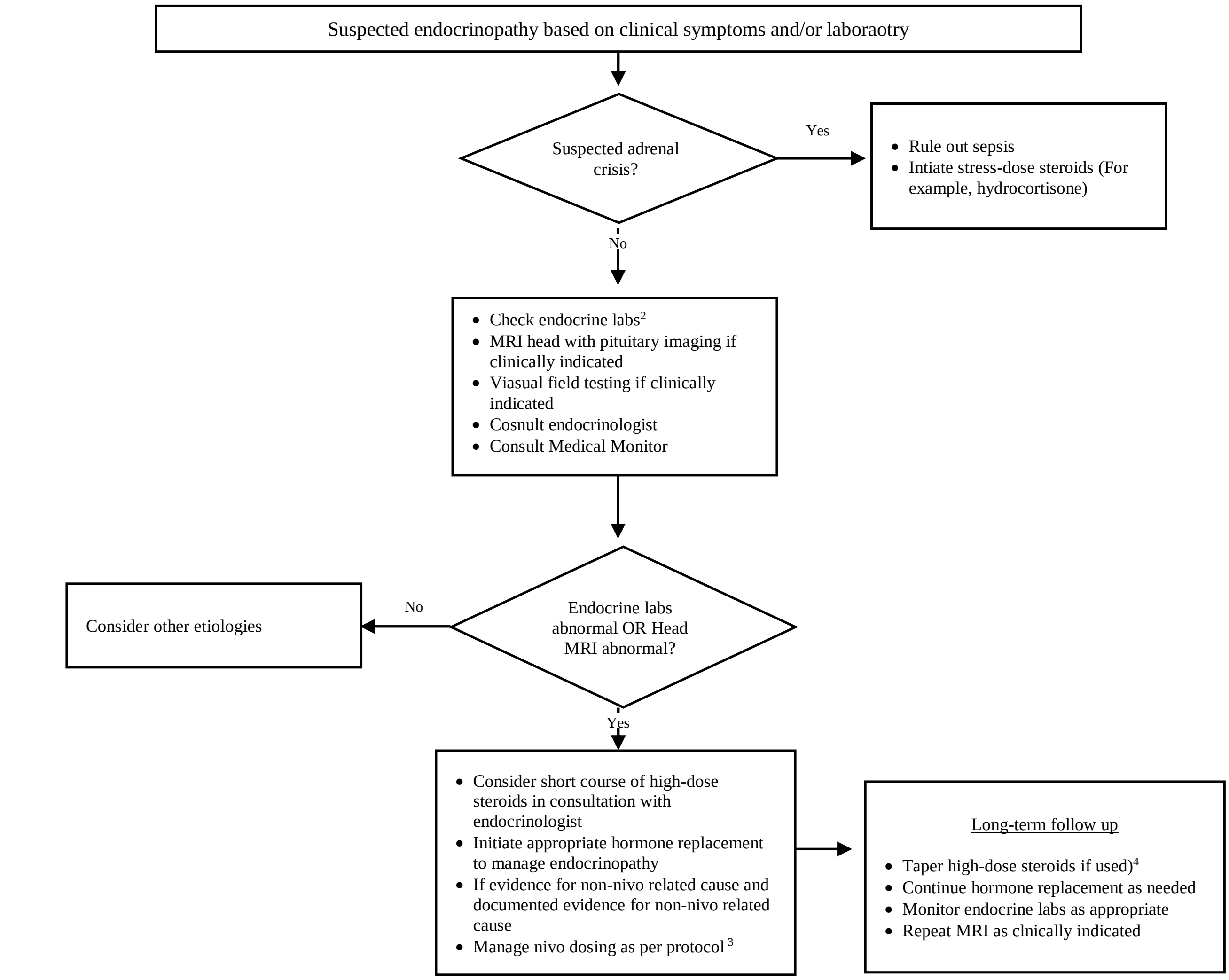

Footnotes

1. Cases have typically been identified through routine monitoring of laboratories or as part of a work-up for symptoms such as fatigue
2. It's important to draw labs at appropriate times; for example, certain labs should be drawn before giving steroids or at specific times of the day
3. Upon resolution or adequate treatment of endocrinopathy, patients may continue nivolumab dosing with appropriate hormone replacement unless limited by protocol
4. Patients may require chronic steroid replacement to maintain physiologic levels

Recommended Management Algorithm for Suspected Hepatotoxicity

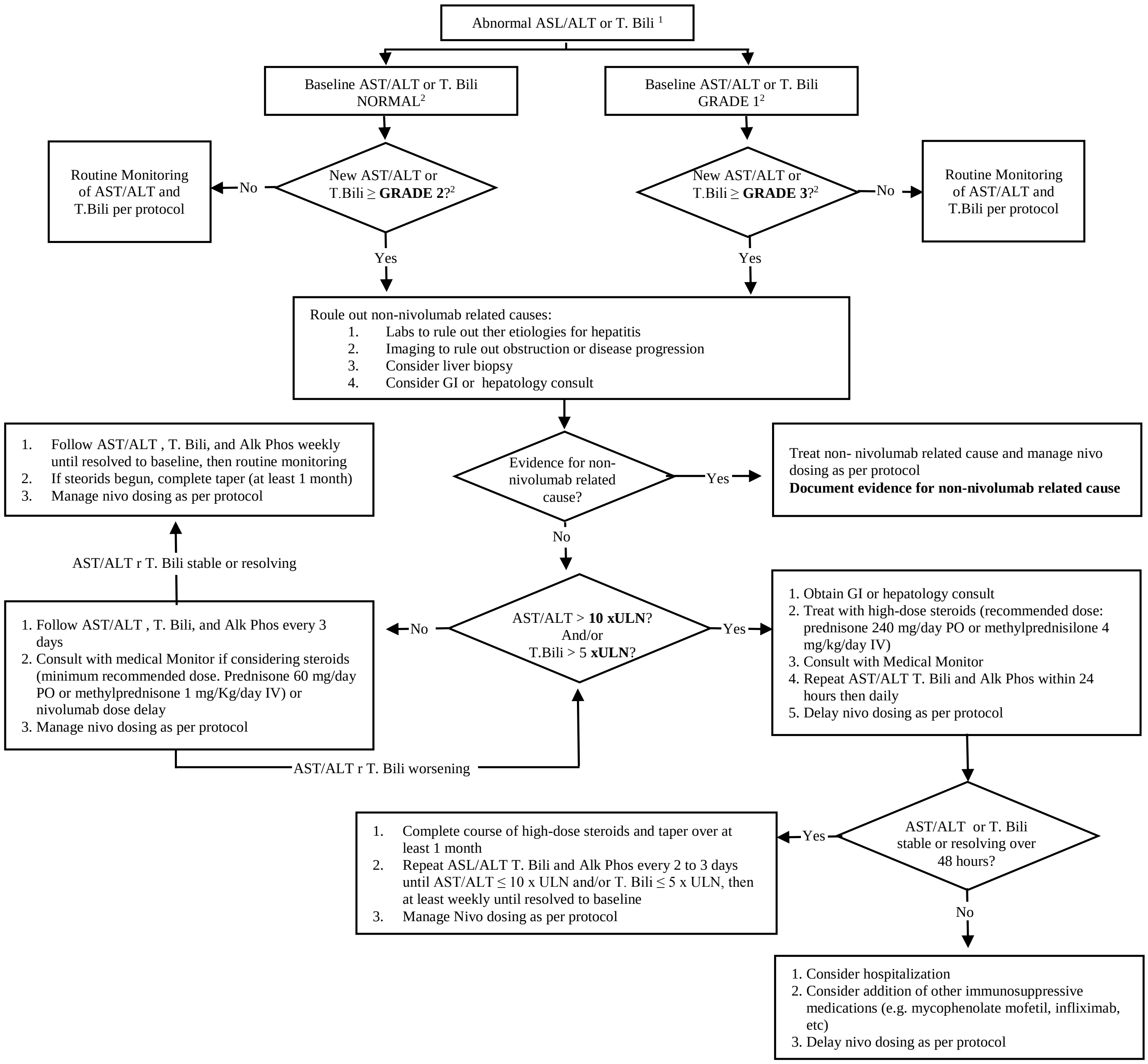

Footnotes

1. If elevations in both AST/ALT and T. Bili are present, the management of nivolumab dosing may be different than if only an isolated AST/ALT of T. Bili abnormality is present and may not be dependent on baseline values. Refer to the specific protocol if concurrent elevations occur
2. Grading as per NCI CTCAE versio 4.03

Recommended Management Algorithm for Nephrotoxicity

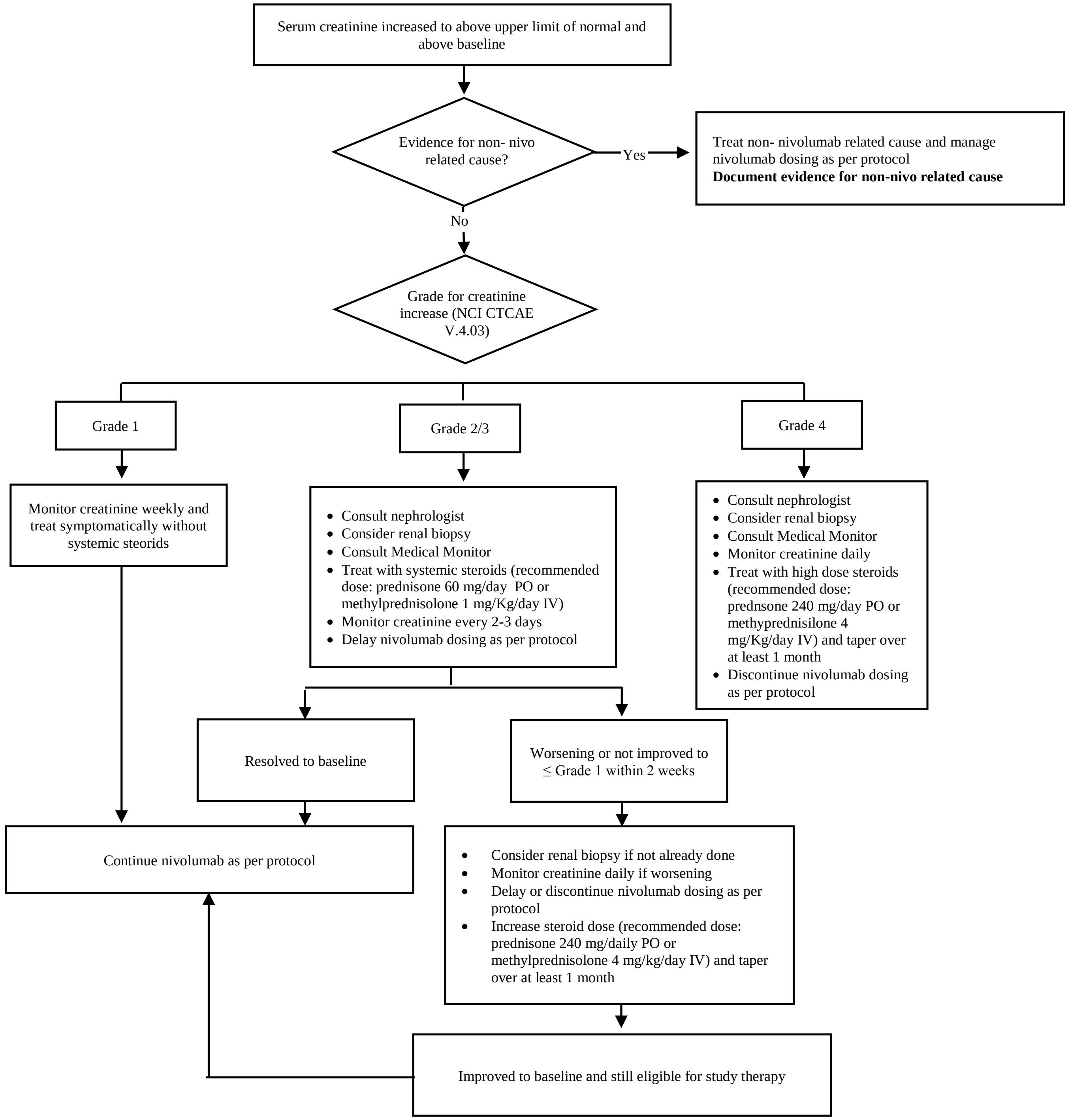

Skin toxicity Management Algorithm

**Severity of skin toxicity**	**Management**	**Follow-up**
Grade 1-2	• Symptomatic therapy,e.g. antihistamine, topical steroids<br>• Continue ipilimumab	**Symptoms resolve**: Continue ipilimuamb<br>**Symptoms persist > 1-2 weeks or recur**:<br>o Continue ipilimumab<br>o Moderate to high dose steroids PO (e.g. prednisole 0.5 – 1 mg/Kg/day)<br>o Once controlled, taper steroids over at least 1 month)
Grade 3-4	• Hold/delay ipilimumab (regardless of relationship)<br>• Take photos of rash<br>• Consider skin biopsy<br>• Dermatology consult<br>• High dose IV steroids (e.g. methylprednisolone 1-2 mg/kg/day)	**Symptoms resolve/ return to grade 1:**<br>o Taper steroids over the least 1 month<br>o Resume ipilimumab<br>o Discontinue if grade 4 toxicity consider related

Rule out non-inflammatory cause. If non.inflammatory cause, treat accordingly and continue ipilimumab

Patients on IV steroids may be switched to oral corticosteroid (e.g. prednisone) at an equivalent dose at start or tapering or earlier once sustained clinical improvement is observed. Lower bioavailability of oral costicosteroids should be taken into account when switching to the equivalent dose of PO costicosteroids.

Neurological toxicity Management Algorithm

Rule out non-inflammatory cause. If non.inflammatory cause, treat accordingly and continue ipilimumab.
Discontinue ipilimumab for any grade 3-4 motor neuropathy, regardless of relationship.

**Severity of neurological toxicity**	**Management**	**Follow-up**
Grade 1	Continue ipilimumab	Continue monitoring the patient. If symptoms worsen, treat as below
Grade 2	• Hold/ delay ipilimumab<br>• Treat symptoms per local guidelines	Resume ipilimumab when resolved/ grade 1<br>If symptoms worsen, treat as below
Grade 3/4 sensory	• Discontinue ipilimumab if consider related<br>• Obtain neuro consult<br>• Treat symptoms per local guidelines<br>• High dose IV steroids (e.g. 1-2 mg/kg/day methulprenisolone)	Symptoms resolve/return to grade 1:<br>○ Taper steroids over at least 1 month
Grade 3/4 motor	• Discontinue ipilimumab **<u>regardless of relationship</u>**<br>• Obtain neuro consult<br>• Treat symptoms per local guidelines<br>• High dose IV steroids (e.g. 1-2 mg/kg/day methylprednisolone)	Symptoms resolve/return to grade 2:<br>○ Taper steroids over at least 1 month<br>Symptoms do not resolve or progress; atypical presentation:<br>○ Consider IVIg or other immunosuppressive therapies per local guidelines

Patients on IV steroids may be switched to oral corticosteroid (e.g. prednisone) at an equivalent dose at start or tapering or earlier once sustained clinical improvement is observed. Lower bioavailability of oral costicosteroids should be taken into account when switching to the equivalent dose of PO costicosteroids.

IV. EORTC QLQ-C30 (version 3)

EORTC QLQ-C30 (version 3)

We are interested in some things about you and your health. Please answer all of the questions yourself by circling the number that best applies to you. There are no "right" or "wrong" answers. The information that you provide will remain strictly confidential.

Please fill in your initials: |__|__|__|__|

Your birthdate (Day, Month, Year): |__|__|__|__|__|__|__|__|

Today's date (Day, Month, Year): 31 |__|__|__|__|__|__|__|__|

		Not at All	A little	Quite Bit	Very Much
1.	Do you have any trouble doing strenuous activities, like carrying a heavy shopping bag or a suitcase?	1	2	3	4
2.	Do you have any trouble taking a long walk?	1	2	3	4
3.	Do you have any trouble taking a short walk outside of the house?	1	2	3	4
4.	Do you need to stay in bed or a chair during the day?	1	2	3	4
5.	Do you need help with eating, dressing, washing yourself or using the toilet?	1	2	3	4

During the past week:

		Not at All	A little	Quite Bit	Very Much
6.	Were you limited in doing either your work or other daily activities?	1	2	3	4
7.	Were you limited in pursuing your hobbies or other leisure time activities?	1	2	3	4
8.	Were you short of breath?	1	2	3	4
9.	Have you had pain?	1	2	3	4
10.	Did you need to rest?	1	2	3	4

11.	Have you had trouble sleeping?	1	2	3	4
12.	Have you felt weak?	1	2	3	4
13.	Have you lacked appetite?	1	2	3	4
14.	Have you felt nauseated?	1	2	3	4
15.	Have you vomited?	1	2	3	4
16.	Have you been constipated?	1	2	3	4
17.	Have you had diarrhea?	1	2	3	4
18	Were you tired?	1	2	3	4
19.	Did pain interfere with your daily activities?	1	2	3	4
20.	Have you had difficulty in concentrating on things, like reading a newspaper or watching television?	1	2	3	4
21.	Did you feel tense?	1	2	3	4
22.	Did you worry?	1	2	3	4
23	Did you feel irritable?	1	2	3	4
24.	Did you feel depressed?	1	2	3	4
25	Have you had difficulty remembering things?	1	2	3	4
26	Has your physical condition or medical treatment interfered with your <u>family</u> life?	1	2	3	4
27	Has your physical condition or medical treatment interfered with your <u>social</u> activities?	1	2	3	4
28.	Has your physical condition or medical treatment caused you financial difficulties?	1	2	3	4

For the following questions please circle the number between 1 and 7 that best applies to you:

29. How would you rate your overall health during the past week?

1	2	3	4	5	6	7
Very Poor						Excellent

30. How would you rate your overall quality of life during the past week?

1	2	3	4	5	6	7
Very Poor						Excellent

V. EQ-5D

Under each heading, please tick the ONE box that best describes your health TODAY.

MOBILITY

I have no problems in walking about ☐
I have slight problems in walking about ☐
I have moderate problems in walking about ☐
I have severe problems in walking about ☐
I am unable to walk about ☐

SELF-CARE

I have no problems washing or dressing myself ☐
I have slight problems washing or dressing myself ☐
I have moderate problems washing or dressing myself ☐
I have severe problems washing or dressing myself ☐
I am unable to wash or dress myself ☐

USUAL ACTIVITIES *(e.g. work, study, housework, family or leisure activities)*

I have no problems doing my usual activities ☐
I have slight problems doing my usual activities ☐
I have moderate problems doing my usual activities ☐
I have severe problems doing my usual activities ☐
I am unable to do my usual activities ☐

PAIN / DISCOMFORT

I have no pain or discomfort ☐
I have slight pain or discomfort ☐
I have moderate pain or discomfort ☐
I have severe pain or discomfort ☐
I have extreme pain or discomfort ☐

ANXIETY / DEPRESSION

I am not anxious or depressed ☐
I am slightly anxious or depressed ☐

I am moderately anxious or depressed ☐
I am severely anxious or depressed ☐
I am extremely anxious or depressed ☐

We would like to know how good or bad your health is TODAY.

This scale is numbered from 0 to 100.
100 means the best health you can imagine.
0 means the worst health you can imagine.

Mark an X on the scale to indicate how your health is TODAY.

Now, please write the number you marked on the scale in the box below.

YOUR HEALTH TODAY [ ]

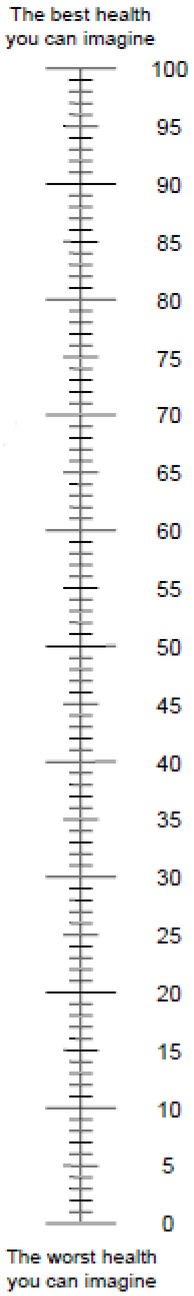

VI. WPAI:GH

**Work Productivity and Activity Impairment Questionnaire:
General Health V2.0 (WPAI:GH)**

The following questions ask about the effect of your health problems on your ability to work and perform regular activities. By health problems we mean any physical or emotional problem or symptom. *Please fill in the blanks or circle a number, as indicated.*

1. Are you currently employed (working for pay)? ____ NO ____ YES
   If NO, check "NO" and skip to question 6.

The next questions are about the **past seven days**, not including today.

2. During the past seven days, how many hours did you miss from work because of your health problems? *Include hours you missed on sick days, times you went in late, left early, etc., because of your health problems. Do not include time you missed to participate in this study.*

   _____HOURS

3. During the past seven days, how many hours did you miss from work because of any other reason, such as vacation, holidays, time off to participate in this study?

   _____HOURS

4. During the past seven days, how many hours did you actually work?

   _____HOURS *(If "0", skip to question 6.)*

5. During the past seven days, how much did your health problems affect your productivity while you were working?

Think about days you were limited in the amount or kind of work you could do, days you accomplished less than you would like, or days you could not do your work as carefully as usual. If health problems affected your work only a little, choose a low number. Choose a high number if health problems affected your work a great deal.

Consider only how much health problems affected productivity while you were working.

Health problems had no effect on my work	0	1	2	3	4	5	6	7	8	9	10	Health problems completely prevented me from working

CIRCLE A NUMBER

6. During the past seven days, how much did your health problems affect your ability to do your regular daily activities, other than work at a job?

By regular activities, we mean the usual activities you do, such as work around the house, shopping, childcare, exercising, studying, etc. Think about times you were limited in the amount or kind of activities you could do and times you accomplished less than you would like. If health problems affected your activities only a little, choose a low number. Choose a high number if health problems affected your activities a great deal.

Consider only how much health problems affected your ability to do your regular daily activities, other than work at a job.

Health problems had no effect on my daily activities	0	1	2	3	4	5	6	7	8	9	10	Health problems completely prevented me from doing my daily activities

CIRCLE A NUMBER

VII. ECOG Performance Status

**Grade**	**ECOG**
0	Fully active, able to carry on all pre-disease performance without restriction
1	Restricted in physically strenuous activity but ambulatory and able to carry out work of a light or sedentary nature, e.g., light house work, office work
2	Ambulatory and capable of all selfcare but unable to carry out any work activities. Up and about more than 50% of waking hours
3	Capable of only limited selfcare, confined to bed or chair more than 50% of waking hours
4	Completely disabled. Cannot carry on any selfcare. Totally confined to bed or chair
5	Dead

CLINICAL STUDY PROTOCOL

SECOMBIT

"Sequential Combo Immuno and Target therapy (SECOMBIT) study"

"A three arms prospective, randomized phase II study to evaluate the best sequential approach with combo immunotherapy (ipilimumab/nivolumab) and combo target therapy (LGX818/MEK162) in patients with metastatic melanoma and BRAF mutation"

Global Chief Investigator (PI): **Dr. Paolo A. Ascierto**
Istituto Nazionale dei Tumori, Fondazione "G. Pascale"
U.O.C. Melanoma, Immunoterapia Oncologica e Terapie Innovative
Via M. Semmola 80131 - Naples

Protocol code: SECOMBIT
Protocol version: 10.0
Protocol date: 24 May 2019
EudraCT number: 2014-004842-92
Sponsor: Fondazione Melanoma (ONLUS)
c/o Istituto Tumori Napoli
Fondazione "G. Pascale"
Via Mariano Semmola 80131 Naples, Italy

CONFIDENTIAL

This document contains confidential information belonging to the Sponsor. Except as may be otherwise agreed to in writing, by accepting or reviewing these materials, you agree to hold such information in confidence and not to disclose it to others (except where required by applicable law), nor to use it for unauthorized purposes. In the event of actual or suspected breach of this obligation Sponsor should be promptly notified.

SPONSOR SIGNATURE

Dr. Gennaro Parisi
Fondazione Melanoma ONLUS
Sponsor representative

09 June 2021

Signature Date

GLOBAL CHIEF INVESTIGATOR

Dr. Paolo A. Ascierto
Istituto Nazionale dei Tumori, Fondazione "G. Pascale"
U.O.C. Melanoma, Immunoterapia Oncologica e Terapie Innovative
Via M. Semmola 80131 - Naples

09 June 2021

Signature Date

STATISTICIAN

Dr. Diana Giannarelli
IFO- Istituto Nazionale Tumori Regina Elena
Unità di Biostatistica
Via Elio Chianesi, n. 53 - (Eur) - Roma

09 June 2021

Signature Date

PROTOCOL APPROVAL

CLINICAL STUDY PROTOCOL

SECOMBIT

"Sequential Combo Immuno and Target therapy (SECOMBIT) study"

"A three arms prospective randomized phase II study to evaluate the best sequential approach with combo immunotherapy (ipilimumab/nivolumab) and combo target therapy (LGX818/MEK162) in patients with metastatic melanoma and BRAF mutation"

I agree to the terms of this study protocol. I will conduct the study according to the procedures specified herein, and according to principles of Good Clinical Practices and local regulations and requirements.

Investigator:

Site Number: ____________________________________

Name: ____________________________________

Signature: ____________________________________

Date: _________________

ADDITIONAL TRIAL PERSONNEL/SITE INFORMATION

**Principal Investigators (PI)**	**Dr. Paolo A. Ascierto**<br>Istituto Nazionale dei Tumori, Fondazione "G. Pascale"<br>U.O.C. Melanoma, Immunoterapia Oncologica e Terapie Innovative<br>Via M. Semmola 80131 - Naples	Tel: +39 081 5903 236<br>Fax: +39 081 5903 841<br>Email paolo.ascierto@gmail.com
**Study coordinator and Data management**	**Dr. Marcello Curvietto**<br>Istituto Nazionale dei Tumori, Fondazione "G. Pascale"<br>U.O.C. Melanoma, Immunoterapia Oncologica e Terapie Innovative<br>Via M. Semmola 80131 - Naples	Tel: +39 081 5903 841<br>Fax: +39 081 5903 841<br>Email curvietto.ma@gmail.com
**Statistician**	**Dr. Diana Giannarelli**<br>IFO- Istituto Nazionale Tumori Regina Elena<br>Unità di Biostatistica<br>Via Elio Chianesi, n. 53 - (Eur) - Roma	Tel: +39 06 52665607<br>Fax: +39 06 52662463<br>Email giannarelli@ifo.it
**Safety desk (SAE reporting)**	**Clinical Research Technology srl**<br>Pharmacovigilance Unit	Tel: +39 089.301545<br>Fax: +39 089.7724155<br>e-mail: pvg@cr-technology.com

**Emergency contact**	**Dr. Paolo A. Ascierto**<br>Istituto Nazionale dei Tumori, Fondazione "G. Pascale"<br>U.O.C. Melanoma, Immunoterapia Oncologica e Terapie Innovative<br>Via M. Semmola 80131 - Naples	Tel: +39 081 5903 236<br>Fax: +39 081 5903 841<br>Email :paolo.ascierto@gmail.com
	**Dr. Antonio Maria Grimaldi**<br>Istituto Nazionale dei Tumori, Fondazione "G. Pascale"<br>U.O.C. Melanoma, Immunoterapia Oncologica e Terapie Innovative<br>Via M. Semmola 80131 - Naples	Tel: +39 081 5903 236<br>Fax: +39 081 5903 841<br>E-mail: dott.a.m.grimaldi@gmail.com
	**Clinical Research Technology srl**<br>Project Management	Tel: +39 089.301545<br>Fax: +39 089.7724155<br>e-mail: pvg@cr-technology.com

TABLE OF CONTENTS

SECOMBIT 1
TABLE OF CONTENTS 5
LIST OF TABLES 7
PROTOCOL SYNOPSIS 8
LIST OF ABBREVIATIONS 17
1. SCHEDULE OF ASSESSMENTS 20
ARM A 20
ARM B 22
ARM C 24
2. BACKGROUND AND RATIONALE 26
2.1 OVERVIEW OF DISEASE EPIDEMIOLOGY AND CURRENT TREATMENT 26
2.2 INTRODUCTION TO INVESTIGATIONAL TREATMENTS AND OTHER STUDY TREATMENT(S) 28
2.3 STUDY RATIONALE 33
2.4 FINAL RISK/BENEFIT STATEMENT 35
3. STUDY AIMS AND DESIGN 38
3.1 OBJECTIVES OF THE STUDY 38
3.2 ENDPOINTS OF THE STUDY 39
3.3 STUDY DESIGN 41
3.4 STUDY SCHEDULE 42
3.5 SCHEDULE OF ASSESSMENTS AND PROCEDURES 44
4. STUDY POPULATION 62
4.1 INCLUSION CRITERIA 62
4.2 EXCLUSION CRITERIA 63
4.3 STUDY TREATMENTS 65
4.4 CONCOMITANT MEDICATION AND TREATMENT 65
4.5 DRUG INTERACTION 66
4.6 CRITERIA FOR PREMATURE WITHDRAWAL 67
4.7 DEFINITION OF END OF TRIAL 68
5. INVESTIGATIONAL MEDICINAL PRODUCTS 70
5.1 COMBO TARGET 70
5.2 COMBO IMMUNO 81
5.3 ACCOUNTABILITY, ASSESSMENT OF COMPLIANCE AND DESTRUCTION OF THE DRUGS 87
5.4 TREATMENT AFTER THE END OF THE STUDY 89
6. STATISTICAL CONSIDERATIONS 90
6.1 STUDY ENDPOINTS 90
6.2 SAMPLE SIZE AND ANALYSIS POPULATIONS 90
6.3 STUDY DURATION 94
7. SAFETY INSTRUCTIONS AND GUIDANCE 95
7.1 WARNING AND PRECAUTIONS 95
7.2 ADVERSE EVENTS AND LABORATORY ABNORMALITIES 95
7.3 TREATMENT AND FOLLOW-UP OF AEs (100 DAYS POST DISCONTINUATION OF STUDY DRUGS) 98
7.4 LABORATORY TEST ABNORMALITIES 99
7.5 HANDLING OF SAFETY PARAMETERS 100
8. DATA COLLECTION AND MANAGEMENT 105
8.1 DATA CONFIDENTIALITY 105
8.2 SITE MONITORING 105
8.3 DATA COLLECTION 106

8.4 DATABASE MANAGEMENT AND QUALITY CONTROL 106
8.5 DATA MONITORING COMMITTEE 106
9. ETHICAL CONSIDERATION 108
9.1 REGULATORY AND ETHICAL COMPLIANCE 108
9.2 RESPONSIBILITIES OF THE INVESTIGATOR AND IEC 108
9.3 INFORMED CONSENT PROCEDURES 108
9.4 PUBLICATION OF STUDY PROTOCOL AND RESULTS 108
9.5 STUDY DOCUMENTATION, RECORD KEEPING AND RETENTION OF DOCUMENTS 108
9.6 CONFIDENTIALITY OF STUDY DOCUMENTS AND PATIENT RECORDS 109
9.7 AUDITS AND INSPECTIONS 109
9.8 FINANCIAL DISCLOSURES 109
10. PROTOCOL ADHERENCE 110
10.1 AMENDMENTS TO THE PROTOCOL 110
11. REFERENCES 111
12. APPENDIX 113
I. RECOMMENDED GUIDELINES FOR THE MANAGEMENT OF STUDY DRUG (LGX818 AND MEK162) INDUCED SKIN TOXICITY 113
II. RECOMMENDED GUIDELINES FOR THE MANAGEMENT OF MEK162 INDUCED DIARRHOEA 115
III. RECOMMENDED ALGORITHMS FOR USE OF NIVOLUMAB AND IPILIMUMAB 117
IV. EORTC QLQ-C30 (VERSION 3) 124
V. EQ-5D 126
VI. WPAI:GH 128
VII. ECOG PERFORMANCE STATUS 130

LIST OF TABLES

Table 1: Schedule of Assessments and Procedures in Arm A 21
Table 2: Schedule of Assessments and Procedures in Arm B 23
Table 3: Schedule of Assessments and Procedures in Arm C 25
Table 4: Summary of study aim – Principal Study 41
Table 5: Summary of study aim – Traslational Study 41
Table 6: Study treatment scheme 43
Table 7: Treatments used in the study 65
Table 8: Combo Target dose and treatment schedule 70
Table 9: Dose reduction for LGX818 and MEK162............ 71
Table 10: Recommended dose modifications associated with treatment-related adverse events........ 79
Table 11: Packaging and labelling 80
Table 12: Combo Immuno dose and treatment schedule 81

PROTOCOL SYNOPSIS

**Study Title**	A three arms prospective randomized phase II study to evaluate the best sequential approach with combo immunotherapy (ipilimumab/nivolumab) and combo target therapy (LGX818/MEK162) in patients with metastatic melanoma and BRAF mutation.
**Study ID**	**SECOMBIT** (Sequential Combo Immuno and Target therapy study)
**Number of participanting sites**	Participating sites: Approximately 30 sites in Italy and Europe<br>**Global Chief Investigator:**<br>Paolo Antonio Ascierto<br>e-mail: paolo.ascierto@gmail.com<br>Tel: +39 0815903431<br>Fax: +39 0815903841<br>Mobile: +39 338 7402333
**Phase**	II
**Study Hypothesis**	To evaluate the best sequencing approach with the combination of target agents (LGX818 plus MEK162) and the combination of immunomodulatory antibodies (ipilimumab plus nivolumab) in patients with metastatic melanoma and BRAF V600 mutation.
**Background and rationale**	The combination BRAF (B-raf murine sarcoma viral oncogene homolog B1) inhibitor plus mitogen-activated protein kinase (MEK) inhibitor seems to be more effective in the V600 BRAF mutated advanced melanoma patients compared to treatment with the BRAF inhibitors alone. In fact, a phase I-II study (*Flaherty et al, 2012*) showed a better overall response rate (ORR) and progression-free survival (PFS) in the combination arm (dabrafenib plus trametinib) with respect to the single agent treatment (dabrafenib): 76% and 9.4 months versus 54% and 5.8 months respectively. Another phase I study with a similar combination (vemurafenib plus cobimetinib) showed an ORR of 85% in vemurafenib-naïve patients (*Martinez Garcia et al, 2012*)<br>Recently, the results of a phase I study about the combination ipilimumab plus nivolumab have been reported (*Wolchock et al, 2013*). In this study at the selected schedule (ipilimumab 3 mg/kg and nivolumab 1 mg/kg), 53% of patients had an objective response, all with tumor reduction of 80% or more. Responses were durable, although longer follow-up is needed.<br>A recent phase I study has shown a high rate of liver toxicity with the combo ipilimumab plus vemurafenib (*Ribas et al, 2013*) which makes difficult a combination with these two different drugs. Moreover, a better efficacy of the sequencing treatment BRAF inhibitors/ipilimumab vs. the single agent treatment was also observed; for this reason it was also suggested to start immunotherapy treatment in the BRAF V600 mutated melanoma population as first option, in order to increase the percentage of patients who can benefit from the sequencing (*Ascierto et al, 2012*; *Ascierto et al, 2013*), considering the possibility of a fast progression of the disease after the BRAF inhibitors treatment (*Ascierto et al, 2012*).

	Taking into account these considerations, it seems impossible to think to combine all the four compounds (the target agents and immunomodulating monoclonal antibodies). The risk of a high rate of toxicity is realistic and would render this approach inapplicable.<br>Sequencing with these different combinations seems to be more feasible. However, also in this case it would be important to start with the best combination in order to give to the patients the best chance to increase the overall survival.<br>The aim of this prospective randomized phase II study is to evaluate the sequencing of these two different combinations and evaluate which is the best of these approaches.
**Primary Objectives**	To define the best sequencing combination treatment in primary efficacy variable overall survival (OS).
**Secondary Objectives**	To evaluate the effects of the two sequencing combination treatments on:<br>• Total PFS;<br>• 3 years PFS rate;<br>• Percentage of patients alive at 3 years;<br>• Best overall response rate (BORR);<br>• Duration of response (DoR);<br>• Toxicity of the investigational medicinal products (IMPs)<br>• Quality of life and general health status defined by:<br>o Health-related quality of life (HRQoL), by means of the 30-item European Organisation for Research and Treatment of Care quality of life questionnaire (EORTC QLQ-C30);<br>o General health status, by means of the European Quality of Life 5-Dimensions(EQ-5D) questionnaire;<br>o Impairment of work productivity and activity, by means of the Work Productivity and Activity Impairment: General Health (WPAI:GH) questionnaire.<br>Biological markers (biomarkers study)<br>The objective of the biomarker study is to focus on understanding mechanisms of action/resistance. In particular, the biomarker study:<br>• Will inform how to sequence targeted RAF/MEK agents with immunotherapy agents (i.e. ipilumumab and nivolumab) in melanoma;<br>• Will be hypothesis-generating only.
**Study plan**	The study will be conducted according to an open-label, prospective, randomized, phase II design.<br>Randomization will be stratified according to stage arranged in the 3 following strata:<br>• IIIb/c – M1a – M1b;<br>• M1c with normal LDH (≤ 2 ULN);<br>• M1c with elevated LDH (> 2 ULN).<br>Subjects will be assessed for response by computed tomography (CT) or Magnetic Resonance Imaging (MRI).

	All measurable and non-measurable lesions must be documented at screening (within 28 days prior to randomization) and re-assessed at each subsequent tumor evaluation (every 8 weeks (+/- 1 week) for the first year, every 12 weeks (+/- 1 week) while the patient is on study). Tumor assessments including measurable and non-measurable lesions (baseline brain CT or MRI, CT/MRI C/A/P, CT/MRI of brain as clinically indicated, bone scan if clinically indicated). Tumor assessments with CT or MRI scans of the brain, chest, abdomen, and pelvis will be performed until disease progression after the second combo treatment per RECIST v1.1. Imaging of the neck should be included, if clinically indicated. In the event positron emission tomography (PET)/CT scanner is used for tumor assessments, the CT portion of the PET/CT must meet criteria for diagnostic quality. All scans will be collected for a possible independent review.<br>When patients discontinue study treatment for reason other than investigator–determined disease progression, tumor assessments should continue to be performed as scheduled for long term follow-up<br>Follow up visit is to be performed within 28 days from discontinuation of treatments and thereafter every 12 weeks until 60 months from randomization for long term survival follow-up. For patients with palpable/superficial lesions, clinical disease assessments by physical examination should be performed at baseline and throughout study treatment as clinically indicated. Color photographs with ruler/calipers will be taken at baseline and at all subsequent tumor assessment time points.<br>The National Cancer Institute Common Toxicity Criteria for Adverse Events (NCI CTC-AE) Version 4.03 will be used to evaluate the clinical safety of the treatment in this study. Patients will be assessed for AEs at each clinical visit and as necessary throughout the study.<br><br>Biomarker study<br>A correlative biological study will be performed for the evaluation of biomarkers on the biological samples available (paraffin-embedded tissue, frozen tissue, blood, serum, etc.).
**Sample size**	A total of 230 patients will be enrolled to ensure a minimum of 207 randomized patients.
**Study population**	The study population will include patients of either sex aged ≥ 18 years with metastatic melanoma and BRAF V600 mutation
**Inclusion criteria**	1) Patients of either sex aged ≥ 18 years;<br>2) Histologically confirmed stage III (unresectable) or stage IV melanoma with the BRAF V600 mutation. Patients with mucosal melanoma (but not those with ocular melanoma) are eligible for study participation;<br>3) Treatment naïve for metastatic disease patients. Previous adjuvant treatment, included checkpoint inhibitors anti CTLA-4, anti PD-1/PDL-1 is allowed, except for stage IV (if completed at least 6 weeks prior to randomization, and all related adverse events have either returned to baseline or stabilized). BRAF inhibitor treatment in adjuvant setting is not permitted.<br>4) Measurable disease by computed tomography (CT) or Magnetic Resonance Imaging (MRI) per RECIST 1.1 criteria;

	5) Presence of BRAF V600E or V600K mutation in tumor tissue prior to enrollment;<br>6) Eastern Cooperative Oncology Group (ECOG) performance status (PS) 0 or 1;<br>7) Tumor tissue from an unresectable or metastatic site of disease must be provided for biomarker analyses. An archive sample is mandatory at the screening visit; however, a new sample collection would be preferable;<br>8) Female subjects of childbearing potential must have a negative pregnancy test result at baseline and must practice two highly effective methods of contraception for the total study duration plus 23 weeks (i.e. 30 days plus the time required for nivolumab to undergo five half lives) after the last dose of nivolumab and ipilimumab and 30 days after the last dose of binimetinib and encorafenib for female subjects. Additional pregnancy testing must be performed every 6 weeks during the treatment Combo-Immuno and every 4 weeks during the treatment Combo-Target, as well as at the end of the systemic exposure;<br>9) Men who are sexually active with women of childbearing potential must practice a reliable method of contraception for the total study duration plus 31 weeks (i.e. 80 days plus the time required for nivolumab to undergo five half lives) after the last dose of nivolumab and ipilimumab and 90 days after the last dose of binimetinib and encorafenib;<br>10) Adequate bone marrow haematological function: absolute neutrophil count (ANC) $\geq$ 1.5 x 10^9/L AND platelet count $\geq$ 100 x 10^9/L AND haemoglobin $\geq$ 9 g/dL;<br>11) Adequate liver function: total bilirubin $\leq$ 1.5 x upper limit of normal (ULN) AND aspartate aminotransferase (AST)/alanine aminotransferase (ALT) $\leq$ 2.5 X ULN (< 5 x ULN if liver metastases);<br>12) Adequate renal function: serum creatinine $\leq$ 1.5 mg/dL OR creatinine clearance $\geq$ 60 mL/min in males and $\geq$ 50 mL/min in females (calculated according to Cockroft-Gault formula);<br>13) Serum calcium levels, international normalised ratio (INR) and partial thromboplastin time were within normal limits;<br>14) Life expectancy of at least 3 months;<br>15) Ability to understand study-related patient information and provision of written informed consent for participation in the study.<br>16) Adequate electrolytes at Baseline, defined as serum potassium and magnesium levels within institutional normal limits (Note: replacement treatment to achieve adequate electrolytes will be allowed).<br>17) Adequate cardiac function:<br>• left ventricular ejection fraction (LVEF) $\geq$ 50% as determined by a multigated --acquisition (MUGA) scan or echocardiogram,<br>• QTc interval $\leq$ 480 ms (preferably the mean from triplicate ECGs)
**Exclusion criteria**	1) Active brain metastases. Subjects with brain metastases are eligible if these have been treated and there is no magnetic resonance imaging (MRI) evidence of progression for at least 4 weeks after treatment is complete and within 28 days prior to first dose of study drug administration. There must also be no requirement for immunosuppressive doses of systemic corticosteroids (> 10 mg/day prednisone equivalents) for at least 2 weeks prior to study drug administration;<br>2) Subjects with active, known or suspected autoimmune disease;

	3) Subjects with a condition requiring systemic treatment with either corticosteroids (>10 mg daily prednisone equivalents) or other immunosuppressive medications within 14 days of treatment;<br>4) Prior treatment for stage III (unresectable) or stage IV melanoma with an anti-Programmed Death receptor-1 (PD-1), anti-Programmed Death-1 ligand-1 (PD-L1), anti-PD-L2, or anti-cytotoxic T lymphocyte associated antigen-4 (anti-CTLA-4) antibody;<br>5) Female subjects who are pregnant (positive pregnancy test), breast-feeding, or who are of childbearing potential and not practicing a reliable method of birth control;<br>6) Evidence of severe or uncontrolled systemic disease or any concurrent condition which in the investigator's opinion makes it undesirable for the patient to participate in the study, or which would jeopardize compliance with the protocol, or would interfere with the results of the study;<br>7) Patients with a history of uncontrolled cardiovascular or interstitial lung disease and evidence or risk of retinal vein occlusion or central serous retinopathy (patients with a history of cardiovascular or interstitial lung disease and evidence or risk of retinal vein occlusion or central serous retinopathy (past or present evidence of rethinophaty central serous retinopathy - CSR -, occlusion of retinal - RVOo retinal degenerative disease) or ophthalmopathy, which according to the ophthalmologic evaluation at baseline could be considered a risk factor for CSR / RVO (eg. cupping of the optic disc, visual field defect, intraocular pressure - (eg: central IOP - > 21 mmHg);<br>8) Previous or concurrent malignancy. Exceptions: adequately treated basal cell or squamous cell skin cancer; in situ carcinoma of the cervix, treated curatively and without evidence of recurrence for at least 3 years prior to study entry; or other solid tumor treated curatively, and without evidence of recurrence for at least 3 years prior to study entry<br>9) History of Gilbert's syndrome;<br>10) Inability to regularly access centre facilities for logistical or other reasons;<br>11) History of poor co-operation, non-compliance with medical treatment, or unreliability;<br>12) Participation in any interventional drug or medical device study within 30 days prior to treatment start.<br>13) Positive test for human immunodeficiency virus (HIV), hepatitis B virus surface antigen (HBV sAg) or hepatitis C virus ribonucleic acid (HCV antibody) indicating acute or chronic infection;<br>14) Known history of testing positive for human immunodeficiency virus (HIV) or known acquired immunodeficiency syndrome (AIDS).<br>15) Receipt of live vaccine within 30 days prior to study drug administration.<br>16) History of severe or life-threatening skin adverse events or reactions to drugs.
**Study treatment duration, dose and schedule**	The following IMPs will be used in the study:<br>• **Arm A**: Combo Target (LGX818 450 mg p.o. od + MEK162. 45 mg p.o. bid) until Progression of Disease (PD); then Combo Immuno (nivolumab 1 mg/kg solution intravenously (i.v.) combined with ipilimumab 3 mg/kg solution i.v. every 3 weeks

	for 4 doses then nivolumab 3 mg/kg solution i.v. every 2 weeks or 240 mg every 2 weeks or 480 mg every 4 weeks) until PD.<br>• **Arm B**: Combo Immuno (nivolumab 1 mg/kg solution i.v. combined with ipilimumab 3 mg/kg solution i.v. every 3 weeks for 4 doses then nivolumab 3 mg/kg solution i.v. every 2 weeks or 240 mg every 2 weeks or 480 mg every 4 weeks) until PD; then Combo Target (LGX818 450 mg p.o. od + MEK162 45 mg p.o. bid) until PD.<br>• **Arm C**: Combo Target (LGX818 450 mg p.o. od + MEK162 45 mg p.o. bid) for 8 weeks followed by Combo Immuno (nivolumab 1 mg/kg solution i.v. combined with ipilimumab 3 mg/kg solution i.v. every 3 weeks for 4 doses then nivolumab 3 mg/kg solution i.v. every 2 weeks or 240 mg every 2 weeks or 480 mg every 4 weeks) until PD; then Combo Target (LGX818 450 mg p.o. od + MEK162 45 mg p.o. bid) until PD.<br>Shift from one combination therapy to the following one can be done (once the procedures of the new screening have been completed) in case of absence of AEs events from the previous combination therapy or in case of grade 1 AEs.<br>In case of grade ≥2 AEs from the previous combination therapy, shift to the following combination therapy can be done only when the AE is resolved or has decreased in intensity to at least grade 1.<br>If the AE does not resolve or decrease to at least grade 1, during the second screening period (28 days), patient should be followed up for additional 28 days. All screening procedures must be repeated before starting the new combination therapy (tumor assessment, to exclude progression disease, included). If AE does not resolve or decrease to al least grade 1, during this additional period (until 56 days after PD1), patient will be permanently discontinued.<br>Treatment with any of the treatment schemes will be continued until the development of progressive disease (as per Investigator assessment), unacceptable toxicity, consent withdrawal, death, reasons deemed by the treating physician or study termination by the Sponsor. To reduce the risk of long-term toxicity, no more than 2 years of ipilimumab/nivolumab dosing will be administered to study patients.<br>Patients in the ARM C, with a progression disease documented at the first tumor evaluation, will be discontinued from the study and will be treated as per institutional standard of care thereafter.<br>Patients who discontinue from the study will be asked to return to the clinic within 28 days of the last dose of IMP for the follow-up visit and to be contacted every 12 weeks until 60 months from randomization for long term survival follow-up.
**Primary endpoint**	OS is the primary endpoint of the study.
**Secondary endpoints**	• Total PFS;<br>• 3 years PFS rate;<br>• Percentage of patients alive at 3 years;<br>• Best overall response rate (BORR);<br>• Duration of response (DoR);<br>• Biological markers (biomarker study);<br>• Health-related quality of life (HRQoL;

	• General health status;<br>• Impairment of work productivity and activity.
**Safety endpoints**	• Toxicity of the IMPs (NCI CTC-AE Version 4.03 criteria);<br>• Adverse events (AEs) and serious adverse events (SAEs);<br>• Vital signs (weight, BMI, heart rate, blood pressure);<br>• Laboratory safety parameters (haematology, blood chemistry, urinalysis).
**Biomarker study endpoints**	<u>Tumor tissue biomarkers:</u><br>• Immune status: CD3, CD8, and CD4 T cells; Activated T cells; T regulatory cells; Dendritic cells;<br>• Resistance to immunotherapy agents: Checkpoint receptors/ligands; myeloid-derived suppressor cells (MDSCs);<br>• Resistance to targeted agents: aberrations in MEK/PI3K pathways, cytokines that interact with tyrosine kinase receptors (VEGF, HGF and their cognate receptors);<br>• Mutational load and neoantigen profile.<br><u>Peripheral blood biomarkers:</u><br>• Immune status/"Resistance" to immunotherapy agents: Activated T cells; Memory/Exhausted T cells; T regulatory cells; MDSCs; Inflammatory response; C-reactive protein (CRP); TCR Sequencing/Gene Expression analysis;<br>• Response/Resistance to targeted agents: Apoptotic tumor cells (as measured by circulating tumor DNA); soluble hepatocyte growth factor (sHGF); serum vascular endothelial growth factor (sVEGF), soluble interleukin-2 receptor (sCD25);<br>Approximately 80-90 patients will take part in the biomarkerstudy.
**Statistical methods**	<u>Sample size determination</u><br>This study is designed as a phase II, randomized trial with no formal comparative test. The sample size is discussed for the primary endpoint of the study (Overall Survival).<br>For each arm, a single-stage design will be used. We have assumed a median PFS of about 10 months for the combo target therapy (LGX818/MEK162) and a similar value for the combo immunotherapy (ipilimumab/nivolumab) derived from the aggregate clinical activity rate of 65% which, using an exponential distribution for PFS, could broadly give a median PFS of about 9.5 months. OS seems to be strictly correlated with total PFS.<br>The null hypothesis is a median OS time of 15 months (i.e. percentage of surviving patients of 33% at 24 months). The altervative hypothesis is a median OS time of 23 months (i.e. percentage of surviving of 48% at 24 months).<br>Using an exact 5% one-sided significance test at least 69 patients have to be randomized in each arm when the power of the study is 80%.<br>For each arm, the strategy will be further investigated if at least 30 patients, alive at 24 months, are observed. Taking in account a 10% drop-out rate, a total of 230 patients will be enrolled to ensure a minimum of 207 randomized patients.<br>If for all arms the null hypothesis will be rejected the best sequence will be identified considering secondary endpoints, mainly total PFS.

	Data handling<br>The Overall Survival (OS) will be calculated as the time from the date of randomization until the date of death for any cause. When survival information is not available at the time of data analysis, the patient will be censored using the last date where it was known the patient was alive.<br>Total Progression Free Survival (TPFS) will be calculated from the date of randomization to the date of the second progression (i.e. the progression to second treatment in Arm A and B and to third treatment for Arm C); any progression or death will be considered as an event if patient cannot complete treatment sequence.<br>Three years PFS rate; calculated from the date of randomization.<br>Duration of response (DoR) will be calculated as the time from the date of first documented response (CR or PR) until the date of the first documented progression or death due to underlying cancer.<br>If the patient with a CR o PR has no progression death due to underlying cancer, the patient will be censored at the date of last adeguate tumor assessment.<br>Statistical analysis<br>A comprehensive Statistical Analysis Plan (SAP) will be prepared before database lock. All enrolled patients in the study will be considered for the Screened Population.<br>All randomized will be considered the Intention-To-Treat population (ITT).<br>The subset of patient of the ITT population receiving at least one dose of the study medication will define the Safety Population (SP).<br>Analysis of efficacy endpoints will be performed in the ITT population whereas the safety analysis will be performed in the Safety Population.<br>No comparative tests between the three arms will be performed and results will be presented as descriptive statistics. The standard summary statistics will be used for both continuous and discrete variables. The objective response rate (ORR) and the percentage of patients alive at 3 years will be reported with its 95% confidence interval (CI).<br>The time-dependent endpoint will be analyzed according to the Kaplan-Meier method.<br>Median with 95% confidence intervals will be derived from the K-M curves and presenting time-dependent endpoints as K-M plot (with a 95% CI over time).<br>Cox's proportional hazard model will be used to assess the impact of known prognostic factors and treatment assigned.<br>The list of the covariates to be included in the Cox's model will be presented and clinically justified in the statistical analysis plan.<br><br>Safety and tolerability data will be presented by treatment received.<br>Appropriate summaries of these data will be presented. Safety and tolerability will be assessed in terms of AEs, laboratory data, ECG data, vital signs and weight, which will be collected for all patients. AEs (both in terms of MedDRA preferred terms and CTCAE grade), laboratory data, ECG data, vital signs data and weight will be listed individually by the patient and summarised by treatment received.
**Duration of the study**	Treatment duration: until second PD (2 years estimated).<br>Global Study Start (First Patient First Visit Date): November 2016.<br>Global Recruitment end (Last Patient First Visit): May 2019.

	Global Study end (Last Patient Last Visit): May 2024*.<br>*This date is dependent on the clinical course of the disease and may therefore occur earlier than indicated.

LIST OF ABBREVIATIONS

AEs	Adverse Events
AESIs	Adverse Events of Special Interest
ALT	Alanine Transaminase (SGPT)
ANC	Absolute Neutrophil Count
AST	Aspartate Transaminase (SGOT)
ATP	Adenosin Triphosphate
AUC	Area Under the plasma concentration-time Curve
BCRP	Breast Cancer Resistant Protein
bid	Bis in die (twice daily)
BMI	Body Mass Index
BORR	Best Overall Response Rate
BRAF	B-raf murine sarcoma vioral oncogene homolog B1
BUN	Blood Urea Nitrogen
C/A/P	Chest/Abdomen/Pelvis
CI	Confidence Interval
CK	Creatin-kinase
Cmax	Maximum plasma concentration
CRP	C-reactive Protein
CT	Computed Tomography
CTC-AE	Common Terminology Criteria for Adverse Events
CTLA-4	Cytotoxic T Lymphocyte Antigen 4
DEHP	Di-(2-ethylexil-phthalate)
DILI	Drug-induced Liver Injury
DoR	Duration of Response
ECG	Electrocardiogram
EchO	Echocardiogram
ECOG	Eastern Cooperative Oncology Group
eCRF	Electronic Case Report Form
EORTC QLQ-C30	30-item European Organisation for Research and Treatment of Care quality of life questionnaire
EQ-5D	European Quality of Life 5-Dimensions
EOS	End of Study Visit
ERK	Extracellular Signal-Regulated Kinase
FDA	Food and Drug Administration

FFPE	Formalin-fixed Paraffin-embedded
HFSR	Hand Foot Skin Reaction
HCG	Human Chorionic Gonadotropine
HGF	Hepatocyte Growth Factor
HRQoL	Health-related Quality of Life
ICF	Informed Consent Form
ICH	International Conference of Harmonization
IMP	Investigational Medicinal Product
INR	International Normalized Ratio
IRB/IEC	Institutional Review Board / Independent Ethics Committee
IV	Intravenous
KA	Keratoacanthoma
Kg	Kilogram
LDH	Lactate dehydrogenase
LFT	Liver Function Tests
LLN	Lower Limit of Normal
LVEF	Left Ventricle Ejection Fraction
MAPK	Mitogen Activated Protein Kinase
MedDRA	Medical Dictionary for Regulatory Activities
MDSCs	Myeloid-derived Suppressor Cells
MEK	Methyl Ethyl Ketone
mg	Milligram
ml	Millilitre
MRI	Magnetic Resonance Imaging
mRNA	Messenger Ribonucleic Acid
MTD	Maximum Tolerated Dose
MUGA	Multiple Gated Acquisition Scan
NCI	National Cancer Institute
od	Once Daily
ORR	Overall Response Rate
OS	Overall Survival
PBMC	Peripheral Blood Mononuclear Cells
PCN	Packaging Control Number
PD	Progression of Disease
PD-1	Programmed Death Receptor-1

PET	Positron Emission Tomography
PFS	Progression-Free Survival
PK	Pharmacokinetic
p.o.	Per os (oral route)
PS	Performance Status
PVC	Polyvinyl Chloride
RECIST	Response Evaluation Criteria In Solid Tumors
RL	Room Light
RT	Room Temperature
RVO	Retinal Vein Occlusion
SAE	Serious Adverse Events
SAP	Statistical Analysis Plan
SCC	Squamous Cell Carcinoma
sCD25	Soluble interleukin-2 receptor
SECOMBIT	Sequential Combo Immuno and target Therapy
sHGF	Soluble Hepatocyte Growth Factor
sICAM-1	Soluble Intercellular Adhesion Molecule-1
SUSAR	Suspected Unexpected Serious Adverse Reactions
sVEGF	Serum Vascular Growth factor
T3	Triiodotyroxine
T4	Tyroxine
TdP	Torsade de Points
TPFS	Total Progression-Free Survival
TSH	Thyroid Stimulating Hormone
ULN	Upper Limit of Normal
VAS	Visual Analog Scale
VEGF	Vascular Endothelial Growth Factor
WBC	White blood cell
WOCBP	WOCBP women of childbearing potential
WPAI:GH	Work Productivity and Activity Impairment: General Health

1. SCHEDULE OF ASSESSMENTS

ARM A

ARM A

Combo Target (LGX818 450 mg p.o. od + MEK162. 45 mg p.o. bid) until progression disease PD; then Combo Immuno (nivolumab 1 mg/kg solution intravenously (i.v.) combined with ipilimumab 3 mg/kg solution i.v. every 3 weeks for 4 doses then nivolumab 3 mg/kg solution i.v.every 2 weeks or 240 mg every 2 weeks or 480 mg every 4 weeks) until progression of disease (PD)

	Screening Phase	TREATMENT PHASE [2]													Follow up [4]	Long term follow up[4]
		Combo Target					Combo immuno							End of treatment [3]		
	Baseline1 [1]	LGX818 + MEK 162				Baseline2 [14]	Nivolumab + Ipilimumab				Nivolumab					
	BAS1	TR1	TR2	TR3	TRSub	BAS2	TR1	TR2	TR3	TR4	TR5[5]	TR6	TRSub	EoT	FU1	FU2
Day	-28 to 0	1	29	57	Q28D	0-28 D	1	22	43	64	85	99	Q14d		28d from EoT	Q12W
Informed Consent[6]	X															
Demographics	X															
Medical History	X															
Physical Examination & Vital signs[7]	X	X	X	X	X	X	X	X	X	X	X	X	X	X	X	
ECOG Performance Status	X	X	X	X	X	X	X	X	X	X	X	X	X	X	X	
Hematology	X	X[9]	X	X	X	X	X	X	X	X	X	X	X	X	X	
Biochemistry, Coagulation and Thyroid function test	X	X[9]	X	X	X	X	X	X	X	X	X	X	X	X	X	
HIV, HBV and HCV test	X															
Cardiac/Muscle Enzymes[23]	X	X	X	X	X	X	X	X	X	X	X	X	X	X	X	
Urinalysis	X	Only if clinically indicated														
Endocrine Panel	X					X	X	X	X	X	X	X	X	X		
BRAF Mutation testing[22]	X															
Pregnancy Test[10]	X	Q 4 W (±1)					Q 6 W (±1)								X	X
Tumor Assessments (CT/MRI)[11]	X	Every 8 weeks (+/- 1 week) for the first year, every 12 weeks (+/- 1 week) thereafter while the patient is on study													X	X
Quality of life and general Health Status [24]	X	X	X	X	X	X	X	X	X	X	X	X	X	X	X	
Biomarkers study: Biopsy[12]	X*		X (4W)			X*	X (4W)						X (PD2)			
Biomarkers study: Blood Drawn[13]	X*		X (4W)			X*	X (4W)						X (PD2)			
Ophthalmologic exam[15]	X					X										
Dermatologic evaluation[16]	X	Q8W				X	Q8W									
ECG[8]	X		X (1month)	Q12W		X	X (1 month)			Q12W						
Echocardiogram or MUGA[17]	X		X (1month)	Q12W		X	X (1 month)			Q12W						
AEs/SAEs[18]	X	X	X	X	X	X	X	X	X	X	X	X	X	X	X	X
Concomitant medication[19]	X	X	X	X	X	X	X	X	X	X	X	X	X	X	X	X
Combo target dispensing & accountability[20]		X	X	X	X											
Combo immuno administration[21]							X	X	X	X	X	X	X			

Notes:
Day 1 = first dose of IMP - D= days - W= weeks - Q=every - * Mandatory

**ARM A**<br>Combo Target (LGX818 450 mg p.o. od + MEK162. 45 mg p.o. bid) until progression disease PD; then Combo Immuno (nivolumab 1 mg/kg solution intravenously (i.v.) combined with ipilimumab 3 mg/kg solution i.v. every 3 weeks for 4 doses then nivolumab 3 mg/kg solution i.v.every 2 weeks or 240 mg every 2 weeks or 480 mg every 4 weeks) until progression of disease (PD)
1. All screening/baseline assessments must be performed -28 to 0 days prior to the first administration of the IMP on Day 1 with the exception of the serum pregnancy test to be done within 24 hours. Results of tests or examinations (including tumor assessments) performed before obtaining informed consent and within the 28 Days prior to Day 1 may be used.<br>2. A window of 3 days prior to the scheduled visit date and 3 days after the scheduled visit date (- 3 days / + 3 days) is allowed for each visit, except for tumor evaluations for which a window of +/- 7 days will apply and for Combo Immuno visits for which a window of +/- 2 days will apply.<br>3. End of Treatment Visit (EoT) will be performed when the patient permanently discontinues all study treatments regardless of when it occurs.<br>4. Follow up visit is to be performed within 28 days from discontinuation of treatments and thereafter every 12 weeks until 60 months from randomization for long term survival follow-up.<br>5. Between treatment 4 and 5 in the nivolumab/Ipilimumab treatment 21 day should passed before starting a q14 schedule assesssment<br>6. Informed consent must be obtained prior to perform any study procedure<br>7. Height is taken at screening only. For patients with palpable/superficial lesions, clinical disease assessments by physical examination should be performed at baseline and throughout study treatment as clinically indicated. Color photographs with ruler/calipers will be taken at baseline and at all subsequent tumor assessment time points.<br>8. A 12-lead ECG will be performed at screening/baseline, at the 2nd baseline, at the 1st month (+/- 1 week) after first dose of each combination treatment and every 12 weeks (+/- 1 week) thereafter during treatment period.<br>9. Hematology and biochemistry assessments do not need to be repeated on Day 1 if performed within 7 days; if it is necessary to repeat these blood tests, the results must be known before the patient receives treatments to ensure inclusion/exclusion criteria related to these tests are met. Test for HIV infection is mandatory at screening.<br>10. Serum pregnancy test to be performed within 24 hours prior to Day 1. A serum/urine pregnancy test to be performed every 6 (± 1) weeks during the treatment Combo-Immuno and every 4 (± 1) weeks during the treatment Combo-Target and at end of treatment and follow-up visit up to 31 weeks after the last dose of nivolumab/ipilimumab<br>11. Tumor assessments including measurable and non-measurable lesions (brain CT or MRI at all baselines, CT/MRI C/A/P, CT/MRI of brain as clinically indicated during treatment period, bone scan if clinically indicated). all measurable and non-measurable lesions must be documented at screening (within 28 days prior to randomization) and re-assessed at each subsequent tumor evaluation (every 8 weeks (+/- 1 week) for the first year, every 12 weeks (+/- 1 week) while the patient is on study). Tumor assessments with CT or MRI scans of the brain, chest, abdomen, and pelvis will be performed until disease progression after the second combo treatment per RECIST v1.1. Imaging of the neck should be included if clinically indicated. In the event PET/CT scanner is used for tumor assessments, the CT portion of the PET/CT must meet criteria for diagnostic quality. All scans can be collected for a possible independent review.<br>12. Biopsy of tumoral lesions for the biological study will be performed at baseline 1, Week 4 and disease progression 1 (DP 1) during Combo Immuno therapy; and at Week 4 and disease progression 2 (DP 2) during Combo Target therapy. Biopsy of tumoral lesions are mandatory at baseline 1 and at the first disease progression (PD1), and are optional at the other time-points.<br>13. Serum, plasma (for circulating tumor DNA) and whole blood for PBMC processing will be taken at baseline 1, Week 4 and disease progression 1 (DP 1) during Combo Immuno therapy; and at Week 4 and disease progression 2 (DP 2) during Combo Target therapy.<br>14. The second baseline visit will be performed -28 to 0 days prior to the first administration of the Combo Immuno treatment on Day 1. 14 days minimum for wash out is required. Shift from the combination therapy to the following one can be done (once the procedures of the new screening have been completed) in case of absence of AEs events from the prevsious combination therapy or in case of grade 1 AEs. In case of grade ≥ 2 AEs from the previous combination therapy, shift to the following combination therapy can be done only when the AE is resolved or has decreased in intensity to at least grade 1. If the AE does not resolve or decrease to at least grade 1, during the second screening period (28 days), patient should be followed up for additional 28 days. All screening procedures must be repeated before starting the new combination therapy (tumor assessment, to exclude progression disease, included). If AE does not resolve or decrease to al least grade 1, during this additional period (until 56 days after PD1), patient will be permanently discontinued.<br>15. An ophthalmologic examination will be performed at the screening/baseline visit, at the 2nd baseline visit and then when clinically indicated.<br>16. A dermatologic evaluation will be performed at the screening/baseline visit and every 8 weeks while the patient is on study.<br>17. An echocardiogram or MUGA will be performed at screening/baseline, at the 2nd baseline, at the 1st month and every 12 weeks while the patient is on study<br>18. From the ICF signature until the end of the study all AEs must be recorded in the eCRF. SAE, and Pregnancy report need to be reported also with the SAE Report Form within 24 hours to the PV Office After the last treatment, any new, non-serious AEs which the Investigator considers may be related to treatments should be reported according Section 7 – Safety instruction and guidance<br>19. All concomitant medications during the study started within 14 Days prior to the screening visit and up to the end of treatment visit must be recorded.<br>20. Combo Target (LGX818 450 mg p.o. od + MEK162 45 mg p.o. bid) will be administered until PD. Patients will keep a diary to record ONLY those occasions when a Combo Target dose was missed. The patient will bring this diary with him/her to each study visit to allow missed doses to be recorded by the Investigator.<br>21. Combo Immuno (nivolumab 1 mg/kg solution i.v. combined with ipilimumab 3 mg/kg solution i.v. every 3 weeks for 4 doses then nivolumab 3 mg/kg solution i.v. every 2 weeks or 240 mg every 2 weeks or 480 mg every 4 weeks) will be administered until PD following the Combo Target and subsequent PD. Please note: subjects may be dosed no less than 12 days from the previous dose.<br>22. Presence of BRAF V600E or V600K mutation in tumor tissue prior to enrolment.<br>23. Troponin, Creatine Kinase (CK). If total CK ≥3 X ULN, then measure isoenzymes and myoglobin in blood or urine weekly.<br>24. Assessment of HRQoL (EORTC QLQ-C30), general health status (EQ-5D) and impairment of work productivity and activity (WPAI:GH) will be performed every 28 days during the Combo Target therapy and every 21 days at administration of nivolimumab+ipilimumab (i.e. every 21 days for 4 doses) and then every two administrations (i.e. every 28 days) of nivolimumab during the Combo Immuno therapy

Table 1: Schedule of Assessments and Procedures in Arm A

ARM B

ARM B

Combo Immuno (nivolumab 1 mg/kg solution i.v.combined with ipilimumab 3 mg/kg solution i.v.every 3 weeks for 4 doses then nivolumab 3 mg/kg solution i.v.every 2 weeks or 240 mg every 2 weeks or 480 mg every 4 weeks) until PD; then Combo Target (LGX818 450 mg p.o. od + MEK162 45 mg p.o. bid) until PD.

	Screening Phase	**TREATMENT PHASE** [2]													Follow up [4]	Long term follow up[4]
		Combo immuno								Combo Target				End of Treat ment [3]		
	Baseline1[1]	Nivolumab + Ipilimumab				Nivolumab			Baseline2 [14]	LGX818 + MEK 162						
	BAS1	TR1	TR2	TR3	TR4	TR5[5]	TR6	TRSub	BAS2	TR1	TR2	TR3	TRSub	EoT	FU1	FU2
Day	-28 to 0	1	22	43	64	85	99	Q14d	0-28 D	1	29	57	Q28D		28d from EoT	Q12W
Informed Consent[6]	X															
Demographics	X															
Medical History	X															
Physical Examination & Vital signs[7]	X	X	X	X	X	X	X	X	X	X	X	X	X	X	X	
ECOG Performance Status	X	X	X	X	X	X	X	X	X	X	X	X	X	X	X	
Hematology	X	X[9]	X	X	X	X	X	X	X	X	X	X	X	X	X	
Biochemistry Coagulation and Thyroid function test	X	X[9]	X	X	X	X	X	X	X	X	X	X	X	X	X	
HIV, HBV and HCV test	X															
Cardiac/Muscle Enzymes[23]	X	X	X	X	X	X	X	X	X	X	X	X	X	X	X	
Urinalysis	X	Only if clinically indicated														
Endocrine Panel	X	X	X	X	X	X	X	X	X							
BRAF Mutation testing[22]	X															
Pregnancy Test[10]	X	Q 6 W (±1)							Q 4 W (±1)						X	X
Tumor Assessments (CT/MRI)[11]	X	Every 8 weeks (+/- 1 week) for the first year, every 12 weeks (+/- 1 week) thereafter while the patient is on study													X	X
Quality of life and general Health Status [24]	X	X	X	X	X	X	X	X	X	X	X	X	X	X	X	
Biomarkers study: Biopsy[12]	X*	X (4W)							X*		X (4W)		X (PD2)			
Biomarkers study: Blood Drawn[13]	X*	X (4W)							X*		X (4W)		X (PD2)			
Ophthalmologic exam[15]	X								X							
Dermatologic evaluation[16]	X	Q8W							X	Q8W						
ECG[8]	X	X (1month)			Q 12 W				X		X (1 month)	Q12W				
Echocardiogram or MUGA[17]	X	X (1month)			Q 12 W				X		X (1 month)	Q12W				
AEs/SAEs[18]	X	X	X	X	X	X	X	X	X	X	X	X	X	X	X	X
Concomitant medication[19]	X	X	X	X	X	X	X	X	X	X	X	X	X	X	X	X
Combo target dispensing & accountability[20]	X									X	X	X	X			
Combo immuno administration[21]	X	X	X	X	X	X	X	X								

Notes:
Day 1 = first dose of IMP - D= days - W= weeks - Q=every - * Mandatory

ARM B<br>Combo Immuno (nivolumab 1 mg/kg solution i.v.combined with ipilimumab 3 mg/kg solution i.v.every 3 weeks for 4 doses then nivolumab 3 mg/kg solution i.v.every 2 weeks or 240 mg every 2 weeks or 480 mg every 4 weeks) until PD; then Combo Target (LGX818 450 mg p.o. od + MEK162 45 mg p.o. bid) until PD.
1. All screening/baseline assessments must be performed -28 to 0 days prior to the first administration of the IMP on Day 1 with the exception of the serum pregnancy test to be done within 24 hours. Results of tests or examinations (including tumor assessments) performed before obtaining informed consent and within the 28 Days prior to Day 1 may be used.<br>2. A window of 3 days prior to the scheduled visit date and 3 days after the scheduled visit date (- 3 days / + 3 days) is allowed for each visit, except for tumor evaluations for which a window of +/- 7 days will apply and for Combo Immuno visits for which a window of +/- 2 days will apply.<br>3. End of Treatment Visit (EOS) will be performed when the patient permanently discontinues all study treatments regardless of when it occurs.<br>4. Follow up visit is to be performed within 28 days from discontinuation of treatments and thereafter every 12 weeks until 60 months from randomization for long term survival follow-up.<br>5. Between treatment 4 and 5 in the nivolumab/Ipilimumab treatment 21 day should passed before starting a q14 schedule assesssment<br>6. Informed consent must be obtained prior to perform any study procedure including screening/baseline assessments.<br>7. Height is taken at screening only. For patients with palpable/superficial lesions, clinical disease assessments by physical examination should be performed at baseline and throughout study treatment as clinically indicated. Color photographs with ruler/calipers will be taken at baseline and at all subsequent tumor assessment time points.<br>8. A 12-lead ECG will be performed at screening/baseline, at the 2nd baseline, at the 1st month (+/- 1 week) after first dose of each combination treatment and every 12 weeks (+/- 1 week) thereafter during treatment period<br>9. Hematology and biochemistry assessments do not need to be repeated on Day 1 if performed within 7 days; if it is necessary to repeat these blood tests, the results must be known before the patient receives treatments to ensure inclusion/exclusion criteria related to these tests are met. Test for HIV infection is mandatory at screening.<br>10. Serum pregnancy test to be performed within 24 hours prior to Day 1. A serum/urine pregnancy test to be performed every 6 (± 1) weeks during the treatment Combo-Immuno and every 4 Q 6 W (± 1) weeks during the treatment Combo-Target and at end of treatment and follow-up visit up to 31 weeks after the last dose of nivolumab/ipilimumaband at end of treatment and follow-up visit up to 31 weeks after the last dose of nivolumab/ipilimumab<br>11. Tumor assessments including measurable and non-measurable lesions (brain CT or MRI at all baselines, CT/MRI C/A/P, CT/MRI of brain as clinically indicated during treatment period, bone scan if clinically indicated); all measurable and non-measurable lesions must be documented at screening (within 28 days prior to randomization) and re-assessed at each subsequent tumor evaluation (every 8 weeks (+/- 1 week) for the first year, every 12 weeks (+/- 1 week) while the patient is on study). Tumor assessments with CT or MRI scans of the brain, chest, abdomen, and pelvis will be performed until disease progression after the second combo treatment per RECIST v1.1. Imaging of the neck should be included if clinically indicated. In the event PET/CT scanner is used for tumor assessments, the CT portion of the PET/CT must meet criteria for diagnostic quality. All scans can be collected for a possible independent review.<br>12. Biopsy of tumoral lesions for the biological study will be performed at baseline 1, Week 4 and disease progression 1 (DP 1) during Combo Immuno therapy; and at Week 4 and disease progression 2 (DP 2) during Combo Target therapy. Biopsy of tumoral lesions are mandatory at baseline 1 and at the first disease progression (PD1), and are optional at the other time-points.<br>13. Serum, plasma (for circulating tumor DNA) and whole blood for PBMC processing will be taken at baseline 1, Week 4 and disease progression 1 (DP 1) during Combo Immuno therapy; and at Week 4 and disease progression 2 (DP 2) during Combo Target therapy.<br>14. The second baseline visit will be performed -28 to 0 days prior to the first administration of the Combo Target treatment on Day 1 14 days minimum for wash out is required. Shift from the combination therapy to the following one can be done (once the procedures of the new screening have been completed) in case of absence of AEs events from the prevsious combination therapy or in case of grade 1 AEs. In case of grade ≥ 2 AEs from the previous combination therapy, shift to the following combination therapy can be done only when the AE is resolved or has decreased in intensity to at least grade 1. If the AE does not resolve or decrease to at least grade 1, during the second screening period (28 days), patient should be followed up for additional 28 days. All screening procedures must be repeated before starting the new combination therapy (tumor assessment, to exclude progression disease, included). If AE does not resolve or decrease to al least grade 1, during this additional period (until 56 days after PD1), patient will be permanently discontinued.<br>15. An ophthalmologic examination will be performed at the screening/baseline visit, at the 2nd baseline visit and then when clinically indicated.<br>16. A dermatologic evaluation will be performed at the screening/baseline visit and every 8 weeks while the patient is on study.<br>17. An echocardiogram or MUGA will be performed at screening/baseline, at the 2nd baseline, at the 1st month and every 12 weeks while the patient is on study<br>18. From the ICF signature until the end of the study all AEs must be recorded in the eCRF. SAE and Pregnancy report need to be reported also with the SAE Report Form within 24 hours to the PV Office After the last treatment, any new, non-serious AEs which the Investigator considers may be related to treatments should be reported according Section 7 – Safety instruction and guidance<br>19. All concomitant medications during the study started within 14 Days prior to the screening visit and up to the end of treatment visit must be recorded.<br>20. Combo Target (LGX818 450 mg p.o. od + MEK162 45 mg p.o. bid) will be administered until second progression disease PD observed after Combo Immuno treatment. Patients will keep a diary to record ONLY those occasions when a Combo Target dose was missed. The patient will bring this diary with him/her to each study visit to allow missed doses to be recorded by the Investigator.<br>21. Combo Immuno (nivolumab 1 mg/kg solution i.v. combined with ipilimumab 3 mg/kg solution i.v. every 3 weeks for 4 doses then nivolumab 3 mg/kg solution i.v. every 2 weeks or 240 mg every 2 weeks or 480 mg every 4 weeks) will be administered until PD. Please note: subjects may be dosed no less than 12 days from the previous dose.<br>22. Presence of BRAF V600E or V600K mutation in tumor tissue prior to enrolment.<br>23. Troponin, Creatine Kinase (CK). If total CK ≥3 X ULN, then measure isoenzymes and myoglobin in blood or urine weekly.<br>24. Assessment of HRQoL (EORTC QLQ-C30), general health status (EQ-5D) and impairment of work productivity and activity (WPAI:GH) will be performed every 28 days during the Combo Target therapy and every 21 days at administration of nivolimumab+ipilimumab (i.e. every 21 days for 4 doses) and then every two administrations (i.e. every 28 days) of nivolimumab during the Combo Immuno therapy

Table 2: Schedule of Assessments and Procedures in Arm B

ARM C

ARM C

Combo Target (LGX818 450 mg p.o. od + MEK162 45 mg p.o. bid) for 8 weeks followed by Combo Immuno (nivolumab 1 mg/kg solution i.v. combined with ipilimumab 3 mg/kg solution i.v. every 3 weeks for 4 doses then nivolumab 3 mg/kg solution i.v. every 2 weeks or 240 mg every 2 weeks or 480 mg every 4 weeks) until PD; then Combo Target (LGX818 450 mg p.o. od + MEK162 45 mg p.o. bid) until PD.

	Screening Phase	TREATMENT PHASE [2]																Follow up [4]	Long term follow up[4]
	Baseline1[1]	Combo Target LGX818 + MEK 162		Baseline 2[13]	Combo Immuno Nivolumab + Ipilimumab				Nivolumab			Baseline 3[14]	Combo Target LGX818 + MEK 162				End of treatment[3]		
	BAS1	TR1	TR2	BAS2	TR3	TR4	TR5	TR6	TR7 [5]	TR8	TRSub	BAS3	TR1	TR2	TR3	TRSub	EoT	FU1	FU2
**Day**	-28 to 0	1	29	0 – 28 days	1	22	43	64	85	99	Q14dup to PD	0 – 28 days	1	29	57	Q28d up to PD		28d from EoT	Q12W
Informed Consent[6]	X																		
Demographics	X																		
Medical History	X																		
Physical Examination & Vital Signs[7]	X	X	X	X	X	X	X	X	X	X	X	X	X	X	X	X	X	X	
ECOG Performance Status	X	X	X	X	X	X	X	X	X	X	X	X	X	X	X	X	X	X	
Hematology	X	X[9]	X	X	X	X	X	X	X	X	X	X	X	X	X	X	X	X	
Biochemistry Coagulation and Thyroid function test	X	X[9]	X	X	X	X	X	X	X	X	X	X	X	X	X	X	X	X	
HIV, HBV and HCV test	X																		
Cardiac/Muscle Enzymes[24]	X	X	X	X	X	X	X	X	X	X	X	X	X	X	X	X	X	X	
Urinalysis	X	Only if clinically indicated																	
Endocrine Panel	X			X	X	X	X	X	X	X	X	X							
BRAF Mutation testing[23]	X																		
Pregnancy Test[8]	X	Q 4 W (± 1)		X	Q 6 W (± 1)							X	Q 4 W (± 1)					X	X
Tumor Assessments (CT/MRI)[11]		Every 8 weeks (+/- 1 week) for the first year, every 12 weeks (+/- 1 week) thereafter while the patient is on study																X	X
Quality of life and general Health Status [25]	X	X	X	X	X	X	X	X	X	X	X	X	X	X	X	X	X	X	X
Biomarkers study: Biopsy[12]	X*			X	X (4W)							X*		X		X (PD)			
Biomarkers study: Blood Drawn[13]	X*			X*	X (4W)							X*		X		X (PD)			
Ophthalmologic exam[16]	X			X								X							
Dermatologic evaluation[17]	X		X	X	Q 6 W							X		X		Q4			
Echocardiogram or MUGA[18]	X		X (1month)	X	X (1month)		Q12W					X		X (1month)		Q12W			
ECG[8]	X		X (1month)	X	X (1month)		Q12W					X		X (1month)		Q12W			
AEs/SAEs[19]	X	X	X	X	X	X	X	X	X	X	X	X	X	X	X	X	X	X	X
Concomitant medication[20]	X	X	X	X	X	X	X	X	X	X	X	X	X	X	X	X	X	X	X
Combo target dispensing & accountability[21]	X	X	X									X	X	X	X	X			
Combo immuno administration[22]					X	X	X	X	X	X	X								

Notes:
Day 1 = first dose of IMP - D= days - W= weeks - Q=every - *Mandatory

ARM C

Combo Target (LGX818 450 mg p.o. od + MEK162 45 mg p.o. bid) for 8 weeks followed by Combo Immuno (nivolumab 1 mg/kg solution i.v. combined with ipilimumab 3 mg/kg solution i.v. every 3 weeks for 4 doses then nivolumab 3 mg/kg solution i.v. every 2 weeks or 240 mg every 2 weeks or 480 mg every 4 weeks) until PD; then Combo Target (LGX818 450 mg p.o. od + MEK162 45 mg p.o. bid) until PD.

1. All screening/baseline assessments must be performed -28 to 0 days prior to the first administration of the IMP on Day 1 with the exception of the serum pregnancy test to be done within 24 hours. Results of tests or examinations (including tumor assessments) performed before obtaining informed consent and within the 28 Days prior to Day 1 may be used.
2. A window of 3 days prior to the scheduled visit date and 3 days after the scheduled visit date (- 3 days / + 3 days) is allowed for each visit, except for tumor evaluations for which a window of +/- 7 days will apply and for Combo Immuno visits for which a window of +/- 2 days will apply.
3. End of Study Visit (EOS) will be performed when the patient permanently discontinues all study treatments regardless of when it occurs.
4. Follow up visit is to be performed within 28 days from discontinuation of treatments and thereafter every 12 weeks until 60 months from randomization for long term survival follow-up.
5. Between treatment 4 and 5 in the nivolumab/Ipilimumab treatment 21 day should passed before starting a q14 schedule assesssment
6. Informed consent must be obtained prior to perform any study procedure including screening/baseline assessments.
7. Height is taken at screening only. For patients with palpable/superficial lesions, clinical disease assessments by physical examination should be performed at baseline and throughout study treatment as clinically indicated. Color photographs with ruler/calipers will be taken at baseline and at all subsequent tumor assessment time points.
8. A 12-lead ECG will be performed at screening/baseline, at the 2nd baseline, at the 1st month and every 12 weeks while the patient is on study
9. Hematology and biochemistry assessments do not need to be repeated on Day 1 if performed within 7 days; if it is necessary to repeat these blood tests, the results must be known before the patient receives treatments to ensure inclusion/exclusion criteria related to these tests are met. Test for HIV infection is mandatory at screening.
10. Serum pregnancy test to be performed within 24 hours prior to Day 1. A serum/urine pregnancy test to be performed every 6 (± 1) weeks during the treatment Combo-Immuno and every 4 (± 1) weeks during the treatment Combo-Target and at end of treatment and follow-up visit up to 31 weeks after the last dose of nivolumab/ipilimumab
11. Tumor assessments including measurable and non-measurable lesions (brain CT or MRI at all baselines, CT/MRI C/A/P, CT/MRI of brain as clinically indicated during treatment period, bone scan if clinically indicated); all measurable and non-measurable lesions must be documented at screening (within 28 days prior to randomization) and re-assessed at each subsequent tumor evaluation (every 8 weeks (+/- 1 week) for the first year, every 12 weeks (+/- 1 week) while the patient is on study). Tumor assessments with CT or MRI scans of the brain chest, abdomen, and pelvis will be performed until disease progression after the second combo treatment per RECIST v1.1. Imaging of the neck should be included if clinically indicated. In the event PET/CT scanner is used for tumor assessments, the CT portion of the PET/CT must meet criteria for diagnostic quality. All scans can be collected for a possible independent review.
12. Biopsy of tumoral lesions for the biological study will be performed at baseline 1, Week 4 and disease progression 1 (DP 1) during Combo Immuno therapy; and at Week 4 and disease progression 2 (DP 2) during the 2nd Combo Target therapy. Biopsy of tumoral lesions are mandatory at baseline 1 and at the first disease progression (PD1), and are optional at the other time-points.
13. Serum, plasma (for circulating tumor DNA) and whole blood for PBMC processing will be taken at baseline 1, at baseline 2, Week 4 and disease progression 1 (DP 1) during Combo Immuno therapy; and at baseline 3, Week 4 and disease progression 2 (DP 2) during the 2nd Combo Target therapy.
14. The second baseline visit will be performed -28 to 0 days prior to the first administration of the Combo Immuno treatment on Day 1. 14 days minimum for wash out is required. Shift from the combination therapy to the following one can be done (once the procedures of the new screening have been completed) in case of absence of AEs events from the prevsious combination therapy or in case of grade 1 AEs. In case of grade $\geq$ 2 AEs from the previous combination therapy, shift to the following combination therapy can be done only when the AE is resolved or has decreased in intensity to at least grade 1. If the AE does not resolve or decrease to at least grade 1, during the second screening period (28 days), patient should be followed up for additional 28 days. All screening procedures must be repeated before starting the new combination therapy (tumor assessment, to exclude progression disease, included). If AE does not resolve or decrease to al least grade 1, during this additional period (until 56 days after PD1), patient will be permanently discontinued. Patients in the ARM C, with a progression disease documented at the first tumor evaluation, will be discontinued from the study and will be treated as per institutional standard of care thereafter. Patients who discontinue from the study will be asked to return to the clinic within 28 days of the last dose of IMP for the follow-up visit and to be contacted every 12 weeks until 60 months from randomization for long term survival follow-up.
15. The third baseline visit will be performed -28 to 0 days prior to the first administration of the second Combo Target treatment on Day 1. 14 days minimum for wash out is required See rules for switching as above.
16. An ophthalmologic examination will be performed at the screening/baseline visit, at the 2nd baseline visit, at the 3rd baseline visit and then when clinically indicated.
17. A dermatologic evaluation will be performed at the screening/baseline visit and every 8 weeks while the patient is on study.
18. An echocardiogram or MUGA will be performed at screening/baseline, at the 2nd and 3rd baseline, at the 1st month (+/- 1 week) after first dose of each combination treatment and every 12 weeks (+/- 1 week) thereafter during treatment period.
19. From the ICF signature until the end of the study all AEs must be recorded in the eCRF. SAE and Pregnancy report need to be reported also with the SAE Report Form within 24 hours to the PV Office After the last treatment, any new, non-serious AEs which the Investigator considers may be related to treatments should be reported according Section 7 – Safety instruction and guidance
20. All concomitant medications during the study started within 14 Days prior to the screening visit and up to the end of treatment visit must be recorded.
21. Combo Target (LGX818 450 mg p.o. od + MEK162. 45 mg p.o bid) will be administered for initial 8 weeks and then will be administered until PD following the Combo Immuno and subsequent PD. Patients will keep a diary to record ONLY those occasions when a Combo Target dose was missed. The patient will bring this diary with him/her to each study visit to allow missed doses to be recorded by the Investigator.
22. Combo Immuno (nivolumab 1 mg/kg solution i.v. combined with ipilimumab 3 mg/kg solution i.v. every 3 weeks for 4 doses then nivolumab 3 mg/kg solution i.v. every 2 weeks or 240 mg every 2 weeks or 480 mg every 4 weeks) will be administered until PD following the first Combo Target. Please note: subjects may be dosed no less than 12 days from the previous dose.
23. Presence of BRAF V600E or V600K mutation in tumor tissue prior to enrollment
24. Troponin, Creatine Kinase (CK). If total CK $\geq$3 X ULN, then measure isoenzymes and myoglobin in blood or urine weekly.
25. Assessment of HRQoL (EORTC QLQ-C30), general health status (EQ-5D) and impairment of work productivity and activity (WPAI:GH) will be performed every 28 days during the Combo Target therapy and every 21 days at administration of nivolimumab+ipilimumab (i.e. every 21 days for 4 doses) and then every two administrations (i.e. every 28 days) of nivolimumab during the Combo Immuno therapy

Table 3: Schedule of Assessments and Procedures in Arm C

2. BACKGROUND AND RATIONALE

2.1 Overview of disease epidemiology and current treatment

2.1.1 Epidemiology of melanoma

Melanoma is the most serious form of skin cancer and strikes adults of all ages. Both incidence of melanoma and mortality rate are rapidly increasing throughout the world, constituting a significant and growing health burden (*Ferlay et al, 2010*). The worldwide incidence of melanoma in 2010 was estimated at 208,251 (*Jemal et al*, *2011*), and approximately 76,250 men and women were diagnosed with melanoma in 2012 in the United States alone (*American Cancer Society, 2012*)

About 80% of melanomas are detected in a localized stage and can be treated with surgical resection. When detected early, the 5-year survival rate of melanoma is above 90%; however, when melanoma is diagnosed after distant metastasis, the prognosis is by contrast very poor.

The 5-year survival rate decreases to 15% with a median survival between 8 and 9 months (Jemal et al, 2011). Advanced melanoma is one of the most aggressive human malignancies. In 2012, an estimated 9,000 melanoma patients in the US have died from their disease (*American Cancer Society, 2012*).

2.1.2 Treatment options in patients with advanced melanoma

For the last 40 years, treatment progresses in advanced melanoma have been largely stagnant, with traditional options, such as chemotherapy, lacking substantial efficacy. This has changed in 2011 with the Food and Drug Administration (FDA) approval of novel immunotherapy, ipilimumab (Yervoy®, Bristol-Myers Squibb), an antibody against the cytotoxic T-lymphocyte antigen-4 (anti-CTLA-4), although melanoma that has spread to distant sites remains rarely curable.

The discovery of the genetic underpinnings of melanoma and their characterization has uncovered potential targets for therapy, B-raf murine sarcoma viral oncogene homolog B1 (BRAF) mutations being principal among them. BRAF mutations have been identified as an important target in melanoma and discovery of oncogenic BRAF mutations highlighted the significant role of BRAF kinase in signalling pathways that control cellular proliferation. More than half of patients with metastatic melanoma have mutations that keep the BRAF protein constantly activated.

Mutations in BRAF exon 15 account for over 95% of activating BRAF mutations in metastatic melanoma. The two most common BRAF V600 mutations V600E and V600K have been reported to account for anywhere

between 66-91% and 7-30% of BRAF V600 mutant metastatic melanoma patients respectively. Both BRAF V600E and V600K mutant patients account for 92-98% of all BRAF V600 mutant metastatic melanoma patients (*Colombino et al, 2012*; *Jakob et al, 2011*; *Greaves et al, 2013*). These mutations constitutively activate BRAF and downstream signal transduction in the RAF/MEK/ERK pathway, which signals for cancer cell proliferation and survival. Moreover, oncogenic BRAF mutations generally correlate with poor prognosis in a variety of different tumor types.

The selective BRAF inhibitor vemurafenib was approved by the FDA (17 August 2011) for the treatment of patients with unresectable or metastatic melanoma with the BRAF V600E mutation as detected by an FDA approved test. In February 2012, vemurafenib received approval in the European Union (EU) as a monotherapy for the treatment of adult patients with BRAF V600 mutation-positive unresectable or metastatic melanoma.

Although studies have shown that BRAF-targeted therapy is effective in BRAF mutant melanoma, data also indicate that the duration of response is often short lived, with resistance developing quickly, within approximately 6 months (*Flaherty et al, 2010*; *Sosman et al, 2012*; *Chapman et al, 2011*; *Hauschild et al, 2012*). Re-activation of mitogen activated protein kinase (MAPK) signalling occurs in the majority of cases of acquired resistance to BRAF inhibitors. In an attempt to delay resistance to BRAF inhibition, the combination of a selective BRAF- and a MEK1/2-inhibitor is currently being investigated by several clinical trials in patients with advanced BRAF mutant melanoma. Results of the first such phase I/II study [NCT01072175] of the combination of investigational agents dabrafenib and trametinib, and of the phase Ib dose escalation study [NCT01271803] of vemurafenib (Zelboraf®, Roche), in combination with an investigational MEK inhibitor GDC-0973 (Roche), have been recently reported (*Flaherty et al, 2012*). Median PFS was 9.4 months in the dabrafenib plus trametinib group vs. 5.8 months in the dabrafenib group (*Flaherty et al, 2012*).
Despite recent treatment breakthroughs, advanced melanoma remains an aggressive disease with a poor prognosis. There is a need to develop new treatment regimens to improve the duration of response and delay the emergence of resistance, expanding the therapeutic options for patients with unresectable or metastatic melanoma, including those with BRAF V600 mutation.

2.2 Introduction to investigational treatments and other study treatment(s)

2.2.1 LGX818

LGX818 (encorafenib) is a highly selective ATP-competitive small molecule RAF kinase inhibitor, which suppresses the RAF/MEK/ERK pathway in tumor cells expressing BRAF V600. The narrow kinase profile and potent anti-proliferative activity of LGX818 translates into a very wide therapeutic index in vivo.

LGX818 was evaluated in rats and Cynomolgus monkeys in toxicology studies ranging from 1 to 4 weeks in duration. Overall, LGX818 was well tolerated at doses at which tumor regression was observed. Significant toxicities were mainly observed in the female rat at the highest dose of 400 mg/kg/day, a dose well above the MTD. Other findings included hyperplasia and hyperkeratosis in the skin (plantar surface of feet) and non-glandular stomach in rat, which was apparent at all dose levels and presented with recovery 4 weeks after stopping treatment, and an absence of the later stages of spermatid maturation in the male rats. Preclinical cardiovascular safety pharmacology data did not indicate a clinical risk for QTc prolongation based on the findings of the hERG assay and ECG evaluation in the GLP 4-week monkey study. Also, there were no clinical signs in the 4-week GLP rat and monkey studies that would indicate an effect on the central nervous system or respiratory system. No teratogenicity studies have been completed to date. For further details on non-clinical pharmacology and toxicology, please refer to the current LGX818 Investigator's Brochure.

The results of a phase I study assessing the maximum tolerated dose (MTD) and of the recommended dose phase II study of LGX818 in patients with locally advanced and metastatic BRAF mutated melanoma have confirmed the potency and the wide therapeutic index of LGX818 with clinical efficacy observed from the lowest tested dose of 50 mg/day up to the MTD of 450 mg/day. For further details, please refer to the current LGX818 Investigator's Brochure.

2.2.2 MEK162

MEK162 (binimetinib), previously named ARRY 438162, is a potent and selective allosteric, ATP (Adenosine Tri-Phosphate) non-competitive inhibitor of MEK1/2 that is active in inhibiting pERK and growth of BRAF mutant cancer cells in the low nanomolar range.

Acute, subchronic, chronic and reproductive toxicity, genotoxicity and phototoxicity studies were completed to support the chronic administration of MEK162 to adult cancer patients. The toxic effects of MEK inhibitors in humans are similar to the toxic effects observed in monkeys. The toxic effects include gastro-intestinal intolerance and diarrhea, rash, central serous retinopathy (only seen in humans) and retinal vein occlusion (rarely

seen in humans). In vitro and in vivo phototoxicity studies conducted in mice indicate that MEK162 has a very low risk of weak phototoxic potential at therapeutic doses. Furthermore, there has been no evidence of phototoxicity or photosensitivity in humans being treated with MEK162 for cancer or for rheumatoid arthritis. Given the embryo-lethal effects seen in rats and rabbits and the teratogenic effects seen in rabbits, MEK162 should not be used in pregnant women. Women of child-bearing potential must be advised to use highly effective contraception methods.

For further details on non-clinical pharmacology and toxicology, please refer to the current MEK162 Investigator's Brochure.

In oncology settings, MEK162 is currently being investigated both as a single agent and in combination with PI3K or RAF inhibitors in patients with selected advanced or metastatic solid tumors, including biliary cancer, colorectal cancer and melanoma.

The safety and efficacy of MEK162 as a single agent in patients with advanced melanoma have been evaluated in one Phase II study (CMEK162X2201), in which 45 mg and 60 mg bid dose levels have been investigated. In study CMEK162X2201, the most frequently treatment-related occurring events (≥ 20%) were dermatitis acneiform, diarrhea, peripheral edema, increase in creatin-kinase (CK) levels, nausea, fatigue and rash. The most common treatment-related Grade 3-4 AEs were CK increased, dermatitis acneiform and diarrhoea.

In the BRAF mutant population, the ORR was 20% (8 PR, 2 confirmed and 6 unconfirmed), and the median PFS was 3.6 months (CI: 2.0; 3.8) among the 41 patients treated with MEK162 at 45 mg bid (i.e. the recommended dose). In patients not previously treated with a BRAF inhibitor (n=34) the median PFS was 3.7 months. The majority of these patients received at least one prior anticancer treatment (*Ascierto et al, 2013*). For further details, please refer to the current MEK162 Investigator's Brochure.

2.2.3 LGX818 and MEK162 combination

PK data from the clinical study in BRAF mutated cancer patients up to doses of 600 mg od LGX818 in combination with 45 mg bid MEK162 show that, although the concentrations of MEK162 tended to be on the higher end of the variability range, the PK parameter of MEK162 remained unchanged when administered with 50 to 600 mg LGX818. Exposure to LGX818 on Day 15, as determined by Cmax and AUC, was 30 to 70% less compared with Day 1, likely due to CYP induction by LGX818 (auto-induction). PK characteristics of LGX818 when co-administered with MEK162 are similar to those observed when given as single agent.

Although clinical data from ongoing studies with LGX818 and MEK162 as single agents suggest that overlapping toxicities for the proposed combination, including effects on the skin (e.g. rash) and gastro-intestinal

system (e.g. nausea) may potentially be dose-limiting, initial reports with a similar class of compounds have shown that selective BRAF inhibitor combines safely with MEK inhibitor with a decreased occurrence of skin toxicities (rash, SCC). These data suggest that the combination of LGX818 with MEK162 may have an improved safety profile compared to the respective single agent therapies.

Soon after the initiation of clinical oncology trials with MEK inhibitors, it was observed that some participants developed an eye condition resembling central serous chorioretinopathy. A recently published article (Umer-Bloch et al, 2014) has examined the clinical features and management of MEK inhibitor-associated retinal syndromes in patients with advanced cutaneous melanoma treated with MEK162 in different Phase 1b or 2 clinical trials. Twenty patients on MEK162 monotherapy and 5 on MEK162 plus LGX818 underwent ophthalmological examinations at regular intervals, including determination of best corrected visual acuity, perimetry, colour vision testing, dilated fundus examination, and multimodal imaging. Grade 1-2 bilateral retinopathies with multiple lesions were observed in 13 of 20 patients on MEK162 monotherapy and in 2 of 5 patients on MEK162 plus LGX818. Retinopathy events appeared during the first 4 weeks, and in some cases, during the first few days of treatment. Patients reported mild and only short-lived visual symptoms. Optical coherence tomography revealed neuroretinal elevations. Central retinal thickness and volume showed dose-dependent increases after the start of treatment, followed by a marked decrease despite continued treatment, which was associated with symptom resolution. No vascular abnormalities were found with fluorescein and indocyanine green angiography. This analysis showed that treatment with MEK162 monotherapy or plus LGX818 induced transient retinopathy with multiple bilateral lesions in some patients. MEK162-induced retinopathy was usually mild, self-limiting, and tolerable as visual function was not seriously impaired.

2.2.4 Nivolumab

Nivolumab is a fully human, IgG4 (kappa) isotype, mAb that binds programmed death receptor-1 (PD-1). Blockade of PD-1 by monoclonal antibodies (mAbs) can enhance the anti--tumor immune response and result in tumor rejection.

In a previous study (*Topalian et al, 2014*), 107 patients with advanced melanoma enrolled between 2008 and 2012 received IV nivolumab in an outpatient setting every 2 weeks in 8-week treatment cycles for up to 96 weeks and were observed for overall survival, long-term safety, and response duration after treatment discontinuation. Nivolumab was administered at 1, 3, or 10 mg/kg during dose escalation. After completion of dose escalation, each dose cohort was expanded to accrue approximately 16 patients. Additional melanoma cohorts randomly assigned to 0.1, 0.3, and 1.0 mg/kg were enrolled. Median overall survival in nivolumab-treated patients (62% with two to five prior systemic therapies) was 16.8 months, and 1- and 2-year survival

rates were 62% and 43%, respectively. Among 33 patients with objective tumor regressions (31%), the Kaplan-Meier estimated median response duration was 2 years. Seventeen patients discontinued therapy for reasons other than disease progression, and 12 (71%) of 17 maintained responses off-therapy for at least 16 weeks (range, 16 to 56+ weeks). Objective response and toxicity rates were similar to those reported previously; in an extended analysis of all 306 patients treated on this trial (including those with other cancer types), exposure-adjusted toxicity rates were not cumulative.

In this study, OS following nivolumab treatment in patients with advanced treatment-refractory melanoma compares favourably with that in literature studies of similar patient populations. Responses were durable and persisted after drug discontinuation. Long-term safety was acceptable.

In the phase III trial (Robert et al.), 418 previously untreated patients who had metastatic melanoma without a BRAF mutation were randomized to receive nivolumab (at a dose of 3 mg per kilogram of body weight every 2 weeks and dacarbazine-matched placebo every 3 weeks) or dacarbazine (at a dose of 1000 mg per square meter of body-surface area every 3 weeks and nivolumab-matched placebo every 2 weeks). The primary end point was overall survival. At 1 year, the overall rate of survival was 72.9% (95% confidence interval [CI], 65.5 to 78.9) in the nivolumab group, as compared with 42.1% (95% CI, 33.0 to 50.9) in the dacarbazine group (hazard ratio for death, 0.42; 99.79% CI, 0.25 to 0.73; P<0.001). The median progression-free survival was 5.1 months in the nivolumab group versus 2.2 months in the dacarbazine group (hazard ratio for death or progression of disease, 0.43; 95% CI, 0.34 to 0.56; P<0.001). The objective response rate was 40.0% (95% CI, 33.3 to 47.0) in the nivolumab group versus 13.9% (95% CI, 9.5 to 19.4) in the dacarbazine group (odds ratio, 4.06; P<0.001). The survival benefit with nivolumab versus dacarbazine was observed across prespecified subgroups, including subgroups defined by status regarding the programmed death ligand 1 (PD-L1). Common adverse events associated with nivolumab included fatigue, pruritus, and nausea. Drugrelated adverse events of grade 3 or 4 occurred in 11.7% of the patients treated withnivolumab and 17.6% of those treated with dacarbazine.

Based on its mechanism of action and data from animal studies, nivolumab can cause fetal harm when administered to a pregnant woman. In animal reproduction studies, administration of nivolumab to cynomolgus monkeys from the onset of organogenesis through delivery resulted in increased abortion and premature infant death. Advise pregnant women of the potential risk to a fetus. Advise females of reproductive potential to use effective contraception during treatment with nivolumab and for at least 5 months after the last dose of nivolumab.

2.2.5 Ipilimumab

Ipilimumab is a recombinant, human monoclonal antibody that binds to the CTLA-4. Blockade of CTLA-4 augments T-cell activation and proliferation and ipilimumab works via T-cell mediated anti-tumor immune responses.

A variety of studies have demonstrated a clinically meaningful and statistically significant survival benefit with the use of ipilimumab in advanced melanoma.

A survival update at a follow-up of >5 years (5.5 to 6 years) for patients with advanced melanoma who previously received ipilimumab in phase II clinical trials has been recently published (*Lebbè et al, 2014*). Patients who previously received ipilimumab 0.3, 3, or 10 mg/kg in one of six phase II trials have been evaluated. Upon enrolment, patients initially received ipilimumab retreatment, extended maintenance therapy, or were followed for survival only. OS rates were evaluated in patients from 4 studies, and safety and best overall response during ipilimumab retreatment at 10 mg/kg were assessed in one study. Five-year OS rates for previously treated patients who received ipilimumab induction at 0.3, 3, or 10 mg/kg were 12.3%, 12.3% to 16.5%, and 15.5% to 28.4%, respectively. Five-year OS rates for treatment-naive patients who received ipilimumab induction at 3 or 10 mg/kg were 26.8% and 21.4% to 49.5%, respectively. Little to no change in OS was observed from year 5 up to year 6. The objective response rate among retreated patients was 23%. Grade 3/4 immune-related adverse events occurred in 25%, 5.9%, and 13.2% of retreated patients who initially received ipilimumab 0.3, 3, and 10 mg/kg, with the most common being observed in the skin (4.2%, 2.9%, 3.8%) and gastrointestinal tract (12.5%, 2.9%, 3.8%), respectively. At a follow-up of 5 to 6 years, ipilimumab continues to demonstrate durable, long-term survival in a proportion of patients with advanced melanoma. In some patients, ipilimumab retreatment can re-establish disease control with a safety profile that is comparable to that observed during ipilimumab induction.

There are no adequate and well-controlled studies of ipilimumab in pregnant women. Ipilimumab is classified in pregnancy category C. Ipilimumab should be used during pregnancy only if the potential benefit justifies the potential risk to the fetus. In a combined study of embryo-fetal and peri-postnatal development, pregnant cynomolgus monkeys received ipilimumab every 3 weeks from the onset of organogenesis in the first trimester through parturition, at exposure levels either 2.6 or 7.2 times higher by AUC than the exposures at the clinical dose of 3 mg/kg of ipilimumab. No treatment-related adverse effects on reproduction were detected during the first two trimesters of pregnancy. Beginning in the third trimester, the ipilimumab-treated groups experienced higher incidences of severe toxicities including abortion, stillbirth, premature delivery (with corresponding lower birth weight), and higher incidences of infant mortality in a dose-related manner compared to controls. Human IgG1 is known to cross the placental barrier and ipilimumab is an IgG1; therefore, ipilimumab has the potential to be transmitted from the mother to the developing fetus

2.2.6 Nivolumab and Ipilimumab combination

Recently, the results of a phase I study about the combination ipilimumab plus nivolumab in patients with advances melanoma have been reported (*Wolchock et al, 2013*). In this study, i.v. doses of nivolumab and ipilimumab were administered every 3 weeks for 4 doses, followed by nivolumab alone every 3 weeks for 4 doses (concurrent regimen). The combined treatment was subsequently administered every 12 weeks for up to 8 doses. In a sequenced regimen, patients previously treated with ipilimumab received nivolumab every 2 weeks for up to 48 doses. A total of 53 patients received concurrent therapy with nivolumab and ipilimumab, and 33 received sequenced treatment. The ORR for all patients in the concurrent-regimen group was 40%. Evidence of clinical activity (conventional, unconfirmed, or immune-related response or stable disease for ≥24 weeks) was observed in 65% of patients. At the maximum doses that were associated with an acceptable level of adverse events (nivolumab at a dose of 1 mg per kilogram of body weight and ipilimumab at a dose of 3 mg/kg), 53% of patients had an objective response, all with tumor reduction of 80% or more. Grade 3 or 4 adverse events related to therapy occurred in 53% of patients in the concurrent-regimen group but were qualitatively similar to previous experience with monotherapy and were generally reversible. Among patients in the sequenced-regimen group, 18% had grade 3 or 4 adverse events related to therapy and the objective-response rate was 20%. In this study, concurrent therapy with nivolumab and ipilimumab had a manageable safety profile and provided clinical activity that appears to be distinct from that in published data on monotherapy, with rapid and deep tumor regression in a substantial proportion of patients.
Recently updated data about the comination have been published (Wolchok et al, 2017). At a minimum follow-up of 36 months, the median overall survival had not been reached in the nivolumab-plus-ipilimumab group and was 37.6 months in the nivolumab group, as compared with 19.9 months in the ipilimumab group (hazard ratio for death with nivolumab plus ipilimumab vs. ipilimumab, 0.55 [P [P<0.001];hazard ratio for death with nivolumab vs. ipilimumab, 0.65 [P<0.001]). The overall survival rate at 3 years was 58% in the nivolumab-plus-ipilimumab group and 52% in the nivolumab group, as compared with 34% in the ipilimumab group. The safety profile was unchanged from the initial report. Treatment-related adverse events of grade 3 or 4 occurred in 59% of the patients in the nivolumab-plus-ipilimumab group, in 21% of those in the nivolumab group, and in 28% of those in the ipilimumab group.

2.3 Study rationale

The combination BRAF inhibitor plus MEK inhibitor seems to be more effective in the V600 BRAF mutated advanced melanoma patients compared to treatment with the BRAF inhibitors alone. In fact, a phase I-II study (*Flaherty et al, 2012*) showed a better ORR and PFS in the combination arm (dabrafenib plus trametinib) respect

to the single agent treatment (dabrafenib): 76% and 9.4 months versus 54% and 5.8 months respectively. Another phase I study with a similar combination (vemurafenib plus cobimetinib) showed an ORR of 85% in vemurafenib-naïve patients (*Martinez Garcia et al, 2012*). The above reported phase I study on the combination ipilimumab plus nivolumab (*Wolchock et al, 2013*) has shown that more than half of patients treated at the selected schedule (ipilimumab 3 mg/kg and nivolumab 1 mg/kg) had an objective response, all with tumor reduction of ≥ 80%. Reponses were durable, although longer follow-up is needed. A recent phase I study has shown a high rate of liver toxicity with the combo ipilimumab plus vemurafenib (*Ribas et al, 2013*) which makes difficult a combination with these two different drugs. Moreover, a better efficacy of the sequencing treatment BRAF inhibitors/ipilimumab vs. the single agent treatment was also observed; for this reason it was also suggested to start immunotherapy treatment in the BRAF V600 mutated melanoma population as first option, in order to increase the percentage of patients who can benefit from the sequencing (*Ascierto et al, 2012*; *Ascierto et al, 2013*), considering the possibility of a fast progression of the disease after the BRAF inhibitors treatment (*Ascierto et al, 2012*). Taking into account these considerations, it seems impossible to think to combine all the four compounds (the target agents and immunomodulating monoclonal antibodies). The risk of a high rate of toxicity is realistic and would render this approach inapplicable. Sequencing with these different combinations seems to be more feasible. However, also in this case it would be important to start with the best combination in order to give to the patients the best chance to increase the overall survival. The aim of this prospective randomized phase II study is to evaluate the sequencing of these different combinations and evaluate which is the best among these approaches.

Taking into account the recent updated data of Check-Mate 067 (Wolchok et al. N Engl J Med. 2017) In the two nivolumab-containing groups, the median overall survival was not reached among patients with BRAF mutations, and the rate of overall survival at 3 years was 68% in the nivolumab-plus-ipilimumab group and 56% in the nivolumab group. In a descriptive analysis, the hazard ratio for death with nivolumab plus ipilimumab versus nivolumab was 0.69 (95% CI, 0.44 to 1.07). Among patients without BRAF mutations, the median overall survival was reached in all three treatment groups.

Additional analyses were performed to investigate efficacy according to the tumor PD-L1 expression level. Descriptive comparisons between the two nivolumab-containing groups suggest that as the data become more mature, better survival outcomes may be obtained with combination therapy than with monotherapy in patients with a lower tumor PD-L1 expression level. However, overall survival was similar between the nivolumab-plus-ipilimumab group and the nivolumab group among patients with a tumor PD-L1 expression level of 1% or more or a level of 5% or more. The overall response rate was higher in the nivolumab-plus-ipilimumab group than in the nivolumab group at each tu- mor PD-L1 expression level tested.

Moreover in the light of the recent published data of Check-Mate 067 the combination therapy resulted in a higher rate of objective response than nivolumab alone regardless of the tumor PD-L1 expression level. Data from a phase 1b study, CA209-038, showed that tumor PD-L1 expression is up-regulated to a greater extent with combination treatment than with nivolumab alone, concomitant with a greater increase in interferon-γ, CXCL9,and CXCL10 expression in the tumor microenvironment. The ROC-curve analyses did not identify a threshold of tumor PD-L1 expression for the discrimination of a difference in overall survival, which suggests that the tumor PD-L1 expression level alone may not be a definitive predictive biomarker of outcomes in patients with advanced melanoma, thus the tumor PD-L1 testing is not required

2.4 Final Risk/Benefit Statement

The SECOMBIT study investigates the best sequence/combination strategy in patients with advanced melanoma. Furthermore, it incorporates an in-depth translational study with a wide range of biomarkers potentially useful to personalize treatment on the grounds of patient/disease's characteristics. Combinations and sequential treatment explored in the SECOMBIT study have been carefully selected on the grounds of the existing data in literature.

In a large phase III trial including 945 previously untreated patients with unresectable stage III or IV melanoma randomized to nivolumab alone, nivolumab plus ipilimumab, or ipilimumab alone, Larkin et al showed that the median progression-free survival was 11.5 months (95% confidence interval [CI], 8.9 to 16.7) with nivolumab plus ipilimumab, as compared with 2.9 months (95% CI, 2.8 to 3.4) with ipilimumab (hazard ratio for death or disease progression, 0.42; 99.5% CI, 0.31 to 0.57; P<0.001), and 6.9 months (95% CI, 4.3 to 9.5) with nivolumab (hazard ratio for the comparison with ipilimumab, 0.57; 99.5% CI, 0.43 to 0.76;P<0.001). In this trial, the increased effectiviness of the combination was obtained at the cost of increased toxicity, with treatment-related adverse events of grade 3 or 4 occurring in 16.3% of the patients in the nivolumab group, 55.0% of those in the nivolumab-plus-ipilimumab group, and 27.3% of those in the ipilimumab group.

Similar to the combination of nivolumab plus ipilumumab, the combination of two targeted agents has also shown incremental benefits vs. the use of single agents. In a double-blind, randomised, placebo-controlled, multicentre study conducted in 490 BRAFV600 mutation-positive advanced IV melanoma patients randomly assigned (1:1) to receive anti-MEK agent cobimetinib or placebo, in combination with oral vemurafenib, median progression-free survival was 12·3 months (95% CI 9·5–13·4) for cobimetinib and vemurafenib versus 7·2 months (5·6–7·5) for placebo and vemurafenib (HR 0·58 [95% CI 0·46–0·72], p<0·0001).

This advantage in progression free survival translated into a median overall survival was 22·3 months (95% CI 20·3–not estimable) for cobimetinib and vemurafenib versus 17·4 months (95% CI 15·0–19·8) for placebo and vemurafenib (HR 0·70, 95% CI 0·55–0·90; p=0·005).
On the other hand, combination of one immunotherapy and one targeted agent has been associated with an unacceptable toxicity profile, as shown in a recent phase I study about the combination of ipilimumab plus vemurafenib (Ribas et al, 2013), which reported high rates of liver toxicity. All hepatic adverse events were asymptomatic and reversible with either temporary discontinuation of the study drugs or administration of glucocorticoids.

The risks of administration of a combination of ipilimumab and nivolumab are known to be potential clinically meaningful drug-related AEs, which may require early recognition and prompt intervention. Management algorithms have therefore been developed for suspected pulmonary toxicity, GI, hepatotoxicity, endocrinopathy, skin toxicity, neurological toxicity and nephrotoxicity (Appendix III). Recommendations are to follow the nivolumab Investigator's Brochure adverse event algorithms. Dosing delay or discontinuation may then be implemented based on individual safety and tolerability (Section 5.2.2).

Ongoing clinical studies suggest that the combination of LGX818 with MEK162 may in fact have an improved safety profile compared to the respective single agent therapies. However, known risks of this combination comprise MEK inhibitor-associated retinal syndromes and other toxic effects on the skin (e.g. rash) and gastro-intestinal system (e.g. nausea). Again, management algorithms are in place (Appendices I and II) to enable timely intervention if required. Dosing modifications, interruptions and delays can then be implemented based on individual safety and tolerability (Section 5.1.2).
Given these considerations, the SECOMBIT study explores the effectiveness of the combined use of 2 immunotherapy agents and of 2 targeted treatment agents, following different sequences of administration.
In conclusion, the risks associated with participation to the SECOMBIT study are those known and predictable on the grounds of the available evidence regarding the toxicity of the combinations explored.
The benefits are highlighted by the proven efficacy of the combination of two immunotherapy or two targeted agents; in this regard it must be noted that not only will patients enrolled in the SECOMBIT study have access to both of these doublet combinations administered according to different sequences and timings, but they will also undergo an extensive biomarker analysis.
Such analysis has the potential to identify useful tools for optimization of treatment, with improved treatment efficacy and better allocation of financial resources.
Updated data were reported on targeted therapies, confirming the excellent results previously reported (Larkin J e t al. Combined vemurafenib and cobimetinib in BRAF-mutated melanoma. N Engl J Med 371:1867–1876

2014; Long GV et al. Combined BRAF and MEK inhibition versus BRAF inhibition alone in melanoma. N Engl J Med 371:1877–1888 2014)

An update on the CoBRIM trial of combined BRAF inhibitor (vemurafenib) plus MEK inhibitor (cobimetinib) in patients with BRAFV600 mutation-positive tumors confirmed its superior impact on progression-free survival (PFS) compared to vemurafenib monotherapy [12.3 vs 7.2 months; hazard ratio (HR) 0.58 (0.46–0.72)]. An update on overall survival (OS) from the Combi-D study of combined dabrafenib plus trametinib in patients with BRAF V600E/K metastatic melanoma was also reported (Long GV et al. 2015).

Patients treated with the combination of dabrafenib and trametinib achieved a median OS of 25.1 months with 51% of patients still alive at 2 years, these findings confirmed results reported from the phase I–II study in 2014 (Flaherty et al. 2014)

Finally, data from a phase Ib/II open-label study of patients with BRAFV600-mutant cutaneous melanoma treated with the newer combination of encorafenib plus binimetinib showed an overall response rate (ORR) of 74.5% and a disease control rate (DCR) of 96.4%. Of interest, in the cohort receiving a dosage regimen of encorafenib 400/450 mg and binimetinib 45 mg, the ORR was 77.5% and the DCR was 100%. e combination was also well tolerated, with no grade 3–4 pyrexia or skin tox- icity events reported (Sullivan RJ et al. 2015).

3. STUDY AIMS AND DESIGN

This prospective randomized phase II study is aimed at evaluating the effects of the sequencing of the two different tested combinations (Combo Target: LGX818 450 mg p.o. od + MEK162 45 mg p.o. bid; Combo Immuno: nivolumab 1 mg/kg solution i.v. combined with ipilimumab 3 mg/kg solution i.v. every 3 weeks for 4 doses then nivolumab 3 mg/kg solution i.v.every 2 weeks or 240 mg every 2 weeks or 480 mg every 4 weeks) and at evaluating which is the best of the three tested approaches.

3.1 Objectives of the study

Primary Objectives

The primary objective is to define the best sequencing combination treatment in primary efficacy variable OS.

Secondary Objectives

The secondary objectives are to evaluate the effects of the two sequencing combination treatments on:

- Total PFS;
- 3 years PFS rate;
- Percentage of patients alive at 3 years;
- Best overall response rate (BORR);
- Duration of response (DoR);
- Biological markers (biomarker study).
- Toxicity of the investigational medicinal products (IMPs).
- Quality of life and general health status defined by:
    - Health-related quality of life (HRQoL), by means of the 30-item European Organisation for Research and Treatment of Care quality of life questionnaire (EORTC QLQ-C30);
    - General health status, by means of the European Quality of Life 5-Dimensions (EQ-5D) questionnaire;
    - Impairment of work productivity and activity, by means of the Work Productivity and Activity Impairment: General Health (WPAI:GH) questionnaire;

Biological markers (biomarker study)

The objective of the biomarker study (to be conducted in a subgroup of approximately 80-90 patients) is to focus on understanding mechanisms of action/resistance. In particular, the biomarker study will inform on how to

sequence targeted RAF/MEK agents with immunotherapy agents (i.e. ipilumumab and nivolumab) in melanoma and will be hypothesis-generating only.

3.2 Endpoints of the study

Primary Endpoint

OS is the primary efficacy endpoint of the study. OS will be calculated as the time from the date of randomization until the date of death from any cause. Any patient not known to have died at the time of data analysis will be censored at the time of the last recorded date on which the patient was known to be alive.

Secondary Endpoints

- Total PFS, calculated from the date of randomization until the date of the second progression (i.e. the progression to second treatment). Any progression or death will be considered as an event if patient cannot complete treatment sequence;
- 3 years PFS rate; calculated from the date of randomization;
- Percentage of patients alive at 3 years;
- Best overall response rate (BORR);
- Duration of response (DoR) calculated as the time from the date of the first documented response (CR or PR) until the date of the first documented progression or death due to underlying cancer. If the patient with a CR or PR has no progression or death due to underlying cancer, the patient will be censored at the date of last adeguate tumor assessment;
- Health-related quality of life (HRQoL), by means of the 30-item European Organisation for Research and Treatment of Care quality of life questionnaire (EORTC QLQ-C30);
- General health status, by means of the European Quality of Life 5-Dimensions (EQ-5D) questionnaire;
- Impairment of work productivity and activity, by means of the Work Productivity and Activity Impairment: General Health (WPAI:GH) questionnaire;
- Biological markers (biomarker study).

Biological markers (Biomarker study)

<u>Tumor tissue biomarkers:</u>

- Immune status: CD3, CD8, and CD4 T cells; Activated T cells; T regulatory cells; Dendritic cells;
- Resistance to immunotherapy agents: Checkpoint receptors/ligands; myeloid-derived suppressor cells (MDSCs);

- Resistance to targeted agents: aberrations in MEK/PI3K pathways, cytokines that interact with tyrosine kinase receptors (VEGF, HGF and their cognate receptors);
- Mutational load and neoantigen profile.

Peripheral blood biomarkers:

- Immune status/“Resistance” to immunotherapy agents: Activated T cells; Memory/Exhausted T cells; T regulatory cells; MDSCs; Inflammatory response; C-reactive protein (CRP); TCR Sequencing/Gene Expression analysis;
- Response/Resistance to targeted agents: Apoptotic tumor cells (as measured by circulating tumor DNA); soluble hepatocyte growth factor (sHGF); serum vascular endothelial growth factor (sVEGF); soluble interleukine-2 receptor (sCD25);
-

Study Aim	Objectives	Endpoint
**Primary**	The primary objective of this study is to define the best sequencing combination treatment in primary efficacy variable OS.	Overall Survival (OS) will be calculated as the time from the date of randomization until the date of death from any cause. Any patient not kinow to have died at the time of data analysis will be censored at the time of the last recorded date on which the patient was known to be alive.
**Secondary**	The secondary objectives of the study are to evaluate the effects of the two sequencing combination treatments on:<br>• Total PFS;<br>• 3 years PFS rate;<br>• Percentage of patients alive at 3 years;<br>• Best overall response rate (BORR);<br>• Duration of response (DoR);<br>• Biological markers (biomarker study);<br>• Toxicity of the investigational medicinal products (IMPs);<br>• Quality of life and general health status defined by:<br>- Health-related quality of life (HRQoL), by means of the 30-item European Organisation for Research and Treatment of Care quality of life questionnaire (EORTC QLQ-C30);<br>- General health status, by means of the European Quality of Life 5-Dimensions (EQ-5D) questionnaire;<br>- Impairment of work productivity and activity, by means of the Work Productivity and Activity Impairment:	• Total PFS, calculated from the date of randomization to the date of the second progression (i.e. the progression to second treatment); any progression or death will be considered as an event if patient cannot complete treatment sequence;<br>• 3 years PFS rate, calculated from the date of randomization;<br>• Percentage of patients alive at 3 years;<br>• Best overall response rate (BORR);<br>• Duration of response (DoR), calculated as the time from the date of first documented response (CR o PR) until the date of the first documented progression or death due to underlying cancer. If a patient with a CR o PR has no progression or death due to underlying cancer, the patient will be censored at the date of last adeguate tumor assessment;<br>• Health-related quality of life (HRQoL), by means of the 30-item European Organisation for Research and Treatment of Care quality of life questionnaire (EORTC QLQ-C30);<br>• General health status, by means of the European Quality of Life 5-Dimensions (EQ-5D) questionnaire;

	General Health (WPAI:GH) questionnaire.	• Impairment of work productivity and activity, by means of the Work Productivity and Activity Impairment: General Health (WPAI:GH) questionnaire;<br>• Biological markers (biomarker study).

Table 4: Summary of study aim – Principal Study

Study Aim	Objectives	End point
**Biomarker Study**	The objective of the biomarker study is to focus on understanding mechanisms of action/resistance. In particular, the biomarker study:<br>• Will inform how to sequence targeted RAF/MEK agents with immunotherapy agents (i.e. ipilumumab and nivolumab) in melanoma;<br>• Will be hypothesis-generating only.	Tumor tissue biomarkers:<br>• Immune status: CD3, CD8, CD4, CD45RO and CD11c T cells; Activated T cells; T regulatory cells; Dendritic cells;<br>• Resistance to immunotherapy agents: Checkpoint receptors/ligands; myeloid-derived suppressor cells (MDSCs);<br>• Resistance to targeted agents: aberrations in MEK/PI3K pathways; mRNA expression (nanostring method).<br>Peripheral blood biomarkers:<br>• Immune status/ "Resistance" to immunotherapy agents: Activated T cells; Memory/Exhausted T cells; T regulatory cells; MDSCs; Inflammatory response; CRP; TCR Sequencing/Gene Expression analysis;<br>• Response/Resistance to targeted agents: Apoptotic tumor cells (as measured by circulating tumor DNA); soluble hepatocyte growth factor (sHGF); serum vascular endothelial growth factor (sVEGF); soluble intercellular adhesion molecule-1 (sICAM-1), soluble interleukine-2 receptor (sCD25);<br>• .

Table 5: Summary of study aim – Traslational Study

3.3 Study Design

The study will be conducted according to an open-label, prospective, randomized, phase II design.

Randomization will be stratified according to stage arranged in the 3 following strata:

- IIIb/c – M1a – M1b;
- M1c with normal LDH (≤ 2ULN);
- M1c with elevated LDH (> 2 ULN).

Subjects will be assessed for response by computed tomography (CT) or Magnetic Resonance Imaging (MRI). All measurable and non-measurable lesions must be documented at screening (within 28 days prior to randomization) and re-assessed at each subsequent tumor evaluation (every 8 weeks (+/- 1 week) for the first year, every 12 weeks (+/- 1 week) while the patient is on study). Tumor assessments with CT or MRI scans of the brain, chest, abdomen, and pelvis will be performed until disease progression after the second combo treatment per RECIST v1.1. Imaging of the neck should be included if clinically indicated. In the event PET/CT scanner is used for tumor assessments, the CT portion of the PET/CT must meet criteria for diagnostic quality. All scans can be collected for a possible independent review.

For patients who discontinue study treatment for reason other than investigator–determined disease progression, tumor assessments should continue to be performed as scheduled.

Patients will continue to be on study and can be switched to the subsequent therapy after PD as per protocol in both arm A and B.

In case of an interruption of the treatment in arm C, fors reasons other than investigator–determined disease progression, during the first 8 weeks (2 cycles of therapy), the patient can continue the study treatment with combo immuno, as scheduled.

For patients with palpable/superficial lesions, clinical disease assessments by physical examination should be performed at baseline and throughout study treatment as clinically indicated. Color photographs with ruler/calipers will be taken at baseline and at all subsequent tumor assessment time points.

The National Cancer Institute Common Toxicity Criteria for Adverse Events (NCI CTC-AE) Version 4.03 will be used to evaluate the clinical safety of the treatment in this study. Patients will be assessed for AEs at each clinical visit and as necessary throughout the study.

<u>Biomarker study</u>

A correlative biological study will be performed for the evaluation of biomarkers on the biological samples available (paraffin-embedded tissue, frozen tissue, blood, serum, etc.). Approximately 80-90 patients will take part in the biomarkerstudy.

3.4 Study Schedule

The following IMPs will be used in the study according the scheme shown in Figure 1:

- **Arm A**: Combo Target (LGX818 450 mg p.o. od + MEK162 45 mg p.o. bid) until PD; then Combo Immuno (nivolumab 1 mg/kg solution i.v. combined with ipilimumab 3 mg/kg solution i.v. every 3 weeks for 4

doses then nivolumab 3 mg/kg solution i.v. every 2 weeks or 240 mg every 2 weeks or 480 mg every 4 weeks) until PD.

- **Arm B**: Combo Immuno (nivolumab 1 mg/kg solution i.v. combined with ipilimumab 3 mg/kg solution i.v. every 3 weeks for 4 doses then nivolumab 3 mg/kg solution i.v. every 2 weeks or 240 mg every 2 weeks or 480 mg every 4 weeks) until PD; then Combo Target (LGX818 450 mg p.o. od + MEK162 45 mg p.o. bid) until PD.
- **Arm C**: Combo Target (LGX818 450 mg p.o. od + MEK162 45 mg p.o. bid) for 8 weeks followed by Combo Immuno (nivolumab 1 mg/kg solution i.v. combined with ipilimumab 3 mg/kg solution i.v. every 3 weeks for 4 doses then nivolumab 3 mg/kg solution i.v. every 2 weeks or 240 mg every 2 weeks or 480 mg every 4 weeks) until PD; then Combo Target (LGX818 450 mg p.o. od + MEK162 45 mg p.o. bid) until PD.

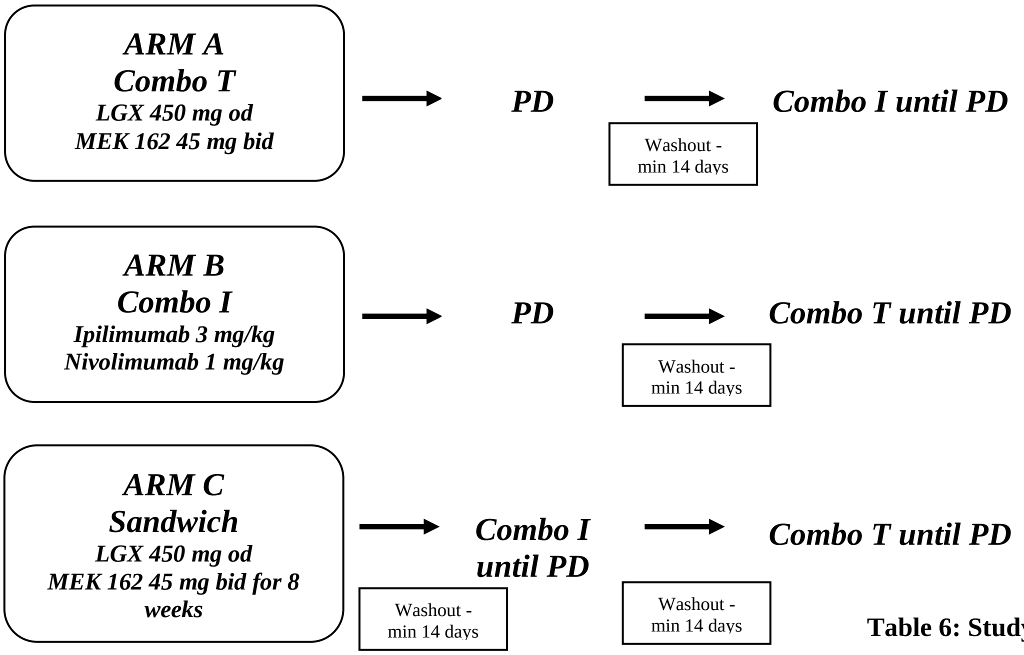

Table 6: Study treatment scheme

Shift from the one combination therapy to the following one can be done (once the procedures of the new screening have been completed) in case of absence of AEs events from the previous combination therapy or in case of grade 1 AEs. In case of grade ≥ 2 AEs from the previous combination therapy, shift to the following combination therapy can be done only when the AE is resolved or has decreased in intensity to at least grade 1.

If the AE does not resolve or decrease to at least grade 1, during the second screening period (28 days), patient should be followed up for additional 28 days.

All screening procedures must be repeated before starting the new combination therapy (including tumor assessment, to exclude progression disease). If AE does not resolve or decrease to at least grade 1, during this additional period (until 56 days after PD1), patient will be permanently discontinued.

Shift from one combination therapy to the following one requires minimum 14 days for washout.
As seen in the previous study GP28384, the combination of andi-PDL1 and combo target therapy resulted in a tolerable and manageable safety profile, with no grade 5 fatal events.

In Study GP28384, treatment with atezolizumab plus vemurafenib was first evaluated, and a tolerable regimen was found. Subsequently, evaluation of the atezolizumab plus cobimetinib plus vemurafenib (atezolizumab + cobimetinib + vemurafenib) regimen was initiated and is ongoing with preliminary safety and efficacy information available. Fifteen patients have been treated with atezo + cobi + vem as of 1 March 2016 and were evaluable for safety. The median safety follow-up from the first dose of any study drug is 5.78 months (1.5−13.3 months). Overall treatment with atezo + vem ± cobi, resulted in a safety profile that appears tolerable and manageable, with no Grade 5 (fatal) events and no atezolizumab−related serious adverse events. Overall, there were no unexpected adverse events, and the majority of adverse events were mild or moderate in severity, manageable with dose modification, and generally reversible.

Treatment with any of the treatment schemes will be continued until the development of progressive disease (as per Investigator assessment), unacceptable toxicity, consent withdrawal, death, reasons deemed by the treating physician or study termination by the Sponsor. To reduce the risk of long-term toxicity, no more than 2 years of ipilimumab and nivolumab dosing will be administered to study patients.

Follow-up will continue until the patient has documented disease progression, starts another cancer therapy or withdraws consent.

Patients in the ARM C, with a progression disease documented at the first tumor evaluation, will be discontinued from the study and will be treated as per institutional standard of care thereafter.

Patients who discontinue from the study will be asked to return to the clinic within 28 days of the last dose of IMP for the follow-up visit and to be contacted every 12 weeks until 60 months from randomization for long term survival follow-up.

3.5 Schedule of Assessments and Procedures

All screening/baseline assessments as outlined in Tables 1 (arm A), 2 (arm B) and 3 (arm C) must be performed within 28 days prior to the first administration of study drugs on Day 1. Results of tests or examinations performed as standard of care before obtaining informed consent and within the 28 days prior to commencing study drugs may be used. All assessments during the study must be performed within a window +/- 3 days of

the day indicated on the schedule of assessment, except for tumor evaluations for which a window of +/- 7 days will apply and for Combo Immuno visits for which a window of +/- 2 days will apply.

Eligibility for the study will be determined by the Investigator from the mandatory screening/baseline assessments performed during screening and according to the study inclusion/exclusion criteria.

First dosing of study drugs will be determined by the patient's eligibility and the laboratory assessments done on Day 1 prior to dosing.

Hematology and biochemistry assessments do not need to be repeated on Day 1 if performed within 7 days; if it is necessary to repeat these blood tests, the results must be known before the patient receives treatments to ensure inclusion/exclusion criteria related to these tests are met. Test for HIV infection is mandatory at screening.

Subject Re-enrollment: This study permits the re-enrollment of patients that was not randomized within 28 days from ICF signature, after obtaining agreement from the medical monitor prior to re--enrolling a subject. If authorized to re-enrollment, the patient must be re-consented and the same subject-code can be used.

3.5.1 Screening Examination and Eligibility Screening Form

Written informed consent must be obtained before any study specific assessments or procedures are performed. All screening/baseline evaluations must be performed between Day -28 and -1. Patients who fulfill all the inclusion and none of the exclusion criteria will be accepted into the study.

3.5.2 Procedures for Enrolment of Eligible Patients

A patient who has fulfilled the entry criteria will be given an identifying number. Each identifying number will be unique to the patient for whom it is issued. A patient number will not be re-used if the patient leaves the study. Under no circumstances will patients who enroll in this study and have completed treatment as specified be permitted to re-enroll in the study. A Patient Enrolment and Identification Code List must be maintained by the Investigator.

Eligible patients will be then randomised in one of the three arms in a 1:1:1 ratio, i.e. an equal number of patients will be assigned to one of the three treatment sequences. In each arm and overall, the randomisation will be stratified according to the stage of disease and baseline value of LDH (normal or high – See section 3.3 of the protocol).

3.5.3 Clinical Assessments and Procedures

The following clinical assessments and procedures must be completed for all patients enrolled in this study at screening/baseline and during study visits. All assessments must be performed within a window of 3 days prior to the scheduled visit date and 3 days after the scheduled visit date (- 3 days / + 3 days) for each visit indicated on the schedule of assessment, except for tumor evaluations for which a window of +/- 7 days will apply and for Combo Immuno visits for which a window of +/- 2 days will apply.

Please refer to Tables 1 (Arm A), 2 (Arm B) and 3 (Arm C) for specific details and time points collected on clinical assessments and procedures outlined below.

Screening/Baseline 1,

- Informed Consent Form;
- Demographic data (age, gender, race);
- Medical history (including demographics, relevant medical history last 5 years, previous and current diseases, prior therapies for melanoma including surgeries and relative responses, prior skin cancer history, therapies and procedures, all medications started within 14 days prior to screening visit);
- Physical exam including height (screening only) and weight and skin examination. Changes from baseline abnormalities should be recorded at each subsequent physical examination. New or worsened abnormalities should be recorded as AEs if appropriate. Tumor lesions accessible by physical examination should be recorded as well for biopsy purpose also. For patients with palpable/superficial lesions, clinical disease assessments by physical examination should be performed at baseline and throughout study treatment as clinically indicated. Color photographs with ruler/calipers will be taken at baseline and at all subsequent tumor assessment time points;
- Vital signs (respiratory rate, pulse, blood pressure and temperature) will be obtained in the same position, as appropriate prior to any blood collection. Two readings of supine blood pressure, in the same arm, separated by 2 min should be recorded in the Source Documents and the mean of the two consecutive readings, should be recorded in the eCRF throughout the study;
- A 12-lead ECG;
- ECOG PS;
- Ophthalmological and dermatological examination;
- Echocardiogram or MUGA;
- Hematology (including hemoglobin, hematocrit, red blood cells, white blood cells, neutrophilis, lymphocytes, monocytes, eosinophhilis, basophilis, platelets count);

- Biochemistry (including glucose, sodium, potassium, calcium, chloride, bicarbonate, magnesium, urea, uric acid, albumin, creatinine, creatinine clearance, total protein, total bilirubin, indirect bilirubin, alkaline phosphatase, ALT, AST, Gamma glutamyltransferase, HDL, LDL, amylase, lipase);
- Serum pregnancy test (within 24 hours prior to commencement of dosing) for women of child-bearing potential confirmed by serum HCG laboratory test;
- Coagulation (including INR, aPTT, PTT, Protrombin time, fibrogen)
- Urinalysis (including glucose, protein, blood, bilirubin, ketones, leukocytes esterase, UWBC/HPF; URBC/HPF, bacteria);
- Thyroid function test (including TSH, Free T4, Free T3);
- Endocrine Panel (including ACTH, total cortisol);
- Screening for HIV, HBV and HCV infections;
- Presence of BRAF V600E or V600K mutation in tumor tissue prior to enrolment;
- Cardiac/Muscle Enzymes: Troponin, Creatine Kinase (CK). If total CK ≥3 X ULN, then measure isoenzymes and myoglobin in blood or urine weekly;
- Tumor assessments including measurable and non-measurable lesions (brain CT or MRI at all baselines, CT/MRI C/A/P, bone scan if clinically indicated);
- Assessment of HRQoL (EORTC QLQ-C30), general health status (EQ-5D) and impairment of work productivity and activity (WPAI:GH);
- Concomitant therapies;
- Assessment of AEs (including SAEs) from time signed Informed Consent is obtained until first dose of study drugs;
- Biopsy of tumoral lesions for the biological study;
- Blood collection for the biological study.

During Study

- Physical exam at every visit;
- Vital signs (respiratory rate, pulse, blood pressure and temperature) will be obtained in the same position, as appropriate prior to any blood collection. Two readings of supine blood pressure, in the same arm, separated by 2 min should be recorded in the Source Documents and the mean of the two consecutive readings, should be recorded in the eCRF throughout the study ECOG PS at every visit;
- A 12-lead ECG will be performed at screening/baseline, at 2nd baseline (and at 3rd baseline in ARM C only) at the 1th month after each baseline and every 12 weeks while the patient is on study;

- The ophthalmologic examination will be performed at the 2nd baseline (and at 3rd baseline in ARM C only) visit and then when clinically indicated;
- The dermatologic evaluation will be performed at the 2nd baseline visit (and at 3rd baseline in ARM C only) and every 8 weeks while the patient is on study;
- The echocardiogram or MUGA will be performed at screening/baseline, at the 2nd baseline (and at the 3rd baseline ARM C only), at the 1st month after each baseline and every 12 weeks while the patient is on study;
- Hematology (including hemoglobin, hematocrit, red blood cells, white blood cells, neutrophilis, lymphocytes, monocytes, eosinophhilis, basophilis, platelets count) at every visit;
- Coagulation (including INR, aPTT, PTT, Protrombin time, fibrogen); Biochemistry (including glucose, sodium , potassium , calcium , choloride bicarbonate [if routinely performed on venous blood samples], magnesium, urea, uric acid, albumin, , creatinine or creatinine clearance, total protein, total bilirubin with fractionation into direct and indirect (if total bilirubin elevated), alkaline phosphatase, AST [SGOT], ALT [SGPT], lactate dehydrogenase (LDH), Gamma glutamyltransferase, HDL, LDL, amylase, lipase, BUN, at every visit;
- Thyroid function test (including TSH, Free T4, Free T3) at every visit;
- Endocrine Panel (including ACTH, total cortisol) to be performed at every vist only during the combo immuno therapy (nivolumab and ipilimumab);
- A serum/urine pregnancy test to be performed every 6 (± 1) weeks (within 24 hours prior to administration of study drug both serum or urine are accepted) during the treatment Combo-Immuno and every 4 (± 1) weeks (within 24 hours prior to administration of study drug both serum or urine are accepted) during the treatment Combo-Target;
- Cardiac/Muscle Enzymes at every visit: Troponin, Creatine Kinase (CK). If total CK ≥3 X ULN, then measure isoenzymes and myoglobin in blood or urine weekly;
- Urinalysis (only if clinically indicated) including glucose, protein, blood, bilirubin, ketones, leukocytes esterase, UWBC/HPF; URBC/HPF, bacteria);
- Biopsy of tumoral lesions for the biological study (if applicable);
- Blood collection for the biological study will be taken (if applicable);
- Tumor assessments of both measurable and non-measurable disease (CT/MRI of brain at all baselines and as clinically indicated during treatment period, CT/MRI C/A/P, bone scan if clinically indicated) every 8 weeks (+/- 1 week) for the first year and every 12 weeks (+/- 1 week) thereafter while the patient is on study and until PD is observed in case of treatment discontinuation due to adverse event;

- Assessment of HRQoL (EORTC QLQ-C30), general health status (EQ-5D) and impairment of work productivity and activity (WPAI:GH) will be performed every 28 days during the Combo Target therapy and every 21 days at administration of nivolimumab+ipilimumab (i.e. every 21 days for 4 doses) and then every two administrations (i.e., every 28 days) of nivolimumab during the Combo Immuno therapy;
- Concomitant therapies throughout the study;
- Assessment of AEs (including SAEs) throughout the study;
- IMPs administration throughout the study.

End of treatment Visit (when patient permanently discontinues all study drugs)

- Physical exam;
- Vital signs (respiratory rate, pulse, blood pressure and temperature) will be obtained in the same position, as appropriate prior to any blood collection. Two readings of supine blood pressure, in the same arm, separated by 2 min should be recorded in the Source Documents and the mean of the two consecutive readings, should be recorded in the eCRF throughout the study12-lead ECG (if clinically indicated);
- ECOG PS;
- Hematology (including hemoglobin, hematocrit, red blood cells, white blood cells, neutrophilis, lymphocytes, monocytes, eosinophhilis, basophilis, platelets count);
- Coagulation (including INR, aPTT, PTT, Protrombin time, fibrogen);
- Biochemistry (including glucose, sodium, potassium, calcium, choloride bicarbonate [if routinely performed on venous blood samples], magnesium, urea, uric acid, albumin, creatinine or creatinine clearance, total protein, total bilirubin with fractionation into direct and indirect (if total bilirubin elevated), alkaline phosphatase, AST [SGOT], ALT [SGPT], lactate dehydrogenase (LDH), Gamma glutamyltransferase, HDL, LDL, amylase, lipase BUN;
- Thyroid function test (including TSH, Free T4, Free T3):
- Cardiac/Muscle Enzymes: Troponin, Creatine Kinase (CK). If total CK ≥3 X ULN, then measure isoenzymes and myoglobin in blood or urine weekly;
- Endocrine Panel (including ACTH, total cortisol) to be performed at every vist only during the combo immuno therapy (nivolumab and ipilimumab);
- Urinalysis (including glucose, protein, blood, bilirubin, ketones, leukocytes esterase, UWBC/HPF; URBC/HPF, bacteria);
- Pregnancy test (serum or urine);

- Tumor assessments if not performed within the prior 6 weeks (CT/MRI C/A/P, CT/MRI of brain as clinically indicated) including assessment of any tumor lesions accessible by physical examination;
- Assessment of HRQoL (EORTC QLQ-C30), general health status (EQ-5D) and impairment of work productivity and activity (WPAI:GH);
- Concomitant therapies;
- Assessment of AEs (including SAEs);

Follow up visit within 28 days from discontinuation of study drugs

- Physical exam;
- Vital signs (respiratory rate, pulse, blood pressure and temperature) will be obtained in the same position, as appropriate prior to any blood collection. Two readings of supine blood pressure, in the same arm, separated by 2 min should be recorded in the Source Documents and the mean of the two consecutive readings, should be recorded in the eCRF throughout the study ECOG PS;
- Hematology ((including hemoglobin, hematocrit, red blood cells, white blood cells, neutrophilis, lymphocytes, monocytes, eosinophhilis, basophilis, platelets count);
- Coagulation (including INR, aPTT, PTT, Protrombin time, fibrogen);
- Biochemistry (including glucose, sodium, potassium, calcium, choloride bicarbonate [if routinely performed on venous blood samples], magnesium, urea, uric acid, albumin, creatinine or creatinine clearance, total protein, total bilirubin with fractionation into direct and indirect (if total bilirubin elevated), alkaline phosphatase, AST [SGOT], ALT [SGPT], lactate dehydrogenase (LDH), Gamma glutamyltransferase, HDL, LDL, amylase, lipase, BUN;
- Thyroid function test (including TSH, Free T4, Free T3) to be performed at every vist only during the combo immuno therapy (nivolumab and ipilimumab);
- Cardiac/Muscle Enzymes: Troponin, Creatine Kinase (CK). If total CK ≥3 X ULN, then measure isoenzymes and myoglobin in blood or urine;
- Serum/urine pregnancy test for a follow up period of 31 weeks after the last dose of nivolumab/ipilimumab;
- Tumor assessments if not performed within the prior 6 weeks and until PD is observed in case of treatment discontinuation due to adverse event (CT/MRI C/A/P, CT/MRI of brain as clinically indicated) including assessment of any tumor lesions accessible by physical examination;
- Assessment of HRQoL (EORTC QLQ-C30), general health status (EQ-5D) and impairment of work productivity and activity (WPAI:GH);
- Monitoring of AEs and SAEs;

- Follow up for disease progression for those patients who have discontinued study drug for any reason (i.e. AE, etc) other than disease progression;
- Administration of alternative anti-cancer therapy.

Long term follow-up visits every 12 weeks until 60 months from randomization

- Monitoring of AEs and SAEs;
- Follow up for disease progression for those patients who have discontinued study drug for any reason (i.e. due to AE, etc) other than disease progression;
- Serum pregnancy test for a follow up period of 31 weeks after the last dose of nivolumab/ipilimumab;
- Administration of alternative anti-cancer therapy.

3.5.4 Biomarker study

Tumor biopsies

The biomarker study requires tumor biopsies at baseline, on-treatment (within first 4 weeks after first dose), and upon progression when feasible. The biopsies at first baseline and at first disease progression are mandatory and part of the main study. All other biopsies are optional as described below per each arm and will be performed only upon patient specific consent.

In arm A, biopsy of tumoral lesions for the biological study will be performed at baseline 1, Week 4 and disease progression 1 (PD 1) during Combo Target therapy; and at Week 4 and disease progression 2 (PD 2) during Combo Immuno therapy. Biopsy of tumoral lesions will be mandatory at baseline 1 and at the first disease progression (PD1) and will be optional at the other time-points. 3

In arm B, biopsy of tumoral lesions for the biological study will be performed at baseline 1, Week 4 and disease progression 1 (PD 1) during Combo Immuno therapy; and at Week 4 and disease progression 2 (PD 2) during Combo Target therapy. Biopsy of tumoral lesions will be mandatory at baseline 1 and at the first disease progression (PD1) and will be optional at the other time-points.

In arm C, biopsy of tumoral lesions for the biological study will be performed at baseline 1, Week 4 of Combo Immuno therapy and disease progression 1 (PD 1) during Combo Immuno therapy; and at Week 4 and disease progression 2 (PD 2) during the 2nd Combo Target therapy. Biopsy of tumoral lesions will be mandatory at baseline 1 and at the first disease progression (PD 1) and will be optional at the other time-points.

The investigator, in consultation with the radiology staff, must determine the degree of risk associated with the procedure and find it acceptable.

Biopsies may be done with local anesthesia or conscious sedation. Institutional guidelines for the safe performance of biopsies should be followed.

Excisional biopsies may be performed to obtain tumor biopsy samples.

Invasive procedures that require general anesthesia should not be performed to obtain a biopsy specimen.

However, if a surgical procedure is performed for a clinical indication, excess tumor tissue may be used for research purposes with the consent of the subject.

Tumor biopsies samples will be taken according to the following scheme

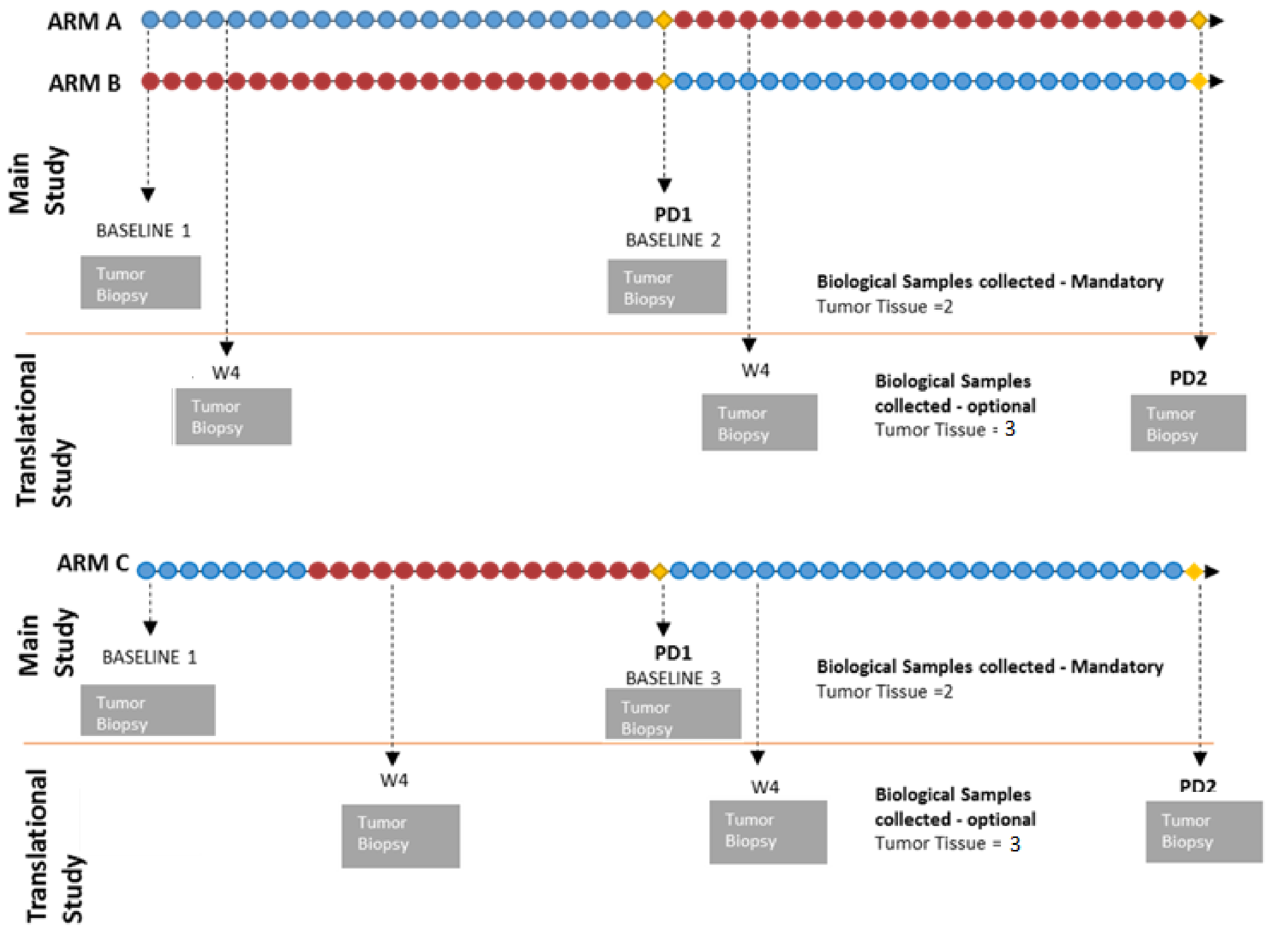

Note: Baseline tumor biopsies can be archival if collected after prior systemic therapy; otherwise a fresh biopsy will be collected

A minimum of 15 x tissue sections will be collected for each patient/time point.

New baseline biopsies will be performed in patients with easily accessible biopsy sites (subcutaneous and lymph nodes) and archived samples (from metastatic site) will be allowed for patients that cannot be biopsied. Procedures for sample collection and processing will be given to Investigators.

Biopsy on the progression site (if feasible) would be preferable. Otherwise biopsies from other metastatic disease site can be used. Sample will remain in the 10% Neutral Buffered Formalin for a minimum of 24-48 hours but no more than 96 hours. A matched paraffin embedded (FFPE) tissue block will be then stored ambient at the site until shipment.

Collection of the on-treatment sample should be encouraged, in order to obtain at least 30 (out of possible 138) matched pre- and on-treatment samples for Arm A/C and 30 (out of 69 patients) matched pre- and on-treatment

samples for Arm B. Path assessments will be regularly performed to ensure that collected samples are of good quality.

Peripheral blood samples:

In arm A, peripheral blood samples will be taken at baseline 1, Week 4 and PD 1 during Combo Target therapy; and at Week 4 and PD 2 during Combo Immuno therapy. Peripheral blood samples will be mandatory at baseline 1 and at PD1, and will be optional at the other time-points.

In arm B, peripheral blood samples will be taken at baseline 1, Week 4 and PD 1 during Combo Immuno therapy; and at Week 4 and PD 2 during Combo Target therapy. Peripheral blood samples will be mandatory at baseline 1 and at PD 1, and will be optional at the other time-points.

In arm C, peripheral blood samples will be taken at baseline 1, baseline 2, Week 4 and disease progression 1 (PD1) during Combo Immuno therapy; and at Week 4 and PD 2 during the 2nd Combo Target therapy. Peripheral blood samples will be mandatory at baseline 1, baseline 2 and at PD1 and will be optional at the other time-points.

Sample handling

The biomarker study requires blood sample collection at baseline, on-treatment (within first 4 weeks after first dose), and upon progression when feasible.

To assess the immunological biomarkers, in all arms the following peripheral blood samples must be collected:

A. 10 ml of non-anticoagulated whole blood for collection of serum (2 tubes x 5 ml tubes with red/yellow stopper);

B. 9 ml of whole blood in heparin for collection of plasma (2 x 4.5 ml tubes with green stopper);

C. 50 ml of whole blood in EDTA tubes for isolation of PBMC (5 x 10 ml tubes with lilac stopper);

D. 5 ml of whole blood in serum tubes for genetic test (1 tube x 5 ml with stopper);

The samples will be processed locally and then shipped in a central laboratory.
The details of sample processing will be provided separately to each site in a study sample manual.

Peripheral blood samples will be taken according to the following scheme :

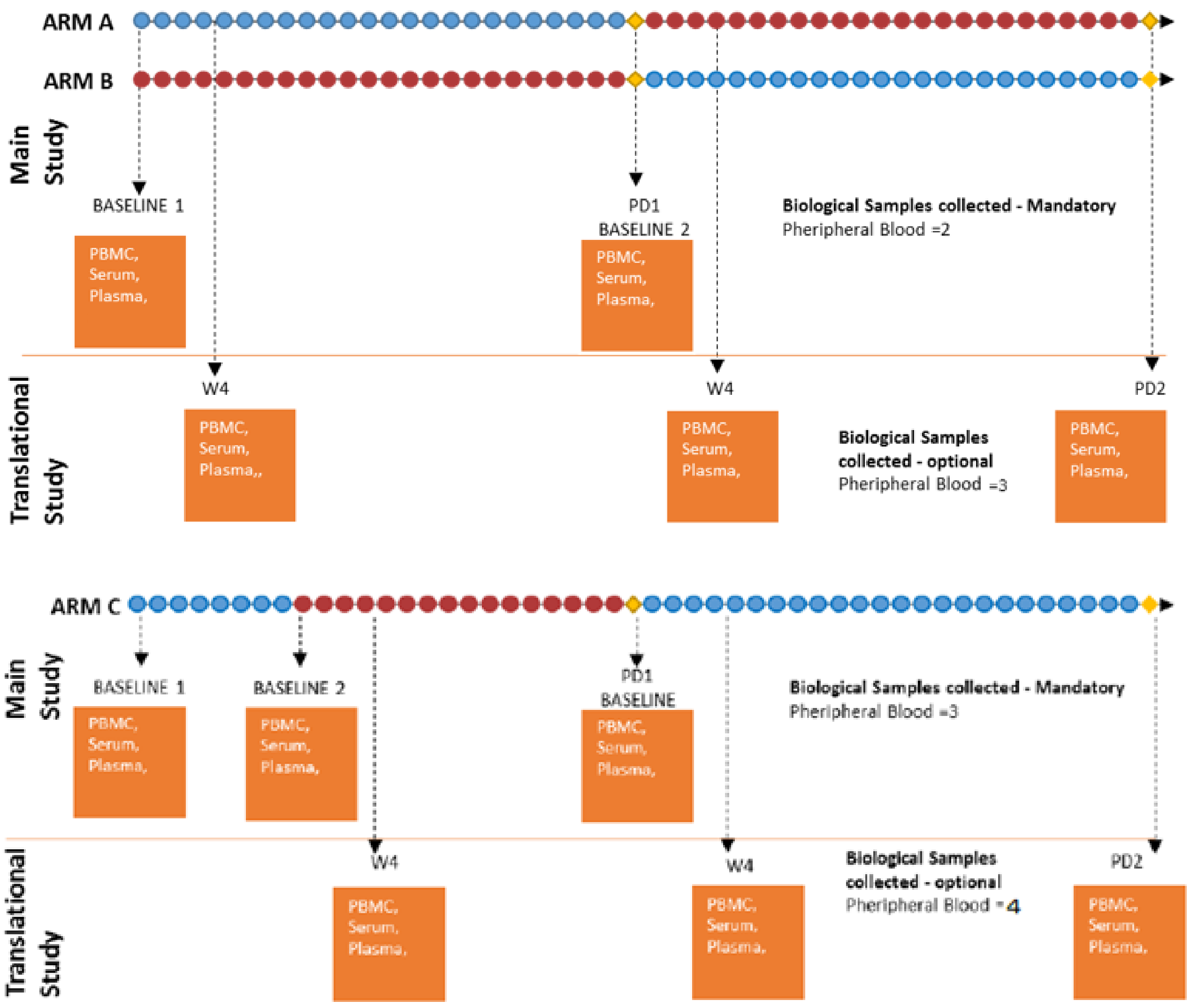

3.5.5 Efficacy Assessments and biomarkers study

Biopsies

Biopsies at baseline, on-treatment (within first 4 weeks after first dose), and upon progression when feasible, will be obtained for patients with accessible tumors upon patient’s consent to participate in the biomarker study.

In arm C, biopsy of tumoral lesions for the biological study will be performed at baseline 1, Week 4 and PD 1 during Combo Immuno therapy; and at Week 4 and PD 2 during the 2nd Combo Target therapy.

Accessible lesions are defined as tumor lesions which are easily biopsiable i.e. cutaneous, sub-cutaneous and palpable lymph nodes. Failure to obtain sufficient tumor sample, after making best efforts, will not be considered a protocol violation. Lesions with the biggest change in size, based on interval evaluation, are recommended to be excised at time of progressive disease. Whenever possible, biopsies at progression should be obtained within 3 days of study drug discontinuation. This may require prolonging the treatment a short time after tumor evaluation demonstrates progressive disease.

A minimum of 15 x tissue sections will be collected for each patient/time point. Biopsies will be immediately transferred into the provided vials, filled with formalin. The biopsies will be fixed for 24±2 hours, transferred into 70% ethanol and then (always in ethanol) shipped to a central pathology lab for paraffin embedding. In cases where a reasonable size biopsy (e.g., excisional biopsy, 5 mm punch, or more than a single 14 gauge core biopsy) could be collected with formalin fixation, every effort should also be made to collect a fresh frozen biopsy at disease progression. Optimally both specimens should be available when biopsy is of a reasonable size.

The following tumor tissue biomarkers will be measured:

- Immune status: CD3, CD8, CD4, CD45RO and CD11c T cells; Activated T cells; T regulatory cells; Dendritic cells;
- Resistance to immunotherapy agents: Checkpoint receptors/ligands; myeloid-derived suppressor cells (MDSCs);
- Resistance to targeted agents: aberrations in MEK/PI3K pathways; mRNA expression (nanostring method).

Gene panel:

Using the Ion Torrent technology, the following gene-panel options (which have been already successfully tested on DNA samples from FFPE tissues) can be used for the assessment of the alterations into the MAPK-PI3K pathways.

1. Hotspot regions in Ion Ampliseq Cancer Hotspot panel (~2,800 mutations of 50 oncogenes and tumor suppressor genes): 1 pool (10-15 ng per DNA sample)*

ABL1	*EGFR*	*GNAS*	*KRAS*	*PTPN11*
AKT1	*ERBB2*	*GNAQ*	*MET*	*RB1*
ALK	*ERBB4*	*HNF1A*	*MLH1*	*RET*
APC	*EZH2*	*HRAS*	*MPL*	*SMAD4*
ATM	*FBXW7*	*IDH1*	*NOTCH1*	*SMARCB1*
BRAF	*FGFR1*	*JAK2*	*NPM1*	*SMO*
CDH1	*FGFR2*	*JAK3*	*NRAS*	*SRC*
CDKN2A	*FGFR3*	*IDH2*	*PDGFRA*	*STK11*
CSF1R	*FLT3*	*KDR*	*PIK3CA*	*TP53*

CTNNB1	*GNA11*	*KIT*	*PTEN*	*VHL*

2. Exons within melanoma-associated genes from *Comprehensive Cancer Panel*: 3 pools (10-15 ng per pool, 30-45 ng per DNA sample)*

**GENE**	**Position**	**GENE**	**Position**
AKT3	chr1	HRAS	chr11
NRAS	chr1	ARID2	chr12
IDH1	chr2	CDK4	chr12
BAP1	chr3	KRAS	chr12
MITF	chr3	RB1	chr13
PIK3CA	chr3	AKT1	chr14
KDR	chr4	IDH2	chr15
KIT	chr4	MAP2K1	chr15
BRAF	chr7	ERBB2	chr17
MET	chr7	NF1	chr17
CDKN2A	chr9	TP53	chr17
GNAQ	chr9	AKT2	chr19
NOTCH1	chr9	GNA11	chr19
PPP6C	chr9	MAP2K2	chr19
PTEN	chr10	GNAS	chr20
CCND1	chr11	DDX3X	chrX

3. Exons within >400 oncogenes and tumor suppressor genes (*Comprehensive Cancer Panel*): 4 pools (10-15 ng per pool, 40-60 ng per DNA sample) *

**amount is referred to good quality DNA; thus, quantity of DNA from FFPE samples could be higher*

Study of peripheral blood biomarkers

Peripheral blood mononuclear cell (PBMC) samples will be collected for the biomarkers study.

The following peripheral blood biomarkers will be measured:

- Immune status/ “Resistance” to immunotherapy agents: Activated T cells; Memory/Exhausted T cells; T regulatory cells; MDSCs; Inflammatory response; CRP; TCR Sequencing/Gene Expression analysis;
- Response/Resistance to targeted agents: Apoptotic tumor cells (as measured by circulating tumor DNA); soluble hepatocyte growth factor (sHGF); serum vascular endothelial growth factor (sVEGF); soluble intercellular adhesion molecule-1 (sICAM-1); soluble interleukine-2 receptor (sCD25).

3.5.6 Tumor Response Criteria

Tumor evaluation will be assessed at screening/baseline (between Day -28 and -1) by means of CT or MRI of the brain, chest, abdomen and pelvis (C/A/P), every 8 weeks (+/- 1 week) for the first year and every 12 weeks (+/- 1 week) thereafter while the patient is on study, and at the end of treatment visit. MRI of brain at all baselines and as clinically indicated during treatment period. A window of +/-7 days of scheduled visit is allowed to complete tumor assessments at the required intervals.

Radiological tumor assessments of C/A/P will be done for measuring extent of disease. In addition, all patients must have brain CT and/or MRI to assess for brain metastasis at all baselines. Patients with known or suspected bone metastases should undergo radionuclide bone scan or PET scan at baseline and as per institutional standard of care thereafter.

All measurable and non-measurable lesions must be documented at screening (within 28 days prior to randomization) and re-assessed at each subsequent tumor evaluation (every 8 weeks (+/- 1 week) for the first year, every 12 weeks (+/- 1 week) while the patient is on study). Tumor assessments with CT or MRI scans of the chest, abdomen, and pelvis will be performed until disease progression after the second combo treatment. Imaging of the neck should be included if clinically indicated. In the event PET/CT scanner is used for tumor assessments, the CT portion of the PET/CT must meet criteria for diagnostic quality. All scans can be collected for a possible independent review.

In case of a single new lesion treated with surgery or radiotherapy, if all the other sites are under response patients should be treated beyond progression with the first therapy. For switching to the next treatment there should be measurable disease and the progression should be evident even in the other lesions.

In case of new brain metastasis (worsening of preexisting) assessed during the tumor evaluation, patients can be switched to the subsequent treatment, without waiting for stable disease, according to the schedule assessment.

As indicated in Section 4.6, patients with PD and evidence of unoperable brain metastases without the involvement of other sites will not undergo the biopsy and will continue treatment according the protocol arm.

For patients who discontinue study treatment for reasons other than investigator–determined disease progression, tumor assessments should continue to be performed as scheduled. Tumor responses will be assessed by the Investigator according to RECIST Criteria (version 1.1). Both measurable and non-measurable lesions will be assessed by the Investigator. For assessing response in patients with measurable disease, the preferred radiologic tumor response assessment is the CT scan with oral and i.v. contrast. If i.v. contrast is contraindicated, a non-contrast chest CT will be done with abdominal/pelvic contrast enhanced MRI. If contrast enhanced MRI is contraindicated then non-contrast MRI will suffice. CT/MRI scans of extremities may be done as appropriate in individual patients. PET scan, bone scan, and ultrasound, are not adequate for RECIST response assessment.

Patients should be assessed at designated time-points using a consistent imaging modality. The same method of assessment and the same technique should be used to characterize each identified and reported lesion at baseline and during follow-up. If more than one method of assessment is used at baseline, select the most accurate method according to RECIST when recording data; in addition, this method should again be performed in all subsequent evaluations. Tumor measurements should be made by the same Investigator/radiologist for each patient during the study to the extent that this is feasible. Objective responses by RECIST (Version 1.1) should be confirmed by repeated assessments at least 4 weeks after initial documentation of response. Clinical lesions will only be considered measurable when they are superficial and > 10 mm diameter as assessed using calipers (e.g. skin nodules). For skin lesions, documentation by color photography with ruler is required. CT scan is the preferred modality for skin lesions and should be used wherever possible.

3.5.7 Outcomes Research Assessments

Health-related Quality of Life

HRQoL will be evaluated by means of the 30-item European Organisation for Research and Treatment of Care quality of life questionnaire (EORTC QLQ-C30) (Appendix 4).

The EORTC QLQ-C30 (*Aaronson et al, 1993*) is the mostcommonly used QoL instrument in advanced melanoma clinical studies.

It is a 30-item instrument that has gained wide acceptance in oncology clinical studies. TheEORTC QLQ--C30 comprises six functional scales (physical functioning, cognitive functioning, emotional functioning, social functioning and global quality of life) as well as nine symptomscales (fatigue, pain, nausea/vomiting, dyspnea, insomnia, appetite loss, constipation, diarrhea, and financial difficulties). Except for the overall health status and global quality of life items, responses for all items are 4-point categorical scales ranging from 0 (Not at all) to 4 (Very much). The overall health status/quality of life responses are 7-point Likert scales.

General Health Status

General health status will be evaluated by means of the European Quality of Life 5-Dimensions (EQ-5D) (*Brooks, 1996*) (Appendix 5), that comprises 5 dimensions of health (mobility, self-care, usual activities, pain/discomfort, anxiety), each consisting of 3 levels (no, some/moderate and extreme problems), and a 0-100 mm visual analog scale (VAS) where 0 = worst imaginable health state and 100 = best imaginable health state. The utility data generated from the EQ-5D is recommended for and commonly used in cost effectiveness analysis.

Work Productivity and Activity Impairment

Impairment of work productivity and activity will be evaluated by means of the Work Productivity and Activity Impairment: General Health (WPAI:GH) questionnaire (*Reilly et al, 1993*) (Appendix 6).

This questionnaire is a 6-item questionnaire yielding four different types of scores. TheWPAI:GH was created as a patient-reported quantitative assessment of the amount ofabsenteeism (work time missed), presenteeism (impairment at work /reduced onthejobeffectiveness), work productivity (overall work impairment/absenteeism plus presenteeism) anddaily activity impairment attributable to general health. WPAI outcomes are expressed asimpairment percentages, with higher numbers indicating greater impairment and lessproductivity, i.e. worse outcomes.

3.5.8 Laboratory Assessments

Hematology and biochemistry will be done as part of regular safety assessments.

Specifically:

At Screening Visit

- Hematology (including hemoglobin, hematocrit, red blood cells, white blood cells, neutrophilis, lymphocytes, monocytes, eosinophhilis, basophilis, platelets count);
- Biochemistry (including glucose, sodium, potassium, calcium, chloride, bicarbonate, magnesium, urea, uric acid, albumin, creatinine, creatinine clearance, total protein, total bilirubin, indirect bilirubin, alkaline phosphatase, ALT, AST, Gamma glutamyltransferase, HDL, LDL, amylase, Lipase)
- Serum pregnancy test (within 24 hours prior to commencement of dosing) for women of child-bearing potential confirmed by serum HCG laboratory test;
- Coagulation (including INR, aPTT, PTT, Protrombin time, fibrogen)
- Urinalysis (including glucose, protein, blood, bilirubin, ketones, leukocytes esterase, UWBC/HPF; URBC/HPF, bacteria)
- Thyroid function test (including TSH, Free T4, Free T3)
- Endocrine Panel (including ACTH, total cortisol)
- Screening for HIV infection;
- Cardiac/Muscle Enzymes: Troponin, Creatine Kinase (CK). If total CK ≥3 X ULN, then measure isoenzymes and myoglobin in blood or urine weekly.

During the study

- Hematology (including hemoglobin, hematocrit, platelet count, WBC, ANC) at every visit;

- Biochemistry (including glucose, sodium, potassium, calcium, chloride, bicarbonate, magnesium, urea, uric acid, albumin, creatinine, creatinine clearance, total protein, total bilirubin, indirect bilirubin, alkaline phosphatase, ALT, AST, Gamma glutamyltransferase, HDL, LDL, amylase, Lipase)
- Coagulation (including INR, aPTT, PTT, Protrombin time, fibrogen)
- Thyroid function test (including TSH, Free T4, Free T3)
- Endocrine Panel (including ACTH, total cortisol)
- Cardiac/Muscle Enzymes: Troponin, Creatine Kinase (CK). If total CK ≥3 X ULN, then measure isoenzymes and myoglobin in blood or urine weekly;
- Urinalysis (only if clinically indicated);
- Serum Pregnancy Test in women of child-bearing potential every 6 (± 1) weeks during the treatment Combo-Immuno and every 4 (± 1) weeks during the treatment Combo-Targetand at end of study treatment and follow-up visit up to 31 weeks after the last dose of nivolumab/ipilimumab.

4. STUDY POPULATION

4.1 Inclusion Criteria

A subject is eligible for the study if all of the following criteria are met:

1) Patients of either sex aged ≥ 18 years;
2) Histologically confirmed stage III (unresectable) or stage IV melanoma with the BRAF V600 mutation. Patients with mucosal melanoma (but not those with ocular melanoma) are eligible for study participation;
3) Treatment naïve for metastatic disease patients. Previous adjuvant treatment, included checkpoint inhibitors anti CTLA-4, anti PD-1/PDL-1 is allowed except for stage IV (if completed at least 6 weeks prior to randomization, and all related adverse events have either returned to baseline or stabilized). BRAF inhibitor treatment in adjuvant setting is not permitted;
4) Measurable disease by computed tomography (CT) or Magnetic Resonance Imaging (MRI) per RECIST 1.1 criteria;
5) Presence of BRAF V600E or V600K mutation in tumor tissue prior to enrolment;
6) Eastern Cooperative Oncology Group (ECOG) performance status (PS) 0 or 1 (appendix 7);
7) Tumor tissue from an unresectable or metastatic site of disease must be provided for biomarker analyses. An archive sample is mandatory at the screening visit; however, a fresh sample would be preferable;
8) Female subjects of childbearing potential must have a negative pregnancy test result at baseline and must practice two highly effective methods of contraception for the total study duration plus 23 weeks (i.e. 30 days plus the time required for nivolumab to undergo five half lives) after the last dose of nivolumab and ipilimumab and 30 days after the last dose of binimetinib and encorafenib for female subjects. Additional pregnancy tests must be performed every 6 weeks during the Combo-Immuno and every 4 weeks during the Combo-Target, as well as at the end of the systemic exposure;
9) Men who are sexually active with women of childbearing potential must practice a reliable method of contraception for the total study duration plus 31 weeks (i.e. 80 days plus the time required for nivolumab to undergo five half lives) after the last dose of nivolumab and ipilimumab and 90 days after the last dose of binimetinib and encorafenib;
10) Adequate bone marrow haematological function: absolute neutrophil count (ANC) ≥ 1.5 x 10^9/L AND platelet count ≥ 100 x 10^9/L AND haemoglobin ≥ 9 g/dL;
11) Adequate liver function: total bilirubin ≤ 1.5 x upper limit of normal (ULN) AND aspartate aminotransferase (AST)/alanine aminotransferase (ALT) ≤ 2.5 X ULN (< 5 x ULN if liver metastases);
12) Adequate renal function: serum creatinine ≤ 1.5 mg/dL OR creatinine clearance ≥ 60 mL/min in males and ≥ 50 mL/min in females (calculated according to Cockroft-Gault formula);

13) Serum calcium levels, international normalised ratio (INR) and partial thromboplastin time were within normal limits;
14) Life expectancy of at least 3 months;
15) Ability to understand study-related patient information and provision of written informed consent for participation in the study;
16) Adequate electrolytes at Baseline, defined as serum potassium and magnesium levels within institutional normal limits (Note: replacement treatment to achieve adequate electrolytes will be allowed);
17) Adequate cardiac function:
    - left ventricular ejection fraction (LVEF) ≥ 50% as determined by a multigated --acquisition (MUGA) scan or echocardiogram;
    - QTc interval ≤ 480 ms (preferably the mean from triplicate ECGs).

4.2 Exclusion Criteria

A subject is excluded from the study if any of the following criteria are met:

1) Active brain metastases. Subjects with brain metastases are eligible if these have been treated and there is no magnetic resonance imaging (MRI) evidence of progression for at least 4 weeks after treatment is complete and within 28 days prior to first dose of study drug administration. There must also be no requirement for immunosuppressive doses of systemic corticosteroids (> 10 mg/day prednisone equivalents) for at least 2 weeks prior to study drug administration;
2) Subjects with active, known or suspected autoimmune disease;
3) Subjects with a condition requiring systemic treatment with either corticosteroids (>10 mg daily prednisone equivalents) or other immunosuppressive medications within 14 days of treatment;
4) Prior treatment for stage III (unresectable) or stage IV melanoma with an anti-Programmed Death receptor-1 (PD-1), anti-Programmed Death-1 ligand-1 (PD-L1), anti-PD-L2, or anti-cytotoxic T lymphocyte associated antigen-4 (anti-CTLA-4) antibody;
5) Female subjects who are pregnant (positive pregnancy test), breast-feeding, or who are of childbearing potential and not practicing a reliable method of birth control;
6) Evidence of severe or uncontrolled systemic disease or any concurrent condition which in the investigator’s opinion makes it undesirable for the patient to participate in the study, or which would jeopardize compliance with the protocol, or would interfere with the results of the study;
7) Patients with a history of uncontrolled cardiovascular or interstitial lung disease and evidence or risk of retinal vein occlusion or central serous retinopathy (Past or present evidence of rethinophaty central serous

retinopathy - CSR -, occlusion of retinal - RVOo retinal degenerative disease) or ophthalmopathy, which according to the ophthalmologic evaluation at baseline could be considered a risk factor for CSR / RVO (e.g., cupping of the optic disc, visual field defect, intraocular pressure - (eg: central IOP - > 21 mmHg);

8) Previous or concurrent malignancy. Exceptions: adequately treated basal cell or squamous cell skin cancer; in situ carcinoma of the cervix, treated curatively and without evidence of recurrence for at least 3 years prior to study entry; or other solid tumor treated curatively, and without evidence of recurrence for at least 3 years prior to study entry
9) History of Gilbert's syndrome;
10) Inability to regularly access centre facilities for logistical or other reasons;
11) History of poor co-operation, non-compliance with medical treatment, or unreliability;
12) Participation in any interventional drug or medical device study within 30 days prior to treatment start;
13) Positive test for human immunodeficiency virus (HIV), hepatitis B virus surface antigen (HBV sAg) or hepatitis C virus ribonucleic acid (HCV antibody) indicating acute or chronic infection;
14) Known history of testing positive for human immunodeficiency virus (HIV) or known acquired immunodeficiency syndrome (AIDS);
15) Receipt of live vaccine within 30 days prior to study drug administration;
16) History of severe or life-threatening skin adverse events or reactions to drugs.

4.2.1 Women of Childbearing Potential

A woman is considered to be of childbearing potential if she is postmenarcheal, has not reached a postmenopausal state (≥ 12 continuous months of amenorrhea with no identified cause other than menopause), and has not undergone surgical sterilization (removal of ovaries and/or uterus).

Examples of contraceptive methods with a failure rate of < 1% per year include bilateral tubal ligation, male sterilization, hormonal contraceptives that inhibit ovulation, hormone‒releasing intrauterine devices, and copper intrauterine devices. Hormonal contraceptive methods must be supplemented by a barrier method. The reliability of sexual abstinence should be evaluated in relation to the duration of the clinical trial and the preferred and usual lifestyle of the patient. Periodic abstinence (e.g., calendar, ovulation, symptothermal, or postovulation methods) and withdrawal are not acceptable methods of contraception

For men: agreement to remain abstinent (refrain from heterosexual intercourse) or use contraceptive measures, and agreement to refrain from donating sperm, as defined below: With female partners of childbearing potential, men must remain abstinent or use a condom plus an additional contraceptive method that together result in a failure rate of < 1% per year during the treatment period and for at least 6 months after the last dose

of study treatment. Men must refrain from donating sperm during this same period. With pregnant female partners, men must remain abstinent or use a condom during the treatment period and for 6 months after the last dose of study treatment to avoid exposing the embryo. The reliability of sexual abstinence should be evaluated in relation to the duration of the clinical trial and the preferred and usual lifestyle of the patient. Periodic abstinence (e.g., calendar, ovulation, symptothermal, or postovulation methods) and withdrawal are not acceptable methods of contraception.

4.3 Study treatments

Treatments information is given in the following table (Table 8).

<table>
<tr><th>Treatment Arm</th><th>Number of Pts Planned</th><th>Type of Study Drug</th><th>Compound</th><th>Minimum dose and unit</th><th>Frequency</th><th>Administration route</th></tr>
<tr><td rowspan="4">Arm A</td><td rowspan="4">69</td><td>Investigational</td><td>LGX818</td><td>450 mg</td><td>Daily</td><td>PO</td></tr>
<tr><td>Investigational</td><td>MEK162</td><td>90 mg</td><td>Daily</td><td>PO</td></tr>
<tr><td>Investigational</td><td>Ipilimumab</td><td>3 mg/kg</td><td>q3 weeks for 4 cycles</td><td>i.v.</td></tr>
<tr><td>Investigational</td><td>Nivolumab</td><td>1 mg/kg</td><td>q3 weeks for 4 cycles then q2 weeks 3 mg/kg or 240 mg q2 weeks or 480 mg q4 weeks</td><td>i.v.</td></tr>
<tr><td rowspan="4">Arm B</td><td rowspan="4">69</td><td>Investigational</td><td>LGX818</td><td>450 mg</td><td>Daily</td><td>PO</td></tr>
<tr><td>Investigational</td><td>MEK162</td><td>90 mg</td><td>Daily</td><td>PO</td></tr>
<tr><td>Investigational</td><td>Ipilimumab</td><td>3 mg/kg</td><td>q3 weeks for 4 cycles</td><td>i.v.</td></tr>
<tr><td>Investigational</td><td>Nivolumab</td><td>1 mg/kg</td><td>q3 weeks for 4 cycles then q2 weeks 3 mg/kg or 240 mg q2 weeks or 480 mg q4 weeks</td><td>i.v.</td></tr>
<tr><td rowspan="4">Arm C</td><td rowspan="4">69</td><td>Investigational</td><td>LGX818</td><td>450 mg</td><td>Daily</td><td>PO</td></tr>
<tr><td>Investigational</td><td>MEK162</td><td>90 mg</td><td>Daily</td><td>PO</td></tr>
<tr><td>Investigational</td><td>Ipilimumab</td><td>3 mg/kg</td><td>q3 weeks for 4 cycles</td><td>i.v.</td></tr>
<tr><td>Investigational</td><td>Nivolumab</td><td>1 mg/kg</td><td>q3 weeks for 4 cycles then q2 weeks 3 mg/kg or 240 mg q2 weeks or 480 mg q4 weeks</td><td>i.v.</td></tr>
<tr><td>TOTAL</td><td>207</td><td colspan="5"></td></tr>
</table>

Table 7: Treatments used in the study

4.4 Concomitant Medication and Treatment

The patient must notify the investigational site about any new medications he/she takes after the start of the study drug. All medications (other than study drug) and significant non-drug therapies (including physical

therapy, herbal/natural medications and blood transfusions) administered during the study must be listed on the Concomitant Medications or the Surgical and Medical Procedures eCRF.

Patients taking concomitant medications chronically should maintain the same dose and dose schedule throughout the study if medically feasible. On the days PK blood sampling is performed, the patient should continue their consistent use of other concomitant medications.

However, if a concomitant medication is used intermittently during the study, this medication should be avoided on these days, if medically feasible.

A single new lesion can be treated with surgery or radiotherapy, provided that all the other measurable lesions have not yet progressed.

Prohibited concomitant therapy

Anticancer therapies (including chemo- or biologic-therapy or radiation therapy, covering >30% of the red bone marrow reserve, and surgery) are prohibited while the patients are receiving study treatment. If such therapeutic measures are required for a patient then the patient must be discontinued from study treatment.

4.5 Drug Interaction

LGX818 is a reversible inhibitor of CYP2B6, CYP2C9, CYP3A4 and UGT1A1. It is also a time dependent inhibitor of CYP3A4. MEK162 is also a reversible inhibitor of CYP2B6.

Permitted medicatiaesons to be used with caution in this study include those that are sensitive substrates of CYP2B6, CYP2C9, CYP3A4, and UGT1A1 or those substrates that have a narrow therapeutic index (NTI).

There is a potential for MEK162 and LGX818 to induce CYP3A4 at concentrations >10-50 uM, which may reduce the effectiveness of hormonal contraception methods. Therefore, the use of at least one form of non-hormonal contraception will be needed during the participation in this study. Caution should be used in patients receiving concomitant treatment with other drugs that are substrates of CYP3A4 as the efficacy of these drugs could be reduced when administered with MEK162 and/or LGX818.

LGX818 has been identified to be primarily metabolized by CYP3A4 *in vitro*. It is advised that LGX818 should be taken with caution when co-administered with strong inhibitors of CYP3A4.

MEK162 has been identified to be primarily metabolized by UGT1A1 *in vitro*. It is advised that inhibitors and inducers of UGT1A1 should be taken with caution.

In vitro data showed that both MEK162 and LGX818 are substrates of P-gp. MEK162 is also a substrate of breast cancer resistant protein (BCRP). Thus, the use of drugs that are known to inhibit or induce P-gp and

BCRP should be used with caution. LGX818 is a BCRP inhibitor. It is also a potent inhibitorof the renal transporters OAT1, OAT3 and OCT2 and the hepatic transporters OATP1B1 and OATP1B3. Therefore, the co-administration of drugs that are known to be sensitive or NTI substrate of BCRP, OAT1, OAT3, OCT2, OATP1B1 and OATP1B3 should be used with caution.

The solubility of MEK162 and LGX818 is pH dependent and a 10-fold decrease in solubility is observed between pH 1 and 2. Patients receiving concomitant treatments that could potentially modify the gastric pH (i.e. PPI) should be instructed to take them at least two hours after the administration of MEK162.

Drugs with a conditional, possible, or known risk to induce Torsade de Pointes (TdP) should be used with caution. Patients receiving such medications must be carefully monitored for potentiating of toxicity due to any individual concomitant medication and may require dose titration of the drug substance. Investigators should use caution when prescribing comedications, as clinical experience with these compounds in patients with cancer is often limited. Investigators should contact the Sponsor when they are unsure whether a drug should be prescribed to a patient in the clinical trial.

As PD-1 blockers modify the immune system in a manner not fully elucidated, use of live vaccines is prohibited during the study, and for three months after last dose of PD-1 blocker (nivolumab).

4.6 Criteria for Premature Withdrawal

Patients have the right to withdraw from the treatment or from the study at any time and irrespective of the reason.

In addition, the Investigator may discontinue a participant, without their consent, from the trial at any time if the Investigator considers it necessary for any reason including:

- Pregnancy
- Ineligibility (either arising during the trial or retrospectively having been overlooked at screening)
- Significant protocol deviation
- Significant non-compliance with treatment regimen or trial requirements
- An adverse event which requires discontinuation of the trial medication or results in inability to continue to comply with trial procedures
- Any other condition which requires discontinuation of the trial medication or results in inability to continue to comply with trial procedures
- Withdrawal of Consent
- Loss to follow up

- After 2 years of nivolumab/ipilimumab dosing, to reduce the risk of long-term toxicity.

Patients who discontinue from the study will be asked to return to the clinic within 28 days of the last dose of IMP for the follow-up visit and to be contacted every 12 weeks until 60 months from randomization for long term survival follow-up.

For patients who discontinue study treatment due to an Adverse Event there are two possible scenarios:

The AE occurs before progression of disease (e.g. for patients in ARM A the combo target treatment is discontinued due to AE): the patient is followed until progression of disease, as per protocol -CT scan every 8 weeks (+/- 1 week) from day 1 during the first year and every 12 weeks (+/- 1 week) thereafter-. When progression of disease is observed, the patient can resume treatment by switching to the following treatment combination required by the protocol (e.g. patients in ARM A will be treated with combo immuno). Besides tumour assessments, only relevant exams to monitor the AE should be performed.

The AE occurs after progression of disease has been observed (e.g. for patients in ARM A the immuno treatment is discontinued due to AE): the patient is followed until the second progression of disease, as per protocol -CT scan every 8 weeks (+/- 1 week) from day 1 during the first year and every 12 weeks (+/- 1 week) thereafter-. When progression of disease is observed the patient is permanently discontinued from any study treatments. Besides tumour assessments, only relevant exams to monitor the AE should be performed.

In case of permanent discontinuation from study treatments, a safety follow-up within 28 days from last dose is required. If lost to follow-up, the Investigator should make every effort to contact the patient by telephone or by sending a registered letter to establish as completely as possible the reason for the withdrawal. A complete final evaluation at the time of the patient’s withdrawal should be made with an explanation of why the patient is withdrawing from the study.

Any administrative or other reasons for withdrawal must be documented and explained to the patient.

If the reason for removal of a patient from the study is an AE, the principal specific event will be recorded on the eCRF. The patient should be followed until the AE has resolved, if possible.

Patients with PD and evidence of unoperable brain metastases without the involvement of other sites will not undergo the biopsy and will continue treatment according to protocol arm.

4.7 Definition of End of Trial

The end of trial is the date of the last visit of the last participant. Last visit will occur 60 months after randomization.

5. INVESTIGATIONAL MEDICINAL PRODUCTS

The investigational drugs are LGX818 and MEK162 given in combination (Combo Target) and nivolumab 1 mg/kg solution intravenously combined with ipilimumab 3 mg/kg solution intravenously every 3 weeks for 4 doses then nivolumab 3 mg/kg solution intravenously every 2 weeks or 240 mg every 2 weeks or 480 mg every 4 weeks (Combo Immuno).

5.1 Combo Target

5.1.1 Recommended Dose

Combo Target (LGX818 450 mg p.o. od + MEK162 45 mg p.o. bid) will be given until PD in arm A, and will be given for 8 weeks and then following PD 1 after Combo Immuno until PD 2 in Arm C.

LGX818 and MEK162 will be administered orally on a daily schedule as a fixed combination dose, and not by body weight or body surface area (Table 9).

Study treatments	Pharmaceutical form and route of administration	Single Dose	Frequency	Total Daily Dose
LGX818[a]	Capsules for oral use	450 mg	Once daily	450 mg
MEK162[b]	Tablets for oral use	45 mg	Twice daily	90mg

[a]LGX818 will be provided as 75 mg and 50 mg capsules
[b]MEK162 will be provided as 15 mg tablets

Table 8: Combo Target dose and treatment schedule

Patients will be supplied with a sufficient number of tablets and/or capsules for the number of doses to be taken prior to the next scheduled visit. In addition, patients will be provided with a dosing diary on which they should record prescribed dose, and whether it was taken or not.

If a patient vomits at any time after dosing, the dose of study drug should not be re-administered. Doses of MEK162 that are omitted for AEs or any other reason should not be made up later in the day, or at the end of the dosing period. Doses of LGX818 that are omitted for AEs or any other reason can be taken up to 12 hours prior to the next dose.

Patients must avoid consumption of grapefruit or grapefruit juice during the entire study and preferably 7 days before the first dose of study medications, due to potential CYP3A4 interaction with the study medications. Orange juice is allowed.

Complete dosing instructions will be provided to study patients and will include the minimum times between doses and instructions for missed doses. Patients will also be instructed not to chew, crush, or dissolve tablets

and/or capsules of study drugs. The investigator or responsible site personnel should instruct the patient to take the study drugs as per protocol (promote compliance). All dosages prescribed and dispensed to the patient and all dose changes and all missed doses during the study must be recorded in the eCRF.

Drug accountability must be performed on a regular basis. Patients will be instructed to return unused study drugs to the site at the next visit. The site personnel will ensure that the appropriate dose of each study drug is administered at each visit and will provide the patient with the correct quantity of drugs for subsequent dosing.

5.1.2 Dose Modifications, Interruption and delays criteria for Combo Target

Patients will be monitored for adverse events at each visit with the NCI CTCAE version 4.03 used for all grading.

For patients who do not tolerate LGX818 and/or MEK162 initial dosing schedule, dose adjustment is permitted in order to allow the patient to continue on study drug (see Table 10). A dose reduction below 50 mg bid for LGX818 and below 15 mg od for MEK162 is not allowed. Dose interruptions of more than 28 consecutive days are not allowed.

Dose level	LGX818	MEK162
0 (starting dose)	450 mg od	45 mg bid
-1	300 mg od	30 mg bid
-2	200 mg od	15 mg bid
-3	100 mg od	-
-4	50 mg od	-

Dose reduction should be based on the highest AE grade

Table 9: Dose reduction for LGX818 and MEK162

Doses of MEK162 that are omitted for AEs or any other reason should not be made up later in the day, or at the end of the dosing period. Doses of LGX818 that are omitted for AEs or any other reason can be taken up to 12 hours prior to the next dose. For both LGX818 and MEK162, when the toxicity that resulted in a dose reduction improves to Grade 1 or less, the dose can be re-escalated at the investigators discretion provided there are no other concomitant toxicities.

If MEK162 is dose reduced due to left ventricular dysfunction, no dose re-escalation is allowed.

All dosing interruptions and changes must be recorded in the eCRF.

In case of adverse events or other reasons, the patient restarts the treatment from the current day (day corresponding to the current treatment cycle day) and not from the last day. Dose reduction/interruption/discontinuation decisions should be based on the CTCAE grade of the toxicity and the guidelines provided below (Table 11). All dose modifications should be based on the worst preceding toxicity (CTCAE version 4.03). In general, doses should not be reduced or interrupted for Grade 1 toxicities, but

treatment to control symptoms should be provided as appropriate. All AEs should be followed weekly or as clinically appropriate until stabilization or resolution. If a patient on the Combo Target therapy discontinues treatment with MEK162, the patient may continue treatment with LGX818. However, due to the limited efficacy of MEK162 alone in the study population, if a patient on the Combo Target therapy discontinues treatment with LGX818, he/she must discontinue treatment with MEK162, complete the end of treatment visit (only if in ARM B and C after first PD) and continue to be followed until disease progression.

Please refer to Table 11 for dose adjustment recommendations for LGX818 and/or MEK162 induced toxicities. Please refer to Appendix 1 and Appendix 2 for additional supportive care guidelines for the management of LGX818/MEK162 and MEK162-induced skin toxicity and diarrhoea respectively.

Recommended Dose Modifications for LGX818/MEK162 combination	
**Worst Toxicity CTCAE v4.03 Grade (unless otherwise specified)[a]**	**Recommended Dose Modifications any time during a cycle of therapy**
No toxicity	Maintain dose level
**Blood and lymphatic system disorder**	
Febrile neutropenia<br>(ANC < 1.0 x 10^9/L, fever ≥ 38.5°C)[b]	Omit dose until resolved, then ↓ 1 dose level of LGX818 and MEK162
**Investigations (blood)**	
Neutropenia (neutrophil count (ANC) decreased)	
Grade 1 (ANC < LLN - 1.5 x 10^9/L) or<br>Grade 2 (ANC < 1.5 - 1.0 x 10^9/L)	Maintain dose level of LGX818 and MEK162
Grade 3 (ANC < 1.0 - 0.5 x 10^9/L)	Omit dose of LGX818 and MEK162 until resolved to ≤ Grade 2, then:<br>- If resolved in ≤ 7 days, maintain dose level of LGX818 and MEK162<br>- If resolved in > 7 days, then ↓ 1 dose level* of LGX818 and maintain dose level of MEK162
Grade 4 (ANC < 0.5 x 10^9/L)	Omit dose of LGX818 and MEK162 until resolved to ≤ Grade 2, then ↓ 1 dose level* of LGX818 and MEK162
Thrombocytopenia (platelet count decreased)	
Grade 1 (PLT < LLN - 75 x 10^9/L) or<br>Grade 2 (PLT <75 - 50 x 10^9/L)	Maintain dose level of LGX818 and MEK162
Grade 3 (PLT < 50-25 x 10^9/L)	Omit dose of LGX818 and MEK162 until resolved to ≤ Grade 1, then:<br>- If resolved in ≤ 7 days, maintain dose level of LGX818 and MEK162.<br>- If resolved in > 7 days and/or with signs of bleeding, then ↓ 1 dose level* of LGX818 and MEK162
Grade 4 (PLT < 25 x 10^9/L)	Omit dose of LGX818 and MEK162 and discontinue patient from study drug treatment.
**Gastrointestinal disorders**	
Diarrhea	
Grade 1	Maintain dose level of LGX818 and MEK162, but initiate anti-diarrhea treatment (see Appendix 3).
Grade 2	Omit dose of LGX818 and MEK162 until resolved to Grade ≤ 1 and then maintain dose level of LGX818 and MEK162<br>- For 2nd occurrence of diarrhea Grade 2 within 15 days, omit dose of LGX818 and MEK162 until resolved to Grade ≤ 1, then reduce MEK162 by 1 dose level* and maintain dose level of LGX818
Grade 3	Omit dose of LGX818 and MEK162 until resolved to Grade ≤ 1, then reduce dose of LGX818 and MEK162 by 1 dose level*.

Grade 4	Omit dose of LGX818 and MEK162 and discontinue patient from study drug treatment.<br>Note: Anti-diarrhea medication is recommended at the first sign of abdominal cramping, loose stools or overt diarrhea.
Nausea/Vomiting	
Grade 1 or 2	Maintain dose level of LGX818 and MEK162
Grade 3	Omit dose of LGX818 and MEK162 until resolved to Grade ≤ 1, then ↓ 1 dose level* of LGX818 and MEK162
Grade 4	Omit dose of LGX818 and MEK162 and discontinue patient from study drug treatment.<br>Note: Omit dose for ≥ grade 3 vomiting or nausea only if the vomiting or nausea cannot be controlled with optimal antiemetics (as per local practice).
Pancreatitis	
Grade 1 or 2	Maintain dose level of LGX818 and MEK162
Grade ≥ 3	Omit dose of LGX818 and MEK162 and discontinue patient from study drug treatment
**Investigations (Renal)**	
Serum creatinine	
Grade 1 (> ULN – 1.5 x ULN)	Maintain dose level of LGX818 and MEK162
Grade 2 (> 1.5 - 3.0 x ULN)	Omit dose of LGX818 and MEK162 until resolved to ≤ Grade 1, then maintain dose level of LGX818 and MEK162
Grade ≥3 (> 3.0 x ULN)	Omit dose of LGX818 and MEK162 and discontinue patient from study treatment.
**Investigations (Hepatic)**	
Bilirubin	
Grade 1 (> ULN – 1.5 x ULN)	Maintain dose level of LGX818 and MEK162
Grade 2 (> 1.5 – 3.0 x ULN)	Omit dose of LGX818 and MEK162 until resolved to Grade ≤ 1, then:<br>- If resolved in ≤ 7 days, maintain dose level of LGX818 and MEK162<br>- If resolved in > 7 days, ↓ 1 dose level* of LGX818 and MEK162

Grade ≥ 3 (> 3.0 x ULN)	Omit dose of LGX818 and MEK162 and discontinue patient from study drug treatment.<br>Note: If Grade 3 or 4 hyperbilirubinemia is due to the indirect (non-conjugated) component only, and hemolysis as the etiology has been ruled out as per institutional guidelines (e.g., review of peripheral blood smear and haptoglobin determination), then ↓ 1 dose level* and continue treatment at the discretion of the investigator.
AST or ALT	
Grade 1	Maintain dose level of LGX818 and MEK162
Grade 2 or Grade 3	Omit dose of LGX818 and MEK162 until resolved to Grade ≤ 1 (or Grade ≤ 2 in case of liver metastasis), then<br>- If resolved in ≤ 7 days, maintain dose level of LGX818 and MEK162<br>- If resolved in > 7 days, ↓ 1 dose level* of LGX818 and MEK162
Grade 4	Omit dose of LGX818 and MEK162 and discontinue patient from study drug treatment.
AST or ALT and Bilirubin	
AST or ALT > 3.0 - 5.0 x ULN and total blood bilirubin ≥ Grade 2	Omit dose of LGX818 and MEK162 until resolved to Grade ≤ 1, then<br>- If resolved in ≤ 7 days, ↓ 1 dose level* of LGX818 and MEK162<br>- If resolved in > 7 days, discontinue patient from study drug treatment.
AST or ALT > 5.0 x ULN and total blood bilirubin ≥Grade 2	Omit dose of LGX818 and MEK162 and discontinue patient from study drug treatment.
**Investigations (Metabolic)**	
Asymptomatic amylase and/or lipase elevation	
Grade 1 (> ULN - 1.5 x ULN) or<br>Grade 2 (> 1.5 - 2.0 x ULN)	Maintain dose level of LGX818 and MEK162
Grade 3 (> 2.0 - 5.0 x ULN)	Omit dose of LGX818 and MEK162 until resolved to Grade ≤ 2, then:<br>- If resolved in ≤ 7 days, maintain dose level of LGX818 and MEK162<br>- If resolved in<br>> 7 days, ↓ 1 dose level* of LGX818 and MEK162
Grade 4 (> 5.0 x ULN)	Omit dose of LGX818 and MEK162 and discontinue patient from study drug treatment.<br>Note: A CT scan or other imaging study to assess the pancreas, liver, and gallbladder must be performed within 1 week of the first occurrence of any grade ≥ 3 of amylase and/or lipase. If asymptomatic Grade 2 elevations of lipase and/or amylase

	occur again at the reduced dose, patients will be discontinued permanently from study treatment.
**Cardiac disorders**	
Cardiac general	
Grade 1 or 2	Maintain dose level of LGX818 and MEK162
Grade 3	Omit dose of LGX818 and MEK162, until resolved to Grade ≤ 1, then ↓ 1 dose level* of LGX818 and MEK162
Grade 4	Omit dose of LGX818 and MEK162 and discontinue patient from study drug treatment.
Creatine phosphokinase (CPK)	
Grade 1 (> ULN – 2.5 x ULN) or<br>Grade 2 (> 2.5 - 5.0 x ULN)	Maintain dose level of LGX818 and MEK162
Grade 3 (> 5.0 - 10.0 x ULN)	If asymptomatic: Maintain dose level of LGX818 and MEK162<br>If symptomatic: Omit dose of MEK162 and maintain dose of LGX818 until resolved to Grade ≤ 1, then:<br>- If resolved in ≤ 28 days, then ↓ 1 dose level* of MEK 162 and maintain dose level of LGX8182<br>- If resolved in > 28 days, then discontinue patient from study drug treatment with LGX818 and MEK162
Grade 4 (> 10.0 x ULN)	Omit dose of MEK162 and maintain dose of LGX818 until resolved to CTCAE Grade ≤ 1, then:<br>- If resolved in ≤ 28 days, then ↓ 1 dose level of MEK162 and maintain dose level of LGX818<br>- If resolved in > 28 days, then discontinue patient from study drug treatment with LGX818 and MEK162
If CPK increase measure serum creatinine to assess for renal impairment.<br>Rhabdomyolysis definition according this protocol:<br>• Muscle symptoms (typically muscle pain, weakness in the literature; specific PTs to be defined per cited current MedDRA version for the CSPD)<br>• CK >10X ULN (CTCAE Grade 4) or CK >10,000 IU/L<br>• hospitalization/medical intervention with i.v. hydration	
**Cardiac Investigation – Prolongation of the QT interval QTcF value**	
QTcF > 500 ms during treatment and change from pre-treatment value remains ≤ 60 ms	Patients should have regular and appropriate (continuous) ECG monitoring in hospital until an adequately trained physician (such as a cardiologist or internist) has reviewed the data. Electrolyte abnormalities including magnesium should be corrected and cardiac risk factors for QT prolongation (e.g., congestive heart failure, bradyarrhythmias) should be controlled.<br>1st or 2nd occurrence:<br>• Temporarily interrupt dosing of encorafenib and binimetinib treatment until QTcF ≤480 ms. Then resume treatment at 1 reduced dose levelb of encorafenib and binimetinib. If a patient restarts binimetinib and

	encorafenib following resolution of Grade 3 QTcF prolongation event, the patient should be evaluated with triplicate predose ECGs on Day 1 of the next cycle, followed by a single postdose ECG and a single predose ECG on Day 15, as well as triplicate predose ECGs and a single postdose ECG on Days 1 and 2 of the subsequent cycle (2nd cycle after the Grade 3 QT prolongation event).<br>3rd occurrence:<br>• Permanently discontinue encorafenib and binimetinib
QTcF increase during treatment is both > 500 ms and > 60 ms change from pre treatment values	Patients should have regular and appropriate (continuous) ECG monitoring in hospital until an adequately trained physician (such as a cardiologist or internist) has reviewed the data. Electrolyte abnormalities including magnesium should be corrected and cardiac risk factors for QT prolongation (e.g., congestive heart failure, bradyarrhythmias) should be controlled.<br>• Permanently discontinue encorafenib and binimetinib
**LV systolic dysfunction (not according CTCAE)**	
Asymptomatic decrease of > 10% in LVEF compared to baseline and the ejection fraction is below the institution's lower limit of normal and CTCAE Grade 2	Omit dose of MEK162 until LVEF recovers (defined as ≥ LLN and decrease ≤ 10% compared to baseline).<br>- If the LVEF recovers ≤ 21 days, then ↓ 1 dose level of MEK162, maintain dose of LGX818 and monitor LVEF 2 weeks after restarting on MEK162, every 4 weeks for 12 weeks and subsequently as per protocol<br>- If the LVEF recovers >21 days, then discontinue patient from study drug treatment with MEK162 and LGX818, and closely monitor LVEF until resolution (or for 16 weeks).
Grade ≥ 3	Omit dose of MEK162 and LGX818 and discontinue patient from study drug treatment.
**Vascular disorders**	
Hypertension	
Grade 1 or 2	Maintain dose level of LGX818 and MEK162
Grade 3 (requiring more than one drug or more intensive therapy than previously)	Omit dose of LGX818 and MEK162, until resolved to Grade ≤ 1, then ↓ 1 dose level* of LGX818 and MEK162
Grade 4 (life-threatening)	Omit dose of LGX818 and MEK162, and discontinue patient from study drug treatment
**Eye disorders**	
Eye disorders – RVO[e]	Note: Results of ophthalmic examinations must be made available upon request. This includes scans/images of fluorescein angiography.
any Grade	Omit dose of LGX818 and MEK162 and discontinue patient from study drug treatment[c]
Eye disorders – Retinal events, Uveitis[e]	Note: Results and images of ophthalmic examinations must be made available upon request. This includes

	scans/images of OCTs.
Grade 1	Maintain dose of LGX818 and MEK162 and increase frequency of ophthalmic monitoring by ophthalmologist to at least every 14 days
Grade 2	Maintain dose of LGX818 and MEK162 and refer the patient to ophthalmologist within one week. Reassess the patient weekly (ophthalmic examination) until resolution to Grade ≤ 1:<br>- If resolved to Grade ≤ 1 in ≤ 21 days, maintain dose of LGX818 and MEK162<br>- If not resolved to Grade ≤ 1 in ≤ 21 days, reduce 1 dose level[d] of LGX818 and MEK162<br>At any time if symptoms worsen, or persist with the same severity for more than 7 days,<br>reduce 1 dose leveld of LGX818 and MEK162
Grade 3	Omit dose of LGX818 and MEK162 and refer the patient to ophthalmologist monitoring within one week[e]:<br>- If resolved to Grade ≤ 1 in ≤ 21 days, reduce 1 dose level[b] of LGX818 and MEK162<br>- If not resolved to Grade ≤ 1 in ≤ 21 days, permanently discontinue LGX818 and MEK162, and refer the patient to ophthalmologist monitoring
Grade 4	Permanently discontinue LGX818 and MEK162, and refer the patient to ophthalmologist monitoring[e]
Eye disorders – any other (i.e. retinal detachment)	
Grade 1 or 2	Maintain dose level of LGX818 and MEK162 and increase frequency of ophthalmic monitoring to at least every 14 days. At any time if symptoms worsen, or persist with the same severity for more than 7 days, reduce 1 dose level[d] of LGX818 and MEK162
Grade 3	Omit dose of LGX818 and MEK162 and refer patient to ophthalmologist monitoring within one week[e]:<br>- If resolved to Grade ≤ 1 in ≤ 21 days, reduce 1 dose level[d] of LGX818 and MEK162<br>- If not resolved to Grade ≤ 1 in ≤ 21 days, permanently discontinue LGX818 and MEK162,<br>and refer the patient to ophthalmologist monitoring[e]
Grade 4	Permanently discontinue LGX818 and MEK162, and refer the patient to ophthalmologist Monitoring[e]
**Skin and subcutaneous tissue disorders**	
Rash/ HFSR/ photosensitivity	
Grade 1	Maintain dose level of LGX818 and MEK162, but consider initiating appropriate skin toxicity therapy (see Appendix 3)
Grade 2	Maintain dose level of LGX818 and MEK162, but initiate/intensify appropriate skin toxicity therapy

Grade 3, despite skin toxicity therapy	Omit dose of LGX818 and MEK162, until resolved to Grade ≤ 1 then:<br>- If resolved in ≤ 7 days, ↓ 1 dose level* of LGX818 and MEK162<br>- If resolved in > 7 days, discontinue patient from study drug treatment with LGX818 and MEK162
Grade 4, despite skin toxicity therapy	Omit dose LGX818 and MEK162, and discontinue patient from study drug treatment with LGX818 and MEK162
**General disorders and administration site conditions**	
Fatigue	
Grade 1 or 2	Maintain dose level of LGX818 and MEK162
Grade 3	Omit dose of LGX818 and MEK162, until resolved to Grade ≤ 1, then:<br>- If resolved in ≤ 7 days, maintain dose level of LGX818 and MEK162<br>- If resolved in > 7 days, ↓ 1 dose level* of LGX818 and MEK162
Edema	
Grade 1 or 2	Maintain dose level of LGX818 and MEK162
Grade 3	Omit dose of LGX818 and MEK162 until resolved to Grade ≤ 1, then:<br>- If resolved in ≤ 14 days, ↓ 1 dose level* of MEK162 and maintain dose of LGX818<br>- If resolved in > 14 days, discontinue patient from study drug treatment with LGX818 and MEK162
**Other adverse events**[c]	
Grade 1 or 2	Maintain dose level of LGX818 and MEK162
Grade 3	Omit dose of LGX818 and MEK162, until resolved to Grade ≤ 1, then ↓ 1 dose level* of LGX818 and MEK162
Grade 4	Omit dose of LGX818 and MEK162 and discontinue patient from study drug treatment
[a] All dose modifications should be based on the worst preceding toxicity.<br>[b] Not CTCAE grading<br>[c] except: 1) lymphopenia unless clinically significant, 2) occurrence of KA and/or cutaneous SCC, 3) alkaline phosphatase, 4) AEs not considered clinically significant like alopecia.<br>[d] Dose reduction below 50 mg QD for LGX818, and below 15 mg BID for MEK162 is not allowed<br>[e] Ophthalmic monitoring mandated for retinal event, posterior uveitis, RVO: further evaluation with specialized retinal imaging (e.g. ocular coherence tomography, angiography)<br>* ↓ 1 dose level refers to: next lower dose level of LGX818, MEK162	

Table 10: Recommended dose modifications associated with treatment-related adverse events

Patients whose treatment is interrupted or permanently discontinued due to an adverse event or clinically significant laboratory value, must be followed up at least once a week (or more frequently if required by

institutional practices, or if clinically indicated) for 4 weeks, and subsequently at approximately 4-week intervals, until resolution or stabilization of the event, whichever comes first.

Appropriate clinical experts such as an ophthalmologist, cardiologist or dermatologist should be consulted as deemed necessary.

Patients who discontinued permanently the LGX818/MEK162 treatment can be followed up until progression disease and remain in the SECOMBIT study unless in the judgment of the Investigator, presents a substantial clinical risk to the subject with the study treatments.

5.1.3 Formulation, Packaging and Labelling of Combo Target

Study drug packaging will bear a label with the identification required by local law, the protocol number, drug identification and dosage (Table 12).

Medication labels (LGX818, MEK162) will be in the local language and comply with the legal requirements of each country in which the study will be conducted. They will include storage conditions for the drugs, and a unique medication number

Study treatments	Packaging	Labelling (and dosing frequency)
MEK162	Tablets in bottles	Labelled as “MEK162” (BID)<br>Study treatment packaging has a label containing a write-in space for the patient number which will be hand-written onto the label by the responsible site personnel.<br>A unique medication number is printed on this label.
LGX818	Capsules in bottles	Labelled as “LGX818” (OD)<br>Study treatment packaging has a label containing a write-in space for the patient number which will be hand-written onto the label by the responsible site personnel.<br>A unique medication number is printed on each part of this label.

Table 11: Packaging and labelling

<u>LGX818</u>

LGX818 75mg and 50 mg will be provided as capsules and packaged per strength into bottle.

LGX818 50 mg will be provided in small quantities to support dose reductions (dose level -2, -3, -4).

Each bottle will be labelled at a minimum with a unique identifier (medication number), the lot number, contents (number of capsules), dosage strength and storage conditions.

<u>MEK162</u>

MEK162 15 mg will be provided as film-coated tablets and packaged into high-density polyethylene bottles. Each bottle will be labelled at a minimum with a unique identifier (medication number), contents (number of tablets), dosage strength, storage conditions.

5.2 Combo Immuno

5.2.1 Recommended Dose

Combo Immuno (nivolumab 1 mg/kg solution (i.v.) combined with ipilimumab 3 mg/kg solution (i.v.) every 3 weeks for 4 doses then nivolumab 3 mg/kg solution (i.v.) every 2 weeks or 240 mg every 2 weeks or 480 mg every 4 weeks) will be given until PD in arm B and C will be given following PD after Combo Target in arm A.

Nivolumab and ipilimumamb will be administered i.v., as reported in Table 13.

Study treatments	Pharmaceutical form and route of administration	Single Dose	Frequency
Nivolumab	Solution for infusion	1 mg/kg	Every 3 weeks for 4 doses in combination with ipilimumab than every two weeks or 240 mg every 2 weeks or 480 mg every 4 weeks until PD as monotherapy
Ipilimumamb	Solution for infusion	3 mg/kg	Every 3 weeks for 4 doses in combination with nivolumab

Table 12: Combo Immuno dose and treatment schedule

The dosing calculations should be based on the body weight. If the subject's weight on the day of dosing differs by > 10% from the weight used to calculate the dose, the dose must be recalculated. All doses should be rounded to the nearest milligram. There will be no dose modifications allowed.

The first dose is to be administered within 3 days following randomization. Nivolumab is to be administered first. The second infusion will always be the ipilimumab study drug and will start not sooner than 30 minutes after completion of the nivolumab infusion. Separate infusion bags and filters for nivolumab and ipilimumab must be used for each infusion. Subjects may be dosed no less than 12 days from the previous dose.

Premedications or medications used to treat infusion-related reactions should be sourced by the investigative sites if available and permitted by local regulations.

Antiemetic premedications should not be routinely administered prior to dosing of drugs. See below for premedication recommendations following a nivolumab or ipilimumab related infusion reaction.

Nivolumab

Nivolumab is to be administered as a 60-minute i.v. infusion, using a volumetric pump with a 0.2/0.22 micron in-line filter at the protocol-specified dose. The drug can be diluted with 0.9% normal saline for delivery but the total drug concentration of the solution cannot be below 0.35 mg/ml. It is not to be administered as an i.v. push or bolus injection. At the end of the infusion, flush the line with a sufficient quantity of normal saline.

Nivolumab vials must be stored at a temperature of 2° C to 8° C and should be protected from light and freezing. If stored in a glass front refrigerator, vials should be stored in the carton. Recommended safety measures for preparation and handling of nivolumab include laboratory coats and gloves.

For details on prepared drug storage and use time of nivolumab under room temperature/light and refrigeration, please refer to the nivolumab Investigator Brochure section for "Recommended Storage and Use Conditions" and/or pharmacy reference sheets.

Care must be taken to assure sterility of the prepared solution as the product does not contain any anti-microbial preservative or bacteriostatic agent. No incompatibilities between nivolumab and polyolefin bags have been observed.

Ipilimumab

Ipilimumab is to be administered as a 90-minute i.v. infusion, using a volumetric pump with a 0.2 to 1.2 micron in-line filter at the protocol-specified dose. The drug can be diluted with 0.9% normal saline or 5% Dextrose Injection to concentrations between 1 mg/mL and 4 mg/mL. It is not to be administered as an i.v. push or bolus injection. Care must be taken to assure sterility of the prepared solutions, since the drug product does not contain any antimicrobial preservatives or bacteriostatic agents.

The nivolumab infusion must be promptly followed by a saline flush to clear the line of nivolumab before starting the ipilimumab infusion.

Ipilimumab injection can be used for i.v. administration without dilution after transferring to a PVC (polyvinyl chloride), non-PVC/non-DEHP (di-2-ethylhexyl-phthalate) or glass containers and is stable for 24 hours at 2-8 ºC or room temperature/room light (RT/RL). For ipilimumab storage instructions, refer to ipilimumab Investigator Brochure and/or pharmacy reference sheets.

Recommended safety measures for preparation and handling include protective clothing, gloves, and safety cabinets.

5.2.2 Dose Modifications, Interruptions and delays criteria for Combo Immuno

Dose delay criteria

Dose delay criteria apply for all drug-related adverse events (regardless of whether or not the event is attributed to nivolumab, ipilimumab, or both). All study drugs must be delayed until treatment can resume

Nivolumab and ipilimumab administration should be delayed for the following:

- Any Grade ≥ 2 non-skin, drug-related adverse event, with the following exceptions:
  - Grade 2 drug-related fatigue or laboratory abnormalities do not require a treatment delay
- Any Grade 3 skin, drug-related adverse event
- Any Grade 3 drug-related laboratory abnormality, with the following exceptions for AST, ALT, or total bilirubin:
  - If a subject has a baseline AST, ALT, or total bilirubin that is within normal limits, delay dosing for drug-related Grade ≥2 toxicity
  - If a subject has baseline AST, ALT, or total bilirubin within the Grade 1 toxicity range, delay dosing for drug-related Grade ≥3 toxicity
- Any adverse event, laboratory abnormality, or intercurrent illness which, in the judgment of the investigator, warrants delaying the dose of study medication.

Because of the potential for clinically meaningful nivolumab or ipilimumab related AEs requiring early recognition and prompt intervention, management algorithms have been developed for suspected pulmonary toxicity, GI, hepatotoxicity, endocrinopathy, skin toxicity, neurological toxicity and nephrotoxicity.

In order to standardize the management across the Combo Immuno therapy, for the overlapping adverse event management algorithms present in both the nivolumab and ipilimumab Investigator's Brochure (GI, hepatic, and endocrine algorithms), the recommendations are to follow the nivolumab Investigator's Brochure adverse event algorithms as opposed to the ipilimumab Investigator's Brochure algorithms. Therefore, the algorithms recommended for utilization of nivolumab and ipilimumab are included in Appendix 3.

Dose modifications criteria

Dose reductions or dose escalations are not permitted for both nivolumab and ipilimumab.

Criteria to resume treatment

Subjects may resume treatment with study drug when the drug-related AE(s) resolve to Grade ≤ 1 or baseline value, with the following exceptions:

- Subjects may resume treatment in the presence of Grade 2 fatigue
- Subjects who have not experienced a Grade 3 drug-related skin AE may resume treatment in the presence of Grade 2 skin toxicity
- Subjects with baseline Grade 1 AST/ALT or total bilirubin who require dose delays for reasons other than a 2-grade shift in AST/ALT or total bilirubin may resume treatment in the presence of Grade 2 AST/ALT OR total bilirubin
- Subjects with combined Grade 2 AST/ALT AND total bilirubin values meeting discontinuation parameters (see below) should have treatment permanently discontinued
- Drug-related pulmonary toxicity, diarrhoea, or colitis, must have resolved to baseline before treatment is resumed
- Drug-related endocrinopathies adequately controlled with only physiologic hormone replacement may resume treatment

If the criteria to resume treatment are met, the subject should restart treatment at the next scheduled time-point per protocol. However, if the treatment is delayed past the next scheduled time-point per protocol, the next scheduled time-point will be delayed until dosing resumes.

If treatment is delayed > 6 weeks, the subject must be permanently discontinued from study therapy, except as specified below.

Discontinuation criteria

Treatment with nivolumab and ipilimumab should be permanently discontinued for the following:

- Any Grade 2 drug-related uveitis or eye pain or blurred vision that does not respond to topical therapy and does not improve to Grade 1 severity within the re-treatment period OR requires systemic treatment
- Any Grade 3 non-skin, drug-related adverse event lasting > 7 days, with the following exceptions
    - Grade 3 drug-related uveitis, pneumonitis, bronchospasm, diarrhoea, colitis, neurologic toxicity, hypersensitivity reaction, or infusion reaction of any duration requires discontinuation
    - Grade 3 drug-related laboratory abnormalities do not require treatment discontinuation except:

a) Grade 3 drug-related thrombocytopenia > 7 days or associated with bleeding requires discontinuation

b) Any drug-related liver function test (LFT) abnormality that meets the following criteria require discontinuation:

  - AST or ALT > 8 x ULN
  - Total bilirubin > 5 x ULN
  - Concurrent AST or ALT > 3 x ULN and total bilirubin > 2 x ULN

- Any Grade 4 drug-related adverse event or laboratory abnormality, except for the following events which do not require discontinuation:

  - Isolated Grade 4 amylase or lipase abnormalities that are not associated with symptoms or clinical manifestations of pancreatitis and decrease to < Grade 4 within 1 week of onset.

  - Isolated Grade 4 electrolyte imbalances/abnormalities that are not associated with clinical sequelae and are corrected with supplementation/appropriate management within 72 hours of their onset

- Any dosing interruption lasting > 6 weeks with the following exceptions:

  - Dosing interruptions to allow for prolonged steroid tapers to manage drug-related adverse events are allowed. Tumor assessments should continue as per protocol even if dosing is interrupted.

  - Dosing interruptions > 6 weeks that occur for non-drug-related reasons may be allowed. Tumor assessments should continue as per protocol even if dosing is interrupted.

- Any adverse event, laboratory abnormality, or intercurrent illness which, in the judgment of the Investigator, presents a substantial clinical risk to the subject with continued nivolumab or ipilimumab dosing.

- After 2 years of nivolumab/ipilimumab dosing, to reduce the risk of long-term toxicity. Indeed, no evidence that more than 2 years of checkpoint blockade is needed in metastatic melanoma. Patients who finish 2 years in complete response or even partial response have a very high long-term duration of remission. Also, efficacy data in patients with high-grade immune-related adverse events from single-agent checkpoint inhibition who stop treatment before 2 years are the same of those who complete 2 years of therapy, and patients receiving ipilimumab/nivolumab who stop treatment due to high-grade immune-related events and receive immune suppressants do as well as—or better than—those who complete 2 years of therapy (CheckMate 067 and KEYNOTE-001 KEYNOTE-006, CheckMate 153).

Patients who discontinued permanently the nivolumab/ipilimumab treatment can be followed up until progression disease and remain in the SECOMBIT study unless in the judgment of the Investigator, presents a substantial clinical risk to the subject with the study treatments.

Treatment of Nivolumab- or Ipilimumab-Related Infusion Reactions

Since nivolumab and ipilimumab contain only human immunoglobulin protein sequences, it is unlikely to be immunogenic and to induce infusion or hypersensitivity reactions. However, if such a reaction were to occur, it might manifest with fever, chills, rigors, headache, rash, pruritis, arthralgias, hypo- or hypertension, bronchospasm, or other symptoms. All Grade 3 or 4 infusion reactions should be reported as a SAE if criteria are met. Infusion reactions should be graded according to NCI CTCAE (version 4.03) guidelines.

Treatment recommendations are provided below and may be modified based on local treatment standards and guidelines as appropriate:

For Grade 1 symptoms: (Mild reaction; infusion interruption not indicated; intervention not indicated)

Remain at bedside and monitor subject until recovery from symptoms. The following prophylactic premedications are recommended for future infusions: diphenhydramine 50 mg (or equivalent) and/or paracetamol 325 to 1000 mg (acetaminophen) at least 30 minutes before additional nivolumab administrations.

For Grade 2 symptoms: (Moderate reaction requires therapy or infusion interruption but responds promptly to symptomatic treatment [eg, antihistamines, non-steroidal anti-inflammatory drugs, narcotics, corticosteroids, bronchodilators, i.v. fluids]; prophylactic medications indicated for ≤24 hours).

Stop the nivolumab or ipilimumab infusion, begin an i.v. infusion of normal saline, and treat the subject with diphenhydramine 50 mg i.v. (or equivalent) and/or paracetamol 325 to 1000 mg (acetaminophen);remain at bedside and monitor subject until resolution of symptoms.

Corticosteroid or bronchodilator therapy may also be administered as appropriate. If the infusion is interrupted, then restart the infusion at 50% of the original infusion rate when symptoms resolve; if no further complications ensue after 30 minutes, the rate may be increased to 100% of the original infusion rate. Monitor subject closely. If symptoms recur then no further nivolumab or ipilimumab will be administered at that visit. Administer diphenhydramine 50 mg i.v. and remain at bedside and monitor the subject until resolution of symptoms. The amount of study drug infused must be recorded on the eCRF. The following prophylactic premedications are recommended for future infusions: diphenhydramine 50 mg (or equivalent) and/or paracetamol 325 to 1000 mg (acetaminophen) should be administered at least 30 minutes before additional nivolumab or ipilimumab

administrations. If necessary, corticosteroids (recommended dose: up to 25 mg of i.v. hydrocortisone or equivalent) may be used.

For Grade 3 or Grade 4 symptoms: (Severe reaction, Grade 3: prolonged [i.e., not rapidly responsive to symptomatic medication and/or brief interruption of infusion]; recurrence of symptoms following initial improvement; hospitalization indicated for other clinical sequelae [e.g., renal impairment, pulmonary infiltrates]). Grade 4: (life-threatening; pressure or ventilator support indicated).

Immediately discontinue infusion of nivolumab or ipilimumab. Begin an i.v. infusion of normal saline, and treat the subject as follows. Recommend bronchodilators, epinephrine 0.2 to 1 mg of a 1:1,000 solution for subcutaneous administration or 0.1 to 0.25 mg of a 1:10,000 solution injected slowly for i.v. administration, and/or diphenhydramine 50 mg i.v. with methylprednisolone 100 mg i.v. (or equivalent), as needed. Subject should be monitored until the investigator is comfortable that the symptoms will not recur. Nivolumab or ipilimumab will be permanently discontinued. Investigators should follow their institutional guidelines for the treatment of anaphylaxis. Remain at bedside and monitor subject until recovery from symptoms. In the case of late-occurring hypersensitivity symptoms (e.g., appearance of a localized or generalized pruritus within 1 week after treatment), symptomatic treatment may be given (e.g., oral antihistamine, or corticosteroids).

5.2.3 Formulation, Packaging and Labelling of Combo Immuno

Nivolumab will be made available as cartons each containing 5 or 10 vials. Ipilimumab will be made available as cartons each containing 4 vials.

Study drug packaging will bear a label with the identification required by local law, the protocol number, drug identification and dosage.

Nivolumab and ipilimumab will be administered open label. Nivolumab and ipilimumab will be required to be labelled locally as per local SOPs and regulations.

Medication labels (nivolumab, ipilimumab) will be in the local language and comply with the legal requirements of each country in which the study will be conducted.

5.3 Accountability, assessment of compliance and destruction of the drugs

Combo Target

Accountability and patient compliance for Combo Target will be assessed by maintaining adequate “drug dispensing” and return records.

These records must contain the following information:

- Documentation of drug shipments received (date received, quantity and batch number);
- Disposition of unused study drug not dispensed to patient.

A Drug Dispensing Log must be kept current and should contain the following information:

- Identification code of the patient to whom the study medication was dispensed;
- Date(s), quantity and batch number of the study medication dispensed to the patient;
- Date(s), quantity and batch number of the study medication returned by the patient.

Patients' compliance will be assessed by maintaining adequate study drug dispensing records. Patients will be asked to return all used and unused drug supply containers (of both LGX818 and MEK162) at any visit as a measure of compliance. The Investigator is responsible for ensuring that dosing is administered in compliance with the protocol.

All supplies, including partially used or empty containers and copies of the dispensing and inventory logs, must be returned to the Monitor at the end of the study, unless alternate destruction has been authorized by the Sponsor, or required by local or institutional regulations.

Study treatments must be received by designated personnel at the study site, handled and stored safely and properly, and kept in a secured location to which only the Investigator and designated site personnel have access.

Upon receipt, the study drugs should be stored according to the instructions specified on the drug labels and Investigator's Brochure for LGX818 and MEK162. Study medication is to be stored in a secure locked area while under the responsibility of the Investigator. Receipt and dispensing of study medication must be recorded by an authorized person at the Investigator's site.

Records of drug formulation, batch number, and number of blisters/bottles dispensed must be recorded in the pharmacy study file.

Combo Immuno

Study treatments must be received by designated personnel at the study site, handled and stored safely and properly, and kept in a secured location to which only the Investigator and designated site personnel have access.

Upon receipt, the study drugs should be stored according to the instructions specified on the drug labels and Investigator's Brochure for nivolumab and ipilimumab. Study medication is to be stored in a secure locked area while under the responsibility of the Investigator. Receipt and dispensing of study medication must be recorded by an authorized person at the Investigator's site.

Nivolumab and ipilimumab such as partially used study drug containers, vials and syringes may be destroyed on site.

Any unused study drugs can only be destroyed after being inspected and reconciled by the responsible Study Monitor unless study drug containers must be immediately destroyed as required for safety, or to meet local regulations (e.g., cytotoxics or biologics).

On-site destruction is allowed provided the following minimal standards are met:

- On-site disposal practices must not expose humans to risks from the drug.
- On-site disposal practices and procedures are in agreement with applicable laws and regulations, including any special requirements for controlled or hazardous substances.
- Written procedures for on-site disposal are available and followed. The procedures must be filed with the site's SOPs and a copy provided to the study sponsor upon request.
- Records are maintained that allow for traceability of each container, including the date disposed of, quantity disposed, and identification of the person disposing the containers. The method of disposal, i.e., incinerator, licensed sanitary landfill, or licensed waste disposal vendor must be documented.
- Accountability and disposal records are complete, updated, and available for the Monitor to review throughout the clinical trial period.

If conditions for destruction cannot be met the responsible Study Monitor will make arrangements for return of study drug.

It is the investigator's responsibility to arrange for disposal of all empty containers, provided that procedures for proper disposal have been established according to applicable state, local, and institutional guidelines and procedures, and provided that appropriate records of disposal are kept.

5.4 Treatment after the End of the Study

Study drugs will not be available to subjects after the study has concluded. However, if further treatment is required, local standard of care will apply.

6. STATISTICAL CONSIDERATIONS

6.1 Study Endpoints

OS is primary endpoint of the study. OS will be calculated from the date of randomization until the date of death from any cause. When survival information is not available at the time of data analysis, the patient will be censored using the last date where it was known the patient was alive.

Secondary endpoints of the study will be:

- Total PFS, calculated from the date of randomization until the date of the second progression (i.e. the progression to second treatment); any progression or death will be considered as an event if patient cannot complete treatment sequence;
- 3 years PFS rate, calculated from the date of randomization;
- Percentage of patients alive at 3 years;
- Best overall response rate (BORR), defined as the best response designation, as determined by the investigator, recorded between the date of randomization and the date of objectively documented progression per RECIST version 1.1 criteria;
- Duration of response (DoR), calculated as the time from the date of first documented response (CR or PR) until the date of the first documented progression or death due to underlying cancer. If a patient with a CR or PR has no progression or death due to underlying cancer, the patient is censored at the date of last adequate tumor assessment;
- Biological markers (biomarkers study).

6.2 Sample Size and Analysis Populations

6.2.1 Sample size

This study is designed as a phase II, randomized trial with no formal comparative test. The sample size is discussed for the primary endpoint Overall Survival (OS).

For each arm, a single-stage design as described by A'Hern (*A'Hern, 2001*) will be used.

We have assumed a median PFS of about 10 months for the combo target therapy (LGX818/MEK162) (*Mc Arthur et al, 2013*) and a similar value for the combo immunotherapy (ipilimumab/nivolumab) derived from the aggregate clinical activity rate of 65% (*Wolchock et al, 2013*) which, using an exponential distribution for PFS, could broadly give a median PFS of about 9.5 months. OS seems to be strictly correlated with total PFS.

The null hypothesis is a median OS time of 15 months (i.e. percentage of surviving patient of 33% at 24 months). The alternative hypothesis is a median OS time of 23 months (i.e. percentage of surviving of 48% at 24 months).

Percentage of surviving at 24 months is derived assuming an OS exponential distribution.

Using an exact 5% one-sided significance test at least 69 patients have to be randomized in each treatment arm when the power of the study is 80%.

For each arm, the strategy will be further investigated if at least 30 patients, alive at 24 months, are observed out of the first 69 patients responding to selection criteria..

Taking in account a 10% drop-out rate, a total of 230 patients will be enrolled to ensure a minimum of 207 randomized patients.

If for all arms the null hypothesis will be rejected the best sequence will be identified considering secondary endpoints, mainly total PFS.

6.2.2 Analysis Populations

Baseline is defined as the last valid visit or day before the treatment start.

The following populations are defined for this study:

All Enrolled Set

All screened subjects who have been enrolled.

Intention To Treat (ITT) set

All randomized patients will be considered for the Intention-To-Treat population (ITT)

Safety Set

All patients of the ITT population receiving at least one dose of the study medication.

Per Protocol Population

The per protocol (PP) population refers to all randomized subjects who were successfully randomized, treated, and stayed on their assigned treatment with no major protocol deviation.

6.2.3 Statistical analysis

Data handling

The Overall Survival (OS) will be calculated as the date of randomization until the date of death from any cause.Any patient not know to have died at the time of data analysis will be censored at the time of the last recorded date on which the patient was know to be alive.

Total Progression Free Survival (TPFS) will be calculated from the date of randomization until the date of the second progression (i.e. the progression to second treatment); any progression or death will be considered as an event if patient cannot complete treatment sequence.

Duration of response (DoR) will be calculated as the time from the date of first documented response (Cr5 o PR) until the date of the first documented progression or death due to underlying cancer. If a patient with CR o PR has no progression or death due to underlying cancer, the patient will be censored at the date of the last adeguate tumor assessment.

Statistical analysis

A comprehensive Statistical Analysis Plan (SAP) will be prepared before database lock.

Deviations from methods described in this paragraph might appear.

In case of such deviations the reason for the deviation will be stated in the statistical analysis plan.

Analysis of efficacy endpoints will be performed in the ITT population whereas the safety analysis will be performed in the Safety Population.

For the primary endpoint a one-sided test will be used. The other tests and 95% confidence intervals will be two-sided.

No comparative tests between the three arms will be perfomed and results will be presented as descriptive statistic. Site related differences will be evaluated.

Continuous variables will be summarized by descriptive statistics (number of cases, mean, standard deviation, median, minimum, maximum, first and third quartile). Categorical variables will be summarized using counts of patients and percentages 95% confidence interval will be employed, unless otherwise specified.

The time-dependent endpoints will be analyzed according to the Kaplan-Meier method. Medians with 95% confidence intervals will be derived from the K-M curves and presenting time-dependent endpoints as K-M plot (with a 95% CI over time).

Cox's proportional hazard model will be used to assess the impact of know prognostic factors and treatment assigned.

The list of the covariates to be included in the Cox's model will be presented and clinically justified in the statistical analysis plan.

The ORR will be calculated as the percentage of ITT population patients who have a CRo o PR before any evidence of progression (as defined by RECIST).

A 95% confidence interval (CI) will be derived for the ORR using Wilson score intervals (CIs for a single proportion).

Percentage of patients alive at 3 years will be reported using Wilson score intervals.

Adverse events are those with start date beyond or equal to the informed consent date,

Analysis includes adverse events with starting date until 30 days after the last study drug dose intake.

All adverse event will be assigned to a Preferred Term (PT) and will be classified by primary System Organ Class (SOC) according to MedDRA thesaurus version 18 or higher.

Adverse events will be reported on a per-patient basis within Preferred Term. This mean that even if a patient will be report the same event repeatedly (i.e., events mapped to the preferred term) the event will be counted only once.

In the latter case, the event will be assigned the worst CTCAE severity and the stroingest relationship to the study drug. The earliest date will regard as start date of the event and the latest date will regard as stop date of the event. Adverse events will be assessed accorind to CTCAE version 4.03.

Appropriate summaries of these data will be presented.

Safety and tolerability will be assessed in terms of AEs, laboratory data, ECG data, vitals signs and weight, which will be collected for all patients. AEs (both in terms of MedDRA preferred terms and CTCAE grade), laboratory data, ECG data, vital signs and weight will be listes individually by patient and summarized by treatment received. ECG changes will be summarized for each treatment group.

Vital signs data will be listes for each patient and changes in vital signs will be summarized for each treatment group.

Previous and concomitant medications will be coded using the ATC dictionary, latest version.

Changes from baseline in EQ-5D and QLQ-C30 total score will be summarized by means of descriptive statistical methods.

6.3 Study Duration

This is a multicenter study that will be conducted in 30 sites located in Europe. It is expected that 230 eligible patients will be enrolled in total for this phase II study by the participating centers to ensure a minimum of 207 evaluable patients.

Treatment duration will be until second PD (2 years estimated). The first patient was enrolled (First Patient First Visit Date) on November 2016, the recruitment will end approximately (Last Patient First Visit) on May 2019, and the Study will end approximately (Last Patient Last Visit) on May 2024. The date of study end is dependent on the clinical course of the disease and may therefore occur earlier than indicated. Patients still on treatment in May 2024 will be treated according to clinical practice.

7. SAFETY INSTRUCTIONS AND GUIDANCE

7.1 Warning and precautions

No evidence available at the time of the submission of this study protocol indicated that special warnings or precautions were appropriate, other than those noted in the provided Investigator's Brochure for all the IMPs. Additional safety information collected between Investigator's Brochure updates will be communicated in the form of Investigator Notifications. This information will be included in the patient informed consent and should be discussed with the patient during the study as needed.

The recommendations to be followed for the management of toxicities and adverse events are detailed in Section 5.1.2 (Combo Target) and in Section 5.2.2 (Combo Immuno).

Adverse events of special interest for LGX818 and MEK162 are detailed in Section 7.5.2.

7.2 Adverse Events and Laboratory Abnormalities

7.2.1 Clinical Adverse Events (AEs)

According to the International Conference of Harmonization (ICH), an AE is any untoward medical occurrence in a patient or clinical investigation subject (patient) administered a pharmaceutical product and which does not necessarily have a causal relationship with this treatment. An AE can therefore be any unfavourable and unintended sign [including an abnormal laboratory finding], symptom, or disease temporally associated with the use of a medicinal (investigational) product, whether or not considered related to the medicinal (investigational) product. Pre-existing conditions which worsen during a study are to be reported as AEs.

7.2.2 Intensity

Intensity of all AEs will be graded according to the NCI Common Terminology Criteria for Adverse Events v4.03 (CTCAE v 4.03 most recent sub-version) on a five-point scale (Grade 1 to 5) and reported in detail on the CRF.

AEs not listed on the CTCAE should be graded as follows:

CTC Grade Equivalent To:

- Grade 1: Mild Discomfort noticed but no disruption of normal daily activity

- Grade 2: Moderate Discomfort sufficient to reduce or affect daily activity; no treatment or medical intervention is indicated although this could improve the overall well-being or symptoms of the patient
- Grade 3: Severe Inability to work or perform normal daily activity; treatment or medical intervention is indicated in order to improve the overall wellbeing or symptoms; delaying the onset of treatment is not putting the survival of the patient at direct risk
- Grade 4: Life threatening/disabling. An immediate threat to life or leading to a permanent mental or physical condition that prevents work or performing normal daily activities; treatment or medical intervention is required in order to maintain survival
- Grade 5: AE resulting in death

7.2.3 Drug AE relationship

The causality relationship of study drug to the AE will be assessed by the Investigator as either: *Related or Not related.*

If there is a reasonable suspected causal relationship to the study medication, i.e. there are facts (evidence) or arguments to suggest a causal relationship, drug-event relationship should be assessed as Yes.

The following criteria should be considered in order to assess the relationship as Yes:

- Reasonable temporal association with drug administration
- It may or may not have been produced by the patient's clinical state, environmental or toxic factors, or other modes of therapy administered to the patient
- Known response pattern to suspected drug
- Disappears or decreases on cessation or reduction in dose
- Reappears on re-challenge

The following criteria should be considered in order to assess the relationship as No:

- It does not follow a reasonable temporal sequence from administration of the drug
- It may readily have been produced by the patient's clinical state, environmental or toxic factors, or other modes of therapy administered to the patient
- It does not follow a known pattern of response to the suspected drug
- It does not reappear or worsen when the drug is re-administered

7.2.4 Serious Adverse Events

A serious adverse event (SAE) is any experience that suggests a significant hazard, contraindication, side effect or precaution. It is any AE that at any dose fulfils at least one of the following criteria:

- is fatal (results in death; NOTE: death is an outcome, not an event)
- is Life-Threatening (NOTE: the term "Life-Threatening" refers to an event in which the patient was at immediate risk of death at the time of the event; it does not refer to an event which could hypothetically have caused a death if it had been more severe).
- requires in-patient hospitalization or prolongation of existing hospitalization;
- results in persistent or significant disability/incapacity;
- is a congenital anomaly/birth defect;
- is a cancer;
- is associated with an overdose;
- is another Important Medical Event (any important adverse events/reactions that is not immediately life-threatening or do not result in death or hospitalization, but may jeopardize the subject or may require medically significant or requires intervention to prevent one or other of the outcomes listed above). Examples of such events include, but are not limited to, intensive treatment in an emergency room or at home for allergic bronchospasm, blood dyscrasias or convulsions that do not result in hospitalization). Potential drug induced liver injury (DILI) is also considered an important medical event.

Note: The term sudden death should be used only when the cause is of a cardiac origin as per standard definition. The terms death and sudden death are clearly distinct and must not be used interchangeably. The study will comply with all local regulatory requirements and adhere to the full requirements of the ICH Guideline for Clinical Safety Data Management, Definitions and Standards for Expedited Reporting.

Overdose

An overdose is a significant variation above the recommended/scheduled dosage for a product. In this current trial an overdose of the IMPs is any dose higher than the dose specified in the in Sections 5.1.1 and 5.2.1 of this protocol.

Planned Hospitalization

A hospitalization planned by the subject prior to signing the informed consent form (ICF) is considered a therapeutic intervention and not the result of a new SAE and should be recorded as medical history. If the

planned hospitalization or procedure is executed as planned, the record in the subject's medical history is considered complete. However, if the event/condition worsens during the trial, it must be reported as an AE.

7.2.5 Progression of Underlying Malignancy

Progression of underlying malignancy is not reported as an AE if it is clearly consistent with the suspected progression of the underlying cancer as defined by RECIST criteria, or other criteria as determined by protocol. Hospitalization due solely to the progression of underlying malignancy should NOT be reported as a SAE. Clinical symptoms of progression may be reported as AEs if the symptom cannot be determined as exclusively due to the progression of the underlying malignancy or does not fit the expected pattern of progression for the disease under study.

Symptomatic deterioration may occur in some patients. In this case, progression is evident in the patient's clinical symptoms, but is not supported by the tumor measurements. Or, the disease progression is so evident that the Investigator may elect not to perform further disease assessments. In such cases, the determination of clinical progression is based on symptomatic deterioration. These determinations should be a rare exception as every effort should be made to document the objective progression of underlying malignancy. If there is any uncertainty about an AE being due only to the disease under study, it should be reported as an AE or SAE.

Deaths due to progression of underlying malignancy occurred during the treatment period should be reported in the AE eCRF.

Deaths due to progression of underlying malignancy occurred during the follow-up period should be reported in the Long Term FU eCRF.

7.3 Treatment and Follow-up of AEs (100 days post discontinuation of study drugs)

After 100 days from the last dose of study drugs continue to follow-up AEs as follows:

- For Related AEs, follow until one of the following occurs:
  - Resolved or improved to baseline;
  - Relationship is reassessed as unrelated;
  - Death;
  - Start of new anti-cancer regimen;
  - Investigator confirms that no further improvement can be expected;
  - Clinical or safety data will no longer be collected, or final database closure.
- For Unrelated severe or life-threatening AEs, follow until one of the following occurs:

- Resolved or improved to baseline;
- Severity improved to Grade 2;
- Death;
- Start of new anti-cancer regimen;
- Investigator confirms that no further improvement can be expected;
- Clinical or safety data will no longer be collected, or final database closure.

Unrelated Grade 1 or Grade 2 AEs: follow-up until 100 days after last dose of study drugs and every 12 weeks until 60 months from randomization for long term follow-up. The final outcome of each AE must be recorded on the CRF.

7.4 Laboratory Test abnormalities

Laboratory test results will be recorded on the laboratory results form of the eCRF or appear on electronically produced laboratory reports submitted directly from the central laboratory, if applicable.

Any laboratory result abnormality fulfilling the criteria for a SAE should be reported as such, in addition to being recorded as an AE in the CRF.

Any treatment-emergent abnormal laboratory result which is clinically significant, i.e., meeting one or more of the following conditions, should be recorded as a single diagnosis on the AE page in the eCRF:

- Accompanied by clinical symptoms
- Leading to a change in study medication (e.g. dose modification, interruption or permanent discontinuation)
- Requiring a change in concomitant therapy (e.g. addition of, interruption of, discontinuation of, or any other change in a concomitant medication, therapy or treatment)

This applies to any protocol and non-protocol specified safety and efficacy laboratory result from tests performed after the first dose of study medication, which falls outside the laboratory reference range and meets the clinical significance criteria.

7.4.1 Follow-up of Abnormal Laboratory Test Values

In the event of medically significant unexplained abnormal laboratory test values, the tests should be repeated and followed up until they have returned to the normal range and/or an adequate explanation of the abnormality is found. If a clear explanation is established it should be recorded on the eCRF.

7.5 Handling of Safety Parameters

7.5.1 Reporting of AEs

Information about all adverse events, whether volunteered by the patient, discovered by Investigator questioning, or detected through physical examination, laboratory test or other means, will be collected on the Adverse Event CRF page, documented in the patient's medical records, and followed as appropriate.

The NCI CTC-AE (Version 4.03) will be used to evaluate the clinical safety of the treatment in this study. Patients will be assessed for AEs at each clinical visit and as necessary throughout the study.

The following Adverse Events of Special Interest (AESI) assessed as non-serious must be recorded as an AE in the CRF.

LGX818

As a result of signals observed from previous LGX818 studies, several AEs requiring a close follow-up were identified. For each category, selected AEs similar in nature, will be identified and grouped:

- Palmar-plantar erythrodysaesthesia syndrome
- Rash and related events
- Squamous cell carcinoma (SCC), keratoacanthoma (KA) and any other suspicious skin lesions.

MEK162

As a result of signals observed from previous MEK162 studies, several AEs requiring a close follow-up were identified. For each category, selected AEs similar in nature, will be identified and grouped:

- Ocular/visual events
- Retinal vein occlusion
- Rash and related events
- Peripheral/generalized edema/anasarca
- Serum creatin-kinase (CK) elevation
- Cardiac failure related events
- Hepatic events.

7.5.2 Reporting of SAEs (immediately reportable)

Any clinical AE, or abnormal laboratory test value assessed as serious (as defined above), or pregnancy case, occurred during the course of this study from the enrolment visit (start of study screening procedures), including

long term follow-up, must be reported to Sponsor and PV Manufacturer within *24 hours* of the Investigator becoming aware of the event (expedited reporting).

The Investigator must complete the SAE form and forward it to Pharmacovigilance Office:

**Emergency contact**	**Clinical Research Technology srl**<br>Pharmacovigilance Office	Tel: +39 089.301545<br>**Fax: +39 089.7724155**<br>**e-mail: pvg@cr-technology.com**
	**Medical Monitor**<br>Dr. Antonio M. Grimaldi<br>Istituto Nazionale dei Tumori, Fondazione "G. Pascale"<br>U.O.C. Melanoma, Immunoterapia Oncologica e Terapie Innovative<br>Via M. Semmola 80131 - Naples	Tel: +39 081 5903 236<br>Fax: +39 081 5903 841<br>E-mail: dott.a.m.grimaldi@gmail.com
	**Dr. Paolo A. Ascierto**<br>Istituto Nazionale dei Tumori, Fondazione "G. Pascale"<br>U.O.C. Melanoma, Immunoterapia Oncologica e Terapie Innovative<br>Via M. Semmola 80131 - Naples	Tel: +39 081 5903 236<br>Fax: +39 081 5903 841<br>Email paolo.ascierto@gmail.com

In addition, report of Adverse Events will be done, according to current local law, to local Health Authorities.

From the first administration of IMPs, all SAEs must be reported. All SAEs related to the administered drugs MUST be collected and reported regardless of the time elapsed from the last study drugs administration, even if the study has been closed. Suspected Unexpected Serious Adverse Reactions (SUSARs) are reported to Investigators at each site and associated IRB/IEC when the following conditions occur:

- The event must be a SAE
- There must be a certain probability that the event is an adverse reaction to the administered drugs
- The adverse reaction must be unexpected, that is to say, not foreseen in the Investigator's Brochure of IMPs. When all patients at a particular site are off treatment as defined by the protocol:
  - only individual SUSAR reports originating in that particular trial will be forwarded to the site and associated IRB/IEC on an expedited basis
  - individual SUSARs considered to be a significant safety issue and/or which result in recommending a change to the ICF, will be reported in an expedited manner to all Investigators and IRBs/IECs
- SUSAR reports originating from other trials using the same IMP will be provided as six monthly SUSAR Reports to Investigators and IRBs/IECs where long-term follow-up studies are carried out, unless they are considered significant.

Unrelated SAEs must be collected and reported during the study and for up to 100 days after the last dose of study medication and every 12 weeks until 60 months from randomization for long-term follow up.

This study adheres to the definition and reporting requirements of ICH Guideline for Clinical Safety Data Management, Definitions and Standards for Expedited Reporting.

7.5.3 Pregnancy

Investigators will counsel WOCBP and male subjects who are sexually active with WOCBP on the importance of pregnancy prevention and the implications of an unexpected pregnancy.

Investigators will advise WOCBP and male subjects who are sexually active with WOCBP on the use of highly effective methods of contraception. Highly effective methods of contraception have a failure rate of < 1% when used consistently and correctly.

As stated in the inclusion criteria, at a minimum, subjects must agree to the use of two methods of contraception, with one method being highly effective and the other method being either highly effective or less effective as listed below:

Highly effective methods of contraception have a failure rate of <1% when used consistently and correctly. WOCBP and female partners of male subjects, who are WOCBP, are expected to use one of the highly effective methods of contraception listed below. Male subjects must inform their female partners who are WOCBP of the contraceptive requirements of the protocol and are expected to adhere to using contraception with their partner. Contraception methods are as follows:

1. Progestogen only hormonal contraception associated with inhibition of ovulation.
2. Hormonal methods of contraception including oral contraceptive pills containing combined estrogen + progesterone, vaginal ring, injectables, implants and intrauterine devices (IUDs) such as Mirena.
3. Nonhormonal IUDs, such as ParaGard.
4. Bilateral tubal occlusion.
5. Vasectomised partner with documented azoospermia 90 days after procedure. Vasectomised partner is a highly effective birth control method provided that partner is the sole sexual partner of the WOCBP trial participant and that the vasectomized partner has received medical assessment of the surgical success.
6. Intrauterine hormone-releasing system (IUS).

7. Complete abstinence:

    a. Complete abstinence is defined as the complete avoidance of heterosexual intercourse (refer to Glossary of Terms).

    b. Complete abstinence is an acceptable form of contraception for all study drugs and must be used throughout the duration of the study treatment (plus 5 half-lives of the investigational drug plus 30 days).

    c. It is not necessary to use any other method of contraception when complete abstinence is elected.

    d. Subjects who choose complete abstinence must continue to have pregnancy tests, as specified in Section 3.5.3.

    e. Acceptable alternate methods of highly effective contraception must be discussed in the event that the subject chooses to forego complete abstinence.

    f. The reliability of sexual abstinence needs to be evaluated in relation to the duration of the clinical trial and the preferred and usual lifestyle of the subject.

Less effective methods of contraception:

1. Diaphragm with spermicide
2. Cervical cap with spermicide
3. Vaginal sponge with spermicide
4. Male or female condom with or without spermicide*
5. Progestogen-only oral hormonal contraception, where inhibition of ovulation is not the primary mode of action.

*A male and a female condom must not be used together.

Unacceptable methods of contraception:

1. Periodic abstinence (calendar, symptothermal, post-ovulation methods)
2. Withdrawal (coitus interruptus)
3. Spermicide only
4. Lactation amenorrhea method (LAM)

Female patients must be instructed to stop taking IMP and immediately inform the Investigator if become pregnant during the study. The Investigator should report all pregnancies within 24 hours to the Sponsor, using the *Clinical Trial Pregnancy Reporting Form*. The Investigator should counsel the patient, discuss the risks of continuing with the pregnancy and the possible effects on the fetus. Serum pregnancy will be performed at screening; every 6 (± 1) weeks during the treatment period Combo-Immuno and every 4 (± 1) weeks during the treatment Combo-Target; at end of study treatment and follow-up visit up to 31 weeks after the last dose of nivolumab/ipilimumab. Pregnancies occurring up to 6 months after the completion of the study medication must also be reported to the Investigator. Pregnancies occurring in the partner of a male patient participating in the study should be reported to the Investigator and the Sponsor. The partner should be counselled, the risks of continuing the pregnancy discussed, as well as the possible effects on the foetus. Monitoring of the patient should continue until conclusion of the pregnancy.

8. DATA COLLECTION AND MANAGEMENT

8.1 Data confidentiality

Information about study subjects will be kept confidential and managed under the applicable laws and regulations. The data collection system for this study uses built-in security features to encrypt all data for transmission in both directions, preventing unauthorized access to confidential participant information. Access to the system will be controlled by a sequence of individually assigned user identification codes and passwords, made available only to authorized personnel who have completed prerequisite training.

8.2 Site monitoring

Before study initiation, at a site initiation visit or at an investigator’s meeting, Sponsor personnel (or designated CRO) will review the protocol and CRFs with the investigators and their staff. During the study, the field monitor will visit the site regularly to check the completeness of patient records, the accuracy of entries on the CRFs, the adherence to the protocol to Good Clinical Practice, the progress of enrolment, and to ensure that study treatment is being stored, dispensed, and accounted according to specifications. Key study personnel must be available to assist the field monitor during these visits. The investigator must maintain source documents for each patient in the study, consisting of case and visit notes (hospital or clinic medical records) containing demographic and medical information, laboratory data, electrocardiograms and the results of any other tests or assessments. All information recorded on CRFs must be traceable to source documents in the patient's file. The investigator must also keep the original signed informed consent form (a signed copy is given to the patient). The investigator must give the monitor access to all relevant source documents to confirm their consistency with the CRF entries.

The Sponsor monitoring standards require full verification for the presence of informed consent, adherence to the inclusion/exclusion criteria and documentation of SAEs.

8.3 Data collection

This study will use an Electronic Data Capture (EDC) system (eClinical platform provided by Clinical Research Technology). The designated investigator staff will enter the data required by the protocol into the Electronic Case Report Forms (eCRF). The eCRFs have been built using eClinical platform provided by Clinical Research Technology, a fully validated secure web-enabled software that conforms to FDA requirements. Investigator site staff will not be given access to the EDC system until they have been trained. Automatic validation programs check for data discrepancies in the eCRFs allow modification or verification of the entered data by the investigator staff. The Principal Investigator is responsible for assuring that the data entered into eCRF is complete, accurate, and that entry and updates are performed in a timely manner.

8.4 Database management and quality control

The Sponsor personnel (or designated CRO) will review the data entered by investigational staff for completeness and accuracy. Electronic data queries stating the nature of the problem and requesting clarification will be created for discrepancies and missing values and sent to the investigational site via the EDC system. Designated investigator site staff is required to respond promptly to queries and to make any necessary changes to the data. Concomitant treatments entered into the database will be coded using the WHO Drug Reference List, which employs the Anatomical Therapeutic Chemical classification system. Medical history/current medical conditions and adverse events will be coded using the Medical dictionary for regulatory activities (MedDRA) terminology. The occurrence of any protocol violations will be determined. After the data has been verified to be complete and accurate, the database will be declared locked. Authorization is required prior to making any database changes to locked data, by joint written agreement between the Biostatistics and Data Management and the Sponsor.

8.5 Data Monitoring Committee

The Data Monitoring Committee (DMC) will be responsible for safeguarding the interests of trial participants, assessing the safety and efficacy of the interventions during the trial, and for monitoring the overall conduct of the clinical trial. The DMC will provide recommendations about stopping or continuing the trial. To contribute to enhancing the integrity of the trial, the DMC may also formulate recommendations relating to the selection/recruitment/retention of participants, their management, improving adherence to protocol-specified

regimens and retention of participants, and the procedures for data management and quality control. The DMC will be advisory to the clinical trial leadership group, the Steering Committee (SC).

The DMC membership and their specialties are as follows:

- Prof. Sanjiv S. Agarwala, MD - Chief of Medical Oncology & Haematology at St. Luke's Cancer Center and Professor of Medicine at Temple University School of Medicine, Philadelphia, USA (Chair of DMC)
- Dr. Paul Nathan MBBS, PhD, FRCP - Consultant Medical Oncologist at Mount Vernon Cancer Centre, Rickmansworth, UK,
- Dr. Michael Postow, MD – Consultant Medical Oncologist, Melanoma-Sarcoma Oncology Service, Memorial Sloan Kettering Cancer Centre, New York, USA.

9. ETHICAL CONSIDERATION

9.1 Regulatory and ethical compliance

This clinical study was designed, shall be implemented and reported in accordance with the ICH Harmonized Tripartite Guidelines for Good Clinical Practice, with applicable local regulations (including European Directive 2001/20/EC), and with the ethical principles laid down in the Declaration of Helsinki.

9.2 Responsibilities of the investigator and IEC

The protocol and the proposed informed consent form must be reviewed and approved by Independent Ethics Committee (IEC) of all participating centers before study start.

9.3 Informed consent procedures

Eligible patients may only be included in the study after providing written (witnessed, where required by law or regulation), IEC-approved informed. Informed consent must be obtained before conducting any study-specific procedures (i.e. all of the procedures described in the protocol). The process of obtaining informed consent should be documented in the patient source documents. The date when a subject’s Informed Consent was actually obtained will be captured in their CRFs.

9.4 Publication of study protocol and results

The key design elements of this protocol will be posted in the publicly accessible database clinicaltrials.gov. The Investigators assure that results of this study will be submitted for publication and reported in scientific meetings.

9.5 Study documentation, record keeping and retention of documents

Each participating site will maintain appropriate medical and research records for this trial, in compliance with Section 4.9 of the ICH E6 GCP, and regulatory and institutional requirements for the protection of confidentiality of subjects. As part of participating in a Fondazione Melanoma-sponsored study, each site will permit authorized representatives of the sponsor(s) and regulatory agencies to examine (and when required by applicable law, to copy) clinical records for the purposes of quality assurance reviews, audits and evaluation of the study safety and progress. Source data are all information, original records of clinical findings, observations, or other activities in a clinical trial necessary for the reconstruction and evaluation of the trial. Examples of these original documents and data records include, but are not limited to, hospital records, clinical and office charts, laboratory notes, memoranda, subjects’ diaries or evaluation checklists, pharmacy dispensing records, recorded data from automated instruments, copies or transcriptions certified after verification as being accurate and complete,

microfiches, photographic negatives, microfilm or magnetic media, x-rays, and subject files and records kept at the pharmacy, at the laboratories, and medico-technical departments involved in the clinical trial. Data collection is the responsibility of the clinical trial staff at the site under the supervision of the site Principal Investigator. The study case report form (CRF) is the primary data collection instrument for the study. The investigator should ensure the accuracy, completeness, legibility, and timeliness of the data reported in the CRFs and all other required reports. Data reported on the CRF, that are derived from source documents, should be consistent with the source documents or the discrepancies should be explained. All data requested on the CRF must be recorded. Any missing data must be explained. For electronic CRFs an audit trail will be maintained by the system. The investigator/institution should maintain the trial documents as specified in Essential Documents for the Conduct of a Clinical Trial (ICH E6 Section 8) and as required by applicable regulations and/or guidelines. The investigator/institution should take measures to prevent accidental or premature destruction of these documents. Essential documents (written and electronic) should be retained for a period of not less than seven (7) years from the completion of the Clinical Trial unless Sponsor provides written permission to dispose of them or, requires their retention for an additional period of time because of applicable laws, regulations and/or guidelines

9.6 Confidentiality of study documents and patient records

The investigator must ensure anonymity of the patients; patients must not be identified by names in any documents submitted to the Sponsor. Signed informed consent forms and patient enrolment log must be kept strictly confidential to enable patient identification at the site.

9.7 Audits and inspections

Source data/documents must be available to inspections by the Sponsor or designee or Health Authorities.

9.8 Financial disclosures

Financial disclosures should be provided by study personnel who is directly involved in the treatment or evaluation of patients at the site - prior to study start.

10. PROTOCOL ADHERENCE

The study shall be conducted as described in this approved protocol. All revisions to the protocol must be discussed with, and be prepared by, the Sponsor. The investigator should not implement any deviation or change to the protocol without prior review and documented approval/favorable opinion from the IEC of an amendment, except where necessary to eliminate an immediate hazard(s) to study subjects.

If a deviation or change to a protocol is implemented to eliminate an immediate hazard(s) prior to obtaining IEC approval/favorable opinion, as soon as possible the deviation or change will be submitted to:

- IEC for review and approval/favorable opinion
- Sponsor
- Regulatory Authority(ies), if required by local regulations.

10.1 Amendments to the protocol

Any change or addition to the protocol can only be made in a written protocol amendment that must be approved by the Sponsor, Health Authorities where required, and the IEC. Only amendments that are required for patient safety may be implemented prior to IEC approval. Notwithstanding the need for approval of formal protocol amendments, the investigator is expected to take any immediate action required for the safety of any patient included in this study, even if this action represents a deviation from the protocol. In such cases, the Sponsor should be notified of this action and the IEC at the study site should be informed within 10 working days.

11. REFERENCES

Aaronson NK, Ahmedzai S, Bergman B et al. The European Organization for Research and Treatment of Cancer QLQ-C30: a quality-of-life instrument for use in international clinical trials in oncology. J Natl Cancer Inst 1993; 85: 365-76.

A'Hern RP. Sample size tables for exact single-stage phase II designs. Statist Med 2001, 20: 859-66.

American Cancer Society. Cancer Facts & Figures 2012. Atlanta: American Cancer Society; 2012.

Ascierto PA, Simeone E, Sileni VC et al. Sequential treatment with ipilimumab and BRAF inhibitors in patients with metastatic melanoma: data from the Italian cohort of the ipilimumab expanded access program. Cancer Invest 2014; 32 (4): 144-9.

Ascierto PA, Schadendorf D, Berking C, et al. MEK162 for patients with advanced melanoma harbouring NRAS or Val600 BRAF mutations: a non-randomised, open-label phase 2 study. Lancet Oncol 2013; 14: 249 – 256.

Ascierto PA, Simeone E, Giannarelli D et al. Sequencing of BRAF inhibitors and ipilimumab in patients with metastatic melanoma: a possible algorithm for clinical use. J Transl Med 2012; 10: 107.

Ascierto PA, Simeone E, Grimaldi AM et al. Do BRAF inhibitors select for populations with different disease progression kinetics?J Transl Med 2013; 11: 61.

Brooks R. EuroQoL: the current state of play. Health Policy 1996; 37 (1): 53-72.

Chapman PB, Hauschild A, Robert C et al. Improved survival with vemurafenib in melanoma with BRAF V600E mutation. N Engl J Med 2011; 364:2507-16.

Colombino M, Capone M, Lissia A et al. BRAF/NRAS mutation frequencies among primary tumors and metastases in patients with melanoma. J ClinOncol 2012; 30: 2522-9.

Ferlay J, Shin HR, Bray F et al. Estimates of worldwide burden of cancer in 2008: GLOBOCAN 2008. Int J Cancer. 2010 Dec 15; 127 (12): 2893-917.

Flaherty KT, Infante JR, DaudA et al. Combined BRAF and MEK inhibition in melanoma with BRAF V600 mutations. N Engl J Med 2012; 367 (18): 1694-703.

Flaherty KT, Puzanov I, Kim KB et al. Inhibition of Mutated, Activated BRAF in Metastatic Melanoma. NEJM 2010; 363: 809-19.

Greaves WO, Verma S, Patel KP et al. Frequency and Spectrum of BRAF Mutations in a Retrospective, Single-Institution Study of 1112 Cases of Melanoma. J MolDiagn 2013; 15: 220-6.

Hauschild A, Grob JJ, Demidov LV, et al. Dabrafenib in BRAF-mutated metastatic melanoma: a multicentre, open-label, phase 3 randomised controlled trial. Lancet 2012; 9839: 358-65.

Jakob JA, Bassett RL Jr, Ng CS et al. NRAS mutation status is an independent prognostic factor in metastatic melanoma.Cancer 2012; 118 (16): 4014-23.

Jemal A, Saraiya M, Patel P et al. Recent trends in cutaneous melanoma incidence and death rates in the United States, 1992-2006. J Am AcadDermatol 2011; 65 (5 Suppl 1): S17-25.

Lebbé C, Weber JS, Maio M et al. Survival follow-up and ipilimumab retreatment for patients with advanced melanoma who received ipilimumab in prior phase II studies. Ann Oncol 2014 Sep 10.

LGX818 Investigator's Brochure.

Martinez-Garcia M, Banerji U, Albanell J et al. First-in-human, phase I dose-escalation study of the safety, pharmacokinetics, and pharmacodynamics of RO5126766, a first-in-class dual MEK/RAF inhibitor in patients with solid tumors. Clin Cancer Res 2012; 18 (17): 4806-19.

McArthur G, Gonzalez R, Pavlick A, et al. Vemurafenib (VEM) and MEK inhibitor, cobimetinib (GDC0973), in advanced BRAF V600-mutated melanoma (BRIM-7): dose-exalation and expansion results of a phase Ib study. Eur J Cancer 2013; 49 (Suppl. 2): abstract 3703

MEK162 Investigator's Brochure.

Reilly MC, Zbrozek AS, Dukes EM. The validity and reproducibility of a work productivity and activity impairment instrument. Pharmacoeconomics 1993; 4 (5): 353-65.

Ribas A, Hodi FS, Callahan M et al. Hepatotoxicity with combination of vemurafenib and ipilimumab. N Engl J Med 2013; 368 (14): 1365-6.

Sosman JA, Kim KB, Schuchter L et al. Survival in BRAF V600- mutant advanced melanoma treated with vemurafenib. N Engl J Med 2012; 366:707–14.

Topalian SL, Sznol M, McDermott DF et al. Survival, durable tumor remission, and long-term safety in patients with advanced melanoma receiving nivolumab.J ClinOncol 2014; 32 (10): 1020-30.

Urner-Bloch U, Urner M, Stieger P et al. Transient MEK inhibitor-associated retinopathy in metastatic melanoma. Ann Oncol 2014; 25 (7): 1437-41.

Wolchok JD, Kluger H, Callahan MK et al. Nivolumab plus ipilimumab in advanced melanoma. N Engl J Med 2013; 369 (2): 122-33.

12. APPENDIX

I. Recommended guidelines for the management of study drug (LGX818 and MEK162) induced skin toxicity

Clinical judgment and experience of the treating physician should guide the management plan of each patient. In general, the following interventions are in addition to the rash dosing guidelines in Table 11 of the protocol:

- Prophylaxis of skin toxicity to be initiated 24 hours prior to the first treatment with study drug or later as needed
- Application of topical agents to the most commonly affected skin areas such as face, scalp, neck, upper chest and upper back

Topical agents include non-oily sunscreen (PABA free, SPF ≥ 30, UVA/UVB protection), topical steroids (preferably mometasone cream and topical erythromycin evening or topical pimocrolimus

Note: Topical agents should be applied on a daily basis starting on Day 1 of study treatment or 24 hours prior to the first dose, and more often as needed.

- Possibly oral doxycycline (100 mg daily) for the first 2-3 weeks of study drug administration.

Other effective medications are antihistamines, other topical corticosteroids, other topical antibiotics and low-dose systemic corticosteroids.

The treatment algorithm based on CTCAE grade is as follows:

Mild rash (CTCAE Grade 1)

- Consider prophylactic rash treatment if not already started
- Topical or other topical corticosteroid (i.e. mometasone cream) and/or topical antibiotic (i.e. erythromycin 2%) are recommended.
- The patient should be reassessed within a maximum of 2 weeks or as per investigator opinion.

Moderate rash (CTCAE Grade 2)

- Use of topical erythromycin or clindamycin (1%) plus topical mometasone or pimecrolimus cream (1%) plus oral antibiotics such as: lymecycline (408 mg OD), doxycycline (100 mg BID) or minocycline (50 to 100 mg OD).
- Although there has been no evidence of phototoxicity or photosensitivity in patients being treated with LGX818 or MEK162, doxycycline (or minocycline as secondline) should be used with thorough UV protection (i.e., avoidance of direct exposure to sunlight, use of sunscreen and sunglasses, etc.).
- Use of acitretin is not recommended

Severe rash (CTCAE Grade 3-4)

CTCAE Grade 3

- In addition to the interventions recommended for moderate rash, consider oral prednisolone at a dose of 0.5 mg/kg. Upon improvement, taper the dose in a stepwise manner (25 mg for 7 days, subsequently decreasing the dose by 5 mg/day every day).
- Alternatively, in addition to the interventions recommended for moderate rash, consider oral isotretinoin (low doses, i.e. 0.3 to 0.5 mg/kg)
- Use of acitretin is not recommended

CTCAE Grade 4

- Immediately discontinue the patient from study drug and treat the patient with oral and topical medications (see recommendation CTCAE Grade 3).

Symptomatic treatment:

It is strongly recommended that patients who develop rash/skin toxicities receive symptomatic treatment:

- For pruritic lesions, use cool compresses and oral antihistaminic agents
- For fissuring, use Monsel's solution, silver nitrate, or zinc oxide cream. If not sufficient use mild steroid ointments or combinations of steroids and antibiotics
- For desquamation, use emollients with mild pH 5/neutral (best containing urea 10%)
- For paronychia, antiseptic bath and local potent corticosteroids, use oral antibiotics and if no improvement is seen, refer to a dermatologist or surgeon
- For infected lesions, obtain bacterial and fungal cultures and treat with topical or systemic antibiotics based on sensitivity of culture

II. Recommended guidelines for the management of MEK162 induced diarrhoea

Proactively investigate for occurrence of diarrhoea and educate patients

a. Remind patients at each visit to contact the Investigator immediately upon the first sign of loose stool or symptoms of abdominal pain. Additionally, at each study visit, each patient should be asked regarding occurrence of diarrhoea or diarrhoea-related symptoms. If the patient has symptoms, the patient should be asked regarding the actions taken for these symptoms and re-instruct if indicated

b. The patients should be instructed on dietary modifications and on early warning signs of diarrhoea and potentially life-threatening illnesses (e.g. severe cramping might be a sign for severe diarrhoea, fever with diarrhoea might be a sign for infection, fever and dizziness on standing might be a sign for shock)

c. Patients should be educated about what to report to the Investigator (i.e., number of stools, stool composition, stool volume)

Anti-diarrhoea therapy

In order to effectively manage diarrhoea and mitigate the escalation in severity or duration of diarrhoea, patient education as outlined above as well as proper management of diarrhoea is important.

Management of diarrhoea should be instituted at the first sign of abdominal cramping, loose stools or overt diarrhoea. All concomitant therapies used for treatment of diarrhoea must be recorded on the Concomitant Medications eCRF. It is recommended that patients be provided loperamide tablets and are instructed on the use of loperamide at on the first day of MEK162 treatment. In addition to the MEK162 induced diarrhea dosing guidelines in Table 11 of the protocol, these instructions should be provided at each visit and the site should ensure that the patient understands the instructions

Explain the frequency of diarrhoea and its relationship to NCI CTCAE grading.

Determine if diarrhoea is complicated or uncomplicated.

Rule out other or concomitant causes.

These may include:

- Infection with Candida, Salmonella, Clostridium difficile, Campylobacter, Giardia, Entamoeba and Cryptosporidium species can lead to severe infections in immunosuppressed patients
- Medication-induced diarrhoea
- Malabsorption/lactose intolerance
- Faecal impaction, partial bowel obstruction

For uncomplicated Grade 1/2 diarrhoea

- Stop all lactose-containing products, alcohol and eat frequent small meals that include bananas, rice, applesauce or toast)
- Stop laxatives, bulk fiber and stool softeners
- Stop high-osmolar food supplements
- Drink 8 to 10 large glasses of clear liquids per day
- Consider administration of standard dose of loperamide: initial administration 4 mg, then 2 mg every 4 hours (maximum of 16 mg/day) or after each unformed stool.
- Discontinue loperamide after 12-hours diarrhoea-free (Grade 0) interval.
- If uncomplicated Grade 1 to 2 diarrhoea persists for more than 24 hours, escalate to high dose loperamide: 2 mg every 2 hours (max. of 16 mg/day) or after each unformed stool.

Note: Oral antibiotics may be started as prophylaxis for infections under the discretion of the physician.

- If uncomplicated Grade 1 to 2 diarrhoea persists after 48 hours of treatment with loperamide, discontinue loperamide and begin a second-line agent which can be an opiate (opium tincture or paregoric), octreotide acetate or steroid (budesonide)

For complicated Grade 1/2 diarrhoea or any Grade 3 to 4 diarrhea

- The patient must call the investigator immediately
- If loperamide has not been initiated, initiate loperamide immediately. Initial administration 4 mg, then 2 mg every 4 hours (maximum of 16 mg/day) or after each unformed stool.
- Administer IV fluids and electrolytes as needed. In case of severe dehydration, replace loperamide by octreotide.
- Monitor/continue IV fluids and antibiotics as needed. Intervention should be continued until the patient is diarrhoea free for at least 24 hours.

Hospitalization may need to be considered.

III. Recommended algorithms for use of nivolumab and ipilimumab

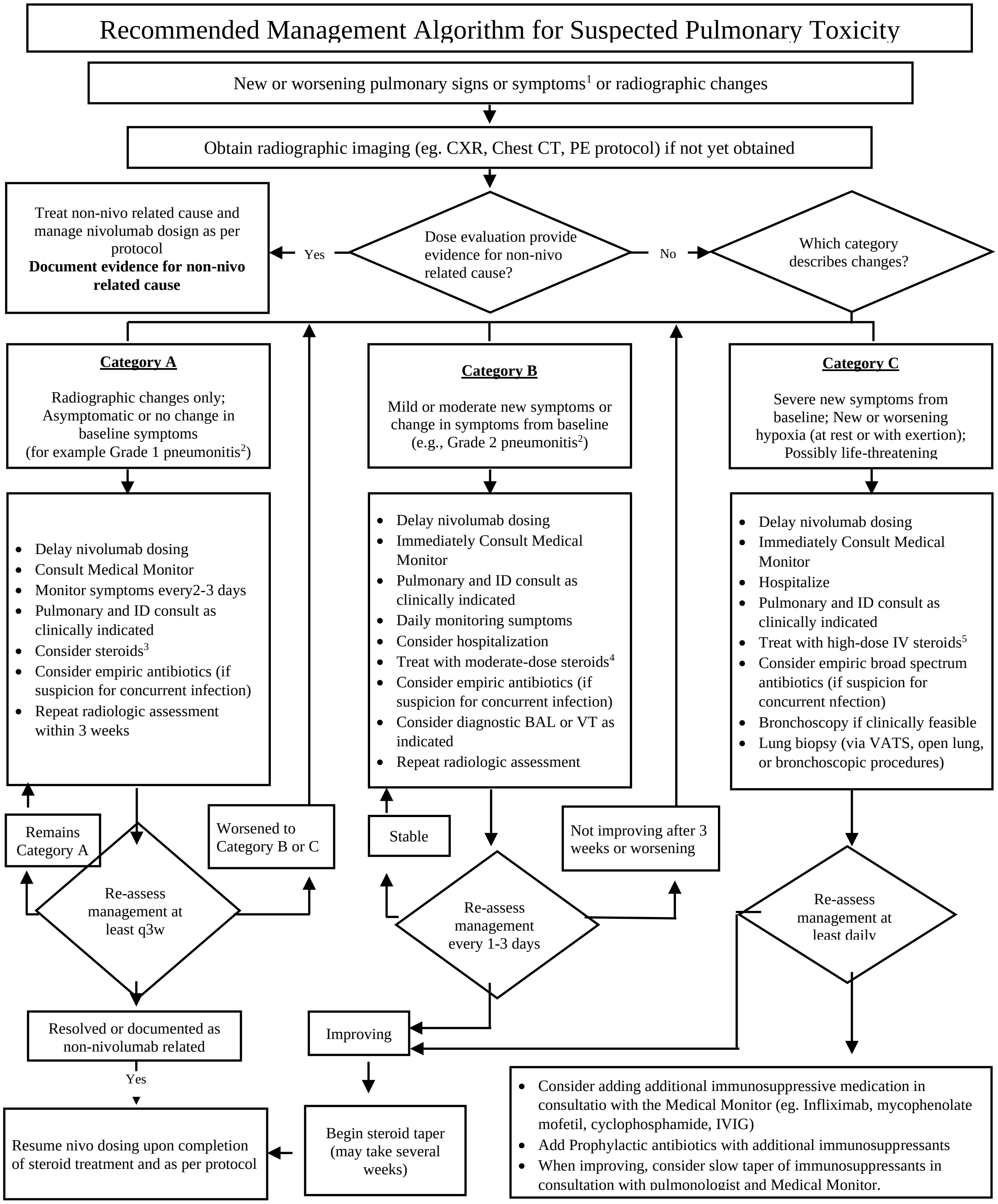

Footnotes
1 Signs and synptoms include dyspnea, cough, hypoxia and other respiratory compliants
2 Grading as per NCI CTCAE 4.03
3 Recommended initial costicosteroid regimen for category A: prednisone 60 mg/day PO or methylprednisone 1 mg/Kg/day IV
4 Recommended initial costicosteroid regimen for category B: prednisone 240 mg/day PO or methylprednisone 4 mg/Kg/day IV
5 Recommended initial costicosteroid regimen for category C: methylprednisone 1 g/Kg/day IV

Recommended Management Algorithm for Diarrhea or Colitis

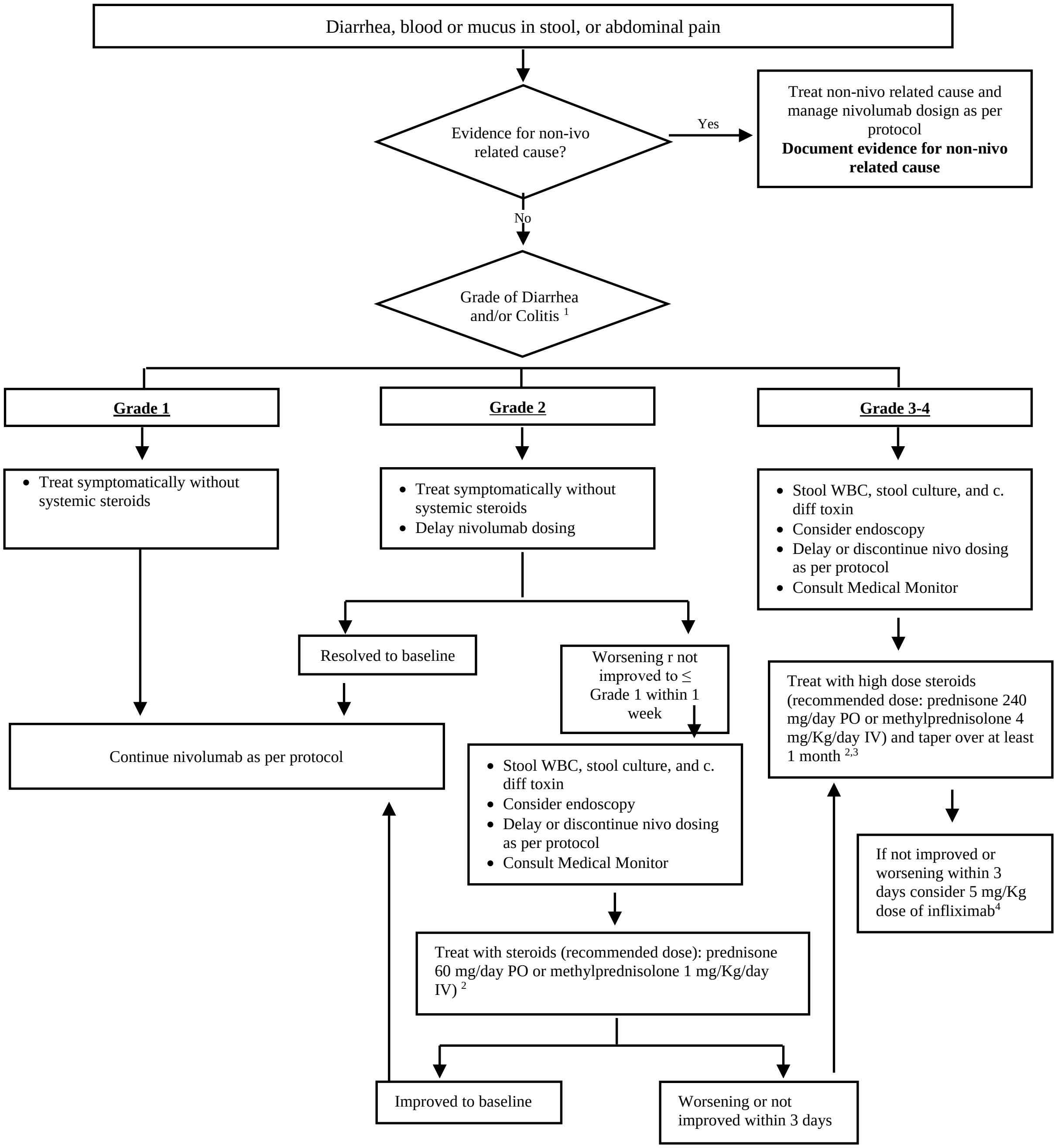

Footnotes
1. Grading as per NCI CTCAE version 4.03. If both diarrhea and colitis are present, manage as per toxicity with higher grade.
2. If infection work-up is positive, do not give steroids, stop following algorithm and treat specific infection.
3. If re-treatment with nivolumab is allowed as per protocol after completion of steroid taper, consult with Medical Monitor if considering re-treatment
4. Do not use infliximab if perforation or sepsis is present

Recommended Management Algorithm for Suspected Endocrinopathy

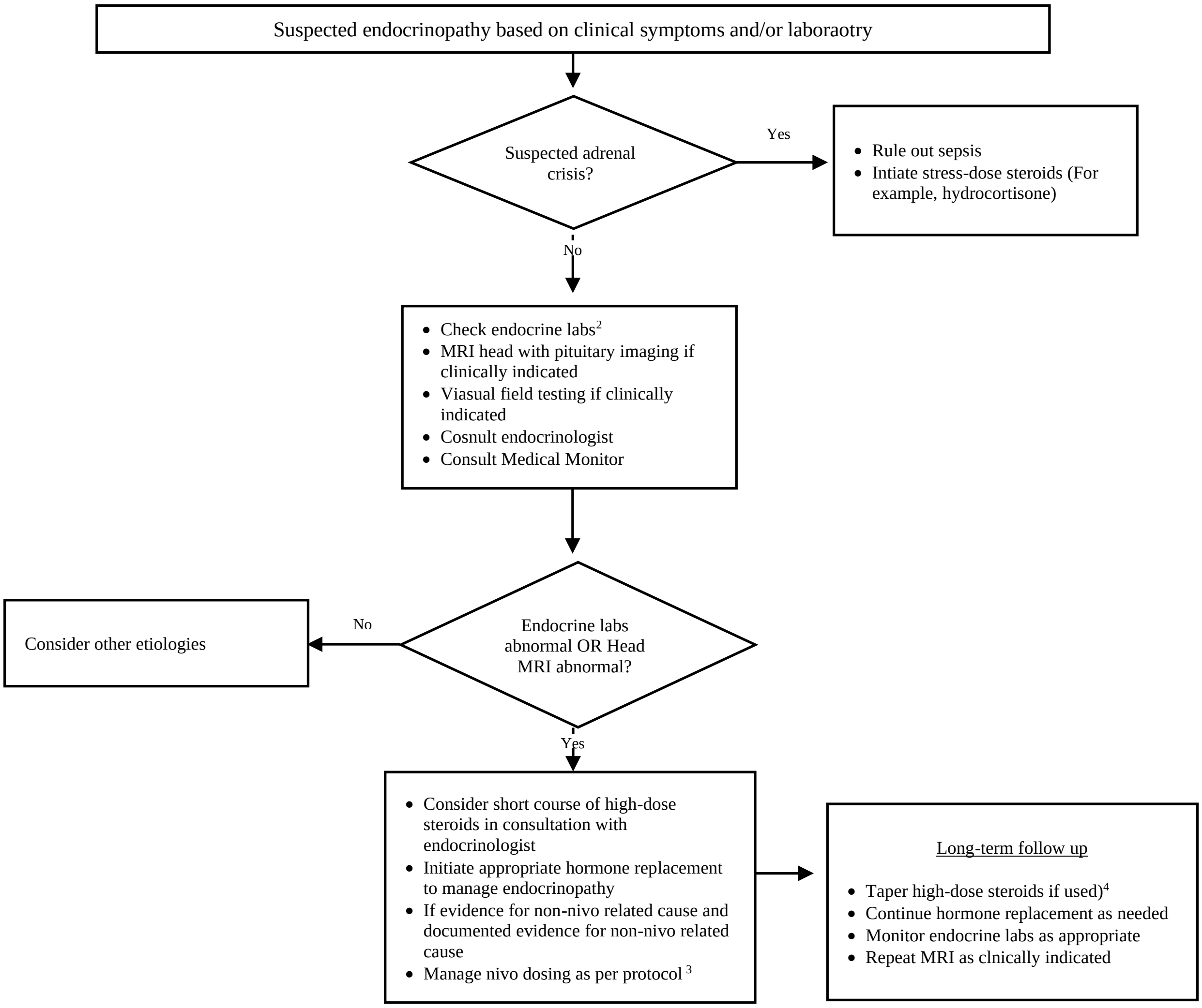

Footnotes
1. Cases have typically been identified through routine monitoring of laboratories or as part of a work-up for symptoms such as fatigue
2. It’s important to draw labs at appropriate times; for example, certain labs should be drawn before giving steroids or at specific times of the day
3. Upon resolution or adequate treatment of endocrinopathy, patients may continue nivolumab dosing with appropriate hormone replacement unless limited by protocol
4. Patients may require chronic steroid replacement to maintain physiologic levels

Recommended Management Algorithm for Suspected Hepatotoxicity

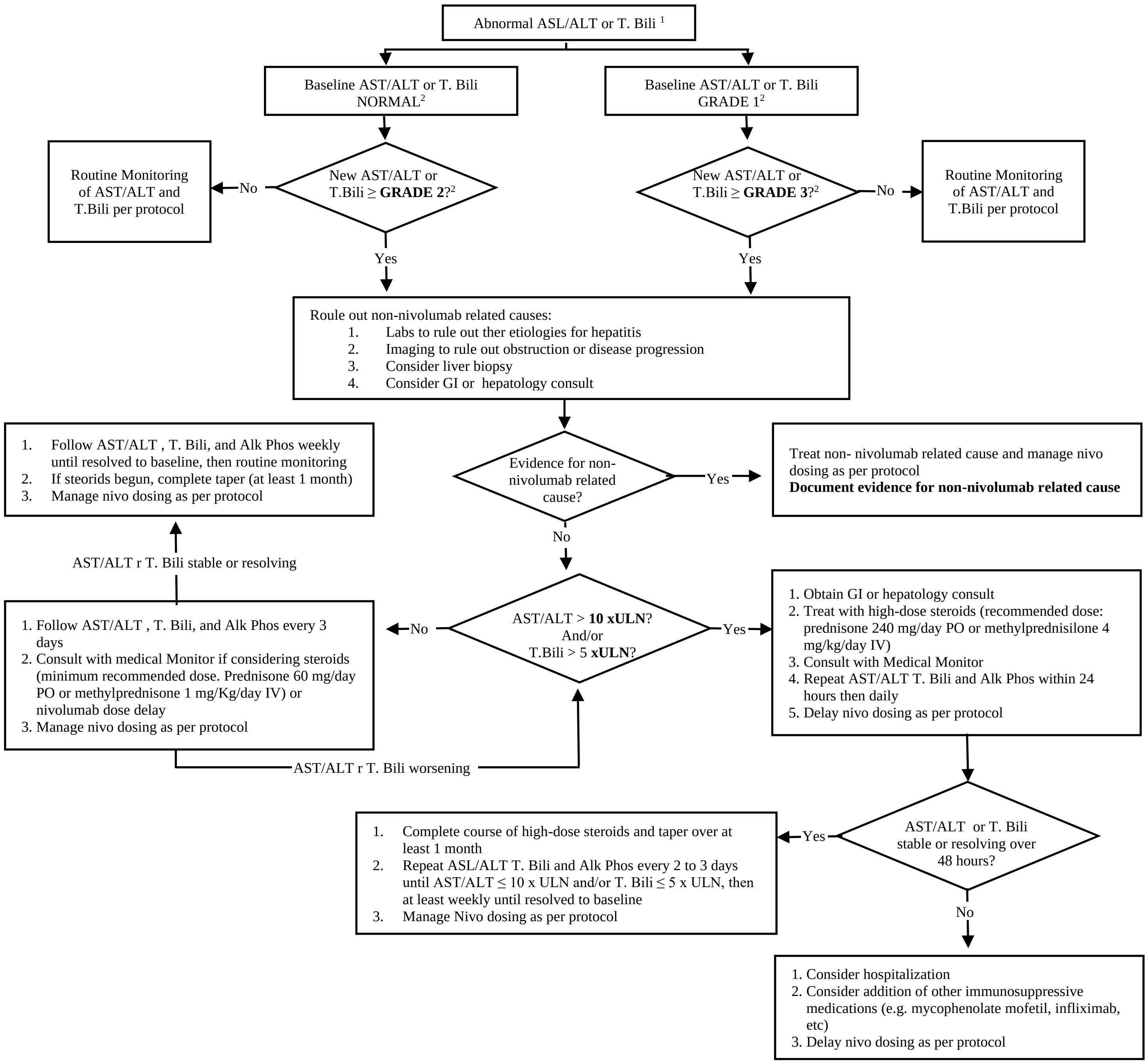

Footnotes

1. If elevations in both AST/ALT and T. Bili are present, the management of nivolumab dosing may be different than if only an isolated AST/ALT of T. Bili abnormality is present and may not be dependent on baseline values. Refer to the specific protocol if concurrent elevations occur
2. Grading as per NCI CTCAE versio 4.03

Recommended Management Algorithm for Nephrotoxicity

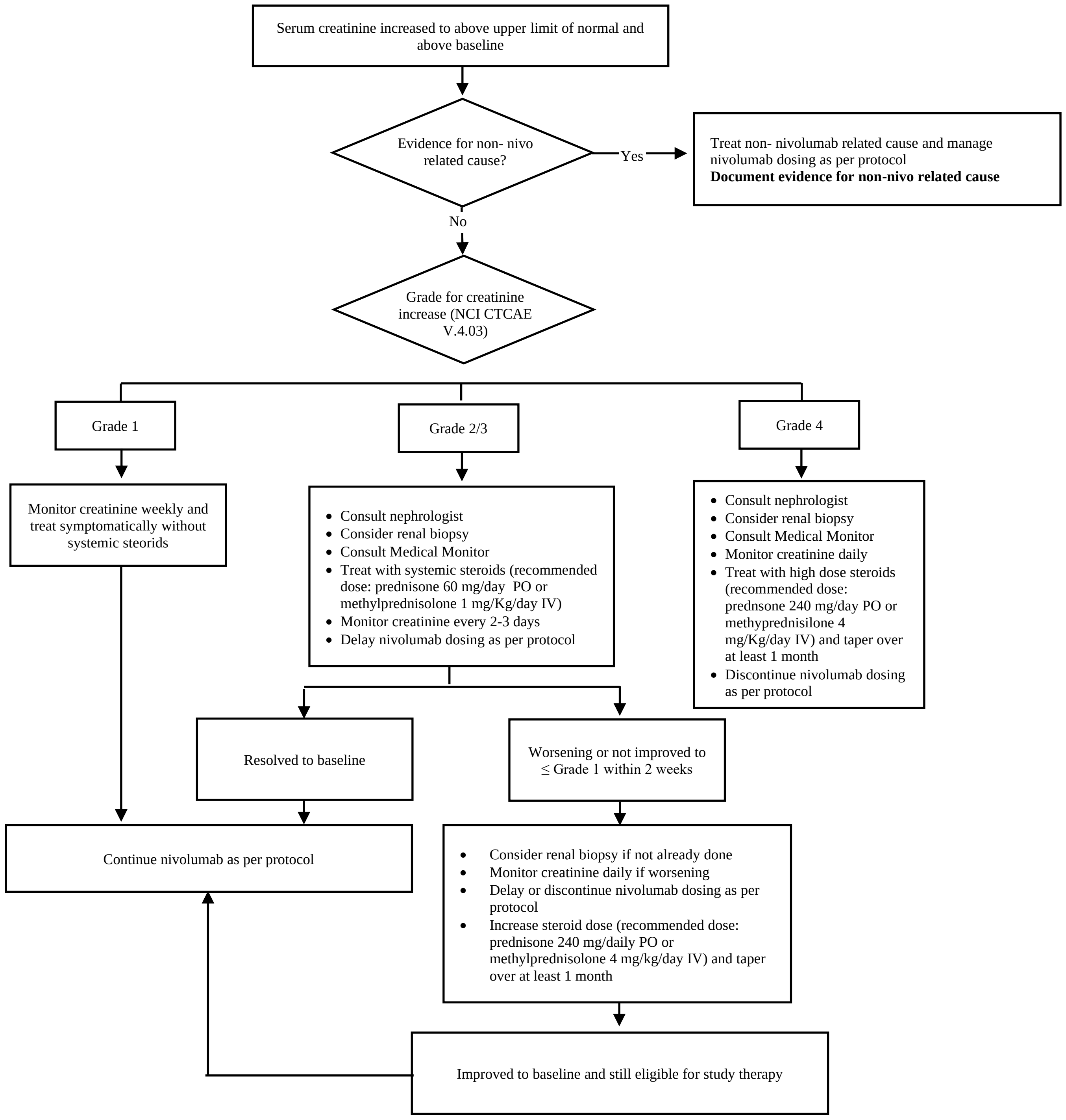

Skin toxicity Management Algorithm

**Severity of skin toxicity**	**Management**	**Follow-up**
Rule out non-inflammatory cause. If non.inflammatory cause, treat accordingly and continue ipilimumab		
Grade 1-2	• Symptomatic therapy,e.g. antihistamine, topical steroids<br>• Continue ipilimumab	**Symptoms resolve**: Continue ipilimuamb<br>**Symptoms persist > 1-2 weeks or recur**:<br>○ Continue ipilimumab<br>○ Moderate to high dose steroids PO (e.g. prednisole 0.5 – 1 mg/Kg/day)<br>○ Once controlled, taper steroids over at least 1 month)
Grade 3-4	• Hold/delay ipilimumab (regardless of relationship)<br>• Take photos of rash<br>• Consider skin biopsy<br>• Dermatology consult<br>• High dose IV steroids (e.g. methylprednisolone 1-2 mg/kg/day)	**Symptoms resolve/ return to grade 1:**<br>○ Taper steroids over the least 1 month<br>○ Resume ipilimumab<br>○ Discontinue if grade 4 toxicity consider related

Patients on IV steroids may be switched to oral corticosteroid (e.g. prednisone) at an equivalent dose at start or tapering or earlier once sustained clinical improvement is observed. Lower bioavailability of oral costicosteroids should be taken into account when switching to the equivalent dose of PO costicosteroids.

Neurological toxicity Management Algorithm

Rule out non-inflammatory cause. If non-inflammatory cause, treat accordingly and continue ipilimumab.
Discontinue ipilimumab for any grade 3-4 motor neuropathy, regardless of relationship.

Severity of neurological toxicity	Management	Follow-up
Grade 1	Continue ipilimumab	Continue monitoring the patient. If symptoms worsen, treat as below
Grade 2	• Hold/ delay ipilimumab<br>• Treat symptoms per local guidelines	Resume ipilimumab when resolved/ grade 1<br>If symptoms worsen, treat as below
Grade 3/4 sensory	• Discontinue ipilimumab if consider related<br>• Obtain neuro consult<br>• Treat symptoms per local guidelines<br>• High dose IV steroids (e.g. 1-2 mg/kg/day methulprenisolone)	Symptoms resolve/return to grade 1:<br>○ Taper steroids over at least 1 month
Grade 3/4 motor	• Discontinue ipilimumab **regardless of relationship**<br>• Obtain neuro consult<br>• Treat symptoms per local guidelines<br>• High dose IV steroids (e.g. 1-2 mg/kg/day methylprednisolone)	Symptoms resolve/return to grade 2:<br>○ Taper steroids over at least 1 month<br>Symptoms do not resolve or progress; atypical presentation:<br>○ Consider IVIg or other immunosuppressive therapies per local guidelines

Patients on IV steroids may be switched to oral corticosteroid (e.g. prednisone) at an equivalent dose at start or tapering or earlier once sustained clinical improvement is observed. Lower bioavailability of oral costicosteroids should be taken into account when switching to the equivalent dose of PO costicosteroids.

IV. EORTC QLQ-C30 (version 3)

We are interested in some things about you and your health. Please answer all of the questions yourself by circling the number that best applies to you. There are no "right" or "wrong" answers. The information that you provide will remain strictly confidential.

Please fill in your initials: |__|__|__|__|

Your birthdate (Day, Month, Year): |__|__|__|__|__|__|__|__|__|

Today's date (Day, Month, Year): 31 |__|__|__|__|__|__|__|__|__|

		Not at All	A little	Quite e Bit	Very Much
1.	Do you have any trouble doing strenuous activities, like carrying a heavy shopping bag or a suitcase?	1	2	3	4
2.	Do you have any trouble taking a long walk?	1	2	3	4
3.	Do you have any trouble taking a short walk outside of the house?	1	2	3	4
4.	Do you need to stay in bed or a chair during the day?	1	2	3	4
5.	Do you need help with eating, dressing, washing yourself or using the toilet?	1	2	3	4

During the past week:

		Not at All	A little	Quite e Bit	Very Much
6.	Were you limited in doing either your work or other daily activities?	1	2	3	4
7.	Were you limited in pursuing your hobbies or other leisure time activities?	1	2	3	4
8.	Were you short of breath?	1	2	3	4
9.	Have you had pain?	1	2	3	4
10.	Did you need to rest?	1	2	3	4
11.	Have you had trouble sleeping?	1	2	3	4
12.	Have you felt weak?	1	2	3	4
13.	Have you lacked appetite?	1	2	3	4
14.	Have you felt nauseated?	1	2	3	4
15.	Have you vomited?	1	2	3	4
16.	Have you been constipated?	1	2	3	4
17.	Have you had diarrhea?	1	2	3	4
18	Were you tired?	1	2	3	4

19.	Did pain interfere with your daily activities?	1	2	3	4
20.	Have you had difficulty in concentrating on things, like reading a newspaper or watching television?	1	2	3	4
21.	Did you feel tense?	1	2	3	4
22.	Did you worry?	1	2	3	4
23	Did you feel irritable?	1	2	3	4
24.	Did you feel depressed?	1	2	3	4
25	Have you had difficulty remembering things?	1	2	3	4
26	Has your physical condition or medical treatment interfered with your family life?	1	2	3	4
27	Has your physical condition or medical treatment interfered with your social activities?	1	2	3	4
28.	Has your physical condition or medical treatment caused you financial difficulties?	1	2	3	4

For the following questions please circle the number between 1 and 7 that best applies to you:

29. How would you rate your overall health during the past week?

1	2	3	4	5	6	7
Very Poor						Excellent

30. How would you rate your overall quality of life during the past week?

1	2	3	4	5	6	7
Very Poor						Excellent

V. EQ-5D

Under each heading, please tick the ONE box that best describes your health TODAY.

MOBILITY
I have no problems in walking about ☐
I have slight problems in walking about ☐
I have moderate problems in walking about ☐
I have severe problems in walking ☐
I am unable to walk about ☐

SELF-CARE
I have no problems washing or dressing myself ☐
I have slight problems washing or dressing myself ☐
I have moderate problems washing or dressing myself ☐
I have severe problems washing or dressing ☐
I am unable to wash or dress myself ☐

USUAL ACTIVITIES *(e.g. work, study, housework, family or leisure activities)*
I have no problems doing my usual activities ☐
I have slight problems doing my usual activities ☐
I have moderate problems doing my usual activities ☐
I have severe problems doing my usual activities ☐
I am unable to do my usual activities ☐

PAIN / DISCOMFORT
I have no pain or discomfort ☐
I have slight pain or discomfort ☐
I have moderate pain or discomfort ☐
I have severe pain or discomfort ☐
I have extreme pain or discomfort ☐

ANXIETY / DEPRESSION
I am not anxious or depressed ☐
I am slightly anxious or depressed ☐
I am moderately anxious or depressed ☐
I am severely anxious or depressed ☐
I am extremely anxious or depressed ☐

We would like to know how good or bad your health is TODAY.

This scale is numbered from 0 to 100.
100 means the best health you can imagine.
0 means the worst health you can imagine.

Mark an X on the scale to indicate how your health is TODAY.

Now, please write the number you marked on the scale in the box below.

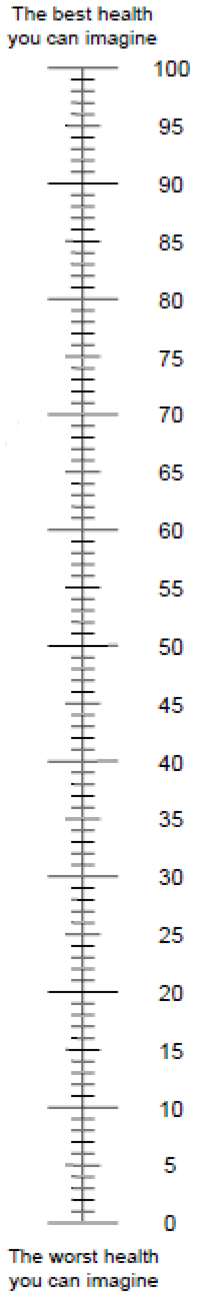

YOUR HEALTH TODAY

VI. WPAI:GH

**Work Productivity and Activity Impairment Questionnaire:
General Health V2.0 (WPAI:GH)**

The following questions ask about the effect of your health problems on your ability to work and perform regular activities. By health problems we mean any physical or emotional problem or symptom. *Please fill in the blanks or circle a number, as indicated.*

1. Are you currently employed (working for pay)? ____ NO ____ YES
   If NO, check "NO" and skip to question 6.

The next questions are about the **past seven days**, not including today.

2. During the past seven days, how many hours did you miss from work because of your health problems? *Include hours you missed on sick days, times you went in late, left early, etc., because of your health problems. Do not include time you missed to participate in this study.*

   _____HOURS

3. During the past seven days, how many hours did you miss from work because of any other reason, such as vacation, holidays, time off to participate in this study?

   _____HOURS

4. During the past seven days, how many hours did you actually work?

   _____HOURS *(If "0", skip to question 6.)*

5. During the past seven days, how much did your health problems affect your productivity while you were working?

Think about days you were limited in the amount or kind of work you could do, days you accomplished less than you would like, or days you could not do your work as carefully as usual. If health problems affected your work only a little, choose a low number. Choose a high number if health problems affected your work a great deal.

Consider only how much health problems affected productivity while you were working.

Health problems had no effect on my work	0	1	2	3	4	5	6	7	8	9	10	Health problems completely prevented me from working

CIRCLE A NUMBER

6. During the past seven days, how much did your health problems affect your ability to do your regular daily activities, other than work at a job?

By regular activities, we mean the usual activities you do, such as work around the house, shopping, childcare, exercising, studying, etc. Think about times you were limited in the amount or kind of activities you could do and times you accomplished less than you would like. If health problems affected your activities only a little, choose a low number. Choose a high number if health problems affected your activities a great deal.

Consider only how much health problems affected your ability to do your regular daily activities, other than work at a job.

Health problems had no effect on my daily activities	0	1	2	3	4	5	6	7	8	9	10	Health problems completely prevented me from doing my daily activities

CIRCLE A NUMBER

VII. ECOG Performance Status

Grade	ECOG
0	Fully active, able to carry on all pre-disease performance without restriction
1	Restricted in physically strenuous activity but ambulatory and able to carry out work of a light or sedentary nature, e.g., light house work, office work
2	Ambulatory and capable of all selfcare but unable to carry out any work activities. Up and about more than 50% of waking hours
3	Capable of only limited selfcare, confined to bed or chair more than 50% of waking hours
4	Completely disabled. Cannot carry on any selfcare. Totally confined to bed or chair
5	Dead
